# Mechanistic Insights into the Propagation Cycle of the Hofmann–Löffler–Freytag Reaction: Halogen vs Hydrogen Atom Transfer

**DOI:** 10.1021/acs.joc.4c02997

**Published:** 2025-04-01

**Authors:** Gabrijel Zubčić, Luka Andrijanić, Iva Džeba, Jiangyang You, Tomislav Friganović, Tomislav Portada, Kristina Pavić, Erim Bešić, Valerije Vrček, Davor Šakić

**Affiliations:** 1 University of Zagreb Faculty of Pharmacy and Biochemistry, Ante Kovačića 1, Zagreb 10000, Croatia; 2 University of Zagreb Faculty of Science, Horvatovac 102 a, Zagreb 10000, Croatia; 3 Ruđer Bošković Institute, Bijenička cesta 54, Zagreb 10000, Croatia

## Abstract

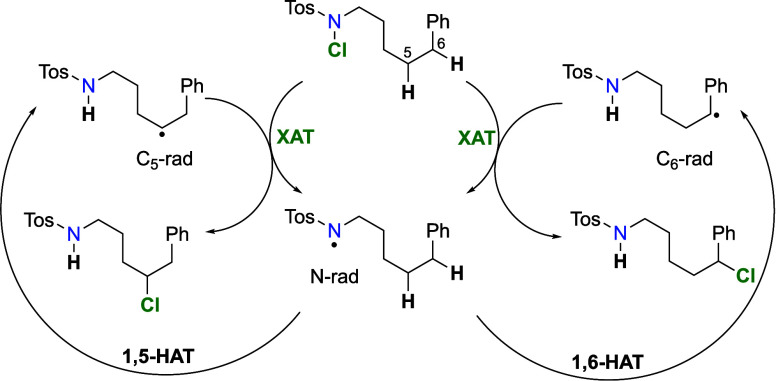

The Hofmann–Löffler–Freytag (HLF) reaction is a method that employs N-chlorinated precursors in radical-mediated rearrangement cycles to synthesize pyrrolidine rings and C–H functionalized products. This study aims to elucidate the mechanism of the propagation cycle, identify the rate-limiting step, and uncover the factors influencing the regioselectivity of the HLF reaction. Combining experimental techniques—laser flash photolysis (LFP), electron paramagnetic resonance (EPR), and nuclear magnetic resonance (NMR)—with computational density functional theory (DFT) calculations and kinetic modeling, we challenge the previous assumption that the hydrogen atom transfer (HAT) step was rate-limiting and regioselectivity was under both thermodynamic and kinetic control. We have identified that the halogen atom transfer (XAT) step in the propagation cycle of the HLF reaction follows pseudo-first-order kinetics and has the largest transition-state barrier. Additionally, we observed that regioselectivity is exclusively controlled by the intramolecular hydrogen atom transfer kinetics, while no thermodynamic preference exists in the formation of C_6_- and C_5_-chlorinated products. Our work predicts how to accelerate the HLF reaction and how we can control the regioselectivity by the smarter selection of substrates based on calculations, which could provide better control of the reaction when implemented in organic synthesis.

## Introduction

**Scheme 1:**
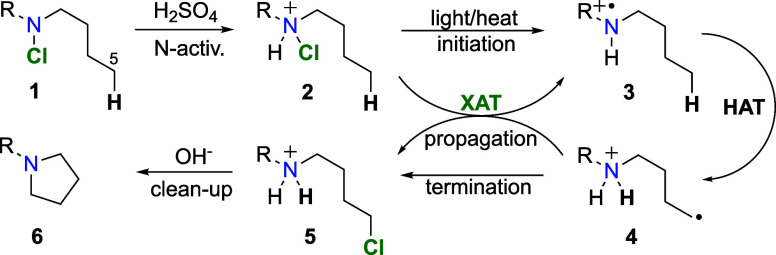
Cyclization of N-Halogenated Amines (the Hofmann–Löffler–Freytag Reaction)^4^

The foundation for the use of N-centered aminyl radicals in organic synthesis, albeit not recognized as such, was laid more than a century ago. In 1881–1885, Hofmann^1^ discovered that treating *N*-bromo-2-propylpiperidine, an *N*-halodialkylamine, with hot sulfuric acid produced a tertiary amine, eventually identified^2^ as octahydroindolizine. Löffler and Freytag^3^ extended the Hofmann reaction to simple secondary amines and discovered it to be a general approach for synthesizing pyrrolidines.^4^ Around 70 years after its discovery, Wawzonek and Helen,^5^ followed by Corey and Hertler,^6^ identified a free radical chain mechanism for this reaction. Upon the activation of *N*-chloroamine **1** with sulfuric acid (Scheme 1), protonated *N*-chloroamine **2** undergoes homolytic cleavage in the presence of heat, light, or initiators. The resulted protonated aminyl radical **3** takes part in an intramolecular hydrogen atom transfer (HAT) abstracting a sterically favorably orientated hydrogen atom to afford, regioselectively, an alkyl radical **4**, which in turn abstracts a chlorine atom to form a chloroalkylammonium ion **5**, which then cyclizes in the presence of a base, providing cyclic tertiary amine **6**.

There are two well-documented modern variants of the Hofmann–Löffler–Freytag Reaction (HLF), one developed by Suarez and co-workers,^7−10^ who used *in situ* halogenation and photochemical activation of *N*-iodoamides to produce cyclization products, and another reported by Corey et al.,^11^ where the required bromoamide precursor was synthesized and characterized in a separate first step. Subsequent irradiation of the bromoamide precursor in CCl_4_ results in the formation of the C_5_-bromo derivative, which can be cyclized to methyl 1-acetyl-3-methylpyrrolidine-2-carboxylate with a 90% yield using a hindered base.

Mechanistic investigations, from the discovery of the HLF reaction until today, point conclusively to a radical chain mechanism involving intramolecular HAT as the first step of the propagation cycle, with halogen atom transfer (XAT) as the second step.^4,6,12−15^ Intermolecular reactions involving neutral or protonated aminyl radicals have been documented but only occur as additions to olefinic and acetylenic hydrocarbons and not as intermolecular HAT.^16−21^ When olefins are present in the solvent, the intermolecular addition of protonated or neutral aminyl radicals to olefins competes with the intramolecular HAT step of the HLF propagation cycle, depending on the reaction conditions.

**Figure 1:**
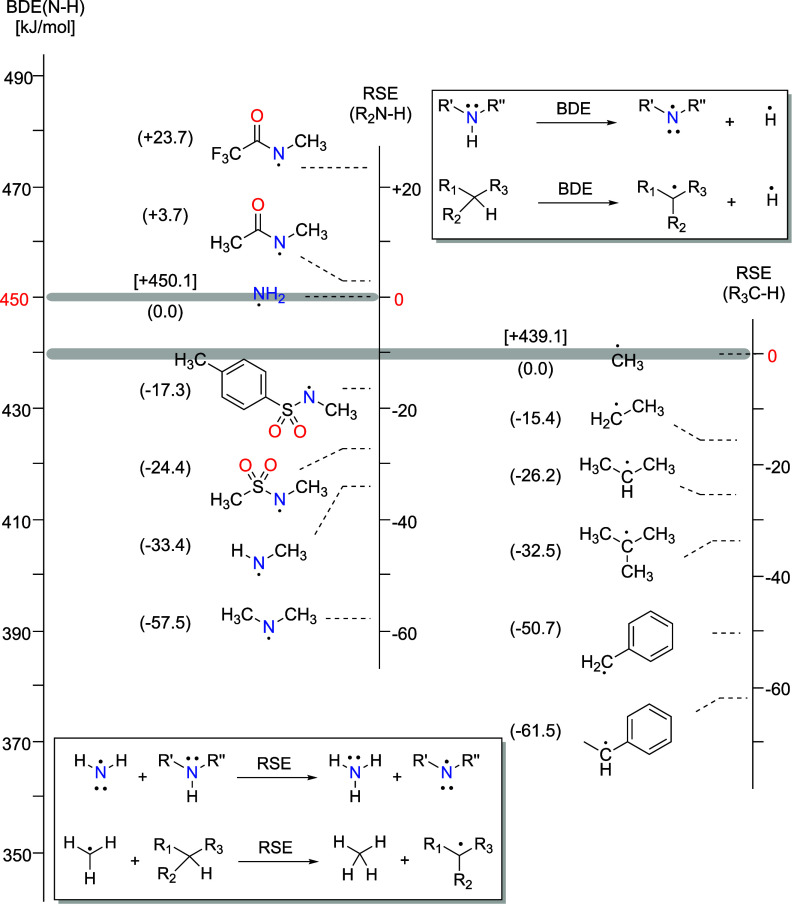
Bond dissociation energies (BDEs) and radical stabilization energies (RSEs) for selected small radical species commonly involved in HLF reactions. Gray bands denote anchor points between RSE and BDE scales. Data calculated at RO-B2PLYP/G3MP2large//B3LYP/6–31G(d) from refs (16, 22), and (25).

To the best of our knowledge, few published studies have investigated the rate-limiting step of the HLF reaction and the inherent causes that guide the regioselectivity. Wolff^4^ has argued that the second step, XAT, has a smaller activation barrier, and from this point on, it was assumed that the HAT step is rate-limiting. In this context, extensive computational studies have been published investigating it,^22−25^ where the thermodynamics of this step is evaluated via the radical stabilization energies (RSEs) for a family of isodesmic reactions and plotted against corresponding activation barriers (Bell–Evans–Polanyi principle). The main conclusion from these studies is that the selectivity of attack by the aminyl radical on a carbon atom depends on the reactivity of the aminyl radical and on the stability of the ensuing carbon radical (see Figure 1). Therefore, aminyl radicals that can abstract hydrogen atoms from carbon generally show a preference for hydrogen in the order of tertiary > secondary > primary. Furthermore, by changing the activating group on the N-centered aminyl radical, the outcome of the reaction can be dramatically influenced. This, however, does not provide an answer to whether the regioselectivity is determined by thermodynamics or kinetics and has not been placed in the context of the propagation cycle.

The vast majority of papers dealing with HLF reaction report on pyrrolidine formation, and there has been just one paper reporting on exclusive piperidine formation.^26^ However, there have been a couple of papers reporting on the functionalization of the C_6_ position with a chlorine atom.^27−29^ To investigate the factors determining regioselectivity and identify the rate-limiting step, we chose a joint experimental and computational approach to study a system that was employed in piperidine synthesis. Our aim is to detect as many as possible radical intermediate species involved in the propagation cycle and analyze major products formed. Quantum chemical calculations have been used extensively to identify these radical intermediates and reaction products. The proposed calculated reaction mechanism should explain the experimental results, observed regioselectivity, and kinetic measurements. Finally, we propose that a combined approach involving both computational techniques and experiments must be employed when addressing fundamental questions, such as regioselectivity, and the rate-limiting step of the reaction sequence must be answered.

## Results and Discussion

**Scheme 2:**
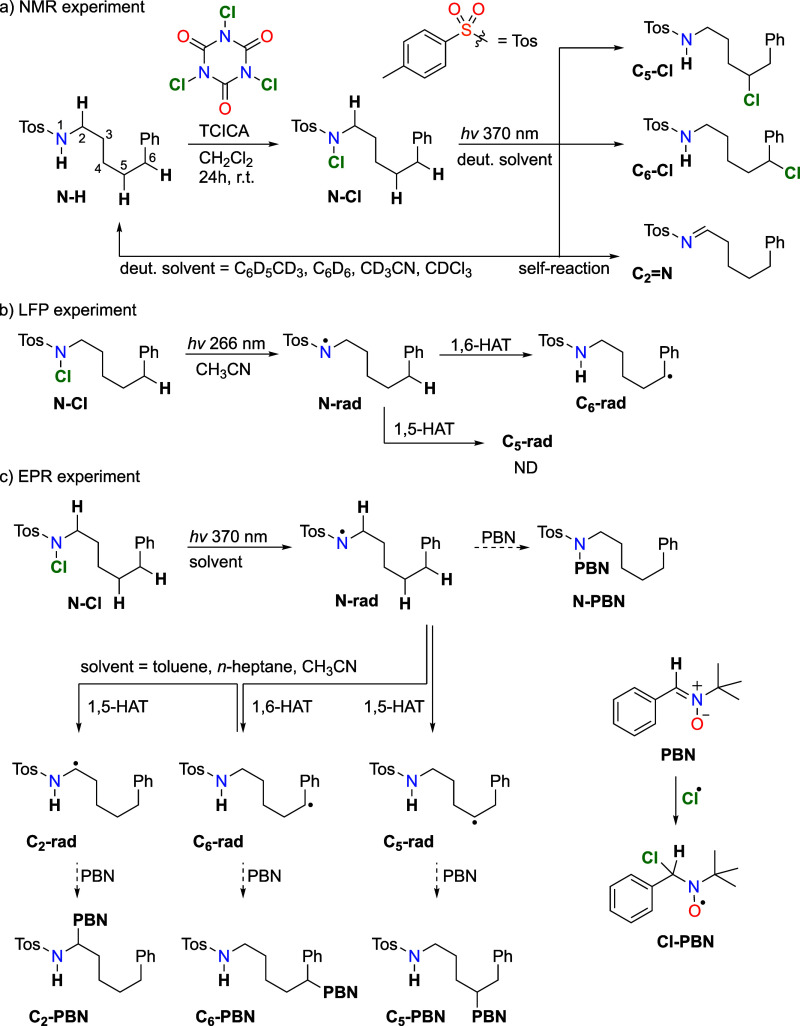
Expected Products and Intermediates of the HLF Reaction Were Measured Using Three Different Techniques (a) Synthesis of **N-Cl** and NMR observed products of the reaction mixture after 370 nm irradiation of **N-Cl**, (b) radical intermediates observed after laser excitation at 266 nm of **N-Cl** in flow cuvettes (3 mL; 3 × 10^–4^ M) at 266 nm in N_2_-purged acetonitrile, and (c) radical intermediates after 370 nm irradiation and PBN spin-trapped products in EPR experiment. Details of the experiments are found in the SI.

To provide experimental evidence for the interplay between two reaction steps in the propagation cycle, we employed NMR, LFP, and EPR techniques (Scheme 2). The overall reaction progress and major product analysis was observed with different NMR techniques, with *off-site* irradiation using a 370 nm Kessil lamp. Direct detection of radical intermediates with measurement of their rearrangement kinetics was performed using LFP via the fourth harmonic of the Nd:YAG laser (266 nm). Additionally, we attempted an *in situ* generation and spin-trapping of N- and C-centered radicals using the phenylbutylnitrone (PBN) spin trap and investigated the resulting adducts with EPR (see Scheme 2c). Finally, we performed extensive DFT calculations and kinetic modeling of the reaction pathways, with the full model described in detail in Section S12 of the Supporting Information.

**Figure 2:**
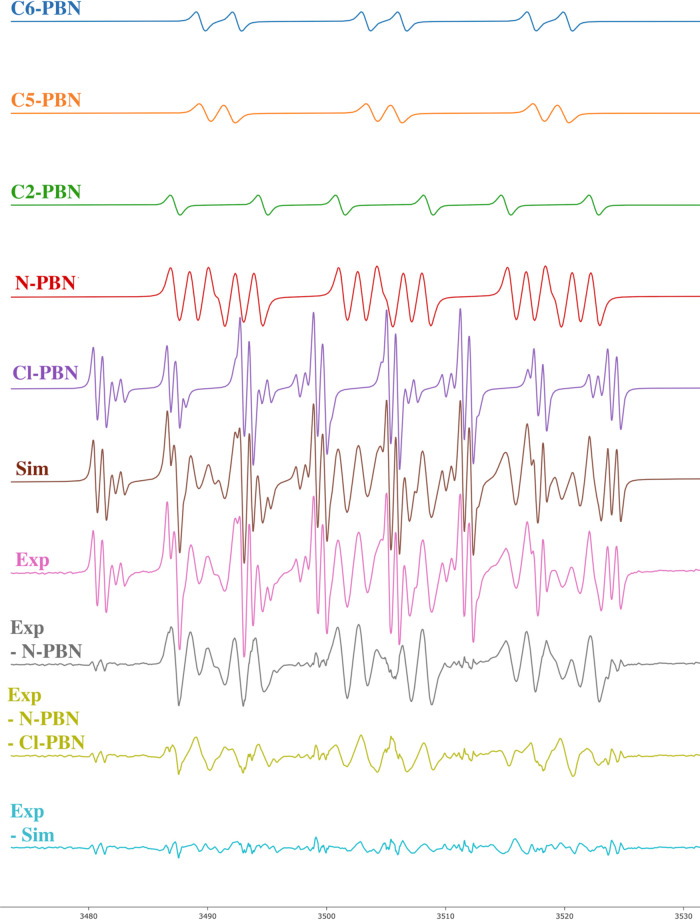
EPR spectra of spin-trapped radical intermediates generated with 370 nm irradiation of **N-Cl**. The simulated spectra of each radical adduct species are denoted in the spectra as **C**_**6**_**-PBN**, **C**_**5**_**-PBN, Cl-PBN**, **C**_**2**_**-PBN**, and **N-PBN**. Total simulated spectrum is labeled as **Sim**, while the experimental spectrum is labeled as **Exp**, with residual signals provided as **Exp–N-PBN**, **Exp–N-PBN–Cl-PBN**, and **Exp–Sim**. Line widths measured with EPR and reaction yields were influenced by the effectiveness of air removal using freeze–pump–thaw cycles with backfill of argon or nitrogen gas. Experimental line widths of less than 0.4 G were deemed satisfactory for the optimal resolution of radical adducts. More information on deconvolution and simulation is deposited in the SI.

Using continuous irradiation with a UV lamp from the bottom of the cavity resonator, the complete reaction sequence was monitored with EPR spectrometry for **N-Cl** (Scheme 2c). The resulting experimental spectrum is shown in Figure 2. The best decomposition of the experimental EPR spectrum for **N-Cl** was found to be the one with one N-centered radical adduct, **N-PBN**; a Cl radical adduct, **Cl-PBN**; and three C-centered radical adducts (assigned as **C**_**6**_**-PBN**, **C**_**5**_**-PBN**, and **C**_**2**_**-PBN**) with different hydrogen hyperfine couplings (*hfc*) (Table 1). This total simulated spectrum, supported by our DFT calculations, aligns well with the experimental data (Figure 2). As a result, we were able to observe a **Cl-PBN** adduct, proving the homolytic cleavage of **N-Cl** bonds generating a chlorine radical that quickly combines with **PBN**. An **N-PBN** adduct was formed from the addition of an N-centered radical to a **PBN** molecule. Calculated EPR parameters for **N-PBN** are in good agreement with the experimental values. Finally, we were able to observe three distinct PBN adducts of C-centered radicals, namely, **C**_**6**_**-PBN**, **C**_**5**_**-PBN**, and **C**_**2**_**-PBN**. Experimental *hfc* values of these three PBN adducts differ enough to distinguish them, although in DFT calculations, **C**_**6**_**-PBN** and **C**_**5**_**-PBN** have similar calculated *g*-factor values, while **C**_**2**_**-PBN** differs from them. Calculated EPR parameters for **C**_**6**_**-PBN** and **C**_**5**_**-PBN** are in satisfactory agreement with the experimental values. The difference in *g*-factors and *hfc* values of the **C**_**2**_**-PBN** radical adduct compared to the rest of C-centered radicals is due to the different connectivity and closer secondary N atom to the radical center.^16^ Again, calculated values for **C**_**2**_**-PBN** have the same trend as the experimental parameters. Additional support to the correct assignment of radicals comes from similar experimental *g*-factors and *hfc* values for **C**_**2**_**-PBN**, **N-PBN**, and **C_5_-PBN** radicals generated from *N*-chloro-*N*-hexyl-4-methylbenzenesulfonamide.^16^ At this point, it is worth noting that the relative weights of the radical adducts are as follows: **N-PBN**, 1; **Cl-PBN**, 0.72; **C**_**6**_**-PBN**, 0.12; **C**_**5**_**-PBN**, 0.17; and **C**_**2**_**-PBN**, 0.11.

**Table 1:** Experimental and Calculated EPR Parameters for the Observed Radicals in the EPR Spectrum^a^

	**N-PBN**		**Cl-PBN**	**C**_**6**_**-PBN**		**C**_**5**_**-PBN**		**C**_**2**_**-PBN**	
	EXP	CALC	EXP	EXP	CALC	EXP	CALC	EXP	CALC
*g*	2.00612	2.0063	2.0077	2.0064	2.00591	2.0064	2.00599	2.0062	2.00611
α_N_	13.82	14.21	12.37	13.96	14.78	13.96	15.32	13.72	14.06
α_H_	2.85	3.87	0.76	3.06	3.67	2.03	2.37	7.38	5.49
α_N’_	1.54	1.57	α_Cl_ 6.23						
ratio	1.00		0.72	0.12		0.17		0.11	

a Calculated at the B3LYP/(C,H,O)EPR-III/(S)def2-QZVP/(N)6-31G(d)//B3LYP/6-31G(d) level of theory. Hyperfine coupling (*hfc*) units are in Gauss.

**Figure 3:**
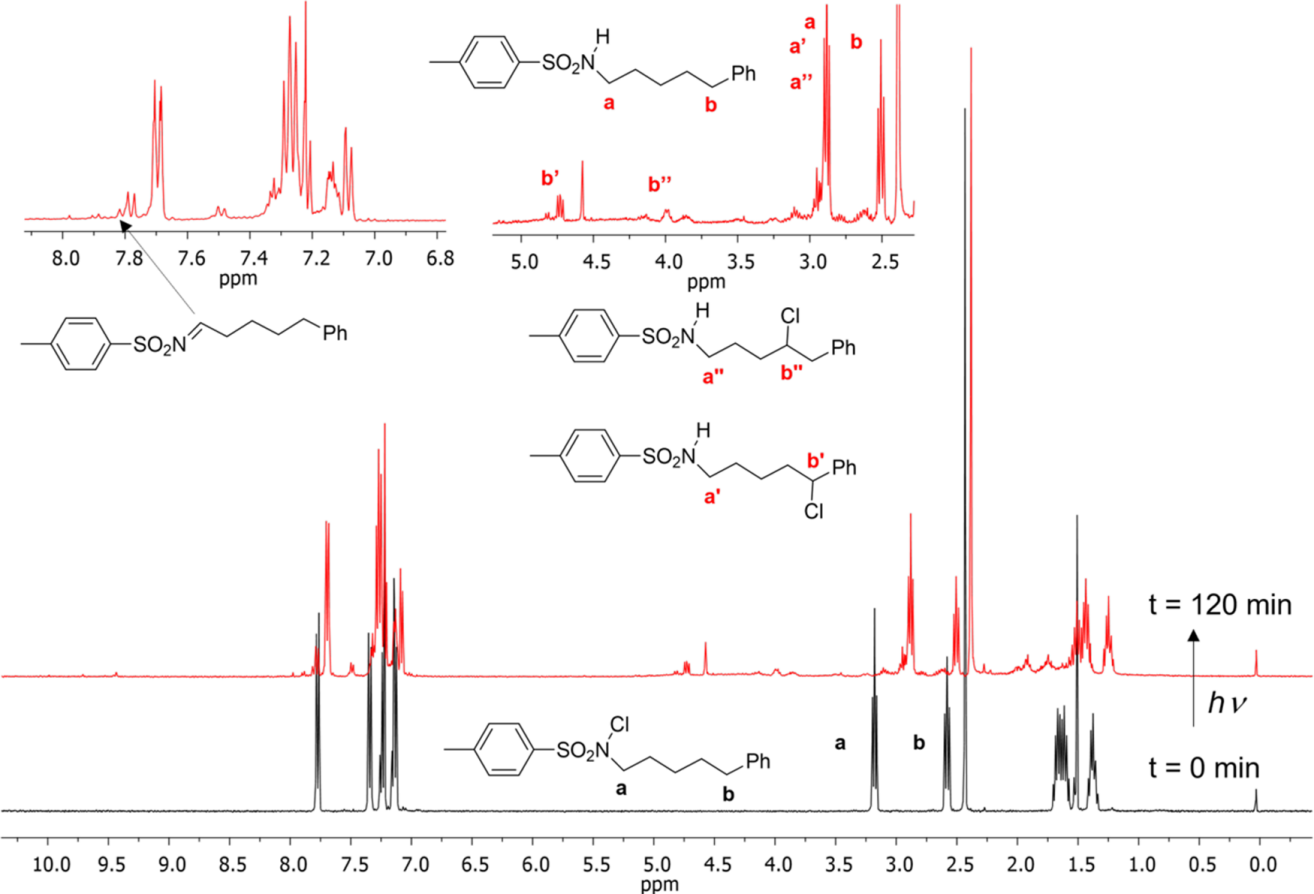
NMR spectra before and after *off-site* irradiation in CDCl_3_. The black and red spectra correspond to the **N-Cl** and reaction mixture of **N-H**, **C**_**6**_**-Cl**, **C**_**5**_**-Cl**, and **C**_**2**_**=N**, respectively. Insets depict selected signal ranges in more detail, with chosen signals assigned. The ^1^H, HSQC, and ^13^C{^1^H} APT spectra for experiments in C_6_D_6_ are deposited in the SI.

Before *off-site* irradiation, ^1^H NMR and ^13^C APT spectra of **N-Cl** (Scheme 2a) were recorded. All signals observed for the starting material are consistent with the expected signals for **N-Cl**. After *off-site* irradiation of the **N-Cl** precursor in toluene, a mixture of products was obtained with total conversion of the starting material, as indicated by the ^1^H NMR spectrum of the reaction mixture. Out of the total **N-Cl** compound in the NMR tube, ∼42% corresponds to the **N-H** product (Figure 3). The mechanism by which this reversal to the amine parent compound occurs has baffled us, although it is a common phenomenon reported in the literature.^18,28−30^ Our first mechanism proposal involves the reaction of chlorine radical or N-centered radical **N-rad** with solvents,^20^ producing solvent radicals and polymerization side reactions resulting in a cloudy reaction mixture.

The analysis of the other products using the ^1^H NMR spectrum combined with the 2D technique, HSQC and ^13^C APT spectra, provides unambiguous evidence that the signals observed at 4.47 and 3.75 ppm correspond to **C**_**6**_**-Cl** and **C**_**5**_**-Cl** products, respectively, with an overall NMR yield of ∼47% (**C**_**6**_**-Cl**/**C**_**5**_**-Cl** = 71:29%) (Figure 3). This was determined from the integral ratios (SI). From additional NMR experiments in other solvents, we found that approximately 50% of **N-H** and 50% of the **C**_**6**_**-Cl** and **C**_**5**_**-Cl** mixture (72:28%) were obtained in deuterated benzene. In deuterated acetonitrile, we obtained 66% of **N-H** and 34% of the **C**_**6**_**-Cl** and **C**_**5**_**-Cl** mixture (57:43%). We can safely conclude that the H atom from the trace water is not involved in the mechanism of converting **N-Cl** back to the parent **N-H** species due to the same pattern of product ratios in a wide selection of solvents. Alas, by examination, we have observed a peak (δ 7.76, t, *J* = 11 Hz, in CDCl_3_) in the aromatic NMR that corresponds to the imine signal and an NMR yield of ∼11%. This indicates the occurrence of a self-reaction facilitated by hydrogen atom transfer (HAT) between two **N-rad** molecules, leading to their termination and the formation of the starting amine (**N-H**) and imine (**C**_**2**_**=N**) products. These disproportionation products are described in the literature,^31,32^ and it is not a coincidence that many chemists deliberately design precursors with the C_2_ position blocked or unavailable.^28−30^ Additionally, the necessity of blocking the C_2_ position in HLF reactions has been studied in detail in our previous work.^15^ Our calculations predict a barrier of Δ*G*^‡^_298_ = +59.4 kJ mol^–1^, with a thermodynamic driving force of Δ*G*_298_ = −134.6 kJ mol^–1^ for this reaction.

LFP measurements were performed on **N-Cl** in acetonitrile to directly detect the transient species generated after laser excitation at 266 nm (4.66 eV) (Scheme 2b). This resulted in the homolytic cleavage of the weakest N–Cl bond (4.03 eV)^33^ and the formation of an aminyl radical and, through subsequent rearrangement reactions, corresponding C-centered radicals. This is confirmed by EPR experiments in toluene, acetonitrile, and *n*-heptane, in which we detected **N-PBN** and **Cl-PBN** after the irradiation of **N-Cl** (Figure 2).

**Figure 4:**
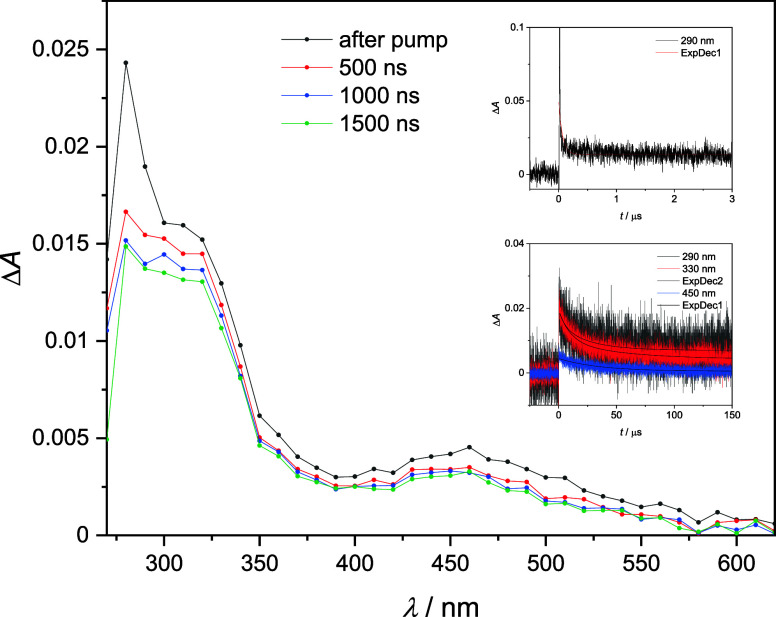
Transient absorption spectra of a N_2_ purged solution of 0.3 mM **N-Cl** in acetonitrile. Flow rate: 2.4 mL/min. *E*_266_ = 22 mJ. *A*_266_ = 0.27. Insets: corresponding time profiles at 290, 330, and 450 nm.

The intramolecular HAT from one of the five carbon atoms of **N-Cl** to the nitrogen atom enables the formation of one or more C-centered radicals. Among these, a benzyl radical, **C**_**6**_**-rad**, is the most likely to be detected by LFP due to the benzene ring acting as a chromophore. The transient absorption spectrum (Figure 4) displays three distinct maxima: one at 280 nm, the second at 310 nm, and the third at 460 nm. The shape of these spectra does not change significantly within the 1500 ns time frame after the laser excitation. However, it is understandable from the spectra that multiple transient species are present, which are deduced from the fact that some maxima disappear faster than others. Furthermore, there are no major changes in the shape of the spectra, which imply that the transient moieties are related and have major structural features in common. Conclusive evidence to support this comes from kinetic data collected at the respective wavelengths. We have a first-order decay with two contributions at 290 and 330 nm. The shorter time scale at 290 nm better reflects the kinetics of the shorter-lived transient species, which lives less than 20 ns and is close to the detection limit, while the longer-lived transient determined from the extended time scale has a lifetime (τ) of 25 μs at 290 nm and 47 μs at 330 nm. Kinetics at 450 nm follows the single exponential decay with only one component. The lifetime of this component is 35 μs. The small differences between the lifetime values may be due to variations in the signal-to-noise ratio (SNR) or due to different quantitative shares of the radicals presented (see the SI). The shorter-lived transient at 290 nm is assigned to an aminyl radical formed by the N–Cl bond cleavage. Amidyl and aminyl radicals have previously been generated by LFP, and their τ values have been reported whether they were directly detected with LFP or indirectly from the detected C-centered radical formed by cyclization/intramolecular HAT. Reported τ values of amidyl and aminyl N-centered radicals range from 5 to 454 ns.^34−37^ This is in agreement with our experimental results.

To check if the aminyl N-centered radical, **N-rad**, absorbs at 290 nm, we have done extensive TD-DFT calculations that indicate a strong absorption peak at 280 nm with an oscillator strength (*f*) of 0.0238 (SI). We conclude that the longer-lived transient is one of the generated C-centered radicals, most likely a benzyl-type **C**_**6**_**-rad**. This is in good agreement with the literature, which reports a lifetime of 40 μs for benzyl radicals, generated from benzyl chloride in hexane, with a maximum at about 315 nm.^38^ Additionally, our measurements of the benzyl radical generated from BnCl in acetonitrile show a maximum at 310 nm and a lifetime of 30 μs (see Section S8 in the SI).S8 in the SI).

**Figure 5:**
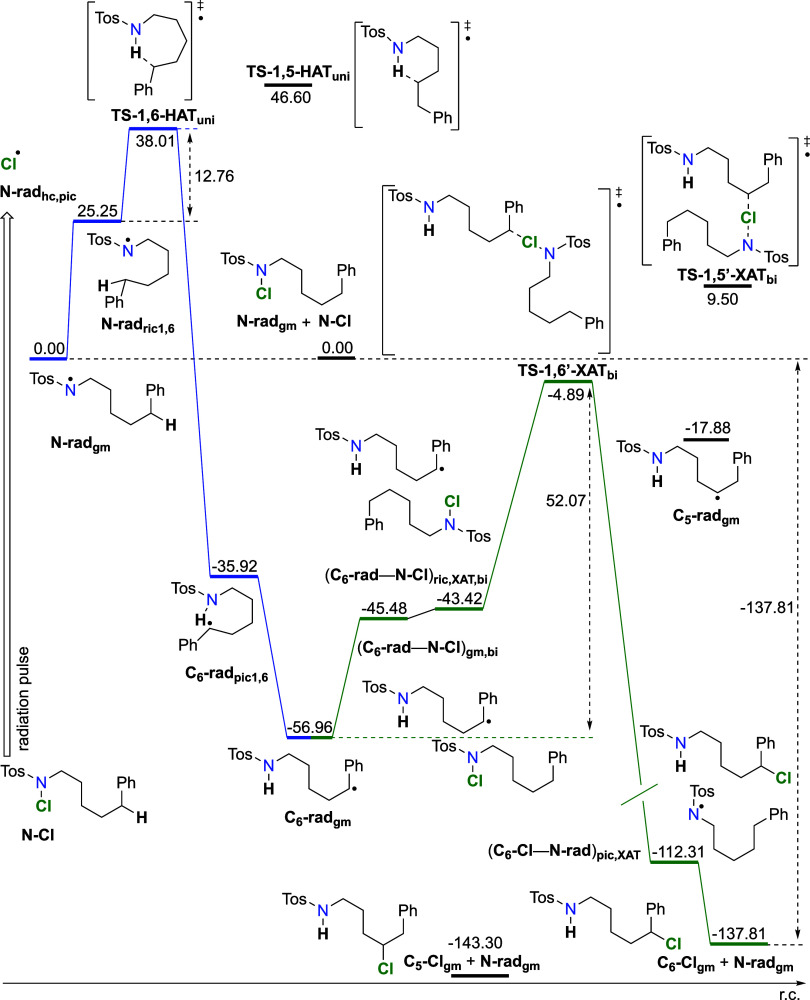
Energy diagram of intra- and intermolecular radical rearrangements for the propagation cycle of HLF for the 1,6-pathway. From the starting N-centered radical **N-rad**, it rearranges via **TS-1,6-HAT** to the **C**_**6**_**-rad**. The next step is the bimolecular reaction from **C**_**6**_**-rad** and **N-Cl**, via **TS-1,6’-XAT**, to **C**_**6**_**-Cl** and **N-rad** formation. Calculated at the RO-B2PLYP/G3MP2Large(SMD,CH_3_CN)//B3LYP/6-31G(d) level of theory. Included in the diagram are chosen points of the 1,5-pathway. The global minimum structure **N-rad**_**gm**_ was taken as the starting point in the unimolecular process, while the global minima of separated reactants, namely, **N-rad**_**gm**_ and **N–Cl**_**gm**_, were taken as the starting point in the bimolecular reaction. Units are given in kJ mol^–1^.

As seen in Figure 5, DFT calculations of the 1,6-HAT step involves rearrangement from the global minimum on the reactant side (**N-rad**_**gm**_) to a prereactive conformer (**N-rad**_**ric1,6**_) that is Δ*G*_298_ = +25.3 kJ mol^–1^ less stable. From there, transition state **TS-1,6-HAT_uni_** is reached with an overall barrier of Δ*G*^‡^_298,gm_(**1,6-HAT**_**uni**_) = +38.0 kJ mol^–1^. When the barrier is defined as in eq 2, Δ*G*^‡^_298,ric_(**1,6-HAT**_**uni**_) equals 12.8 kJ mol^–1^. In the first case (Δ*G*^‡^_298,gm_), the calculated *t*_1/2_ of the aminyl radical is 514 ns, while for the second case (Δ*G*^‡^_298,ric_), the calculated *t*_1/2_ of 0.02 ns is in far better agreement with LFP experimental results. This led us to the conclusion that upon homolytic cleavage (hc) of the N–Cl bond, the N-centered aminyl radical **N-rad**_**hc,pic**_ exists as a high energy conformer on the potential energy surface and is almost equal in energy to the prereactive conformer **N-rad**_**ric1,6**_ of the IRC path. This is a case where Boltzmann distribution does not apply, and the low energy state is unavailable due to kinetic reasons, with **N-rad**_**hc**_**_,_**_**pic**_ quickly rearranging to **N-rad**_**ric1,6**_. On the product side, the first local minimum encountered is **C_6_-rad**_**pic1,6**_ at Δ*G*_298_ = −35.9 kJ mol^–1^, which then rearranges to the most stable conformer, a global minima (gm) **C_6_-rad**_**gm,uni**_ with an overall Gibbs free energy of reaction of Δ*G*_298_ = −57.0 kJ mol^–1^. At this point, **C_6_-rad** and **N–Cl** species are separated in the solvent. When they meet in the solvent cage due to diffusion, a complex (**C6-rad–N-Cl**)**_gm,bi_** is formed at Δ*G*_298_ = −45.5 kJ mol^–1^, after which a prereactive conformer for the **XAT** is formed, (**C6-rad**–**N-Cl**)**_ric,XAT,bi_**, at Δ*G*_298_ = −43.4 kJ mol^–1^. From there, the transition state **TS-1,6’-XAT_bi_** is reached with an overall barrier of Δ*G*^‡^_298,gm_**(1,6’-XAT**_**bi**_) = +52.1 kJ mol^–1^. If the barrier is calculated according to eq 2, the Δ*G*^‡^_298,ric_**(1,6’-XAT**_**bi**_) value is at +38.5 kJ mol^–1^. For the first case, the *t*_1/2_ of the **C**_**6**_**-rad** species is 149 μs, and for the second case, the *t*_1/2_ is 633 ns. The first case describes the *t*_1/2_ of radical **C**_**6**_**-rad** closer to the experimental results.

Hence, due to the better fit with the experiment, we use Δ*G*^‡^_298,ric_(**1,6-HAT**_**uni**_) calculated from the prereactive minimum for the HAT reaction as a first step in the propagation cycle, while for the second step, which involves a bimolecular XAT reaction, we use Δ*G*^‡^_298,gm_(**1,6’-XAT**_**bi**_) again due to the better fit to the experimental results.

We also performed TD-DFT calculations that show that **C**_**6**_**-rad** has an absorption peak at 295.36 nm with an oscillator strength of *f* = 0.0362 and that the N-centered aminyl radical, **N-rad**, has a strong absorption peak at 290 nm with an oscillator strength (*f*) of 0.0238 (SI). Our experimental lifetimes of the radicals in the LFP agree with the lifetimes reported in the literature sources and with the calculated data. This leads us to the conclusion that we have observed an N-centered aminyl radical and benzylic-type **C**_**6**_**-rad** in the LFP experiments.

**Figure 6:**
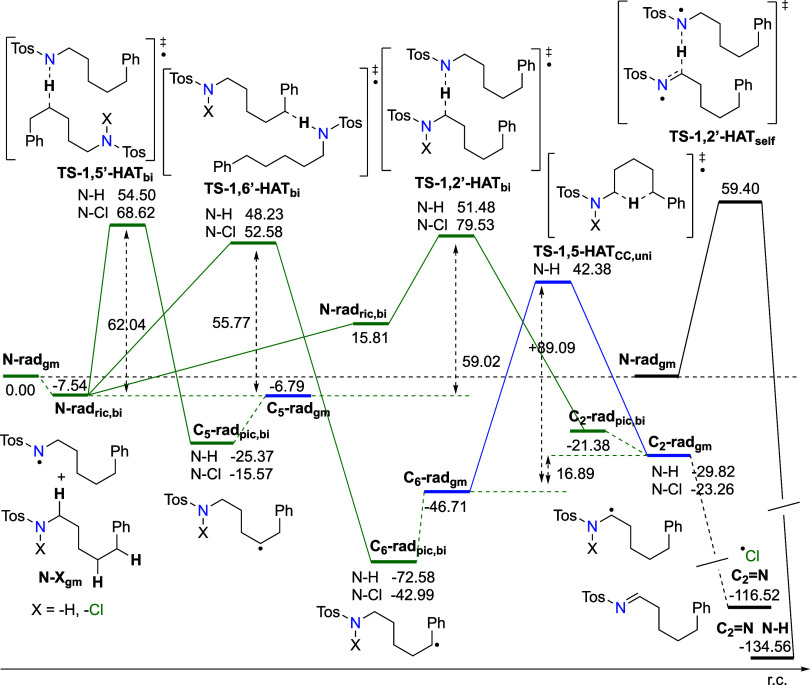
Energy diagram of intra- and intermolecular radical rearrangements to relevant C-centered radicals derived from **N-H** and **N-Cl**, and recombination through self-reaction to **C_2_=N** and **N-H** species. Calculated at the RO-B2PLYP/G3MP2Large(SMD,CH_3_CN)//B3LYP/6-31G(d) level of theory. Global minima of separated reactants, namely, **N-rad**_**gm**_, **N–Cl**_**gm**_, and **N–H**_**gm**_, were taken as the starting point in the bimolecular reaction, while the global minimum structure on the reactant side, **N-rad**_**gm**_, was taken as the starting point in the unimolecular process, and the dimer of **N-rad**_**gm**_ was taken as the starting point for the self-reaction. Units are in kJ mol^–1^.

As seen in Figure 2, there is a **C**_**2**_**-rad** in the EPR spectra that can be formed via HAT from either **C**_**6**_**-rad** (**1,5-HAT**_**CC,uni**_) or **N-rad** (**1,2’-HAT**_**bi**_). A similar radical has been observed in the EPR spectra in the *N*-hexyl-4-methylbenzenesulfonamide system.^16^ When compared to the other bimolecular HAT reactions (Figure 6), the barrier for the formation via **1,2’-HAT**_**bi**_ is between the barriers for the C-centered alkylic-type radical and benzyl-type radical. Thermodynamically, **C**_**2**_**-rad** is the second most stable C-centered radical, with stability closer to the **C**_**6**_**-rad** than the **C**_**5**_**-rad**. Calculated reaction energies for all these processes are slightly higher than the barriers calculated in the 1,6-pathway. This is especially true for the unimolecular HAT converting **C**_**6**_**-rad** to **C**_**2**_**-rad**, with the highest calculated barrier of Δ*G*^‡^_298_ = +89.1 kJ mol^–1^. However, the **C**_**2**_**=N** imine species, the product observed in the NMR experiment, is generated primarily by the self-reaction of two **N-rad** (see above) and then from **C**_**2**_**-rad**. Another pathway for imine production involves 1,2’-HAT transfer between **N-Cl** and **N-rad**, but this reaction is kinetically less favored with a higher calculated barrier (Δ*G*^‡^_298_ = +79.53 kJ/mol).

At this point, it is worth noting that the first-order decay kinetics shown in the insets of Figure 4 does not reach zero but forms an onset close to zero. This implies that there is a steady state for a C-centered radical intermediate, which is presumably an intermediate species in the propagation cycle. Furthermore, the decay kinetics for **C**_**6**_**-rad** can only be accurately described if the rate constant for the preceding elementary reaction is significantly larger or larger than the rate constant of the subsequent elementary reaction, assuming that these rate constants are the primary contributors to the observed experimental rate constants. This is corroborated by our DFT calculations as the calculated rate constant (eq 4) for 1,6-HAT is 2.4 × 10^10^ s^–1^, while for the bimolecular **XAT**, the rate constant is 7 orders of magnitude lower, namely, 4.63 × 10^3^ s^–1^. Additionally, the lifetime of the N-centered aminyl radical **N-rad** is much shorter when compared to the lifetime of the C-centered radical **C**_**6**_**-rad**. This is viable only when the second step is slower than the first. Thus, we conclude that the slow step of the propagation cycle is intermolecular **XAT**. On the product side, the first local minimum encountered is (**C_6_-Cl–N-rad**)**_pic,XAT_** at Δ*G*_298_ = −112.3 kJ mol^–1^, which then rearranges to the most stable conformer **C_6_-Cl**_**gm**_ with an overall Gibbs free energy of reaction of Δ*G*_298,rx,gm_ = −137.8 kJ mol^–1^.

**Figure 7:**
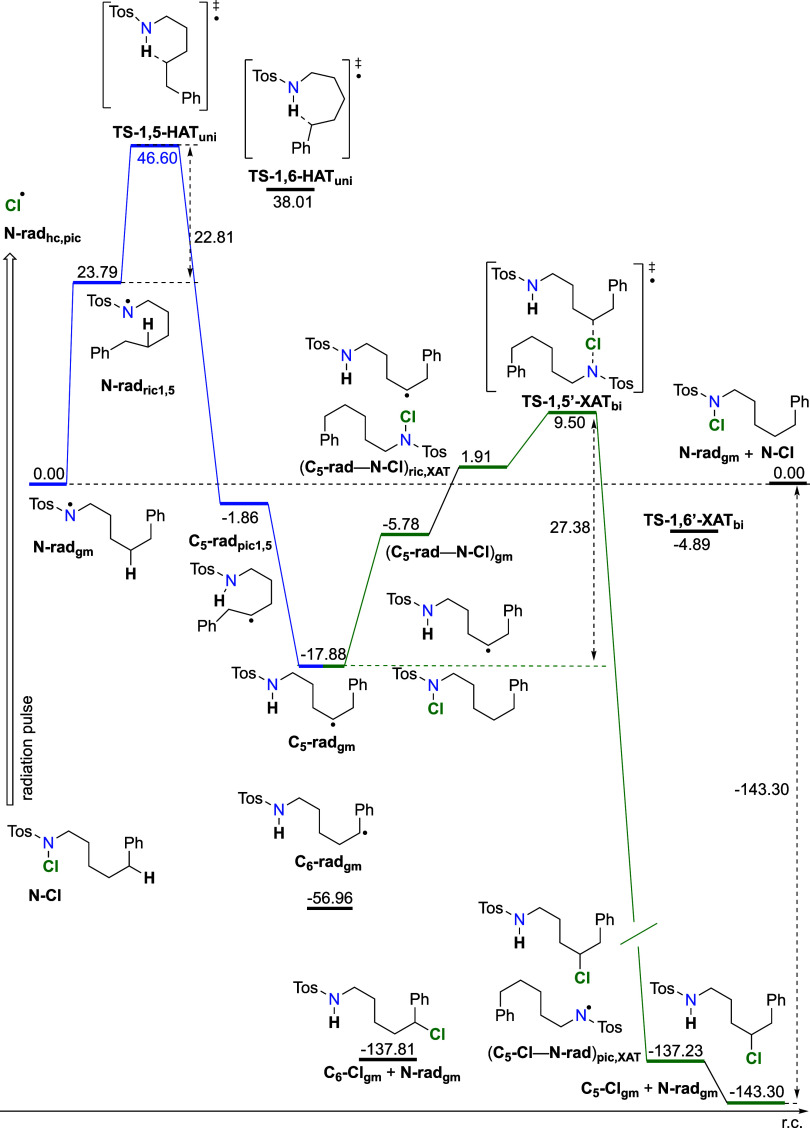
Energy diagram of intra- and intermolecular radical rearrangements for the propagation cycle of HLF for the 1,5-pathway. From the starting N-centered radical **N-rad**, it rearranges via **TS-1,5-HAT** to the **C**_**5**_**-rad**. The next step is the bimolecular reaction from **C**_**5**_**-rad** and **N-Cl**, via **TS-1,5′-XAT**, to **C**_**5**_**-Cl** and **N-rad** formation. Calculated at RO-B2PLYP/G3MP2Large(SMD,CH_3_CN)//B3LYP/6-31G(d) level of theory. Included in the diagram are chosen points of the 1,6-pathway. The global minimum structure on the reactant side, **N-rad**_**gm**_, was taken as the starting point in unimolecular process, while the global minima of separated reactants, namely, **N-rad**_**gm**_ and **N-Cl**_**gm**_, were taken as the starting point in bimolecular reaction. Units are in kJ mol^–1^.

For the 1,5-pathway (Figure 7), the HAT step involves rearrangement from the global minimum on the reactant side (**N-rad**_**gm**_) to a prereactive conformer (**N-rad**_**ric1,5**_) that is Δ*G*_298_ = +23.8 kJ mol^–1^ less stable. From there, transition state **TS-1,5-HAT_uni_** is reached with an overall barrier of Δ*G*^‡^_298_ = +46.6 kJ mol^–1^. When the barrier is defined from **N-rad**_**ric1,5**_, it amounts to only 22.8 kJ mol^–1^, which includes a fast rearrangement step between high-energy conformer **N-rad**_**hc,pic**_ and **N-rad**_**ric1,5**_. On the product side, the first local minimum encountered is **C_5_-rad**_**pic**_ at Δ*G*_298_ = −1.9 kJ mol^–1^, which then rearranges to the most stable conformer **C_5_-rad**_**gm,uni**_ with an overall Gibbs free energy of reaction of Δ*G*_298_ = −17.9 kJ mol^–1^. Species needed for the bimolecular reaction, namely, the **C_5_-rad** radical and **N-Cl**, are still separated in the solvent. When they meet in the solvent cage due to diffusion, the complex (**C_5_-rad**–**N-Cl**)**_gm,bi_** is formed at Δ*G*_298_ = 1.9 kJ mol^–1^, which leads to the prereactive conformer of the **XAT** reaction (**C**_**5**_**-rad–N-Cl**)**_ric,XAT_** at Δ*G*_298_ = 5.8 kJ mol^–1^. From there, the transition state is reached with an overall barrier of Δ*G*^‡^_298,gm_(**1,5′-XAT**_**bi**_)= +27.4 kJ mol^–1^, while it amounts to Δ*G*^‡^_298,ric_(**1,5′-XAT**_**bi**_) = 7.6 kJ mol^–1^ when the barrier is defined as in eq 2. On the product side, the first local minimum encountered is (**C**_**5**_**-Cl**–**N-rad**)**_pic,XAT_** at Δ*G*_298_ = −137.2 kJ mol^–1^, which then rearranges to the most stable conformer **C_6_-Cl**_**gm**_ with an overall Gibbs free energy of reaction of Δ*G*_298,rx,gm_ = −143.3 kJ mol^–1^. To check if the **C**_**5**_**-rad** radical moiety was detected in our LFP measurements at 290 nm, we have done TD-DFT calculations that show that it has a strong absorption peak at 238.04 nm with an oscillator strength of *f* = 0.0290 (SI). This excludes the possibility of it being detected in this experimental setup.

The complete energy schemes (Figures 5 and 7) for the 1,6- and 1,5-pathways allow us to compare similarities between the two processes. As observed, we notice that there is an early transition state for the second step and that the second step is irreversible, which in turn makes the whole cycle irreversible, even though the N-centered aminyl radical is regenerated. Furthermore, there is no thermodynamic preference for the formation of **C6-Cl** over **C5-Cl**, with both products in the same energy range (∼−140 kJ mol^–1^), as compared to the starting **N-Cl** compound. The definition of the barrier from the global minimum, as in eq 3, describes experimental results much better for the second step, namely, the XAT reaction, while the definition of the barrier from the prereactive minimum, as in eq 2, fits much better with the experimental results for the first step, namely, the HAT step. Moreover, NMR analysis of the product mixture in toluene shows that we have 71.5% **C**_**6**_**-Cl** and 28.5% **C**_**5**_**-Cl**. This is an indication that the kinetics of the HAT step determines regioselectivity, as the barrier for 1,6-HAT is 13.8 kJ mol^–1^ and that for 1,5-HAT is 23.8 kJ mol^–1^. When Bodenstein approximation of quasi-stationary behavior^39,40^ and the long chain approximation are applied to radical chain reactions, the reaction rates of the individual steps in the cycle are equal. However, different rate constants strongly imply the quite large steady-state concentration of the C-centered radicals, **C**_**5**_**-rad** and **C**_**6**_**-rad**, each in its own cycle, compared to **N-rad** created in the propagation step. When these approximations are applied in our kinetic model, we obtained that the HLF reaction follows pseudo-first-order kinetics with respect to the second step. Thus, we propose that the rate of the propagation cycle of the HLF reaction is controlled by the XAT step, which is additionally supported by both the LFP experiments and DFT calculations.

There is another interpretation of the reaction sequence in the literature, which was proposed by Muñiz for the selective synthesis of piperidines.^26^ Instead of uni(intra)molecular HAT, they considered a bi(inter)molecular HAT, where the N-centered succinimide radical extracts selectively only the C_6_-H, providing the C-centered benzylic radical. Since there is no outside chlorinating agent in our reaction (halogenation was performed in the previous reaction step and quantitatively removed), the only possible **1,6’-HAT**_**bi**_ is the reaction of **N-rad** with **N-Cl**, where the hydrogen extraction comes from the N-Cl species. The calculated barrier is Δ*G*^‡^_298,ric_(**1,6’-HAT**_**bi**_) = +52.6 kJ mol^–1^, which corresponds to a *t*_1/2_ of 8.73 ms for the **N-Cl–C**_**6**_**-rad** species. Similar results, Δ*G*^‡^_298,ric_(**1,5′-HAT**_**bi**_) = +68.62 kJ mol^–1^ and *t*_1/2_ of 98.28 ms, are obtained for the 1,5-pathway (see Figure 6). This is in stark contrast to the experimental values obtained from the LFP experiments, where degradation is much quicker. Products of these reactions, **N-Cl–C**_**6**_**-rad** and **N-Cl–C**_**5**_**-rad**, are lower in energy, with a thermodynamic driving force of – 43.0 and – 15.6 kJ mol^–1^, respectively. As discussed above, **C**_**2**_**-rad** is also a good candidate for the HAT reaction.^16^ Yet this radical or downstream products (imine and **C**_**2**_**-Cl**) were not observed in the Muñiz synthesis,^26^ which is another reason why this reaction should be re-examined. After the C-centered radical formation, a uni(intra)molecular XAT process can be envisioned as the final step in the reaction sequence. It should be noted that Muñiz and co-workers make this a bimolecular XAT process with their halogen source (*N*-bromo-succinimide complex with I_2_). For the unimolecular TS-1,6-XAT, from the **N-Cl–C**_**6**_**-rad**_**ric**_ structure, the kinetic barrier is Δ*G*^‡^_298,ric_(**1,6-XAT**_**uni**_) = +101.9 kJ mol^–1^ due to a very extended structure. The thermodynamics of this reaction is exergonic, with postreactive intermediate complex **N-rad**–**C**_**6**_**-Cl**_**pic**_ being −70.5 kJ mol^–1^. For the similar unimolecular TS-1,5-XAT, the reaction barrier is lower (Δ*G*^‡^_298,ric_(**1,5-XAT**_**uni**_) = +75.1 kJ mol^–1^), with thermodynamics of this step comparable to the 1,6-XAT. It should be noted that the starting points for both **XAT**_**uni**_ processes, namely, **N-Cl–C**_**6**_**-rad**_**ric**_ and **N-Cl–C**_**5**_**-rad**_**ric**_, have a 50.4 kJ mol^–1^ difference in energy, with the benzyl type of C-radical more stable than the alkyl type of C-radical. In conclusion, pathways with bimolecular HAT and unimolecular XAT are unfavorable and thus should be excluded as possible mechanisms of the propagation cycle.

To support our experimental and computational results, we have done kinetic modeling of the HLF reactions in the case when only one major product is formed in a yield greater than 99%. In our kinetic modeling, we applied a steady-state approximation for the concentration of the N-centered radical **N-rad**, which we consider reasonable since the **N-rad** is continuously consumed and regenerated throughout the reaction cycle (see Section S12 in the SI)Section S12 in the SI). This approximation also indirectly implies steady states for the **C_5_-rad** and **C_6_-rad** intermediates, as their concentrations are tied to the steady state behavior of **N-rad** through the dominant **C_6_-Cl** pathway. Additionally, the reverse reactions in the second step of each pathway (reactions from chlorinated products **C_5_-Cl**, **C_6_-Cl**, and **C_2_–Cl** back to their respective radicals and **N-Cl**) were neglected. This is supported by Gibbs energy profiles (Figures 5–7), which indicate significantly higher energy barriers for these reverse reactions. As a result, they are unlikely to contribute significantly to the overall kinetics. Using the approximations, we derived a simplified equation that describes the formation of all (final) chlorinated products, as presented in eq 1.
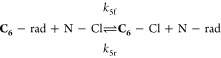

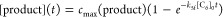
1Here, *c*_max_ represents the maximum concentration of the product, while *k*_5f_ and *k*_5r_ denote forward and reverse rate constants, respectively. The full derivation is presented in the SI. The exponent −*k*_5f_[C_6_]_0_ applies to all products as **N-Cl** depletion is predominantly controlled by the **C_6_-Cl** pathway. As this pathway dictates the overall reaction kinetics, the time-dependent behavior of all products follows the same exponential decay. This pseudo-first-order approximation is in good accordance with the experimental data from the LFP measurements. In addition, we also estimated the reaction half-lives and product ratios, which support the model’s consistency and generally align with the experimental data ( see Section S12 in the SI).Section S12 in SI).

**Scheme 3:**
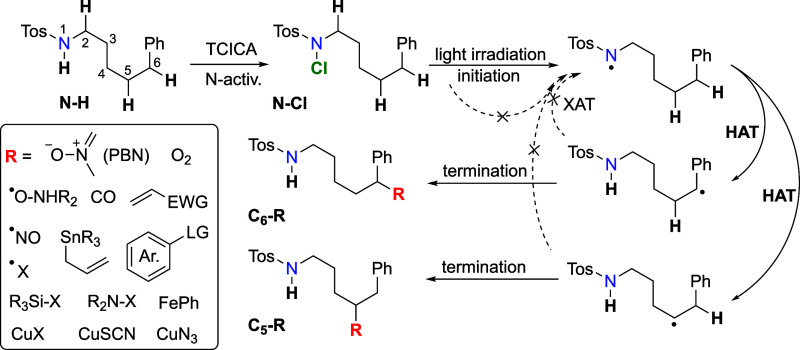
Propagation vs Termination Step in the HLF Reactions Different selection of termination traps may compete with XAT and the closing of the propagation site.

Understanding that XAT is the bottleneck step in the HLF reaction is crucial for controlling the reaction kinetics, predicting the duration of each step and the overall process, and designing specific termination steps.^69^ Formed carbon-centered radicals can react with common halogenating agents in a one-pot reaction setup, such as trichloroisocyanuric acid (TCICA), *N*-chlorosuccinimide (NCS), *N*-bromosuccinimide (NBS), *N*-iodosuccinimide (NIS), or even iodine/sodium iodide (I_2_/NaI), with lower or negligible activation barriers, leading to functionalized *sp*^3^-hybridized carbon centers (see Scheme 3). This pathway bypasses the propagation step, where nitrogen-centered radicals are converted to carbon-centered radicals that promptly terminate with the halogen source. New nitrogen-centered radicals must then be generated via irradiation, which can occur under one-pot reaction conditions, where N-halogenation happens simultaneously with the HLF reaction.Scheme 3

Moreover, this mechanism opens possibilities for remote δ- and *ε*-site functionalization if suitable targets for radical addition are present. For such transformations to occur, the termination reaction must be both faster than the XAT step and thermodynamically more favorable than the halogenated products. An example is the reaction of carbon-centered radicals with a PBN spin trap, yielding an extremely stable NO-type radical in our EPR experiments. Other potential reactions can involve weakly bonded main group molecules (N–X, Si–X, Sn–H, and Sn–allyl), metal salts (CuX, CuSCN, and CuN_3_), and π-systems (alkenes and arenes). We are currently exploring these possibilities in our laboratory and encourage other researchers to investigate these avenues, as they may lead to novel strategies in radical chemistry and synthetic methodologies.

## Conclusions

Using NMR spectroscopy, laser flash photolysis LFP, and EPR spectroscopy in combination with DFT calculations and kinetic modeling, we monitored the reaction profile and identified significant intermediate radicals and products in the Hofmann–Löffler–Freytag reaction (HLF). Our results indicate that there is no thermodynamic preference for the formation of **C**_**6**_**-Cl** over **C**_**5**_**-Cl**, and the difference in ratios (72 vs 28%, respectively) is due to the **C**_**6**_**-Cl** being a kinetic product. Thus, the kinetics of the HAT step in the propagation cycle guides the regioselectivity of the HLF reaction, and by calculating the barrier for the HAT step, we can predict the major product formed. Moreover, we deduce that the slow step of the HLF reaction is the bimolecular XAT step. Additionally, we propose that adding halogen sources, typically used as chlorinating agents, or other traps may interfere with the XAT step of the propagation cycle, promoting the earlier termination of a reaction cycle. We recommend that future research on radical mechanisms be conducted using a combination of experimental techniques paired with rigorous quantum-chemical calculations and kinetic modeling for a comprehensive overview of the reaction.

## Materials and Methods

The purchased compounds were sourced from Sigma-Aldrich (St. Louis, MO, USA) (trichloroisocyanuric acid (TCICA), Celite S, hydrochloric acid (37%), acetone, silicone oil, petroleum ether, and cyclohexane), Fisher Scientific (Waltham, MA, USA) (toluene (anhydrous), acetonitrile (anhydrous), 1,4-dioxane (anhydrous), tetrahydrofuran (anhydrous), *N*,*N*-dimethylformamide (anhydrous), *N*,*N*-dimethylacetamide, 1,2-dichloroethane (anhydrous), dichloromethane (anhydrous), dichloromethane (CH_2_Cl_2_)), and Kemika (Zagreb, Croatia) (sodium hydroxide). All reagents and chemicals were obtained commercially and used without further purification unless otherwise noted. The starting material, 4-methyl-*N*-(5-phenylpentyl)benzenesulfonamide, **N-H**, was provided by the research group of Prof. Hendrik Zipse from Ludwig-Maximilian University, Munich, Germany, and ^1^H and ^13^C{^1^H} NMR spectra values of the compound (see the SI) correspond to the previously reported values.^26^

Chromatographic purification of the products was carried out using column chromatography filled with silica gel (Macherey-Nagel) 0.063–0.2 mm, and appropriate solvent mixtures of petroleum ether/ethyl acetate were used as eluents. Thin-layer chromatography (TLC) was performed on precoated ALUGRAM SIL G/UV254 0.20 mm silica gel 60 plates with a fluorescent indicator UV254 (Macherey-Nagel) in the appropriate solvent system. TLC spots were observed via the illumination with UV light at a wavelength of 254 nm after the immersion of the plate in an aqueous solution of KMnO_4_ (3 g KMnO_4_, 20 g K_2_CO_3_, 5 mL aq. NaOH 5%, and 300 mL water) followed by heating. If TLC spots were not visible after illumination with UV light, they were detected using an iodine chamber.

NMR spectra of the reaction mixture were obtained on a Varian Inova 400 NMR spectrometer operating at 399.90 MHz for ^1^H NMR and 100.6 MHz for ^13^C{^1^H} NMR and are reported as chemical shifts (δ) in ppm. The spectra were imported and processed in the MestreNova 11.0.4 program.^41^

EPR spectroscopy was performed by using a Bruker ELEXSYS E500 EPR spectrometer with an ER4122SHQE cavity resonator. As this cavity resonator does not have an optical window for illumination, the light source was mounted underneath the cavity with light coming through the bottom of the EPR 4 mm inner-diameter tube. EPR deconvolution and simulation were done using an EasySpin module with the MATLAB program package.^42^ EPR visualization and spectroscopy were done using the VisualEPR Web page.^43^ For experiments, 35 mg of **N-Cl** was dissolved in the 0.3 mL of solvent (∼0.04 M), degassed, and then mixed with degassed 10 mg of **PBN** dissolved in 0.3 mL of the same solvent (∼0.015 M).

Transient absorption spectroscopy (TAS) measurements were performed by using a nanosecond laser flash photolysis setup. The setup consisted of a Nd:YAG laser (Quantel, Q-smart 450) and an LP980 transient absorption spectrometer (Edinburgh Instruments). The ground state absorption of the samples was adjusted to 0.3 at the 266 nm laser excitation wavelength (5 ns pulse duration and 10 Hz). The laser pulse energy at 266 nm was in the range of 10–23 mJ (30–70 mJ cm^–2^). Kinetic measurements were performed in 1 cm quartz cells sealed with rubber septa. The transient absorption spectra were measured in a flow cell with a flow rate set to 2.4 mL/min to ensure that no light was absorbed by the photoproducts. All solutions were prepared immediately before the experiments. Solutions were purged with high-purity N_2_ for 20 min prior to the kinetics measurements and for 1 h before spectra measurements. All measurements were performed at 25 °C. UV–vis spectra of the sample solutions were recorded by using a Varian Cary 4000 spectrophotometer (Figures S15 and S16).

The conformational space for all the local minima and saddle points of the first order on the energy diagram was investigated using the Conformer–Rotamer Ensemble Sampling Tool (CREST)^44^ coupled with the xtb-GFN2 program package and meta-dynamics simulation using xtb-GFN1^45^ and xtb-GFN2.^46^ The obtained structures were reoptimized using the B3LYP/6-31G(d) level of theory.^47−49^ For each structure with a stable wave function, a frequency calculation was performed to identify the minima and transition-state structures. The lowest lying conformers, e.g., with the lowest energy value, for each species were labeled global minima (gm) on the potential energy surface (PES). Transition state structures were differentiated from the minima by having exactly one imaginary frequency. From all transition state conformers, an intrinsic reaction coordinate (IRC) search was performed to characterize the corresponding reaction/product channel, and the last point in the forward and reverse direction was then optimized to the nearest local minimum, i.e., reactive complex. On the reactant side, the obtained structure was termed prereactive intermediate complex (ric), while on the product side, the optimized structure was named postreactive intermediate complex (pic). Single point energies were obtained with the universal continuum solvation model SMD,^50^ with acetonitrile as a solvent and RO-B2PLYP^52,53^ with a G3MP2 large basis set^54^ on geometries obtained at the B3LYP/6-31G(d) level of theory, with additional D3 dispersion correction.^55^ The thermal corrections to the free energy were derived from the frequency calculations under conditions of 298.15 K and 1 atm. The activation free energies (Δ*G*^‡^_298_) of each elementary reaction are defined in two distinct ways through the following equations:
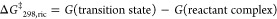
2
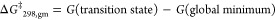
3

According to transition state theory (TST),^56,57^ approximate reaction rate constants for elementary reactions, in which the reactants directly generate products, were estimated based on the Eyring–Polanyi equation as eq. 4:
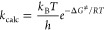
4where *k*_B_ is the Boltzmann constant, *T* is the temperature, *h* is Planck’s constant, *R* is the molar gas constant, and Δ*G*^‡^ is the activation free energy.

Calculations of EPR parameters were done using the B3LYP functional and a mixed basis set: EPR-III was used for C, H, and O atoms; def2-QZVP was used for the S atom; and 6–31G(d) was used for the N atom. A small basis set on the N atom is necessary for the correct calculations of the *g*-factor and hyperfine constants (*hfc*).^58,59^ When using a larger basis set for the N atom, e.g., EPR-III or def2-QZVP, the obtained results systematically underestimate the *hfc*. Calculations were performed on Gaussian version 16.C01^60^ using the advanced computing service (clusters Isabella and Supek) provided by the University of Zagreb University Computing Centre (SRCE)^61^ and the computational resources of the PharmInova project (sw.pharma.hr) at the University of Zagreb Faculty of Pharmacy and Biochemistry.^62^

Electronic transition spectra were calculated at the gas phase and in acetonitrile with the time-dependent^63^ CAM-B3LYP^64^/TZVP/PCM^51^ method at the molecular geometries optimized at the B3LYP/TZVP level.

To account for the entropic effect of the presence of solvent molecules around a solute, the cell model presented by Ardura et al. was used.^65^ This model is proposed to explicitly evaluate the effect of the loss of translation degrees of freedom in solution on the Gibbs activation energy in a bimolecular (or higher order of molecularity) reaction.^66^

We performed kinetic modeling of the reaction pathways, with the full model described in detail in Section S12 of the Supporting Information. The complete mathematical model, which incorporates all possible reaction steps, leads to a system of nonlinear differential equations that is exceedingly complex and likely impossible to solve analytically due to the nonlinearity and the interdependence of the species’ concentrations.^67−70^ Numerical methods like Runge–Kutta could be applied; however, the potential solution may be highly sensitive to the initial conditions, particularly to the concentration of radicals formed after the laser pulse.^71,72^ In such calculations, the uncertainty in starting conditions may induce numerical instability.^69,73,74^ Therefore, some necessary approximations were applied to reduce the complexity of the model.

## Data Availability

The data underlying this study are available in the published article and its Supporting Information.

## Supporting Information Available

Supporting information with experimental details, synthesis procedure, reactant and product characterization, EPR simulation parameters, calculation procedures, geometries and energies of optimized structures, and recorded NMR and EPR spectra (PDF)
