# Supplementary material for: Mechanistic Insights into the Propagation Cycle of the Hofmann–Löffler–Freytag Reaction: Halogen vs Hydrogen Atom Transfer
Source: J Org Chem. 2025 Apr 1;90(14):4873–87. doi: 10.1021/acs.joc.4c02997 (PMC11998013; doi:10.1021/acs.joc.4c02997)
Supplement: Supplementary file 1 — jo4c02997_si_001.pdf [file jo4c02997_si_001.pdf]

SUPPORTING INFORMATION

Mechanistic Insights into the Propagation Cycle of the Hofmann–Löffler–Freytag Reaction. Halogen *vs* Hydrogen Atom Transfer

*Gabrijel Zubčić,[1] Luka Andrijanić,[2] Iva Džeba,[3] Jiangyang You,[3] Tomislav Friganović,[2] Tomislav Portada,[3] Kristina Pavić,[1] Erim Bešić,[1] Valerije Vrček,[1] and Davor Šakić[1],**

[1] University of Zagreb Faculty of Pharmacy and Biochemistry, Ante Kovačića 1, Zagreb 10000, Croatia

[2] University of Zagreb Faculty of Science, Horvatovac 102 a, Zagreb 10000, Croatia

[3] Ruđer Bošković Institute, Bijenička cesta 54, Zagreb 10000, Croatia

AUTHOR INFORMATION

Corresponding Author

*Davor Šakić − University of Zagreb Faculty of Pharmacy and Biochemistry, Ante Kovačića 1, 10000 Zagreb, Croatia; orcid.org/0000-0002-8871-6622; e-mail: davor.sakic@pharma.unizg.hr

Table of Contents

S1. Summary of the Experimental Methods 3

S2. Synthesis procedure for preparation of N-chlorosulfonamides with trichloroisocyanuric acid. 6

S3. Degassing procedure and EPR measurements 10

S4. EPR measurements of N-Cl in toluene 11

S5. EPR measurements of N-Cl in n-heptane 15

S6. EPR measurements of N-Cl in acetonitrile 17

S7. NMR spectra of N-Cl reaction mixture under irradiation in toluene 20

S8. Laser Flash Photolysis Measurements 24

S9. UV calculations 28

S10. DFT results and optimized geometries 42

S11. EPR calculations of PBN adducts 203

S12. Kinetic modeling 227

S13. References 242

S1. Summary of the Experimental Methods

The purchased compounds were sourced from Kefo [sulfuric acid (98%), methanol, petroleum ether, p-toluenesulfonyl chloride, silica gel, pyridine, silver acetate, ethyl acetate, cyclohexane, trifluoroacetic anhydride, toluene, and trichloroisocyanuric acid], Ru-Ve [hydrochloric acid (37%), acetone, silicon oil, petroleum ether, and cyclohexane], and Biovit [toluene (anhydrous), acetonitrile (anhydrous), 1,4-dioxane (anhydrous), tetrahydrofuran (anhydrous), N,N-dimethylformamide (anhydrous), N,N-dimethylacetamide, 1,2-dichloroethane (anhydrous), and dichloromethane (anhydrous)]. All reagents and chemicals were obtained commercially and used without further purification, unless otherwise noted.

Chromatographic purification of the products was carried out using column chromatography filled with silica gel (Macherey-Nagel) 0.063–0.2 mm, and appropriate solvent mixtures were used as eluents: petroleum ether/ethyl acetate. Thin-layer chromatography (TLC) was performed on precoated TLC plates ALUGRAM SIL G/UV254, 0.20 mm silica gel 60 with a fluorescent indicator UV254 (Macherey-Nagel) in the appropriate solvent system. TLC spots were observed after illumination with UV light at a wavelength of 254 nm and after immersion in an aqueous solution of $KMnO_4$ (3 g $KMnO_4$, 20 g K_2CO_3, 5 mL aq. NaOH 5%, and 300 mL water) followed by heating. If TLC spots were not visible after illumination with UV light, they were detected utilizing an iodine chamber.

NMR spectra of the reaction mixture were obtained on a Varian Inova 400 NMR spectrometer operating at 399.90 MHz for 1H NMR and 100.6 MHz for 13C NMR and are reported as chemical shifts (δ) in parts per million (ppm). Spectra were referenced internally according to residual solvent signals (^{1}H: $CDCl_3$, 7.26 ppm; ^{13}C: $CDCl_3$, 77.0 ppm;). Spectra were imported and processed in the MestreNova 14.2.0 program. Data for NMR spectra use the following abbreviations to describe multiplicity: s, singlet; br s, broad singlet; d, doublet; t, triplet; q, quartet; dd, doublet of doublets; td, triplet of doublets; ddd, doublet of doublet of doublets; ddt, doublet of doublet of triplets; app dd, apparent doublet of doublets; m, multiplet. Coupling constant (J) are reported in units of Hertz (Hz). The spectra were imported and processed in the MestreNova 11.0.4 program.[1] Structural assignments were made with additional information from gCOSY, gHSQC, and gHMBC experiments.

EPR spectroscopy was done by using a Bruker E500 ELEXSYS EPR spectrometer with an ER4122SHQE cavity resonator. As this cavity resonator does not have an optical window for illumination, the light source was mounted underneath the cavity, with light coming through the bottom of the EPR 4 mm-inner-diameter tube. EPR deconvolution and simulation was done

using an EasySpin module with the MATLAB program package.[2] EPR visualization and spectroscopy were done using the VisualEPR Web page.[3]

For experiments, 7 mg of **N-Cl** was dissolved in the 0.4 mL of solvent (~0.05 M), degassed (see later), and then mixed with degassed 5 mg of **PBN** dissolved in 0.2 mL of the same solvent in threefold excess (~0.15 M).

Irradiation was performed *in situ* (EPR) and *off-site* (NMR) with Kessil PR-160L 370 ± 10 nm gen-2 LED UV, with average intensity 137 mW/cm^2 when sample is 6 cm from the lamp, according to manufacturer.[4]

Transient absorption spectroscopy (TAS) measurements were performed using a nanosecond laser flash photolysis setup. The setup consists of a Nd:YAG laser (Quantel, Q-smart 450) and an LP980 transient absorption spectrometer (Edinburgh Instruments). The ground state absorption of the samples was adjusted to 0.3 at the 266 nm laser excitation wavelength (5 ns pulse duration, 10 Hz). The laser energy at 266 nm was in the range of 10–23 mJ (30–70 mJ cm^{-2}). Kinetic measurements were performed in 1 cm quartz cells sealed with rubber septa. The transient absorption spectra were measured in the flow cell, with a flow rate set to 2.4 mL/min to ensure that no light was absorbed by the photoproducts. All solutions were prepared immediately before the experiments. Solutions were purged with high purity N_2 for 20 min prior to the kinetics measurements and for 1 h before spectra measurements. All measurements were performed at 25°C. UV–Vis spectra of the sample solutions were recorded using a Varian Cary 4000 spectrophotometer (Figures S1-S3).

The conformational space for all the local minima and local maxima on the energy diagram was sampled and investigated using the Conformer–Rotamer Ensemble Sampling Tool─CREST[5] coupled with the xtb-GFN2 program package and MD simulation using xtb-GFN1[6] and xtb-GFN2.[7] The obtained structures were reoptimized using the B3LYP/6-31G(d) level of theory.[8–10] For each structure with a stable wave function, frequency calculation was performed to identify the minima and transition-state structures. From all of the conformers of transition-states, an intrinsic reaction coordinate search was performed to characterize the corresponding reaction and product complexes/reactive conformers, the last point in the forward and reverse direction was then optimized to the nearest local minimum i.e. reactive complex. Single point energies were obtained with universal continuum solvation model SMD[11] and RO-B2PLYP[13,14] with a G3MP2 large basis set[15] on geometries obtained at the B3LYP/6-31G(d) level of theory, with additional D3 dispersion correction.[16] Zero Point Energy (ZP) calculated for gas-phase structures were used for correction in solvent.

Calculations of EPR parameters were done using the B3LYP functional and the mixed basis set: EPR-III for C, H, and O atoms, def2-QZVP for the S atom, and 6-31G(d) for the N atom. A small basis set on the N atom is necessary for the correct calculations of the g-factor and hfcs.[17,18] When using a larger basis set for the N atom, e.g., EPR-III or def2-QZVP, the obtained results systematically underestimate the hfc. Calculations were performed on the Gaussian version 16.C01[19] using the advanced computing service (clusters Isabella and Supek) provided by the University of Zagreb University Computing Centre─SRCE[20] and the computational resources of the PharmInova project (sw.pharma.hr) at the University of Zagreb Faculty of Pharmacy and Biochemistry.[21]

Electronic transition spectra were calculated at the gas phase and in acetonitrile with Time Dependent[22] CAM-B3LYP[23]/TZVP/PCM[12] method at the molecular geometries optimized at B3LYP/TZVP level.

In order to account for the entropic effect of the presence of solvent molecules around a solute, the cell model presented by Ardura et al. was used.[24] This model is proposed in order to explicitly evaluate the effect of the loss of translation degrees of freedom in solution on the Gibbs activation energy in bimolecular (or higher order of molecularity) reaction.[25]

We performed kinetic modelling of the reaction pathways, with the full model described in detail in subsection S12 of the Supporting Information. The complete mathematical model, which incorporates all possible reaction steps, leads to a system of nonlinear differential equations that is exceedingly complex and likely impossible to solve analytically due to the nonlinearity and the interdependence of the species' concentrations.[26–29] Numerical methods like Runge-Kutta could possibly be applied; however, the potential solution may be highly sensitive to the initial conditions, particularly to the concentration of radicals formed after the laser pulse.[30,31] In such calculations, the uncertainty in starting conditions may induce numerical instability.[28,32,33] Therefore, some approximations were necessary to reduce the complexity of the model.

S2. Synthesis procedure for preparation of *N*-chlorosulfonamides with trichloroisocyanuric acid.

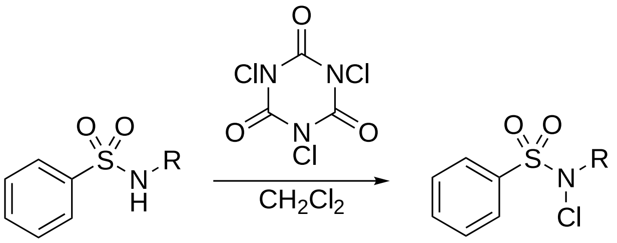

A round bottom flask with a magnetic stir bar was charged with 1.5 equiv (2.3 mmol) of trichloroisocyanuric acid (TCICA), providing 4.5 equiv of chlorine atoms, then evacuated and backfilled with N_2. Anhydrous CH_2Cl_2 was then added to create a suspension. Sulfonamide (**N-H**, 1.5 mmol, 1.0 equiv) was then added to the suspension *via* syringe. The suspension was stirred at room temperature and monitored by TLC until complete consumption of starting material was observed.

Upon complete consumption of starting material as judged by TLC, the reaction was diluted with H_2O and the biphasic suspension was transferred to a separatory funnel. The reaction flask was rinsed with CH_2Cl_2 (5 mL) to ensure quantitative transfer. The organic phase was separated, and the aqueous phase was extracted three times with CH_2Cl_2. The combined organic phases were dried with Na_2SO_4, filtered, and concentrated under reduced pressure. The crude material was purified by silica gel column chromatography eluting with a Cyclohexane:EtOAc (85:15) solvent system as noted below.

4-methyl-*N*-(5-phenylpentyl)benzenesulfonamide **N-H**

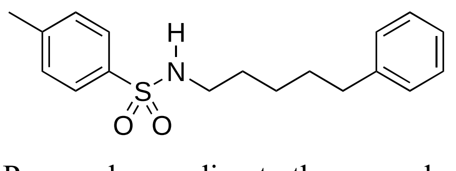

Prepared according to the general procedure described in *J. Phys. Chem. A* **2024**, 128, 13, 2574–2583. Briefly, to a round bottom flask with a magnetic stir bar, 259 mg of 5-phenylpentyl-1-amine (M_r = 163.26, 1.59 mmol) was added to the 20 mL of CH_2Cl_2. The solution was cooled in the ice bath, and while stirring, 161 mg (1,59 mmol) of triethylamine (M_r = 101.193) was added, followed by addition of 303 mg (1.59 mmol) tosyl chloride (M_r = 190.64) dissolved in the 5 mL of CH_2Cl_2. After three hours, the reaction was quenched with 10 % HCl(aq), 30 ml of water, neutralized with 5 % $NaHCO_3$(aq), extracted in DCM, and dried over Na_2SO_4. After evaporation, clear oil was isolated with traces of unreacted tosyl

chloride. After purification *via* subsequent filtration over cellulose filter, 425 mg (84 %) of N-H was obtained.

^{1}H NMR (400 MHz, $CDCl_3$) δ 7.67 (d, *J* = 8.3 Hz, 2H), 7.23 (d, *J* = 7.8 Hz, 2H), 7.21 – 7.16 (m, 2H), 7.10 (t, *J* = 7.4 Hz, 1H), 7.05 (d, *J* = 6.7 Hz, 2H), 4.30 (t, *J* = 6.2 Hz, 1H), 2.85 (q, *J* = 7.1 Hz, 2H), 2.48 (t, *J* = 7.6 Hz, 2H), 2.35 (s, 3H), 1.54 – 1.45 (m, 2H), 1.44 – 1.33 (m, 2H), 1.26 – 1.17 (m, 2H).

^{13}C{^{1}H} NMR (101 MHz, $CDCl_3$) δ 143.3, 142.2, 136.9, 130.2, 129.6, 128.3, 127.1, 125.7, 43.1, 35.6, 30.8, 29.4, 26.1, 21.5

This checks out with earlier work by Mũniz et al. (10.1021/acscatal.7b00928).

^{1}H NMR (500 MHz, CDCl3): δ = 7.74 (d, *J* = 8.3 Hz, 2H), 7.30 (dd, *J* = 8.6, 0.7 Hz, 2H), 7.28-7.25 (m, 2H), 7.19-7.16 (m, 1H), 7.13-7.12 (m, 2H), 4.41 (t, *J* = 6.2 Hz, 1H), 2.92 (td, *J* = 7.1, 6.1 Hz, 2H), 2.58-2.52 (m, 2H), 2.42 (s, 3H), 1.59-1.45 (m, 6H), 1.32-1.26 (m, 2H).

^{13}C{^{1}H} NMR (126 MHz, CDCl3): δ = 143.4, 142.2, 137.0, 129.7, 128.3, 128.3, 127.1, 125.8, 43.1, 35.7, 30.8, 29.5, 26.1, 21.5

N-chloro-4-methyl-*N*-(5-phenylpentyl)benzenesulfonamide **N-Cl**

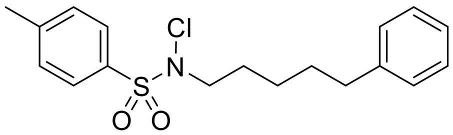

Prepared from *N*-chloro-4-methyl-*N*-(5-phenylpentyl)benzenesulfonamide (570 mg, 1.69 mmol) and TCICA (698 mg, 3 mmol) following general procedure A. The product was obtained as white solid (558 mg, >99% yield) after silica gel flash column chromatography using cyclohexane:EtOAc (85:15).

^{1}H NMR (400 MHz, toluene): δ 7.70 (d, *J* = 8.3 Hz, 2H), 7.19 – 6.92 (m, 8H), 6.78 (d, *J* = 8.2 Hz, 2H), 3.06 (t, *J* = 6.8 Hz, 2H), 2.37 (t, *J* = 7.7 Hz, 2H), 1.91 (s, 3H), 1.48 – 1.34 (m, 4H), 1.22 – 1.07 (m, 2H).

^{13}C{^{1}H} NMR (101 MHz, toluene): δ 134.4, 134.2, 133.8, 133.3, 133.2, 132.8, 132.0, 130.6, 61.3, 40.6, 35.8, 31.9, 30.6, 25.4, 25.2, 25.0, 24.8, 24.6.

a)
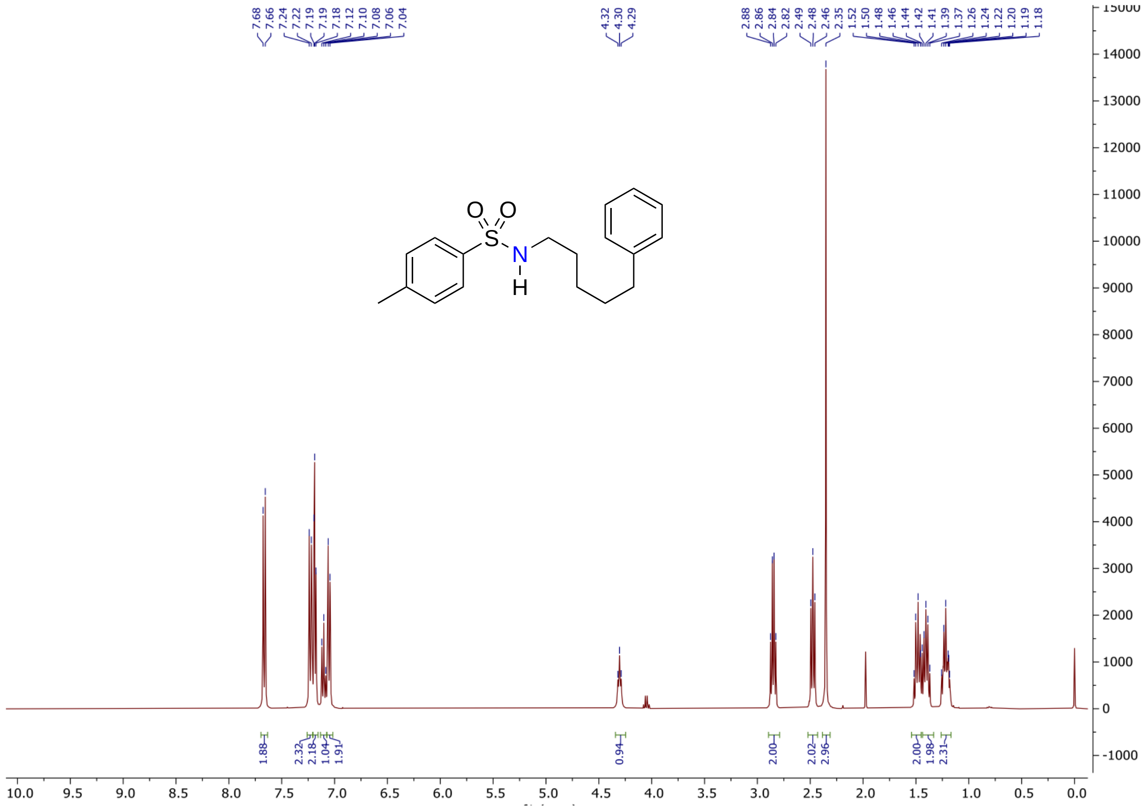

b)
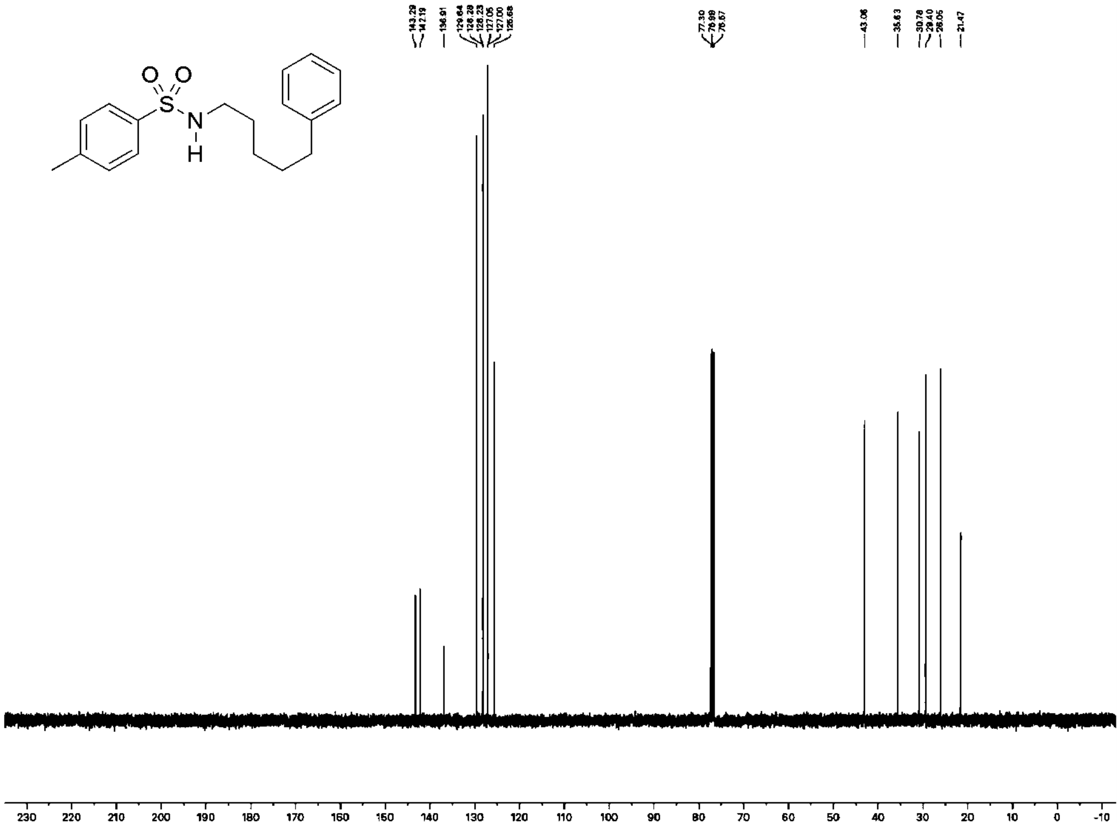

Figure S1. a) ^{1}H NMR spectra and b) 13C{^{1}H} NMR spectra of **N-H**, $CDCl_3$, 400 MHz

a)

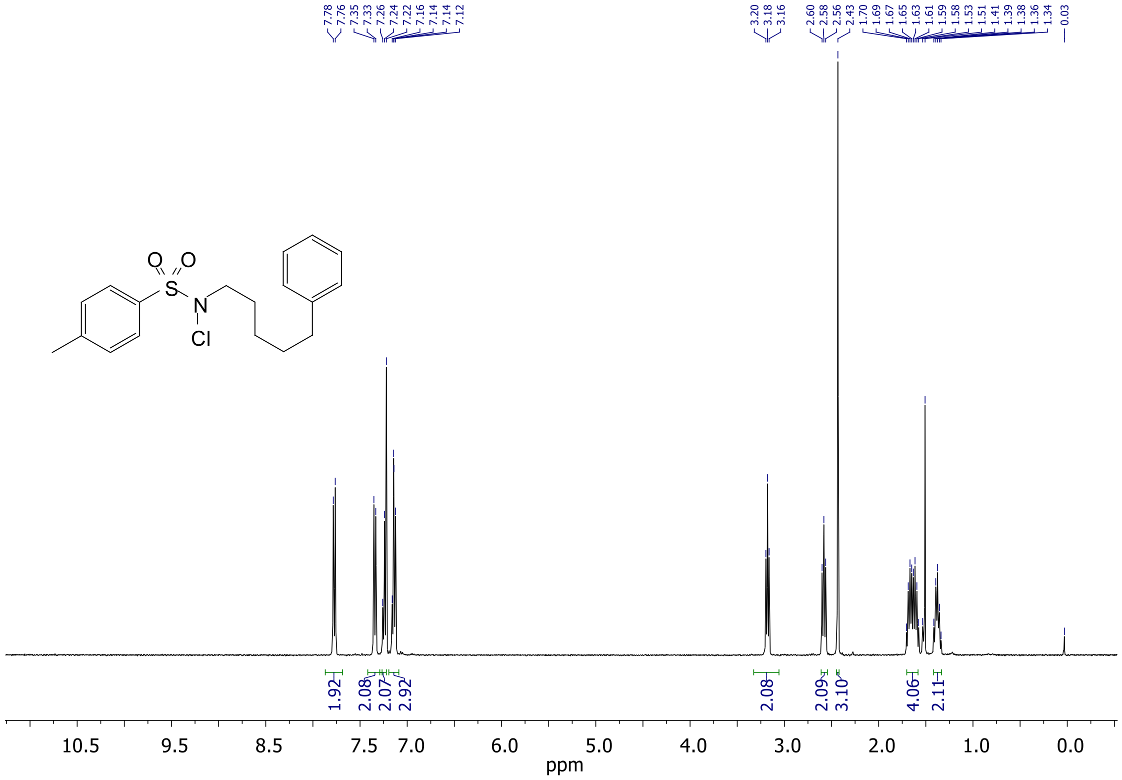

b)

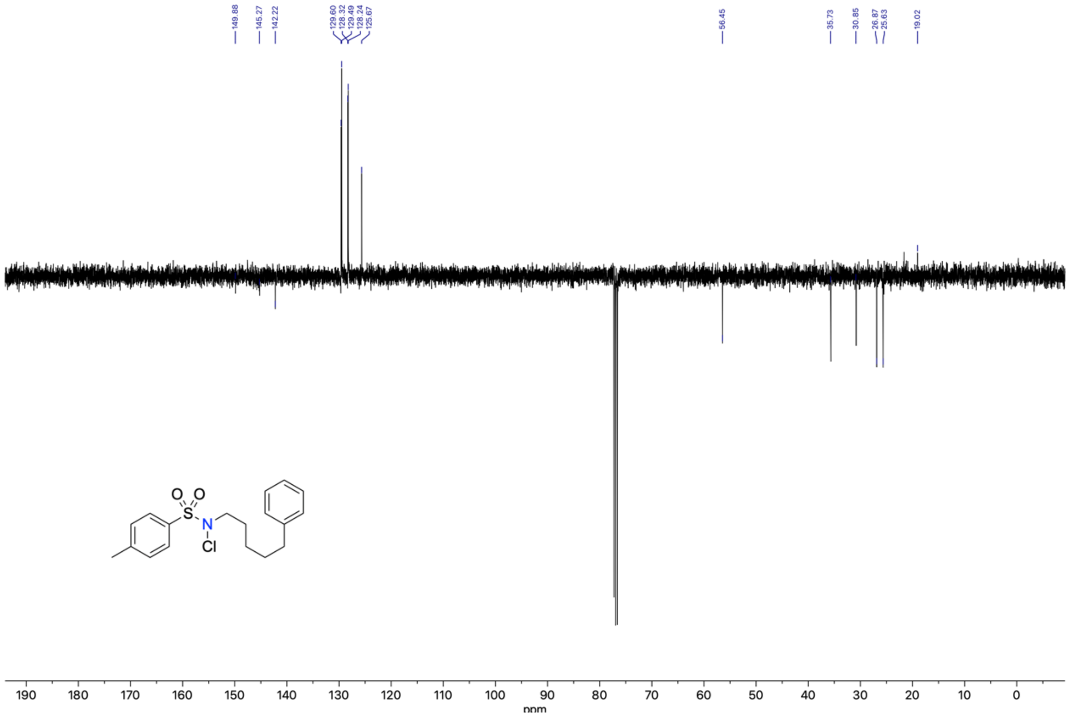

Figure S2. a) ^{1}H NMR spectra and b) 13C{^{1}H} NMR spectra of **N-Cl**, $CDCl_3$, 400 MHz

S3. Degassing procedure and EPR measurements

The vacuum line was turned on preferably the day before use (this is only necessary for working on high vacuum, 10^{-6} mm Hg, with the use of an additional diffusion pump otherwise it can be turned on the day of use, at least 2 hours before usage). After every usage of the manifold, trap of the vacuum line was disassembled and washed with methanol while the vacuum grease was removed with isopropanol. Trap was then dried in a drying oven. For the preparation of working solutions, Schlenk flasks were used. First step in sample preparation was stock solution preparation in the following manner. Compound (approx. 30 mg) and spin trap (PBN) (approx. 10 mg) were weighed separately on an analytical balance and transferred quantitively to corresponding Schlenk flasks. To each flask approx. 0.3 mL of solvent (toluene or heptane) was added. Schlenk flask was then attached to the vacuum line *via* a joint that has been greased. The joint was covered with parafilm and fixed with a suitable plastic extension to ensure it was hermetically sealed. The stopcock of the Schlenk flask was then closed. Flask was emersed in liquid nitrogen and evacuated by opening of the main cap on the vacuum line and the cap leading to the flask. Vacuum was then created above the frozen liquid. Liquid nitrogen was removed from underneath the flask and the cap leading to the flask was closed while the solution was left to defrost. Once the solution was defrosted liquid nitrogen was added again. As soon as the solution was frozen again the cap leading to the flask was opened. Whole cycle was repeated as many times as necessary for each stock solution. After degassing, positive pressure of nitrogen gas was created by opening of the stopcock, the flask was then detached from the vacuum line and closed with an appropriate plug. The same procedure was done for the solution of PBN in solvent. Finally, both flasks were transferred to a glovebox in which they were mixed by transferring a small amount to an EPR tube.

S4. EPR measurements of **N-Cl** in toluene

Two different stock solutions were prepared. Into the first flask *N*-chloro-4-methyl-*N*-(5-phenylpentyl)benzensulfonamide **N-Cl** (38 mg, 0.012 mmol) and toluene (0.3 mL) were added. Into the second flask PBN (7 mg, 0.004 mmol) and toluene (0.3 mL) were added. Inside a glovebox, small volumes from both solutions were transferred to an EPR tube which was then used for the measurement.

The deoxygenated solutions were sealed in the Schlenk flasks and transferred into a glovebox charged with nitrogen gas (an erLab Captair 2200A pyramid glove bag charged with nitrogen. The glove bag was then charged and discharged multiple times until the oxygen concentration reached below one percent of the air level). Small volumes of both solutions, typically 0.1 mL (actually amounts depend on the solution available in each Schlenk flask), were pipetted into one 5 mm PYREX NMR tube under the nitrogen atmosphere and sealed by a Sigma-Aldrich Precision Seal rubber septa plug. The EPR measurements were performed on a Bruker ELEXSYS E500 spectrometer with an ER049X (superX) microwave bridge and an original ER4122SHQE cavity resonator. In-situ UV light illumination was provided by a Kessil 370 nm gen 2 LED UV lamp from the bottom port of the resonator. The spectra were usually collected shortly before, during, and after UV illumination as a two-dimensional time delay experiment. Typical measurement parameters are as follows: microwave frequency ∼9.85 GHz (as recorded by the spectrometer), microwave power 10 mW, modulation frequency 100 kHz, modulation amplitude 0.1 G, lock-in amplifier time constant 10.24 ms, sweep range 60 G, sweep time 20.97 s, one sweep at each time point, and 5s waiting time between points. The total number of points was usually set to cover(much) longer than the time needed for the main reaction to finish. (The receiver gain needed to be lowered from 60 dB initially to 40 dB near the later peak of the oxo-PBN signal to avoid saturation.)

The thermal drifts of the magnetic field were compensated in the post processing by aligning the points using Cl-PBN and/or oxo-PBN signals as internal standard(s). Simulations were performed on averages exacted from the relevant period(s) of the aligned spectra using the EASYSPIN package. Experimental and simulated spectra are denoted at the bottom as blue and purple traces, respectively. Another, approximately constant spectrometer bias ($\Delta g \approx$-0.000205) was calibrated by measuring standard samples solid 2,2-diphenyl-1-picrylhydrazyl (DPPH) radical (Bruker, 0103D139) and the aqueous solution of the peroxylamine disulfonate (PADS, i.e., Frémy's salt) radical offline and used to produce the g-factors listed.

Resulting spectra can be decomposed in the following manner

1) The best decomposition of the **N-Cl** EPR spectra includes one Cl-centred radical abduct , one N-centered radical abduct, with triplet from N-atom in PBN, doublet from Hα-atom in PBN, and triplet splitting from N-atom from the **PBN** (Here splitting from H-atom ($\alpha_{H,exp}$) is larger than splitting from N-atom ($\alpha_{N\prime,exp}$) of **PBN**, due to larger spin population on H-atom), and three C-centered radical abducts with different hydrogen hyperfine couplings. This is shown below and in **Figures S16, S18 and S20**. This deconvolution has a strong foundation in extensive quantum-chemical calculations (see EPR calculations on PBN adducts section in the SI)

Cl-PBN adduct was first identified in the spectra. EPR parameters of this radical adduct correspond to the literature references.[28] Shown as orange trace in **Figure S16.**

Simulated values: g_{exp}= 2.0077, $\alpha_{N,exp}$ = 12.37 G, $\alpha_{Cl,exp}$ = 6.23 G, and $\alpha_{H,exp}$ = 0.76 G.

N-PBN is characterized by large triplet splitting coming from N-atom in PBN. Doublet splitting comes from H-atom of PBN, while third triplet splitting comes from N-atom in from fragment stemming from **7**. Shown as green trace in **Figure S16.**

Simulated values: g_{exp}= 2.0063, $\alpha_{N,exp}$ = 14.21 G, $\alpha_{H,exp}$ = 3.87 G, and $\alpha_{N\prime,exp}$ = 1.57 G.

C5-PBN is a *C*-centered alkyl radicals. Shown as purple trace.

Simulated values: g_{exp}= 2.0064, $\alpha_{N,exp}$ = 13.96 G, $\alpha_{H,exp}$ = 2.03 G.

C6-PBN is a *C*-centered benzyl radical. Shown as red trace.

Simulated values: g_{exp}= 2.0064, $\alpha_{N,exp}$ = 13.96 G, $\alpha_{H,exp}$ = 3.06 G

C2-PBN is a *C*-centered radical adjacent to amide. Shown as brown trace.

Simulated values: g_{exp}= 2.0062, $\alpha_{N,exp}$ = 13.72 G, $\alpha_{H,exp}$ = 7.38 G.

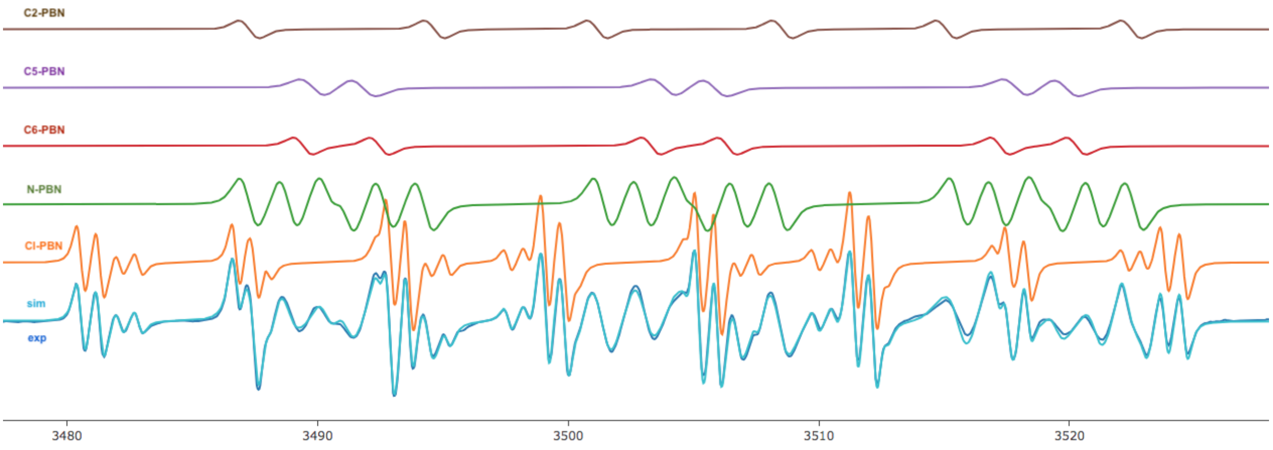

Figure S3. EPR spectra of spin-trapped radical intermediates generated with 370 nm irradiation of **N-Cl** in toluene. Experimental spectra is in blue color, while green, red, purple and brown correspond to simulated spectra for **N-PBN**, **C6-PBN**, **C5-PBN** and **C2-PBN** respectively.

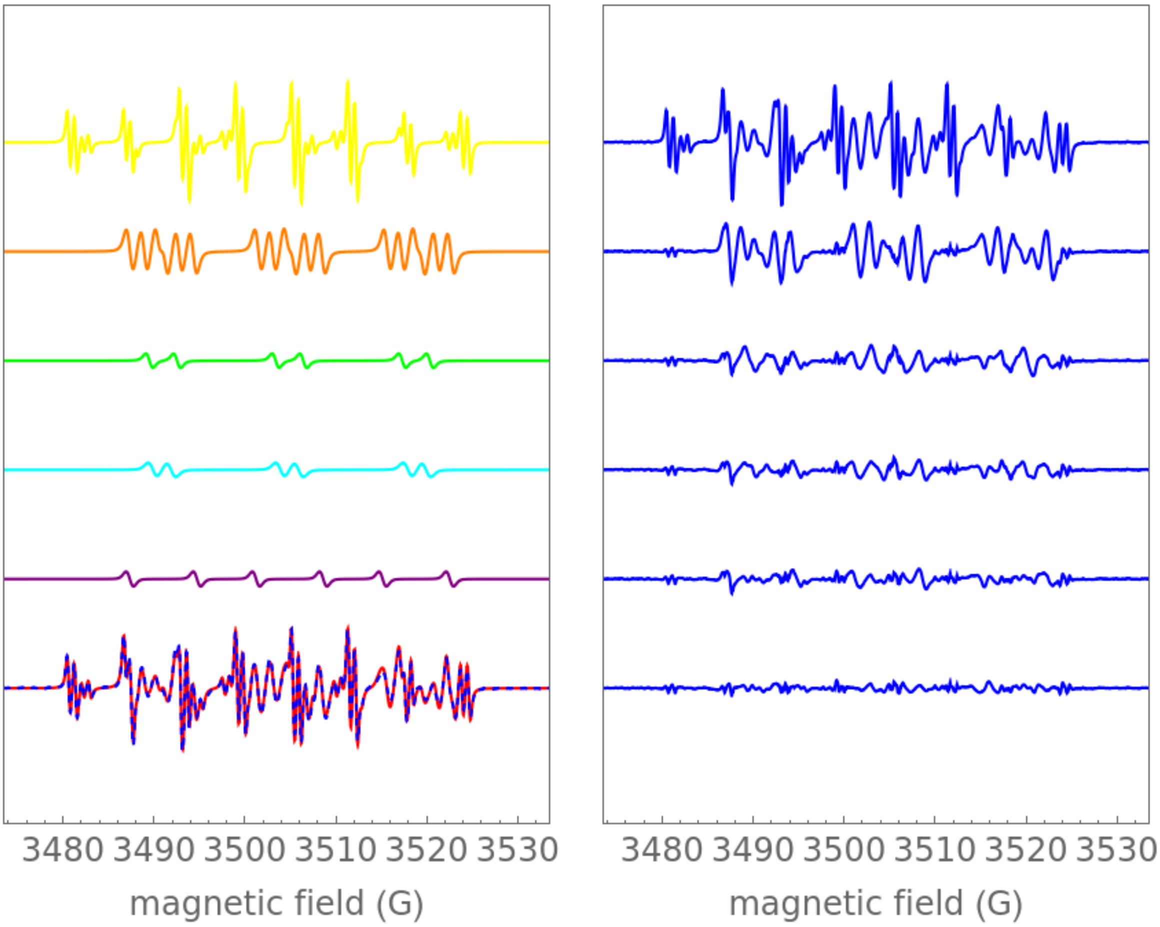

Figure S4. The deconvolution components and residues of the EPR spin abduct spectrum of **N-Cl** spin trapping experiment in toluene. Left panel shows, from upper to lower, **Cl-PBN** (yellow), **N-PBN** (orange), **C_6-PBN** (green), **C_5-PBN** (cyan), and **C_2-PBN** (purple) components of the deconvolution, as well as a comparison between the total simulated spectrum (red) and the experimental spectrum (blue, dashed) at the bottom. Right panel shows from upper to lower the experimental spectrum and the residue after each of the deconvolution components on the right panel is subtracted.

S5. EPR measurements of **N-Cl** in n-heptane

Two different stock solutions were prepared. Into the first flask *N*-chloro-*N*-hexyl-4-methylbenzenesulfonamide **N-Cl** (38 mg, 0.012 mmol) and n-heptane (0.3 mL) were added. Into the second flask PBN (10 mg, 0.0056 mmol) and n-heptane (0.3 mL) were added. Both solutions were deoxygenated by the freeze-pump-thaw method already described.

N-PBN is characterized by large triplet splitting coming from N-atom in PBN. Doublet splitting comes from H-atom of PBN, while third triplet splitting comes from N-atom in from fragment stemming from **7**. Shown as green trace in **Figure S18.**

Simulated values: g_{exp}=2.0064, $\alpha_{N,exp}$ = 14.14 G, $\alpha_{H,exp}$ = 3.95 G, and $\alpha_{N\prime,exp}$ = 1.58 G.

C5-PBN is a *C*-centered alkyl radicals. Shown as purple trace.

Simulated values: g_{exp}=2.0064, $\alpha_{N,exp}$ = 13.85 G, $\alpha_{H,exp}$ = 1.89 G.

C6-PBN is a *C*-centered benzyl radical. Shown as red trace.

Simulated values: g_{exp}= 2.0064, $\alpha_{N,exp}$ = 13.94 G, $\alpha_{H,exp}$ = 2.97 G

C2-PBN is a *C*-centered radical adjacent to amide. Shown as brown trace.

Simulated values: g_{exp}= 2.0064, $\alpha_{N,exp}$ = 14.14 G, $\alpha_{H,exp}$ = 7.38 G.

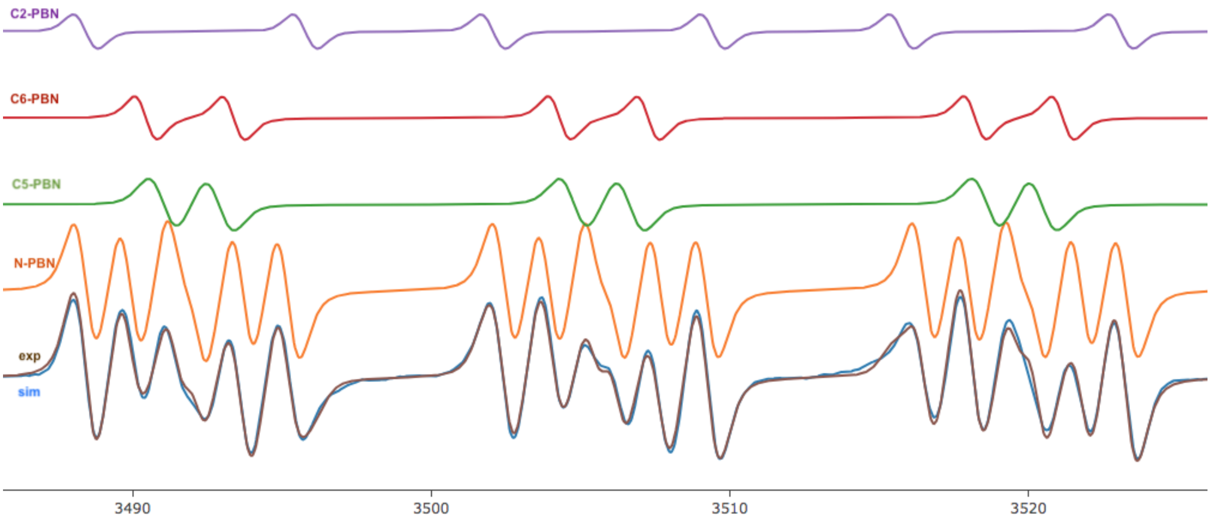

Figure S5. EPR spectra of spin-trapped radical intermediates generated with 370 nm irradiation of **N-Cl** in toluene. Experimental spectra is in brown color, while orange, green, red and purple correspond to simulated spectra for **N-PBN**, **C5-PBN**, **C6-PBN** and C2-**PBN** respectively.

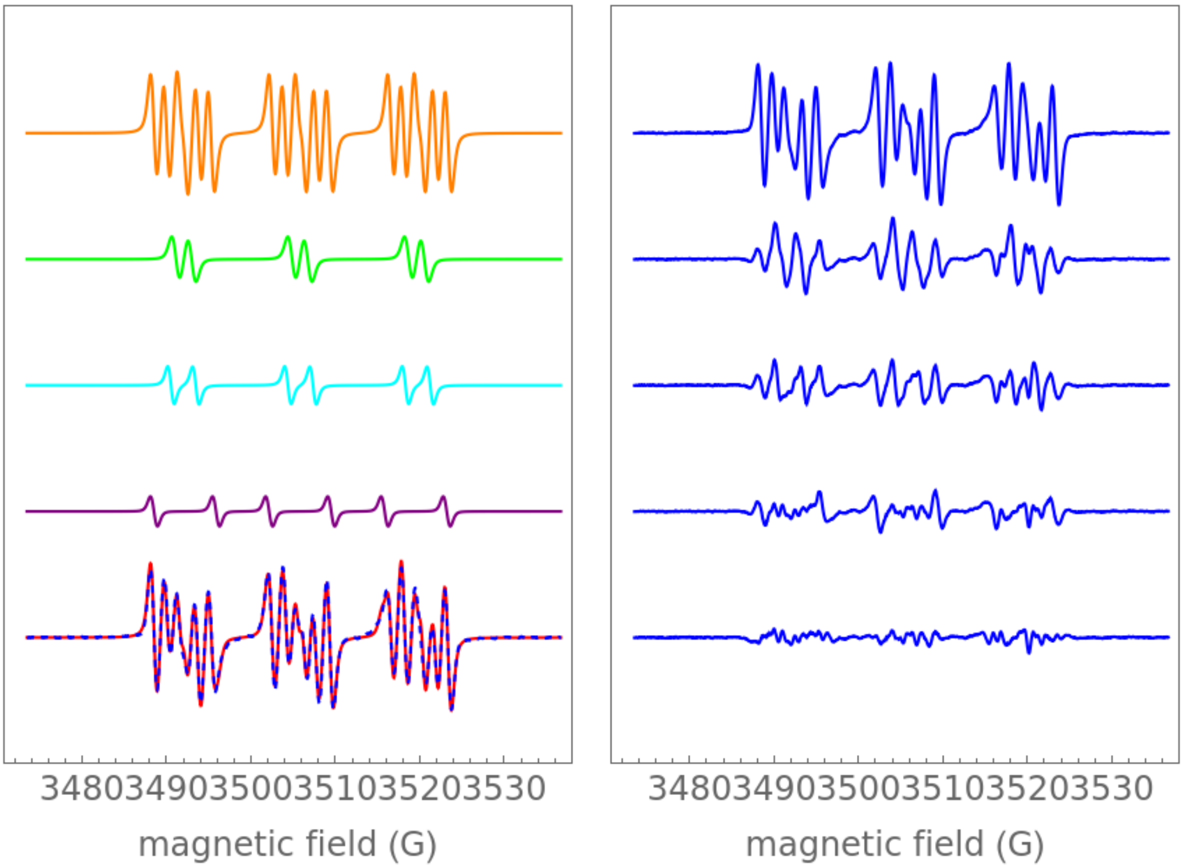

Figure S6. The deconvolution components and residues of the EPR spin abduct spectrum of **N-Cl** spin trapping experiment in n-heptane. Left panel shows, from upper to lower, the **N-PBN** (orange), **C_5-PBN** (green), **C_6-PBN** (cyan), and **C_2-PBN** (purple) components of the deconvolution, as well as a comparison between the total simulated spectrum (red) and the experimental spectrum (blue, dashed) at the bottom. Right panel shows from upper to lower the experimental spectrum and the residue after each of the deconvolution components on the right panel is subtracted.

S6. EPR measurements of **N-Cl** in acetonitrile

Two different stock solutions were prepared. Into the first flask *N*-chloro-*N*-hexyl-4-methylbenzenesulfonamide **N-Cl** (4.3 mg, 0.0012 mmol) was dissolved in 0.5 mL acetonitrile. The solution was deoxygenated by the freeze-pump-thaw method on a Schlenk line. PBN (1.0 mg, 0.00056 mmol) was dissolved in 0.084 mL of pre-deoxygenated acetonitrile under nitrogen. Two components are mixed by a 3:1 ratio and loaded into one 2 mm quartz EPR tube (Wilmadlabglass 712-SQ-100M) until the liquid column saturates the resonator active window under nitrogen. The EPR tube was then closed by a rubber cap for the experiment.

Cl-PBN adduct was first identified in the spectra. EPR parameters of this radical adduct correspond to the literature references.[28] Shown as orange trace in **Figure S18.**

Simulated values: g_{exp}= 2.0074, $\alpha_{N,exp}$ = 12.66 G, $\alpha_{Cl,exp}$ = 6.30 G, and $\alpha_{H,exp}$ = 0.80 G.

N-PBN is characterized by large triplet splitting coming from N-atom in PBN. Doublet splitting comes from H-atom of PBN, while third triplet splitting comes from N-atom in from fragment stemming from **7**. Shown as green trace in **Figure S18.**

Simulated values: g_{exp}= 2.0061 , $\alpha_{N,exp}$ = 14.21 G, $\alpha_{H,exp}$ = 3.87 G, and $\alpha_{N',exp}$ = 1.57 G.

C5-PBN is a *C*-centered alkyl radicals. Shown as purple trace.

Simulated values: g_{exp}= 2.0061, $\alpha_{N,exp}$ = 14.37 G, $\alpha_{H,exp}$ = 1.98 G.

C6-PBN is a *C*-centered benzyl radical. Shown as red trace.

Simulated values: g_{exp}= 2.0061 , $\alpha_{N,exp}$ = 14.22 G, $\alpha_{H,exp}$ = 3.23 G

C2-PBN is a *C*-centered radical adjacent to amide. Shown as brown trace.

Simulated values: g_{exp}=2.0061, $\alpha_{N,exp}$ = 13.66G, $\alpha_{H,exp}$ = 7.69 G.

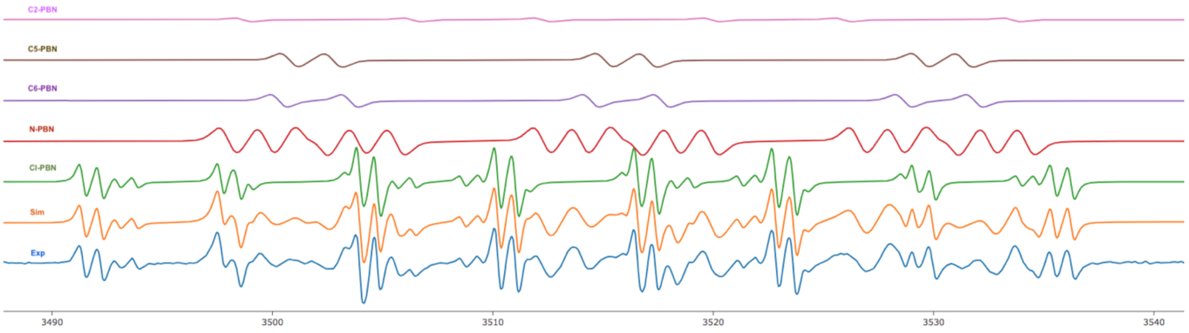

Figure S7. EPR spectra of spin-trapped radical intermediates generated with 370 nm irradiation of **N-Cl** in toluene. Experimental spectra is in brown color, while orange, green, red and purple correspond to simulated spectra for **N-PBN**, **C5-PBN**, **C6-PBN** and **C2-PBN** respectively.

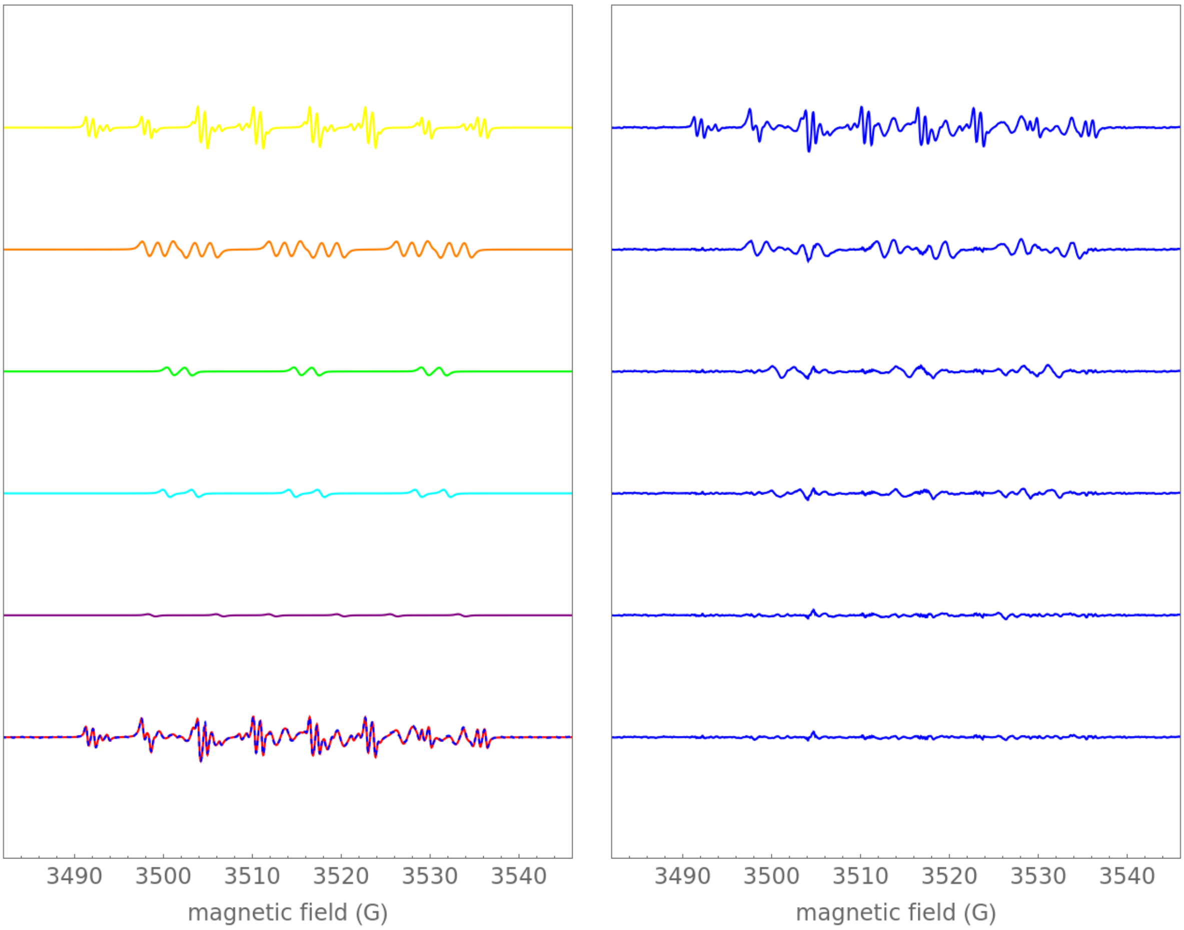

Figure S8. The tentative deconvolution components and residues of the EPR spin abduct spectrum of **N-Cl** spin trapping experiment in acetonitrile. Left panel shows, from upper to lower, **Cl-PBN** (yellow), **N-PBN** (orange), **C_5-PBN** (green), **C_6-PBN** (cyan), and **C_2-PBN** (purple) components of the deconvolution, as well as a comparison between the total simulated spectrum (red) and the experimental spectrum (blue, dashed) at the bottom. Right panel shows from upper to lower the experimental spectrum and the residue after each of the deconvolution components on the right panel is subtracted. The **C_2-PBN** component of this deconvolution is very weak, so it is not sure if we really observed it.

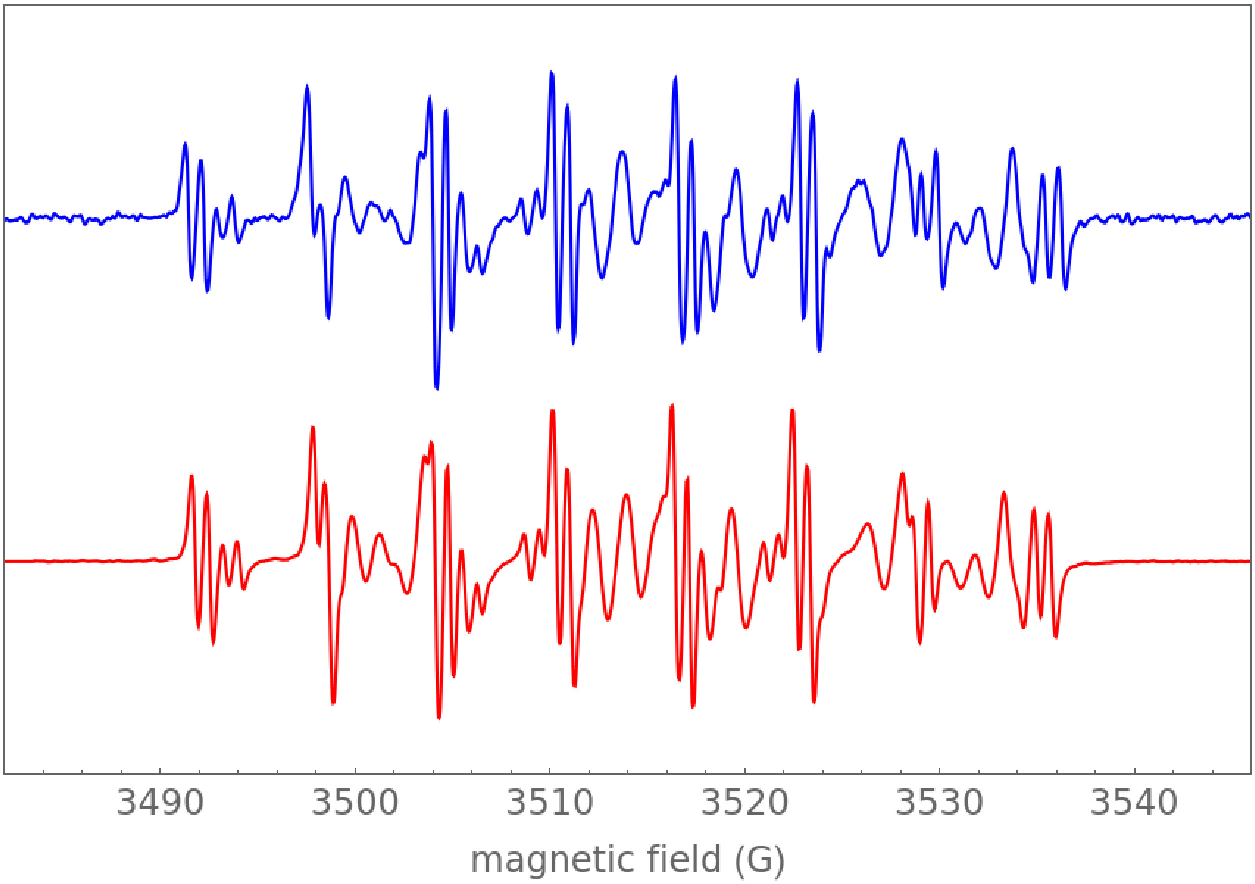

Figure S9. A comparison between an averaged spin abduct spectra of **N-Cl** in acetonitrile (blue) and toluene (red). The red spectrum is shifted and rescaled manually to match the center and amplitude of the blue spectrum.

S7. NMR spectra of **N-Cl** reaction mixture under irradiation in toluene

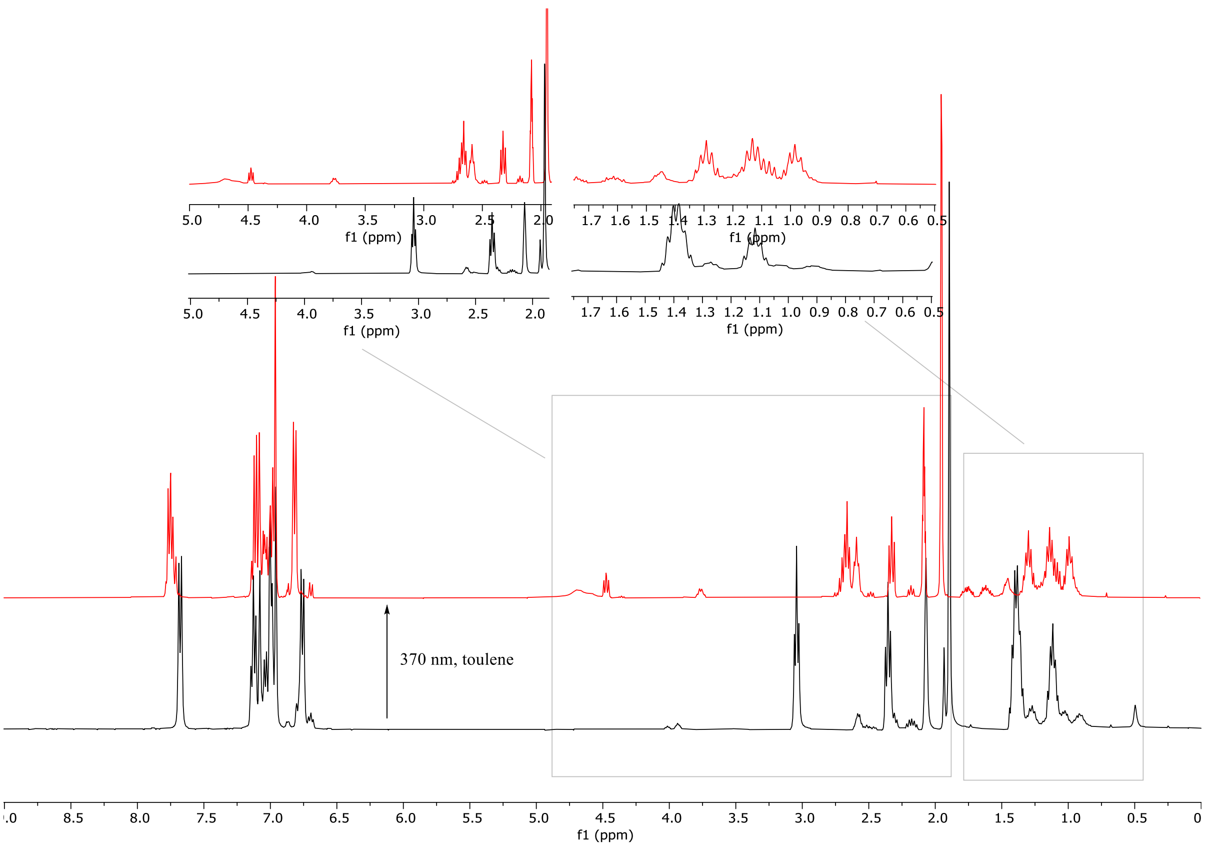

Figure S10. ^{1}H NMR spectra of **N-Cl** reaction mixture under irradiation in C_6D_6.

Before *off-site* irradiation, ^{1}H NMR spectra of **N-Cl** was taken. All signals observed for **N-Cl** are fully compatible with the expected ones.[30]

After *off-site* irradiation of **N-Cl** the ^{1}H NMR spectra of the corresponding reaction mixture was taken. In the upfield region one new triplet and new one quintet were observed, at 4.48 and 3.77 ppm, which correspond to methine groups in **C_6-Cl** and **C_5-Cl** and a new triplet at 2.59 ppm which is common to both products. Additionally, two new triplets at 2.66 ppm and 2.52 ppm are formed which correspond to **N-H**. The ratio of the products is determined from the integral ratios, and it follows that 52 % of **N-H** and 48 % of **C_6-Cl** and **C_5-Cl** mixture (76 % : 24 %) is formed.

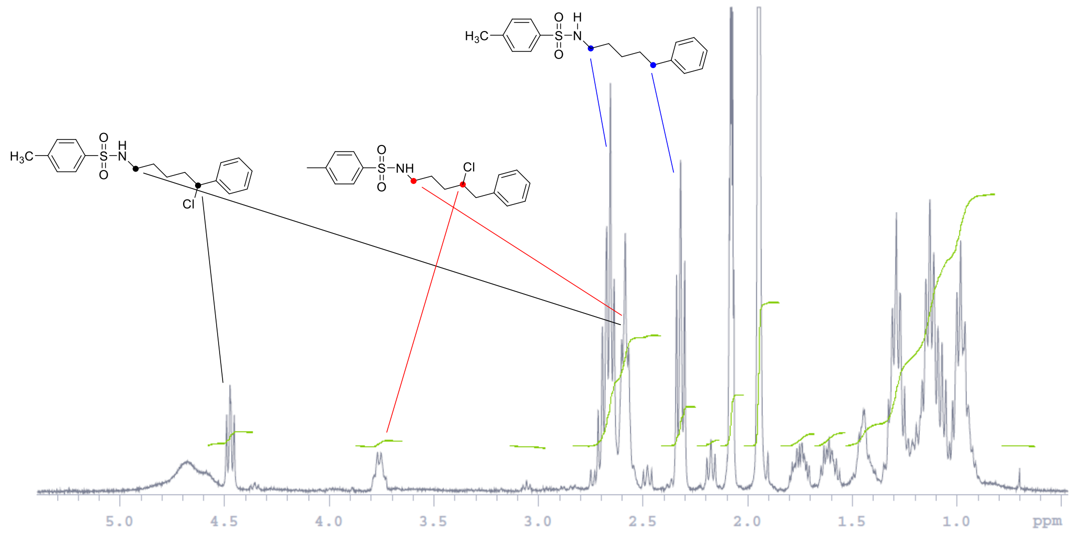

Figure S11. ^{1}H NMR spectra of **N-Cl** reaction mixture under irradiation in C_6D_6

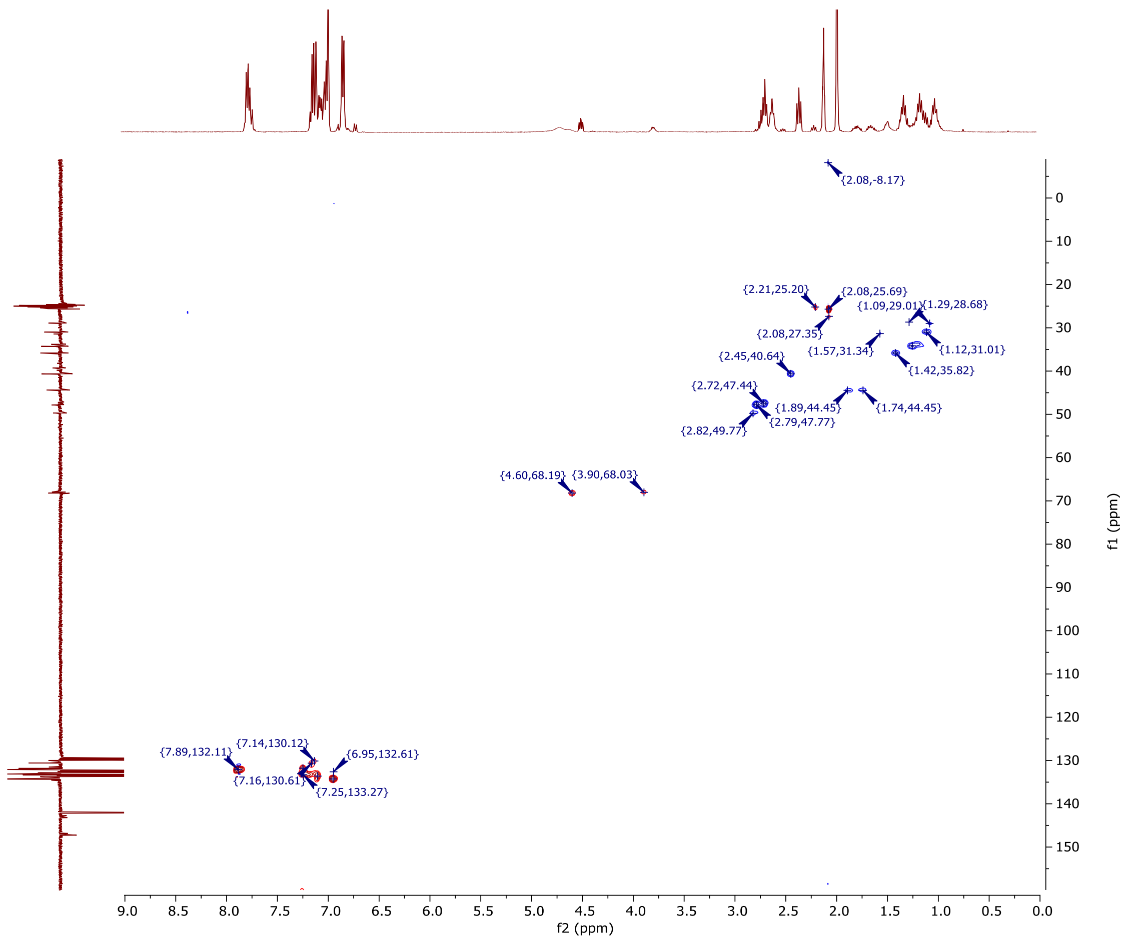

Figure S12. ^{1}H gHSQC NMR spectra of sulfonamide **N-Cl** reaction mixture, C_6D_6, 400 MHz

After *off-site* irradiation of **N-Cl** the ^{1}H gHSQC NMR spectra of the corresponding reaction mixture was taken. From this spectrum it can be seen that the signals of the methyne groups at 68.19 ppm and 68.03 ppm in the $^{13}C\{^{1}H\}$ ATP spectrum correspond to the triplet at 4.60 ppm and quintet at 3.90 ppm in the ^{1}H gHSQC NMR.

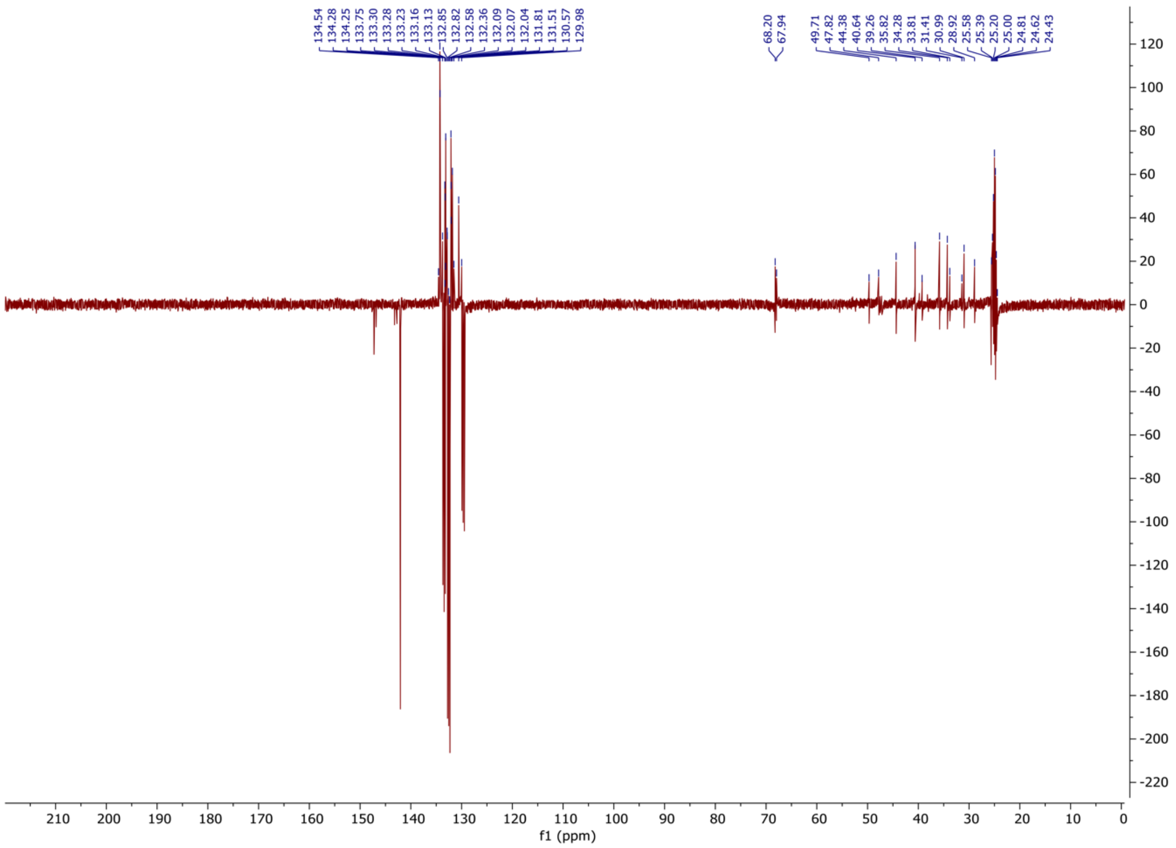

Figure S13. $^{13}C\{^{1}H\}$ APT NMR spectra of **N-Cl** reaction mixture, C_6D_6, 400 MHz

After *off-site* irradiation of **N-Cl** the ^{13}C APT NMR spectra of the corresponding reaction mixture was taken. From this spectrum two signals from the methyne groups at 68.20 ppm and 67.94 ppms can be seen. In total there are 38 signals in the spectrum corresponding to three products **N-H**, **C_5-Cl** and **C_6-Cl**.

$^{13}C\{^{1}H\}$ NMR (101 MHz, toluene) δ 147.3, 147.2, 146.8, 143.2, 142.7, 134.5, 134.3, 134.3, 133.8, 133.3, 133.3, 133.2, 133.2, 133.1, 132.9, 132.8, 132.6, 132.4, 132.1, 132.1, 132.0, 131.8, 131.5, 130.6, 130.0, 68.2, 67.9, 49.7, 47.8, 44.4, 40.6, 39.3, 35.8, 34.3, 33.8, 31.4, 31.0, 28.9.

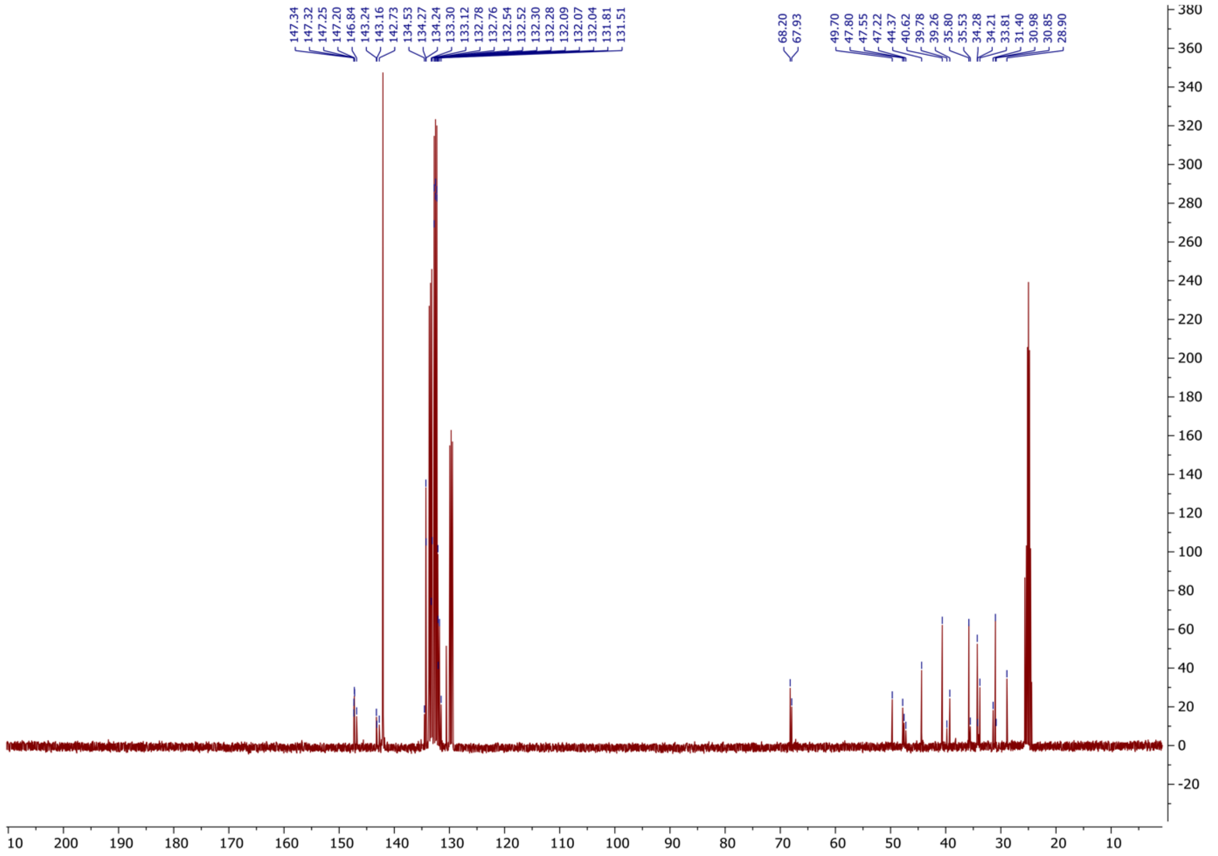

Figure S14. $^{13}C\{^1H\}$ NMR spectra of **N-Cl** reaction mixture in C_6D_6, 400 MHz

After *off-site* irradiation of **N-Cl** the ^{13}C APT NMR spectra of the corresponding reaction mixture was taken. From this spectrum two signals from the methyne groups at 68.20 ppm and 67.94 ppms can be seen. In total there are 38 signals in the spectrum corresponding to three products **N-H**, **C$_5$-Cl** and **C$_6$-Cl**.

$^{13}C\{^1H\}$ NMR (101 MHz, toluene) δ 147.3, 147.3, 147.2, 147.2, 146.8, 143.2, 143.2, 142.7, 134.3, 134.2, 133.3, 133.1, 132.8, 132.7, 132.5, 132.5, 132.3, 132.3, 132.1, 132.1, 132.0, 131.8, 131.5, 68.2, 67.9, 49.7, 47.8, 47.6, 47.2, 44.4, 40.6, 39.8, 39.3, 35.8, 35.5, 34.3, 34.2, 33.8, 31.4, 31.0, 30.9, 28.9.

S8. Laser Flash Photolysis Measurements

The absorption spectra of **N-Cl** and **BnCl** in ACN were recorded before and after purging the solutions with N_2, as well as after 10 and 300 pump-probe shots in a standard quartz cell without using the flow system (Figures S15-S16). Only a small change in the absorption spectrum was observed after purging the solutions with N_2, due to small increase in solution concentration as the solvent was removed. However, after pump-probe measurements, more pronounced changes in the absorption spectrum were evident, showing consumption of the original compound at the absorption maximum along with the formation of photoproducts at longer wavelengths. To avoid parallel excitation of the formed photoproducts, a flow system was used for TAS spectra measurements.

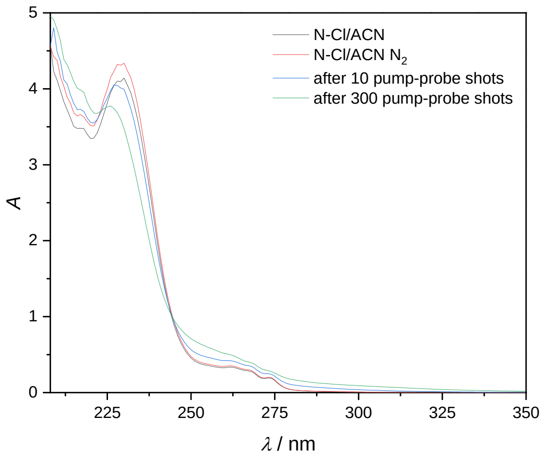

Figure S15. Ground state absorption spectra of 0.3 mM **N-Cl** solution in ACN before and after TAS measurements. E_{266} = 15 mJ, A_{266} = 0.30.

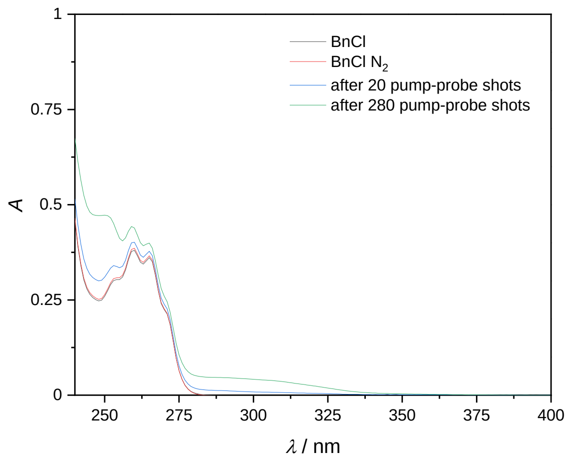

Figure S16. Ground state absorption spectra of 1.6 mM BnCl solution in ACN before and after TAS measurements. $E_{266} = 10$ mJ, $A_{266} = 0.35$.

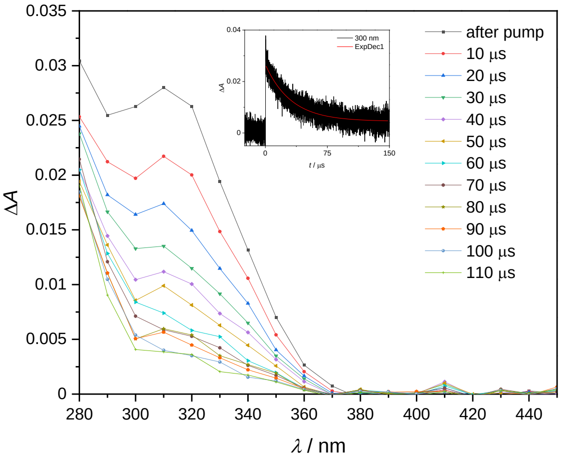

Figure S17. Transient absorption spectra of N_2 purged solution of 1.6 mM BnCl in ACN. Flow rate: 2.4 mL/min. $E_{266} = 10$ mJ. $A_{266} = 0.35$. Insets: Corresponding time profile at 300 nm.

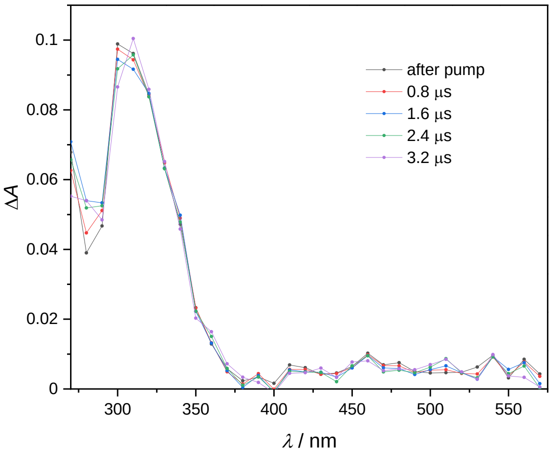

Figure S18. Transient absorption spectra of N_2 purged solution of 1.6 mM BnCl in ACN. Flow rate: 2.4 mL/min. E_{266} = 22 mJ. A_{266} = 0.35.

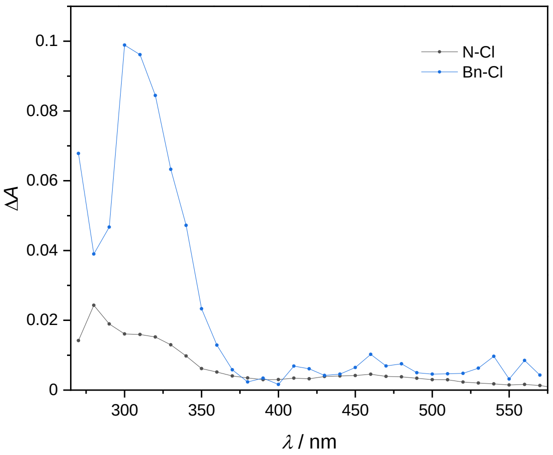

Figure S19. Transient absorption spectra of N_2 purged solutions of 0.3 mM N-Cl and 1.6 mM BnCl in ACN right after pump. Flow rate: 2.4 mL/min. E_{266} = 20-22 mJ. A_{266} = 0.30.

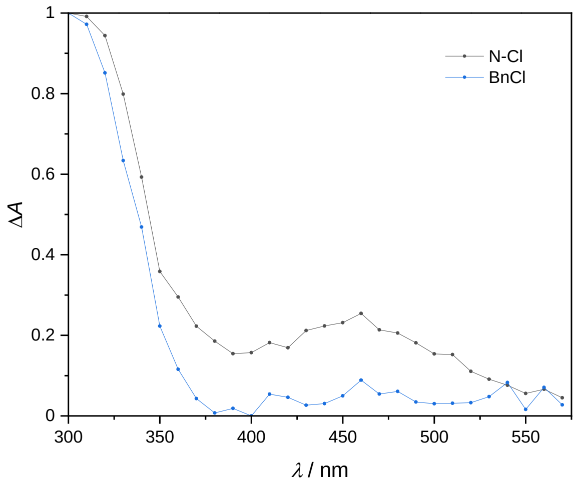

Figure S20. Normalized transient absorption spectra from Figure S19.

S9. UV calculations

On optimized structures single point calculations were performed utilizing time dependent DFT theory with UCAM-B3LYP functional which is a hybrid exchange–correlation functional that combines hybrid qualities of B3LYP and the long-range correction presented by Tawada et al. and TZVP basis set. This was done in order to calculate excitation energies for the 10 lowest lying singlet electronic states. In each case, optimized geometry and inital guess were read from the checkpoint file.

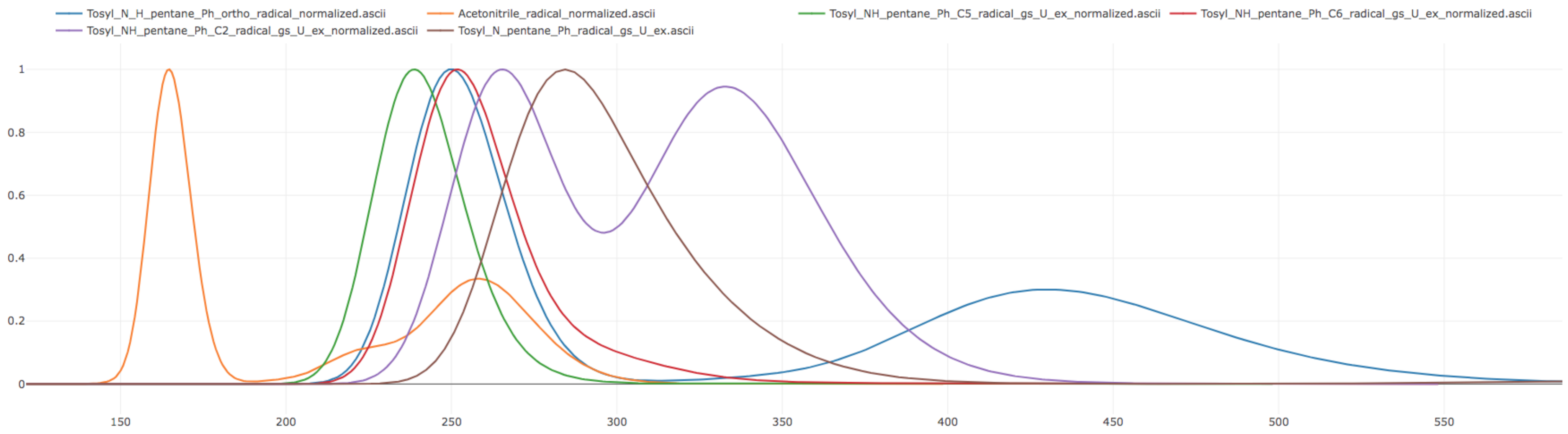

Figure S21. Normalized UV spctra calculated with TD CAM-B3LYP/TZVP/PCM method for the 10 lowest lying singlet states of all the possible radicals present in the system.

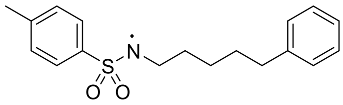

Figure S22. Optimized geometry of Tosyl_N_pentane_Ph_radical_gs_ex.

Table S1. Excitation energies for **N-rad.**

Excited state	Excitation energy (eV)	Excitation energy (nm)	f (oscillator strenght)	S^2
**1**	1.7714eV	699.91	0.0005	0.760
**2**	3.4927eV	354.98	0.0001	2.616
**3**	3.5753eV	346.78	0.0000	2.738
**4**	3.7145eV	333.79	0.0011	0.884
**5**	3.7845eV	327.61	0.0017	0.827
**6**	4.0352eV	307.26	0.0062	0.811
**7**	4.1167eV	301.18	0.0016	0.919
**8**	4.2695eV	290.39	0.0014	0.801
**9**	4.4299eV	279.88	0.0238	0.824
**10**	4.6148eV	268.67	0.0001	2.715

44
-1302.377113
C -0.171838 3.256455 -0.838525
N -0.598499 1.878409 -0.689256
S -1.788661 1.677740 0.531605
O -1.070152 1.719566 1.825208
O -2.914114 2.600382 0.274960
C -2.347499 -0.002123 0.231755
C -1.747803 -1.051860 0.915454
C -3.381908 -0.223406 -0.670238
C -2.191037 -2.347044 0.678872
H -0.956243 -0.856607 1.626120
C -3.809346 -1.524728 -0.894622
H -3.848587 0.610058 -1.177925
C -3.224032 -2.604544 -0.226148
H -4.614226 -1.703767 -1.597934
C 1.161209 3.548823 -0.088254
H 1.212817 4.637863 -0.001079
H 1.081890 3.162144 0.928447
C 2.433917 3.059629 -0.785521
H 3.288946 3.467950 -0.234339
H 2.478694 3.511051 -1.782914
C 2.620498 1.544105 -0.928076
C 2.736545 0.792743 0.409661
H 1.799543 0.887295 0.964790
H -1.726123 -3.169558 1.209495
C -3.720537 -4.008436 -0.451228
H -4.070561 -4.147828 -1.475068
H -2.939860 -4.743842 -0.253285
H -4.559245 -4.232958 0.214502
H 3.531028 1.365460 -1.508278
C 3.071178 -0.670563 0.233243
C 2.115590 -1.570268 -0.250009
C 4.347636 -1.157738 0.521016
C 2.427283 -2.911932 -0.439578
H 1.117742 -1.214778 -0.481021
C 4.664331 -2.500569 0.334292
H 5.102663 -0.477950 0.900785
C 3.704451 -3.383377 -0.147771
H 1.671314 -3.591646 -0.815051
H 5.661017 -2.856035 0.567641
H 3.947666 -4.428828 -0.293720
H -0.934095 3.964857 -0.503703
H 1.797127 1.119590 -1.507795
H 3.510664 1.266232 1.021406
H -0.001799 3.408008 -1.909249

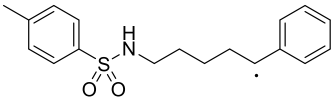

Figure S23. Optimized geometry of Tosyl_NH_pentane_Ph_C6_radical_gs_ex

Table S2. Excitation energies for **C_6-rad**.

Excited state	Excitation energy (eV)	Excitation energy (nm)	f (oscillator strenght)	S^2
**1**	**3.4783eV**	**356.45**	**0.0010**	**993**
**2**	3.4938eV	354.87	0.0000	2.803
**3**	3.5256eV	351.67	0.0002	1.064
**4**	4.1977eV	295.36	0.0362	1.040
**5**	4.4707eV	277.33	0.0070	2.459
**6**	4.6389eV	267.27	0.0000	2.742
**7**	4.6473eV	266.79	0.0000	2.802
**8**	4.7210eV	262.62	0.0018	0.906
**9**	4.9276eV	251.61	0.4632	0.803
**10**	4.9796eV	248.98	0.0001	2.799

44
-1302.394794
C 0.965641 -1.970828 -1.088448
N -0.359736 -2.517329 -0.766692
S -1.118881 -2.085051 0.687592
O -2.021755 -3.213435 0.983616
O -0.119296 -1.620509 1.670009
C -2.115539 -0.652338 0.231744
C -3.354418 -0.852063 -0.369436
C -1.643207 0.625314 0.496740
C -4.119961 0.250910 -0.715621
H -3.715750 -1.856517 -0.545369
C -2.423268 1.720006 0.136987
H -0.688928 0.763815 0.986754
C -3.665888 1.553157 -0.474709
H -2.054992 2.717996 0.342704
C 2.133438 -2.740361 -0.465433
H 2.012311 -2.744599 0.620307
H 2.068141 -3.784326 -0.793646
C 3.525256 -2.204558 -0.826863
H 3.631237 -2.134576 -1.915704
H 4.261716 -2.943586 -0.499818
C 3.897757 -0.849048 -0.186504
C 3.313192 0.348832 -0.858430
H 3.416502 0.382312 -1.939986
H -5.089746 0.099323 -1.175858
C -4.499569 2.740965 -0.879235
H -5.548372 2.600167 -0.610315
H -4.146663 3.655819 -0.402523
H -4.459893 2.894420 -1.961623
H -0.458486 -3.520094 -0.882428
H 4.993584 -0.759345 -0.226192
C 2.720667 1.474748 -0.247490
C 2.295704 2.569799 -1.053953
C 2.507090 1.591068 1.154548
C 1.714306 3.692772 -0.500283
H 2.443195 2.511697 -2.126449
C 1.921344 2.720573 1.696965
H 2.798410 0.783011 1.811581
C 1.521323 3.781980 0.881733
H 1.408407 4.510419 -1.142296
H 1.772258 2.779414 2.768637
H 1.069898 4.665164 1.316030
H 1.051662 -1.969188 -2.179113
H 0.978594 -0.927176 -0.777042
H 3.642357 -0.876042 0.874843

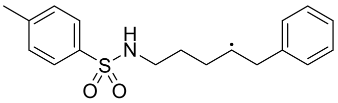

Figure S24. Optimized geometry of Tosyl_NH_pentane_Ph_C5_radical_gs_ex

Table S3. Excitation energies for **C_5-rad** of compound **N-Cl**.

Excited state	Excitation energy (eV)	Excitation energy (nm)	f (oscillator strenght)	S^2
**1**	3.4827eV	356.00	0.0000	2.754
**2**	3.5714eV	347.16	0.0000	2.720
**3**	4.6442eV	266.96	0.0000	2.754
**4**	4.6580eV	266.17	0.0000	2.754
**5**	4.6604eV	266.04	0.0004	2.416
**6**	4.7317eV	262.03	0.0005	2.531
**7**	4.9547eV	250.24	0.0013	1.283
**8**	4.9587eV	250.03	0.0000	2.754
**9**	5.1593eV	240.31	0.0047	2.080
**10**	5.2085eV	238.04	0.0290	0.890

44
-1302.379208
C 0.167984 -1.886389 -1.555030
N 1.096986 -2.056233 -0.433818
S 1.287614 -0.949260 0.811100
O 0.121445 -0.045578 0.827648
O 1.670672 -1.756719 1.984204
C 2.715769 0.053583 0.344813
C 3.993733 -0.395414 0.658466
C 2.523602 1.256902 -0.321256
C 5.089033 0.370863 0.284854
H 4.124636 -1.323811 1.198289
C 3.632430 2.010260 -0.689674
H 1.521911 1.604070 -0.535724
C 4.928566 1.582715 -0.394538
H 3.484462 2.949602 -1.209763
C -0.938444 -2.945157 -1.574499
H -1.451247 -2.873430 -2.539091
H -0.480644 -3.940528 -1.549593
C -1.967591 -2.817245 -0.440818
H -1.457860 -2.818795 0.528686
H -2.584099 -3.732024 -0.443597
C -2.836636 -1.611639 -0.552796
C -3.443782 -0.958254 0.645908
H -2.696708 -0.921029 1.446411
H 6.086197 0.023661 0.530045
C 6.123716 2.421693 -0.764438
H 6.419854 3.064203 0.070161
H 6.983849 1.799812 -1.017508
H 5.908384 3.068817 -1.615571
C -3.965548 0.436037 0.373620
C -3.090510 1.526027 0.370384
C -5.314081 0.661507 0.095239
C -3.556226 2.807556 0.096298
H -2.041819 1.360779 0.589355
C -5.782357 1.943033 -0.183143
H -6.006761 -0.173322 0.100993
C -4.903698 3.020919 -0.182936
H -2.866375 3.643377 0.105616
H -6.833853 2.098914 -0.393826
H -5.265983 4.019900 -0.393924
H -3.199155 -1.323632 -1.535126
H -4.268100 -1.575910 1.040072
H -0.274156 -0.892677 -1.475170
H 1.289344 -2.993952 -0.109300
H 0.734712 -1.925815 -2.489846

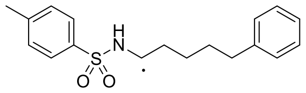

Figure S25. Optimized geometry of Tosyl_N_pentane_Ph_C2_radical_gs_ex.

Table S4. Excitation energies for **C_2-rad**.

Excited state	Excitation energy (eV)	Excitation energy (nm)	f (oscillator strenght)	S^2
1	3.4936eV	354.89	0.0005	2.706
2	3.5785eV	346.47	0.0000	2.755
3	3.7253eV	332.82	0.0210	877
4	4.5980eV	269.65	0.0003	2.709
5	4.6421eV	267.09	0.0000	2.614
6	4.6521eV	266.51	0.0187	0.949
7	4.7209eV	262.63	0.0000	2.754
8	4.7662eV	260.13	0.0000	2.754
9	4.7921eV	258.73	0.0039	0.917
10	5.0075eV	247.60	0.0002	2.713

```
 44
 -1302.382426
C -0.187846 -2.118820 -0.560291
N  0.816902 -1.388795  0.069923
S  2.489861 -1.598831 -0.441590
O  3.186737 -2.521894  0.478567
O  2.422430 -1.869230 -1.886510
C  3.154968  0.053436 -0.172071
C  2.815191  1.077843 -1.050116
C  4.005896  0.276732  0.901111
C  3.336182  2.344739 -0.836681
H  2.160390  0.882212 -1.888815
C  4.519289  1.554530  1.098492
H  4.268050 -0.539073  1.561391
C  4.194940  2.604400  0.238275
H  5.184891  1.733800  1.934642
C -1.049543 -3.069309  0.204543
H -1.729651 -3.553523 -0.501311
H -0.418763 -3.872952  0.617497
C -1.881092 -2.484982  1.369487
H -1.231797 -1.927981  2.056396
H -2.259702 -3.330841  1.950619
C -3.073658 -1.607185  0.967099
C -2.713642 -0.206359  0.438525
H -2.082750 -0.300996 -0.447669
H  3.073829  3.146810 -1.516875
C  4.775027  3.979695  0.438928
H  5.610482  4.151450 -0.246048
H  4.032523  4.756125  0.246108
H  5.149307  4.110352  1.454625
H -3.726392 -1.487969  1.837504
C -3.932051  0.623998  0.109836
C -4.494738  0.596263 -1.168798
C -4.547156  1.415385  1.083379
C -5.637207  1.332293 -1.466220
H -4.028119 -0.004431 -1.942101
C -5.689290  2.154127  0.791670
H -4.122337  1.458372  2.080687
C -6.239660  2.114164 -0.485614
H -6.054261  1.298933 -2.465757
H -6.147137  2.764659  1.560961
H -7.127262  2.690695 -0.715912
H -3.670524 -2.133322  0.214231
H -2.112713  0.311834  1.192739
H -0.124020 -2.147237 -1.637399
H  0.792872 -1.420759  1.084663
```

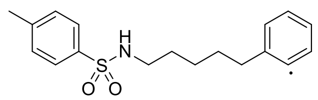

Figure S26. Optimized geometry of Tosyl_NH_pentane_Ph_ortho_radical_gs_ex.

Table S5. Excitation energies for ortho radical.

Excited state	Excitation energy (eV)	Excitation energy (nm)	f (oscillator strenght)	S^2
1	2.8813eV	430.30	0.0017	760
2	3.5192eV	352.31	0.0000	2.756
3	3.5769eV	346.62	0.0001	0.784
4	3.6468eV	339.98	0.0000	2.749
5	4.5978eV	269.66	0.0000	2.749
6	4.6540eV	266.40	0.0000	2.751
7	4.7661eV	260.14	0.0005	2.696
8	4.8350eV	256.43	0.0001	2.720
9	4.9779eV	249.07	0.0052	0.811
10	5.0250eV	246.73	0.0000	2.746

44
-1302.348507
C 0.298275 -3.351995 -0.876457
N 0.810560 -1.956086 -0.800309
S 1.664497 -1.583019 0.645616
O 0.657993 -1.508696 1.720415
O 2.832782 -2.474752 0.814441
C 2.270704 0.067618 0.252103
C 1.381553 1.136894 0.213212
C 3.628513 0.248785 0.021776
C 1.870499 2.404061 -0.068881
H 0.326818 0.984845 0.399886
C 4.099067 1.526941 -0.257296
H 4.300801 -0.597338 0.072375
C 3.232526 2.620874 -0.305539
H 5.157695 1.674079 -0.436434
C -1.068390 -3.564472 -0.221305
H -1.189069 -4.647979 -0.118971
H -1.048286 -3.168712 0.795036
C -2.280147 -3.031540 -1.000610
H -3.181388 -3.471547 -0.558779
H -2.233813 -3.419537 -2.025106
C -2.470070 -1.510108 -1.063891
C -2.708465 -0.856823 0.306245
H -1.810767 -0.959305 0.922274
H 1.181059 3.239607 -0.100964
C 3.752595 4.008500 -0.574414
H 4.709589 3.981852 -1.096549
H 3.050188 4.587056 -1.176680
H 3.903326 4.552755 0.362581
H -3.331357 -1.301205 -1.706423
C -3.080040 0.605005 0.214723
C -2.342441 1.536579 -0.484269
C -4.222128 1.126227 0.844912
C -2.612563 2.872442 -0.622847
C -4.552795 2.476496 0.751487
H -4.856451 0.455217 1.415013
C -3.758079 3.355417 0.022932
H -1.980049 3.533576 -1.204034
H -5.441381 2.842903 1.250996
H -4.020676 4.404215 -0.051648
H 1.026825 -4.048880 -0.449039
H -1.606303 -1.038931 -1.536955
H -3.509043 -1.386743 0.831234
H 0.230057 -3.578985 -1.943345
H 1.458469 -1.773869 -1.562264

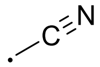

Figure S27. Optimized geometry of Acetonitrile_radical_gs_ex.

Table S6. Excitation energies for acetonitrile radical.

Excited state	Excitation energy (eV)	Excitation energy (nm)	f (oscillator strenght)	S^2
1	4.0252eV	308.02	0.0000	0.841
2	4.7938eV	258.63	0.0289	0.883
3	5.5398eV	223.81	0.0090	0.918
4	5.7387eV	216.05	0.0000	0.856
5	6.1902eV	200.29	0.0010	2.710
6	7.3006eV	169.83	0.0000	0.936
7	7.3939eV	167.68	0.0037	0.781
8	7.5368eV	164.51	0.0839	0.799
9	7.8371eV	158.20	0.0000	2.077
10	8.2319eV	150.61	0.0006	2.745

```
 5
 -132.107965
C -0.187124 -0.000250  0.000670
N -1.354224 -0.000011 -0.000330
C  1.189341 -0.000070 -0.000181
H  1.734930 -0.932934 -0.000314
H  1.731340  0.934931 -0.000312
```

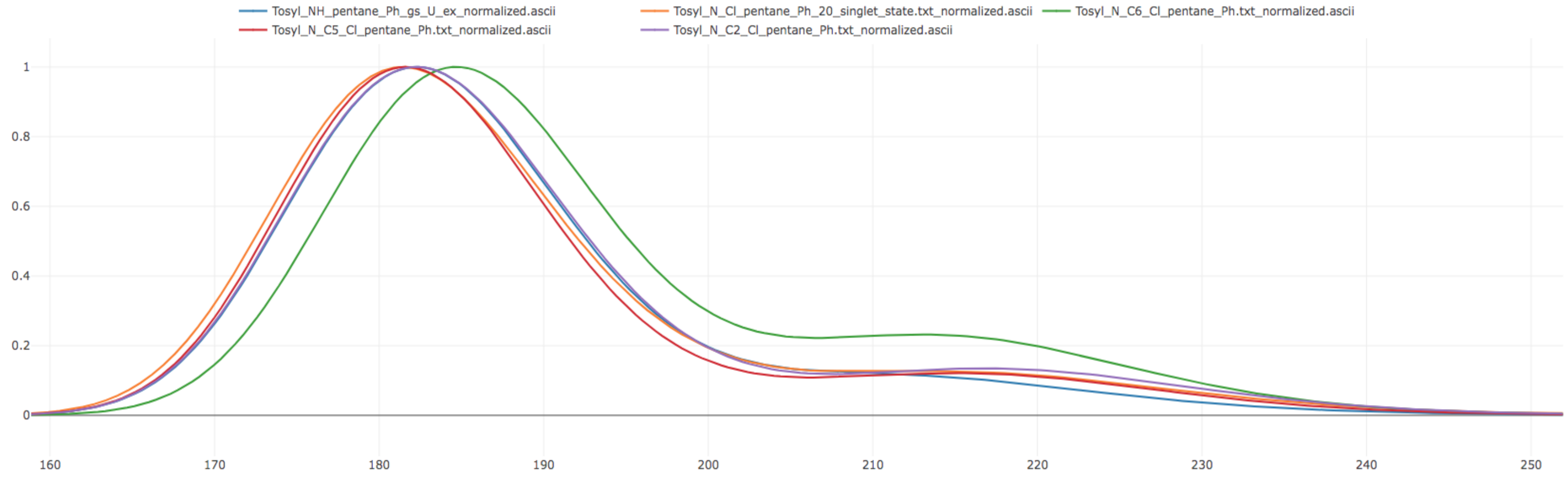

Figure S28. Normalized UV spctra calculated with TD CAM-B3LYP/TZVP/PCM method for the 10 lowest lying singlet states of all the possible ground state products and reactant.

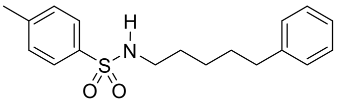

Figure S29. Optimized geometry of Tosyl_Nh_pentane_Ph_gs_ex.

Table S7. Excitation energies for **N-H**.

Excited state	Excitation energy (eV)	Excitation energy (nm)	f (oscillator strenght)	S^2
1	5.3528eV	231.63	0.0096	0
2	5.4328eV	228.21	0.0037	0
3	5.8207eV	213.01	0.2239	0
4	6.0711eV	204.22	0.0431	0
5	6.3567eV	195.04	0.0075	0
6	6.5543eV	189.16	0.0077	0
7	6.6647eV	186.03	0.7850	0
8	6.7462eV	183.78	0.2750	0
9	6.8205eV	181.78	0.0889	0
10	6.8892eV	179.97	1.3530	0

45
-1303.024311
C -0.235146 3.477076 -0.665703
N -0.873843 2.134449 -0.727299
S -1.454022 1.526425 0.770246
O -0.265468 1.208331 1.583434
O -2.496926 2.410192 1.334509
C -2.245733 0.005629 0.213555
C -1.461568 -1.075438 -0.177212
C -3.632617 -0.067992 0.203427
C -2.087282 -2.241534 -0.593030
H -0.382140 -1.010686 -0.150104
C -4.241006 -1.246243 -0.215094
H -4.219635 0.781049 0.527621
C -3.482622 -2.347101 -0.618323
H -5.322842 -1.309052 -0.224444
C 1.250848 3.467269 -0.303001
H 1.538849 4.520585 -0.222491
H 1.379414 3.043574 0.693949
C 2.180088 2.777049 -1.316785
H 3.174566 3.229999 -1.239748
H 1.827921 3.004289 -2.329387
C 2.330200 1.255366 -1.169510
C 3.132470 0.834860 0.075657
H 2.667092 1.255712 0.968830
H -1.480335 -3.085458 -0.899575
C -4.146034 -3.631276 -1.041293
H -5.175285 -3.462754 -1.359590
H -3.606867 -4.105499 -1.863016
H -4.169715 -4.345145 -0.212630
H 2.834694 0.863208 -2.058358
C 3.230828 -0.663736 0.238575
C 4.171467 -1.406913 -0.480144
C 2.363627 -1.346411 1.096329
C 4.244178 -2.789925 -0.350331
H 4.861367 -0.895084 -1.142854
C 2.433733 -2.730521 1.229598
H 1.633079 -0.782911 1.666131
C 3.373251 -3.457973 0.505597
H 4.985359 -3.345860 -0.912505
H 1.758269 -3.239457 1.907414
H 3.432693 -4.534350 0.613234
H -0.780308 4.133869 0.020807
H 1.343645 0.791317 -1.141179
H 4.138568 1.262614 0.008823
H -0.355125 3.894189 -1.668526
H -1.674952 2.150608 -1.352659

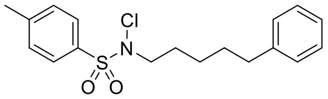

Figure S30. Optimized geometry of Tosyl_NCl_pentane_Ph_gs_ex.

Table S8. Excitation energies for **N-Cl**.

Excited state	Excitation energy (eV)	Excitation energy (nm)	f (oscillator strenght)	S^2
**1**	4.3890eV	282.49	0.0062	0
**2**	5.2998eV	233.94	0.0166	0
**3**	5.4372eV	228.03	0.0034	0
**4**	5.6952eV	217.70	0.1885	0
**5**	5.7586eV	215.30	0.0797	0
**6**	6.0642eV	204.45	0.0398	0
**7**	6.0687eV	204.30	0.0167	0
**8**	6.0794eV	203.94	0.0154	0
**9**	6.2570eV	198.15	0.0033	0
**10**	6.3566eV	195.05	0.0151	0

45

C -0.646703 -3.160457 0.340745
N -0.865686 -1.678889 0.464143
S -2.211723 -1.154125 -0.581739
O -1.737194 -1.533996 -1.923521
O -3.503415 -1.650045 -0.079894
C -2.164052 0.631897 -0.410665
C -1.192526 1.353148 -1.095540
C -3.128027 1.265282 0.363469
C -1.185037 2.736069 -0.985474
H -0.461227 0.843376 -1.707445
C -3.106334 2.650647 0.456155
H -3.879108 0.681191 0.877217
C -2.139077 3.405784 -0.212355
H -3.853921 3.151883 1.059508
C 0.446168 -3.499086 -0.679715
H 0.239200 -4.517019 -1.025345
H 0.335199 -2.862309 -1.556613
C 1.880378 -3.467338 -0.133348
H 2.545066 -3.860471 -0.910609
H 1.948473 -4.173673 0.701965
C 2.422744 -2.111337 0.336134
C 2.541051 -1.058565 -0.778771
H 1.545140 -0.841225 -1.172149
H -0.425867 3.303452 -1.510449
C -2.141425 4.909216 -0.130148
H -2.602691 5.257310 0.794732
H -1.128935 5.312023 -0.181551
H -2.706982 5.339769 -0.961901
H 3.414367 -2.271162 0.770843
C 3.190806 0.223811 -0.312920
C 2.499766 1.117235 0.512161
C 4.504116 0.537405 -0.668933
C 3.103224 2.284782 0.966119
H 1.478922 0.894639 0.801412
C 5.112379 1.705633 -0.217794
H 5.056836 -0.140084 -1.310767
C 4.413525 2.584150 0.602702
H 2.549434 2.962745 1.604959
H 6.131936 1.928840 -0.509119
H 4.884144 3.493799 0.955545
H -1.587270 -3.660039 0.094647
H 1.800197 -1.712034 1.140800
H 3.121704 -1.476417 -1.606850
H -0.351880 -3.507130 1.329523
Cl -1.243903 -1.241563 2.158639

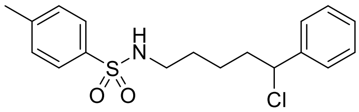

Figure S31. Optimized geometry of Tosyl_NH_pentane_Ph_C6Cl_gs_ex.

Table S9. Excitation energies for **C_6-Cl**.

Excited state	Excitation energy (eV)	Excitation energy (nm)	f (oscillator strenght)	S^2
1	5.3448eV	231.97	0.0043	0
2	5.3843eV	230.27	0.0042	0
3	5.7440eV	215.85	0.2724	0
4	5.8562eV	211.71	0.1540	0
5	6.5230eV	190.07	0.0947	0
6	6.6074eV	187.65	0.1785	0
7	6.6479eV	186.50	0.4603	0
8	6.6876eV	185.40	0.0407	0
9	6.7439eV	183.85	0.8958	0
10	6.8541eV	180.89	0.2959	0

```
45
-1762.654158
C  0.421932 -0.443330 -0.145444
N  1.464197 -0.954196  0.756322
S  2.895043 -1.599932  0.123744
O  3.381590 -2.519975  1.168464
O  2.697678 -2.055692 -1.267267
C  3.994588 -0.169050  0.049307
C  4.427080  0.422669  1.232558
C  4.422790  0.299843 -1.183670
C  5.292679  1.503071  1.168565
H  4.092446  0.038846  2.187206
C  5.291795  1.386540 -1.229330
H  4.084415 -0.184737 -2.089531
C  5.739620  2.002808 -0.061221
H  5.626598  1.755907 -2.191626
C -0.507450 -1.517830 -0.713539
H  0.100345 -2.244432 -1.255990
H -0.981092 -2.049461  0.115254
C -1.572052 -0.969474 -1.673674
H -2.112089 -1.817744 -2.107092
H -1.062154 -0.487068 -2.514534
C -2.606730  0.033056 -1.134472
C -3.632972 -0.514280 -0.138883
Cl -2.899092 -0.625815  1.573472
H  5.630365  1.967095  2.088258
C  6.700969  3.161395 -0.111730
H  6.748813  3.596252 -1.110436
H  6.412526  3.947794  0.588599
H  7.711127  2.840574  0.158778
H -3.185577  0.391227 -1.991706
C -4.910221  0.278608 -0.064672
C -4.907218  1.650001  0.205809
C -6.129204 -0.357166 -0.299824
C -6.096394  2.365743  0.234080
H -3.972492  2.156165  0.413227
C -7.322413  0.359509 -0.274903
H -6.147098 -1.422177 -0.502034
C -7.308560  1.723127 -0.008820
H -6.079729  3.427307  0.448862
H -8.260171 -0.149988 -0.459758
H -8.235258  2.283181  0.014740
H  0.918882  0.095299 -0.955303
H -2.124923  0.917370 -0.711896
H -3.866276 -1.550632 -0.375332
H -0.142592  0.293864  0.428019
H  1.134838 -1.573604  1.488713
```

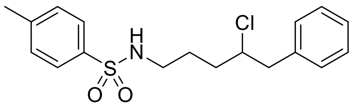

Figure S32. Optimized geometry of Tosyl_NH_pentane_Ph_C5Cl_gs_ex.

Table S10. Excitation energies for **C_5-Cl**.

Excited state	Excitation energy (eV)	Excitation energy (nm)	f (oscillator strenght)	S^2
1	5.3389eV	232.23	0.0054	0
2	5.4531eV	227.37	0.0011	0
3	5.7313eV	216.33	0.3133	0
4	6.0921eV	203.52	0.0297	0
5	6.4966eV	190.84	0.0901	0
6	6.6396eV	186.74	0.3367	0
7	6.8205eV	181.78	1.2281	0
8	6.8804eV	180.20	0.7480	0
9	6.8936eV	179.86	0.5004	0
10	7.0461eV	175.96	0.0146	0

45
-1762.656041
C 0.629190 -3.050658 0.327336
N 1.291254 -1.830994 -0.168226
S 1.542554 -0.555909 0.931723
O 0.454308 0.442173 0.821787
O 1.837683 -1.194647 2.226368
C 3.038057 0.213359 0.287146
C 2.943282 1.406946 -0.414535
C 4.268816 -0.393058 0.518227
C 4.105858 1.995233 -0.901855
H 1.977743 1.870284 -0.566498
C 5.416451 0.209701 0.026858
H 4.324124 -1.316124 1.079754
C 5.355200 1.410534 -0.690565
H 6.377934 -0.258216 0.204143
C -0.873604 -2.960139 0.618528
H -1.171192 -3.930357 1.025969
H -1.039942 -2.238339 1.421566
C -1.749057 -2.635837 -0.614727
H -2.616974 -3.298933 -0.645273
H -1.185673 -2.845826 -1.527260
C -2.254986 -1.192469 -0.672276
C -3.378673 -0.903011 0.323277
H -3.018743 -1.222599 1.307097
H 4.036456 2.926229 -1.451984
C 6.614018 2.066878 -1.193238
H 7.321756 1.329056 -1.575259
H 6.401033 2.779840 -1.990175
H 7.115027 2.611608 -0.387632
Cl -2.854927 -0.861623 -2.389914
C -3.815243 0.541785 0.408652
C -2.956703 1.515306 0.927928
C -5.085569 0.929434 -0.017106
C -3.362332 2.842294 1.015271
H -1.962119 1.237497 1.258514
C -5.494476 2.256795 0.071636
H -5.759872 0.186513 -0.427438
C -4.633433 3.217857 0.589278
H -2.684648 3.583826 1.421251
H -6.485113 2.538497 -0.264854
H -4.949293 4.251679 0.660428
H 1.162347 -3.354539 1.227225
H -1.443793 -0.481693 -0.534933
H -4.232902 -1.539156 0.079606
H 0.815750 -3.813799 -0.432777
H 0.895499 -1.462234 -1.027157

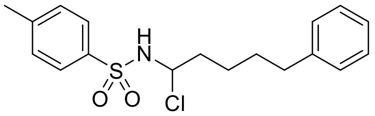

Figure S33. Optimized geometry of Tosyl_NH_pentane_Ph_C2Cl_gs_ex.

Table S11. Excitation energies for **C_2-Cl.**

Excited state	Excitation energy (eV)	Excitation energy (nm)	f (oscillator strenght)	S^2
1	5.3102eV	233.48	0.0071	0
2	5.4447eV	227.71	0.0029	0
3	5.6905eV	217.88	0.3193	0
4	6.0903eV	203.58	0.0330	0
5	6.6007eV	187.84	0.3801	0
6	6.6340eV	186.89	0.4770	0
7	6.8305eV	181.51	0.0492	0
8	6.8535eV	180.91	0.6153	0
9	6.8762eV	180.31	0.9334	0
10	6.9016eV	179.65	0.3032	0

45
-1762.659692
C -0.390671 2.200217 0.378305
N -0.151317 0.831898 0.634931
S -1.180649 -0.142541 1.582793
O -0.356675 -1.325741 1.873457
O -1.703198 0.749742 2.628103
C -2.563310 -0.679904 0.560494
C -2.421156 -1.815345 -0.229538
C -3.751964 0.039015 0.581843
C -3.487418 -2.222023 -1.019897
H -1.498455 -2.379996 -0.212475
C -4.807884 -0.385708 -0.213001
H -3.848854 0.910741 1.213986
C -4.694262 -1.516565 -1.025972
H -5.736053 0.173492 -0.201078
C 0.876076 3.038571 0.210870
H 0.550574 4.080204 0.176949
H 1.436801 2.917611 1.142321
C 1.782886 2.789681 -1.003744
H 2.535907 3.584413 -0.993990
H 1.196929 2.940578 -1.913159
C 2.514692 1.442251 -1.103955
C 3.396589 1.085480 0.106053
H 2.790959 1.066681 1.014565
H -3.380915 -3.105716 -1.638112
C -5.856269 -1.984493 -1.861725
H -6.478640 -1.147777 -2.181725
H -5.518169 -2.518666 -2.750568
H -6.493025 -2.667106 -1.291004
H 3.144089 1.470721 -1.998287
C 4.088814 -0.249208 -0.047406
C 3.529562 -1.409230 0.495294
C 5.284752 -0.359996 -0.761488
C 4.146238 -2.645282 0.324782
H 2.609054 -1.346637 1.066294
C 5.904055 -1.593381 -0.933416
H 5.740466 0.530608 -1.181323
C 5.334944 -2.741881 -0.391179
H 3.699183 -3.531625 0.758748
H 6.834106 -1.657056 -1.485721
H 5.817811 -3.702855 -0.520380
H -0.992403 2.593386 1.191446
H 1.809571 0.626222 -1.295597
H 4.142190 1.875553 0.242000
Cl -1.507073 2.476597 -1.134063
H 0.373662 0.297253 -0.043415

S10. DFT results and optimized geometries

Showing only the top 10 structures obtained after ensemble sorting using CREST. For all calculated structures and energies, please consult Zenodo repository. Energies in the title of .xyz correspond to enthalpies calculated at RO-B2PLYP-D3/G3MP2-large//B3LYP/6-31G(d), using enthalpies and single point energies of B3LYP/6-31G(d) calculations, and single point energies at RO-B2PLYP-D3/G3MP2-large level of theory.

TS-1,5-HAT$_{uni}$

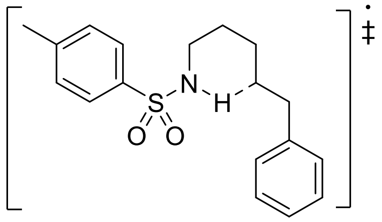

Name	E(B3LYP)	G(B3LYP)	E(RO-B2PLYP-D3)	G(RO-B2PLYP-D3)	NImag
Tosyl_NH_pentane_Ph_1_5_HAT_TS_0000_cregen_B2PLYP	-1302.419625	-1302.036339	-1301.941582	-1301.63464568	-1722.0377
Tosyl_NH_pentane_Ph_1_5_HAT_TS_0003_cregen_B2PLYP	-1302.419605	-1302.036320	-1301.940967	-1301.63444209	-1721.7907
Tosyl_NH_pentane_Ph_1_5_HAT_TS_0009_cregen_B2PLYP	-1302.419605	-1302.036320	-1301.940819	-1301.63428458	-1721.7940
Tosyl_NH_pentane_Ph_1_5_HAT_TS_0008_cregen_B2PLYP	-1302.419429	-1302.035976	-1301.940622	-1301.63403108	-1596.8746
Tosyl_NH_pentane_Ph_1_5_HAT_TS_0005_cregen_B2PLYP	-1302.417903	-1302.034532	-1301.941759	-1301.63396097	-1749.5326
Tosyl_NH_pentane_Ph_1_5_HAT_TS_0007_cregen_B2PLYP	-1302.417914	-1302.034544	-1301.941798	-1301.63387317	-1749.4575
Tosyl_NH_pentane_Ph_1_5_HAT_TS_0001_cregen_B2PLYP	-1302.419625	-1302.036340	-1301.940742	-1301.63380726	-1721.9304
Tosyl_NH_pentane_Ph_1_5_HAT_TS_0010_cregen_B2PLYP	-1302.419625	-1302.036339	-1301.940728	-1301.63379032	-1722.0593
Tosyl_NH_pentane_Ph_1_5_HAT_TS_0006_cregen_B2PLYP	-1302.416926	-1302.033536	-1301.940679	-1301.63340529	-1719.5650
Tosyl_NH_pentane_Ph_1_5_HAT_TS_0002_cregen_B2PLYP	-1302.419625	-1302.036340	-1301.940158	-1301.63323368	-1721.6654

44
-1301.63464568
C 0.774617 2.351529 -1.434351
C 2.031886 1.739046 -0.785885
C 1.919631 0.225668 -0.716614
C 2.606594 -0.524601 0.415678
C -0.485237 1.991620 -0.629206
N -0.622562 0.527044 -0.581110
H 0.668212 1.983868 -2.463020
H 0.861335 3.442860 -1.486632
H 2.150700 2.149261 0.226183
H 2.924885 2.035660 -1.354672
H 2.038420 -0.259047 -1.692817
H 0.618829 0.106290 -0.576067
H 2.211506 -1.545606 0.459324
H 2.357262 -0.049377 1.371773
H -1.373487 2.401643 -1.130427
H -0.430307 2.431348 0.375141
S -1.310714 -0.129846 0.826948
O -1.230989 0.801392 1.966044
O -0.751336 -1.483421 0.937296
C -3.034759 -0.260605 0.353009
C -3.959937 0.651471 0.858602
C -3.430414 -1.279573 -0.518422
C -5.298382 0.539983 0.480580
H -3.632920 1.423834 1.546550
C -4.768816 -1.374617 -0.884175
H -2.697432 -1.986899 -0.891736
C -5.723464 -0.469208 -0.392190
H -6.024181 1.246855 0.875135
H -5.081997 -2.166349 -1.560737
C -7.176972 -0.602322 -0.779524
H -7.288522 -0.810928 -1.849465
H -7.654580 -1.429549 -0.238302
H -7.738615 0.308540 -0.550462
C 4.115799 -0.554700 0.229197
C 4.708677 -1.497095 -0.623542
C 4.943530 0.373225 0.875169
C 6.088725 -1.512935 -0.824742
H 4.081669 -2.230394 -1.126614
C 6.325284 0.360719 0.676621
H 4.502481 1.104124 1.549281
C 6.902539 -0.581763 -0.175676
H 6.529717 -2.256233 -1.484034
H 6.950855 1.084268 1.193139
H 7.978330 -0.594754 -0.328838
44
-1301.63444209
C -1.231891 -2.357271 1.609993
C -2.508441 -2.035305 0.812159
C -2.176108 -1.598174 -0.603638
C -3.141380 -0.705037 -1.372638
C -0.283580 -1.138903 1.637517
N 0.013901 -0.613503 0.292306
H -0.703372 -3.204912 1.159558
H -1.484651 -2.636924 2.640103
H -3.075852 -1.247666 1.323075
H -3.154420 -2.927345 0.785018
H -1.782135 -2.407642 -1.226525
H -1.065288 -0.918263 -0.374124
H -4.013945 -1.313657 -1.661009
H -2.658491 -0.414800 -2.315153
H 0.649669 -1.407088 2.145234
H -0.748116 -0.315753 2.193357
S 1.286669 -1.371726 -0.549707

O 0.912924 -1.308814 -1.968694
O 1.672343 -2.654735 0.064253
C 2.628398 -0.216732 -0.258599
C 2.624533 1.020450 -0.910055
C 3.667133 -0.571707 0.599885
C 3.672517 1.906211 -0.686479
H 1.815053 1.274731 -1.586209
C 4.710897 0.330399 0.811912
H 3.658987 -1.544407 1.079846
C 4.731361 1.577559 0.176789
H 3.674161 2.869155 -1.191929
H 5.524502 0.056220 1.478916
C 5.871263 2.544304 0.392334
H 6.505057 2.237156 1.229755
H 6.508047 2.608680 -0.499430
H 5.503597 3.556160 0.598268
C -3.624277 0.530580 -0.635911
C -4.968661 0.668583 -0.269006
C -2.735215 1.567535 -0.312731
C -5.419322 1.808661 0.400516
H -5.672620 -0.123880 -0.514522
C -3.181540 2.706322 0.357613
H -1.685482 1.477121 -0.579966
C -4.526004 2.832176 0.715956
H -6.468051 1.895172 0.673058
H -2.477809 3.499181 0.598149
H -4.873100 3.721387 1.235526
44
-1301.63428458
C -3.155669 -2.371393 -1.344384
C -3.718840 -1.071976 -0.741141
C -2.659303 0.014892 -0.679713
C -2.784384 1.046690 0.430254
C -1.980723 -2.887484 -0.506039
N -0.910342 -1.866986 -0.514563
H -2.819692 -2.193124 -2.374080
H -3.931295 -3.144800 -1.383553
H -4.094744 -1.278031 0.270531
H -4.580597 -0.727563 -1.333290
H -2.412223 0.440496 -1.659028
H -1.573071 -0.740149 -0.505239
H -2.642737 0.549440 1.397550
H -3.824947 1.414398 0.425921
H -1.570712 -3.806120 -0.940994
H -2.328616 -3.132581 0.507083
S 0.103227 -1.937584 0.838752
O 0.403835 -3.359338 1.069051
O -0.427311 -1.135337 1.958584
C 1.567238 -1.107666 0.223653
C 2.070506 -1.401430 -1.046224
C 2.248753 -0.243147 1.079461
C 3.257476 -0.804332 -1.459966
H 1.530034 -2.075505 -1.701479
C 3.440455 0.340404 0.650552
H 1.847312 -0.034454 2.065115
C 3.960547 0.075319 -0.622204
H 3.647609 -1.025865 -2.450551
H 3.973946 1.013441 1.317271
C 5.233454 0.735763 -1.095271
H 5.844915 0.047681 -1.689091
H 5.013840 1.604223 -1.730299
H 5.838411 1.089175 -0.254393
C -1.841314 2.225132 0.302040
C -2.123760 3.267618 -0.591882
C -0.667334 2.293805 1.062245
C -1.254848 4.350369 -0.727494
H -3.038077 3.234376 -1.181761
C 0.203888 3.376918 0.929183
H -0.439503 1.488987 1.755633
C -0.086008 4.407972 0.034624
H -1.493285 5.151596 -1.422462
H 1.110558 3.414710 1.527677
H 0.590651 5.252590 -0.065897
44
-1301.63403108
C -0.953669 2.762083 -1.290298
C -2.247664 2.414023 -0.528685
C -1.940860 1.887764 0.861129
C -2.947146 1.009202 1.598673
C -0.035551 1.535235 -1.404936
N 0.310009 1.083809 -0.049109
H -0.417183 3.568020 -0.773479
H -1.191077 3.121958 -2.297964
H -2.813516 1.664139 -1.093849
H -2.886332 3.309340 -0.463379
H -1.517624 2.655238 1.520754
H -0.815021 1.250574 0.615625
H -3.714839 1.677584 2.022696
H -2.438216 0.555259 2.457987
H 0.894312 1.815945 -1.920121
H -0.518177 0.744094 -1.993690
S 0.764665 -0.538294 0.128554
O 0.350805 -1.363748 -1.018525
O 0.361323 -0.913308 1.492703
C 2.553529 -0.412419 0.085677
C 3.242632 -0.799894 -1.062438
C 3.235171 0.073366 1.205243
C 4.633895 -0.695537 -1.086100
H 2.693883 -1.189169 -1.913400
C 4.621876 0.169856 1.163159
H 2.682003 0.358942 2.093724
C 5.343416 -0.211897 0.019920
H 5.175927 -1.000099 -1.978036
H 5.157086 0.544804 2.032440
C 6.850821 -0.123585 0.000292
H 7.300619 -0.934644 0.587788
H 7.244135 -0.197612 -1.018224
H 7.202050 0.819987 0.432883
C -3.633068 -0.064321 0.776283
C -4.900655 0.161014 0.221855
C -3.014051 -1.302234 0.552191
C -5.534064 -0.819384 -0.543980
H -5.400072 1.112073 0.397493
C -3.643841 -2.282263 -0.216343
H -2.035940 -1.499096 0.982015
C -4.904841 -2.045076 -0.766873
H -6.519093 -0.626317 -0.961538
H -3.144962 -3.233137 -0.382787
H -5.395251 -2.810993 -1.362073
44
-1301.63396097
C 3.894792 -1.273313 -0.225612
C 3.924347 0.254175 -0.408071
C 2.591081 0.890897 -0.053050
C 2.236710 2.201774 -0.735860
C 2.750712 -1.887163 -1.057128
N 1.431976 -1.293615 -0.755137
H 3.752494 -1.523415 0.832420
H 4.848681 -1.714091 -0.540595
H 4.181846 0.490524 -1.450548
H 4.722591 0.684610 0.217063
H 2.366974 0.868605 1.017176
H 1.748792 -0.070217 -0.466863
H 2.289111 2.073569 -1.824800
H 3.022787 2.935760 -0.484978
H 2.694750 -2.969585 -0.904576
H 2.929280 -1.705905 -2.124454
S 0.665436 -1.960017 0.621781
O 1.046584 -1.231967 1.845023
O 0.843324 -3.419561 0.556580
C -1.050472 -1.584960 0.273292
C -1.588490 -0.364303 0.679724
C -1.834405 -2.535822 -0.382566
C -2.928963 -0.093354 0.408772
H -0.974890 0.357235 1.208215
C -3.172493 -2.249142 -0.638873
H -1.399986 -3.487786 -0.668197
C -3.739046 -1.024662 -0.254017
H -3.349163 0.858636 0.722190
H -3.789773 -2.990552 -1.140789

C -5.184539 -0.714775 -0.563780
H -5.577290 0.066686 0.094384
H -5.299623 -0.362114 -1.597429
H -5.817267 -1.602134 -0.453535
C 0.885468 2.768466 -0.347805
C 0.671574 3.291733 0.936006
C -0.178852 2.778534 -1.256942
C -0.571501 3.808734 1.300827
H 1.488149 3.299046 1.654972
C -1.423902 3.299776 -0.898389
H -0.031557 2.374455 -2.255831
C -1.624436 3.816773 0.382039
H -0.715876 4.212919 2.299387
H -2.235160 3.302319 -1.621657
H -2.590441 4.229036 0.661671
44
-1301.63387317
C 3.892042 -1.284385 -0.218648
C 3.926022 0.242809 -0.402897
C 2.593756 0.883405 -0.051142
C 2.244142 2.194699 -0.735624
C 2.748061 -1.896160 -1.051840
N 1.430289 -1.298671 -0.753419
H 3.746866 -1.532802 0.839390
H 4.845400 -1.728100 -0.531107
H 4.186093 0.477201 -1.445179
H 4.724218 0.671936 0.223200
H 2.367297 0.862386 1.018611
H 1.749902 -0.075713 -0.466323
H 2.297214 2.065281 -1.824387
H 3.032165 2.926556 -0.484662
H 2.688825 -2.978251 -0.898175
H 2.929393 -1.716602 -2.118989
S 0.658815 -1.961139 0.622580
O 1.039692 -1.233368 1.846081
O 0.832051 -3.421311 0.558972
C -1.055310 -1.581039 0.271099
C -1.590606 -0.358879 0.677081
C -1.838379 -2.525774 -0.394290
C -2.928030 -0.081523 0.398509
H -0.975170 0.361591 1.204893
C -3.173661 -2.232590 -0.658485
H -1.403968 -3.475353 -0.687779
C -3.739219 -1.009784 -0.267543
H -3.344177 0.874425 0.705322
H -3.788541 -2.966205 -1.174573
C -5.196785 -0.715848 -0.532883
H -5.389691 0.361319 -0.563521
H -5.526892 -1.148583 -1.483398
H -5.833802 -1.139810 0.254749
C 0.894229 2.765709 -0.349288
C 0.681460 3.293094 0.933023
C -0.169997 2.775657 -1.258537
C -0.560440 3.813989 1.296288
H 1.498000 3.300507 1.652031
C -1.413868 3.300809 -0.901570
H -0.023633 2.368349 -2.256243
C -1.613291 3.821888 0.377389
H -0.703967 4.221307 2.293696
H -2.224987 3.303174 -1.625000
H -2.578317 4.237270 0.655815
44
-1301.63380726
C -0.841285 2.573408 -0.360073
C -2.078640 1.701990 -0.639388
C -1.902333 0.308920 -0.060468
C -2.655634 -0.860898 -0.679017
C 0.429353 1.925914 -0.951796
N 0.603761 0.522125 -0.537472
H -0.703040 2.699043 0.719903
H -0.968713 3.573016 -0.793420
H -2.246206 1.636780 -1.723962
H -2.971306 2.181624 -0.211335
H -1.910644 0.291613 1.034016
H -0.622010 0.126272 -0.313983
H -2.238449 -1.796272 -0.287512
H -2.500919 -0.866154 -1.765096
H 1.309806 2.511036 -0.663740
H 0.370225 1.921501 -2.047259
S 1.418149 0.269245 0.936473
O 0.811996 -0.933856 1.521680
O 1.530617 1.507124 1.728480
C 3.064105 -0.142232 0.352967
C 4.113193 0.748022 0.572297
C 3.278760 -1.361247 -0.297908
C 5.392902 0.412155 0.126945
H 3.925979 1.680446 1.093909
C 4.559676 -1.678197 -0.735006
H 2.452359 -2.047214 -0.451297
C 5.636715 -0.798827 -0.530842
H 6.214845 1.102769 0.298640
H 4.731420 -2.625231 -1.241376
C 7.024301 -1.167745 -0.998807
H 7.019883 -1.488821 -2.047044
H 7.716273 -0.325230 -0.906223
H 7.431944 -1.999272 -0.409763
C -4.144543 -0.797277 -0.373031
C -5.052758 -0.275265 -1.303241
C -4.633459 -1.232470 0.867210
C -6.414146 -0.191735 -1.005315
H -4.691888 0.059390 -2.273411
C -5.992706 -1.150470 1.168382
H -3.942035 -1.644597 1.599141
C -6.888419 -0.628206 0.232477
H -7.104092 0.210059 -1.742993
H -6.353437 -1.498583 2.132807
H -7.948094 -0.566138 0.465139
44
-1301.63379032
C -2.368124 -3.146107 -1.384552
C -3.207374 -2.034069 -0.731658
C -2.454768 -0.715652 -0.725902
C -2.791414 0.274629 0.373120
C -1.073365 -3.372950 -0.594530
N -0.291171 -2.114607 -0.570844
H -2.125661 -2.872996 -2.419833
H -2.929867 -4.086586 -1.419032
H -3.456454 -2.324821 0.298174
H -4.162165 -1.920135 -1.267772
H -2.360353 -0.270321 -1.722623
H -1.212328 -1.192982 -0.577181
H -2.379694 -0.104381 1.321016
H -3.884642 0.278981 0.513444
H -0.456710 -4.139019 -1.078610
H -1.319594 -3.737972 0.411796
S 0.637001 -1.947866 0.841196
O 1.339858 -3.225471 1.032550
O -0.166502 -1.392404 1.945709
C 1.824832 -0.703806 0.343017
C 1.854487 0.519356 1.008543
C 2.748135 -0.997772 -0.665285
C 2.817544 1.464316 0.648931
H 1.132888 0.723381 1.791743
C 3.697645 -0.043424 -1.012182
H 2.718307 -1.959196 -1.167053
C 3.750175 1.200490 -0.360407
H 2.841552 2.420849 1.164753
H 4.414682 -0.265713 -1.798987
C 4.804146 2.215603 -0.733781
H 4.594269 3.193688 -0.290380
H 5.796107 1.899838 -0.385753
H 4.870387 2.342214 -1.820537
C -2.325554 1.700565 0.143943
C -3.107723 2.772327 0.596203
C -1.112497 1.990523 -0.494796
C -2.692201 4.092564 0.422144
H -4.055407 2.568631 1.090547
C -0.693221 3.310414 -0.672754
H -0.483635 1.181964 -0.856066
C -1.480907 4.367234 -0.215215

H -3.317921 4.906381 0.779709
H 0.252779 3.509040 -1.169937
H -1.156107 5.394805 -0.355761
44
-1301.63340529
C -0.859012 2.386489 0.512004
C -1.994305 1.654654 1.253262
C -1.911408 0.144889 1.067110
C -2.458330 -0.459427 -0.224300
C 0.515912 1.877664 0.991442
N 0.643596 0.410266 0.921897
H -0.923013 3.467135 0.689380
H -0.937518 2.227885 -0.568894
H -2.965919 2.022808 0.892305
H -1.942184 1.898156 2.322074
H -2.190693 -0.432523 1.954191
H -0.600362 -0.001434 1.047857
H -2.058221 -1.472556 -0.338347
H -2.098430 0.112335 -1.087241
H 0.668395 2.151894 2.042688
H 1.312255 2.349692 0.404119
S 1.183084 -0.222536 -0.561545
O 0.937283 0.694813 -1.690118
O 0.673807 -1.599170 -0.607030
C 2.954839 -0.268429 -0.285372
C 3.786730 0.580679 -1.012786
C 3.482011 -1.171996 0.642909
C 5.165313 0.524792 -0.801319
H 3.355755 1.262039 -1.738499
C 4.857490 -1.212827 0.840250
H 2.819794 -1.830791 1.194677
C 5.721052 -0.367516 0.122959
H 5.818336 1.183635 -1.368340
H 5.272400 -1.913408 1.561151
C 7.214277 -0.440693 0.335551
H 7.466159 -0.450609 1.402210
H 7.630214 -1.357563 -0.101836
H 7.727646 0.408126 -0.126292
C -3.977770 -0.493138 -0.222535
C -4.667307 -1.497061 0.473287
C -4.724825 0.487027 -0.889526
C -6.061319 -1.520477 0.503112
H -4.103778 -2.272166 0.988635
C -6.120865 0.467531 -0.862146
H -4.207725 1.263786 -1.448713
C -6.793840 -0.535629 -0.163751
H -6.576583 -2.311150 1.042549
H -6.681387 1.233258 -1.392364
H -7.880217 -0.554185 -0.143599
44
-1301.63323368
C 0.504303 3.240478 -1.325900
C 1.845534 2.998165 -0.613112
C 2.178171 1.517526 -0.570896
C 3.108914 1.027715 0.533366
C -0.639260 2.547912 -0.569008
N -0.357698 1.106311 -0.466768
H 0.553867 2.856797 -2.353025
H 0.285400 4.312525 -1.388875
H 1.794921 3.399156 0.408845
H 2.647844 3.548677 -1.128582
H 2.408273 1.101324 -1.558075
H 0.959910 1.030399 -0.421693
H 2.555855 0.973200 1.480496
H 3.882580 1.797619 0.685107
H -1.579752 2.672290 -1.124497
H -0.767116 3.009214 0.418663
S -0.948099 0.334650 0.925733
O -1.195624 1.284221 2.024130
O -0.069675 -0.823234 1.141401
C -2.538448 -0.259833 0.351209
C -3.705868 0.360156 0.793486
C -2.587323 -1.342832 -0.531895
C -4.938056 -0.113013 0.340554
H -3.643666 1.189331 1.490120
C -3.824393 -1.799574 -0.972768
H -1.668185 -1.818308 -0.857610
C -5.018105 -1.194504 -0.545055
H -5.851666 0.364737 0.685646
H -3.868185 -2.642180 -1.658849
C -6.353604 -1.718376 -1.016485
H -6.350905 -1.906100 -2.096200
H -6.599759 -2.668624 -0.525026
H -7.161629 -1.014083 -0.796318
C 3.782446 -0.297093 0.235039
C 3.138877 -1.513766 0.501002
C 5.060904 -0.323785 -0.338122
C 3.764035 -2.724644 0.198744
H 2.144704 -1.507438 0.938330
C 5.686884 -1.534134 -0.640565
H 5.574909 0.613460 -0.543795
C 5.038405 -2.740354 -0.371977
H 3.252929 -3.659738 0.413784
H 6.681169 -1.533764 -1.080263
H 5.523851 -3.685220 -0.602496

TS-1,6-HAT$_{uni}$

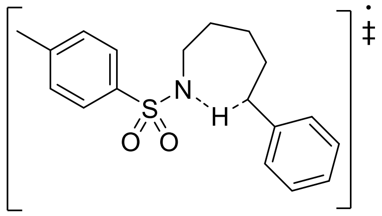

Name	E(B3LYP)	G(B3LYP)	E(RO-B2PLYP-D3)	G(RO-B2PLYP-D3)	NImag
Tosyl_NH_pentane_Ph_1_6_HAT_TS_0002_cregen_B2PLYP	-1302.426188	-1302.042582	-1301.946806	-1301.63791727	-1483.7522
Tosyl_NH_pentane_Ph_1_6_HAT_TS_0001_cregen_B2PLYP	-1302.426187	-1302.042579	-1301.946779	-1301.63779235	-1482.4409
Tosyl_NH_pentane_Ph_1_6_HAT_TS_0015_cregen_B2PLYP	-1302.426177	-1302.042571	-1301.946213	-1301.63746639	-1483.1920
Tosyl_NH_pentane_Ph_1_6_HAT_TS_0014_cregen_B2PLYP	-1302.426188	-1302.042581	-1301.946173	-1301.63725071	-1483.0456
Tosyl_NH_pentane_Ph_1_6_HAT_TS_0000_cregen_B2PLYP	-1302.426177	-1302.042571	-1301.945956	-1301.63720763	-1482.9049
Tosyl_NH_pentane_Ph_1_6_HAT_TS_0004_cregen_B2PLYP	-1302.426177	-1302.042571	-1301.945913	-1301.63716703	-1483.3321
Tosyl_NH_pentane_Ph_1_6_HAT_TS_0005_cregen_B2PLYP	-1302.426188	-1302.042581	-1301.945848	-1301.63693231	-1483.6941
Tosyl_NH_pentane_Ph_1_6_HAT_TS_0003_cregen_B2PLYP	-1302.426188	-1302.042581	-1301.945838	-1301.63689518	-1483.1164
Tosyl_NH_pentane_Ph_1_6_HAT_TS_0012_cregen_B2PLYP	-1302.426188	-1302.042582	-1301.943608	-1301.63469339	-1483.1049
Tosyl_NH_pentane_Ph_1_6_HAT_TS_0010_cregen_B2PLYP	-1302.426188	-1302.042582	-1301.943610	-1301.63468201	-1483.0756

44
-1301.63791727
C -2.965416 -2.374910 -1.359757
C -3.946610 -1.203565 -1.169118
C -3.329015 0.205272 -1.200811
C -2.428918 0.499645 -0.007653
C -1.958349 -2.607860 -0.226365
N -0.880227 -1.600555 -0.273235
H -4.490319 -1.338757 -0.222475
H -4.702033 -1.262898 -1.963248
H -4.137338 0.951595 -1.228906
H -2.768384 0.326575 -2.137243
H -1.528679 -0.396894 -0.117077
H -2.476153 -2.628029 0.745555
H -3.553905 -3.296149 -1.462589
H -2.410848 -2.250346 -2.299289
H -2.883442 0.198888 0.942686
S 0.210680 -1.733740 1.029707
O 0.482332 -3.164903 1.238235
O -0.239310 -0.901335 2.159153
C 1.682945 -0.984296 0.335472
C 2.599848 -1.792990 -0.338536
C 1.904941 0.384347 0.484587
C 3.746730 -1.214381 -0.875822
H 2.415824 -2.858244 -0.427699
C 3.057449 0.945879 -0.063006
H 1.191484 0.998693 1.022786
C 3.994397 0.160367 -0.747660
H 4.463957 -1.840748 -1.400989
H 3.231431 2.013436 0.047318
C 5.256899 0.771644 -1.307529
H 6.086284 0.685853 -0.592886
H 5.570782 0.270258 -2.229234
H 5.124320 1.836039 -1.526626
H -1.481846 -3.587451 -0.346152
C -1.744256 1.804337 0.100592
C -1.348682 2.274012 1.371826
C -1.437087 2.598017 -1.022668
C -0.687907 3.490825 1.513657
H -1.555769 1.662074 2.245166
C -0.777495 3.816446 -0.878597
H -1.719794 2.261661 -2.015371
C -0.400345 4.269857 0.388282
H -0.399134 3.834911 2.503280
H -0.556941 4.415553 -1.758235
H 0.112259 5.221734 0.498081
44
-1301.63779235
C -2.960782 -2.374471 -1.366519
C -3.942742 -1.203627 -1.176767
C -3.325590 0.205469 -1.205610
C -2.428464 0.499030 -0.010029
C -1.956032 -2.608389 -0.231279
N -0.878465 -1.600356 -0.274311
H -4.488459 -1.340005 -0.231449
H -4.696437 -1.262381 -1.972580
H -4.134139 0.951503 -1.234919
H -2.762772 0.327898 -2.140583
H -1.527768 -0.397162 -0.118056
H -2.475942 -2.630357 0.739480
H -3.548799 -3.295769 -1.471530
H -2.404287 -2.248798 -2.304765
H -2.885241 0.197411 0.938957
S 0.209950 -1.734690 1.030567
O 0.481272 -3.166011 1.238433
O -0.242249 -0.903238 2.159824
C 1.682879 -0.984316 0.338586
C 2.604466 -1.793565 -0.328516

C 1.905090 0.383913 0.490255
C 3.754748 -1.215374 -0.858716
H 2.424664 -2.860046 -0.411386
C 3.061219 0.945102 -0.050308
H 1.192637 0.997023 1.031185
C 3.998973 0.160497 -0.734698
H 4.479107 -1.843479 -1.371886
H 3.238898 2.011125 0.068316
C 5.235665 0.780976 -1.340197
H 5.503014 1.715715 -0.837147
H 6.094605 0.104073 -1.280061
H 5.079976 1.013451 -2.402161
H -1.478634 -3.587493 -0.351497
C -1.744204 1.803708 0.100924
C -1.350960 2.271967 1.373399
C -1.435142 2.598735 -1.020865
C -0.690578 3.488685 1.517811
H -1.559527 1.658992 2.245658
C -0.775927 3.817059 -0.874214
H -1.716132 2.263540 -2.014450
C -0.401066 4.269034 0.393855
H -0.403609 3.831657 2.508344
H -0.553884 4.417230 -1.752752
H 0.111283 5.220814 0.505654
44
-1301.63746639
C -1.055783 -2.081781 1.922931
C 0.121651 -1.751156 2.859880
C 1.021832 -0.582155 2.416085
C 1.882603 -0.897523 1.202090
C -0.702505 -2.822643 0.617360
N 0.133260 -2.113800 -0.360229
H 0.734001 -2.652507 3.012074
H -0.294488 -1.498778 3.843445
H 1.675934 -0.296868 3.253590
H 0.385777 0.289214 2.212386
H 1.107892 -1.355885 0.339014
H -0.156387 -3.741138 0.870530
H -1.748001 -2.739376 2.466000
H -1.615964 -1.166773 1.696299
H 2.479722 -1.806407 1.354413
S -0.594650 -1.197987 -1.567837
O -1.493802 -2.123403 -2.276287
O 0.507763 -0.519609 -2.252479
C -1.638663 0.088330 -0.862641
C -1.072176 1.314575 -0.504544
C -3.003053 -0.146386 -0.681049
C -1.881945 2.299451 0.057506
H -0.017023 1.495416 -0.681783
C -3.798040 0.850983 -0.117794
H -3.432902 -1.091058 -0.996828
C -3.253466 2.085769 0.261263
H -1.443938 3.256149 0.332866
H -4.861334 0.670213 0.021098
C -4.127663 3.173977 0.837767
H -4.495219 3.842397 0.047894
H -5.003758 2.758318 1.345946
H -3.578338 3.792400 1.555481
H -1.623573 -3.134376 0.109670
C 2.682371 0.172024 0.558726
C 2.515137 1.538839 0.854259
C 3.645532 -0.182349 -0.408634
C 3.289169 2.507888 0.217432
H 1.790700 1.846583 1.601859
C 4.412366 0.784610 -1.048087
H 3.774917 -1.231274 -0.662686
C 4.238611 2.136769 -0.736935
H 3.153296 3.556249 0.470288
H 5.144854 0.486676 -1.793142
H 4.837864 2.893981 -1.234977
44
-1301.63725071
C -1.063880 -2.068535 1.931006
C 0.111719 -1.732717 2.868413
C 1.014062 -0.567565 2.418911
C 1.877809 -0.892200 1.209466
C -0.708274 -2.817796 0.630869
N 0.130353 -2.115839 -0.349228
H 0.722869 -2.633601 3.027994
H -0.306417 -1.473474 3.849354
H 1.666156 -0.276962 3.256148
H 0.379358 0.302916 2.207327
H 1.104747 -1.355031 0.347378
H -0.163591 -3.735206 0.890963
H -1.757725 -2.722239 2.476694
H -1.622977 -1.154653 1.697252
H 2.473159 -1.800910 1.369541
S -0.594012 -1.206101 -1.563580
O -1.493561 -2.134248 -2.267923
O 0.510643 -0.533700 -2.250464
C -1.636753 0.085824 -0.866621
C -3.002689 -0.143672 -0.690437
C -1.069498 1.314629 -0.518425
C -3.798093 0.860713 -0.140219
H -3.434591 -1.087047 -1.007287
C -1.879658 2.306410 0.030677
H -0.014962 1.494387 -0.700291
C -3.251477 2.095467 0.235885
H -4.863865 0.686571 -0.012618
H -1.442388 3.267599 0.291049
C -4.114256 3.169317 0.854968
H -5.160406 3.069545 0.548486
H -4.088487 3.113108 1.951505
H -3.771202 4.170585 0.573963
H -1.628485 -3.131758 0.122995
C 2.681009 0.171895 0.561387
C 3.647198 -0.190350 -0.400017
C 2.514239 1.540944 0.846657
C 4.417347 0.771232 -1.043586
H 3.776383 -1.241192 -0.646138
C 3.291580 2.504622 0.205743
H 1.787412 1.854771 1.589387
C 4.243976 2.125741 -0.742618
H 5.152153 0.467246 -1.783898
H 3.155953 3.554892 0.450682
H 4.845814 2.878769 -1.243879
44
-1301.63720763
C 0.537787 3.270903 -0.963218
C 2.045106 3.378161 -0.666075
C 2.864410 2.096588 -0.896749
C 2.580845 0.988870 0.110982
C -0.287161 2.452307 0.037538
N -0.047326 1.010504 -0.118375
H 2.187458 3.724850 0.368091
H 2.460296 4.164840 -1.309153
H 3.936278 2.342083 -0.857410
H 2.670564 1.737849 -1.915819
H 1.334683 0.817458 0.002892
H -0.100989 2.794834 1.065254
H 0.118031 4.285424 -0.967408
H 0.383388 2.863926 -1.971372
H 2.648814 1.348046 1.144763
S -0.732769 0.055888 1.115037
O -0.968937 0.830870 2.346230
O 0.062890 -1.176604 1.162339
C -2.331528 -0.323582 0.396034
C -2.413331 -1.272688 -0.627587
C -3.472162 0.327346 0.864512
C -3.654991 -1.563457 -1.182256
H -1.515656 -1.776516 -0.970250
C -4.709236 0.022380 0.295698
H -3.386572 1.046990 1.671826
C -4.821166 -0.922847 -0.731503
H -3.724460 -2.302299 -1.977235
H -5.602138 0.524148 0.660556
C -6.164522 -1.268400 -1.328481
H -6.922105 -0.521527 -1.071934
H -6.111073 -1.337535 -2.420833
H -6.520633 -2.239800 -0.961229

H -1.357353 2.621540 -0.157184
C 3.222474 -0.333062 -0.053824
C 3.355352 -1.181485 1.065287
C 3.693040 -0.801179 -1.297072
C 3.949587 -2.433088 0.951627
H 2.971681 -0.849992 2.025843
C 4.290628 -2.053946 -1.409772
H 3.598639 -0.179095 -2.181520
C 4.423406 -2.875251 -0.287230
H 4.041090 -3.067900 1.828680
H 4.654396 -2.391329 -2.376853
H 4.888403 -3.853270 -0.377698
44
-1301.63716703
C 3.141808 -2.395144 -0.262372
C 4.042362 -1.151947 -0.372787
C 3.441649 0.166913 0.149423
C 2.327657 0.718239 -0.730161
C 1.890268 -2.406712 -1.160134
N 0.819377 -1.439121 -0.854554
H 4.354525 -1.021046 -1.420321
H 4.961266 -1.352998 0.192628
H 4.240166 0.921502 0.212690
H 3.072305 0.012154 1.169130
H 1.460498 -0.210681 -0.777487
H 2.175485 -2.205531 -2.200602
H 3.737657 -3.273722 -0.544829
H 2.843084 -2.549989 0.781478
H 2.617668 0.745211 -1.788410
S 0.021396 -1.714437 0.622884
O 0.614661 -0.894075 1.695082
O -0.104073 -3.170381 0.804917
C -1.608602 -1.057762 0.269088
C -2.585946 -1.911846 -0.244570
C -1.887173 0.285718 0.519473
C -3.853968 -1.404632 -0.516668
H -2.352892 -2.957271 -0.416026
C -3.161376 0.775580 0.237454
H -1.122925 0.935235 0.932181
C -4.162453 -0.056810 -0.281194
H -4.618550 -2.066908 -0.915854
H -3.381022 1.823347 0.427521
C -5.549901 0.475319 -0.551113
H -5.534464 1.551193 -0.753161
H -6.012171 -0.025879 -1.408156
H -6.208371 0.314511 0.312905
H 1.436471 -3.403658 -1.132116
C 1.614637 1.951714 -0.332833
C 1.419801 2.313567 1.016267
C 1.076984 2.794272 -1.327753
C 0.732152 3.478907 1.348768
H 1.794057 1.670277 1.804650
C 0.389266 3.957179 -0.994318
H 1.211128 2.527511 -2.373570
C 0.214983 4.306704 0.347896
H 0.597948 3.742058 2.394706
H -0.008426 4.593979 -1.780128
H -0.318995 5.215646 0.611723
44
-1301.63693231
C 3.138960 -2.398545 -0.262954
C 4.041075 -1.156351 -0.372189
C 3.441442 0.162890 0.150315
C 2.328580 0.715883 -0.729648
C 1.887486 -2.408312 -1.160842
N 0.817790 -1.439458 -0.855034
H 4.354178 -1.025256 -1.419414
H 4.959292 -1.358794 0.193842
H 4.240645 0.916666 0.214575
H 3.071255 0.007903 1.169684
H 1.460390 -0.211856 -0.777626
H 2.172935 -2.207275 -2.201270
H 3.733849 -3.277633 -0.545860
H 2.839857 -2.553791 0.780734
H 2.619168 0.742857 -1.787739
S 0.019574 -1.713921 0.622491
O 0.613184 -0.893122 1.694171
O -0.106510 -3.169692 0.805397
C -1.610081 -1.056518 0.268386
C -2.588792 -1.910897 -0.241376
C -1.886855 0.288360 0.514874
C -3.856791 -1.402893 -0.513682
H -2.357190 -2.957106 -0.409976
C -3.160493 0.778772 0.232782
H -1.121299 0.938238 0.924592
C -4.163389 -0.054303 -0.282129
H -4.622572 -2.065444 -0.909980
H -3.378604 1.827486 0.419623
C -5.550167 0.480178 -0.550790
H -6.186989 0.387617 0.339113
H -5.525558 1.540394 -0.823472
H -6.041904 -0.068820 -1.360542
H 1.432528 -3.404734 -1.132983
C 1.616973 1.950141 -0.332181
C 1.420813 2.310977 1.017009
C 1.082349 2.794686 -1.327035
C 0.734899 3.477297 1.349670
H 1.792599 1.666107 1.805270
C 0.396425 3.958615 -0.993435
H 1.217499 2.528710 -2.372922
C 0.220861 4.307137 0.348871
H 0.599603 3.739616 2.395677
H 0.001147 4.596990 -1.779188
H -0.311714 5.216865 0.612829
44
-1301.63689518
C 3.144278 -2.390957 -0.269265
C 4.042721 -1.146354 -0.381088
C 3.441233 0.171179 0.143560
C 2.324419 0.721558 -0.733038
C 1.890567 -2.403818 -1.163986
N 0.819014 -1.437912 -0.855407
H 4.352066 -1.014260 -1.429307
H 4.963324 -1.346399 0.181912
H 4.238717 0.926945 0.205753
H 3.074367 0.014946 1.163938
H 1.458549 -0.208606 -0.779024
H 2.173016 -2.201705 -2.205030
H 3.740710 -3.268490 -0.553730
H 2.848349 -2.546882 0.775218
H 2.611938 0.749810 -1.791932
S 0.024676 -1.715062 0.623667
O 0.619055 -0.894188 1.694853
O -0.098223 -3.171255 0.805432
C -1.606707 -1.060276 0.272464
C -2.585976 -1.917115 -0.232846
C -1.887010 0.282410 0.525162
C -3.856882 -1.412826 -0.496907
H -2.354160 -2.963973 -0.397074
C -3.164094 0.769332 0.251150
H -1.123990 0.931804 0.940325
C -4.164924 -0.064013 -0.266465
H -4.625022 -2.079037 -0.882429
H -3.387166 1.814732 0.449961
C -5.537473 0.479322 -0.585701
H -6.315734 -0.272559 -0.417390
H -5.773764 1.356648 0.025005
H -5.604533 0.785961 -1.638118
H 1.438248 -3.401422 -1.135423
C 1.610449 1.953595 -0.332983
C 1.069888 2.796651 -1.325896
C 1.417543 2.313517 1.016915
C 0.381210 3.958224 -0.989793
H 1.202562 2.531379 -2.372282
C 0.728941 3.477526 1.352083
H 1.794013 1.669711 1.803822
C 0.208869 4.305860 0.353160
H -0.018742 4.595475 -1.774090
H 0.596252 3.739204 2.398584
H -0.325874 5.213748 0.619056

44
-1301.63469339
C -0.494396 3.292625 -0.780082
C -1.867061 3.492090 -0.099373
C -2.550616 2.208583 0.411022
C -2.533856 1.077095 -0.609933
C 0.419251 2.261805 -0.097453
N 0.109788 0.907396 -0.592039
H -2.531146 4.001418 -0.810631
H -1.751737 4.175502 0.751944
H -3.586904 2.441261 0.694294
H -2.054340 1.871934 1.329773
H -1.286141 0.818478 -0.693915
H 1.466022 2.463413 -0.368125
H -0.621614 2.985281 -1.825351
H 0.023381 4.258973 -0.807956
H -2.724090 1.427065 -1.631765
S 0.530237 -0.353076 0.469520
O -0.107618 -1.557888 -0.069595
O 0.320944 0.027341 1.880758
C 2.299022 -0.470000 0.194044
C 2.767400 -1.088199 -0.969253
C 3.185333 0.050614 1.136774
C 4.139122 -1.177178 -1.183014
H 2.061607 -1.497850 -1.684159
C 4.557351 -0.049217 0.906003
H 2.799318 0.510114 2.040545
C 5.054991 -0.661547 -0.251584
H 4.508562 -1.657871 -2.085829
H 5.252303 0.351128 1.640193
C 6.541367 -0.791689 -0.484171
H 6.799663 -0.613411 -1.533830
H 7.107580 -0.085121 0.130635
H 6.891847 -1.801335 -0.232449
H 0.344805 2.337130 0.996201
C -3.249980 -0.180735 -0.320703
C -3.805519 -0.925075 -1.381232
C -3.389473 -0.684790 0.988378
C -4.492301 -2.111205 -1.144788
H -3.697036 -0.556129 -2.398553
C -4.075447 -1.872094 1.223884
H -2.942147 -0.152830 1.821971
C -4.632506 -2.589163 0.161142
H -4.919115 -2.664453 -1.977135
H -4.169994 -2.245226 2.240006
H -5.165827 -3.517158 0.348828

44
-1301.63468201
C -0.494664 3.292992 -0.780097
C -1.866890 3.492182 -0.098431
C -2.550134 2.208416 0.411738
C -2.533869 1.077384 -0.609716
C 0.419429 2.262046 -0.098236
N 0.109752 0.907705 -0.592938
H -2.531410 4.001969 -0.808953
H -1.750935 4.175104 0.753196
H -3.586277 2.440888 0.695707
H -2.053309 1.871383 1.330051
H -1.286216 0.818957 -0.694603
H 1.466044 2.463741 -0.369433
H -0.622511 2.985910 -1.825367
H 0.023042 4.259377 -0.808046
H -2.724805 1.427764 -1.631279
S 0.529681 -0.352886 0.468738
O -0.107995 -1.557632 -0.070744
O 0.319694 0.027473 1.879900
C 2.298572 -0.469860 0.194040
C 2.767469 -1.088167 -0.969213
C 3.184399 0.050109 1.137312
C 4.139089 -1.177615 -1.182172
H 2.061894 -1.497809 -1.684341
C 4.556704 -0.050229 0.907273
H 2.798109 0.509211 2.041167
C 5.054732 -0.662135 -0.250114
H 4.508861 -1.658690 -2.084686
H 5.251369 0.349287 1.642142
C 6.540996 -0.790527 -0.484462
H 6.882955 -1.815623 -0.290684
H 6.804465 -0.554253 -1.521549
H 7.110074 -0.124221 0.171110
H 0.345537 2.337158 0.995467
C -3.249559 -0.180707 -0.320537
C -3.805203 -0.924985 -1.381055
C -3.388462 -0.685088 0.988479
C -4.491544 -2.111377 -1.144649
H -3.697154 -0.555783 -2.398330
C -4.074001 -1.872653 1.223945
H -2.941018 -0.153176 1.822040
C -4.631185 -2.589658 0.161225
H -4.918452 -2.664577 -1.976978
H -4.168108 -2.246037 2.240016
H -5.164169 -3.517854 0.348881

N-rad$_{gm}$

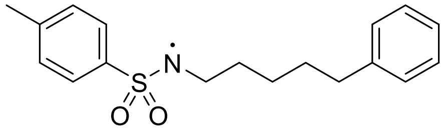

Name	E(B3LYP)	G(B3LYP)	E(RO-B2PLYP-D3)	G(RO-B2PLYP-D3)
Conformers				
Tosyl_N_pentane_Ph_radical_0007_cregen_B2PLYP	-1302.445291	-1302.056098	-1301.959840	-1301.65239562
Tosyl_N_pentane_Ph_radical_0009_cregen_B2PLYP	-1302.445539	-1302.056781	-1301.959801	-1301.65214015
Tosyl_N_pentane_Ph_radical_0003_cregen_B2PLYP	-1302.445291	-1302.056098	-1301.959532	-1301.65208305
Tosyl_N_pentane_Ph_radical_0001_cregen_B2PLYP	-1302.445512	-1302.056632	-1301.959530	-1301.65097493
Tosyl_N_pentane_Ph_radical_0006_cregen_B2PLYP	-1302.445957	-1302.056499	-1301.959553	-1301.64866334
Tosyl_N_pentane_Ph_radical_0015_cregen_B2PLYP	-1302.446982	-1302.058066	-1301.956913	-1301.64848689
Tosyl_N_pentane_Ph_radical_0019_cregen_B2PLYP	-1302.446982	-1302.058066	-1301.957011	-1301.64839666
Tosyl_N_pentane_Ph_radical_0002_cregen_B2PLYP	-1302.445512	-1302.056632	-1301.956591	-1301.64802272
Tosyl_N_pentane_Ph_radical_0016_cregen_B2PLYP	-1302.446989	-1302.058072	-1301.956920	-1301.64801046
Tosyl_N_pentane_Ph_radical_0000_cregen_B2PLYP	-1302.442346	-1302.053639	-1301.956591	-1301.64797293
Pre-reactive complexes for 1,5-HAT				
Tosyl_NH_pentane_Ph_1_5_HAT_TS_0001_cregen_B2PLYP_IRC_reverse_opt_cregen_B2PLYP_ric	-1302.444455	-1302.055224	-1301.955292	-1301.64703138
Tosyl_NH_pentane_Ph_1_5_HAT_TS_0000_cregen_B2PLYP_IRC_reverse_opt_cregen_B2PLYP_ric	-1302.445247	-1302.056469	-1301.957413	-1301.65041682
Tosyl_NH_pentane_Ph_1_5_HAT_TS_0002_cregen_B2PLYP_IRC_reverse_opt_cregen_B2PLYP_ric	-1302.446989	-1302.058070	-1301.959552	-1301.65087931
Tosyl_NH_pentane_Ph_1_5_HAT_TS_0003_cregen_B2PLYP_IRC_reverse_opt_cregen_B2PLYP_ric	-1302.444463	-1302.055231	-1301.955502	-1301.64712386
Tosyl_NH_pentane_Ph_1_5_HAT_TS_0004_cregen_B2PLYP_IRC_reverse_opt_cregen_B2PLYP_ric	-1302.445291	-1302.056098	-1301.956621	-1301.64917291
Tosyl_NH_pentane_Ph_1_5_HAT_TS_0007_cregen_B2PLYP_IRC_reverse_opt_cregen_B2PLYP_ric	-1302.445291	-1302.056098	-1301.956626	-1301.64916312
Tosyl_NH_pentane_Ph_1_5_HAT_TS_0006_cregen_B2PLYP_IRC_reverse_opt_cregen_B2PLYP_ric	-1302.443980	-1302.054591	-1301.955573	-1301.64658842
Tosyl_NH_pentane_Ph_1_5_HAT_TS_0008_cregen_B2PLYP_IRC_reverse_opt_cregen_B2PLYP_ric	-1302.445512	-1302.056633	-1301.957674	-1301.64910916
Tosyl_NH_pentane_Ph_1_5_HAT_TS_0010_cregen_B2PLYP_IRC_reverse_opt_cregen_B2PLYP_ric	-1302.446989	-1302.058072	-1301.959553	-1301.65066652
Tosyl_NH_pentane_Ph_1_5_HAT_TS_0009_cregen_B2PLYP_IRC_reverse_opt_cregen_B2PLYP_ric	-1302.446982	-1302.058066	-1301.959529	-1301.65107136
Pre-reactive complexes for 1,6-HAT				
Tosyl_NH_pentane_Ph_1_6_HAT_TS_0000_cregen_B2PLYP_IRC_reverse_opt_cregen_B2PLYP_ric	-1302.446989	-1302.058072	-1301.959552	-1301.65066963
Tosyl_NH_pentane_Ph_1_6_HAT_TS_0001_cregen_B2PLYP_IRC_reverse_opt_cregen_B2PLYP_ric	-1302.446982	-1302.058066	-1301.959528	-1301.65086809
Tosyl_NH_pentane_Ph_1_6_HAT_TS_0003_cregen_B2PLYP_IRC_reverse_opt_cregen_B2PLYP_ric	-1302.442053	-1302.052593	-1301.953127	-1301.64241189
Tosyl_NH_pentane_Ph_1_6_HAT_TS_0002_cregen_B2PLYP_IRC_reverse_opt_cregen_B2PLYP_ric	-1302.446989	-1302.058072	-1301.959552	-1301.65066181
Tosyl_NH_pentane_Ph_1_6_HAT_TS_0004_cregen_B2PLYP_IRC_reverse_opt_cregen_B2PLYP_ric	-1302.442053	-1302.052593	-1301.953126	-1301.64240642
Tosyl_NH_pentane_Ph_1_6_HAT_TS_0005_cregen_B2PLYP_IRC_reverse_opt_cregen_B2PLYP_ric	-1302.442053	-1302.052593	-1301.953127	-1301.64241097
Tosyl_NH_pentane_Ph_1_6_HAT_TS_0007_cregen_B2PLYP_IRC_reverse_opt_cregen_B2PLYP_ric	-1302.440358	-1302.051008	-1301.951852	-1301.64327275

Tosyl_NH_pentane_Ph_1_6_HAT_TS_0006_cregen_B2PLYP_IRC_reverse_opt_cregen_B2PLYP_ric	-1302.442069	-1302.053284	-1301.954586	-1301.64621444
Tosyl_NH_pentane_Ph_1_6_HAT_TS_0009_cregen_B2PLYP_IRC_reverse_opt_cregen_B2PLYP_ric	-1302.445957	-1302.056500	-1301.956920	-1301.64590809
Tosyl_NH_pentane_Ph_1_6_HAT_TS_0008_cregen_B2PLYP_IRC_reverse_opt_cregen_B2PLYP_ric	-1302.442069	-1302.053284	-1301.954586	-1301.64621011
Tosyl_NH_pentane_Ph_1_6_HAT_TS_0000_cregen_B2PLYP_IRC_reverse_opt_cregen_B2PLYP_ric	-1302.446989	-1302.058072	-1301.959552	-1301.65066963

44
-1301.65239562
C -0.237876 -3.358936 -0.417876
N -0.609695 -1.951609 -0.427701
S -1.997870 -1.648523 0.514714
O -3.116351 -2.419288 -0.049482
O -1.609961 -1.810426 1.924839
C -2.288844 0.084289 0.198276
C -1.674248 1.042541 1.005371
C -3.142985 0.454737 -0.841761
C -1.916087 2.391725 0.753303
H -1.027722 0.734461 1.819996
C -3.370923 1.808107 -1.078083
H -3.625121 -0.307446 -1.444471
C -2.764800 2.795545 -0.287442
H -4.034342 2.103130 -1.887280
C 1.173711 -3.585278 -0.970798
H 1.253450 -4.650668 -1.224179
H 1.275200 -3.035791 -1.914262
C 2.316453 -3.234217 -0.000849
H 3.257489 -3.592155 -0.442378
H 2.177347 -3.813862 0.922896
C 2.475782 -1.750581 0.369903
C 2.848163 -0.839024 -0.817225
H 2.052514 -0.879914 -1.569960
H -1.442076 3.142729 1.380275
C -3.047363 4.258461 -0.532094
H -3.961782 4.572991 -0.011959
H -2.231392 4.891306 -0.169137
H -3.192558 4.465800 -1.597424
H 3.258492 -1.667694 1.135923
C 3.067687 0.600357 -0.402312
C 1.993684 1.499789 -0.349503
C 4.337935 1.060612 -0.030260
C 2.183012 2.818934 0.064005
H 1.001358 1.160144 -0.637087
C 4.532376 2.379223 0.384006
H 5.185044 0.378530 -0.070629
C 3.453924 3.264249 0.432757
H 1.336188 3.500427 0.092365
H 5.527286 2.716400 0.664002
H 3.603851 4.292621 0.751116
H -0.976469 -3.882775 -1.048794
H 1.556009 -1.374762 0.835074
H 3.758097 -1.228911 -1.294194
H -0.344186 -3.792412 0.589224
44
-1301.65214015
C -0.248490 -3.354691 -0.423718
N -0.616354 -1.946281 -0.427816
S -1.997826 -1.641435 0.523624
O -3.120459 -2.415181 -0.027997
O -1.597495 -1.798002 1.930889
C -2.292120 0.089983 0.202255
C -3.163423 0.455389 -0.825038
C -1.669476 1.052105 0.998776
C -3.398510 1.807678 -1.061519
H -3.658524 -0.309812 -1.413222
C -1.918348 2.399841 0.746541
H -1.016968 0.747746 1.810033
C -2.778353 2.798923 -0.286971
H -1.443793 3.153501 1.369913
C 1.159790 -3.583314 -0.984031
H 1.234957 -4.647876 -1.242263
H 1.258739 -3.030161 -1.925628
C 2.308156 -3.240137 -0.017929
H 3.246027 -3.598384 -0.465897
H 2.172014 -3.824073 0.903552
C 2.473430 -1.758901 0.359692
C 2.841267 -0.841897 -0.824683
H 2.040126 -0.875085 -1.571958
H -4.081197 2.098467 -1.856040
C -3.019541 4.262182 -0.570655
H -4.033480 4.435994 -0.945477
H -2.877508 4.873592 0.326100
H -2.322614 4.633282 -1.333775
H 3.260869 -1.682207 1.121498
C 3.069780 0.594005 -0.402619
C 1.999466 1.496754 -0.333576
C 4.345064 1.047434 -0.039595
C 2.197201 2.812343 0.087243
H 1.003334 1.162383 -0.614129
C 4.547981 2.362536 0.381747
H 5.189378 0.362761 -0.092567
C 3.473102 3.250817 0.446922
H 1.353094 3.496563 0.128760
H 5.546666 2.694407 0.654544
H 3.629514 4.276405 0.771068
H -0.991594 -3.874579 -1.052572
H 1.557431 -1.383251 0.832391
H 3.746220 -1.232627 -1.310352
H -0.351229 -3.790948 0.582565
44
-1301.65208305
C 0.263368 -3.425370 -0.489566
N 0.826276 -2.089956 -0.627026
S 1.596603 -1.542225 0.785882
O 0.537178 -1.267859 1.772757
O 2.694647 -2.462245 1.118402
C 2.292247 0.007298 0.236733
C 3.655779 0.076084 -0.044726
C 1.469438 1.130271 0.115474
C 4.198231 1.290403 -0.463866
H 4.276992 -0.804831 0.075603
C 2.029742 2.331097 -0.308507
H 0.413028 1.070370 0.356250
C 3.397853 2.430804 -0.607839
H 1.394319 3.208171 -0.403459
C -0.994744 -3.596089 -1.358678
H -0.789532 -3.185939 -2.355593
H -1.157567 -4.674282 -1.485608
C -2.269087 -2.967147 -0.770286
H -2.438668 -3.382328 0.234332
H -3.125236 -3.290362 -1.379494
C -2.253035 -1.436465 -0.687938
C -3.520819 -0.850655 -0.035127
H -3.664511 -1.329425 0.943423
H 5.262030 1.352189 -0.678786
C 3.984399 3.736543 -1.088390
H 5.074180 3.747799 -0.989814
H 3.746687 3.907726 -2.146474
H 3.582454 4.586564 -0.526186
H -2.127472 -1.009449 -1.692231
C -3.440026 0.651024 0.145154
C -4.149524 1.524639 -0.687394
C -2.619489 1.201799 1.142716
C -4.046423 2.909203 -0.533225
H -4.793244 1.115931 -1.463648

C -2.513678 2.584119 1.300495
H -2.059883 0.539530 1.800488
C -3.227751 3.443976 0.461635
H -4.609546 3.568523 -1.189068
H -1.880198 2.989851 2.085508
H -3.150176 4.520829 0.587083
H 0.062325 -3.707154 0.553844
H -1.385498 -1.108840 -0.105967
H -4.400614 -1.106803 -0.639982
H 1.047053 -4.111155 -0.854402
44
-1301.65097493
C -0.263095 -3.425044 -0.489057
N -0.826043 -2.089629 -0.626609
S -1.596467 -1.541869 0.786211
O -2.694106 -2.462203 1.119195
O -0.537020 -1.266804 1.772852
C -2.292778 0.007225 0.236690
C -3.656413 0.075614 -0.043840
C -1.470116 1.130280 0.113942
C -4.199348 1.289654 -0.463535
H -4.277420 -0.805333 0.077325
C -2.030825 2.330630 -0.310498
H -0.413477 1.070564 0.353771
C -3.399320 2.429996 -0.608744
H -1.395532 3.207673 -0.406812
C 0.994634 -3.595960 -1.358641
H 1.157371 -4.674169 -1.485541
H 0.789056 -3.185899 -2.355517
C 2.269266 -2.967040 -0.770827
H 3.125096 -3.290122 -1.380548
H 2.439402 -3.382372 0.233635
C 2.253192 -1.436361 -0.688229
C 3.521284 -0.850620 -0.035993
H 4.400743 -1.106532 -0.641432
H -5.263244 1.351080 -0.678003
C -3.986138 3.736100 -1.088003
H -5.078092 3.735801 -1.017372
H -3.607326 4.582530 -0.504472
H -3.722426 3.923648 -2.137035
H 1.385924 -1.108865 -0.105788
C 3.440577 0.651014 0.144800
C 2.619157 1.201540 1.141780
C 4.151230 1.524827 -0.686549
C 2.513623 2.583811 1.300137
H 2.058658 0.539143 1.798663
C 4.048397 2.909350 -0.531795
H 4.795626 1.116310 -1.462340
C 3.228843 3.443874 0.462465
H 1.879435 2.989346 2.084681
H 4.612411 3.568820 -1.186722
H 3.151453 4.520687 0.588369
H -1.046983 -4.110923 -0.853277
H 2.127108 -1.009195 -1.692393
H 3.665621 -1.329673 0.942336
H -0.061642 -3.706499 0.554369
44
-1301.64866334
C -0.259956 -3.427263 -0.484185
N -0.824582 -2.092913 -0.625152
S -1.597056 -1.543112 0.785768
O -2.695586 -2.462882 1.117438
O -0.539567 -1.267142 1.774224
C -2.292118 0.005839 0.234376
C -1.469585 1.129070 0.114971
C -3.653516 0.071884 -0.058406
C -2.028260 2.328161 -0.316355
H -0.413414 1.069301 0.356823
C -4.194292 1.284281 -0.484796
H -4.273300 -0.811173 0.053316
C -3.395195 2.426705 -0.620762
H -5.255251 1.342507 -0.714386
C 0.998385 -3.598484 -1.352820
H 1.162412 -4.676742 -1.477635
H 0.792803 -3.190515 -2.350550
C 2.272082 -2.967074 -0.765668
H 3.128340 -3.289515 -1.375136
H 2.442905 -3.381155 0.239196
C 2.253830 -1.436361 -0.684582
C 3.521885 -0.848115 -0.034531
H 4.400966 -1.103703 -0.640652
H -1.391781 3.203928 -0.416124
C -3.990455 3.743227 -1.059383
H -4.162925 4.402521 -0.198547
H -3.322037 4.275562 -1.744793
H -4.951972 3.601428 -1.562350
H 1.386799 -1.109568 -0.101364
C 3.439465 0.653662 0.144286
C 4.147791 1.527228 -0.689304
C 2.618731 1.204539 1.141629
C 4.043340 2.911831 -0.536385
H 4.791621 1.118459 -1.465429
C 2.511576 2.586891 1.298166
H 2.060066 0.542335 1.800269
C 3.224474 3.446697 0.458256
H 4.605556 3.571105 -1.193051
H 1.877955 2.992718 2.083015
H 3.145846 4.523584 0.582742
H -1.042755 -4.115024 -0.847187
H 2.125881 -1.010368 -1.689004
H 3.667984 -1.325663 0.944274
H -0.058586 -3.705956 0.559998
44
-1301.64848689
C 0.021260 -3.313333 -0.782842
N 0.530540 -1.954829 -0.744556
S 1.273313 -1.533017 0.728810
O 0.185159 -1.044296 1.597972
O 2.159455 -2.607036 1.202328
C 2.280504 -0.152381 0.215718
C 1.672381 1.057417 -0.133733
C 3.666689 -0.297819 0.193689
C 2.475185 2.125450 -0.519037
H 0.592973 1.163624 -0.098030
C 4.453639 0.787391 -0.191505
H 4.114089 -1.242755 0.482142
C 3.874655 2.008825 -0.556968
H 5.535419 0.682093 -0.205324
C -1.454984 -3.434114 -0.293059
H -1.657242 -4.512689 -0.265886
H -1.514642 -3.076930 0.739806
C -2.504420 -2.752622 -1.185381
H -3.488682 -3.163854 -0.917096
H -2.326835 -3.055789 -2.227035
C -2.576465 -1.217823 -1.113615
C -3.122924 -0.679283 0.225286
H -2.501202 -1.042308 1.050065
H 2.008882 3.069211 -0.791066
C 4.727706 3.174599 -0.995940
H 4.656315 3.327274 -2.080644
H 4.406140 4.106811 -0.518083
H 5.782472 3.014437 -0.753154
H -3.222697 -0.860060 -1.926517
C -3.170691 0.834206 0.274863
C -4.171574 1.546153 -0.401433
C -2.201226 1.559826 0.982055
C -4.201884 2.940866 -0.379660
H -4.939781 1.000595 -0.946371
C -2.228854 2.956406 1.006818
H -1.427015 1.020273 1.522334
C -3.228052 3.652273 0.324618
H -4.989755 3.472540 -0.907621
H -1.472955 3.499998 1.568650
H -3.253132 4.738721 0.346829
H 0.644558 -3.994893 -0.189957
H -1.583191 -0.792197 -1.299988
H -4.132319 -1.086416 0.381514
H 0.059248 -3.621174 -1.835275
44
-1301.64839666

C -0.058214 -3.227835 -0.905003
N 0.433082 -1.872649 -0.715250
S 1.510023 -1.727947 0.600404
O 0.668086 -1.592249 1.803358
O 2.538279 -2.777378 0.529488
C 2.286947 -0.157249 0.262498
C 1.714113 1.014346 0.760165
C 3.465891 -0.125403 -0.484329
C 2.330746 2.233825 0.489315
H 0.807403 0.968336 1.353387
C 4.066694 1.103927 -0.744156
H 3.905718 -1.049474 -0.844035
C 3.512145 2.299191 -0.263638
H 4.984269 1.135752 -1.326330
C -1.407179 -3.486070 -0.162066
H -1.513541 -4.577499 -0.115600
H -1.309883 -3.137621 0.870485
C -2.655990 -2.906331 -0.842427
H -3.532724 -3.268615 -0.285781
H -2.737542 -3.342968 -1.848243
C -2.742449 -1.376808 -0.965122
C -2.799678 -0.632136 0.383862
H -1.881112 -0.829555 0.950168
H 1.888395 3.149513 0.873491
C 4.189750 3.623205 -0.523279
H 4.736336 3.614913 -1.471827
H 3.466865 4.444794 -0.552252
H 4.914370 3.854090 0.268767
H -3.643103 -1.127503 -1.541945
C -2.979094 0.863505 0.218611
C -1.947352 1.652576 -0.313128
C -4.178781 1.494398 0.570539
C -2.110689 3.027007 -0.485191
H -1.008340 1.182325 -0.595966
C -4.347073 2.870544 0.401209
H -4.990210 0.900521 0.986214
C -3.312721 3.642668 -0.127894
H -1.297584 3.618765 -0.898931
H -5.286828 3.338048 0.684560
H -3.440685 4.713852 -0.260319
H 0.677684 -3.973969 -0.581341
H -1.890528 -1.003273 -1.546030
H -3.628137 -1.033858 0.982689
H -0.226080 -3.344036 -1.983444
44
-1301.64802272
C 0.698766 -3.258649 -0.956281
N 0.994000 -1.845163 -0.794852
S 1.970534 -1.497813 0.555347
O 1.091608 -1.540221 1.737840
O 3.191092 -2.318280 0.512393
C 2.414786 0.200157 0.231047
C 1.478454 1.211572 0.463814
C 3.696269 0.493917 -0.230891
C 1.838219 2.531548 0.214452
H 0.490933 0.967316 0.840848
C 4.038911 1.824727 -0.470600
H 4.410565 -0.307148 -0.387484
C 3.119885 2.859457 -0.257713
H 5.038649 2.061388 -0.825691
C -0.544890 -3.733064 -0.144543
H -0.582731 -4.821078 -0.286269
H -0.359319 -3.552322 0.919039
C -1.889823 -3.125592 -0.566248
H -2.682139 -3.707878 -0.073184
H -2.035917 -3.274841 -1.646677
C -2.079123 -1.642883 -0.220034
C -3.493549 -1.127430 -0.555554
H -4.233237 -1.722058 -0.002326
H 1.114389 3.322698 0.394146
C 3.487507 4.297662 -0.534284
H 4.558516 4.408206 -0.728589
H 2.948854 4.680548 -1.410558
H 3.227417 4.945167 0.310994
H -1.338336 -1.039444 -0.758402
C -3.674939 0.340418 -0.231548
C -3.403125 1.327798 -1.188484
C -4.082309 0.750006 1.045659
C -3.533253 2.682936 -0.880935
H -3.090369 1.029643 -2.187209
C -4.213731 2.103403 1.359226
H -4.302529 -0.001575 1.801123
C -3.939298 3.075839 0.395676
H -3.323137 3.431547 -1.640861
H -4.535440 2.398092 2.354947
H -4.046402 4.130352 0.636173
H 1.557589 -3.881139 -0.672254
H -1.879246 -1.487659 0.848541
H -3.693461 -1.296409 -1.622326
H 0.494670 -3.405176 -2.023994
44
-1301.64801046
C -0.017419 -3.314365 -0.780175
N -0.527757 -1.956263 -0.743953
S -1.272831 -1.533565 0.728111
O -2.158455 -2.607968 1.201710
O -0.186261 -1.042711 1.598041
C -2.280835 -0.154250 0.213192
C -3.666614 -0.302764 0.184395
C -1.673477 1.055718 -0.136816
C -4.453949 0.780105 -0.206398
H -4.113177 -1.249737 0.467407
C -2.476674 2.121437 -0.527787
H -0.594082 1.162338 -0.102035
C -3.875927 2.003361 -0.567397
H -2.010704 3.063711 -0.805478
C 1.458287 -3.433571 -0.288322
H 1.516406 -3.074905 0.744117
H 1.661138 -4.511990 -0.259370
C 2.508413 -2.752681 -1.180253
H 2.332432 -3.057524 -2.221688
H 3.492620 -3.162806 -0.910062
C 2.579225 -1.217730 -1.110676
C 3.123535 -0.676772 0.228118
H 4.133051 -1.082866 0.386252
H -5.535184 0.670934 -0.230206
C -4.731739 3.179704 -0.971407
H -4.773460 3.928877 -0.170132
H -4.328976 3.680035 -1.859164
H -5.758885 2.872791 -1.190400
H 1.585870 -0.793130 -1.298969
C 3.170044 0.836820 0.275471
C 2.198962 1.562760 0.980100
C 4.171375 1.548516 -0.400429
C 2.225460 2.959398 1.002751
H 1.424337 1.023435 1.520012
C 4.200563 2.943283 -0.380756
H 4.940805 1.002726 -0.943403
C 3.225128 3.655005 0.320981
H 1.468285 3.503258 1.562603
H 4.988794 3.474760 -0.908375
H 3.249329 4.741505 0.341570
H -0.053763 -3.623369 -1.832337
H 3.226236 -0.860672 -1.923269
H 2.501064 -1.039067 1.052663
H -0.640965 -3.995822 -0.187421
44
-1301.64797293
C -0.698251 -3.258625 -0.956397
N -0.993644 -1.845179 -0.794932
S -1.970169 -1.497961 0.555319
O -3.190544 -2.318711 0.512527
O -1.091151 -1.540070 1.737747
C -2.414835 0.199882 0.230924
C -1.478571 1.211508 0.463137
C -3.696527 0.493336 -0.230580
C -1.838651 2.531361 0.213685
H -0.490822 0.967483 0.839724
C -4.039493 1.824062 -0.470387
H -4.410709 -0.307889 -0.386869

C -3.120602 2.858980 -0.257964
H -5.039374 2.060485 -0.825228
C 0.545332 -3.732995 -0.144526
H 0.359646 -3.552280 0.919041
H 0.583247 -4.821005 -0.286263
C 1.890265 -3.125426 -0.566087
H 2.036503 -3.274701 -1.646492
H 2.682577 -3.707615 -0.072903
C 2.079372 -1.642683 -0.219907
C 3.493783 -1.127099 -0.555280
H 3.693874 -1.296167 -1.622005
H -1.114844 3.322654 0.392857
C -3.488719 4.297187 -0.533872
H -2.943449 4.683559 -1.404465
H -4.558297 4.406180 -0.736696
H -3.237027 4.942891 0.315368
H 1.879357 -1.487444 0.848639
C 3.674968 0.340799 -0.231397
C 4.081861 0.750566 1.045913
C 3.403455 1.328045 -1.188546
C 4.213107 2.104000 1.359357
H 4.301845 -0.000917 1.801543
C 3.533410 2.683234 -0.881117
H 3.091075 1.029764 -2.187350
C 3.938975 3.076310 0.395586
H 4.534447 2.398836 2.355154
H 3.323527 3.431733 -1.641217
H 4.045952 4.130854 0.636002
H -0.494008 -3.405081 -2.024091
H 1.338580 -1.039347 -0.758383
H 4.233459 -1.721589 -0.001884
H -1.557066 -3.881189 -0.672507

44
-1301.65107136
C -0.994802 -3.596031 -1.358577
C -2.269411 -2.966984 -0.770847
C -2.253205 -1.436311 -0.688289
C -3.521284 -0.850439 -0.036149
C 0.262908 -3.425123 -0.488969
N 0.825951 -2.089761 -0.626624
H -0.789170 -3.186072 -2.355483
H -1.157609 -4.674241 -1.485391
H -2.439636 -3.382267 0.233619
H -3.125240 -3.290011 -1.380601
H -2.127018 -1.009189 -1.692460
H -1.385935 -1.108883 -0.105814
H -3.665777 -1.329518 0.942145
H -4.400712 -1.106221 -0.641687
H 1.046761 -4.111079 -0.853124
H 0.061399 -3.706491 0.554469
S 1.596341 -1.541931 0.786227
O 2.693983 -2.462259 1.119219
O 0.536887 -1.266853 1.772851
C 2.292653 0.007164 0.236693
C 3.656271 0.075484 -0.044049
C 1.470074 1.130278 0.114217
C 4.199226 1.289510 -0.463679
H 4.277226 -0.805521 0.076969
C 2.030818 2.330657 -0.310171
H 0.413472 1.070637 0.354225
C 3.399240 2.429943 -0.608660
H 5.263102 1.350902 -0.678269
H 1.395580 3.207764 -0.406229
C 3.986123 3.735920 -1.088186
H 5.077775 3.737402 -1.012953
H 3.726671 3.920742 -2.138778
H 3.603540 4.583036 -0.508164
C -3.440424 0.651178 0.144720
C -4.150994 1.525107 -0.686573
C -2.618938 1.201569 1.141724
C -4.048020 2.909613 -0.531744
H -4.795434 1.116701 -1.462386
C -2.513267 2.583818 1.300160
H -2.058510 0.539077 1.798572
C -3.228408 3.444000 0.462537
H -4.611974 3.569174 -1.186632
H -1.879031 2.989251 2.084717
H -3.150909 4.520798 0.588503
44
-1301.65087931
C 0.998374 3.598264 -1.353332
C 2.272105 2.967055 -0.766031
C 2.253947 1.436328 -0.684815
C 3.521883 0.848163 -0.034477
C -0.259994 3.427018 -0.484755
N -0.824160 2.092403 -0.625270
H 0.792907 3.190156 -2.351028
H 1.162277 4.676529 -1.478271
H 2.442836 3.381229 0.238808
H 3.128357 3.289537 -1.375485
H 2.126243 1.010249 -1.689230
H 1.386783 1.109564 -0.101783
H 3.667842 1.325848 0.944290
H 4.401079 1.103660 -0.640470
H -1.042979 4.114399 -0.848061
H -0.058819 3.706118 0.559351
S -1.596842 1.543011 0.785737
O -2.695020 2.463097 1.117637
O -0.539251 1.266703 1.774010
C -2.292409 -0.005698 0.234317
C -3.654093 -0.071917 -0.056025
C -1.469753 -1.128953 0.113264
C -4.195430 -1.284517 -0.481933
H -4.273998 0.810832 0.057437
C -2.028816 -2.327815 -0.317420
H -0.413315 -1.069115 0.353933
C -3.396427 -2.426435 -0.619989
H -5.256838 -1.343030 -0.709189
H -1.392407 -3.203589 -0.418009
C -3.990047 -3.742649 -1.061797
H -3.353466 -4.238907 -1.802782
H -4.094872 -4.431401 -0.213422
H -4.982758 -3.607882 -1.501891
C 3.439459 -0.653604 0.144528
C 2.617373 -1.204437 1.140792
C 4.149254 -1.527212 -0.687770
C 2.510326 -2.586775 1.297501
H 2.057572 -0.542229 1.798464
C 4.044920 -2.911810 -0.534663
H 4.794144 -1.118481 -1.463035
C 3.224688 -3.446627 0.458874
H 1.875608 -2.992562 2.081485
H 4.608296 -3.571101 -1.190318
H 3.146107 -4.523501 0.583503
44
-1301.65066652
C -0.999365 -3.598401 -1.352762
C -2.272812 -2.966739 -0.765330
C -2.254373 -1.435987 -0.684736
C -3.521913 -0.847390 -0.033994
C 0.259195 -3.427366 -0.484397
N 0.823832 -2.093020 -0.625277
H -0.793946 -3.190522 -2.350562
H -1.163585 -4.676642 -1.477474
H -2.443287 -3.380490 0.239727
H -3.129318 -3.289306 -1.374378
H -2.127063 -1.010324 -1.689379
H -1.386906 -1.109095 -0.102234
H -3.667501 -1.324762 0.944971
H -4.401425 -1.102884 -0.639531
H 1.041871 -4.115112 -0.847701
H 0.058055 -3.706242 0.559782
S 1.596472 -1.543406 0.785680
O 2.694774 -2.463408 1.117451
O 0.538956 -1.267188 1.774040
C 2.291909 0.005372 0.234268

C 1.469606 1.128736 0.114600
C 3.653396 0.071153 -0.058215
C 2.028599 2.327704 -0.316690
H 0.413363 1.069187 0.356193
C 4.194484 1.283418 -0.484558
H 4.272980 -0.812024 0.053668
C 3.395621 2.425993 -0.620752
H 1.392291 3.203566 -0.416703
H 5.255502 1.341432 -0.713931
C 3.991314 3.742411 -1.059100
H 3.322207 4.276078 -1.742783
H 4.165919 4.400646 -0.197870
H 4.951848 3.600247 -1.563845
C -3.439040 0.654394 0.144554
C -4.147394 1.528023 -0.688937
C -2.617786 1.205205 1.141521
C -4.042467 2.912632 -0.536305
H -4.791631 1.119319 -1.464761
C -2.510151 2.587547 1.297765
H -2.059109 0.542938 1.800087
C -3.223083 3.447426 0.457940
H -4.604727 3.571947 -1.192892
H -1.876131 2.993338 2.082312
H -3.144059 4.524310 0.582205
44
-1301.65041682
C 1.274303 2.158172 -1.371622
C 2.238503 1.572149 -0.327175
C 1.830896 0.209901 0.247467
C 2.874341 -0.386407 1.219976
C -0.116617 2.528386 -0.838313
N -0.969631 1.356129 -0.708481
H 1.152161 1.460956 -2.210392
H 1.719496 3.073075 -1.784177
H 2.353701 2.292354 0.497092
H 3.228295 1.483880 -0.793147
H 1.655905 -0.496822 -0.575143
H 0.878221 0.301686 0.783046
H 2.439954 -1.283182 1.681282
H 3.050169 0.325648 2.036835
H -0.624694 3.190240 -1.560933
H -0.043626 3.100920 0.098652
S -2.239915 1.591773 0.397601
O -3.045961 2.741800 -0.039038
O -1.642524 1.565768 1.743337
C -3.202348 0.105385 0.169013
C -4.272049 0.115596 -0.726487
C -2.878512 -1.041824 0.895124
C -5.017393 -1.048587 -0.900855
H -4.517319 1.023515 -1.266995
C -3.635119 -2.195442 0.706625
H -2.054937 -1.023360 1.600870
C -4.713257 -2.218361 -0.190629
H -5.851690 -1.047434 -1.597859
H -3.387621 -3.092689 1.268520
C -5.546491 -3.465832 -0.362309
H -4.944805 -4.371096 -0.230559
H -6.355504 -3.502011 0.379263
H -6.010420 -3.505207 -1.352997
C 4.195376 -0.749795 0.571733
C 4.296381 -1.880011 -0.253625
C 5.342902 0.030351 0.765108
C 5.501701 -2.217242 -0.868438
H 3.419748 -2.505587 -0.410456
C 6.553098 -0.302247 0.152126
H 5.288341 0.906291 1.408019
C 6.636487 -1.427506 -0.668090
H 5.557287 -3.100033 -1.500511
H 7.430587 0.317358 0.319243
H 7.577460 -1.690438 -1.144062
44
-1301.64917291
C -0.540469 3.736657 -0.138496
C -1.885051 3.129733 -0.562159
C -2.074521 1.646651 -0.217617
C -3.488493 1.131077 -0.554837
C 0.703793 3.264240 -0.950438
N 0.997415 1.849832 -0.793981
H -0.355404 3.553668 0.924784
H -0.578411 4.824962 -0.277975
H -2.030179 3.280105 -1.642558
H -2.677827 3.711462 -0.069178
H -1.875583 1.490419 0.850995
H -1.333163 1.043916 -0.755978
H -3.687563 1.301042 -1.621609
H -4.228899 1.724800 -0.001610
H 1.562774 3.885036 -0.663181
H 0.501091 3.414873 -2.017849
S 1.973740 1.496602 0.554727
O 1.097116 1.542247 1.738794
O 3.198039 2.311408 0.510165
C 2.409698 -0.203308 0.229405
C 3.686792 -0.502265 -0.242408
C 1.469804 -1.210257 0.464769
C 4.021245 -1.833752 -0.487433
H 4.402238 0.296232 -0.406698
C 1.821520 -2.531754 0.209782
H 0.483939 -0.961687 0.843305
C 3.099534 -2.865128 -0.267073
H 5.015810 -2.074023 -0.854633
H 1.092955 -3.319161 0.386391
C 3.474205 -4.307396 -0.510904
H 4.358359 -4.390068 -1.150393
H 3.700886 -4.819407 0.433450
H 2.655755 -4.857887 -0.987674
C -3.669354 -0.337191 -0.232430
C -3.393932 -1.323539 -1.189421
C -4.079596 -0.748215 1.043386
C -3.523340 -2.679048 -0.883247
H -3.078880 -1.024275 -2.187091
C -4.210329 -2.102011 1.355576
H -4.302625 0.002537 1.798850
C -3.932283 -3.073391 0.392011
H -3.310430 -3.426832 -1.643210
H -4.534315 -2.397817 2.350227
H -4.038852 -4.128209 0.631411
44
-1301.64916312
C 0.537992 -3.737665 -0.138038
C 1.882646 -3.131187 -0.562135
C 2.072669 -1.648188 -0.217545
C 3.486613 -1.132875 -0.555275
C -0.706343 -3.265096 -0.949770
N -0.999175 -1.850420 -0.794118
H 0.353223 -3.554393 0.925242
H 0.575588 -4.826009 -0.277307
H 2.027378 -3.281548 -1.642587
H 2.675395 -3.713185 -0.069432
H 1.874219 -1.492016 0.851169
H 1.331243 -1.045219 -0.755551
H 3.685204 -1.302729 -1.622155
H 4.227137 -1.726804 -0.002433
H -1.565528 -3.885299 -0.661834
H -0.504128 -3.416493 -2.017166
S -1.975176 -1.495874 0.554472
O -1.099064 -1.543097 1.738849
O -3.200679 -2.308838 0.509284
C -2.408406 0.204782 0.229368
C -1.466970 1.210207 0.465093
C -3.684928 0.505833 -0.242640
C -1.816542 2.532297 0.210248
H -0.481569 0.960043 0.843784
C -4.017234 1.837895 -0.487499
H -4.401595 -0.291509 -0.407218
C -3.093937 2.867772 -0.266800
H -1.086748 3.318509 0.387117
H -5.011351 2.079798 -0.854839
C -3.466229 4.310672 -0.510544
H -2.647132 4.859645 -0.987964
H -4.350670 4.394830 -1.149439

H -3.691327 4.823216 0.433896
C 3.667846 0.335316 -0.232716
C 4.079676 0.746028 1.042690
C 3.391154 1.321900 -1.189098
C 4.210724 2.099748 1.355075
H 4.303698 -0.004917 1.797668
C 3.520867 2.677336 -0.882724
H 3.074856 1.022893 -2.186449
C 3.931399 3.071367 0.392120
H 4.535946 2.395314 2.349393
H 3.306958 3.425304 -1.642225
H 4.038214 4.126127 0.631667
44
-1301.64910916
C -1.204147 2.806477 1.228187
C -2.313131 1.758573 1.418891
C -1.820793 0.349886 1.771730
C -2.958376 -0.678802 1.935125
C -0.289368 2.557015 0.019009
N 0.740651 1.574030 0.320952
H -0.580201 2.878172 2.128342
H -1.670973 3.790031 1.087272
H -2.921042 1.712929 0.505281
H -2.986598 2.112471 2.212776
H -1.244346 0.387841 2.706281
H -1.124988 -0.004574 1.003098
H -3.618350 -0.362251 2.753832
H -2.513289 -1.631590 2.256169
H 0.254379 3.485533 -0.227753
H -0.874954 2.302947 -0.876894
S 1.310461 0.761354 -1.059928
O 1.696392 1.734377 -2.092046
O 0.313185 -0.281966 -1.358789
C 2.789674 -0.017903 -0.434108
C 4.028698 0.568421 -0.690411
C 2.688252 -1.206487 0.292200
C 5.180359 -0.045584 -0.200637
H 4.084038 1.483607 -1.269931
C 3.849332 -1.803027 0.774283
H 1.717019 -1.657067 0.466541
C 5.110845 -1.235279 0.536232
H 6.149291 0.406408 -0.397040
H 3.777248 -2.727613 1.341753
C 6.364209 -1.910452 1.039156
H 6.674562 -2.716317 0.361069
H 7.197871 -1.205303 1.113187
H 6.208506 -2.359980 2.025681
C -3.794916 -0.914069 0.689427
C -5.189968 -0.793962 0.733404
C -3.198837 -1.272634 -0.530284
C -5.972494 -1.029752 -0.399053
H -5.670174 -0.514402 1.669025
C -3.976898 -1.505345 -1.664464
H -2.117680 -1.359896 -0.603570
C -5.367462 -1.387224 -1.603824
H -7.053540 -0.931029 -0.338205
H -3.493142 -1.778418 -2.598813
H -5.972069 -1.569771 -2.488430
44
-1301.64712386
C -1.232876 -2.789304 0.280176
C -2.378316 -1.905863 -0.231037
C -1.955998 -0.743973 -1.138586
C -3.146212 0.047728 -1.724377
C -0.277874 -2.120958 1.317627
N 0.547981 -1.065920 0.758190
H -0.636421 -3.171738 -0.554808
H -1.657088 -3.659625 0.797408
H -2.951025 -1.520575 0.623565
H -3.069331 -2.555779 -0.788592
H -1.350199 -1.134425 -1.966963
H -1.304105 -0.060632 -0.580308
H -3.787935 -0.636426 -2.295301
H -2.752151 0.775393 -2.446433
H 0.343295 -2.905389 1.769660
H -0.885332 -1.650868 2.100128
S 1.962366 -1.632400 -0.007047
O 1.578225 -1.953597 -1.393371
O 2.634014 -2.639737 0.828942
C 2.963769 -0.154460 -0.018376
C 2.756756 0.807572 -1.009437
C 3.946248 0.013537 0.957265
C 3.543010 1.955821 -1.009098
H 1.998970 0.649912 -1.769484
C 4.724426 1.169777 0.939808
H 4.099344 -0.754225 1.708052
C 4.537234 2.155427 -0.038569
H 3.386572 2.709267 -1.777208
H 5.491923 1.306791 1.697402
C 5.404146 3.391248 -0.069325
H 5.832202 3.605874 0.914832
H 6.239826 3.266607 -0.770587
H 4.837369 4.270171 -0.394380
C -3.981570 0.774866 -0.689085
C -5.262841 0.328030 -0.341100
C -3.481610 1.915707 -0.042987
C -6.022538 0.994726 0.622931
H -5.672088 -0.550649 -0.835184
C -4.235118 2.585457 0.920379
H -2.491246 2.284585 -0.302318
C -5.510616 2.126280 1.258005
H -7.015397 0.630132 0.874301
H -3.828597 3.469509 1.405270
H -6.100334 2.648887 2.006516
44
-1301.64707133
C -1.395290 -3.489386 -0.169447
C -2.643886 -2.911467 -0.851724
C -2.734658 -1.381866 -0.970333
C -2.798388 -0.641302 0.380647
C -0.045213 -3.226656 -0.908779
N 0.441907 -1.870350 -0.716490
H -1.499070 -4.581157 -0.125183
H -1.301264 -3.142527 0.863947
H -3.521099 -3.277888 -0.298552
H -2.721318 -3.345568 -1.858966
H -3.634335 -1.133708 -1.549177
H -1.882124 -1.003970 -1.547519
H -1.880593 -0.836736 0.948865
H -3.626941 -1.047940 0.976023
H -0.210142 -3.342248 -1.987737
H 0.691906 -3.971103 -0.584012
S 1.515058 -1.723898 0.602049
O 2.545838 -2.770988 0.533441
O 0.669616 -1.590582 1.802798
C 2.288696 -0.151173 0.265664
C 1.714377 1.018533 0.765918
C 3.471546 -0.116737 -0.474924
C 2.331828 2.239081 0.501462
H 0.809912 0.969576 1.362331
C 4.073128 1.113519 -0.728276
H 3.917505 -1.040224 -0.828520
C 3.512727 2.307798 -0.251905
H 1.892396 3.152551 0.894095
H 4.997752 1.146583 -1.299100
C 4.157555 3.638305 -0.557643
H 5.244219 3.545034 -0.652839
H 3.781366 4.047104 -1.504867
H 3.944844 4.375940 0.222689
C -2.983172 0.854063 0.219047
C -1.951692 1.649126 -0.304175
C -4.187619 1.478752 0.565738
C -2.119840 3.023374 -0.472990
H -1.009037 1.183659 -0.582813
C -4.360784 2.854677 0.399566
H -4.998929 0.880167 0.974846
C -3.326600 3.632810 -0.120993
H -1.306777 3.619938 -0.879855
H -5.304176 3.317323 0.678778
H -3.458301 4.703859 -0.250819

44
-1301.64703138
C -1.210817 2.395895 -0.250626
C -2.180163 1.371795 -0.854812
C -1.811603 -0.098199 -0.619745
C -2.862288 -1.093910 -1.160052
C 0.183802 2.499426 -0.942409
N 1.010317 1.317707 -0.774738
H -1.063357 2.202159 0.817046
H -1.649229 3.398530 -0.337404
H -2.278647 1.558209 -1.935293
H -3.172005 1.562957 -0.423982
H -1.665249 -0.267412 0.455328
H -0.847494 -0.314192 -1.096339
H -2.454318 -2.108916 -1.062745
H -3.002822 -0.920603 -2.235282
H 0.693820 3.393081 -0.559300
H 0.029640 2.612829 -2.022303
S 1.887708 1.293826 0.686810
O 0.985593 0.715159 1.698499
O 2.529773 2.596265 0.923450
C 3.160590 0.101667 0.305006
C 4.427678 0.549848 -0.067491
C 2.873197 -1.262220 0.394245
C 5.415857 -0.387914 -0.362576
H 4.632074 1.614037 -0.115289
C 3.872634 -2.182810 0.094114
H 1.886159 -1.590356 0.702217
C 5.156680 -1.762933 -0.287187
H 6.405637 -0.045102 -0.653401
H 3.655376 -3.246086 0.160337
C 6.239974 -2.773226 -0.579620
H 5.837780 -3.655665 -1.088600
H 7.027929 -2.346393 -1.207885
H 6.712331 -3.121437 0.348415
C -4.205218 -1.027988 -0.459679
C -5.329473 -0.476430 -1.087601
C -4.350676 -1.513294 0.848835
C -6.560879 -0.407258 -0.431878
H -5.239911 -0.100797 -2.104783
C -5.577265 -1.446905 1.508667
H -3.492297 -1.951919 1.353751
C -6.688872 -0.892047 0.869762
H -7.419648 0.023387 -0.940750
H -5.667070 -1.832087 2.521278
H -7.646119 -0.841772 1.381881

44
-1301.65086809
C -0.994508 -3.596049 -1.358762
C -2.269188 -2.967044 -0.771120
C -2.253032 -1.436368 -0.688502
C -3.521156 -0.850537 -0.036412
C 0.263122 -3.425167 -0.489034
N 0.826181 -2.089809 -0.626631
H -2.439489 -3.382365 0.233317
H -3.124953 -3.290080 -1.380957
H -2.126802 -1.009206 -1.692648
H -1.385796 -1.108955 -0.105966
H -3.665725 -1.329695 0.941830
H 0.061529 -3.706535 0.554387
H -1.157302 -4.674250 -1.485670
H -0.788780 -3.186028 -2.355621
H -4.400546 -1.106267 -0.642029
S 1.596284 -1.541874 0.786331
O 2.694039 -2.462033 1.119430
O 0.536673 -1.266979 1.772840
C 2.292438 0.007326 0.236881
C 3.656119 0.075752 -0.044063
C 1.469906 1.130396 0.114909
C 4.198961 1.289788 -0.463425
H 4.277117 -0.805232 0.076889
C 2.030620 2.330985 -0.309296
H 0.413394 1.070783 0.355321
C 3.398829 2.430304 -0.608167
H 5.262869 1.351311 -0.677905
H 1.395431 3.208168 -0.404763
C 3.985896 3.735564 -1.089392
H 3.578205 4.586777 -0.533222
H 5.074957 3.749518 -0.983323
H 3.755317 3.902225 -2.149791
H 1.047006 -4.111124 -0.853123
C -3.440321 0.651072 0.144588
C -2.618932 1.201391 1.141713
C -4.150852 1.525068 -0.686670
C -2.513300 2.583629 1.300285
H -2.058547 0.538856 1.798555
C -4.047922 2.909561 -0.531702
H -4.795235 1.116727 -1.462566
C -3.228391 3.443875 0.462688
H -1.879137 2.988999 2.084935
H -4.611846 3.569170 -1.186565
H -3.150926 4.520663 0.588758
44
-1301.65066963
C 0.999766 3.598408 -1.352714
C 2.273171 2.966559 -0.765383
C 2.254622 1.435806 -0.684983
C 3.521969 0.847065 -0.033978
C -0.258790 3.427377 -0.484339
N -0.823487 2.093049 -0.625282
H 2.443669 3.380157 0.239734
H 3.129724 3.289114 -1.374373
H 2.127524 1.010289 -1.689716
H 1.386999 1.108898 -0.102738
H 3.667424 1.324450 0.944997
H -0.057637 3.706182 0.559853
H 1.164076 4.676648 -1.477315
H 0.794269 3.190649 -2.350545
H 4.401629 1.102438 -0.639345
S -1.596213 1.543487 0.785703
O -2.694376 2.463626 1.117513
O -0.538722 1.267117 1.774043
C -2.291869 -0.005188 0.234271
C -1.469723 -1.128662 0.114513
C -3.653391 -0.070793 -0.058082
C -2.028903 -2.327537 -0.316780
H -0.413458 -1.069264 0.356056
C -4.194668 -1.282974 -0.484435
H -4.272855 0.812457 0.053889
C -3.395961 -2.425637 -0.620758
H -1.392719 -3.203483 -0.416855
H -5.255716 -1.340853 -0.713707
C -3.991815 -3.741938 -1.059242
H -4.952747 -3.599667 -1.563201
H -3.323159 -4.275177 -1.743709
H -4.165651 -4.400615 -0.198203
H -1.041448 4.115169 -0.847596
C 3.438882 -0.654708 0.144585
C 2.617497 -1.205413 1.141489
C 4.147213 -1.528423 -0.688855
C 2.509718 -2.587752 1.297741
H 2.058804 -0.543092 1.799989
C 4.042138 -2.913011 -0.536214
H 4.791533 -1.119770 -1.464634
C 3.222627 -3.447707 0.457989
H 1.875584 -2.993469 2.082234
H 4.604371 -3.572407 -1.192744
H 3.143481 -4.524582 0.582260
44
-1301.65066181
C -0.998409 -3.598728 -1.352837
C -2.272050 -2.967214 -0.765670
C -2.253672 -1.436442 -0.684858
C -3.521361 -0.847987 -0.034372
C 0.259907 -3.427515 -0.484165
N 0.824471 -2.093145 -0.625193
H -2.442678 -3.381025 0.239342
H -3.128407 -3.289857 -1.374875

H -2.125970 -1.010528 -1.689337
H -1.386395 -1.109759 -0.101953
H -3.667153 -1.325485 0.944518
H 0.058512 -3.706192 0.560021
H -1.162489 -4.676978 -1.477655
H -0.792763 -3.190774 -2.350565
H -4.400747 -1.103502 -0.640095
S 1.596938 -1.543247 0.785754
O 2.695581 -2.462873 1.117458
O 0.539424 -1.267405 1.774243
C 2.291716 0.005817 0.234259
C 3.653143 0.072133 -0.058285
C 1.468932 1.128852 0.114625
C 4.193711 1.284626 -0.484709
H 4.273134 -0.810757 0.053586
C 2.027402 2.328021 -0.316724
H 0.412735 1.068833 0.356323
C 3.394380 2.426849 -0.620900
H 5.254698 1.343048 -0.714130
H 1.390757 3.203642 -0.416696
C 3.989414 3.743505 -1.059432
H 4.163190 4.402165 -0.198367
H 4.950259 3.601800 -1.563716
H 3.320254 4.276472 -1.743614
H 1.042707 -4.115296 -0.847124
C -3.438744 0.653798 0.144421
C -2.617474 1.204627 1.141338
C -4.147554 1.527417 -0.688728
C -2.510228 2.586981 1.297870
H -2.058538 0.542393 1.799735
C -4.043027 2.912008 -0.535797
H -4.791832 1.118708 -1.464509
C -3.223581 3.446834 0.458400
H -1.876158 2.992772 2.082378
H -4.605653 3.571305 -1.192090
H -3.144841 4.523714 0.582876
44
-1301.64853189
C 0.555744 0.262006 1.077034
C 1.609531 -0.719975 1.601828
C 3.058975 -0.251432 1.393449
C 3.505089 -0.193018 -0.083400
C -0.906436 -0.171450 1.362684
N -1.189656 -1.432155 0.706929
H 1.463698 -1.697255 1.126012
H 1.442409 -0.873784 2.677532
H 3.730454 -0.932884 1.931852
H 3.201306 0.737807 1.851847
H 2.876310 0.517511 -0.633644
H -1.007892 -0.363183 2.441299
H 0.689993 1.244986 1.550445
H 0.654547 0.405746 -0.004110
H 3.336624 -1.176349 -0.542175
S -2.187085 -1.415108 -0.663845
O -2.746935 -2.761360 -0.771969
O -1.336400 -0.849035 -1.725740
C -3.549741 -0.285440 -0.379029
C -3.498909 1.011390 -0.893851
C -4.657888 -0.726315 0.350850
C -4.571057 1.873509 -0.667433
H -2.639854 1.325844 -1.477191
C -5.716482 0.150313 0.569616
H -4.692372 -1.745228 0.722608
C -5.692208 1.460083 0.064944
H -4.537859 2.882439 -1.070934
H -6.581262 -0.188953 1.134561
C -6.863025 2.388560 0.280368
H -7.650878 2.202996 -0.461458
H -7.310519 2.246355 1.269753
H -6.566212 3.437809 0.186970
H -1.595504 0.640733 1.097182
C 4.959078 0.201178 -0.236382
C 5.972645 -0.766716 -0.209898
C 5.330484 1.545742 -0.371400
C 7.316127 -0.403950 -0.312319
H 5.703953 -1.816868 -0.113937
C 6.672500 1.914778 -0.473889
H 4.557649 2.311403 -0.402108
C 7.671104 0.939871 -0.443971
H 8.085570 -1.171706 -0.294755
H 6.937602 2.963499 -0.582515
H 8.716679 1.224265 -0.527751
44
-1301.64852830
C 0.555632 0.261357 1.077169
C 1.609430 -0.720865 1.601488
C 3.058885 -0.252278 1.393306
C 3.504798 -0.192596 -0.083539
C -0.906539 -0.172025 1.363078
N -1.189980 -1.432660 0.707271
H 1.463562 -1.697931 1.125244
H 1.442351 -0.875155 2.677130
H 3.730350 -0.934280 1.931035
H 3.201371 0.736542 1.852557
H 2.876066 0.518551 -0.633036
H -1.007777 -0.363850 2.441691
H 0.690071 1.244214 1.550786
H 0.654182 0.405335 -0.003965
H 3.336085 -1.175473 -0.543200
S -2.187050 -1.415175 -0.663799
O -2.747105 -2.761308 -0.772325
O -1.336019 -0.849035 -1.725382
C -3.549567 -0.285332 -0.379037
C -4.657903 -0.726139 0.350606
C -3.498464 1.011554 -0.893682
C -5.716400 0.150613 0.569316
H -4.692604 -1.745099 0.722215
C -4.570528 1.873800 -0.667331
H -2.639271 1.325952 -1.476848
C -5.691850 1.460449 0.064820
H -6.581326 -0.188601 1.134071
H -4.537123 2.882771 -1.070709
C -6.862546 2.389077 0.280246
H -7.650801 2.203040 -0.461032
H -7.309524 2.247511 1.269962
H -6.565768 3.438261 0.186036
H -1.595586 0.640237 1.097768
C 4.958823 0.201508 -0.236424
C 5.330358 1.546126 -0.370617
C 5.972285 -0.766506 -0.210684
C 6.672394 1.915086 -0.473037
H 4.557593 2.311881 -0.400706
C 7.315797 -0.403813 -0.313037
H 5.703509 -1.816696 -0.115365
C 7.670901 0.940049 -0.443874
H 6.937601 2.963847 -0.581023
H 8.085154 -1.171667 -0.296061
H 8.716494 1.224388 -0.527612
44
-1301.64732478
C -1.394389 3.288804 -1.377580
C -2.714232 2.982904 -0.644808
C -2.847630 1.598742 0.011738
C -2.739469 0.413564 -0.966357
C -0.129479 3.180685 -0.512246
N 0.378090 1.818877 -0.482788
H -3.534693 3.121554 -1.363288
H -2.862796 3.746681 0.131872
H -3.822310 1.554038 0.515519
H -2.095709 1.489259 0.802094
H -1.733654 0.406809 -1.406250
H 0.678749 3.786859 -0.958282
H -1.268897 2.638080 -2.251013
H -1.450277 4.315532 -1.761753
H -3.441341 0.563888 -1.798028
S 1.348917 1.478114 0.871664
O 0.404121 0.983361 1.890582
O 2.244474 2.600210 1.186376
C 2.332533 0.120427 0.259864
C 1.726434 -1.108370 -0.021032

C 3.704385 0.304374 0.093706
C 2.515429 -2.156057 -0.482615
H 0.658762 -1.243281 0.117744
C 4.478280 -0.761233 -0.365576
H 4.150853 1.265315 0.324995
C 3.900579 -2.002182 -0.660138
H 2.049658 -3.112428 -0.707244
H 5.548637 -0.624203 -0.496230
C 4.743096 -3.159435 -1.139895
H 4.277474 -3.665045 -1.993346
H 5.741852 -2.830900 -1.442639
H 4.866333 -3.910209 -0.348672
H -0.285801 3.599433 0.493489
C -3.013465 -0.934217 -0.325550
C -4.002942 -1.782988 -0.837773
C -2.279833 -1.370712 0.790315
C -4.253508 -3.031526 -0.264154
H -4.584559 -1.461970 -1.699572
C -2.528295 -2.617296 1.366507
H -1.515915 -0.727845 1.221403
C -3.515417 -3.454418 0.841276
H -5.027517 -3.671122 -0.681273
H -1.950570 -2.932875 2.231850
H -3.708794 -4.424495 1.291772
44
-1301.64719058
C -1.395705 3.288785 -1.377007
C -2.715122 2.982526 -0.643635
C -2.847926 1.598170 0.012600
C -2.739892 0.413285 -0.965881
C -0.130394 3.180874 -0.512240
N 0.377464 1.819134 -0.482993
H -3.535979 3.121224 -1.361650
H -2.863398 3.746082 0.133320
H -3.822404 1.553081 0.516727
H -2.095661 1.488669 0.802606
H -1.734284 0.406918 -1.406282
H 0.677559 3.787024 -0.958825
H -1.270450 2.638157 -2.250541
H -1.451965 4.315538 -1.761045
H -3.442156 0.563686 -1.797215
S 1.348461 1.478355 0.871289
O 0.403997 0.983480 1.890400
O 2.243970 2.600562 1.185853
C 2.332213 0.120789 0.259360
C 1.726307 -1.107750 -0.022159
C 3.704243 0.304923 0.093379
C 2.515543 -2.155333 -0.484121
H 0.658545 -1.242715 0.115887
C 4.478184 -0.760294 -0.366153
H 4.150449 1.266009 0.324559
C 3.900577 -2.001460 -0.660950
H 2.049804 -3.111443 -0.709770
H 5.548495 -0.622961 -0.497108
C 4.744827 -3.158889 -1.137186
H 4.919404 -3.877146 -0.325482
H 4.252835 -3.704519 -1.949891
H 5.722479 -2.822382 -1.495352
H -0.286212 3.599771 0.493495
C -3.013268 -0.934748 -0.325367
C -4.002385 -1.783814 -0.837797
C -2.279403 -1.371236 0.790350
C -4.252399 -3.032611 -0.264501
H -4.584150 -1.462823 -1.699506
C -2.527300 -2.618082 1.366205
H -1.515744 -0.728166 1.221583
C -3.514090 -3.455482 0.840786
H -5.026156 -3.672431 -0.681745
H -1.949385 -2.933668 2.231418
H -3.707052 -4.425764 1.291020
44
-1301.64621444
C 1.433328 2.846556 1.349590
C 2.548340 1.841440 1.698321
C 2.125200 0.472029 2.270070
C 1.809935 -0.640219 1.243935
C 0.594458 2.570147 0.095974
N -0.463156 1.602515 0.340990
H 3.179888 2.334550 2.448836
H 3.193935 1.691888 0.822201
H 1.251422 0.602334 2.922482
H 2.934936 0.111074 2.917742
H 0.954581 -0.344353 0.628754
H 0.110160 3.500966 -0.245351
H 0.753122 2.965945 2.203090
H 1.907325 3.825161 1.194713
H 1.486412 -1.527107 1.807287
S -1.332369 1.222470 -1.077091
O -0.426361 0.474622 -1.965932
O -2.000670 2.445592 -1.546302
C -2.570710 0.098427 -0.454092
C -2.308745 -1.272165 -0.440263
C -3.787490 0.607479 0.001927
C -3.281846 -2.139210 0.051278
H -1.363870 -1.648719 -0.817066
C -4.747271 -0.276025 0.489674
H -3.977661 1.674799 -0.035207
C -4.512028 -1.658271 0.522282
H -3.084032 -3.208079 0.065669
H -5.696888 0.113736 0.847693
C -5.572116 -2.609783 1.022875
H -6.228041 -2.931793 0.203251
H -5.128690 -3.511200 1.457957
H -6.206317 -2.140326 1.781772
H 1.225524 2.243001 -0.746214
C 2.972180 -1.017125 0.344535
C 2.911891 -0.806528 -1.039986
C 4.135528 -1.593472 0.877850
C 3.984145 -1.154587 -1.865893
H 2.012185 -0.379625 -1.477626
C 5.207354 -1.941862 0.056872
H 4.199993 -1.777902 1.948501
C 5.136224 -1.721121 -1.320944
H 3.914175 -0.983999 -2.937296
H 6.097445 -2.390121 0.491618
H 5.970376 -1.993322 -1.962425
44
-1301.64621011
C 1.433160 2.846749 1.349713
C 2.548291 1.841609 1.698014
C 2.125316 0.472184 2.269868
C 1.809922 -0.640138 1.243853
C 0.594090 2.570545 0.096185
N -0.463359 1.602701 0.341136
H 3.180102 2.334661 2.448344
H 3.193596 1.692097 0.821673
H 1.251659 0.602473 2.922450
H 2.935198 0.111274 2.917383
H 0.954547 -0.344307 0.628684
H 0.109543 3.501378 -0.244751
H 0.753102 2.965926 2.203360
H 1.907063 3.825416 1.194938
H 1.486418 -1.526950 1.807327
S -1.332367 1.222477 -1.077031
O -0.426117 0.474644 -1.965647
O -2.000701 2.445490 -1.546471
C -2.570671 0.098368 -0.454079
C -3.787536 0.607350 0.001805
C -2.308577 -1.272193 -0.440102
C -4.747274 -0.276200 0.489540
H -3.977790 1.674653 -0.035394
C -3.281649 -2.139292 0.051412
H -1.363610 -1.648684 -0.816740
C -4.511928 -1.658433 0.522227
H -5.696942 0.113505 0.847485
H -3.083721 -3.208139 0.065941
C -5.572045 -2.609991 1.022666
H -6.228537 -2.931105 0.203138
H -5.128701 -3.511912 1.456774
H -6.205686 -2.140916 1.782267

H 1.225058 2.243766 -0.746212
C 2.972142 -1.017158 0.344478
C 2.912071 -0.806247 -1.040008
C 4.135299 -1.593846 0.877831
C 3.984365 -1.154353 -1.865838
H 2.012486 -0.379118 -1.477674
C 5.207170 -1.942276 0.056926
H 4.199571 -1.778508 1.948457
C 5.136263 -1.721229 -1.320850
H 3.914576 -0.983547 -2.937218
H 6.097110 -2.390809 0.491699
H 5.970441 -1.993458 -1.962284
44
-1301.64603879
C -1.457998 -3.433679 -0.288452
C -2.508005 -2.752771 -1.180539
C -2.578993 -1.217833 -1.110862
C -3.123448 -0.677030 0.227940
C 0.017772 -3.314358 -0.780007
N 0.527917 -1.956127 -0.743690
H -3.492228 -3.163012 -0.910594
H -2.331759 -3.057501 -2.221963
H -3.226025 -0.860839 -1.923467
H -1.585693 -0.793084 -1.299096
H -2.500927 -1.039227 1.052483
H 0.641312 -3.995687 -0.187114
H -1.516355 -3.075109 0.744010
H -1.660840 -4.512103 -0.259623
H -4.132900 -1.083313 0.386013
S 1.272843 -1.533293 0.728329
O 2.158451 -2.607624 1.202160
O 0.186208 -1.042314 1.598120
C 2.280905 -0.154043 0.213346
C 1.673602 1.056010 -0.136389
C 3.666675 -0.302754 0.184073
C 2.476833 2.121659 -0.527556
H 0.594229 1.162754 -0.101287
C 4.454021 0.780009 -0.206907
H 4.113194 -1.249813 0.466871
C 3.876034 2.003403 -0.567605
H 2.010912 3.064006 -0.805069
H 5.535234 0.670678 -0.231119
C 4.732016 3.179676 -0.971465
H 5.757609 2.871745 -1.196290
H 4.326024 3.683910 -1.855506
H 4.778882 3.925845 -0.167656
H 0.054378 -3.623299 -1.832167
C -3.170240 0.836558 0.275349
C -4.171538 1.548110 -0.400761
C -2.199461 1.562637 0.980247
C -4.200972 2.942868 -0.381035
H -4.940748 1.002206 -0.943934
C -2.226203 2.959274 1.002949
H -1.424895 1.023411 1.520342
C -3.225824 3.654732 0.320964
H -4.989170 3.474237 -0.908815
H -1.469255 3.503245 1.562999
H -3.250212 4.741228 0.341576

C_6-rad

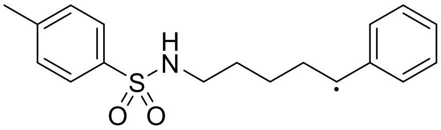

Name	E(B3LYP)	G(B3LYP)	E(RO-B2PLYP-D3)	G(RO-B2PLYP-D3)
Conformers				
Tosyl_NH_pentane_Ph_C6_radical_0040_cregen_B2PLYP	-1302.458101	-1302.068881	-1301.983459	-1301.67596659
Tosyl_NH_pentane_Ph_C6_radical_0027_cregen_B2PLYP	-1302.454653	-1302.065359	-1301.984732	-1301.67467640
Tosyl_NH_pentane_Ph_C6_radical_0035_cregen_B2PLYP	-1302.458101	-1302.068882	-1301.982199	-1301.67466790
Tosyl_NH_pentane_Ph_C6_radical_0012_cregen_B2PLYP	-1302.456231	-1302.067117	-1301.982533	-1301.67465040
Tosyl_NH_pentane_Ph_C6_radical_0034_cregen_B2PLYP	-1302.454653	-1302.065360	-1301.984166	-1301.67435195
Tosyl_NH_pentane_Ph_C6_radical_0033_cregen_B2PLYP	-1302.453669	-1302.064698	-1301.984205	-1301.67364041
Tosyl_NH_pentane_Ph_C6_radical_0026_cregen_B2PLYP	-1302.453691	-1302.064719	-1301.984730	-1301.67356914
Tosyl_NH_pentane_Ph_C6_radical_0023_cregen_B2PLYP	-1302.456272	-1302.067076	-1301.984092	-1301.67336483
Tosyl_NH_pentane_Ph_C6_radical_0008_cregen_B2PLYP	-1302.458003	-1302.068952	-1301.983801	-1301.67312020
Tosyl_NH_pentane_Ph_C6_radical_0030_cregen_B2PLYP	-1302.453691	-1302.064720	-1301.984171	-1301.67300209
Post-reactive complexes for 1,6-HAT				
Tosyl_NH_pentane_Ph_1_6_HAT_TS_0014_cregen_B2PLYP_IRC_reverse_opt_cregen_B2PLYP_pic	-1302.453669	-1302.064698	-1301.981470	-1301.67090564
Tosyl_NH_pentane_Ph_1_6_HAT_TS_0015_cregen_B2PLYP_IRC_reverse_opt_cregen_B2PLYP_pic	-1302.453691	-1302.064719	-1301.981509	-1301.67031493
Tosyl_NH_pentane_Ph_1_6_HAT_TS_0012_cregen_B2PLYP_IRC_forward_opt_cregen_B2PLYP_pic	-1302.455027	-1302.065897	-1301.977273	-1301.66890363
Tosyl_NH_pentane_Ph_1_6_HAT_TS_0000_cregen_B2PLYP_IRC_forward_opt_cregen_B2PLYP_pic	-1302.454797	-1302.065779	-1301.977582	-1301.66884705
Tosyl_NH_pentane_Ph_1_6_HAT_TS_0010_cregen_B2PLYP_IRC_forward_opt_cregen_B2PLYP_pic	-1302.455027	-1302.065897	-1301.977271	-1301.66866910
Tosyl_NH_pentane_Ph_1_6_HAT_TS_0013_cregen_B2PLYP_IRC_forward_opt_cregen_B2PLYP_pic	-1302.454583	-1302.065402	-1301.976385	-1301.66761747
Tosyl_NH_pentane_Ph_1_6_HAT_TS_0006_cregen_B2PLYP_IRC_forward_opt_cregen_B2PLYP_pic	-1302.454116	-1302.064733	-1301.976377	-1301.66683770
Tosyl_NH_pentane_Ph_1_6_HAT_TS_0008_cregen_B2PLYP_IRC_forward_opt_cregen_B2PLYP_pic	-1302.454116	-1302.064733	-1301.976376	-1301.66676997
Tosyl_NH_pentane_Ph_1_6_HAT_TS_0001_cregen_B2PLYP_IRC_forward_opt_cregen_B2PLYP_pic	-1302.455046	-1302.065911	-1301.976633	-1301.66645925
Tosyl_NH_pentane_Ph_1_6_HAT_TS_0007_cregen_B2PLYP_IRC_forward_opt_cregen_B2PLYP_pic	-1302.455689	-1302.066261	-1301.975921	-1301.66624321

```
 44
-1301.67596659
C  0.005996 -2.884506  0.519915
N  0.262699 -2.031835 -0.648491
S -0.987228 -1.408208 -1.573204
O -0.329226 -0.768160 -2.713549
O -1.966143 -2.488067 -1.727102
C -1.793105 -0.104475 -0.630129
C -1.214514  1.166822 -0.579361
C -2.978042 -0.377011  0.054780
C -1.829818  2.162828  0.174341
H -0.303866  1.375583 -1.131329
C -3.581371  0.634101  0.802301
H -3.424743 -1.362960 -0.016260
C -3.020324  1.915833  0.874827
H -4.507223  0.424350  1.332735
C  0.130898 -2.209122  1.902394
H -0.219422 -1.171940  1.834136
H -0.556488 -2.720169  2.589423
C  1.534469 -2.264105  2.531721
H  1.836348 -3.315862  2.632270
H  1.473857 -1.865891  3.553857
C  2.653598 -1.507787  1.779940
C  2.435960 -0.027202  1.729468
H  2.139289  0.439290  2.668782
H -1.378641  3.151312  0.215324
C -3.695959  3.015542  1.659263
H -2.964065  3.684495  2.124676
H -4.329306  3.631913  1.007541
H -4.337290  2.610284  2.448482
H  1.034337 -1.373397 -0.592356
H  2.782809 -1.932487  0.777040
C  2.577751  0.830535  0.612756
C  2.284239  2.221596  0.753297
C  2.989089  0.388082 -0.680824
C  2.375353  3.093133 -0.319496
H  1.978543  2.591043  1.729315
C  3.066014  1.268868 -1.751696
```

H 3.263582 -0.651414 -0.835742
C 2.759707 2.625366 -1.585733
H 2.146398 4.146487 -0.178056
H 3.368952 0.897561 -2.726665
H 2.823359 3.308402 -2.427540
H -0.997872 -3.292498 0.377745
H 0.690489 -3.741822 0.461533
H 3.601015 -1.719272 2.304402
44
-1301.67467640
C 2.500092 -2.156859 -0.726601
N 2.703697 -1.496358 0.566003
S 3.189555 0.096883 0.713359
O 3.367744 0.293969 2.153681
O 4.269827 0.290922 -0.256666
C 1.834291 1.161713 0.199130
C 1.854238 1.743148 -1.068240
C 0.765866 1.388883 1.072217
C 0.786869 2.550631 -1.463925
H 2.703379 1.576181 -1.722202
C -0.293067 2.190535 0.659003
H 0.776038 0.957117 2.068065
C -0.300262 2.784688 -0.613386
H -1.128854 2.359916 1.333121
C 1.061056 -2.153129 -1.266723
H 0.729822 -1.122225 -1.438579
H 1.079722 -2.646609 -2.249559
C 0.061643 -2.881033 -0.358474
H -0.037284 -2.343618 0.594562
H 0.449581 -3.882379 -0.120006
C -1.350729 -3.047007 -0.978187
C -2.070453 -1.763536 -1.267037
H -1.941287 -1.329030 -2.256269
H 0.804063 3.010587 -2.449033
C -1.448737 3.668764 -1.036219
H -1.444110 4.615499 -0.480948
H -1.398583 3.910425 -2.102423
H -2.410943 3.182748 -0.839240
H 2.116011 -1.786321 1.340433
H -1.947285 -3.673704 -0.302347
C -2.889511 -1.049110 -0.358976
C -3.541468 0.148374 -0.780411
C -3.116814 -1.467909 0.985134
C -4.359978 0.867389 0.076493
H -3.393109 0.488200 -1.802785
C -3.933064 -0.736856 1.836983
H -2.642514 -2.373587 1.350860
C -4.562074 0.434598 1.395558
H -4.853721 1.768402 -0.279516
H -4.086513 -1.080568 2.856869
H -5.204115 0.998605 2.066008
H 3.183716 -1.675994 -1.431656
H 2.847961 -3.192328 -0.612814
H -1.248666 -3.615773 -1.912430
44
-1301.67466790
C 0.072751 -2.622309 -0.542021
N -0.267308 -1.714097 0.562198
S 0.948647 -1.177981 1.590449
O 0.275631 -0.331414 2.576359
O 1.728717 -2.359669 1.967796
C 2.033114 -0.113243 0.626151
C 1.711768 1.238481 0.474178
C 3.170236 -0.647722 0.018415
C 2.537021 2.050637 -0.300007
H 0.835467 1.645917 0.968005
C 3.984887 0.180565 -0.753229
H 3.420032 -1.692623 0.169140
C 3.684277 1.538222 -0.924680
H 4.873307 -0.234165 -1.223553
C -1.149678 -3.407095 -1.042691
H -0.763138 -4.288092 -1.572587
H -1.694082 -3.785263 -0.168144
C -2.122393 -2.694799 -2.001525
H -2.822917 -3.455891 -2.367618
H -1.571806 -2.345538 -2.887394
C -2.952548 -1.524195 -1.431123
C -2.240498 -0.203145 -1.405304
H -1.445282 -0.062915 -2.137395
H 2.292019 3.104109 -0.414520
C 4.590138 2.437619 -1.731875
H 5.160962 1.870762 -2.474325
H 4.021935 3.212448 -2.257588
H 5.314134 2.949795 -1.084386
H -0.879032 -0.938709 0.305012
H -3.323597 -1.792544 -0.432152
C -2.593944 0.925311 -0.622035
C -1.868706 2.146125 -0.770023
C -3.657008 0.913295 0.328153
C -2.184597 3.268152 -0.020851
H -1.054095 2.183631 -1.489683
C -3.963071 2.043109 1.072916
H -4.236456 0.006816 0.471928
C -3.234068 3.227822 0.908416
H -1.616812 4.185043 -0.157253
H -4.776704 2.005136 1.792417
H -3.478791 4.107397 1.496644
H 0.555366 -2.092027 -1.379330
H 0.799794 -3.329811 -0.135816
H -3.858377 -1.419427 -2.054406
44
-1301.67465040
C 2.616758 -2.236705 -0.471697
N 2.631392 -1.330130 0.690662
S 3.294204 0.218874 0.555308
O 3.688034 0.561289 1.926878
O 4.255712 0.281518 -0.552811
C 1.875637 1.205981 0.081531
C 1.849402 1.820889 -1.167432
C 0.805851 1.342243 0.972441
C 0.726473 2.567090 -1.533091
H 2.695502 1.711992 -1.837238
C -0.306121 2.082072 0.589412
H 0.845395 0.866156 1.946784
C -0.366821 2.701822 -0.670873
H -1.149316 2.170159 1.269672
C 1.308587 -3.036422 -0.536455
H 1.419431 -3.813882 -1.305616
H 1.161668 -3.564836 0.416596
C 0.088689 -2.161250 -0.844565
H 0.204223 -1.715608 -1.842301
H 0.052536 -1.325458 -0.137572
C -1.255894 -2.926489 -0.784508
C -2.425953 -2.059971 -1.141445
H -2.716207 -2.028536 -2.190306
H 0.700620 3.045425 -2.509025
C -1.592462 3.486681 -1.072397
H -2.495090 2.868647 -0.995869
H -1.739386 4.355067 -0.418044
H -1.516158 3.852355 -2.100874
H 2.987282 -1.739835 1.550899
H -1.374613 -3.351200 0.222012
C -3.118091 -1.193326 -0.261015
C -4.159209 -0.353992 -0.759600
C -2.828647 -1.101895 1.132555
C -4.853240 0.511368 0.071592
H -4.405560 -0.404686 -1.817771
C -3.531062 -0.233444 1.956690
H -2.045821 -1.722639 1.557435
C -4.546370 0.582013 1.438794
H -5.643491 1.134797 -0.339366
H -3.290972 -0.189510 3.016272
H -5.093886 1.257315 2.090087
H 2.732936 -1.628308 -1.373793
H 3.482431 -2.910850 -0.439274
H -1.213662 -3.780088 -1.474418
44
-1301.67435195
C -1.201690 -1.619274 -1.127414
N -0.972721 -1.836094 0.308483

S 0.539788 -2.327981 0.851968
O 0.457966 -2.229633 2.311456
O 0.852053 -3.582878 0.164470
C 1.739157 -1.113245 0.283135
C 1.795049 0.143116 0.893583
C 2.593786 -1.430224 -0.772443
C 2.709297 1.084452 0.431265
H 1.136079 0.379879 1.722649
C 3.509373 -0.476693 -1.218697
H 2.545707 -2.416004 -1.222307
C 3.582170 0.790769 -0.628185
H 4.179326 -0.724519 -2.038499
C -2.621008 -2.027132 -1.544034
H -2.725785 -1.792994 -2.613341
H -2.705930 -3.116217 -1.447854
C -3.785575 -1.384758 -0.770670
H -3.787349 -1.756173 0.262239
H -4.717095 -1.747681 -1.222349
C -3.814665 0.166466 -0.755598
C -3.035104 0.781599 0.367290
H -3.286830 0.413028 1.362926
H 2.745205 2.064685 0.899896
C 4.589933 1.812487 -1.098618
H 5.448702 1.862320 -0.416379
H 4.974965 1.568100 -2.093522
H 4.152617 2.816294 -1.139926
H -1.324681 -1.084439 0.897745
H -4.866793 0.477503 -0.644392
C -2.099090 1.843143 0.308047
C -1.522551 2.338286 1.518164
C -1.684670 2.468147 -0.905665
C -0.606769 3.378218 1.512024
H -1.825166 1.888068 2.461005
C -0.764533 3.506573 -0.899500
H -2.099585 2.132388 -1.850820
C -0.216940 3.973092 0.303017
H -0.194704 3.737241 2.451721
H -0.469906 3.964418 -1.840362
H 0.496812 4.791910 0.299242
H -0.996205 -0.574328 -1.397786
H -0.488917 -2.245425 -1.671463
H -3.495212 0.552257 -1.730902
44
-1301.67364041
C -0.308230 -2.317892 -0.806723
N -0.345678 -1.744659 0.545425
S 1.084087 -1.550536 1.407707
O 0.685421 -0.937009 2.675358
O 1.790050 -2.832011 1.337623
C 2.088743 -0.347090 0.523730
C 1.896668 1.016662 0.760494
C 3.036566 -0.782154 -0.404136
C 2.658171 1.943958 0.052728
H 1.171362 1.340931 1.499493
C 3.789011 0.160241 -1.104281
H 3.193631 -1.844883 -0.555116
C 3.614376 1.533751 -0.888636
H 4.530262 -0.178196 -1.824323
C -1.626894 -3.025193 -1.149523
H -1.533625 -3.417364 -2.172488
H -1.724351 -3.892107 -0.484879
C -2.915037 -2.193905 -1.040311
H -3.044422 -1.850249 -0.006416
H -3.758621 -2.867411 -1.240867
C -3.039161 -0.983189 -2.007726
C -2.184633 0.207230 -1.681413
H -1.289134 0.369812 -2.277684
H 2.515360 3.005549 0.241142
C 4.458995 2.547593 -1.623217
H 5.347461 2.817279 -1.037033
H 4.809134 2.157652 -2.584362
H 3.903192 3.472115 -1.812213
H -0.924329 -0.911549 0.636795
H -4.096746 -0.681925 -2.020447
C -2.446908 1.152019 -0.656733
C -1.539075 2.231176 -0.432812
C -3.598207 1.090566 0.184318
C -1.767826 3.173807 0.557050
H -0.652189 2.303471 -1.057791
C -3.815403 2.040259 1.172677
H -4.319970 0.291653 0.045801
C -2.906880 3.087885 1.370525
H -1.059784 3.985987 0.701444
H -4.700932 1.967786 1.798726
H -3.082794 3.826279 2.147257
H -0.065290 -1.553570 -1.558550
H 0.495833 -3.058509 -0.814195
H -2.809129 -1.323745 -3.025478
44
-1301.67356914
C 2.499939 -2.157132 -0.726556
N 2.703383 -1.496850 0.566213
S 3.189525 0.096314 0.713767
O 3.367474 0.293272 2.154137
O 4.270022 0.290149 -0.256056
C 1.834588 1.161495 0.199368
C 0.766160 1.389020 1.072356
C 1.854789 1.742830 -1.068051
C -0.292515 2.190955 0.659005
H 0.776118 0.957301 2.068227
C 0.787678 2.550586 -1.463875
H 2.703928 1.575571 -1.721943
C -0.299442 2.785030 -0.613421
H 0.805060 3.010447 -2.449024
C 1.061031 -2.152986 -1.267016
H 0.730026 -1.121962 -1.438588
H 1.079831 -2.646126 -2.250021
C 0.061281 -2.881048 -0.359263
H -0.037647 -2.344092 0.594037
H 0.448898 -3.882626 -0.121248
C -1.351074 -3.046261 -0.979233
C -2.070365 -1.762386 -1.267358
H -1.940468 -1.327013 -2.256115
H -1.128306 2.360618 1.333049
C -1.447566 3.669523 -1.036337
H -1.397781 3.910406 -2.102736
H -2.410000 3.184298 -0.838530
H -1.442042 4.616661 -0.481750
H 2.115103 -1.786560 1.340298
H -1.947863 -3.673207 -0.303843
C -2.889765 -1.048539 -0.359148
C -3.541100 0.149529 -0.779907
C -3.117986 -1.468441 0.984466
C -4.359893 0.868042 0.077143
H -3.392016 0.490213 -1.801891
C -3.934526 -0.737890 1.836471
H -2.644158 -2.374568 1.349696
C -4.562916 0.434145 1.395705
H -4.853128 1.769538 -0.278352
H -4.088674 -1.082446 2.855968
H -5.205169 0.997770 2.066276
H 3.183839 -1.676316 -1.431376
H 2.847549 -3.192694 -0.612827
H -1.249063 -3.614479 -1.913820
44
-1301.67336483
C 0.826872 -2.702783 -0.433505
N 2.144396 -2.201415 -0.035763
S 2.365745 -0.979874 1.087098
O 1.369317 -1.210810 2.136086
O 3.807033 -0.970327 1.347054
C 1.969718 0.597373 0.318172
C 2.958529 1.280660 -0.394045
C 0.679393 1.118965 0.427461
C 2.640877 2.488180 -1.011481
H 3.965504 0.879104 -0.442720
C 0.378958 2.327946 -0.198853
H -0.074252 0.594492 1.004853
C 1.349865 3.030025 -0.926183
H -0.628255 2.727948 -0.115189

C 0.250470 -2.083700 -1.716049
H 0.073234 -1.012073 -1.565711
H 1.003790 -2.168644 -2.512285
C -1.041641 -2.771566 -2.187035
H -0.867003 -3.853610 -2.270874
H -1.281002 -2.422106 -3.200060
C -2.275914 -2.537340 -1.282039
C -2.766929 -1.123128 -1.305896
H -2.980226 -0.717119 -2.295092
H 3.410400 3.023010 -1.563308
C 1.024590 4.355921 -1.572155
H 1.622792 4.520948 -2.474455
H -0.033210 4.418649 -1.847530
H 1.232772 5.188712 -0.887296
H 2.875195 -2.208428 -0.740745
H -2.061665 -2.870424 -0.261112
C -3.009659 -0.266482 -0.206421
C -3.514051 1.049711 -0.440682
C -2.778243 -0.632777 1.153636
C -3.764362 1.927512 0.602056
H -3.706491 1.356492 -1.466298
C -3.028193 0.256389 2.189790
H -2.396691 -1.621153 1.388753
C -3.522436 1.541631 1.929052
H -4.154290 2.919729 0.388460
H -2.835407 -0.051555 3.214026
H -3.717271 2.230467 2.745813
H 0.922405 -3.789861 -0.560660
H 0.168298 -2.547870 0.424737
H -3.079179 -3.198950 -1.646592
44
-1301.67312020
C -0.256198 -1.255949 1.246044
N 0.581537 -2.096697 0.377924
S 1.346182 -1.435861 -0.961838
O 2.136712 -2.531061 -1.526955
O 0.306214 -0.740043 -1.726758
C 2.492539 -0.182479 -0.374648
C 2.053474 1.130672 -0.190005
C 3.810188 -0.542726 -0.082248
C 2.944982 2.082526 0.301868
H 1.035101 1.404152 -0.445512
C 4.687446 0.422975 0.405695
H 4.142322 -1.561093 -0.255910
C 4.271691 1.747412 0.606316
H 5.714884 0.145337 0.628600
C -1.359304 -2.075854 1.926346
H -0.905217 -2.786996 2.631195
H -1.946324 -1.374548 2.533807
C -2.284929 -2.842309 0.967831
H -3.069772 -3.325192 1.566043
H -1.721146 -3.646608 0.481519
C -2.940678 -1.980937 -0.137999
C -3.757436 -0.841844 0.388105
H -4.522336 -1.099811 1.121710
H 2.605227 3.105429 0.444840
C 5.239684 2.793974 1.104356
H 5.776028 3.261956 0.268261
H 4.723398 3.592231 1.647236
H 5.992597 2.359718 1.770286
H 1.200943 -2.745452 0.857517

44
-1301.67090564
C -0.755376 -2.236082 1.924732
C 0.392181 -1.786201 2.849572
C 1.189232 -0.531223 2.431662
C 2.276776 -0.773109 1.426728
C -0.378989 -2.870819 0.573116
N 0.074464 -1.975761 -0.494084
H 1.089641 -2.623483 3.001106
H -0.049003 -1.585363 3.833979
H 1.644712 -0.104431 3.343026
H 0.490673 0.238617 2.074363
H 0.894136 -1.400682 -0.310147
H -3.590494 -2.651234 -0.724925
C -3.658569 0.525116 0.032354
C -4.530300 1.476031 0.645866
C -2.722960 1.029529 -0.922532
C -4.469854 2.824200 0.333182
H -5.255934 1.120652 1.374243
C -2.673801 2.383388 -1.225945
H -2.038057 0.352812 -1.423055
C -3.540310 3.293785 -0.606200
H -5.149404 3.520350 0.818778
H -1.954558 2.736325 -1.961160
H -3.495499 4.350816 -0.853119
H 0.347811 -0.736740 2.004855
H -0.707484 -0.491370 0.610314
H -2.169154 -1.636291 -0.832212
44
-1301.67300209
C -0.299627 -2.315428 -0.810653
N -0.341128 -1.744359 0.542278
S 1.086404 -1.548569 1.407843
O 0.683922 -0.936473 2.674975
O 1.794557 -2.828865 1.338558
C 2.090242 -0.342986 0.525769
C 1.895658 1.020556 0.762866
C 3.046346 -0.776281 -0.394073
C 2.661225 1.949080 0.061496
H 1.170444 1.343129 1.502702
C 3.803185 0.167622 -1.087860
H 3.211392 -1.838710 -0.538497
C 3.620993 1.540754 -0.877433
H 4.556015 -0.169726 -1.796223
C -1.615575 -3.026123 -1.156875
H -1.519580 -3.415937 -2.180490
H -1.711351 -3.894673 -0.494127
C -2.906446 -2.199052 -1.047837
H -3.038468 -1.858140 -0.013374
H -3.747636 -2.874686 -1.251256
C -3.032785 -0.986487 -2.012658
C -2.182556 0.205824 -1.682058
H -1.286432 0.372471 -2.276272
H 2.521559 3.010044 0.255720
C 4.427748 2.558574 -1.648236
H 4.663978 3.432532 -1.031892
H 5.369076 2.133302 -2.010266
H 3.873502 2.920321 -2.524611
H -0.921797 -0.912676 0.633803
H -4.091287 -0.688523 -2.026460
C -2.449507 1.147368 -0.655596
C -1.545252 2.228602 -0.427281
C -3.602203 1.080510 0.183128
C -1.778561 3.168125 0.564463
H -0.657361 2.305001 -1.050343
C -3.824012 2.027138 1.173394
H -4.321360 0.279828 0.041320
C -2.918903 3.076898 1.375543
H -1.073093 3.981930 0.712221
H -4.710495 1.950553 1.797597
H -3.098412 3.812848 2.153769
H -0.057633 -1.549224 -1.560868
H 0.506690 -3.053598 -0.817926
H -2.799968 -1.323917 -3.030819

H 0.412481 -3.617753 0.721195
H -1.331411 -2.995636 2.471865
H -1.444094 -1.398507 1.755403
H 2.702110 -1.776271 1.393514
S -0.960680 -1.294371 -1.608968
O -1.971897 -2.310895 -1.914571
O -0.092553 -0.708666 -2.631775
C -1.825038 0.077844 -0.824152
C -3.122097 -0.103138 -0.343742
C -1.185601 1.315008 -0.701256
C -3.775058 0.961562 0.278318
H -3.615216 -1.059903 -0.479109
C -1.851588 2.365863 -0.075231

H -0.189957 1.455245 -1.110111
C -3.151955 2.206965 0.428955
H -4.790128 0.823847 0.643080
H -1.359468 3.331976 0.011400
C -3.853827 3.349837 1.123501
H -4.937855 3.200762 1.145213
H -3.515089 3.448892 2.163416
H -3.652172 4.304963 0.626379
H -1.238563 -3.409770 0.165938
C 2.896516 0.212723 0.616606
C 3.958873 -0.153762 -0.262631
C 2.509864 1.585794 0.630280
C 4.581879 0.782381 -1.071668
H 4.274353 -1.194007 -0.293659
C 3.143501 2.514796 -0.183180
H 1.714771 1.910968 1.294031
C 4.180493 2.125255 -1.040963
H 5.384856 0.471424 -1.734557
H 2.832683 3.556028 -0.150457
H 4.669062 2.857656 -1.676918
44
-1301.67031493
C -0.748223 -2.249153 1.914933
C 0.401851 -1.806620 2.840231
C 1.197071 -0.547698 2.430763
C 2.281243 -0.779845 1.419936
C -0.375320 -2.875797 0.558562
N 0.077095 -1.974597 -0.503931
H 1.100057 -2.644882 2.982706
H -0.036566 -1.614181 3.827537
H 1.655537 -0.129000 3.344386
H 0.496904 0.224993 2.082953
H 0.896355 -1.399772 -0.316861
H 0.415592 -3.624520 0.700506
H -1.323897 -3.011642 2.458351
H -1.436366 -1.409723 1.752597
H 2.708598 -1.781826 1.377839
S -0.959204 -1.287013 -1.613884
O -1.970037 -2.302257 -1.925096
O -0.092053 -0.694533 -2.633637
C -1.824487 0.080107 -0.821282
C -1.185727 1.316310 -0.690589
C -3.118705 -0.107016 -0.334336
C -1.850045 2.361393 -0.052261
H -0.189066 1.459048 -1.096091
C -3.769609 0.951213 0.299743
H -3.608303 -1.065822 -0.468065
C -3.149324 2.198370 0.451947
H -1.355650 3.325064 0.047176
H -4.779713 0.806784 0.675793
C -3.873083 3.349908 1.108619
H -4.387189 3.968810 0.361334
H -4.630675 2.997110 1.815814
H -3.180497 4.003909 1.649064
H -1.236232 -3.411315 0.149677
C 2.896173 0.213289 0.615055
C 2.507197 1.585534 0.640678
C 3.955842 -0.144587 -0.270943
C 3.136141 2.521824 -0.168066
H 1.714147 1.904247 1.309983
C 4.574145 0.798787 -1.075178
H 4.273016 -1.184002 -0.311100
C 4.170536 2.140668 -1.032729
H 2.823688 3.562240 -0.126185
H 5.375130 0.494299 -1.743457
H 4.655407 2.878757 -1.664929
44
-1301.66890363
C -1.133492 2.602360 -1.476235
C -2.427567 3.118485 -0.820203
C -3.199228 2.117173 0.069596
C -3.744073 0.948880 -0.693564
C 0.014568 2.273204 -0.505406
N -0.021407 0.861073 -0.079505
H -3.101556 3.461111 -1.617509
H -2.189082 4.006543 -0.219447
H -4.034809 2.670203 0.531557
H -2.570451 1.798166 0.908614
H -0.945896 0.454958 0.046874
H 0.976824 2.429547 -1.006237
H -1.340513 1.725251 -2.102823
H -0.774005 3.384076 -2.156749
H -4.259247 1.192956 -1.622876
S 0.948529 0.391890 1.217171
O 0.300282 -0.806630 1.764298
O 1.252598 1.546016 2.074397
C 2.479439 -0.093114 0.420996
C 2.517231 -1.271110 -0.331275
C 3.617172 0.695279 0.575861
C 3.709998 -1.648998 -0.936948
H 1.624899 -1.880379 -0.431539
C 4.806347 0.300300 -0.040402
H 3.567888 1.596326 1.177603
C 4.872780 -0.871212 -0.802481
H 3.744053 -2.564674 -1.522691
H 5.696594 0.912909 0.078370
C 6.163396 -1.306717 -1.454677
H 6.014412 -1.545822 -2.514159
H 6.929276 -0.528119 -1.388218
H 6.563710 -2.208919 -0.974796
H -0.013785 2.949344 0.359240
C -3.691945 -0.420946 -0.339833
C -4.270445 -1.392850 -1.212965
C -3.074232 -0.911259 0.849598
C -4.223481 -2.745764 -0.922446
H -4.753239 -1.049409 -2.125078
C -3.021629 -2.271148 1.123621
H -2.633632 -0.220761 1.561572
C -3.594906 -3.200155 0.246972
H -4.673905 -3.459108 -1.608157
H -2.524149 -2.608944 2.028173
H -3.552649 -4.262554 0.469268
44
-1301.66884705
C 0.440095 3.122708 -0.730825
C 1.950383 3.386264 -0.558726
C 2.921301 2.239132 -0.908490
C 3.055922 1.170388 0.136156
C -0.262185 2.264298 0.331595
N -0.066453 0.821760 0.101693
H 2.145022 3.725875 0.469007
H 2.202041 4.235904 -1.206072
H 3.914983 2.688063 -1.086087
H 2.629640 1.803318 -1.874786
H 0.918034 0.539362 0.089819
H 0.055600 2.565026 1.339909
H -0.058876 4.100613 -0.714238
H 0.242116 2.693594 -1.722036
H 2.845066 1.457201 1.166462
S -0.872039 -0.230487 1.153369
O -1.095328 0.399541 2.463450
O -0.146108 -1.500792 1.046724
C -2.474913 -0.401360 0.368113
C -2.574753 -1.090403 -0.844507
C -3.605630 0.125099 0.988125
C -3.823207 -1.239434 -1.437378
H -1.685250 -1.504503 -1.307861
C -4.851601 -0.033740 0.377830
H -3.504871 0.641638 1.936575
C -4.980882 -0.714725 -0.837525
H -3.905601 -1.774576 -2.380574
H -5.736393 0.376084 0.858630
C -6.330803 -0.901616 -1.488353
H -7.102754 -0.308602 -0.988689
H -6.309021 -0.608510 -2.544524
H -6.644141 -1.952859 -1.452990
H -1.340777 2.446213 0.270351
C 3.576706 -0.132719 -0.074939
C 3.674599 -1.044355 1.017594
C 4.019287 -0.594128 -1.349133

C 4.178210 -2.323249 0.845530
H 3.332362 -0.723194 1.998120
C 4.525416 -1.876030 -1.508411
H 3.968597 0.069660 -2.206759
C 4.609782 -2.751187 -0.417678
H 4.233199 -2.998000 1.695479
H 4.859897 -2.201881 -2.490128
H 5.004051 -3.754617 -0.550613
44
-1301.66866910
C -1.133468 2.603454 -1.475524
C -2.427064 3.119109 -0.818150
C -3.198220 2.117091 0.071274
C -3.743853 0.949533 -0.692486
C 0.015313 2.273426 -0.505875
N -0.020967 0.861214 -0.080374
H -3.101538 3.462471 -1.614731
H -2.188105 4.006640 -0.216810
H -4.033398 2.669805 0.534347
H -2.568878 1.797239 0.909532
H -0.945443 0.454702 0.044782
H 0.977171 2.429583 -1.007550
H -1.341043 1.726916 -2.102740
H -0.774385 3.385706 -2.155634
H -4.259837 1.194437 -1.621129
S 0.948287 0.391194 1.216372
O 0.299716 -0.807593 1.762522
O 1.252116 1.544830 2.074344
C 2.479599 -0.093598 0.420821
C 2.518150 -1.272144 -0.330427
C 3.616774 0.695912 0.574670
C 3.711130 -1.649605 -0.936100
H 1.626253 -1.882143 -0.430131
C 4.806055 0.301398 -0.041533
H 3.566833 1.597558 1.175462
C 4.873308 -0.870909 -0.802451
H 3.745704 -2.565602 -1.521293
H 5.695769 0.914990 0.076244
C 6.164844 -1.306807 -1.452549
H 6.579907 -2.191065 -0.952034
H 6.011909 -1.575248 -2.504289
H 6.921374 -0.517365 -1.411186
H -0.012029 2.949244 0.359050
C -3.692242 -0.420525 -0.339501
C -4.272041 -1.391594 -1.212707
C -3.073823 -0.911871 0.849122
C -4.225699 -2.744697 -0.922975
H -4.755355 -1.047355 -2.124244
C -3.021884 -2.271949 1.122365
H -2.632066 -0.222083 1.561056
C -3.596476 -3.200117 0.245698
H -4.677119 -3.457379 -1.608718
H -2.523874 -2.610543 2.026327
H -3.554744 -4.262661 0.467404
44
-1301.66761747
C -2.551513 -2.845388 -0.125140
C -3.729037 -2.001327 -0.653589
C -3.450036 -0.513979 -0.945519
C -2.888515 0.255515 0.214583
C -1.259711 -2.807192 -0.965498
N -0.343682 -1.673280 -0.709626
H -4.549385 -2.070281 0.073296
H -4.104748 -2.458974 -1.578898
H -4.392871 -0.052361 -1.285348
H -2.782139 -0.430885 -1.819501
H -0.821412 -0.771521 -0.704112
H -0.672107 -3.711591 -0.792836
H -2.306659 -2.564362 0.905482
H -2.892258 -3.888294 -0.078424
H -2.839331 -0.242058 1.179611
S 0.597402 -1.761558 0.703875
O 1.040641 -3.156075 0.817039
O -0.085910 -1.105691 1.831950
C 1.995584 -0.736280 0.254379
C 1.985606 0.622076 0.568223
C 3.085866 -1.318408 -0.396635
C 3.081405 1.406888 0.207804
H 1.139920 1.055587 1.091841
C 4.171796 -0.520325 -0.741943
H 3.081034 -2.381769 -0.611359
C 4.185758 0.853367 -0.451475
H 3.078458 2.466581 0.450321
H 5.026694 -0.970339 -1.241260
C 5.363569 1.709309 -0.852740
H 6.313525 1.232003 -0.586547
H 5.379402 1.875216 -1.937790
H 5.330068 2.690267 -0.369048
H -1.493345 -2.777080 -2.037275
C -2.405770 1.586807 0.152536
C -2.452764 2.373687 -1.035253
C -1.836842 2.191294 1.313418
C -1.966753 3.674021 -1.051412
H -2.883529 1.952405 -1.938921
C -1.353897 3.490292 1.283921
H -1.778268 1.605700 2.227183
C -1.414679 4.244653 0.102586
H -2.018337 4.254119 -1.969437
H -0.927523 3.926140 2.183982
H -1.038086 5.263451 0.083030
44
-1301.66683770
C 0.531394 2.892595 -0.538013
C 2.070426 2.916751 -0.380697
C 2.654674 2.467434 0.990400
C 2.617022 0.998872 1.296205
C -0.097389 1.625593 -1.134142
N -0.084251 0.504586 -0.181094
H 2.402048 3.952242 -0.531377
H 2.534119 2.330975 -1.185748
H 2.124857 3.012239 1.782104
H 3.695364 2.820935 1.024858
H 0.858822 0.240637 0.111576
H -1.147345 1.833505 -1.368321
H 0.048033 3.115265 0.422329
H 0.254358 3.708662 -1.217963
H 1.854530 0.646388 1.987961
S -0.876631 -0.918823 -0.632245
O -0.340984 -1.940121 0.276787
O -0.846093 -1.117681 -2.088253
C -2.575363 -0.561371 -0.182342
C -2.917312 -0.432557 1.167310
C -3.541674 -0.450656 -1.179501
C -4.240926 -0.180860 1.509554
H -2.154194 -0.532089 1.932166
C -4.866356 -0.197679 -0.817423
H -3.254501 -0.568853 -2.218767
C -5.236431 -0.059847 0.524885
H -4.511102 -0.079058 2.558075
H -5.622998 -0.109776 -1.593106
C -6.672406 0.195167 0.916768
H -7.111426 -0.686021 1.401946
H -6.751049 1.025825 1.627758
H -7.289460 0.435561 0.045753
H 0.398730 1.361763 -2.078839
C 3.509582 0.025920 0.775682
C 3.319768 -1.353194 1.090541
C 4.611102 0.354471 -0.068692
C 4.172270 -2.327922 0.597427
H 2.473090 -1.636482 1.710600
C 5.460712 -0.630297 -0.551461
H 4.794377 1.392025 -0.331070
C 5.251837 -1.976851 -0.225089
H 3.996303 -3.370757 0.846972
H 6.295614 -0.352067 -1.189696
H 5.918708 -2.742855 -0.610380
44
-1301.66676997
C 0.531446 2.892448 -0.537644
C 2.070451 2.916699 -0.380129

C 2.654471 2.467311 0.991030
C 2.616858 0.998699 1.296640
C -0.097201 1.625599 -1.134245
N -0.084042 0.504246 -0.181614
H 2.402005 3.952236 -0.530649
H 2.534338 2.331048 -1.185158
H 2.124446 3.011969 1.782693
H 3.695123 2.820894 1.025738
H 0.859040 0.239996 0.110763
H -1.147155 1.833562 -1.368389
H 0.047946 3.114787 0.422706
H 0.254415 3.708696 -1.217379
H 1.854205 0.646078 1.988143
S -0.876782 -0.918857 -0.633093
O -0.341066 -1.940627 0.275382
O -0.846710 -1.117096 -2.089189
C -2.575345 -0.561435 -0.182574
C -3.541722 -0.448880 -1.179523
C -2.917071 -0.434098 1.167235
C -4.866183 -0.195599 -0.817014
H -3.254684 -0.565827 -2.218969
C -4.240547 -0.182065 1.509933
H -2.153916 -0.534912 1.931885
C -5.236072 -0.059372 0.525558
H -5.622834 -0.106120 -1.592516
H -4.510539 -0.081248 2.558589
C -6.672157 0.194579 0.917721
H -7.116378 -0.693769 1.384900
H -6.749649 1.012087 1.643802
H -7.285540 0.453429 0.049380
H 0.398956 1.362136 -2.079023
C 3.509445 0.025861 0.775956
C 3.319516 -1.353355 1.090332
C 4.611175 0.354631 -0.068056
C 4.172091 -2.327951 0.597098
H 2.472676 -1.636812 1.710092
C 5.460859 -0.630019 -0.550954
H 4.794568 1.392254 -0.330084
C 5.251863 -1.976666 -0.225065
H 3.996011 -3.370860 0.846258
H 6.295910 -0.351598 -1.188910
H 5.918803 -2.742574 -0.610424
44
-1301.66645925
C -2.440612 -2.753879 -1.376499
C -3.692489 -1.870449 -1.195149
C -3.503790 -0.339574 -1.230382
C -2.922089 0.258388 0.017508
C -1.450196 -2.837041 -0.204826
N -0.501663 -1.700678 -0.219658
H -4.197255 -2.148218 -0.258486
H -4.390662 -2.133506 -1.999932
H -4.493908 0.113825 -1.415488
H -2.899747 -0.069034 -2.109101
H -0.995811 -0.803836 -0.188118
H -2.001346 -2.907622 0.745521
H -2.796069 -3.776803 -1.556782
H -1.893253 -2.455753 -2.280737
H -3.027664 -0.313004 0.938911
S 0.632342 -1.693770 1.046685
O 1.086844 -3.079717 1.205717
O 0.104217 -0.945410 2.199929
C 1.958758 -0.724877 0.332161
C 2.966560 -1.375317 -0.382426
C 1.979151 0.658613 0.508315
C 3.999149 -0.621978 -0.933762
H 2.942929 -2.454917 -0.485804
C 3.018632 1.396498 -0.056937
H 1.201404 1.147436 1.085709
C 4.039168 0.772988 -0.786730
H 4.791475 -1.124925 -1.483125
H 3.039461 2.474989 0.078838
C 5.148681 1.581601 -1.416102
H 5.275929 2.546526 -0.915273
H 6.104849 1.048802 -1.376910
H 4.935244 1.786619 -2.473630
H -0.846094 -3.742097 -0.295682
C -2.350666 1.552646 0.129318
C -1.828691 1.990535 1.383656
C -2.265268 2.461070 -0.965864
C -1.266347 3.249625 1.527074
H -1.859562 1.307518 2.228177
C -1.702578 3.719910 -0.807999
H -2.656014 2.168672 -1.935763
C -1.199597 4.126950 0.434595
H -0.877256 3.556645 2.494466
H -1.655220 4.395906 -1.658037
H -0.762406 5.114611 0.551037
44
-1301.66624321
C -1.036288 -2.888052 -0.926648
C -2.543787 -2.559895 -1.061314
C -3.280243 -2.121487 0.236870
C -2.902968 -0.782611 0.797083
C -0.054271 -1.786024 -1.359441
N 0.019725 -0.583111 -0.514635
H -3.059262 -3.459490 -1.421405
H -2.694277 -1.797447 -1.838596
H -3.103674 -2.889027 1.001161
H -4.358100 -2.145913 0.022971
H -0.883881 -0.169432 -0.284682
H 0.956764 -2.201008 -1.415989
H -0.795678 -3.198641 0.096279
H -0.815686 -3.751947 -1.568324
H -2.183666 -0.758170 1.612246
S 0.960795 -0.613506 0.879999
O 1.191196 -1.991285 1.338718
O 0.346056 0.381332 1.771199
C 2.540812 0.015685 0.310301
C 2.621807 1.311917 -0.208409
C 3.678132 -0.778252 0.433508
C 3.857191 1.803319 -0.612849
H 1.727744 1.920801 -0.294542
C 4.911287 -0.268010 0.021651
H 3.592273 -1.776793 0.847921
C 5.021563 1.023100 -0.505014
H 3.923793 2.810043 -1.018814
H 5.800779 -0.886031 0.114870
C 6.355837 1.577795 -0.943816
H 7.149636 0.830018 -0.855910
H 6.323617 1.914417 -1.987076
H 6.643759 2.444420 -0.335666
H -0.306643 -1.438722 -2.369575
C -3.411156 0.468176 0.358973
C -2.934490 1.674030 0.958749
C -4.391932 0.596992 -0.669140
C -3.413175 2.912228 0.560490
H -2.171433 1.603080 1.729408
C -4.863765 1.843128 -1.056178
H -4.782198 -0.294113 -1.151623
C -4.382684 3.010313 -0.447980
H -3.031725 3.812874 1.034866
H -5.615122 1.912086 -1.839014
H -4.757067 3.982376 -0.756396

C_5-rad

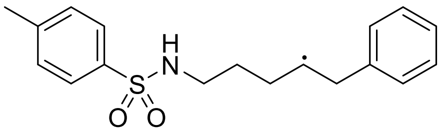

Name	E(B3LYP)	G(B3LYP)	E(RO-B2PLYP-D3)	G(RO-B2PLYP-D3)
Conformers				
Tosyl_NH_pentane_Ph_C5_radical_0035_cregen_B2PLYP	-1302.438778	-1302.050435	-1301.967290	-1301.66270851
Tosyl_NH_pentane_Ph_C5_radical_0032_cregen_B2PLYP	-1302.438778	-1302.050435	-1301.966635	-1301.66216298
Tosyl_NH_pentane_Ph_C5_radical_0026_cregen_B2PLYP	-1302.438728	-1302.050031	-1301.968932	-1301.66112249
Tosyl_NH_pentane_Ph_C5_radical_0033_cregen_B2PLYP	-1302.438778	-1302.050435	-1301.965077	-1301.66052558
Tosyl_NH_pentane_Ph_C5_radical_0024_cregen_B2PLYP	-1302.438729	-1302.050031	-1301.967057	-1301.65926790
Tosyl_NH_pentane_Ph_C5_radical_0037_cregen_B2PLYP	-1302.439638	-1302.051007	-1301.966561	-1301.65893745
Tosyl_NH_pentane_Ph_C5_radical_0029_cregen_B2PLYP	-1302.438482	-1302.049683	-1301.966608	-1301.65877940
Tosyl_NH_pentane_Ph_C5_radical_0018_cregen_B2PLYP	-1302.438030	-1302.049594	-1301.967212	-1301.65870628
Tosyl_NH_pentane_Ph_C5_radical_0008_cregen_B2PLYP	-1302.440070	-1302.051410	-1301.965848	-1301.65811993
Tosyl_NH_pentane_Ph_C5_radical_0030_cregen_B2PLYP	-1302.438482	-1302.049683	-1301.965938	-1301.65810374
Post-reactive complexes for 1,5-HAT				
Tosyl_NH_pentane_Ph_1_5_HAT_TS_0008_cregen_B2PLYP_IRC_forward_opt_cregen_B2PLYP_pic	-1302.440762	-1302.052214	-1301.963658	-1301.65547281
Tosyl_NH_pentane_Ph_1_5_HAT_TS_0007_cregen_B2PLYP_IRC_forward_opt_cregen_B2PLYP_pic	-1302.439950	-1302.051482	-1301.962563	-1301.65511866
Tosyl_NH_pentane_Ph_1_5_HAT_TS_0005_cregen_B2PLYP_IRC_forward_opt_cregen_B2PLYP_pic	-1302.439950	-1302.051482	-1301.962563	-1301.65510882
Tosyl_NH_pentane_Ph_1_5_HAT_TS_0002_cregen_B2PLYP_IRC_forward_opt_cregen_B2PLYP_pic	-1302.441429	-1302.053074	-1301.962790	-1301.65509019
Tosyl_NH_pentane_Ph_1_5_HAT_TS_0000_cregen_B2PLYP_IRC_forward_opt_cregen_B2PLYP_pic	-1302.439830	-1302.051096	-1301.962190	-1301.65506872
Tosyl_NH_pentane_Ph_1_5_HAT_TS_0003_cregen_B2PLYP_IRC_forward_opt_cregen_B2PLYP_pic	-1302.440926	-1302.052428	-1301.963334	-1301.65498054
Tosyl_NH_pentane_Ph_1_5_HAT_TS_0006_cregen_B2PLYP_IRC_forward_opt_cregen_B2PLYP_pic	-1302.439031	-1302.050237	-1301.962185	-1301.65436766
Tosyl_NH_pentane_Ph_1_5_HAT_TS_0011_cregen_B2PLYP_IRC_forward_opt_cregen_B2PLYP_pic	-1302.440673	-1302.052294	-1301.962562	-1301.65421074
Tosyl_NH_pentane_Ph_1_5_HAT_TS_0004_cregen_B2PLYP_IRC_forward_opt_cregen_B2PLYP_pic	-1302.440203	-1302.051911	-1301.961505	-1301.65388478
Tosyl_NH_pentane_Ph_1_5_HAT_TS_0009_cregen_B2PLYP_IRC_forward_opt_cregen_B2PLYP_pic	-1302.440389	-1302.051828	-1301.961582	-1301.65299069

44
-1301.66270851
C -0.636714 -1.868853 -0.529744
N 0.084166 -1.992110 0.745741
S 1.765757 -2.004662 0.748524
O 2.147291 -2.083999 2.159882
O 2.173535 -3.005736 -0.239072
C 2.315908 -0.403142 0.138292
C 2.503838 0.648239 1.038800
C 2.528189 -0.215217 -1.228988
C 2.899855 1.894916 0.558790
H 2.362246 0.479485 2.101385
C 2.921287 1.039656 -1.692590
H 2.408424 -1.049104 -1.912599
C 3.113867 2.112027 -0.810817
H 3.090741 1.185417 -2.756799
C -2.122026 -2.162924 -0.310554
H -2.234040 -3.197250 0.035774
H -2.507673 -1.513753 0.485102
C -2.976076 -1.950052 -1.591217
H -4.004944 -2.254987 -1.346631
H -2.621742 -2.644444 -2.365330
C -2.984687 -0.554753 -2.137029
C -3.799775 0.549597 -1.523717
H -4.804374 0.176034 -1.277624
H 3.057180 2.710985 1.260307
C 3.578953 3.456904 -1.316689
H 3.196239 4.274023 -0.696303
H 4.674855 3.523877 -1.302334
H 3.256223 3.631593 -2.348176
H -0.258376 -1.369526 1.475692
H -2.274993 -0.295067 -2.918895
C -3.190711 1.148514 -0.253244
C -3.873180 1.095984 0.968100
C -1.923245 1.749473 -0.282365
C -3.305562 1.621137 2.132091
H -4.859125 0.637886 1.009303
C -1.350804 2.272152 0.877227

H -1.378226 1.804505 -1.222037
C -2.040333 2.207497 2.091430
H -3.853026 1.569047 3.069708
H -0.363935 2.725297 0.832621
H -1.595904 2.613927 2.995982
H -0.511604 -0.868800 -0.970312
H -0.201197 -2.600327 -1.216618
H -3.937334 1.351483 -2.261916
44
-1301.66216298
C -0.327330 1.014269 2.090040
N -0.348903 1.321994 0.658624
S 0.947839 1.961390 -0.166599
O 0.439906 2.213090 -1.518499
O 1.507188 3.015712 0.683491
C 2.230572 0.705917 -0.325581
C 3.254768 0.636424 0.620477
C 2.184719 -0.197393 -1.390092
C 4.225585 -0.357788 0.504855
H 3.296177 1.365684 1.422619
C 3.163112 -1.184461 -1.491310
H 1.404025 -0.109572 -2.138598
C 4.196373 -1.282984 -0.547677
H 3.130039 -1.884762 -2.322694
C 0.015801 -0.435633 2.470451
H 1.026863 -0.684133 2.124178
H 0.041735 -0.482078 3.567644
C -0.984580 -1.491047 1.953827
H -2.010436 -1.174341 2.194642
H -0.824605 -2.418127 2.537788
C -0.875463 -1.812706 0.495492
C -2.049566 -2.322657 -0.295919
H -2.486914 -3.207123 0.198388
H 5.024732 -0.409910 1.240514
C 5.271420 -2.334690 -0.685284
H 5.693048 -2.607341 0.287627
H 4.885101 -3.244432 -1.156667
H 6.099006 -1.971615 -1.309022
H -0.936098 0.735329 0.071642
H 0.121329 -1.906887 0.069316
C -3.150803 -1.283000 -0.477575
C -2.964184 -0.199321 -1.350062
C -4.353065 -1.365264 0.234224
C -3.949123 0.776926 -1.501111
H -2.042514 -0.121644 -1.924367
C -5.343097 -0.390555 0.084478
H -4.518759 -2.203667 0.907543
C -5.143264 0.684245 -0.781917
H -3.782269 1.608427 -2.180253
H -6.270589 -0.473917 0.645239
H -5.911545 1.443671 -0.898511
H 0.376368 1.712397 2.552444
H -1.320982 1.267854 2.482126
H -1.704826 -2.662111 -1.280206
44
-1301.66112249
C 0.386266 -2.459881 1.118017
N 0.363760 -1.875439 -0.224745
S -1.011685 -1.720454 -1.147471
O -0.535071 -1.355443 -2.483858
O -1.822236 -2.915339 -0.895156
C -1.956534 -0.312413 -0.539153
C -1.531023 0.984974 -0.839341
C -3.102233 -0.526219 0.226980
C -2.256711 2.068677 -0.351933
H -0.650770 1.142085 -1.455218
C -3.819240 0.571571 0.703917
H -3.427986 -1.540717 0.430679
C -3.410776 1.881934 0.424753
H -4.714576 0.405512 1.298305
C 0.079625 -1.509000 2.287225
H -0.971777 -1.200720 2.249325
H 0.203544 -2.083584 3.216387
C 0.965897 -0.249022 2.338151
H 0.644663 0.349954 3.211487
H 0.752541 0.383664 1.466988
C 2.434657 -0.522915 2.422414
C 3.465257 0.433582 1.885934
H 4.459129 -0.025519 1.968476
H -1.926268 3.078521 -0.583905
C -4.207579 3.066485 0.917789
H -4.822775 2.803795 1.784212
H -3.554692 3.898478 1.203349
H -4.883569 3.439090 0.136824
H 1.074349 -1.185929 -0.455663
H 2.771286 -1.295490 3.112712
C 3.217034 0.856996 0.445264
C 2.834884 2.164064 0.124750
C 3.339778 -0.075877 -0.597586
C 2.573978 2.532655 -1.198415
H 2.739842 2.901687 0.918681
C 3.074638 0.285085 -1.919627
H 3.651031 -1.093226 -0.368207
C 2.687262 1.593708 -2.223813
H 2.281146 3.554706 -1.425124
H 3.166707 -0.453340 -2.711060
H 2.480278 1.875535 -3.252308
H -0.318373 -3.295688 1.111187
H 1.388152 -2.887042 1.243363
H 3.506133 1.343410 2.512408
44
-1301.66052558
C 1.191379 -2.428676 1.258333
N 1.496904 -2.210118 -0.160649
S 2.864202 -1.388358 -0.664547
O 3.967905 -1.875689 0.166736
O 2.855881 -1.504447 -2.124231
C 2.663696 0.361026 -0.285487
C 3.174331 0.876883 0.906676
C 1.990021 1.188554 -1.187837
C 2.988299 2.227321 1.201263
H 3.731320 0.231048 1.577236
C 1.812790 2.535010 -0.877462
H 1.639331 0.784312 -2.132000
C 2.300142 3.074789 0.322449
H 1.298843 3.181852 -1.584630
C 0.210284 -1.430355 1.893271
H 0.158941 -1.658695 2.966273
H 0.608174 -0.411332 1.810067
C -1.216995 -1.483464 1.313063
H -1.878691 -0.907290 1.987190
H -1.592927 -2.517659 1.360294
C -1.361266 -0.955211 -0.081999
C -2.559605 -1.288617 -0.929286
H -2.817898 -2.349149 -0.809754
H 3.395107 2.631639 2.125050
C 2.078458 4.529804 0.660555
H 2.106381 5.158316 -0.235826
H 1.097921 4.679959 1.131734
H 2.836037 4.899224 1.358926
H 0.691804 -2.007953 -0.751836
H -0.826075 -0.035394 -0.322105
C -3.772066 -0.434327 -0.562368
C -3.949900 0.833489 -1.132407
C -4.707476 -0.880036 0.380523
C -5.033345 1.634719 -0.771586
H -3.235942 1.192313 -1.871033
C -5.792463 -0.080854 0.745069
H -4.591026 -1.866065 0.825268
C -5.958397 1.180358 0.170759
H -5.158304 2.612222 -1.230418
H -6.511363 -0.447094 1.473535
H -6.804565 1.802033 0.450607
H 0.784701 -3.445201 1.345428
H 2.146129 -2.431278 1.791320
H -2.321045 -1.132636 -1.989228
44
-1301.65926790
C -1.301181 -2.778850 0.802769
N -0.676741 -2.171370 -0.379630

S -1.537065 -1.225836 -1.461175
O -0.582929 -0.929468 -2.532687
O -2.807543 -1.916496 -1.696880
C -1.931080 0.342355 -0.669397
C -3.182731 0.526121 -0.080600
C -0.969714 1.356964 -0.634599
C -3.465114 1.733439 0.558695
H -3.926569 -0.261195 -0.141947
C -1.268835 2.554998 0.009611
H -0.007962 1.212771 -1.116686
C -2.517432 2.763767 0.615614
H -0.522438 3.345428 0.035609
C -1.058814 -2.038707 2.127420
H -1.493601 -1.032335 2.079593
H -1.604990 -2.580270 2.911082
C 0.428591 -1.943511 2.533868
H 0.886684 -2.943433 2.490777
H 0.467653 -1.653352 3.601211
C 1.237027 -0.970725 1.729692
C 2.721012 -1.115523 1.551546
H 2.957486 -2.160310 1.293901
H -4.441716 1.879156 1.014167
C -2.840134 4.080447 1.281203
H -3.165640 4.824937 0.542690
H -3.646185 3.973129 2.013963
H -1.965796 4.495406 1.794132
H 0.251140 -1.782639 -0.212338
H 0.805223 0.014234 1.553600
C 3.295202 -0.176482 0.505585
C 2.967145 -0.331233 -0.849791
C 4.138474 0.879209 0.869917
C 3.464825 0.548122 -1.811810
H 2.320955 -1.147886 -1.164653
C 4.642345 1.760185 -0.089974
H 4.404335 1.013203 1.916403
C 4.305365 1.598248 -1.433887
H 3.196221 0.409134 -2.855431
H 5.297976 2.572018 0.214534
H 4.695689 2.282392 -2.182582
H -2.370655 -2.871243 0.593650
H -0.911122 -3.802549 0.882507
H 3.237608 -0.938866 2.513385
44
-1301.65893745
C 0.331040 -2.416944 0.973773
N 0.283765 -1.792015 -0.351634
S -1.116420 -1.621791 -1.240730
O -0.670737 -1.190296 -2.567783
O -1.898465 -2.842277 -1.025409
C -2.072353 -0.259361 -0.553158
C -3.182517 -0.526638 0.247852
C -1.689231 1.057139 -0.825372
C -3.905668 0.537081 0.788315
H -3.477505 -1.554766 0.428858
C -2.420416 2.106219 -0.274973
H -0.838458 1.254888 -1.470144
C -3.538662 1.865581 0.538436
H -2.123086 3.130868 -0.485607
C 0.089633 -1.490238 2.174694
H -0.936771 -1.104395 2.142526
H 0.168495 -2.101247 3.084601
C 1.065391 -0.288513 2.278778
H 0.730748 0.318722 3.133887
H 0.944323 0.349597 1.395692
C 2.505736 -0.652220 2.468229
C 3.566484 -0.631016 1.398585
H 3.666987 -1.630727 0.936537
H -4.773471 0.329773 1.409891
C -4.342601 3.013517 1.101351
H -5.048411 3.402807 0.355747
H -4.926373 2.704714 1.974223
H -3.697143 3.846520 1.400514
H 0.959910 -1.060535 -0.555272
H 2.779801 -1.132607 3.407046
C 3.360689 0.388391 0.291862
C 3.345490 1.761840 0.579023
C 3.176459 -0.013878 -1.037689
C 3.145250 2.701597 -0.430978
H 3.488523 2.091767 1.605555
C 2.966117 0.924884 -2.053503
H 3.206932 -1.073009 -1.286699
C 2.949640 2.286101 -1.751637
H 3.140758 3.761194 -0.188138
H 2.816032 0.587113 -3.075042
H 2.790155 3.019006 -2.537827
H -0.402746 -3.227360 0.962131
H 1.318091 -2.887925 1.058673
H 4.540311 -0.452312 1.879978
44
-1301.65877940
C -0.325711 1.010318 2.092684
N -0.349365 1.321450 0.662074
S 0.945878 1.963678 -0.163323
O 0.435955 2.218178 -1.513916
O 1.505480 3.016410 0.688515
C 2.228646 0.708615 -0.326204
C 3.256716 0.640637 0.615481
C 2.184176 -0.190023 -1.394962
C 4.231609 -0.349024 0.492841
H 3.302290 1.372080 1.415402
C 3.166389 -1.172302 -1.503112
H 1.405850 -0.097049 -2.145306
C 4.199577 -1.274175 -0.559407
H 3.138530 -1.863873 -2.341957
C 0.020194 -0.439912 2.469385
H 1.031282 -0.685837 2.121392
H 0.047508 -0.488798 3.566448
C -0.979011 -1.495792 1.951507
H -2.005100 -1.181509 2.194503
H -0.816688 -2.424014 2.533043
C -0.871389 -1.813638 0.492266
C -2.045924 -2.322979 -0.298906
H -2.482704 -3.208125 0.194623
H 5.037912 -0.395171 1.220961
C 5.243636 -2.359595 -0.671194
H 4.900146 -3.287951 -0.195507
H 5.466576 -2.595185 -1.717149
H 6.178319 -2.068940 -0.181166
H -0.937194 0.736159 0.074362
H 0.124777 -1.903628 0.063791
C -3.147488 -1.283246 -0.478325
C -2.961519 -0.198136 -1.349205
C -4.349325 -1.366838 0.234008
C -3.946694 0.778186 -1.498144
H -2.040169 -0.119392 -1.923875
C -5.339608 -0.392056 0.086340
H -4.514517 -2.206329 0.906092
C -5.140432 0.684149 -0.778443
H -3.780348 1.610812 -2.176025
H -6.266770 -0.476468 0.647488
H -5.908891 1.443640 -0.893401
H 0.377408 1.708451 2.555958
H -1.319337 1.261381 2.486431
H -1.701911 -2.661074 -1.283914
44
-1301.65870628
C -0.227933 -2.162952 -1.124878
N -0.418071 -1.652192 0.238479
S 0.906681 -1.497545 1.258237
O 0.389209 -0.801195 2.440022
O 1.538615 -2.816458 1.341075
C 2.088148 -0.407205 0.447833
C 3.226558 -0.948724 -0.149589
C 1.848376 0.969432 0.408763
C 4.129813 -0.100785 -0.790885
H 3.402420 -2.017659 -0.095359
C 2.758586 1.799937 -0.238706
H 0.964754 1.385164 0.882476
C 3.913292 1.281740 -0.845641
H 2.572262 2.870905 -0.270772

C -1.532520 -2.706163 -1.720606
H -1.284838 -3.114678 -2.708965
H -1.882493 -3.546097 -1.107824
C -2.667293 -1.673057 -1.875431
H -3.383770 -2.069371 -2.620592
H -2.272792 -0.750436 -2.331330
C -3.401718 -1.352878 -0.611223
C -4.146616 -0.061693 -0.422929
H -4.925371 -0.196356 0.338847
H 5.019409 -0.521905 -1.253098
C 4.908867 2.199014 -1.515124
H 4.407790 3.015698 -2.046144
H 5.580152 2.657108 -0.776761
H 5.533094 1.658218 -2.233389
H -1.012864 -0.827937 0.309587
H -3.475196 -2.122839 0.152301
C -3.248739 1.104439 -0.008241
C -3.085767 2.224169 -0.832556
C -2.562836 1.070865 1.216487
C -2.267405 3.288108 -0.444317
H -3.612205 2.268303 -1.783617
C -1.744037 2.131216 1.608196
H -2.671320 0.210973 1.873601
C -1.595427 3.246213 0.777908
H -2.159042 4.150516 -1.097243
H -1.224925 2.080562 2.561297
H -0.964641 4.076479 1.084614
H 0.191316 -1.390490 -1.789489
H 0.500629 -2.976141 -1.059738
H -4.665451 0.217212 -1.352672
44
-1301.65811993
C 1.571051 -1.673848 -1.407122
N 0.508921 -0.918728 -0.711582
S -0.573978 -1.779755 0.250973
O -0.792998 -3.060083 -0.433372
O -0.167390 -1.756974 1.666501
C -2.051827 -0.771807 0.115297
C -2.667430 -0.608327 -1.129201
C -2.598979 -0.204982 1.263840
C -3.835133 0.141201 -1.214503
H -2.231746 -1.059282 -2.014632
C -3.772651 0.544314 1.159880
H -2.109343 -0.355301 2.219881
C -4.407643 0.728328 -0.073319
H -4.201645 0.989145 2.054425
C 2.582960 -2.424498 -0.526516
H 2.072430 -3.250741 -0.018774
H 3.323857 -2.881251 -1.198646
C 3.307032 -1.558378 0.523289
H 3.952468 -2.233237 1.115307
H 2.571673 -1.171003 1.240634
C 4.125931 -0.441590 -0.041304
C 4.179057 0.920679 0.594141
H 5.062213 1.465047 0.238570
H -4.314608 0.273792 -2.181604
C -5.687437 1.522761 -0.181956
H -6.542648 0.866754 -0.389333
H -5.903236 2.064800 0.743773
H -5.637235 2.252427 -0.998487
H 0.854712 -0.106277 -0.199881

44
-1301.65547281
C -0.823534 2.600894 -1.241990
C -2.260566 2.479897 -0.689187
C -2.359789 2.169015 0.773717
C -3.570989 1.507983 1.375341
C -0.068468 1.271577 -1.358188
N 0.221820 0.728661 -0.019149
H -0.240793 3.296273 -0.623763
H -0.872517 3.036569 -2.247727
H -2.814448 1.729961 -1.269829
H -2.773448 3.441281 -0.886263
H -1.712665 2.732758 1.446340

H 4.709493 -0.623970 -0.941838
C 2.928444 1.745795 0.298178
C 2.782996 2.401223 -0.933790
C 1.879497 1.829892 1.225001
C 1.625768 3.120369 -1.231240
H 3.588155 2.348280 -1.663553
C 0.714522 2.544391 0.926913
H 1.976103 1.337286 2.189813
C 0.584350 3.191442 -0.302394
H 1.535771 3.625449 -2.189518
H -0.088011 2.594840 1.657951
H -0.319643 3.747333 -0.534951
H 1.076867 -2.379092 -2.079900
H 2.082511 -0.927906 -2.025229
H 4.283121 0.824749 1.685300
44
-1301.65810374
C 0.291991 -1.664542 -1.241717
N 0.358347 -1.072888 0.099593
S -0.918634 -1.242055 1.167658
O -1.261554 -2.665773 1.188895
O -0.506603 -0.511952 2.370348
C -2.326467 -0.369375 0.466332
C -3.253303 -1.061965 -0.314299
C -2.464758 1.003972 0.686195
C -4.321538 -0.367279 -0.881822
H -3.146663 -2.132555 -0.453353
C -3.537371 1.681086 0.112784
H -1.748397 1.525476 1.312726
C -4.481776 1.009430 -0.678952
H -3.648659 2.748733 0.287237
C 1.612576 -1.476866 -1.996016
H 1.447283 -1.836436 -3.020696
H 1.839512 -0.406007 -2.078638
C 2.839769 -2.229366 -1.407197
H 3.642572 -2.159540 -2.157266
H 2.575234 -3.293627 -1.331304
C 3.355947 -1.748977 -0.086771
C 4.279992 -0.570081 0.044090
H 4.983642 -0.551240 -0.800059
H -5.046170 -0.906677 -1.487013
C -5.658344 1.750175 -1.268625
H -5.375772 2.757004 -1.594659
H -6.077151 1.218560 -2.128853
H -6.462476 1.863051 -0.529543
H 0.752020 -0.134383 0.148426
H 2.911200 -2.152373 0.817710
C 3.565636 0.782299 0.105045
C 2.707712 1.090298 1.173717
C 3.735795 1.736060 -0.905918
C 2.037533 2.313849 1.223570
H 2.552378 0.364520 1.968107
C 3.068717 2.963579 -0.857736
H 4.402053 1.518136 -1.738077
C 2.215622 3.256291 0.206182
H 1.379816 2.528623 2.061818
H 3.218700 3.689774 -1.652749
H 1.696894 4.210390 0.246324
H 0.072926 -2.729408 -1.115400
H -0.524752 -1.224339 -1.836564
H 4.884376 -0.684335 0.955035

H -0.602759 0.740409 0.586896
H -4.431667 2.200858 1.334527
H -3.385391 1.331964 2.442787
H 0.889001 1.437799 -1.865058
H -0.644229 0.559959 -1.965092
S 0.900023 -0.814772 0.082918
O 0.609688 -1.604942 -1.121547
O 0.513400 -1.300321 1.415492
C 2.660032 -0.472933 0.080047
C 3.427648 -0.828130 -1.026618
C 3.245480 0.127385 1.198755
C 4.799636 -0.568738 -1.013315
H 2.953891 -1.307446 -1.876482

C 4.612961 0.377275 1.194818
H 2.635028 0.386071 2.057667
C 5.412026 0.034357 0.090763
H 5.402080 -0.844567 -1.875234
H 5.072694 0.843458 2.063184
C 6.898918 0.297811 0.111893
H 7.406628 -0.361650 0.827381
H 7.350096 0.130499 -0.870787
H 7.117629 1.328611 0.414194
C -3.973125 0.202269 0.704346
C -5.130458 0.118100 -0.078280
C -3.176586 -0.943755 0.847906
C -5.486018 -1.078080 -0.706705
H -5.762087 0.996585 -0.194541
C -3.526191 -2.138349 0.218646
H -2.275021 -0.909747 1.455755
C -4.683174 -2.209370 -0.561309
H -6.390169 -1.123186 -1.308631
H -2.890708 -3.011409 0.337631
H -4.955770 -3.140457 -1.050991
44
-1301.65511866
C 2.329014 -3.163603 -0.237580
C 3.388975 -2.066051 -0.461493
C 3.006538 -0.706080 0.036391
C 3.675400 0.542211 -0.463665
C 1.047662 -2.994085 -1.070089
N 0.211941 -1.816135 -0.753328
H 2.070075 -3.217696 0.826800
H 2.768771 -4.134365 -0.503124
H 3.646728 -2.014753 -1.531439
H 4.320771 -2.393279 0.039931
H 2.468375 -0.642986 0.981430
H 0.777798 -0.964050 -0.705143
H 3.927887 0.424100 -1.527609
H 4.642472 0.691001 0.051589
H 0.406136 -3.872313 -0.964074
H 1.301573 -2.903701 -2.134048
S -0.722394 -1.908708 0.662834
O 0.028731 -1.412596 1.828813
O -1.307918 -3.254395 0.680759
C -2.004793 -0.710633 0.303857
C -1.867745 0.600910 0.756787
C -3.129584 -1.109583 -0.421623
C -2.870824 1.525287 0.464783
H -0.994860 0.888830 1.332774
C -4.122075 -0.174348 -0.698241
H -3.221938 -2.138739 -0.752224
C -4.009875 1.155132 -0.261259
H -2.766416 2.549918 0.812792
H -5.001184 -0.480061 -1.260625
C -5.108369 2.151926 -0.545216
H -4.748454 3.181846 -0.457586
H -5.519703 2.019079 -1.551848
H -5.939873 2.033207 0.161941
C 2.819861 1.782126 -0.269135
C 3.024790 2.640688 0.817022
C 1.773108 2.067510 -1.157295
C 2.206054 3.754640 1.014679
H 3.833565 2.435214 1.514901
C 0.952702 3.179548 -0.965229
H 1.604798 1.415507 -2.012429
C 1.166965 4.027513 0.124008
H 2.383007 4.410196 1.863450
H 0.149618 3.385656 -1.668059
H 0.532248 4.896935 0.273759
44
-1301.65510882
C 2.327878 -3.164349 -0.237610
C 3.388202 -2.067174 -0.461639
C 3.006283 -0.707065 0.036251
C 3.675672 0.540955 -0.463745
C 1.046544 -2.994443 -1.070068
N 0.211203 -1.816240 -0.753302
H 2.068972 -3.218299 0.826786
H 2.767286 -4.135276 -0.503126
H 3.645880 -2.015987 -1.531610
H 4.319938 -2.394718 0.039695
H 2.468079 -0.643780 0.981254
H 0.777261 -0.964288 -0.705244
H 3.928087 0.422800 -1.527705
H 4.642828 0.689305 0.051482
H 0.404753 -3.872473 -0.964017
H 1.300448 -2.904164 -2.134037
S -0.723125 -1.908462 0.662871
O 0.028165 -1.412500 1.828802
O -1.309045 -3.253978 0.680884
C -2.005187 -0.710037 0.303864
C -1.867523 0.601623 0.756314
C -3.130315 -1.108811 -0.421163
C -2.870320 1.526269 0.464263
H -0.994384 0.889416 1.331982
C -4.122540 -0.173287 -0.697833
H -3.223140 -2.138047 -0.751384
C -4.009719 1.156290 -0.261352
H -2.765436 2.550982 0.811896
H -5.001920 -0.478867 -1.259862
C -5.107895 2.153440 -0.545289
H -4.747034 3.183237 -0.460050
H -5.520951 2.019153 -1.551017
H -5.938330 2.036639 0.163441
C 2.820686 1.781240 -0.269129
C 3.026216 2.639825 0.816895
C 1.773848 2.066962 -1.157079
C 2.207986 3.754137 1.014621
H 3.835063 2.434086 1.514611
C 0.953946 3.179361 -0.964944
H 1.605081 1.414944 -2.012113
C 1.168812 4.027351 0.124154
H 2.385398 4.409707 1.863285
H 0.150796 3.385735 -1.667620
H 0.534498 4.897059 0.273958
44
-1301.65509019
C 0.467778 3.092976 -1.182346
C 1.946374 3.038631 -0.738492
C 2.585792 1.684389 -0.808051
C 3.737080 1.313955 0.081998
C -0.515734 2.468885 -0.186238
N -0.328553 1.008685 -0.120595
H 0.353047 2.610798 -2.161830
H 0.178235 4.143195 -1.311186
H 2.034596 3.442286 0.281036
H 2.512987 3.740652 -1.379517
H 2.449619 1.107239 -1.722930
H 0.658915 0.737655 -0.114674
H 3.492964 1.588879 1.120373
H 4.618779 1.931217 -0.173153
H -1.543628 2.660570 -0.515603
H -0.395108 2.933530 0.801781
S -1.100822 0.164595 1.120609
O -1.395200 1.051533 2.254911
O -0.293219 -1.049815 1.303243
C -2.668180 -0.289752 0.380016
C -3.843108 0.299874 0.840434
C -2.694494 -1.248245 -0.637598
C -5.059938 -0.071629 0.265088
H -3.799124 1.029847 1.641451
C -3.915155 -1.605994 -1.198258
H -1.771131 -1.706518 -0.976352
C -5.116526 -1.025007 -0.757490
H -5.979245 0.386201 0.621682
H -3.940538 -2.351126 -1.989968
C -6.434701 -1.439381 -1.366830
H -6.394398 -1.412349 -2.461982
H -6.696804 -2.465830 -1.079983
H -7.250054 -0.786048 -1.041948
C 4.123880 -0.151788 0.008767
C 3.282271 -1.138867 0.542426
C 5.315297 -0.549638 -0.608858

C 3.626931 -2.488006 0.457836
H 2.350567 -0.860299 1.029616
C 5.663335 -1.899458 -0.694013
H 5.979107 0.205168 -1.025728
C 4.818814 -2.873269 -0.160310
H 2.961309 -3.236995 0.878463
H 6.594452 -2.187654 -1.175464
H 5.087543 -3.924537 -0.223398
44
-1301.65506872
C 0.701580 0.106319 -2.033594
C 2.075186 0.460207 -1.427769
C 2.319577 -0.066047 -0.046358
C 3.393896 0.506114 0.837701
C -0.485900 0.843286 -1.404302
N -0.720236 0.376544 -0.026574
H 0.531823 -0.976172 -1.969132
H 0.715261 0.361470 -3.100616
H 2.208200 1.553008 -1.438879
H 2.854623 0.068598 -2.108371
H 2.000122 -1.088384 0.160278
H 0.128759 0.395493 0.544842
H 3.177502 0.267139 1.886640
H 3.404196 1.600595 0.751736
H -1.393086 0.630312 -1.981201
H -0.322218 1.929415 -1.441553
S -1.945449 1.135844 0.858116
O -2.147166 2.519838 0.405298
O -1.628503 0.841085 2.260186
C -3.398078 0.203257 0.374561
C -4.379061 0.811776 -0.405061
C -3.542334 -1.117394 0.810294
C -5.515088 0.081449 -0.759720
H -4.252476 1.842736 -0.717466
C -4.680136 -1.829222 0.447958
H -2.774319 -1.571975 1.427390
C -5.684216 -1.243100 -0.341516
H -6.282808 0.552880 -1.368234
H -4.796533 -2.856771 0.784580
C -6.922460 -2.025687 -0.708882
H -6.667033 -3.017457 -1.099697
H -7.565301 -2.180962 0.167095
H -7.515838 -1.505507 -1.466880
C 4.775287 -0.038015 0.476804
C 5.222873 -1.255428 1.007565
C 5.604956 0.644123 -0.422614
C 6.466326 -1.777386 0.650730
H 4.593141 -1.795136 1.711922
C 6.849648 0.124643 -0.782878
H 5.277923 1.595795 -0.836165
C 7.284110 -1.089082 -0.248027
H 6.798925 -2.719744 1.078485
H 7.482101 0.672120 -1.477108
H 8.254226 -1.493029 -0.524726
44
-1301.65498054
C -0.888601 -2.470424 1.444653
C -2.367526 -2.270497 1.057101
C -2.593986 -1.931241 -0.383277
C -3.832710 -1.214767 -0.844681
C -0.084585 -1.171606 1.623752
N -0.030207 -0.241010 0.484225
H -0.395739 -3.103276 0.699657
H -0.837692 -3.005273 2.403037
H -2.828101 -1.508004 1.705342
H -2.906070 -3.207815 1.297266
H -1.928623 -2.368456 -1.123313
H -0.939604 0.083059 0.155303
H -4.734308 -1.726225 -0.466941
H -3.889806 -1.257059 -1.938899
H 0.948970 -1.409091 1.899730
H -0.501429 -0.589189 2.454960
S 0.919107 -0.596376 -0.856801
O 0.405483 0.283764 -1.915577
O 1.036708 -2.044459 -1.090324
C 2.537062 -0.023516 -0.334876
C 2.718526 1.321290 0.003770
C 3.607735 -0.914249 -0.325693
C 3.986042 1.764356 0.362675
H 1.874972 2.003543 -0.009799
C 4.874292 -0.451570 0.037497
H 3.444072 -1.950682 -0.600033
C 5.084167 0.887191 0.384946
H 4.130404 2.808624 0.629906
H 5.711385 -1.145100 0.048413
C 6.455713 1.389915 0.767866
H 7.180091 0.572261 0.829081
H 6.830671 2.113993 0.033603
H 6.437144 1.898324 1.739264
C -3.888836 0.242779 -0.395315
C -4.792417 0.667420 0.585587
C -3.009784 1.187534 -0.950337
C -4.821803 1.999869 1.006213
H -5.485250 -0.050502 1.019638
C -3.037688 2.518630 -0.531969
H -2.299994 0.879315 -1.715411
C -3.944040 2.929725 0.449039
H -5.533197 2.309280 1.767875
H -2.352291 3.235120 -0.977231
H -3.966767 3.966753 0.773288
44
-1301.65436766
C -0.847234 2.158734 0.195377
C -2.052002 1.828924 1.108302
C -2.298720 0.371319 1.379722
C -2.663334 -0.595680 0.281892
C 0.520220 1.831749 0.812900
N 0.778330 0.405669 1.088009
H -0.865159 3.235732 -0.020381
H -0.936059 1.643825 -0.766320
H -2.950587 2.261335 0.631744
H -1.934399 2.363429 2.060577
H -2.584978 0.090246 2.392631
H -0.013122 -0.055135 1.545137
H -2.439825 -1.619792 0.605288
H -2.056556 -0.412241 -0.612666
H 0.634091 2.354005 1.771678
H 1.324149 2.192802 0.163145
S 1.275244 -0.592788 -0.179816
O 0.787335 -0.109973 -1.482255
O 0.956042 -1.950904 0.276117
C 3.052713 -0.356965 -0.170446
C 3.681974 0.153603 -1.303246
C 3.792584 -0.734137 0.954772
C 5.070987 0.297489 -1.302326
H 3.087865 0.428747 -2.167882
C 5.174219 -0.584199 0.937695
H 3.288083 -1.135066 1.827692
C 5.836201 -0.067314 -0.189420
H 5.565198 0.697920 -2.183937
H 5.753095 -0.873766 1.811609
C 7.339583 0.074928 -0.194054
H 7.691568 0.620019 0.689708
H 7.829164 -0.907145 -0.181932
H 7.689347 0.609566 -1.082257
C -4.142292 -0.489885 -0.081912
C -5.115727 -1.148114 0.682711
C -4.562759 0.297841 -1.161520
C -6.470948 -1.023519 0.377744
H -4.806233 -1.769693 1.520488
C -5.918787 0.426613 -1.469151
H -3.820181 0.802910 -1.775485
C -6.877886 -0.232766 -0.699417
H -7.209929 -1.547956 0.978355
H -6.224114 1.037181 -2.315215
H -7.933400 -0.136689 -0.939626
44
-1301.65421074
C 0.464731 3.046852 0.067849
C 1.842874 3.044997 -0.639002

C 2.560004 1.726638 -0.698841
C 3.060437 1.052890 0.550669
C -0.634464 2.294369 -0.694780
N -0.454581 0.835347 -0.819196
H 0.136864 4.089902 0.175165
H 0.547162 2.636011 1.079149
H 2.478514 3.773204 -0.102632
H 1.719529 3.444288 -1.654779
H 2.969669 1.396899 -1.651097
H 0.495025 0.570021 -1.091381
H 2.217266 0.780698 1.204765
H 3.645383 1.783878 1.138801
H -0.714612 2.682795 -1.718334
H -1.606677 2.464211 -0.220552
S -0.916412 -0.149210 0.469581
O -0.773548 0.532993 1.765817
O -0.224519 -1.422888 0.227288
C -2.672256 -0.356004 0.175125
C -3.578973 0.027507 1.160504
C -3.108897 -0.949093 -1.013811
C -4.943158 -0.178034 0.944934
H -3.215123 0.477337 2.077869
C -4.470592 -1.145849 -1.210745
H -2.389567 -1.244889 -1.770276
C -5.409404 -0.765367 -0.236212
H -5.654100 0.122498 1.710415
H -4.815043 -1.603708 -2.135036
C -6.883739 -0.999756 -0.463365
H -7.489055 -0.541364 0.324324
H -7.209848 -0.586128 -1.424950
H -7.114851 -2.072365 -0.480028
C 3.913683 -0.170700 0.277969
C 5.292495 -0.041146 0.064540
C 3.338529 -1.446257 0.201146
C 6.081866 -1.156532 -0.218751
H 5.752521 0.943296 0.126129
C 4.126843 -2.563420 -0.082831
H 2.270377 -1.567078 0.365517
C 5.499624 -2.423294 -0.293551
H 7.150924 -1.036605 -0.376204
H 3.664940 -3.546084 -0.135122
H 6.112290 -3.294557 -0.510597
44
-1301.65388478
C -0.431841 3.198979 0.087212
C -1.872520 3.005182 -0.430001
C -2.515836 1.702065 -0.060930
C -3.672979 1.170664 -0.851172
C 0.623247 2.370292 -0.660045
N 0.464584 0.907301 -0.570318
H -0.380454 2.965363 1.156542
H -0.156613 4.256554 -0.022345
H -1.895853 3.141984 -1.522741
H -2.486572 3.833419 -0.026586
H -2.395250 1.355408 0.964837
H -0.507127 0.609017 -0.680453
H -3.389249 1.123730 -1.917609
H -4.501804 1.903783 -0.830314
H 1.624753 2.618854 -0.295436
H 0.606235 2.618904 -1.729107
S 1.083467 0.100571 0.777196
O 0.290037 -1.133755 0.858917
O 1.202592 0.995085 1.938199
C 2.750366 -0.303728 0.255152
C 3.832543 0.192506 0.977877
C 2.944656 -1.149187 -0.841668
C 5.127247 -0.156318 0.587912
H 3.656239 0.838007 1.831402
C 4.240322 -1.485870 -1.215202
H 2.091241 -1.531996 -1.391615
C 5.352052 -0.996764 -0.507667
H 5.974399 0.231086 1.148325
H 4.395994 -2.140832 -2.069267
C 6.751700 -1.386262 -0.919537
H 7.505474 -0.814436 -0.370049
H 6.937286 -2.451005 -0.729024
H 6.913763 -1.218763 -1.990785
C -4.215081 -0.179215 -0.411058
C -3.373919 -1.193509 0.066941
C -5.588149 -0.443332 -0.507006
C -3.894181 -2.435798 0.435324
H -2.305430 -1.021528 0.166895
C -6.110150 -1.684921 -0.143731
H -6.256804 0.335086 -0.869594
C -5.262577 -2.687769 0.330099
H -3.223153 -3.205198 0.807975
H -7.179143 -1.865520 -0.224604
H -5.665920 -3.654894 0.618711
44
-1301.65299069
C -1.435784 -3.299440 -1.431211
C -2.811849 -2.636944 -1.198644
C -2.805652 -1.139316 -1.164724
C -3.813747 -0.349393 -0.380674
C -0.447691 -3.151815 -0.268557
N 0.054599 -1.763278 -0.199227
H -0.976734 -2.904635 -2.346740
H -1.593657 -4.373110 -1.593502
H -3.246861 -3.026572 -0.266583
H -3.487412 -2.985058 -2.003409
H -2.271387 -0.615544 -1.958334
H -0.713332 -1.086936 -0.211250
H -4.075885 -0.902161 0.532689
H -4.756548 -0.263867 -0.952185
H 0.416260 -3.802365 -0.421932
H -0.931117 -3.467045 0.668243
S 1.083251 -1.389808 1.094762
O 1.808380 -2.621072 1.426655
O 0.327672 -0.656743 2.124271
C 2.233358 -0.233813 0.351107
C 3.456114 -0.704808 -0.127659
C 1.902805 1.119942 0.270661
C 4.351088 0.195363 -0.702054
H 3.701923 -1.757006 -0.032452
C 2.808172 2.003597 -0.313059
H 0.958124 1.477742 0.667359
C 4.041191 1.558354 -0.810557
H 5.309462 -0.166088 -1.067062
H 2.555779 3.059391 -0.375388
C 5.004336 2.520824 -1.463953
H 6.042636 2.197427 -1.336987
H 4.815638 2.594405 -2.543328
H 4.908228 3.529068 -1.047998
C -3.331608 1.045608 -0.018940
C -3.767253 2.168739 -0.731302
C -2.413719 1.227797 1.026089
C -3.299741 3.445786 -0.412060
H -4.482912 2.043851 -1.541414
C -1.947065 2.502860 1.349550
H -2.058104 0.372323 1.595286
C -2.388052 3.616680 0.630252
H -3.652744 4.306022 -0.975161
H -1.245085 2.622194 2.170876
H -2.028307 4.610232 0.884668

N-Cl

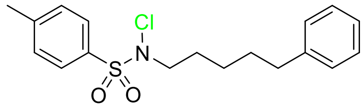

Name	E(B3LYP)	G(B3LYP)	E(RO-B2PLYP-D3)	G(RO-B2PLYP-D3)
Conformers				
Tosyl_NCl_pentane_Ph_0010_cregen_B2PLYP.log	-1762.654524	-1762.260835	-1762.076081	-1761.76519811
Tosyl_NCl_pentane_Ph_0002_cregen_B2PLYP.log	-1762.653206	-1762.259573	-1762.075911	-1761.76439085
Tosyl_NCl_pentane_Ph_0000_cregen_B2PLYP.log	-1762.653206	-1762.259574	-1762.075914	-1761.76438531
Tosyl_NCl_pentane_Ph_0001_cregen_B2PLYP.log	-1762.653205	-1762.259574	-1762.075909	-1761.76436441
Tosyl_NCl_pentane_Ph_0015_cregen_B2PLYP.log	-1762.654524	-1762.260835	-1762.075191	-1761.76431286
Tosyl_NCl_pentane_Ph_0013_cregen_B2PLYP.log	-1762.651287	-1762.257565	-1762.073422	-1761.76193400
Tosyl_NCl_pentane_Ph_0005_cregen_B2PLYP.log	-1762.656749	-1762.262956	-1762.073468	-1761.76145058
Tosyl_NCl_pentane_Ph_0008_cregen_B2PLYP.log	-1762.656748	-1762.263899	-1762.076075	-1761.76138921
Tosyl_NCl_pentane_Ph_0007_cregen_B2PLYP.log	-1762.656751	-1762.262957	-1762.073481	-1761.76090789
Tosyl_NCl_pentane_Ph_0012_cregen_B2PLYP.log	-1762.656748	-1762.262954	-1762.073396	-1761.76083510

45
-1762.338036
C 0.677801 -2.276919 0.009619
N -0.324500 -1.181670 -0.107329
S -1.977798 -1.801508 -0.185410
O -2.269335 -2.432571 -1.475195
O -2.091779 -2.559176 1.065045
C -2.917067 -0.290363 -0.051468
C -3.634851 0.167212 -1.155172
C -2.957665 0.381372 1.172890
C -4.398935 1.326139 -1.026852
H -3.590846 -0.378384 -2.091278
C -3.722786 1.538202 1.278338
H -2.401346 0.000655 2.022642
C -4.455724 2.027114 0.184834
H -3.756628 2.070149 2.225886
C 2.037924 -1.781858 0.510549
H 2.686841 -2.668050 0.506171
H 2.475125 -1.086115 -0.212090
C 2.018482 -1.168779 1.927711
H 2.974414 -1.390279 2.421526
H 1.253298 -1.690610 2.517719
C 1.763558 0.352082 2.011429
C 3.032956 1.234036 1.947186
H 3.691981 0.956959 2.780772
H -4.959749 1.691169 -1.883422
C -5.306398 3.266525 0.324604
H -4.816440 4.019760 0.950874
H -5.520317 3.718481 -0.648780
H -6.269138 3.029528 0.796246
Cl 0.020536 -0.184083 -1.543431
H 1.271594 0.573471 2.968027
C 3.819691 1.170995 0.653211
C 5.041411 0.489162 0.579249
C 3.342290 1.799689 -0.507078
C 5.760872 0.424738 -0.616268
H 5.436299 0.006478 1.470780
C 4.056397 1.739011 -1.703357
H 2.399522 2.341269 -0.470847
C 5.269625 1.048659 -1.763086
H 6.707016 -0.109689 -0.648554
H 3.668157 2.234916 -2.589447
H 5.828115 1.002797 -2.694232
H 0.779093 -2.808945 -0.944709
H 0.246317 -2.966090 0.742364
H 1.057856 0.655512 1.230501
H 2.729420 2.274129 2.129190
45
-1762.338420
C -0.676189 -2.277723 0.008073
N 0.326007 -1.182226 -0.107712
S 1.979319 -1.801974 -0.186167
O 2.093201 -2.560697 1.063674
O 2.270892 -2.431982 -1.476471
C 2.917697 -0.290342 -0.051127
C 2.959364 0.379748 1.174535
C 3.639500 0.165138 -1.152587
C 3.727986 1.533558 1.282358
H 2.405707 -0.003764 2.024767
C 4.407463 1.321722 -1.021833
H 3.600381 -0.382868 -2.087504
C 4.460517 2.024074 0.188721
H 4.976052 1.681955 -1.875216
C -2.036517 -1.783245 0.508972
H -2.473599 -1.087040 -0.213293
H -2.685329 -2.669506 0.503774
C -2.017495 -1.171158 1.926572
H -1.252145 -1.693067 2.516292
H -2.973378 -1.393411 2.420139
C -1.763220 0.349752 2.011344
C -3.032974 1.231216 1.947588
H -2.729938 2.271272 2.130656
H 3.767576 2.060532 2.232525
C 5.275169 3.288438 0.319423
H 4.626023 4.173439 0.339009
H 5.856361 3.294653 1.248288
H 5.970050 3.406717 -0.517322
Cl -0.018903 -0.183469 -1.543059
H -1.057581 0.654013 1.230685
C -3.819350 1.169140 0.653340
C -3.341700 1.798883 -0.506276
C -5.041029 0.487338 0.578468
C -4.055541 1.739320 -1.702769
H -2.398923 2.340406 -0.469346
C -5.760224 0.424016 -0.617267
H -5.436095 0.003798 1.469455
C -5.268756 1.049028 -1.763394
H -3.667094 2.236022 -2.588320
H -6.706342 -0.110412 -0.650259
H -5.827065 1.004042 -2.694691
H -0.244816 -2.967401 0.740399
H -0.777082 -2.809013 -0.946708
H -1.271409 0.570690 2.968118
H -3.692115 0.953107 2.780736
45
-1762.339386

C 0.461714 -1.521970 1.766940
N -0.609392 -0.647489 1.213765
S -1.209999 -1.001440 -0.382174
O -0.451436 -0.335658 -1.447189
O -1.346095 -2.461582 -0.359222
C -2.826840 -0.245374 -0.298309
C -3.808902 -0.822958 0.511466
C -3.100381 0.873839 -1.081247
C -5.076989 -0.254178 0.539332
H -3.580573 -1.704117 1.101460
C -4.379800 1.428536 -1.040188
H -2.324672 1.293997 -1.711642
C -5.382396 0.880138 -0.231553
H -4.600715 2.301050 -1.649495
C 1.884748 -1.423481 1.196117
H 2.151763 -0.370522 1.064109
H 2.556078 -1.807750 1.977669
C 2.132758 -2.226159 -0.091894
H 1.485156 -1.865941 -0.898376
H 1.833947 -3.266777 0.094315
C 3.593851 -2.215185 -0.586338
C 3.993735 -1.020927 -1.485382
H 3.286685 -0.966410 -2.322778
H -5.846557 -0.698561 1.165787
C -6.760831 1.493442 -0.175151
H -6.899745 2.242047 -0.960757
H -7.540912 0.732126 -0.289473
H -6.932106 1.987874 0.789648
Cl -0.240962 1.072651 1.406573
H 3.761558 -3.125858 -1.176289
C 4.071569 0.332275 -0.807248
C 3.061887 1.289762 -0.975543
C 5.169294 0.660617 0.002396
C 3.143620 2.535333 -0.348632
H 2.197311 1.051809 -1.590714
C 5.254829 1.903024 0.630375
H 5.969707 -0.064977 0.135413
C 4.240113 2.847019 0.456168
H 2.348428 3.262373 -0.492830
H 6.116763 2.137266 1.250337
H 4.306534 3.817628 0.940856
H 0.060315 -2.535047 1.663860
H 0.465785 -1.277370 2.833887
H 4.281205 -2.277819 0.269606
H 4.974517 -1.249231 -1.924475
45
-1762.339552
C 0.595297 -0.694402 -0.842339
N -0.244763 -1.748456 -0.227147
S -1.221194 -1.252498 1.133494
O -1.974318 -2.430760 1.545461
O -0.243075 -0.603293 2.011181
C -2.374756 -0.012592 0.555363
C -1.988481 1.330208 0.526006
C -3.646906 -0.406232 0.132632
C -2.891630 2.282515 0.059187
H -1.007551 1.623413 0.884576
C -4.534513 0.562411 -0.329329
H -3.935800 -1.449783 0.187956
C -4.172130 1.916310 -0.380057
H -5.528931 0.262261 -0.650042
C 1.791046 -1.280136 -1.598039
H 1.433754 -1.795081 -2.498489
H 2.381646 -0.424096 -1.945299
C 2.663467 -2.253665 -0.787172
H 3.489579 -2.582698 -1.433695
H 2.068603 -3.149545 -0.572725
C 3.246205 -1.750934 0.546697
C 4.302231 -0.623775 0.459578
H 5.024476 -0.867024 -0.331162
H -2.599760 3.329607 0.044001
C -5.132856 2.952878 -0.910918
H -5.030194 3.060838 -1.998870
H -6.172692 2.676521 -0.708709
H -4.948014 3.936261 -0.467008
Cl -1.198942 -2.607654 -1.447675
H 3.728184 -2.611844 1.028198
C 3.778657 0.784971 0.243302
C 2.919703 1.382238 1.180454
C 4.166653 1.542478 -0.869997
C 2.459647 2.687968 1.003698
H 2.606946 0.821031 2.057855
C 3.707950 2.849440 -1.052109
H 4.841325 1.103429 -1.602175
C 2.850778 3.427401 -0.115477
H 1.802507 3.131086 1.748112
H 4.024740 3.415386 -1.924570
H 2.496137 4.445708 -0.251513
H 0.950239 -0.090974 -0.004582
H -0.001739 -0.050144 -1.502021
H 2.435890 -1.453634 1.223393
H 4.872108 -0.631500 1.399358
45
-1762.343725
C -0.262183 1.801005 -1.046629
N 1.115732 1.357505 -0.712477
S 1.199468 0.136597 0.544912
O 1.172772 0.702781 1.895937
O 0.175592 -0.826399 0.117833
C 2.823573 -0.539238 0.241646
C 3.827838 -0.346318 1.188029
C 3.046566 -1.294637 -0.912929
C 5.081574 -0.914783 0.963710
H 3.623554 0.233174 2.081492
C 4.304133 -1.850609 -1.118344
H 2.246471 -1.445949 -1.629543
C 5.340844 -1.668048 -0.187873
H 4.485822 -2.441044 -2.012983
C -1.042132 2.570944 0.024977
H -1.025467 2.010686 0.965612
H -0.533740 3.521145 0.225124
C -2.495753 2.856508 -0.397783
H -2.492163 3.460950 -1.316374
H -2.956474 3.486096 0.375475
C -3.380135 1.616635 -0.626371
C -3.550066 0.716815 0.615906
H -4.008635 1.306503 1.421776
H 5.869586 -0.770935 1.698302
C 6.705993 -2.262139 -0.438022
H 7.245478 -1.695126 -1.207736
H 7.319133 -2.255183 0.468073
H 6.632581 -3.296506 -0.791888
Cl 2.188757 2.725600 -0.349641
H -2.983045 1.009682 -1.450773
C -4.386373 -0.512892 0.331685
C -3.772050 -1.723223 -0.019138
C -5.786290 -0.464153 0.377386
C -4.536080 -2.852030 -0.319655
H -2.685570 -1.778390 -0.047754
C -6.553682 -1.590561 0.078129
H -6.279465 0.465177 0.656478
C -5.929965 -2.789440 -0.272982
H -4.041084 -3.783032 -0.584594
H -7.638451 -1.533479 0.124133
H -6.525745 -3.669001 -0.502874
H -0.770021 0.872605 -1.318317
H -0.157046 2.396290 -1.960269
H -4.371561 1.950665 -0.959730
H -2.566422 0.398315 0.978403
45
-1762.343831
C -0.263380 1.802673 -1.046546
N 1.114928 1.359920 -0.713000
S 1.199812 0.138162 0.543472
O 1.172898 0.703318 1.894926
O 0.176632 -0.825340 0.115858
C 2.824089 -0.537039 0.239962
C 3.829484 -0.340780 1.184459
C 3.047976 -1.289969 -0.916055
C 5.084723 -0.904938 0.957679

H 3.626361 0.242056 2.076007
C 4.307043 -1.841594 -1.123934
H 2.248954 -1.438276 -1.634490
C 5.343284 -1.660260 -0.192706
H 4.491101 -2.425443 -2.022410
C -1.043558 2.571465 0.025721
H -1.026099 2.010764 0.966078
H -0.535852 3.521974 0.226145
C -2.497559 2.856076 -0.396370
H -2.494787 3.460877 -1.314728
H -2.958471 3.485011 0.377306
C -3.381073 1.615612 -0.625093
C -3.549833 0.715203 0.616917
H -4.008485 1.304260 1.423202
H 5.875116 -0.754272 1.688344
C 6.697256 -2.286664 -0.424171
H 7.455134 -1.861933 0.240827
H 6.665413 -3.368722 -0.242218
H 7.031836 -2.143416 -1.457665
Cl 2.187068 2.728490 -0.349492
H -2.983830 1.009270 -1.449871
C -4.385377 -0.514999 0.332602
C -5.785307 -0.467300 0.378998
C -3.770342 -1.724744 -0.018996
C -6.552024 -1.594149 0.079675
H -6.279024 0.461563 0.658690
C -4.533696 -2.853990 -0.319583
H -2.683836 -1.779100 -0.048178
C -5.927604 -2.792436 -0.272206
H -7.636812 -1.537874 0.126225
H -4.038151 -3.784526 -0.585137
H -6.522854 -3.672342 -0.502154
H -0.770667 0.874093 -1.318650
H -0.158877 2.398570 -1.959859
H -4.372882 1.949006 -0.957949
H -2.565804 0.397282 0.978879
45
-1762.339499
C -0.598048 -0.696637 -0.841507
N 0.241839 -1.749944 -0.224783
S 1.221033 -1.251275 1.132969
O 0.244858 -0.599076 2.010608
O 1.973848 -2.429150 1.546614
C 2.375210 -0.013561 0.551403
C 3.644644 -0.409878 0.122961
C 1.988963 1.329144 0.516841
C 4.530442 0.556276 -0.347549
H 3.930476 -1.454498 0.173742
C 2.890338 2.278954 0.041623
H 1.006836 1.623480 0.871179
C 4.170797 1.910966 -0.396171
H 2.595940 3.325139 0.015378
C -1.795542 -1.283099 -1.593863
H -2.385673 -0.427357 -1.942629
H -1.440155 -1.800949 -2.493391
C -2.667895 -2.253382 -0.779006
H -2.073790 -3.149398 -0.563040
H -3.495408 -2.583162 -1.423352
C -3.247971 -1.746309 0.554399
C -4.302753 -0.618097 0.465821
H -4.871099 -0.622479 1.406549
H 5.520127 0.253130 -0.679950
C 5.151675 2.952712 -0.877833
H 4.638444 3.820391 -1.304492
H 5.774675 3.316059 -0.049851
H 5.826639 2.546494 -1.638105
Cl 1.193004 -2.613591 -1.444511
H -2.436269 -1.448170 1.229055
C -3.777812 0.789397 0.244750
C -2.916550 1.388222 1.178775
C -4.166750 1.544259 -0.870016
C -2.455193 2.692894 0.997588
H -2.602982 0.829082 2.057209
C -3.706756 2.850144 -1.056563
H -4.843171 1.103990 -1.599842
C -2.847309 3.429674 -0.122992
H -1.796278 3.137290 1.739670
H -4.024303 3.414016 -1.930090
H -2.491659 4.447165 -0.262468
H -0.001337 -0.054732 -1.503765
H -0.951181 -0.090719 -0.004783
H -3.730309 -2.605294 1.038962
H -5.026584 -0.862686 -0.323054
45
-1762.343722
C -0.262133 1.801139 -1.046631
N 1.115748 1.357562 -0.712472
S 1.199398 0.136556 0.544829
O 1.172643 0.702636 1.895892
O 0.175541 -0.826395 0.117620
C 2.823510 -0.539280 0.241602
C 3.827768 -0.346326 1.187988
C 3.046521 -1.294696 -0.912956
C 5.081512 -0.914774 0.963687
H 3.623467 0.233173 2.081444
C 4.304099 -1.850653 -1.118354
H 2.246429 -1.446046 -1.629566
C 5.340801 -1.668055 -0.187885
H 4.485801 -2.441106 -2.012979
C -1.042096 2.571005 0.025021
H -1.025414 2.010694 0.965625
H -0.533733 3.521209 0.225221
C -2.495726 2.856555 -0.397719
H -2.492147 3.461047 -1.316277
H -2.956460 3.486091 0.375574
C -3.380083 1.616675 -0.626371
C -3.549982 0.716798 0.615868
H -4.008519 1.306449 1.421785
H 5.869517 -0.770905 1.698283
C 6.705977 -2.262089 -0.438025
H 7.318961 -2.255494 0.468179
H 6.632603 -3.296315 -0.792306
H 7.245613 -1.694786 -1.207422
Cl 2.188789 2.725615 -0.349466
H -2.982980 1.009767 -1.450800
C -4.386301 -0.512905 0.331652
C -5.786225 -0.464078 0.377120
C -3.771993 -1.723322 -0.018892
C -6.553634 -1.590486 0.077918
H -6.279392 0.465323 0.655993
C -4.536044 -2.852133 -0.319353
H -2.685513 -1.778551 -0.047363
C -5.929930 -2.789458 -0.272906
H -7.638408 -1.533334 0.123733
H -4.041056 -3.783204 -0.584070
H -6.525725 -3.669022 -0.502749
H -0.770004 0.872785 -1.318421
H -0.156943 2.396503 -1.960213
H -4.371512 1.950713 -0.959714
H -2.566323 0.398288 0.978317
45
-1762.343660
C -1.626502 -2.916560 0.070639
N -1.601949 -1.465349 -0.267141
S -2.202752 -0.439115 1.034288
O -3.661008 -0.498232 1.163197
O -1.334575 -0.822087 2.153950
C -1.733315 1.175304 0.436734
C -0.380823 1.526874 0.400997
C -2.728262 2.078029 0.066031
C -0.029758 2.800915 -0.030822
H 0.383724 0.821342 0.706959
C -2.354659 3.352830 -0.358487
H -3.770737 1.783452 0.115229
C -1.007597 3.731713 -0.418853
H -3.124486 4.063843 -0.646958
C -0.620250 -3.710625 -0.773611
H -0.941189 -4.760529 -0.746938
H -0.700054 -3.396341 -1.821715
C 0.843249 -3.636468 -0.302918

H 1.439400 -4.301458 -0.944231
H 0.912662 -4.054523 0.712438
C 1.464738 -2.234053 -0.311329
C 2.957760 -2.228548 0.074134
H 3.524233 -2.837418 -0.642945
H 1.021786 3.075014 -0.063344
C -0.606392 5.105051 -0.900835
H -0.142813 5.052410 -1.894321
H -1.469153 5.774060 -0.972326
H 0.126336 5.565170 -0.228210
Cl -2.507460 -1.165197 -1.768818
H 0.919551 -1.588788 0.384766
C 3.528190 -0.826555 0.121751
C 4.194731 -0.277352 -0.981488
C 3.355842 -0.023451 1.259192
C 4.672452 1.034121 -0.954568
H 4.342662 -0.886027 -1.871190
C 3.830470 1.289042 1.291683
H 2.849403 -0.434361 2.130374
C 4.490548 1.823490 0.182364
H 5.190910 1.437483 -1.820789
H 3.693893 1.890005 2.187313
H 4.868700 2.842170 0.208303
H -2.642393 -3.313020 -0.043159
H -1.357639 -2.972792 1.130094
H 1.345929 -1.778772 -1.303776
H 3.075898 -2.709278 1.054854
45
-1762.342071
C 0.282631 2.024147 -0.079802
N -1.049198 1.434913 0.223023
S -1.190363 -0.267601 -0.177912
O -0.656206 -0.313150 -1.545046
O -0.650496 -1.151525 0.859826
C -2.965378 -0.453258 -0.217273
C -3.694590 0.155427 -1.242491
C -3.588820 -1.241776 0.747573
C -5.072195 -0.026932 -1.285336
H -3.188553 0.757985 -1.989089
C -4.971440 -1.413966 0.684733
H -2.997749 -1.707651 1.528221
C -5.731844 -0.813598 -0.326304
H -5.465703 -2.025334 1.435229
C 1.479221 1.511081 0.726944
H 1.323280 1.746173 1.786115
H 1.525399 0.419888 0.660336
C 2.818476 2.117909 0.272997
H 3.598419 1.746500 0.948627
H 2.789949 3.208483 0.410651
C 3.236645 1.820087 -1.178901
C 3.313161 0.323493 -1.559701
H 3.621272 0.263859 -2.612622
H -5.647817 0.446234 -2.077039
C -7.225464 -1.019690 -0.402842
H -7.622159 -1.432317 0.529575
H -7.748137 -0.078977 -0.608790
H -7.484673 -1.716540 -1.210431
Cl -1.544890 1.739392 1.902343
H 4.217990 2.282904 -1.348441
C 4.270121 -0.492004 -0.711460
C 3.802063 -1.495642 0.147247
C 5.653262 -0.261154 -0.768074
C 4.685763 -2.242166 0.930344
H 2.734949 -1.700776 0.198412
C 6.539352 -1.003539 0.011807
H 6.040885 0.504659 -1.437245
C 6.057539 -1.997790 0.866857
H 4.298951 -3.016512 1.587938
H 7.607338 -0.809758 -0.051230
H 6.746912 -2.578636 1.474050
H 0.151647 3.104147 0.049153
H 0.409576 1.834951 -1.148755
H 2.551068 2.314798 -1.880479
H 2.313079 -0.122885 -1.514752

C_5-Cl_{gm}

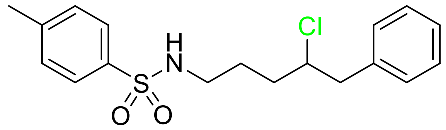

Name	E(B3LYP)	G(B3LYP)	E(RO-B2PLYP-D3)	G(RO-B2PLYP-D3)
Conformers				
Tosyl_NH_pentane_Ph_C5Cl_0002_cregen_B2PLYP.log	-1762.703567	-1762.308596	-1762.132458	-1761.81989468
Tosyl_NH_pentane_Ph_C5Cl_0016_cregen_B2PLYP.log	-1762.701085	-1762.305960	-1762.132865	-1761.81829447
Tosyl_NH_pentane_Ph_C5Cl_0003_cregen_B2PLYP.log	-1762.699834	-1762.304914	-1762.132481	-1761.81784045
Tosyl_NH_pentane_Ph_C5Cl_0015_cregen_B2PLYP.log	-1762.698598	-1762.303747	-1762.130587	-1761.81778380
Tosyl_NH_pentane_Ph_C5Cl_0014_cregen_B2PLYP.log	-1762.698161	-1762.303406	-1762.130353	-1761.81686076
Tosyl_NH_pentane_Ph_C5Cl_0017_cregen_B2PLYP.log	-1762.700654	-1762.305630	-1762.131469	-1761.81618731
Tosyl_NH_pentane_Ph_C5Cl_0018_cregen_B2PLYP.log	-1762.701270	-1762.306100	-1762.128370	-1761.81563541
Tosyl_NH_pentane_Ph_C5Cl_0001_cregen_B2PLYP.log	-1762.703567	-1762.308596	-1762.127400	-1761.81513898
Tosyl_NH_pentane_Ph_C5Cl_0006_cregen_B2PLYP.log	-1762.698598	-1762.303747	-1762.127917	-1761.81510629
Tosyl_NH_pentane_Ph_C5Cl_0009_cregen_B2PLYP.log	-1762.701085	-1762.305960	-1762.129366	-1761.81478675

45
-1762.390644
C 0.493283 1.403835 1.169806
N 0.469430 -0.066048 1.182780
S 1.122016 -0.953726 -0.080843
O 0.951442 -2.354076 0.310418
O 0.539176 -0.402169 -1.310007
C 2.885537 -0.614497 -0.139929
C 3.757561 -1.387804 0.630821
C 3.368180 0.424750 -0.937777
C 5.120636 -1.103751 0.606444
H 3.372092 -2.216272 1.216293
C 4.735743 0.695053 -0.948380
H 2.684249 0.991034 -1.560915
C 5.631179 -0.056360 -0.175060
H 5.114273 1.497260 -1.577032
C -0.740504 1.990482 1.868402
H -0.704725 1.745301 2.938897
H -0.667286 3.081929 1.797681
C -2.089379 1.514292 1.307544
H -2.898304 2.041678 1.828919
H -2.212106 0.446558 1.522601
C -2.280556 1.666813 -0.199885
C -3.647367 1.183456 -0.710312
H -3.699734 1.396116 -1.784825
H 5.802013 -1.712361 1.196034
C 7.107603 0.260229 -0.172016
H 7.415954 0.744180 -1.104110
H 7.361703 0.942431 0.650161
H 7.711176 -0.643951 -0.041248
H 0.666153 -0.508818 2.076496
H -1.483580 1.155795 -0.741650
C -3.891555 -0.294794 -0.460895
C -4.974974 -0.713889 0.321752
C -3.047202 -1.271264 -1.012065
C -5.216170 -2.070644 0.547419
H -5.639805 0.030521 0.754724
C -3.280221 -2.626726 -0.779727
H -2.191875 -0.975970 -1.614331
C -4.367260 -3.031715 -0.001800
H -6.065855 -2.373893 1.153970
H -2.607426 -3.365893 -1.205741
H -4.549043 -4.088411 0.175789
H 0.507194 1.715604 0.122827
H -4.435053 1.777315 -0.232526
Cl -2.084681 3.440837 -0.687347
H 1.409144 1.792740 1.637579
45
-1762.390451
C 0.490119 1.405176 1.172063
N 0.468005 -0.064761 1.184212
S 1.123027 -0.951035 -0.079111
O 0.953415 -2.351766 0.311200
O 0.541072 -0.399408 -1.308667
C 2.886393 -0.610935 -0.137001
C 3.368473 0.432119 -0.930218
C 3.757775 -1.381553 0.637167
C 4.735074 0.707334 -0.934668
H 2.684042 1.002458 -1.549097
C 5.119907 -1.092645 0.618926
H 3.371764 -2.207097 1.226403
C 5.630673 -0.046241 -0.163699
H 5.800050 -1.694293 1.217072
C -0.745109 1.990003 1.869644
H -0.710223 1.744327 2.940049
H -0.673054 3.081569 1.799577
C -2.092842 1.512612 1.307058
H -2.902904 2.038991 1.827683
H -2.214733 0.444679 1.521613
C -2.282422 1.665431 -0.200526
C -3.648086 1.180717 -0.712672
H -3.699362 1.393407 -1.787229
H 5.112401 1.517357 -1.553989
C 7.113482 0.236584 -0.201028
H 7.613079 -0.388222 -0.953133
H 7.316504 1.281055 -0.458068
H 7.586861 0.023888 0.763210
H 0.664320 -0.507736 2.077916
H -1.484247 1.155474 -0.741521
C -3.890989 -0.297801 -0.463605
C -4.974831 -0.718089 0.317803
C -3.044953 -1.273326 -1.013857
C -5.214825 -2.075113 0.543129
H -5.640947 0.025583 0.750064
C -3.276780 -2.629045 -0.781850
H -2.189259 -0.977060 -1.615123
C -4.364264 -3.035240 -0.005175
H -6.064866 -2.379304 1.148709
H -2.602704 -3.367471 -1.207122
H -4.545105 -4.092139 0.172166
H 0.504762 1.717551 0.125277
H -4.436977 1.773701 -0.235791
Cl -2.087974 3.439870 -0.687215
H 1.405010 1.794849 1.641065
45
-1762.390461
C 0.490014 1.405368 1.171839
N 0.467970 -0.064547 1.184021
S 1.123037 -0.950886 -0.079215
O 0.953372 -2.351587 0.311158
O 0.541179 -0.399326 -1.308838
C 2.886411 -0.610834 -0.137019
C 3.757768 -1.381774 0.636840
C 3.368523 0.432500 -0.929856
C 5.119920 -1.092928 0.618675
H 3.371707 -2.207495 1.225791
C 4.735128 0.707658 -0.934221
H 2.684130 1.003091 -1.548545
C 5.630714 -0.046254 -0.163554
H 5.112482 1.517903 -1.553235
C -0.745193 1.990142 1.869488
H -0.710182 1.744515 2.939900
H -0.673225 3.081714 1.799403
C -2.092941 1.512637 1.307038
H -2.903004 2.039015 1.827664
H -2.214783 0.444725 1.521701
C -2.282632 1.665381 -0.200540
C -3.648268 1.180561 -0.712643
H -3.699599 1.393268 -1.787195
H 5.800040 -1.694840 1.216579
C 7.113529 0.236529 -0.200814
H 7.613073 -0.387977 -0.953200
H 7.316582 1.281099 -0.457420
H 7.586933 0.023407 0.763316
H 0.664173 -0.507534 2.077740
H -1.484454 1.155515 -0.741611
C -3.890981 -0.297987 -0.463566
C -3.044895 -1.273405 -1.013942
C -4.974655 -0.718410 0.317993
C -3.276495 -2.629148 -0.781872
H -2.189341 -0.977040 -1.615349
C -5.214427 -2.075466 0.543375
H -5.640810 0.025176 0.750339
C -4.363809 -3.035479 -0.005029
H -2.602368 -3.367483 -1.207217
H -6.064344 -2.379767 1.149071
H -4.544478 -4.092399 0.172354
H 0.504586 1.717716 0.125043
H -4.437224 1.773458 -0.235754
Cl -2.088345 3.439858 -0.687209
H 1.404915 1.795112 1.640768
45
-1762.390453
C 0.490209 1.405224 1.172093
N 0.468069 -0.064711 1.184322
S 1.123029 -0.951094 -0.078940
O 0.953463 -2.351789 0.311502
O 0.541033 -0.399598 -1.308537
C 2.886386 -0.610981 -0.136957
C 3.368409 0.432037 -0.930253

C 3.757818 -1.381558 0.637194
C 4.735006 0.707266 -0.934790
H 2.683942 1.002338 -1.549128
C 5.119944 -1.092640 0.618863
H 3.371844 -2.207075 1.226492
C 5.630654 -0.046265 -0.163837
H 5.800126 -1.694255 1.216996
C -0.745019 1.990101 1.869622
H -0.710143 1.744497 2.940042
H -0.672956 3.081661 1.799480
C -2.092747 1.512660 1.307063
H -2.902806 2.039052 1.827678
H -2.214628 0.444734 1.521665
C -2.282351 1.665424 -0.200521
C -3.648047 1.180768 -0.712623
H -3.699369 1.393493 -1.787172
H 5.112292 1.517264 -1.554170
C 7.113459 0.236570 -0.201254
H 7.316466 1.280945 -0.458687
H 7.586823 0.024249 0.763075
H 7.613079 -0.388519 -0.953107
H 0.664329 -0.507653 2.078053
H -1.484200 1.155442 -0.741528
C -3.891013 -0.297752 -0.463602
C -4.974971 -0.718010 0.317658
C -3.044956 -1.273301 -1.013780
C -5.215058 -2.075028 0.542919
H -5.641107 0.025681 0.749856
C -3.276877 -2.629014 -0.781835
H -2.189179 -0.977069 -1.614944
C -4.364474 -3.035180 -0.005304
H -6.065187 -2.379191 1.148387
H -2.602783 -3.367458 -1.207048
H -4.545385 -4.092075 0.171985
H 0.504885 1.717548 0.125292
H -4.436898 1.773764 -0.235692
Cl -2.087847 3.439846 -0.687264
H 1.405093 1.794904 1.641099
45
-1762.386948
C 0.250628 0.578164 1.913334
N 0.143806 0.919449 0.482695
S -1.062059 2.023122 0.027475
O -1.335846 2.981537 1.107055
O -0.646974 2.484238 -1.302850
C -2.500512 0.967956 -0.130525
C -3.528462 1.062781 0.804927
C -2.585507 0.078433 -1.206118
C -4.650618 0.243709 0.665187
H -3.450087 1.775443 1.618849
C -3.709760 -0.729519 -1.328679
H -1.785715 0.032087 -1.937914
C -4.758306 -0.662959 -0.395115
H -3.779740 -1.422853 -2.163337
C 1.421951 -0.377533 2.174126
H 2.353711 0.078081 1.822798
H 1.519584 -0.452785 3.265091
C 1.263805 -1.817323 1.638816
H 0.288108 -2.193138 1.967463
H 2.015757 -2.457656 2.118119
C 1.359310 -2.026767 0.119120
C 2.769964 -1.971907 -0.501809
H 2.696778 -2.378665 -1.516753
H -5.455775 0.315444 1.391987
C -5.968994 -1.553065 -0.543001
H -6.714242 -1.349048 0.231501
H -6.449224 -1.411294 -1.518653
H -5.692180 -2.612237 -0.472108
H 1.020763 1.265062 0.087392
H 0.679568 -1.352218 -0.400975
C 3.389516 -0.587884 -0.571932
C 4.523310 -0.263182 0.183636
C 2.850234 0.395304 -1.417049
C 5.092397 1.011144 0.115523
H 4.965209 -1.016039 0.832639
C 3.408962 1.673134 -1.481139
H 1.983220 0.162122 -2.031016
C 4.532779 1.985647 -0.711163
H 5.972550 1.240436 0.710489
H 2.966511 2.420364 -2.134099
H 4.970455 2.978701 -0.761711
H -0.691881 0.102160 2.209804
H 3.424465 -2.648938 0.059493
Cl 0.686973 -3.713217 -0.242802
H 0.368296 1.480557 2.527646
45
-1762.386957
C 0.250597 0.578156 1.913309
N 0.143831 0.919463 0.482676
S -1.062017 2.023099 0.027391
O -1.335803 2.981595 1.106900
O -0.646940 2.484125 -1.302968
C -2.500478 0.967933 -0.130528
C -3.528464 1.062893 0.804872
C -2.585449 0.078279 -1.206015
C -4.650633 0.243830 0.665185
H -3.450094 1.775633 1.618726
C -3.709716 -0.729662 -1.328524
H -1.785616 0.031805 -1.937758
C -4.758308 -0.662950 -0.395025
H -3.779666 -1.423116 -2.163086
C 1.421904 -0.377549 2.174135
H 2.353678 0.078069 1.822846
H 1.519497 -0.452812 3.265103
C 1.263783 -1.817334 1.638802
H 0.288090 -2.193165 1.967441
H 2.015742 -2.457659 2.118101
C 1.359290 -2.026747 0.119105
C 2.769941 -1.971903 -0.501827
H 2.696743 -2.378630 -1.516782
H -5.455806 0.315655 1.391958
C -5.969085 -1.552932 -0.542932
H -6.450073 -1.410087 -1.518061
H -5.692215 -2.612175 -0.473416
H -6.713751 -1.349744 0.232347
H 1.020808 1.265043 0.087388
H 0.679554 -1.352187 -0.400982
C 3.389516 -0.587887 -0.571911
C 2.850263 0.395324 -1.417021
C 4.523298 -0.263213 0.183689
C 3.409007 1.673151 -1.481071
H 1.983260 0.162166 -2.031012
C 5.092399 1.011108 0.115615
H 4.965173 -1.016089 0.832685
C 4.532809 1.985635 -0.711063
H 2.966579 2.420397 -2.134028
H 5.972541 1.240379 0.710606
H 4.970498 2.978685 -0.761580
H -0.691928 0.102154 2.209734
H 3.424431 -2.648960 0.059457
Cl 0.686922 -3.713192 -0.242855
H 0.368247 1.480538 2.527644
45
-1762.389916
C 0.456340 2.094488 1.245135
N 1.408973 0.969431 1.332034
S 1.591058 -0.114897 0.052537
O 1.049256 -1.427140 0.438887
O 1.107806 0.574655 -1.151909
C 3.368469 -0.296720 -0.074528
C 3.973752 -1.467149 0.378095
C 4.125096 0.732585 -0.641959
C 5.358986 -1.599499 0.269788
H 3.365768 -2.261407 0.797389
C 5.503578 0.581931 -0.742413
H 3.637374 1.631105 -1.005534
C 6.143315 -0.583781 -0.288810
H 6.095918 1.379910 -1.183722
C -0.965960 1.797575 1.745253
H -0.903778 1.465472 2.791647

H -1.517137 2.746022 1.760919
C -1.765115 0.752158 0.953187
H -2.685065 0.516832 1.498466
H -1.195231 -0.184905 0.900229
C -2.128444 1.138847 -0.479983
C -2.713698 -0.014576 -1.316672
H -1.870408 -0.694112 -1.497781
H 5.835022 -2.511141 0.621956
C 7.638997 -0.739170 -0.425898
H 8.011516 -1.578554 0.168962
H 7.921668 -0.922228 -1.470634
H 8.165977 0.166111 -0.103879
H 1.367976 0.454473 2.209193
H -1.256950 1.518858 -1.013499
C -3.862335 -0.790335 -0.705057
C -5.187272 -0.348870 -0.833093
C -3.622527 -1.982034 -0.005868
C -6.239051 -1.070303 -0.267421
H -5.393174 0.566779 -1.379896
C -4.672422 -2.706439 0.560902
H -2.602755 -2.349282 0.087801
C -5.985395 -2.251013 0.433341
H -7.259126 -0.711583 -0.379071
H -4.463888 -3.629498 1.095909
H -6.805433 -2.815199 0.869978
H 0.457491 2.430571 0.206114
H -3.004586 0.389775 -2.292331
Cl -3.289390 2.580568 -0.459376
H 0.884324 2.908397 1.842532
45
-1762.386966
C 0.250527 0.578135 1.913269
N 0.143775 0.919377 0.482605
S -1.062059 2.023081 0.027333
O -1.335782 2.981587 1.106849
O -0.646985 2.484090 -1.303030
C -2.500557 0.967956 -0.130558
C -2.585631 0.078395 -1.206116
C -3.528425 1.062801 0.804976
C -3.709889 -0.729560 -1.328562
H -1.785884 0.032012 -1.937959
C -4.650595 0.243720 0.665350
H -3.449976 1.775467 1.618887
C -4.758376 -0.662957 -0.394926
H -5.455677 0.315457 1.392232
C 1.421742 -0.377672 2.174128
H 2.353581 0.077871 1.822917
H 1.519259 -0.452992 3.265098
C 1.263494 -1.817406 1.638708
H 0.287653 -2.193088 1.967083
H 2.015221 -2.457893 2.118157
C 1.359361 -2.026798 0.119020
C 2.770178 -1.971892 -0.501541
H 2.697301 -2.378796 -1.516451
H -3.779920 -1.422949 -2.163170
C -5.969183 -1.552892 -0.542850
H -6.713252 -1.350640 0.233247
H -6.450974 -1.408975 -1.517430
H -5.692161 -2.612182 -0.474773
H 1.020751 1.264997 0.087350
H 0.679755 -1.352211 -0.401203
C 3.389652 -0.587837 -0.571706
C 2.850387 0.395268 -1.416932
C 4.523390 -0.263037 0.183904
C 3.409080 1.673109 -1.481091
H 1.983418 0.162013 -2.030933
C 5.092443 1.011300 0.115719
H 4.965269 -1.015824 0.832999
C 4.532845 1.985718 -0.711078
H 2.966640 2.420275 -2.134132
H 5.972552 1.240666 0.710720
H 4.970491 2.978782 -0.761680
H -0.692035 0.102224 2.209720
H 3.424590 -2.648798 0.060018
Cl 0.687125 -3.713201 -0.243096
H 0.368251 1.480543 2.527543
45
-1762.386959
C 0.250834 0.578499 1.913382
N 0.143733 0.919499 0.482628
S -1.062371 2.023321 0.027711
O -1.336261 2.981364 1.107584
O -0.647338 2.484908 -1.302460
C -2.500641 0.967939 -0.130573
C -3.529039 1.063083 0.804357
C -2.585017 0.077917 -1.205785
C -4.651058 0.243867 0.664432
H -3.451091 1.776082 1.618024
C -3.709139 -0.730199 -1.328522
H -1.784874 0.031295 -1.937181
C -4.758142 -0.663306 -0.395509
H -3.778637 -1.423947 -2.162873
C 1.422266 -0.377087 2.174109
H 2.353941 0.078490 1.822505
H 1.520142 -0.452173 3.265061
C 1.263983 -1.816951 1.639054
H 0.288311 -2.192667 1.967904
H 2.015960 -2.457273 2.118335
C 1.359248 -2.026699 0.119357
C 2.769837 -1.971933 -0.501756
H 2.696490 -2.378771 -1.516659
H -5.456569 0.315867 1.390814
C -5.968653 -1.553637 -0.543462
H -6.715055 -1.348029 0.229514
H -6.447399 -1.413785 -1.520111
H -5.692026 -2.612684 -0.469995
H 1.020567 1.265512 0.087380
H 0.679483 -1.352149 -0.400717
C 3.389507 -0.587986 -0.572070
C 2.850203 0.395203 -1.417178
C 4.523440 -0.263349 0.183308
C 3.409054 1.672964 -1.481442
H 1.983074 0.162060 -2.030995
C 5.092654 1.010912 0.115019
H 4.965356 -1.016204 0.832299
C 4.533022 1.985412 -0.711654
H 2.966596 2.420195 -2.134395
H 5.972922 1.240148 0.709836
H 4.970787 2.978419 -0.762330
H -0.691582 0.102481 2.210106
H 3.424358 -2.648982 0.059509
Cl 0.686892 -3.713096 -0.242155
H 0.368522 1.481036 2.527454
45
-1762.388700
C 0.881969 2.731950 -0.271807
N 1.076574 1.359433 -0.758456
S 1.328569 0.055015 0.257354
O 1.110130 0.499383 1.640197
O 0.566691 -1.056906 -0.330083
C 3.068550 -0.347538 0.101334
C 3.493286 -1.164605 -0.948762
C 3.979549 0.164515 1.025076
C 4.848510 -1.456961 -1.074084
H 2.769556 -1.575160 -1.645049
C 5.332848 -0.139195 0.882822
H 3.629022 0.777041 1.848731
C 5.789157 -0.950824 -0.164099
H 6.045188 0.257062 1.602241
C -0.571834 3.230108 -0.266056
H -0.529954 4.284146 0.038666
H -0.968641 3.234758 -1.289112
C -1.540522 2.479262 0.669322
H -2.305338 3.175348 1.033539
H -0.990998 2.122307 1.549888
C -2.263953 1.268134 0.069509
C -3.078376 0.498658 1.117941
H -3.826890 1.172864 1.550762
H 5.182101 -2.094120 -1.889569
C 7.252110 -1.301821 -0.293450

H 7.464087 -2.277077 0.164307
H 7.557967 -1.363982 -1.343245
H 7.887723 -0.562294 0.203599
H 0.571478 1.081878 -1.593888
H -1.569681 0.585773 -0.421667
C -3.738212 -0.771360 0.621838
C -5.130253 -0.861495 0.510213
C -2.961791 -1.886742 0.274332
C -5.738232 -2.035552 0.061888
H -5.743256 -0.002882 0.773987
C -3.567025 -3.059582 -0.176891
H -1.877960 -1.836206 0.351812
C -4.957498 -3.138961 -0.283262
H -6.821224 -2.086102 -0.017608
H -2.950434 -3.914765 -0.441824
H -5.428165 -4.054779 -0.631549
H 1.489554 3.398506 -0.896992
H -2.369989 0.249873 1.920983
Cl -3.371000 1.833728 -1.296001
H 1.295452 2.770411 0.739446

C_6-Cl_{gm}

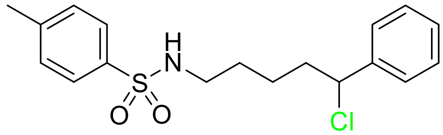

Name	E(B3LYP)	G(B3LYP)	E(RO-B2PLYP-D3)	G(RO-B2PLYP-D3)
Conformers				
Tosyl_NH_pentane_Ph_C5Cl_0002_cregen_B2PLYP.log	-1762.703567	-1762.308596	-1762.132458	-1761.81989468
Tosyl_NH_pentane_Ph_C5Cl_0016_cregen_B2PLYP.log	-1762.701085	-1762.305960	-1762.132865	-1761.81829447
Tosyl_NH_pentane_Ph_C5Cl_0003_cregen_B2PLYP.log	-1762.699834	-1762.304914	-1762.132481	-1761.81784045
Tosyl_NH_pentane_Ph_C5Cl_0015_cregen_B2PLYP.log	-1762.698598	-1762.303747	-1762.130587	-1761.81778380
Tosyl_NH_pentane_Ph_C5Cl_0014_cregen_B2PLYP.log	-1762.698161	-1762.303406	-1762.130353	-1761.81686076
Tosyl_NH_pentane_Ph_C5Cl_0017_cregen_B2PLYP.log	-1762.700654	-1762.305630	-1762.131469	-1761.81618731
Tosyl_NH_pentane_Ph_C5Cl_0018_cregen_B2PLYP.log	-1762.701270	-1762.306100	-1762.128370	-1761.81563541
Tosyl_NH_pentane_Ph_C5Cl_0001_cregen_B2PLYP.log	-1762.703567	-1762.308596	-1762.127400	-1761.81513898
Tosyl_NH_pentane_Ph_C5Cl_0006_cregen_B2PLYP.log	-1762.698598	-1762.303747	-1762.127917	-1761.81510629
Tosyl_NH_pentane_Ph_C5Cl_0009_cregen_B2PLYP.log	-1762.701085	-1762.305960	-1762.129366	-1761.81478675

45
-1762.385209
C -1.059847 2.051160 -0.797112
N -1.034039 1.349277 0.499544
S -1.295193 -0.314515 0.552530
O -0.608792 -1.020006 -0.547850
O -1.024441 -0.697512 1.940466
C -3.053670 -0.416694 0.220322
C -3.503721 -1.111588 -0.899982
C -3.955904 0.151532 1.125729
C -4.877634 -1.226965 -1.120881
H -2.784653 -1.554573 -1.580442
C -5.319871 0.027035 0.889077
H -3.588665 0.681840 1.998186
C -5.803281 -0.663320 -0.235884
H -6.025095 0.468888 1.589076
C 0.183487 1.916525 -1.691199
H 0.363407 0.851839 -1.870287
H -0.073856 2.348289 -2.667162
C 1.455481 2.611271 -1.138505
H 1.267925 3.023292 -0.139240
H 1.701837 3.474385 -1.768142
C 2.696837 1.707690 -1.068514
C 2.613336 0.566083 -0.049875
H 1.677821 0.017834 -0.165835
H -5.232808 -1.767009 -1.994897
C -7.289183 -0.795223 -0.470190
H -7.503031 -1.301075 -1.416384
H -7.766294 -1.371040 0.332580
H -7.777048 0.186627 -0.494378
H -0.242824 1.568280 1.107320
H 3.586277 2.312687 -0.857696
C 3.762587 -0.407344 -0.105441
C 5.095294 0.007109 0.028180
C 3.491606 -1.762532 -0.331648
C 6.134604 -0.915448 -0.070879
H 5.318720 1.051399 0.228996
C 4.533279 -2.686305 -0.435318
H 2.459292 -2.091377 -0.424602
C 5.856567 -2.264896 -0.306140
H 7.163393 -0.582726 0.038924
H 4.308039 -3.734760 -0.611029
H 6.668642 -2.983108 -0.382333
H -1.246084 3.106872 -0.564010
H -1.946329 1.693294 -1.331907
H 2.863992 1.238320 -2.048470
Cl 2.474987 1.291807 1.664298
45
-1762.386375
C 0.728192 2.955524 0.305396
N 0.960249 1.504815 0.411943
S 1.446951 0.618741 -0.928694
O 0.437748 -0.422472 -1.185364
O 1.834782 1.581447 -1.966368
C 2.929362 -0.226358 -0.376979
C 4.170818 0.388833 -0.544082
C 2.827522 -1.489693 0.206947
C 5.317929 -0.269151 -0.106213
H 4.234087 1.357407 -1.028535
C 3.985822 -2.131328 0.640834
H 1.857966 -1.967204 0.300595
C 5.245337 -1.532435 0.498087
H 3.910317 -3.118405 1.090505
C -0.594059 3.410389 -0.336454
H -0.591597 4.508175 -0.299991
H -0.595840 3.137397 -1.398975
C -1.862350 2.874601 0.361630
H -2.668154 3.614176 0.276265
H -1.663060 2.773424 1.436301
C -2.361539 1.530852 -0.209779
C -3.154221 0.666530 0.770663
H -3.913982 1.259404 1.285341
H 6.287613 0.203290 -0.243262
C 6.491466 -2.224029 0.997541
H 7.373038 -1.929832 0.418813
H 6.689644 -1.967665 2.046828
H 6.395289 -3.313299 0.942153
H 0.270332 0.989357 0.956876
H -1.533689 0.938932 -0.607815
C -3.781982 -0.559981 0.162292
C -5.144168 -0.809898 0.367976
C -3.037517 -1.447628 -0.630134
C -5.760916 -1.919257 -0.212566
H -5.726626 -0.130730 0.986708
C -3.654106 -2.556523 -1.206361
H -1.976479 -1.277773 -0.790731
C -5.015915 -2.795308 -1.001703
H -6.819749 -2.097993 -0.045575
H -3.068351 -3.237077 -1.818360
H -5.491895 -3.661183 -1.454268

H 1.570987 3.376205 -0.247799
H 0.788082 3.338178 1.331880
H -3.025130 1.722770 -1.064268
Cl -2.019650 0.152131 2.176124
45
-1762.386776
C -1.906033 -2.159799 -1.243619
N -2.546954 -1.710543 0.004150
S -3.600030 -0.386124 -0.087297
O -4.241622 -0.305708 -1.406965
O -4.411377 -0.479683 1.131120
C -2.464784 0.995865 0.035248
C -1.572756 1.075533 1.108609
C -2.533161 2.011414 -0.917773
C -0.738445 2.184761 1.214515
H -1.521331 0.275254 1.838822
C -1.693551 3.118504 -0.792156
H -3.231114 1.924982 -1.743456
C -0.789433 3.225536 0.272112
H -1.742419 3.910653 -1.535206
C -0.911016 -3.291531 -0.957790
H -1.467020 -4.166452 -0.591731
H -0.473840 -3.594058 -1.919336
C 0.205157 -2.959739 0.046670
H 0.807096 -3.866223 0.200604
H -0.235661 -2.708100 1.016099
C 1.124420 -1.818231 -0.404435
C 2.350550 -1.595878 0.487057
H 2.862819 -2.546452 0.654570
H -0.036179 2.242829 2.042224
C 0.094964 4.440602 0.421551
H 0.221499 4.965143 -0.530792
H -0.338304 5.154733 1.134150
H 1.087124 4.171064 0.799592
H -3.048474 -2.450103 0.496122
H 0.564817 -0.879583 -0.479663
C 3.329871 -0.584306 -0.056656
C 2.940097 0.730216 -0.352196
C 4.651447 -0.969932 -0.308945
C 3.853256 1.632219 -0.893959
H 1.923432 1.051650 -0.142951
C 5.566773 -0.067530 -0.854345
H 4.967612 -1.984373 -0.076564
C 5.169219 1.236092 -1.148923
H 3.538681 2.648280 -1.117268
H 6.588833 -0.383943 -1.044207
H 5.879465 1.941823 -1.571130
H -1.389429 -1.294641 -1.672100
H -2.646539 -2.488195 -1.984703
H 1.514634 -2.033827 -1.410406
Cl 1.809882 -1.098725 2.193061
45
-1762.387925
C 0.293111 2.550941 -0.843098
N -0.545678 1.369469 -0.551305
S -1.656170 1.502024 0.724017
O -1.094586 0.944227 1.964134
O -2.148954 2.883592 0.682205
C -2.956690 0.394351 0.182567
C -3.765657 0.758524 -0.898126
C -3.168497 -0.799985 0.867140
C -4.787295 -0.095851 -1.296121
H -3.595463 1.698747 -1.412218
C -4.199372 -1.645627 0.452865
H -2.538132 -1.053668 1.712547
C -5.021293 -1.310399 -0.628813
H -4.368234 -2.578908 0.984216
C 1.325209 2.942776 0.228970
H 1.894338 3.800165 -0.158068
H 0.788176 3.300660 1.114955
C 2.288715 1.823089 0.654636
H 1.707268 0.981473 1.049801
H 2.892671 2.184061 1.497511
C 3.230646 1.347777 -0.465585
C 4.028195 0.102117 -0.063460
H 4.618215 0.310715 0.831929
H -5.418591 0.182724 -2.136638
C -6.146561 -2.217808 -1.065574
H -6.107097 -3.182515 -0.550892
H -7.123544 -1.766099 -0.851452
H -6.110237 -2.407649 -2.144687
H -0.005583 0.512915 -0.428934
H 3.932172 2.149377 -0.723703
C 3.184733 -1.130569 0.161677
C 2.355246 -1.642458 -0.848545
C 3.191512 -1.760834 1.410851
C 1.547016 -2.752003 -0.607256
H 2.365667 -1.187893 -1.835708
C 2.380557 -2.871491 1.655072
H 3.833339 -1.377953 2.200693
C 1.555110 -3.368436 0.647629
H 0.915728 -3.141734 -1.401378
H 2.396930 -3.345915 2.632365
H 0.925033 -4.233754 0.834181
H -0.388669 3.386303 -1.017640
H 0.780570 2.330119 -1.799243
H 2.670358 1.119484 -1.379939
Cl 5.305515 -0.227445 -1.363678
45
-1762.386776
C -1.906082 -2.159853 -1.243619
N -2.546979 -1.710512 0.004131
S -3.599994 -0.386068 -0.087332
O -4.241533 -0.305603 -1.407022
O -4.411383 -0.479604 1.131060
C -2.464717 0.995893 0.035264
C -2.533149 2.011531 -0.917659
C -1.572641 1.075473 1.108592
C -1.693541 3.118613 -0.791987
H -3.231141 1.925166 -1.743318
C -0.738326 2.184695 1.214553
H -1.521176 0.275130 1.838733
C -0.789364 3.225551 0.272243
H -0.036030 2.242704 2.042239
C -0.911085 -3.291590 -0.957745
H -1.467103 -4.166491 -0.591659
H -0.473915 -3.594164 -1.919279
C 0.205100 -2.959785 0.046696
H 0.807060 -3.866259 0.200612
H -0.235698 -2.708173 1.016141
C 1.124314 -1.818239 -0.404415
C 2.350544 -1.595951 0.486949
H 2.862851 -2.546531 0.654312
H -1.742461 3.910832 -1.534959
C 0.095067 4.440592 0.421700
H 0.221917 4.964929 -0.530718
H -0.338347 5.154907 1.134022
H 1.087104 4.171051 0.800063
H -3.048487 -2.450023 0.496185
H 0.564688 -0.879593 -0.479504
C 3.329794 -0.584308 -0.056757
C 4.651389 -0.969857 -0.309069
C 2.939951 0.730206 -0.352246
C 5.566667 -0.067391 -0.854443
H 4.967608 -1.984290 -0.076722
C 3.853062 1.632272 -0.893985
H 1.923275 1.051591 -0.142978
C 5.169044 1.236221 -1.148974
H 6.588742 -0.383745 -1.044321
H 3.538435 2.648325 -1.117256
H 5.879249 1.942004 -1.571163
H -1.389476 -1.294727 -1.672164
H -2.646608 -2.488278 -1.984669
H 1.514412 -2.033750 -1.410450
Cl 1.810061 -1.098984 2.193080
45
-1762.383223
C -1.122264 -2.448573 -1.077210
N -1.221219 -2.041390 0.338627
S -2.676095 -1.291033 0.823580

O -3.788512 -1.736170 -0.025839
O -2.711371 -1.469999 2.279009
C -2.366096 0.430507 0.449195
C -1.700450 1.224847 1.385733
C -2.790279 0.959675 -0.770415
C -1.436763 2.556581 1.077847
H -1.414913 0.804701 2.344547
C -2.513460 2.294634 -1.061920
H -3.343069 0.338347 -1.466948
C -1.825067 3.108696 -0.152618
H -2.839941 2.710714 -2.011535
C 0.122209 -3.309889 -1.317181
H 0.023644 -4.238787 -0.736566
H 0.099353 -3.613333 -2.372525
C 1.486850 -2.670920 -1.003835
H 2.254988 -3.424303 -1.214690
H 1.564051 -2.461541 0.069050
C 1.805724 -1.390522 -1.815740
C 1.598067 -0.100121 -1.008089
H 0.580821 -0.054753 -0.618856
H -0.921413 3.179660 1.804369
C -1.487124 4.538712 -0.496500
H -0.496006 4.597733 -0.964866
H -2.208630 4.963987 -1.201384
H -1.465452 5.172543 0.395995
H -1.085429 -2.821473 0.983604
H 1.179524 -1.347997 -2.714521
C 2.580468 0.085031 0.125440
C 2.119462 0.067255 1.447247
C 3.955333 0.231556 -0.109515
C 3.012486 0.185646 2.515095
H 1.056104 -0.048120 1.638535
C 4.845807 0.356473 0.954248
H 4.324895 0.269833 -1.130617
C 4.377320 0.331749 2.271245
H 2.638189 0.166997 3.535227
H 5.907765 0.476073 0.756675
H 5.073697 0.429036 3.099804
H -1.076450 -1.533916 -1.679984
H -2.015583 -3.000542 -1.395564
H 2.843419 -1.412612 -2.165188
Cl 1.688953 1.337373 -2.176809
45
-1762.387930
C 0.292843 2.551109 -0.842921
N -0.545947 1.369621 -0.551162
S -1.656556 1.502238 0.724027
O -1.095028 0.944754 1.964309
O -2.149531 2.883724 0.681933
C -2.956845 0.394285 0.182612
C -3.766075 0.758442 -0.897899
C -3.168190 -0.800253 0.866958
C -4.787501 -0.096156 -1.295933
H -3.596240 1.698841 -1.411786
C -4.198864 -1.646132 0.452640
H -2.537663 -1.053905 1.712256
C -5.021022 -1.310932 -0.628854
H -4.367371 -2.579570 0.983827
C 1.324986 2.942822 0.229152
H 1.894078 3.800281 -0.157782
H 0.787985 3.300546 1.115222
C 2.288559 1.823107 0.654619
H 1.707180 0.981452 1.049790
H 2.892623 2.184036 1.497436
C 3.230363 1.347896 -0.465755
C 4.028159 0.102381 -0.063688
H 4.618297 0.311130 0.831588
H -5.419015 0.182400 -2.136292
C -6.146025 -2.218613 -1.065733
H -6.109114 -2.409006 -2.144733
H -6.106785 -3.183058 -0.550547
H -7.123137 -1.766831 -0.852372
H -0.005845 0.513078 -0.428730
H 3.931720 2.149596 -0.724025
C 3.184943 -1.130431 0.161671
C 2.355192 -1.642388 -0.848300
C 3.192262 -1.760787 1.410794
C 1.547247 -2.752097 -0.606819
H 2.365173 -1.187742 -1.835430
C 2.381594 -2.871611 1.655210
H 3.834281 -1.377842 2.200449
C 1.555892 -3.368630 0.648013
H 0.915741 -3.141869 -1.400747
H 2.398390 -3.346104 2.632462
H 0.926051 -4.234088 0.834710
H -0.388951 3.386480 -1.017344
H 0.780259 2.330374 -1.799105
H 2.669959 1.119481 -1.380003
Cl 5.305310 -0.227101 -1.364099
45
-1762.386381
C 0.726538 2.960825 0.306153
N 0.962121 1.510483 0.411111
S 1.448560 0.627044 -0.931448
O 0.436805 -0.409832 -1.195821
O 1.842502 1.592544 -1.964191
C 2.926173 -0.225186 -0.377916
C 2.819788 -1.496102 0.187138
C 4.169214 0.393136 -0.525021
C 3.974979 -2.143396 0.622746
H 1.849651 -1.975103 0.265615
C 5.312238 -0.269998 -0.086097
H 4.236310 1.368489 -0.995179
C 5.234843 -1.542278 0.499892
H 6.282887 0.204885 -0.207571
C -0.596922 3.413692 -0.334529
H -0.596299 4.511459 -0.297380
H -0.599033 3.141458 -1.397258
C -1.863801 2.875285 0.364094
H -2.670766 3.613769 0.280253
H -1.663350 2.773287 1.438475
C -2.361428 1.531384 -0.208298
C -3.150961 0.664420 0.772296
H -3.910692 1.255341 1.289255
H 3.895964 -3.136482 1.058173
C 6.478688 -2.238260 0.998938
H 6.735660 -1.905122 2.013271
H 6.343504 -3.323822 1.036321
H 7.341800 -2.024168 0.359672
H 0.273865 0.993182 0.956453
H -1.533071 0.941614 -0.608469
C -3.777923 -0.562274 0.163472
C -5.138948 -0.815502 0.372759
C -3.033930 -1.446909 -0.632739
C -5.755020 -1.925188 -0.207863
H -5.721026 -0.138683 0.994412
C -3.649826 -2.556133 -1.209071
H -1.973770 -1.274411 -0.796256
C -5.010487 -2.798245 -1.000750
H -6.812962 -2.106501 -0.038034
H -3.064449 -3.234330 -1.824040
H -5.485933 -3.664363 -1.453407
H 1.568215 3.383932 -0.246867
H 0.785966 3.342304 1.333101
H -3.026752 1.723194 -1.061467
Cl -2.013016 0.149804 2.175012
45
-1762.386304
C 0.724213 2.961432 0.303699
N 0.959560 1.511282 0.411392
S 1.448426 0.625423 -0.928524
O 0.438449 -0.413783 -1.190484
O 1.841335 1.588814 -1.963625
C 2.927321 -0.223558 -0.373553
C 4.169509 0.395159 -0.524095
C 2.821514 -1.488877 0.204587
C 5.312996 -0.262864 -0.078033
H 4.235151 1.370006 -0.995502
C 3.976756 -2.130854 0.647081
H 1.850767 -1.964699 0.294336

C 5.237010 -1.532247 0.513739
H 3.897516 -3.116460 1.099263
C -0.598665 3.413042 -0.339131
H -0.598489 4.510856 -0.303490
H -0.599469 3.139262 -1.401456
C -1.866187 2.875190 0.358806
H -2.673367 3.613184 0.272750
H -1.667014 2.775069 1.433599
C -2.362607 1.530148 -0.211965
C -3.153131 0.664498 0.769023
H -3.913923 1.255940 1.283820
H 6.282458 0.215476 -0.195267
C 6.486967 -2.249571 0.965096
H 7.247313 -1.545737 1.319557
H 6.273939 -2.956748 1.773150
H 6.932410 -2.820911 0.139853
H 0.270876 0.994811 0.956948
H -1.533551 0.939981 -0.610083
C -3.778576 -0.563563 0.161366
C -5.139918 -0.816795 0.368583
C -3.032822 -1.449486 -0.631770
C -5.754586 -1.927755 -0.211097
H -5.723357 -0.138965 0.987857
C -3.647329 -2.559976 -1.207153
H -1.972399 -1.277014 -0.793610
C -5.008311 -2.802092 -1.000933
H -6.812790 -2.109061 -0.042899
H -3.060599 -3.239169 -1.819732
H -5.482653 -3.669209 -1.452838
H 1.566436 3.383523 -0.249269
H 0.782634 3.344799 1.329997
H -3.026947 1.720302 -1.066269
Cl -2.017050 0.152811 2.174311
45
-1762.387921
C 0.292894 2.551090 -0.842955
N -0.545874 1.369614 -0.551177
S -1.656436 1.502154 0.724077
O -1.094881 0.944517 1.964281
O -2.149357 2.883669 0.682139
C -2.956791 0.394301 0.182610
C -3.765977 0.758507 -0.897912
C -3.168218 -0.800232 0.866950
C -4.787445 -0.096035 -1.295970
H -3.596070 1.698886 -1.411815
C -4.198932 -1.646047 0.452611
H -2.537700 -1.053939 1.712237
C -5.021064 -1.310788 -0.628891
H -4.367493 -2.579485 0.983782
C 1.325062 2.942848 0.229093
H 1.894181 3.800255 -0.157917
H 0.788082 3.300664 1.115136
C 2.288585 1.823112 0.654601
H 1.707171 0.981478 1.049767
H 2.892642 2.184022 1.497432
C 3.230401 1.347871 -0.465748
C 4.028143 0.102325 -0.063668
H 4.618237 0.311047 0.831643
H -5.418912 0.182553 -2.136354
C -6.146186 -2.218356 -1.065700
H -6.110233 -2.407592 -2.144930
H -6.106185 -3.183328 -0.551556
H -7.123240 -1.767102 -0.850932
H -0.005801 0.513040 -0.428847
H 3.931793 2.149546 -0.724000
C 3.184855 -1.130451 0.161652
C 3.192127 -1.760837 1.410762
C 2.355100 -1.642354 -0.848342
C 2.381404 -2.871626 1.655144
H 3.834156 -1.377938 2.200432
C 1.547097 -2.752031 -0.606894
H 2.365124 -1.187705 -1.835471
C 1.555690 -3.368587 0.647925
H 2.398164 -3.346143 2.632386
H 0.915593 -3.141762 -1.400845
H 0.925797 -4.234013 0.834602
H -0.388894 3.386466 -1.017398
H 0.780309 2.330349 -1.799140
H 2.670015 1.119481 -1.380015
Cl 5.305340 -0.227193 -1.364008

TS-1,5'-XAT$_{bi}$

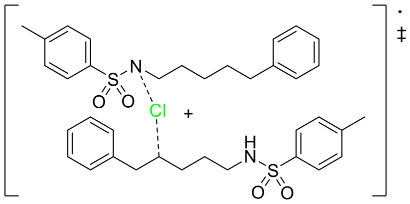

Name	E(B3LYP)	G(B3LYP)	E(RO-B2PLYP-D3)	G(RO-B2PLYP-D3)	NImag
Tosyl_NH_pentane_Ph_C5_H_Tosyl_N_pentane_TS_md_00150_B2PLYP	-3065.094652	-3064.311339	-3064.053151	-3063.41096055	-278.8939
Tosyl_NH_pentane_Ph_C5_H_Tosyl_N_pentane_TS_md_00100_B2PLYP	-3065.095112	-3064.311749	-3064.050914	-3063.41080092	-226.7895
Tosyl_NH_pentane_Ph_C5_H_Tosyl_N_pentane_TS_md_00200_B2PLYP	-3065.096812	-3064.313634	-3064.050451	-3063.40892257	-276.9114
Tosyl_NH_pentane_Ph_C5_H_Tosyl_N_pentane_TS_md_00250_B2PLYP	-3065.096812	-3064.313634	-3064.050453	-3063.40889221	-276.9058
Tosyl_NH_pentane_Ph_C5_H_Tosyl_N_pentane_TS_md_00030_B2PLYP	-3065.097080	-3064.313679	-3064.048323	-3063.40886236	-268.4221
Tosyl_NH_pentane_Ph_C5_H_Tosyl_N_pentane_TS_md_00080_B2PLYP	-3065.097108	-3064.313672	-3064.050665	-3063.40864119	-257.6465
Tosyl_NH_pentane_Ph_C5_H_Tosyl_N_pentane_TS_md_00012_B2PLYP	-3065.097108	-3064.313671	-3064.050666	-3063.40863897	-257.7207
Tosyl_NH_pentane_Ph_C5_H_Tosyl_N_pentane_TS_md_00450_B2PLYP	-3065.095102	-3064.311560	-3064.051772	-3063.40845794	-265.1690

89
-3063.41096055
C -1.056334 -2.164633 2.054278
C -2.368144 -2.971720 2.032548
C -3.597830 -2.214745 1.508637
C -4.876100 -3.077224 1.534855
C -0.313297 -2.052674 0.717023
N -1.096638 -1.317304 -0.297295
H -2.219807 -3.885043 1.437187
H -2.577454 -3.311562 3.057281
H -3.414448 -1.866304 0.487793
H -3.759712 -1.313646 2.116700
H 0.672044 -1.595033 0.871297
H -0.348885 -2.646612 2.742507
H -1.234851 -1.161054 2.459788
H -4.703214 -3.984103 0.939354
S -0.669233 -1.557505 -1.942918
O -1.586798 -0.734681 -2.730453
O -0.624062 -3.017056 -2.095106
C 0.990876 -0.929727 -2.229122
C 1.157914 0.363286 -2.726281
C 2.097278 -1.741654 -1.966685
C 2.447957 0.847320 -2.949016
H 0.285870 0.963845 -2.959472
C 3.377646 -1.243248 -2.194381
H 1.952326 -2.756767 -1.613469
C 3.576374 0.057152 -2.684621
H 2.579446 1.847090 -3.356322
H 4.239212 -1.875002 -1.994630
C 4.972058 0.582214 -2.925391
H 4.953251 1.546104 -3.443100
H 5.512420 0.718377 -1.980399
H 5.558132 -0.116145 -3.533920
H -0.169631 -3.056020 0.298909
C -6.092693 -2.349145 1.003176
C -6.351248 -2.297539 -0.374177
C -6.971755 -1.682026 1.866509
C -7.453405 -1.602166 -0.873601
H -5.682127 -2.811216 -1.061259
C -8.074992 -0.983129 1.373326
H -6.790551 -1.714120 2.939072
C -8.320565 -0.940855 -0.000109
H -7.639758 -1.584139 -1.944671
H -8.745894 -0.476920 2.063123
H -9.183751 -0.404979 -0.386613
H -5.067647 -3.412591 2.563436
Cl -1.087788 0.565760 0.109705
C 1.281313 2.900280 1.546445
C 0.550266 3.195972 0.227952
C -0.935661 2.991464 0.290502
C -1.798002 3.437871 -0.861077
C 2.812563 2.977238 1.434412
N 3.473432 1.799791 0.834155
H 0.960399 2.589937 -0.593730
H 0.747038 4.244790 -0.062213
H -1.399906 3.003253 1.276251
H 3.109394 3.842884 0.828266
H 0.953920 3.628714 2.300450
H 0.994966 1.910836 1.920593
H -1.582991 4.508667 -1.033119
S 3.764980 0.470028 1.843639
O 2.780323 -0.597219 1.606832
O 3.953492 1.013426 3.193216
C 5.338721 -0.125940 1.223159
C 5.483321 -1.485902 0.954112
C 6.422366 0.748384 1.096602
C 6.726229 -1.970554 0.543292
H 4.630618 -2.146955 1.062915
C 7.652930 0.248554 0.682100
H 6.297149 1.805107 1.307926
C 7.827152 -1.117224 0.402487
H 6.840572 -3.030854 0.331968
H 8.495355 0.927853 0.575738
C 9.176624 -1.647899 -0.018353
H 9.097211 -2.649569 -0.451485
H 9.651813 -0.993620 -0.757756
H 9.857754 -1.712365 0.840062
H 3.258553 3.105843 2.422203
C -3.287484 3.257070 -0.642090
C -3.957880 2.126348 -1.126161

C -4.012170 4.213595 0.081225
C -5.319657 1.949355 -0.875795
H -3.409162 1.372843 -1.684722
C -5.374819 4.041219 0.328087
H -3.505981 5.103082 0.452786
C -6.030763 2.904399 -0.147219
H -5.822452 1.057183 -1.237979
H -5.921947 4.794078 0.890014
H -7.089516 2.759802 0.050313
H 3.045708 1.474732 -0.033170
H -1.476952 2.925838 -1.778223
89
-3063.41080092
C 2.000099 -1.080322 -2.724103
C 3.451186 -1.546431 -2.946141
C 4.478627 -0.991836 -1.948829
C 5.903495 -1.509219 -2.231171
C 1.208601 -1.851366 -1.660288
N 1.755190 -1.680136 -0.299975
H 3.482923 -2.646051 -2.932894
H 3.754330 -1.249134 -3.960273
H 4.187547 -1.258873 -0.927978
H 4.477443 0.105943 -1.992965
H 0.147552 -1.563721 -1.698714
H 1.436448 -1.208585 -3.658455
H 1.975068 -0.007272 -2.498875
H 5.901786 -2.605649 -2.155126
S 1.300876 -2.867495 0.860936
O 1.955032 -2.493730 2.111849
O 1.586064 -4.142680 0.191561
C -0.474255 -2.783648 1.132866
C -0.963144 -2.100129 2.249609
C -1.346020 -3.426104 0.252069
C -2.336683 -2.061910 2.475858
H -0.266279 -1.634816 2.937839
C -2.720296 -3.365397 0.486880
H -0.954017 -3.992759 -0.585679
C -3.238699 -2.686498 1.598204
H -2.717275 -1.553686 3.359007
H -3.399045 -3.873183 -0.193743
C -4.726009 -2.630444 1.858928
H -4.959736 -2.943366 2.883198
H -5.111658 -1.610454 1.736181
H -5.277137 -3.281508 1.173740
H 1.278158 -2.924731 -1.869324
C 6.944603 -0.937813 -1.291669
C 7.027916 -1.377949 0.038008
C 7.832461 0.059729 -1.714930
C 7.967053 -0.836926 0.916438
H 6.350259 -2.154353 0.386632
C 8.776283 0.603987 -0.840756
H 7.785898 0.411391 -2.743577
C 8.846393 0.157649 0.479905
H 8.015747 -1.195526 1.941523
H 9.459147 1.373006 -1.193642
H 9.581575 0.576582 1.162011
H 6.178332 -1.271488 -3.267736
Cl 1.350850 0.086272 0.326463
C -1.716658 1.706764 0.686359
C -0.632416 2.185981 1.660860
C 0.723372 2.377261 1.050558
C 1.879347 2.752043 1.938739
C -3.074513 1.532924 1.376335
N -4.114158 0.884878 0.550594
H -0.555079 1.495548 2.513843
H -0.941836 3.154390 2.098994
H 0.757948 2.734575 0.022157
H -2.966185 0.900069 2.264911
H -1.814349 2.415894 -0.144745
H -1.407566 0.749122 0.252404
H 1.561417 3.629314 2.532910
S -4.793471 1.819650 -0.700486
O -4.102499 1.564481 -1.972993
O -4.901549 3.182125 -0.167189
C -6.430176 1.101963 -0.826870
C -6.775982 0.385927 -1.970646
C -7.352837 1.301392 0.204615
C -8.061924 -0.147607 -2.074088
H -6.048450 0.258191 -2.764917
C -8.628628 0.762445 0.084081
H -7.072056 1.873170 1.083013
C -9.004739 0.030752 -1.055340
H -8.335668 -0.707500 -2.964792
H -9.349398 0.913787 0.884141
C -10.402239 -0.527615 -1.179054
H -10.477426 -1.242733 -2.003731
H -10.712808 -1.035694 -0.258972
H -11.130205 0.272186 -1.366487
H -3.479314 2.493625 1.704828
C 3.165480 3.086126 1.210214
C 4.227754 2.175826 1.165084
C 3.307536 4.318672 0.558242
C 5.405006 2.484946 0.480687
H 4.130219 1.214211 1.661866
C 4.480888 4.631710 -0.128627
H 2.494947 5.042265 0.593491
C 5.532718 3.714155 -0.168519
H 6.219185 1.766751 0.455911
H 4.574976 5.593450 -0.626613
H 6.449166 3.955519 -0.700465
H -3.781398 0.007682 0.148345
H 2.048684 1.954319 2.674491
89
-3063.40930538
C 3.099595 -1.286279 -2.636640
C 4.599900 -1.494014 -2.357710
C 5.152829 -0.723219 -1.150575
C 6.656212 -0.982978 -0.927959
C 2.143354 -2.148051 -1.803664
N 2.185512 -1.834277 -0.356070
H 4.798565 -2.568539 -2.231168
H 5.159310 -1.187460 -3.252907
H 4.598854 -0.998572 -0.247274
H 4.989297 0.352874 -1.299345
H 1.117385 -2.058778 -2.188165
H 2.888878 -1.549441 -3.682283
H 2.834586 -0.226967 -2.532222
H 6.806056 -2.057430 -0.751987
S 1.647891 -3.091477 0.722868
O 1.784216 -2.551102 2.070974
O 2.406512 -4.259284 0.267885
C -0.090626 -3.403438 0.420214
C -1.049015 -2.821138 1.253532
C -0.470293 -4.250387 -0.624535
C -2.397686 -3.093045 1.027736
H -0.732117 -2.196389 2.081396
C -1.823941 -4.496953 -0.843217
H 0.284417 -4.734023 -1.235302
C -2.810293 -3.924075 -0.027496
H -3.144765 -2.671465 1.697121
H -2.120057 -5.159204 -1.652430
C -4.275156 -4.185115 -0.278217
H -4.820131 -4.321478 0.662611
H -4.736392 -3.340326 -0.805568
H -4.421028 -5.081393 -0.888806
H 2.451766 -3.195233 -1.873005
C 7.232183 -0.193617 0.229035
C 8.040691 0.929517 0.008339
C 6.948245 -0.555088 1.554376
C 8.549159 1.673373 1.076203
H 8.281220 1.219330 -1.012734
C 7.452970 0.184119 2.624147
H 6.326398 -1.426402 1.748602
C 8.255542 1.302971 2.389053
H 9.178662 2.538039 0.880890
H 7.221964 -0.116186 3.643070
H 8.651455 1.877892 3.222049
H 7.207462 -0.739851 -1.846374
Cl 1.402404 -0.268079 -0.018361
C -2.005744 1.334469 0.219839

C -1.128162 2.374663 0.931139
C 0.189526 2.613187 0.263709
C 1.283749 3.343118 0.988486
C -3.345666 1.116700 0.929841
N -4.153755 0.010011 0.385044
H -0.962520 2.071505 1.977494
H -1.685933 3.330186 0.997586
H 0.246328 2.530361 -0.819594
H -3.181847 0.888444 1.990806
H -2.194476 1.639396 -0.815405
H -1.465306 0.379932 0.178792
H 0.861624 4.265462 1.431254
S -5.126402 0.285201 -0.966249
O -5.373331 -1.058454 -1.509765
O -4.589055 1.347860 -1.827440
C -6.630610 0.922092 -0.228199
C -7.335751 0.144042 0.691607
C -7.110523 2.169526 -0.629504
C -8.528218 0.632332 1.219463
H -6.953271 -0.825714 0.992213
C -8.305900 2.641797 -0.091087
H -6.549385 2.751186 -1.352657
C -9.032209 1.884814 0.839022
H -9.079093 0.030828 1.938049
H -8.681323 3.614264 -0.400775
C -10.326057 2.411287 1.413056
H -10.168075 3.356336 1.946935
H -11.059440 2.608152 0.621720
H -10.772784 1.700738 2.114762
H -3.959439 2.023443 0.898513
C 2.477929 3.708869 0.129582
C 3.722571 3.098203 0.320677
C 2.355189 4.675144 -0.879242
C 4.820372 3.443833 -0.471675
H 3.837196 2.344916 1.096214
C 3.446276 5.020532 -1.676433
H 1.395954 5.164257 -1.037523
C 4.684031 4.405666 -1.473556
H 5.778929 2.962052 -0.298375
H 3.331747 5.773978 -2.451770
H 5.536643 4.678138 -2.090235
H -3.614559 -0.837186 0.206097
H 1.616687 2.748640 1.853840
89
-3063.40916101
C 2.687009 -1.222899 -2.685556
C 4.208310 -1.455351 -2.626075
C 4.940122 -0.716577 -1.496589
C 6.456590 -0.996541 -1.501993
C 1.842232 -2.091757 -1.746571
N 2.087909 -1.813783 -0.311297
H 4.406405 -2.534702 -2.549656
H 4.639216 -1.137440 -3.586056
H 4.520095 -1.005779 -0.527666
H 4.772146 0.364348 -1.596300
H 0.774622 -1.981429 -1.983637
H 2.327189 -1.458263 -3.696511
H 2.455722 -0.163176 -2.521902
H 6.617409 -2.074360 -1.359606
S 1.655828 -3.087891 0.801136
O 1.960025 -2.578961 2.133612
O 2.333706 -4.256453 0.235144
C -0.111237 -3.359034 0.692121
C -0.964657 -2.717658 1.592802
C -0.616049 -4.229243 -0.278768
C -2.336856 -2.954339 1.510135
H -0.551420 -2.074792 2.362209
C -1.989648 -4.444806 -0.350451
H 0.062096 -4.752685 -0.944013
C -2.873813 -3.812945 0.537028
H -3.002160 -2.483623 2.230809
H -2.383110 -5.126821 -1.099586
C -4.361592 -4.042259 0.437335
H -4.816814 -3.313744 -0.245615
H -4.584492 -5.043165 0.053898
H -4.847582 -3.934288 1.412840
H 2.122092 -3.140918 -1.878765
C 7.208114 -0.224607 -0.437894
C 7.125173 -0.598564 0.911676
C 7.980251 0.897248 -0.767326
C 7.788459 0.128598 1.900701
H 6.535218 -1.469548 1.189091
C 8.647781 1.628168 0.218255
H 8.062920 1.198263 -1.809757
C 8.552499 1.246843 1.557436
H 7.711994 -0.180915 2.939972
H 9.245739 2.492036 -0.061538
H 9.071971 1.812296 2.326535
H 6.867969 -0.749898 -2.490105
Cl 1.397704 -0.248336 0.164076
C -2.024226 1.493011 0.367224
C -1.281750 2.666624 1.023534
C 0.094398 2.893560 0.481272
C 1.049713 3.805133 1.196187
C -3.420903 1.283724 0.960573
N -4.132720 0.094420 0.456798
H -1.226654 2.511781 2.113333
H -1.888338 3.586074 0.898616
H 0.281012 2.676052 -0.568832
H -3.358941 1.170611 2.050521
H -2.115080 1.657399 -0.712074
H -1.437018 0.575182 0.502365
H 0.650013 4.837310 1.181149
S -4.975287 0.197517 -1.001080
O -5.149675 -1.202211 -1.416120
O -4.375454 1.172891 -1.922291
C -6.550798 0.874337 -0.478167
C -7.316217 0.198658 0.473822
C -7.022249 2.041966 -1.079908
C -8.561523 0.710071 0.830039
H -6.938612 -0.709034 0.932676
C -8.271016 2.538618 -0.711621
H -6.412606 2.544637 -1.822761
C -9.058990 1.884135 0.245719
H -9.159229 0.188768 1.573443
H -8.639740 3.449425 -1.177462
C -10.410634 2.435279 0.633019
H -10.896616 1.812464 1.389828
H -10.324193 3.450773 1.038362
H -11.078753 2.491720 -0.234935
H -4.059314 2.154488 0.775297
C 2.452729 3.811774 0.621326
C 3.415990 2.901862 1.075316
C 2.807468 4.711765 -0.391559
C 4.702177 2.889172 0.533344
H 3.155960 2.195544 1.860258
C 4.091331 4.702169 -0.939834
H 2.072522 5.429724 -0.750560
C 5.042307 3.790513 -0.477908
H 5.438452 2.179562 0.899584
H 4.349110 5.410889 -1.723044
H 6.044813 3.780329 -0.896765
H -3.555134 -0.745881 0.420829
H 1.083385 3.532004 2.262203
89
-3063.40892257
C 0.707402 -2.684761 0.800611
C 1.983025 -3.459161 1.180079
C 3.291291 -2.898181 0.604303
C 4.519260 -3.731255 1.023869
C 0.423808 -1.417408 1.616263
N 1.426177 -0.355661 1.385390
H 2.058700 -3.512373 2.276417
H 1.868060 -4.497631 0.836565
H 3.425587 -1.859918 0.923594
H 3.228541 -2.877285 -0.492896
H -0.593238 -1.058776 1.408307
H -0.165188 -3.333461 0.956590
H 0.715000 -2.440040 -0.268887
H 4.581542 -3.739132 2.120868

S 1.518365 0.875303 2.594919
O 2.510804 1.841434 2.124432
O 1.712246 0.124514 3.841372
C -0.064513 1.710195 2.686310
C -0.250406 2.907218 1.994127
C -1.104761 1.144309 3.430216
C -1.497444 3.531065 2.038905
H 0.579217 3.346966 1.451876
C -2.344291 1.775020 3.453939
H -0.937361 0.232581 3.993096
C -2.564196 2.972157 2.755379
H -1.641566 4.472319 1.513341
H -3.159078 1.328868 4.017254
C -3.925949 3.621525 2.772131
H -4.655780 2.976831 2.268584
H -4.280465 3.777626 3.797883
H -3.915669 4.593279 2.268199
H 0.493002 -1.655288 2.684201
C 5.818802 -3.211277 0.446559
C 6.472990 -2.115587 1.028126
C 6.384930 -3.789295 -0.697091
C 7.654730 -1.613006 0.483693
H 6.050275 -1.653177 1.917627
C 7.567632 -3.290652 -1.246434
H 5.894240 -4.643318 -1.160046
C 8.207336 -2.199513 -0.657063
H 8.146617 -0.764749 0.953295
H 7.990649 -3.757724 -2.132548
H 9.130147 -1.811188 -1.080351
H 4.372365 -4.775131 0.714184
Cl 1.112076 0.447556 -0.333025
C -1.694498 0.355748 -2.416968
C -1.016642 1.722901 -2.246680
C 0.481179 1.691043 -2.342733
C 1.249599 2.976388 -2.176922
C -3.226656 0.424983 -2.474444
N -3.915279 0.871473 -1.249893
H -1.301086 2.184341 -1.289262
H -1.389478 2.411142 -3.030322
H 0.920241 0.939991 -2.998835
H -3.547670 1.108123 -3.271504
H -1.351318 -0.095215 -3.358781
H -1.390682 -0.315438 -1.609350
H 0.795866 3.720912 -2.856665
S -4.187391 -0.222286 0.005036
O -4.601684 0.621972 1.133375
O -3.081535 -1.179818 0.164301
C -5.594680 -1.145302 -0.609450
C -6.801817 -0.481549 -0.851687
C -5.484944 -2.522835 -0.786754
C -7.900402 -1.213795 -1.285890
H -6.872478 0.591019 -0.704117
C -6.599410 -3.241535 -1.223453
H -4.541302 -3.016880 -0.582681
C -7.818715 -2.603916 -1.477932
H -8.840175 -0.701807 -1.478522
H -6.516981 -4.315977 -1.365790
C -9.027752 -3.384153 -1.935145
H -9.482925 -2.930177 -2.823070
H -8.769363 -4.418912 -2.178640
H -9.799089 -3.408029 -1.154951
H -3.636095 -0.558223 -2.728020
C 2.733647 2.878264 -2.471116
C 3.664530 2.668517 -1.445438
C 3.194793 2.983123 -3.790242
C 5.025288 2.560612 -1.737918
H 3.326585 2.575290 -0.416884
C 4.554606 2.875601 -4.083720
H 2.482743 3.156782 -4.595353
C 5.474499 2.663119 -3.055476
H 5.733618 2.392003 -0.931450
H 4.894636 2.962452 -5.112608
H 6.534612 2.579236 -3.279755
H -3.566485 1.736361 -0.841246
H 1.081802 3.369835 -1.165243

89
-3063.40889221
C 0.707980 -2.685386 0.801369
C 1.983972 -3.459139 1.180934
C 3.291914 -2.897802 0.604768
C 4.520312 -3.730202 1.024414
C 0.423923 -1.417975 1.616772
N 1.426143 -0.356086 1.385941
H 2.059865 -3.511913 2.277277
H 1.869374 -4.497785 0.837833
H 3.425811 -1.859387 0.923731
H 3.228993 -2.877272 -0.492427
H -0.593163 -1.059587 1.408572
H -0.164332 -3.334420 0.957509
H 0.715442 -2.440869 -0.268177
H 4.582762 -3.737703 2.121405
S 1.517936 0.875079 2.595289
O 2.510604 1.841074 2.125009
O 1.711312 0.124497 3.841947
C -0.064937 1.710075 2.685923
C -0.250293 2.907184 1.993746
C -1.105670 1.144259 3.429211
C -1.497258 3.531222 2.037915
H 0.579723 3.346841 1.452024
C -2.345131 1.775133 3.452299
H -0.938719 0.232447 3.992090
C -2.564466 2.972404 2.753778
H -1.640946 4.472562 1.512388
H -3.160314 1.329034 4.015062
C -3.926092 3.622055 2.770010
H -4.656012 2.977246 2.266749
H -4.280680 3.778788 3.795645
H -3.915543 4.593546 2.265578
H 0.492966 -1.655693 2.684755
C 5.819527 -3.209788 0.446766
C 6.473555 -2.113948 1.028226
C 6.385471 -3.787521 -0.697122
C 7.654964 -1.610942 0.483464
H 6.050979 -1.651759 1.917908
C 7.567840 -3.288454 -1.246793
H 5.894903 -4.641658 -1.159997
C 8.207389 -2.197167 -0.657523
H 8.146738 -0.762580 0.952995
H 7.990725 -3.755311 -2.133083
H 9.129945 -1.808516 -1.081068
H 4.373849 -4.774242 0.715075
Cl 1.112238 0.446872 -0.332631
C -1.694436 0.355264 -2.416241
C -1.016352 1.722348 -2.246280
C 0.481453 1.690193 -2.342469
C 1.250166 2.975384 -2.176822
C -3.226579 0.424720 -2.473731
N -3.915115 0.871404 -1.249181
H -1.300619 2.184016 -1.288919
H -1.389146 2.410506 -3.030013
H 0.920287 0.938997 -2.998560
H -3.547529 1.107814 -3.270856
H -1.351308 -0.095998 -3.357929
H -1.390739 -0.315765 -1.608448
H 0.796485 3.719976 -2.856524
S -4.187705 -0.222481 0.005548
O -4.601970 0.621688 1.133963
O -3.082139 -1.180343 0.164838
C -5.595147 -1.145009 -0.609317
C -6.802068 -0.480908 -0.851572
C -5.485701 -2.522533 -0.786990
C -7.900767 -1.212785 -1.286163
H -6.872484 0.591648 -0.703794
C -6.600252 -3.240844 -1.224058
H -4.542182 -3.016836 -0.582970
C -7.819397 -2.602864 -1.478505
H -8.840364 -0.700514 -1.478892
H -6.518035 -4.315258 -1.366745
C -9.028654 -3.382866 -1.935527
H -9.796550 -3.413351 -1.152140

H -9.488631 -2.924386 -2.818606
H -8.769089 -4.415566 -2.186439
H -3.636130 -0.558468 -2.727198
C 2.734147 2.876921 -2.471243
C 3.665146 2.667133 -1.445678
C 3.195110 2.981471 -3.790457
C 5.025833 2.558864 -1.738351
H 3.327330 2.574155 -0.417059
C 4.554853 2.873594 -4.084129
H 2.482973 3.155156 -4.595484
C 5.474859 2.661059 -3.055997
H 5.734252 2.390201 -0.931972
H 4.894742 2.960205 -5.113083
H 6.534917 2.576890 -3.280432
H -3.565850 1.736068 -0.840439
H 1.082639 3.368903 -1.165126
89
-3063.40886236
C 3.801331 1.144134 -0.066708
C 4.530977 0.614636 -1.312508
C 5.595421 -0.456075 -1.033662
C 6.372005 -0.867306 -2.301313
C 2.957091 0.076309 0.636253
N 2.238755 0.651543 1.788464
H 3.794017 0.219564 -2.027791
H 5.009181 1.466750 -1.814875
H 5.135632 -1.353213 -0.598091
H 6.305074 -0.083425 -0.281831
H 2.265297 -0.404111 -0.072083
H 3.153998 1.978066 -0.358791
H 4.524739 1.543982 0.654838
H 5.659151 -1.228798 -3.054591
S 1.725184 -0.449370 3.005857
O 1.010946 0.340673 4.005093
O 2.949709 -1.189235 3.336109
C 0.557025 -1.630078 2.316846
C -0.801028 -1.500211 2.615323
C 1.015569 -2.699885 1.543841
C -1.699328 -2.449004 2.127453
H -1.130952 -0.685263 3.250396
C 0.100158 -3.624621 1.044315
H 2.077440 -2.826168 1.362134
C -1.269731 -3.517540 1.322832
H -2.751143 -2.373886 2.397032
H 0.458203 -4.456163 0.442893
C -2.258712 -4.517318 0.773612
H -2.821980 -4.092884 -0.067454
H -1.755009 -5.420552 0.416205
H -2.987168 -4.814351 1.536744
H 3.608953 -0.697024 1.054976
C 7.417628 -1.930366 -2.039080
C 8.711173 -1.579959 -1.628403
C 7.108242 -3.291329 -2.164982
C 9.666230 -2.557850 -1.348415
H 8.972842 -0.528115 -1.531044
C 8.059284 -4.274012 -1.886455
H 6.111439 -3.583259 -2.489957
C 9.342805 -3.909999 -1.476053
H 10.664512 -2.263566 -1.034448
H 7.798759 -5.323911 -1.994401
H 10.086046 -4.673250 -1.261378
H 6.849744 0.024442 -2.729128
Cl 0.779043 1.743598 1.143385
C -2.500543 1.237952 0.065479
C -2.217744 2.639363 0.620583
C -0.914655 3.231145 0.175219
C -0.491438 4.567168 0.726879
C -3.864044 0.698472 0.511575
N -4.096437 -0.715154 0.168821
H -2.265270 2.636133 1.719380
H -3.021848 3.328105 0.295894
H -0.591265 2.997076 -0.838177
H -3.965429 0.774085 1.601733
H -2.460283 1.245633 -1.029234
H -1.716813 0.554998 0.413187
H -1.347325 5.256228 0.603097
S -4.620586 -1.117662 -1.384117
O -4.224853 -2.524306 -1.542927
O -4.216576 -0.109546 -2.374484
C -6.402878 -1.012749 -1.228881
C -7.068743 -1.838429 -0.317406
C -7.109728 -0.145455 -2.058527
C -8.454842 -1.778413 -0.237076
H -6.505228 -2.511892 0.319814
C -8.502068 -0.098803 -1.963968
H -6.572514 0.478163 -2.764681
C -9.194448 -0.909615 -1.058158
H -8.976044 -2.416221 0.472900
H -9.056536 0.578823 -2.608188
C -10.700921 -0.868118 -0.964860
H -11.143525 -1.812482 -1.305975
H -11.032206 -0.713106 0.068707
H -11.119040 -0.064181 -1.577826
H -4.682328 1.285556 0.080795
C 0.729880 5.173252 0.065362
C 1.982565 5.142257 0.689800
C 0.623784 5.772974 -1.197018
C 3.103078 5.694472 0.065988
H 2.081411 4.676929 1.666702
C 1.741611 6.323783 -1.824193
H -0.345036 5.814823 -1.691443
C 2.986374 6.286031 -1.192785
H 4.067118 5.663095 0.566791
H 1.639084 6.787425 -2.801919
H 3.857944 6.718323 -1.677066
H -3.306010 -1.319568 0.392386
H -0.343252 4.484233 1.811602
89
-3063.40864119
C 3.037175 -1.233407 -2.624683
C 4.535081 -1.488418 -2.370893
C 5.133282 -0.707628 -1.192561
C 6.624965 -1.025812 -0.968833
C 2.072679 -2.054355 -1.759785
N 2.163347 -1.725277 -0.323812
H 4.697745 -2.565840 -2.219684
H 5.089380 -1.224643 -3.282925
H 4.571905 -0.929646 -0.279422
H 5.016224 0.369376 -1.374134
H 1.042453 -1.938775 -2.126085
H 2.798267 -1.498615 -3.663844
H 2.809996 -0.165299 -2.522292
H 6.728575 -2.098720 -0.754308
S 1.598308 -2.927115 0.770123
O 1.701456 -2.353503 2.109232
O 2.356175 -4.123586 0.382233
C -0.138212 -3.270514 0.456942
C -1.106679 -2.737900 1.309737
C -0.504420 -4.108343 -0.600407
C -2.449928 -3.045937 1.091269
H -0.799118 -2.123046 2.148583
C -1.852016 -4.387595 -0.816369
H 0.257528 -4.561697 -1.225080
C -2.848074 -3.862306 0.019940
H -3.202001 -2.664502 1.778964
H -2.135365 -5.040320 -1.637995
C -4.307415 -4.158571 -0.229914
H -4.434228 -5.095043 -0.782024
H -4.863427 -4.239686 0.710450
H -4.774921 -3.359917 -0.820904
H 2.343650 -3.113610 -1.835863
C 7.240614 -0.220757 0.156556
C 6.928406 -0.505445 1.494550
C 8.114454 0.842736 -0.106493
C 7.469402 0.250254 2.534766
H 6.255912 -1.329806 1.721874
C 8.659733 1.602586 0.931729
H 8.376468 1.072963 -1.137378
C 8.337461 1.309027 2.257281
H 7.215732 0.009265 3.564033

H 9.340330 2.419266 0.703573
H 8.761867 1.896282 3.067367
H 7.181742 -0.840424 -1.897384
Cl 1.329771 -0.013035 -0.011776
C -1.976829 1.067933 0.159029
C -0.997102 1.940854 0.952620
C 0.307053 2.213445 0.266406
C 1.378563 2.954494 1.020627
C -3.313595 0.894607 0.889780
N -4.207480 -0.115108 0.298676
H -0.806446 1.500248 1.942307
H -1.469865 2.921205 1.157951
H 0.275360 2.354054 -0.813054
H -3.140544 0.582640 1.927779
H -2.161631 1.498784 -0.830949
H -1.525519 0.081651 0.002309
H 0.893456 3.826597 1.498117
S -5.156130 0.290431 -1.036484
O -5.477352 -1.004589 -1.653150
O -4.550311 1.368373 -1.830989
C -6.626915 0.969809 -0.270090
C -7.399955 0.168564 0.576292
C -7.011424 2.274751 -0.569251
C -8.560952 0.693361 1.131286
H -7.090253 -0.847631 0.796713
C -8.181059 2.785185 -0.002539
H -6.401536 2.873825 -1.236569
C -8.971411 2.008348 0.851394
H -9.163028 0.074673 1.792545
H -8.482618 3.804035 -0.231959
C -10.243010 2.556178 1.453749
H -11.123933 2.037969 1.054315
H -10.258267 2.423271 2.541918
H -10.360308 3.623068 1.241958
H -3.863917 1.840586 0.937720
C 2.546472 3.437413 0.185046
C 2.360927 4.446079 -0.771215
C 3.828822 2.904445 0.356634
C 3.428793 4.906615 -1.541047
H 1.372404 4.880585 -0.909411
C 4.902897 3.366576 -0.407959
H 3.991227 2.119231 1.090278
C 4.704516 4.367803 -1.359743
H 3.266207 5.690523 -2.276368
H 5.891025 2.941690 -0.254345
H 5.539089 4.729671 -1.954565
H -3.738802 -0.996225 0.088965
H 1.733622 2.335200 1.855031
89
-3063.40863897
C 3.037010 -1.233342 -2.624661
C 4.534931 -1.488365 -2.370976
C 5.133198 -0.707646 -1.192632
C 6.624880 -1.025886 -0.968973
C 2.072610 -2.054375 -1.759747
N 2.163333 -1.725334 -0.323770
H 4.697581 -2.565791 -2.219833
H 5.089191 -1.224550 -3.283020
H 4.571849 -0.929679 -0.279480
H 5.016176 0.369370 -1.374163
H 1.042358 -1.938867 -2.125998
H 2.798045 -1.498493 -3.663824
H 2.809808 -0.165247 -2.522191
H 6.728461 -2.098786 -0.754386
S 1.598349 -2.927226 0.770113
O 1.701568 -2.353716 2.109260
O 2.356194 -4.123666 0.382079
C -0.138190 -3.270600 0.457018
C -1.106605 -2.737981 1.309870
C -0.504466 -4.108412 -0.600322
C -2.449874 -3.045978 1.091457
H -0.798991 -2.123150 2.148712
C -1.852078 -4.387632 -0.816220
H 0.257445 -4.561772 -1.225036
C -2.848087 -3.862319 0.020134
H -3.201911 -2.664529 1.779187
H -2.135475 -5.040342 -1.637842
C -4.307449 -4.158502 -0.229691
H -4.774863 -3.359949 -0.820890
H -4.434364 -5.095106 -0.781557
H -4.863492 -4.239320 0.710680
H 2.343667 -3.113605 -1.835856
C 7.240628 -0.220765 0.156314
C 8.114496 0.842670 -0.106879
C 6.928485 -0.505325 1.494351
C 8.659867 1.602589 0.931244
H 8.376459 1.072795 -1.137801
C 7.469568 0.250448 2.534469
H 6.255988 -1.329651 1.721785
C 8.337647 1.309166 2.256839
H 9.340483 2.419223 0.702980
H 7.215944 0.009570 3.563773
H 8.762126 1.896470 3.066852
H 7.181600 -0.840578 -1.897574
Cl 1.329740 -0.013083 -0.011646
C -1.976789 1.067717 0.159245
C -0.997134 1.940738 0.952814
C 0.307045 2.213324 0.266642
C 1.378548 2.954349 1.020901
C -3.313607 0.894445 0.889910
N -4.207492 -0.115230 0.298732
H -0.806512 1.500225 1.942548
H -1.469936 2.921094 1.158030
H 0.275376 2.353959 -0.812816
H -3.140650 0.582443 1.927915
H -2.161523 1.498464 -0.830790
H -1.525452 0.081428 0.002664
H 0.893409 3.826347 1.498546
S -5.156039 0.290341 -1.036494
O -5.477296 -1.004678 -1.653147
O -4.550113 1.368228 -1.830993
C -6.626825 0.969823 -0.270196
C -7.399949 0.168648 0.576176
C -7.011257 2.274771 -0.569432
C -8.560950 0.693522 1.131087
H -7.090302 -0.847551 0.796659
C -8.180899 2.785282 -0.002802
H -6.401304 2.873789 -1.236740
C -8.971333 2.008516 0.851118
H -9.163091 0.074888 1.792339
H -8.482397 3.804138 -0.232277
C -10.242948 2.556420 1.453369
H -10.258399 2.423309 2.541509
H -10.360061 3.623365 1.241754
H -11.123883 2.038414 1.053696
H -3.863888 1.840449 0.937825
C 2.546370 3.437456 0.185308
C 3.828743 2.904469 0.356657
C 2.360711 4.446309 -0.770735
C 4.902731 3.366758 -0.407965
H 3.991236 2.119115 1.090133
C 3.428490 4.907007 -1.540593
H 1.372167 4.880829 -0.908740
C 4.704238 4.368168 -1.359531
H 5.890879 2.941852 -0.254540
H 3.265819 5.691056 -2.275745
H 5.538751 4.730152 -1.954369
H -3.738829 -0.996355 0.089023
H 1.733704 2.334960 1.855195
89
-3063.40845794
C 1.608608 -3.020435 -0.674764
C 2.975256 -3.610189 -0.278920
C 4.162264 -2.643551 -0.399005
C 5.498675 -3.296807 0.006940
C 0.885693 -2.213791 0.410041
N 1.632937 -1.003439 0.816306
H 2.918959 -3.995402 0.749970
H 3.171584 -4.483742 -0.917217
H 3.985720 -1.758054 0.219853

H 4.238144 -2.287267 -1.436196
H -0.129364 -1.964556 0.076533
H 0.922355 -3.838113 -0.932778
H 1.705496 -2.412279 -1.582620
H 5.423081 -3.637024 1.048922
S 1.178643 -0.314010 2.328531
O 1.962598 0.911322 2.479798
O 1.310273 -1.426756 3.277158
C -0.557460 0.148891 2.277975
C -0.920576 1.427613 1.851729
C -1.527207 -0.772229 2.682481
C -2.271852 1.776098 1.823689
H -0.158905 2.145940 1.570780
C -2.870426 -0.405664 2.650511
H -1.225972 -1.751331 3.038686
C -3.266421 0.870532 2.221948
H -2.554370 2.778845 1.511086
H -3.625707 -1.119182 2.969935
C -4.724178 1.264648 2.226391
H -5.351903 0.478167 1.793878
H -5.078756 1.438329 3.250720
H -4.892258 2.185371 1.659337
H 0.812564 -2.822365 1.319274
C 6.682789 -2.364352 -0.138169
C 6.955032 -1.395168 0.838284
C 7.515420 -2.422174 -1.263395
C 8.024356 -0.511155 0.694386
H 6.321119 -1.334826 1.720481
C 8.587522 -1.540301 -1.412641
H 7.322992 -3.170663 -2.029659
C 8.845785 -0.580532 -0.433110
H 8.218359 0.230447 1.465312
H 9.223008 -1.606591 -2.292453
H 9.681673 0.105279 -0.544761
H 5.662993 -4.196134 -0.602277
Cl 1.512409 0.319865 -0.582108
C -1.032127 1.146353 -3.130249
C -0.307062 2.215233 -2.298685
C 1.184948 2.056926 -2.291082
C 2.107637 3.176269 -1.877612
C -2.564807 1.203529 -3.014782
N -3.146653 0.553976 -1.822186
H -0.674005 2.227051 -1.261938
H -0.558875 3.214930 -2.694270
H 1.606410 1.382985 -3.036765
H -2.910057 2.245180 -2.999370
H -0.760139 1.279530 -4.186081
H -0.685422 0.147116 -2.842296
H 3.079373 2.746043 -1.606408
S -3.321291 -1.131111 -1.867653
O -2.255329 -1.792157 -1.098293
O -3.539845 -1.481958 -3.275410
C -4.848812 -1.370936 -0.959707
C -4.875645 -2.297334 0.081108
C -6.009706 -0.697980 -1.350832
C -6.080384 -2.543297 0.742197
H -3.963161 -2.809229 0.365723
C -7.200845 -0.952888 -0.679075
H -5.974972 0.022745 -2.160967
C -7.258253 -1.881252 0.374011
H -6.104136 -3.265587 1.554516
H -8.103982 -0.425454 -0.976580
C -8.565902 -2.171950 1.070842
H -9.192260 -2.839903 0.465280
H -8.406570 -2.658640 2.037910
H -9.142369 -1.255967 1.240702
H -3.027553 0.722218 -3.878071
C 1.617020 4.101136 -0.779042
C 0.953636 5.294667 -1.093917
C 1.831375 3.788898 0.571386
C 0.502102 6.152487 -0.088918
H 0.799445 5.563023 -2.137445
C 1.381598 4.646075 1.578048
H 2.345405 2.871709 0.843313
C 0.713835 5.828901 1.251998
H -0.005783 7.076032 -0.355098
H 1.560703 4.384595 2.617463
H 0.370110 6.497686 2.036783
H -2.707385 0.821553 -0.940773
H 2.298924 3.776800 -2.785877

TS-1,6’-XAT$_{bi}$

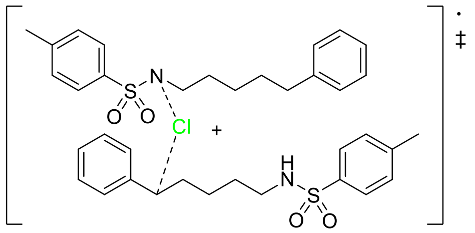

89
-3063.42768447
C -2.960640 -0.379257 2.966159
C -4.096075 0.644605 2.832242
C -5.495761 0.048988 3.056443
C -6.004595 -0.890129 1.936594
C -1.559742 0.219879 2.821771
N -1.363553 0.836363 1.481380
H -3.933133 1.445400 3.567136
H -4.048205 1.119538 1.845628
H -6.215263 0.870351 3.170341
H -5.507063 -0.503059 4.007349
H -0.787249 -0.535047 3.019081
H -3.080323 -1.193919 2.243011
H -2.998954 -0.845780 3.960981
H -6.933762 -1.359529 2.285819
S 0.042172 1.878568 1.410589
O 1.288612 1.090583 1.339550
O -0.156436 2.809413 2.524747
C -0.201948 2.725743 -0.136335
C 0.522850 2.342471 -1.267534
C -1.104766 3.790797 -0.169795
C 0.324361 3.047508 -2.451960
H 1.225908 1.516632 -1.235590
C -1.286531 4.477486 -1.367190
H -1.646062 4.077849 0.725154
C -0.578059 4.119117 -2.523143
H 0.885992 2.754879 -3.335442
H -1.987370 5.307347 -1.403550
C -0.769441 4.872524 -3.817388
H -1.024987 4.191767 -4.637860
H -1.566094 5.617837 -3.735444
H 0.150259 5.394947 -4.108752
H -1.428615 1.043353 3.530137
C -6.266924 -0.187712 0.619276
C -5.366119 -0.277699 -0.450212
C -7.422860 0.589091 0.448759
C -5.608587 0.393415 -1.651088
H -4.467442 -0.881408 -0.348187
C -7.670494 1.258689 -0.749144
H -8.140408 0.663506 1.263868
C -6.761177 1.163948 -1.805317
H -4.892994 0.311799 -2.465409
H -8.575862 1.850227 -0.860507
H -6.952820 1.683321 -2.740671
H -5.289630 -1.707224 1.781393
Cl -1.247579 -0.413409 0.220513
C 2.543464 -3.434008 1.670049
C 2.773854 -4.583052 0.671109
C 1.992018 -4.492938 -0.658144
C 0.522414 -4.727283 -0.498839
C 3.318201 -2.139802 1.377739
N 2.751452 -1.359536 0.262686
H 2.509109 -5.529618 1.163501
H 3.846209 -4.647395 0.443560
H 2.396747 -5.274641 -1.324930
H 2.207635 -3.543208 -1.150019
H 3.341359 -1.508722 2.275594
H 1.471468 -3.208961 1.749289
H 2.863645 -3.769159 2.666643
H 0.243197 -5.591395 0.106135
S 3.736981 -0.808840 -0.961669
O 2.865915 0.023812 -1.805413
O 4.439413 -1.970540 -1.516639
C 4.994446 0.252052 -0.229344
C 4.617002 1.388370 0.494552
C 6.339791 -0.063638 -0.412819
C 5.605263 2.204386 1.035930
H 3.568501 1.628347 0.642069
C 7.316428 0.770002 0.134421
H 6.608919 -0.947843 -0.980190
C 6.968292 1.911317 0.865667
H 5.313960 3.086770 1.601024
H 8.366133 0.527156 -0.011680
C 8.025273 2.803300 1.472358
H 7.844478 3.857223 1.231095
H 8.032229 2.720992 2.566870
H 9.025323 2.541200 1.113804
H 4.356124 -2.388778 1.132621
C -0.539469 -4.029887 -1.121518
C -0.355678 -2.866987 -1.930192
C -1.878831 -4.499438 -0.962441
C -1.436139 -2.247549 -2.545878
H 0.639108 -2.454737 -2.063183
C -2.948263 -3.872062 -1.581496
H -2.049760 -5.380541 -0.347795
C -2.739195 -2.742263 -2.385432
H -1.263487 -1.368277 -3.161331
H -3.953970 -4.262636 -1.446780
H -3.577088 -2.256171 -2.877175
H 2.077116 -0.643904 0.537384
89
-3063.42332482
C -0.868598 -4.278725 -1.643289
C -2.335604 -4.067413 -2.063847
C -3.137001 -3.122774 -1.156947
C -4.602549 -2.959713 -1.607279
C 0.120946 -3.200225 -2.102053
N -0.195539 -1.867850 -1.560559
H -2.834706 -5.047029 -2.077897
H -2.366717 -3.697541 -3.099206
H -2.653765 -2.141363 -1.128879
H -3.120568 -3.506676 -0.126961
H 1.148308 -3.502017 -1.850728
H -0.801672 -4.398163 -0.555022
H -0.501784 -5.218985 -2.077571
H -4.617449 -2.571227 -2.634652
S 0.570668 -0.554144 -2.337588
O 0.161123 0.648322 -1.593622
O 0.208193 -0.694067 -3.754293
C 2.350532 -0.704743 -2.206024
C 3.028486 -0.092547 -1.148128
C 3.038863 -1.438113 -3.176482
C 4.411794 -0.232999 -1.068640
H 2.498796 0.507346 -0.416468
C 4.422287 -1.568110 -3.073845
H 2.501701 -1.870317 -4.013838
C 5.128604 -0.975186 -2.018299
H 4.941753 0.252584 -0.253331
H 4.962339 -2.129356 -3.832451
C 6.624925 -1.138165 -1.897682
H 7.077488 -1.419756 -2.853691

H 7.101151 -0.213592 -1.553977
H 6.877033 -1.921603 -1.170696
H 0.058218 -3.099805 -3.193148
C -5.390195 -2.035983 -0.702367
C -5.325992 -0.644942 -0.868046
C -6.167424 -2.542205 0.347872
C -6.014204 0.213722 -0.009820
H -4.731735 -0.232405 -1.680757
C -6.857826 -1.688401 1.210002
H -6.234644 -3.619233 0.488800
C -6.782758 -0.305681 1.034738
H -5.956421 1.288776 -0.161616
H -7.458307 -2.103968 2.015491
H -7.323617 0.361288 1.701048
H -5.086167 -3.945591 -1.639653
Cl 0.241098 -1.857589 0.410994
C -2.164406 0.241647 1.887564
C -1.681703 -0.432470 3.186796
C -0.161633 -0.598904 3.404921
C 0.500134 -1.827645 2.851316
C -1.634642 1.662843 1.641549
N -0.233624 1.626042 1.195113
H -2.170475 -1.413236 3.271983
H -2.060498 0.163152 4.028393
H 0.002955 -0.679642 4.495537
H 0.363188 0.307895 3.100359
H -2.268217 2.167629 0.897683
H -1.937614 -0.380099 1.016926
H -3.259306 0.305656 1.936321
H -0.097935 -2.737517 2.884801
S 0.783658 2.917591 1.435012
O 2.101056 2.458478 0.967884
O 0.591329 3.365625 2.819588
C 0.256068 4.256476 0.357559
C 0.297775 4.083921 -1.030515
C -0.191942 5.451185 0.918057
C -0.116332 5.127077 -1.852423
H 0.636209 3.146507 -1.461324
C -0.598236 6.487598 0.075936
H -0.214741 5.560874 1.996868
C -0.566154 6.344778 -1.315943
H -0.090893 4.994164 -2.931458
H -0.945777 7.421778 0.510534
C -0.990501 7.472570 -2.226517
H -1.498818 8.267002 -1.671439
H -0.124255 7.921527 -2.729554
H -1.669403 7.118869 -3.011018
H -1.669013 2.251757 2.563507
C 1.928408 -2.025072 2.909912
C 2.844548 -0.944729 2.953087
C 2.448386 -3.342967 2.937130
C 4.210360 -1.182427 3.046069
H 2.484829 0.076360 2.882317
C 3.813863 -3.572050 3.034889
H 1.758010 -4.181561 2.890076
C 4.702419 -2.491942 3.093842
H 4.897946 -0.341827 3.079565
H 4.191158 -4.590526 3.067041
H 5.771569 -2.670044 3.170777
H -0.079678 1.183440 0.284726
89
-3063.42298073
C -1.462220 4.702311 -0.598498
C -2.534615 5.302006 0.341151
C -3.823259 4.475610 0.500340
C -3.649855 3.088769 1.153665
C -0.414594 3.866733 0.142473
N 0.616861 3.351401 -0.798538
H -2.095838 5.494856 1.331254
H -2.821261 6.285471 -0.051030
H -4.293066 4.346345 -0.484619
H -4.536476 5.055331 1.101173
H -0.863306 3.046889 0.715253
H -0.929442 5.504978 -1.120774
H -1.947177 4.095766 -1.372740
H -2.997371 2.467356 0.528356
S 2.063439 2.791840 0.039508
O 1.816253 1.517780 0.739430
O 2.474499 3.968779 0.809456
C 3.189662 2.520841 -1.309736
C 3.493576 1.221148 -1.712958
C 3.777142 3.637186 -1.916068
C 4.404513 1.047326 -2.755640
H 3.041993 0.358393 -1.234132
C 4.678271 3.435856 -2.953955
H 3.534954 4.637272 -1.573091
C 5.005626 2.140301 -3.390597
H 4.646542 0.037435 -3.074905
H 5.140273 4.295960 -3.432381
C 5.991098 1.942911 -4.516866
H 6.978597 2.340704 -4.252069
H 6.110679 0.884237 -4.763870
H 5.666007 2.467190 -5.423598
H 0.137456 4.506336 0.837661
C -4.966673 2.369134 1.359364
C -5.718743 2.564128 2.525710
C -5.479921 1.515253 0.373285
C -6.947523 1.926919 2.702972
H -5.333737 3.218201 3.305695
C -6.708346 0.875043 0.545468
H -4.909092 1.348793 -0.538133
C -7.446876 1.079446 1.712282
H -7.512439 2.088300 3.617578
H -7.086027 0.213838 -0.230182
H -8.401529 0.578988 1.850308
H -3.140743 3.209658 2.119827
Cl -0.051179 2.033740 -1.793009
C -1.812894 -0.913219 1.399942
C -2.660504 -1.761520 0.436881
C -2.113523 -1.880050 -1.007102
C -3.021089 -2.671404 -1.896125
C -0.436810 -1.492678 1.763760
N 0.555192 -1.257863 0.701824
H -3.670251 -1.330221 0.399742
H -2.775986 -2.770775 0.855454
H -1.110051 -2.308298 -0.981849
H -1.998764 -0.862405 -1.412925
H -0.087849 -1.061779 2.713331
H -1.680400 0.100763 0.996458
H -2.373333 -0.796069 2.337284
H -3.953953 -2.191499 -2.193141
S 1.798335 -2.342658 0.472512
O 2.651883 -1.742568 -0.561892
O 1.193290 -3.666255 0.283542
C 2.749754 -2.413975 1.997687
C 3.420778 -1.270748 2.445420
C 2.808933 -3.605610 2.718133
C 4.152934 -1.334825 3.626699
H 3.365542 -0.343001 1.883568
C 3.552609 -3.651534 3.898302
H 2.281358 -4.479316 2.351175
C 4.235139 -2.523964 4.369554
H 4.671423 -0.446405 3.979573
H 3.601511 -4.580760 4.460755
C 5.056497 -2.582118 5.635581
H 4.817638 -3.470432 6.228389
H 6.129945 -2.615659 5.407905
H 4.888223 -1.699404 6.262778
H -0.519103 -2.575106 1.903621
C -2.821490 -4.000435 -2.341086
C -1.672283 -4.779207 -2.006646
C -3.804632 -4.621377 -3.170055
C -1.531458 -6.077726 -2.475579
H -0.894654 -4.360016 -1.375649
C -3.650617 -5.918429 -3.631602
H -4.690846 -4.051089 -3.440397
C -2.511567 -6.661486 -3.289514
H -0.645137 -6.646053 -2.204479
H -4.418279 -6.360703 -4.262187
H -2.390577 -7.678681 -3.651809

H 0.892204 -0.296218 0.628490
 89
-3063.42113128
C 2.820729 -3.572835 -0.866510
C 3.531111 -2.248932 -0.544119
C 3.829188 -1.363158 -1.760746
C 4.528247 -0.030504 -1.406338
C 1.423771 -3.428938 -1.486512
N 0.526613 -2.593489 -0.619487
H 2.920131 -1.684147 0.171843
H 4.472181 -2.484627 -0.032164
H 2.895226 -1.116724 -2.282877
H 4.451306 -1.917717 -2.478169
H 0.976453 -4.409608 -1.688648
H 2.745362 -4.178083 0.040320
H 3.418212 -4.151502 -1.586486
H 4.574324 0.586969 -2.312829
S -0.436984 -3.566529 0.502490
O 0.583854 -4.392932 1.154577
O -1.580895 -4.184278 -0.170692
C -1.012465 -2.319888 1.635775
C -0.166400 -1.905898 2.666179
C -2.297377 -1.790056 1.490492
C -0.618379 -0.932209 3.554862
H 0.816547 -2.351438 2.775861
C -2.724001 -0.811181 2.383618
H -2.950288 -2.140697 0.698710
C -1.894756 -0.363853 3.424629
H 0.027999 -0.612276 4.368414
H -3.716390 -0.386502 2.265137
C -2.367538 0.717475 4.363052
H -2.406609 1.676973 3.834784
H -3.376145 0.505710 4.735825
H -1.700934 0.823583 5.224582
H 1.488679 -2.902572 -2.439539
C 5.926141 -0.186299 -0.842676
C 7.019741 -0.387960 -1.697601
C 6.166622 -0.150688 0.538021
C 8.309200 -0.551393 -1.191722
H 6.857281 -0.410763 -2.773445
C 7.455732 -0.312934 1.050795
H 5.332357 0.002558 1.219471
C 8.532220 -0.514450 0.186624
H 9.141829 -0.702432 -1.874068
H 7.617393 -0.280635 2.125278
H 9.536693 -0.638358 0.582247
H 3.899549 0.516501 -0.691315
Cl -0.564976 -1.589639 -1.620540
C -1.919421 1.694280 -3.318748
C -3.364654 2.070206 -3.689573
C -4.450791 1.701398 -2.661504
C -4.578674 0.225350 -2.432760
C -1.265836 2.522377 -2.203762
N -1.815265 2.194607 -0.877943
H -3.606449 1.579580 -4.642963
H -3.411228 3.150272 -3.882979
H -5.411571 2.102259 -3.031048
H -4.253465 2.222436 -1.718333
H -0.175815 2.370914 -2.227444
H -1.861901 0.626848 -3.062750
H -1.289376 1.821237 -4.209089
H -4.217633 -0.434944 -3.221252
S -1.552625 3.251258 0.400741
O -2.140083 2.587742 1.569051
O -1.999878 4.570351 -0.051389
C 0.222194 3.357170 0.669769
C 0.934818 4.431230 0.136485
C 0.874867 2.359228 1.401067
C 2.313588 4.503189 0.338649
H 0.408602 5.208528 -0.407115
C 2.250701 2.448147 1.591902
H 0.310309 1.537342 1.830536
C 2.993376 3.518958 1.066308
H 2.867980 5.344650 -0.069232
H 2.757351 1.679345 2.170939
C 4.482694 3.609322 1.299941
H 5.002825 2.714633 0.937332
H 4.914958 4.476909 0.792649
H 4.708925 3.701331 2.369599
H -1.457767 3.586467 -2.371267
C -5.206936 -0.390927 -1.322934
C -5.786850 0.349059 -0.250641
C -5.289234 -1.813730 -1.247798
C -6.408503 -0.296173 0.810309
H -5.745504 1.433439 -0.265699
C -5.907451 -2.447485 -0.180653
H -4.852901 -2.404918 -2.049515
C -6.475571 -1.695532 0.858586
H -6.850228 0.294261 1.609443
H -5.951574 -3.533113 -0.152511
H -6.964145 -2.193200 1.691440
H -1.668237 1.230623 -0.581330
 89
-3063.41832885
C -1.707432 -3.702338 -1.376435
C -2.767408 -2.657645 -0.994920
C -3.799011 -3.138377 0.035143
C -4.785015 -2.035172 0.481632
C -0.730718 -4.154149 -0.280867
N 0.226924 -3.169151 0.286289
H -2.272426 -1.753952 -0.620919
H -3.289773 -2.360141 -1.912759
H -3.291042 -3.503359 0.935861
H -4.363088 -3.987287 -0.378258
H -0.097103 -4.957280 -0.673627
H -1.125598 -3.332849 -2.229272
H -2.205372 -4.618732 -1.726434
H -5.418976 -2.442459 1.280048
S -0.286873 -2.145680 1.591812
O -1.074249 -3.055704 2.433013
O -0.869888 -0.875918 1.136781
C 1.274560 -1.785259 2.378616
C 1.892187 -2.779221 3.139930
C 1.828688 -0.509398 2.259746
C 3.091638 -2.484596 3.783002
H 1.436733 -3.759324 3.233583
C 3.029653 -0.236975 2.911412
H 1.338644 0.263376 1.679182
C 3.679724 -1.215245 3.678172
H 3.576899 -3.251492 4.381261
H 3.455838 0.759010 2.823156
C 4.985635 -0.903945 4.369250
H 5.797046 -0.780517 3.640725
H 4.920750 0.030607 4.937945
H 5.276326 -1.702024 5.059008
H -1.253479 -4.551773 0.592682
C -5.665858 -1.486525 -0.622384
C -6.794123 -2.198717 -1.055421
C -5.376823 -0.265453 -1.247731
C -7.605463 -1.710583 -2.079685
H -7.042145 -3.144543 -0.577513
C -6.185853 0.228615 -2.273624
H -4.506120 0.303612 -0.928625
C -7.303796 -0.492881 -2.693725
H -8.477328 -2.278315 -2.395201
H -5.941786 1.178527 -2.742783
H -7.936802 -0.109206 -3.489523
H -4.205677 -1.219147 0.931897
Cl 1.147930 -2.283311 -0.931960
C 2.678333 3.380577 -2.250902
C 2.849831 2.467051 -3.475125
C 2.371410 1.003653 -3.266632
C 3.094115 0.284191 -2.167213
C 1.214736 3.702593 -1.923593
N 1.039225 4.560518 -0.739294
H 3.911231 2.446918 -3.751047
H 2.312064 2.894832 -4.333955
H 2.483795 0.472909 -4.220285
H 1.295823 1.001935 -3.046651
H 0.740092 4.216410 -2.769431

H 3.146410 2.923569 -1.372281
H 3.207211 4.326677 -2.437185
H 2.711000 0.414386 -1.157706
S 0.912112 3.846583 0.786109
O 1.394214 4.874292 1.716183
O 1.510288 2.499080 0.781668
C -0.855386 3.642854 0.987562
C -1.404941 2.362593 1.033485
C -1.659629 4.777728 1.124924
C -2.781109 2.224018 1.213435
H -0.779447 1.483954 0.934159
C -3.030960 4.618072 1.298276
H -1.216899 5.768062 1.097056
C -3.614512 3.341683 1.346386
H -3.203303 1.223499 1.258530
H -3.661063 5.498211 1.400706
C -5.099927 3.174403 1.561770
H -5.497702 2.337691 0.977455
H -5.322081 2.967232 2.616915
H -5.650566 4.078477 1.282849
H 0.639868 2.788941 -1.748930
C 4.292101 -0.460191 -2.309599
C 4.945654 -0.662956 -3.560926
C 4.900810 -1.047232 -1.159490
C 6.118942 -1.398884 -3.646763
H 4.520967 -0.233306 -4.463175
C 6.071695 -1.781154 -1.257840
H 4.421808 -0.912037 -0.192884
C 6.693811 -1.964708 -2.501275
H 6.595411 -1.536008 -4.614406
H 6.509365 -2.217983 -0.363670
H 7.612073 -2.540351 -2.576049
H 1.710475 5.322950 -0.665541
89
-3063.41756606
C -3.936106 1.992426 -0.216560
C -4.871066 1.032587 0.531360
C -6.067816 1.721186 1.205763
C -7.088789 2.378346 0.248005
C -2.698140 1.303097 -0.802267
N -1.820815 0.731461 0.273523
H -5.235041 0.260530 -0.160593
H -4.290681 0.509243 1.300547
H -5.698679 2.491717 1.897164
H -6.594998 0.981848 1.823361
H -2.127824 1.977267 -1.453669
H -3.623361 2.811440 0.437801
H -4.459014 2.449164 -1.068299
H -7.843319 2.891715 0.859221
S -0.579609 1.867244 0.789946
O -1.352648 3.034354 1.227524
O 0.461939 2.026181 -0.234084
C 0.109363 1.037195 2.209459
C -0.605243 1.053770 3.410252
C 1.356510 0.420353 2.113304
C -0.056242 0.428779 4.525571
H -1.567874 1.550482 3.466494
C 1.888945 -0.197012 3.243882
H 1.910090 0.413756 1.182307
C 1.196293 -0.202790 4.462170
H -0.606542 0.433766 5.463214
H 2.861438 -0.675637 3.166070
C 1.795666 -0.846268 5.689347
H 1.022727 -1.272588 6.337485
H 2.497136 -1.642552 5.421690
H 2.350239 -0.109395 6.285385
H -3.010289 0.450747 -1.409508
C -7.780791 1.403913 -0.684497
C -8.726312 0.492248 -0.190523
C -7.494728 1.378164 -2.055589
C -9.360009 -0.417168 -1.036417
H -8.972656 0.500547 0.869432
C -8.125951 0.469543 -2.908251
H -6.773948 2.084472 -2.462447
C -9.060654 -0.432867 -2.400908
H -10.092034 -1.111503 -0.631590
H -7.888532 0.470641 -3.969138
H -9.555378 -1.139577 -3.061864
H -6.594599 3.159579 -0.342098
Cl -1.031693 -0.776240 -0.284510
C 4.008791 -2.809107 -2.242216
C 2.791414 -3.468387 -2.910100
C 1.414216 -2.933567 -2.429000
C 1.185239 -3.091614 -0.955567
C 4.194392 -1.333114 -2.617268
N 5.344377 -0.686541 -1.963433
H 2.830078 -4.547651 -2.717654
H 2.854559 -3.343254 -4.000996
H 0.633270 -3.446597 -3.003471
H 1.328097 -1.870301 -2.688461
H 4.339347 -1.229479 -3.700294
H 3.927373 -2.889796 -1.152829
H 4.915070 -3.359064 -2.534800
H 1.605313 -2.324660 -0.308821
S 5.160785 -0.005513 -0.428922
O 6.448620 -0.219416 0.241817
O 3.900528 -0.477927 0.172218
C 4.969067 1.745948 -0.746967
C 3.691072 2.296234 -0.864266
C 6.109102 2.541895 -0.865974
C 3.562319 3.660929 -1.110204
H 2.803994 1.684796 -0.749566
C 5.958959 3.905100 -1.113349
H 7.093928 2.102257 -0.748602
C 4.689199 4.485396 -1.241215
H 2.564138 4.084221 -1.187093
H 6.844069 4.530352 -1.200888
C 4.530321 5.964178 -1.503634
H 3.991765 6.144780 -2.441980
H 3.954360 6.448396 -0.705718
H 5.499788 6.467116 -1.570946
H 3.307251 -0.748889 -2.360727
C 0.579442 -4.204863 -0.320035
C 0.054238 -5.324832 -1.029458
C 0.478375 -4.235319 1.103486
C -0.526145 -6.391283 -0.357701
H 0.110858 -5.347190 -2.113551
C -0.104465 -5.304864 1.763266
H 0.864896 -3.390809 1.668793
C -0.612173 -6.394318 1.040649
H -0.917117 -7.232481 -0.924830
H -0.168488 -5.297511 2.848464
H -1.068621 -7.232345 1.559794
H 6.196988 -1.242612 -1.954232
89
-3063.41536825
C 1.436967 -3.771328 0.093055
C 2.501320 -2.663820 0.103190
C 2.819335 -2.065157 -1.272610
C 3.905382 -0.964884 -1.242148
C 0.044303 -3.344184 -0.416306
N -0.476377 -2.156780 0.306339
H 2.175228 -1.863349 0.779031
H 3.419583 -3.081420 0.534786
H 1.911147 -1.622791 -1.703190
H 3.134251 -2.863772 -1.960228
H -0.655622 -4.188850 -0.376488
H 1.336602 -4.184127 1.099680
H 1.761646 -4.592426 -0.563323
H 3.957650 -0.502544 -2.236811
S -1.344046 -2.603139 1.745539
O -0.301759 -3.175723 2.614802
O -2.554667 -3.379946 1.453605
C -1.807855 -1.007383 2.394860
C -0.868674 -0.280254 3.130275
C -3.091562 -0.511467 2.171599
C -1.216764 0.978955 3.610124
H 0.109529 -0.706024 3.327429
C -3.418865 0.751917 2.659819
H -3.812794 -1.103179 1.619404

C -2.486158 1.524864 3.363540
H -0.491948 1.553588 4.181745
H -4.413647 1.149857 2.476812
C -2.815951 2.927766 3.805861
H -2.451007 3.641901 3.057158
H -3.895399 3.074497 3.914162
H -2.338308 3.173483 4.760515
H 0.119957 -3.034658 -1.461030
C 5.285350 -1.456325 -0.854829
C 6.117252 -2.066896 -1.804618
C 5.763711 -1.332054 0.457291
C 7.382772 -2.539673 -1.456966
H 5.769188 -2.167037 -2.830913
C 7.030075 -1.803129 0.811648
H 5.133148 -0.863942 1.210061
C 7.844658 -2.409200 -0.145432
H 8.011138 -3.006009 -2.211626
H 7.378714 -1.696227 1.835775
H 8.831306 -2.774480 0.126663
H 3.587903 -0.175121 -0.549101
Cl -1.832792 -1.264527 -0.951900
C -0.602616 0.885180 -3.532840
C -2.048980 1.199089 -3.956220
C -3.153890 1.045833 -2.892446
C -3.554392 -0.364292 -2.554777
C 0.079015 1.942305 -2.653168
N -0.590393 2.096694 -1.351523
H -2.303841 0.562358 -4.815052
H -2.084116 2.231830 -4.327593
H -4.058256 1.542960 -3.282169
H -2.883712 1.604183 -1.989367
H 1.140942 1.676928 -2.529158
H -0.547767 -0.093431 -3.044156
H 0.009984 0.807261 -4.441109
H -3.217622 -1.147718 -3.230541
S -0.201140 3.414348 -0.385630
O -1.033903 3.277917 0.812489
O -0.269917 4.595579 -1.249712
C 1.513451 3.220989 0.118163
C 2.518184 3.889640 -0.581948
C 1.826448 2.388622 1.197139
C 3.848142 3.715768 -0.198261
H 2.253918 4.554078 -1.397654
C 3.159486 2.228066 1.566000
H 1.033694 1.892592 1.748585
C 4.191948 2.882541 0.873772
H 4.631089 4.243341 -0.737322
H 3.404253 1.592626 2.414091
C 5.634431 2.678832 1.270844
H 5.753035 2.682256 2.360161
H 6.009550 1.713645 0.906362
H 6.278114 3.460999 0.856845
H 0.036157 2.918715 -3.144694
C -4.732734 -0.666879 -1.784909
C -5.420302 0.321456 -1.038110
C -5.237505 -1.990518 -1.759981
C -6.561285 -0.003056 -0.313836
H -5.051559 1.342039 -1.028744
C -6.375913 -2.308853 -1.033206
H -4.710581 -2.764372 -2.311699
C -7.045059 -1.316572 -0.306579
H -7.079788 0.769009 0.248282
H -6.743937 -3.330749 -1.025136
H -7.935514 -1.565969 0.263557
H -0.664865 1.240274 -0.801392
89
-3063.41536108
C -1.438222 3.769203 0.097131
C -2.502572 2.661673 0.105790
C -2.820776 2.065061 -1.270860
C -3.906986 0.964916 -1.242007
C -0.045615 3.342833 -0.413017
N 0.475592 2.155021 0.308568
H -2.176466 1.860191 0.780430
H -3.420772 3.078701 0.538080
H -1.912675 1.623258 -1.702196
H -3.135683 2.864735 -1.957251
H 0.654126 4.187632 -0.372806
H -1.337761 4.180597 1.104322
H -1.762984 4.591227 -0.558049
H -3.958923 0.503680 -2.237200
S 1.343698 2.600746 1.747773
O 0.301391 3.171678 2.618099
O 2.553486 3.378853 1.455830
C 1.809203 1.004858 2.395504
C 0.871019 0.276293 3.130760
C 3.093206 0.510210 2.171117
C 1.220392 -0.983073 3.609259
H -0.107440 0.701042 3.328840
C 3.421803 -0.753349 2.658004
H 3.813654 1.103022 1.619078
C 2.490083 -1.527721 3.361471
H 0.496330 -1.558854 4.180674
H 4.416818 -1.150301 2.474115
C 2.821186 -2.930802 3.802216
H 2.343547 -3.178105 4.756457
H 2.457105 -3.644427 3.052602
H 3.900757 -3.076560 3.910613
H -0.121570 3.034082 -1.457948
C -5.287096 1.456080 -0.854810
C -6.118717 2.067179 -1.804509
C -5.765935 1.330894 0.457048
C -7.384430 2.539564 -1.457030
H -5.770286 2.168017 -2.830611
C -7.032493 1.801581 0.811234
H -5.135619 0.862333 1.209740
C -7.846798 2.408163 -0.145758
H -8.012572 3.006307 -2.211624
H -7.381504 1.693959 1.835158
H -8.833600 2.773126 0.126204
H -3.590026 0.174281 -0.549718
Cl 1.831896 1.264155 -0.950893
C 0.603237 -0.884393 -3.533879
C 2.049735 -1.198245 -3.956823
C 3.154367 -1.044225 -2.892885
C 3.553610 0.366236 -2.555102
C -0.078565 -1.941471 -2.654286
N 0.590733 -2.096014 -1.352588
H 2.304671 -0.561834 -4.815869
H 2.085173 -2.231144 -4.327724
H 4.059168 -1.540614 -3.282522
H 2.884495 -1.602830 -1.989868
H -1.140484 -1.676009 -2.530379
H 0.548194 0.094304 -3.045380
H -0.009133 -0.806654 -4.442319
H 3.215611 1.149498 -3.230445
S 0.201299 -3.413706 -0.386827
O 1.034361 -3.277750 0.811136
O 0.269536 -4.594808 -1.251136
C -1.513101 -3.219933 0.117465
C -2.518279 -3.887808 -0.582779
C -1.825492 -2.388069 1.196983
C -3.848049 -3.713721 -0.198596
H -2.254474 -4.551916 -1.398903
C -3.158383 -2.227268 1.566333
H -1.032399 -1.892652 1.748484
C -4.191256 -2.881009 0.874055
H -4.631331 -4.240761 -0.737696
H -3.402677 -1.592263 2.414885
C -5.633590 -2.677076 1.271554
H -6.009947 -1.713720 0.903498
H -6.276891 -3.461483 0.861190
H -5.751278 -2.676245 2.360958
H -0.035771 -2.917841 -3.145894
C 4.732026 0.669884 -1.785778
C 5.421074 -0.317922 -1.039645
C 5.235385 1.994058 -1.760755
C 6.562124 0.007607 -0.315932
H 5.053416 -1.338897 -1.030354
C 6.373859 2.313411 -1.034530

H 4.707312 2.767502 -2.311952
C 7.044488 1.321641 -0.308574
H 7.081783 -0.764061 0.245663
H 6.740785 3.335701 -1.026365
H 7.934998 1.571831 0.261128
H 0.665034 -1.239529 -0.802513
89
-3063.41520028
C -3.451105 1.510274 2.583429
C -4.331303 1.678341 1.342364
C -5.632923 0.870948 1.423268
C -6.473538 0.912976 0.123941
C -2.215497 2.422939 2.661635
N -1.183429 2.362368 1.605447
H -4.575287 2.741155 1.198784
H -3.762392 1.374707 0.459996
H -6.248434 1.255750 2.248860
H -5.405932 -0.176485 1.667476
H -1.676252 2.204202 3.590476
H -3.128900 0.465684 2.665921
H -4.037732 1.725546 3.489388
H -7.486910 0.558346 0.350808
S -1.411364 3.309936 0.200778
O -1.832658 4.626930 0.709049
O -2.186317 2.677900 -0.878706
C 0.285813 3.417328 -0.369851
C 1.245861 4.044512 0.426346
C 0.608247 2.938900 -1.638078
C 2.545248 4.175138 -0.055762
H 0.975745 4.423702 1.406093
C 1.914917 3.078401 -2.105654
H -0.160987 2.471387 -2.242643
C 2.902394 3.691726 -1.324534
H 3.294223 4.669950 0.558425
H 2.169342 2.709365 -3.096418
C 4.322530 3.808482 -1.824449
H 4.919550 2.942236 -1.509341
H 4.360905 3.850736 -2.917805
H 4.813604 4.705625 -1.432526
H -2.524455 3.474537 2.702171
C -5.908396 0.078172 -1.008327
C -6.340133 -1.244196 -1.193085
C -4.935723 0.583957 -1.883582
C -5.817672 -2.041431 -2.213123
H -7.105498 -1.649331 -0.533629
C -4.407750 -0.210748 -2.904144
H -4.578440 1.603073 -1.765106
C -4.845401 -1.526210 -3.073855
H -6.177921 -3.059145 -2.343223
H -3.658971 0.206550 -3.572722
H -4.442630 -2.140215 -3.875433
H -6.574416 1.957747 -0.198623
Cl -0.768135 0.467321 1.092972
C 1.185834 -3.304671 -1.810962
C -0.154565 -2.635392 -2.159776
C -0.532298 -1.417257 -1.272645
C -0.485766 -1.735697 0.197275
C 2.402987 -2.379689 -1.953922
N 3.670814 -2.959103 -1.477235
H -0.947098 -3.389148 -2.080282
H -0.146827 -2.301010 -3.206785
H -1.526623 -1.064558 -1.566028
H 0.153268 -0.587211 -1.472530
H 2.553600 -2.100590 -3.004354
H 1.154697 -3.683894 -0.784613
H 1.327684 -4.176594 -2.465297
H 0.508091 -1.792263 0.636050
S 4.020768 -2.906758 0.177126
O 4.890799 -4.064059 0.411355
O 2.789159 -2.707917 0.958601
C 4.989229 -1.409448 0.349852
C 4.393584 -0.252410 0.849756
C 6.341048 -1.428265 -0.004423
C 5.165380 0.902664 0.985853
H 3.348688 -0.259165 1.140365
C 7.095329 -0.269153 0.143041
H 6.792389 -2.342248 -0.376042
C 6.522897 0.912791 0.641391
H 4.700878 1.805956 1.372963
H 8.148660 -0.280898 -0.126610
C 7.365860 2.151244 0.833090
H 7.949586 2.087883 1.760770
H 8.078897 2.282697 0.011905
H 6.748074 3.052372 0.896203
H 2.255145 -1.445723 -1.405012
C -1.490010 -2.518271 0.874975
C -2.810452 -2.665422 0.383965
C -1.150891 -3.168060 2.088296
C -3.739564 -3.433083 1.075670
H -3.111422 -2.165251 -0.530886
C -2.083636 -3.937513 2.770912
H -0.139089 -3.065041 2.471958
C -3.383252 -4.073099 2.268669
H -4.748173 -3.528213 0.683777
H -1.802036 -4.434911 3.694983
H -4.114364 -4.672365 2.804584
H 3.852312 -3.908601 -1.798267
89
-3063.41505078
C -0.304112 4.492238 -1.258879
C -1.617168 3.772573 -1.586701
C -2.813542 4.730065 -1.635334
C -4.159542 4.045118 -1.962696
C 0.935756 3.576614 -1.272740
N 0.811811 2.438501 -0.328110
H -1.522936 3.259774 -2.556752
H -1.796318 2.991391 -0.840354
H -2.629274 5.507017 -2.391218
H -2.901300 5.251583 -0.672171
H 1.850286 4.156668 -1.092753
H -0.382481 4.989581 -0.288517
H -0.111076 5.274282 -2.008437
H -4.916601 4.825430 -2.116617
S 1.291072 2.876959 1.285574
O 0.234456 3.803258 1.726648
O 2.697660 3.290919 1.361461
C 1.084927 1.331028 2.153456
C -0.198434 0.933956 2.532685
C 2.202633 0.552803 2.454473
C -0.357558 -0.281505 3.194615
H -1.050239 1.570864 2.318999
C 2.020425 -0.660175 3.113058
H 3.191100 0.892457 2.166659
C 0.741991 -1.106516 3.474689
H -1.352704 -0.596164 3.500483
H 2.885711 -1.278492 3.337583
C 0.550701 -2.462871 4.105329
H 1.401753 -2.738299 4.737120
H -0.356853 -2.501303 4.716847
H 0.459480 -3.223774 3.319789
H 1.023009 3.117595 -2.261608
C -4.644044 3.087224 -0.893075
C -5.238137 3.573726 0.280961
C -4.499440 1.701007 -1.034131
C -5.670586 2.705125 1.282459
H -5.365907 4.646856 0.408427
C -4.929169 0.825179 -0.034808
H -4.045814 1.302444 -1.938939
C -5.516827 1.325384 1.127666
H -6.131383 3.104927 2.182249
H -4.801905 -0.246326 -0.166801
H -5.857924 0.647577 1.906342
H -4.066149 3.511302 -2.917774
Cl 2.153277 1.026928 -0.979510
C 1.185920 -1.188818 -3.624857
C 2.415224 -2.077518 -3.352403
C 3.147882 -1.897965 -2.007260
C 3.878829 -0.594602 -1.828559
C -0.140953 -1.655416 -3.004275
N -0.156778 -1.733234 -1.538475

H 3.146328 -1.905939 -4.154545
H 2.116757 -3.129848 -3.429767
H 3.900961 -2.700133 -1.939362
H 2.480421 -2.077921 -1.157877
H -0.942354 -0.975020 -3.319984
H 1.384758 -0.154533 -3.323858
H 1.014110 -1.160073 -4.709959
H 3.968419 0.047582 -2.702371
S -0.143711 -3.178896 -0.716359
O 0.624150 -2.933521 0.513607
O 0.227295 -4.254615 -1.646522
C -1.850416 -3.473049 -0.242757
C -2.551112 -4.529577 -0.819232
C -2.452961 -2.649535 0.713300
C -3.874603 -4.758556 -0.436678
H -2.060496 -5.163510 -1.549608
C -3.771521 -2.893262 1.082607
H -1.894707 -1.834395 1.163207
C -4.504774 -3.949702 0.514828
H -4.422579 -5.583457 -0.885151
H -4.241499 -2.256414 1.828725
C -5.932627 -4.203784 0.936074
H -6.550929 -3.306817 0.811223
H -6.386021 -5.008784 0.350090
H -5.989051 -4.488323 1.994288
H -0.393090 -2.651138 -3.379214
C 4.874480 -0.403087 -0.807282
C 5.004297 -1.287402 0.292363
C 5.762616 0.697813 -0.885319
C 5.984785 -1.080268 1.255180
H 4.326388 -2.129586 0.387525
C 6.739173 0.899208 0.079823
H 5.664285 1.394732 -1.713501
C 6.856534 0.010541 1.155165
H 6.074318 -1.770812 2.089515
H 7.409715 1.750217 0.001623
H 7.619890 0.168916 1.911753
H 0.282794 -0.978098 -1.016979

$(C_6$-rad---N-Cl$)_{XAT,bi}$

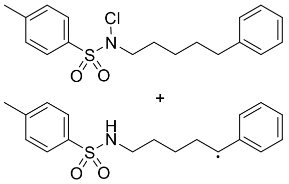

Name	E(B3LYP)	G(B3LYP)	E(RO-B2PLYP-D3)	G(RO-B2PLYP-D3)
Conformers				
Tosyl_NH_pentane_Ph_C6_radical_Tosyl_N_Cl_pentane_ separate_no_hydrogen_bond_0019_B2PLYP	-3065.126366	-3064.340853	-3064.075608	-3063.42943927
Tosyl_NH_pentane_Ph_C6_radical_Tosyl_N_Cl_pentane_ separate_no_hydrogen_bond_0037_B2PLYP	-3065.126366	-3064.340853	-3064.075601	-3063.42943332
Tosyl_NH_pentane_Ph_C6_radical_Tosyl_N_Cl_pentane_ separate_no_hydrogen_bond_0027_B2PLYP	-3065.120268	-3064.335196	-3064.074677	-3063.42931403
Tosyl_NH_pentane_Ph_C6_radical_Tosyl_N_Cl_pentane_ separate_no_hydrogen_bond_0031_B2PLYP	-3065.120269	-3064.335191	-3064.074636	-3063.42925921
Tosyl_NH_pentane_Ph_C6_radical_Tosyl_N_Cl_pentane_ separate_no_hydrogen_bond_0004_B2PLYP	-3065.120269	-3064.335192	-3064.074655	-3063.42925287
Tosyl_NH_pentane_Ph_C6_radical_Tosyl_N_Cl_pentane_ separate_no_hydrogen_bond_0024_B2PLYP	-3065.120269	-3064.335188	-3064.074643	-3063.42924700
Tosyl_NH_pentane_Ph_C6_radical_Tosyl_N_Cl_pentane_ separate_no_hydrogen_bond_0033_B2PLYP	-3065.120269	-3064.335190	-3064.074652	-3063.42923851
Tosyl_NH_pentane_Ph_C6_radical_Tosyl_N_Cl_pentane_ separate_no_hydrogen_bond_0035_B2PLYP	-3065.120269	-3064.335192	-3064.074659	-3063.42923608
Tosyl_NH_pentane_Ph_C6_radical_Tosyl_N_Cl_pentane_ separate_no_hydrogen_bond_0009_B2PLYP	-3065.120268	-3064.335192	-3064.074659	-3063.42923593
Tosyl_NH_pentane_Ph_C6_radical_Tosyl_N_Cl_pentane_ separate_no_hydrogen_bond_0022_B2PLYP	-3065.120269	-3064.335190	-3064.074649	-3063.42923439
Pre-reactive complexes for XAT				
Tosyl_NH_pentane_Ph_C6_H_Tosyl_N_pentane_TS_md_ 00200_IRC_reverse_opt_B2PLYP_ric	-3065.119189	-3064.333838	-3064.063372	-3063.42236127
Tosyl_NH_pentane_Ph_C6_H_Tosyl_N_pentane_TS_md_ 00020_IRC_reverse_opt_B2PLYP_ric	-3065.108112	-3064.322854	-3064.053020	-3063.41651019
Tosyl_NH_pentane_Ph_C6_H_Tosyl_N_pentane_TS_md_ 00020_IRC_forward_opt_B2PLYP_ric	-3065.108192	-3064.322945	-3064.052321	-3063.41276493
Tosyl_NH_pentane_Ph_C6_H_Tosyl_N_pentane_TS_md_ 00900_IRC_reverse_opt_B2PLYP_ric	-3065.115006	-3064.330046	-3064.066310	-3063.42393203
Tosyl_NH_pentane_Ph_C6_H_Tosyl_N_pentane_TS_md_ 00850_IRC_forward_opt_B2PLYP_ric	-3065.117494	-3064.332557	-3064.070715	-3063.42865015
Tosyl_NH_pentane_Ph_C6_H_Tosyl_N_pentane_TS_md_ 00750_IRC_reverse_opt_B2PLYP_ric	-3065.115006	-3064.330046	-3064.066309	-3063.42391305
Tosyl_NH_pentane_Ph_C6_H_Tosyl_N_pentane_TS_md_ 00550_IRC_reverse_opt_B2PLYP_ric	-3065.110777	-3064.325637	-3064.057182	-3063.41909248

89
-3063.42943927
C -3.804177 3.403348 -1.168126
C -3.972577 2.069445 -1.913977
C -4.524551 0.913996 -1.070003
C -4.655825 -0.390939 -1.882629
C -2.840118 3.374142 0.026534
N -1.558067 2.714350 -0.320737
H -3.005448 1.770204 -2.335395
H -4.640808 2.244378 -2.768688
H -5.503632 1.187249 -0.649572
H -3.863262 0.721367 -0.217333
H -2.674931 4.383875 0.424336
H -3.471269 4.171436 -1.875481
H -4.772336 3.745523 -0.776358
H -5.368692 -0.230206 -2.703733
S -0.476972 2.422308 1.044387
O -0.231194 3.646220 1.813201
O 0.654267 1.688342 0.455144
C -1.474682 1.310384 2.018448
C -1.755290 0.026791 1.539715
C -1.928514 1.744826 3.266242
C -2.524986 -0.820997 2.332057
H -1.398258 -0.303449 0.568719
C -2.688268 0.872551 4.042862
H -1.687013 2.743528 3.613527
C -3.001694 -0.416601 3.588588
H -2.774063 -1.808703 1.955095

H -3.047278 1.201379 5.014758
C -3.821620 -1.361766 4.432449
H -4.403700 -0.825437 5.188272
H -4.511937 -1.944939 3.813966
H -3.174996 -2.075827 4.959285
H -3.259830 2.757622 0.827034
C -5.093568 -1.574591 -1.044718
C -4.169197 -2.553417 -0.651838
C -6.424016 -1.707620 -0.621918
C -4.564803 -3.631031 0.145097
H -3.134679 -2.468228 -0.977040
C -6.821649 -2.780039 0.177325
H -7.158364 -0.964533 -0.927503
C -5.891319 -3.746758 0.565856
H -3.835856 -4.387253 0.427308
H -7.859518 -2.865650 0.490119
H -6.200800 -4.588428 1.180378
H -3.686120 -0.614286 -2.342751
C 3.247672 0.848350 -2.825918
C 3.434106 1.929436 -1.754209
C 4.913607 2.197210 -1.373575
C 5.649058 1.032485 -0.779601
C 1.801028 0.721498 -3.328887
N 0.804795 0.288978 -2.341371
H 2.874790 1.664524 -0.850269
H 3.004094 2.874274 -2.117297
H 4.931622 3.046769 -0.677191
H 3.873521 1.090149 -3.698189
H 3.596821 -0.121354 -2.450366
H 6.254650 0.425636 -1.450022
S 0.364781 -1.296892 -2.161311
O 0.308182 -1.895302 -3.497832
O -0.825853 -1.272958 -1.292812
C 1.655135 -2.158575 -1.250674
C 2.029240 -1.712677 0.021757
C 2.256385 -3.279146 -1.820750
C 3.019243 -2.399075 0.716929
H 1.565852 -0.833564 0.460217
C 3.241386 -3.960947 -1.104034
H 1.952804 -3.604774 -2.809682
C 3.636425 -3.536220 0.169434
H 3.327777 -2.040237 1.695366
H 3.711391 -4.836043 -1.546484
C 4.694488 -4.279845 0.948667
H 5.421506 -3.585534 1.384056
H 4.250314 -4.845556 1.778144
H 5.234130 -4.991271 0.315682
H 1.740874 0.015575 -4.161405
C 5.627476 0.646321 0.583170
C 4.832618 1.304732 1.567374
C 6.435474 -0.442670 1.027515
C 4.851277 0.896599 2.894187
H 4.192914 2.131381 1.274891
C 6.453924 -0.835577 2.356874
H 7.056154 -0.960153 0.299536
C 5.659547 -0.172169 3.304478
H 4.231618 1.416075 3.620797
H 7.093936 -1.658399 2.666802
H 5.675531 -0.480123 4.346189
H 0.697305 0.836799 -1.487769
Cl -0.660136 3.696362 -1.507060
H 1.457209 1.691242 -3.711379
H 5.447619 2.527720 -2.274803
89
-3063.42943332
C -3.806335 -3.401997 1.168087
C -3.973799 -2.068003 1.913988
C -4.525418 -0.912245 1.070192
C -4.655465 0.392799 1.882823
C -2.842486 -3.373395 -0.026765
N -1.559898 -2.714498 0.320261
H -3.006371 -1.769293 2.335091
H -4.641850 -2.242578 2.768914
H -5.504841 -1.184900 0.650170
H -3.864356 -0.720087 0.217242
H -2.678085 -4.383231 -0.424631
H -3.473749 -4.170288 1.875373
H -4.774776 -3.743586 0.776504
H -5.368124 0.232614 2.704210
S -0.478933 -2.423078 -1.045130
O -0.234000 -3.647158 -1.813930
O 0.652862 -1.689695 -0.456206
C -1.476226 -1.310649 -2.019052
C -1.756371 -0.027022 -1.540181
C -1.930308 -1.744841 -3.266857
C -2.525851 0.821089 -2.332409
H -1.399223 0.302985 -0.569146
C -2.689804 -0.872252 -4.043358
H -1.689180 -2.743609 -3.614209
C -3.002765 0.416977 -3.588942
H -2.774644 1.808820 -1.955331
H -3.049008 -1.200888 -5.015250
C -3.822350 1.362498 -4.432736
H -4.510105 1.948261 -3.813872
H -3.175220 2.074172 -4.962193
H -4.407137 0.826148 -5.186459
H -3.261938 -2.756560 -0.827157
C -5.092587 1.576796 1.045086
C -4.167524 2.554851 0.651852
C -6.423084 1.710951 0.622820
C -4.562546 3.632808 -0.144899
H -3.132942 2.468755 0.976671
C -6.820127 2.783726 -0.176252
H -7.157941 0.968483 0.928683
C -5.889140 3.749658 -0.565127
H -3.833093 4.388441 -0.427383
H -7.858046 2.870215 -0.488635
H -6.198144 4.591604 -1.179511
H -3.685416 0.615399 2.342538
C 3.247374 -0.851165 2.824617
C 3.434053 -1.930901 1.751581
C 4.913696 -2.198547 1.371409
C 5.649779 -1.033195 0.779449
C 1.800650 -0.724853 3.327460
N 0.804624 -0.291367 2.340170
H 2.875280 -1.664654 0.847685
H 3.003602 -2.876103 2.113199
H 4.931933 -3.047166 0.673891
H 3.873026 -1.094093 3.696716
H 3.596653 0.119001 2.450374
H 6.254808 -0.427173 1.451123
S 0.364758 1.294698 2.161373
O 0.308176 1.892055 3.498364
O -0.825848 1.271545 1.292815
C 1.655178 2.157021 1.251444
C 2.028639 1.712664 -0.021733
C 2.257138 3.276532 1.822815
C 3.018723 2.399528 -0.716302
H 1.564621 0.834398 -0.461228
C 3.242248 3.958830 1.106683
H 1.953969 3.601034 2.812242
C 3.636682 3.535620 -0.167454
H 3.326657 2.041918 -1.695385
H 3.712770 4.833147 1.550114
C 4.695225 4.279386 -0.945894
H 5.425886 3.585465 -1.375804
H 4.252380 4.840312 -1.779300
H 5.230492 4.994901 -0.313824
H 1.740364 -0.019761 4.160673
C 5.629553 -0.645523 -0.582919
C 4.835579 -1.302765 -1.568613
C 6.438182 0.443809 -1.025296
C 4.855724 -0.893300 -2.894995
H 4.195411 -2.129586 -1.277623
C 6.458097 0.838062 -2.354236
H 7.058212 0.960416 -0.296141
C 5.664632 0.175722 -3.303353
H 4.236760 -1.411928 -3.622802
H 7.098556 1.661099 -2.662666
H 5.681847 0.484665 -4.344754

H 0.697074 -0.838566 1.486163
Cl -0.662327 -3.697278 1.506202
H 1.456760 -1.694975 3.708930
H 5.447096 -2.530386 2.272510
89
-3063.42931403
C -1.459925 4.015848 -2.161310
C -1.887851 2.665848 -2.757279
C -3.074630 1.990408 -2.057440
C -3.550883 0.726465 -2.803384
C -1.036146 3.992970 -0.688666
N -0.007822 2.958482 -0.413246
H -1.029991 1.981635 -2.751971
H -2.140571 2.832607 -3.813706
H -3.913917 2.696064 -1.973499
H -2.800391 1.710294 -1.033039
H -0.693512 4.984196 -0.366893
H -0.642304 4.431311 -2.760832
H -2.288265 4.734673 -2.231707
H -3.893953 1.015203 -3.806428
S 0.323703 2.772539 1.313611
O 0.363969 4.072017 1.989980
O 1.463512 1.852418 1.389002
C -1.152490 1.900032 1.811271
C -1.301242 0.550188 1.475663
C -2.114259 2.570669 2.567041
C -2.443131 -0.123710 1.894664
H -0.533007 0.034948 0.909557
C -3.253141 1.876195 2.974638
H -1.963373 3.610683 2.836284
C -3.439422 0.528468 2.641568
H -2.561164 -1.173566 1.641616
H -4.007282 2.391507 3.563563
C -4.682545 -0.215961 3.061246
H -5.316999 0.393493 3.711498
H -5.272833 -0.506307 2.183379
H -4.429208 -1.137211 3.599027
H -1.888766 3.711882 -0.061281
C -4.651018 -0.021832 -2.080319
C -4.364110 -1.161785 -1.317948
C -5.980373 0.421502 -2.135400
C -5.372209 -1.839992 -0.628991
H -3.341877 -1.530336 -1.267986
C -6.991302 -0.249090 -1.446496
H -6.226300 1.298760 -2.730642
C -6.690708 -1.383966 -0.689217
H -5.123464 -2.730091 -0.056228
H -8.015830 0.109228 -1.507373
H -7.479223 -1.913634 -0.160919
H -2.693025 0.057353 -2.950716
C 3.788170 -1.491850 2.299952
C 2.406438 -1.182782 2.894302
C 1.403989 -2.347885 2.793556
C 0.911151 -2.586585 1.396582
C 4.709386 -0.262593 2.205510
N 4.367514 0.717790 1.171208
H 1.970777 -0.304157 2.405077
H 2.532607 -0.912725 3.951571
H 0.550838 -2.143003 3.457049
H 4.286267 -2.244933 2.927924
H 3.691634 -1.940799 1.303891
H 1.484006 -2.150207 0.580327
S 5.023183 0.671894 -0.353510
O 6.415566 0.236018 -0.202790
O 4.664260 1.945298 -0.981620
C 4.191095 -0.619422 -1.300492
C 2.927814 -0.374847 -1.845105
C 4.820438 -1.850361 -1.491448
C 2.294030 -1.380385 -2.573265
H 2.460196 0.596424 -1.719067
C 4.170835 -2.846148 -2.220850
H 5.815052 -2.009693 -1.088921
C 2.898964 -2.632189 -2.768060
H 1.319092 -1.183346 -3.014259
H 4.666005 -3.801785 -2.376170
C 2.190356 -3.723577 -3.535258
H 2.901633 -4.421541 -3.988401
H 1.535236 -4.308454 -2.875465
H 1.564058 -3.310951 -4.333533
H 5.740031 -0.569030 2.009819
C -0.236119 -3.338659 1.042681
C -1.061711 -3.996540 2.002105
C -0.614080 -3.461548 -0.328673
C -2.180876 -4.719351 1.610022
H -0.804234 -3.939031 3.055332
C -1.733554 -4.186147 -0.708622
H 0.000496 -2.971441 -1.080247
C -2.529834 -4.822251 0.256545
H -2.787852 -5.216103 2.363034
H -1.992899 -4.265700 -1.761413
H -3.401214 -5.397103 -0.043920
H 3.471662 1.200755 1.215474
Cl 1.521882 3.357397 -1.228229
H 4.717340 0.273486 3.163471
H 1.869401 -3.264741 3.197944
89
-3063.42925921
C -1.468569 4.020249 -2.155851
C -1.893452 2.670804 -2.755157
C -3.080505 1.991924 -2.059177
C -3.546518 0.723737 -2.804435
C -1.045875 3.994513 -0.682921
N -0.018353 2.958950 -0.408610
H -1.034486 1.987999 -2.749195
H -2.144194 2.838915 -3.811841
H -3.923343 2.693962 -1.980900
H -2.809846 1.715317 -1.032864
H -0.703030 4.984988 -0.359077
H -0.651045 4.438297 -2.753710
H -2.297944 4.737962 -2.225358
H -3.884683 1.007913 -3.810452
S 0.312136 2.770208 1.318017
O 0.349513 4.068456 1.996949
O 1.453515 1.851951 1.392685
C -1.162836 1.894020 1.812870
C -1.307148 0.543668 1.477464
C -2.128113 2.562302 2.566295
C -2.448075 -0.133248 1.894267
H -0.536128 0.030289 0.913462
C -3.266057 1.864914 2.971478
H -1.980518 3.602716 2.835828
C -3.447891 0.516532 2.638500
H -2.562407 -1.183686 1.641886
H -4.022892 2.378347 3.558591
C -4.690276 -0.230858 3.055086
H -5.323656 0.373973 3.710691
H -5.282090 -0.514784 2.176100
H -4.436125 -1.156019 3.585641
H -1.899053 3.712673 -0.056620
C -4.647276 -0.028019 -2.086027
C -5.977649 0.411694 -2.146252
C -4.360316 -1.167491 -1.322995
C -6.989461 -0.261874 -1.461611
H -6.223612 1.288506 -2.742135
C -5.369320 -1.848658 -0.638250
H -3.337338 -1.533462 -1.269296
C -6.688780 -1.396186 -0.703483
H -8.014710 0.093687 -1.526421
H -5.120415 -2.738225 -0.064733
H -7.477930 -1.928136 -0.178433
H -2.683853 0.059263 -2.944174
C 3.797417 -1.477640 2.299837
C 2.415038 -1.175373 2.896263
C 1.418042 -2.345334 2.796647
C 0.923542 -2.584869 1.400418
C 4.711349 -0.243251 2.201178
N 4.362739 0.732113 1.164347
H 1.974365 -0.298796 2.407891
H 2.541455 -0.904929 3.953400
H 0.565402 -2.145314 3.462173

H 4.300985 -2.226700 2.928250
H 3.701612 -1.929028 1.304810
H 1.495886 -2.149328 0.583352
S 5.017936 0.686140 -0.360549
O 6.413341 0.260274 -0.209282
O 4.650034 1.955166 -0.992363
C 4.194412 -0.613795 -1.303229
C 2.930605 -0.378805 -1.851105
C 4.830693 -1.842189 -1.487021
C 2.303454 -1.391475 -2.575001
H 2.457629 0.590611 -1.730656
C 4.187499 -2.845343 -2.212131
H 5.825581 -1.994080 -1.082300
C 2.915364 -2.641149 -2.762205
H 1.328196 -1.202253 -3.018696
H 4.687980 -3.799121 -2.361641
C 2.213347 -3.740160 -3.524588
H 1.604698 -3.336553 -4.341001
H 2.927957 -4.450083 -3.953106
H 1.541874 -4.309621 -2.867783
H 5.743624 -0.544119 2.005435
C -0.224401 -3.336489 1.047807
C -1.049491 -3.993940 2.007984
C -0.603702 -3.459226 -0.323216
C -2.169457 -4.716060 1.616934
H -0.791005 -3.936664 3.060973
C -1.723980 -4.183120 -0.702138
H 0.010437 -2.969424 -1.075358
C -2.519777 -4.818704 0.263775
H -2.776011 -5.212480 2.370514
H -1.984365 -4.262503 -1.754687
H -3.391785 -5.393016 -0.035888
H 3.464136 1.210006 1.208170
Cl 1.511799 3.356775 -1.223005
H 4.717003 0.295598 3.157591
H 1.888971 -3.260275 3.199022
89
-3063.42925287
C -1.462346 4.014663 -2.162961
C -1.888179 2.663762 -2.758323
C -3.076418 1.988421 -2.060907
C -3.544543 0.719172 -2.803006
C -1.040101 3.993016 -0.689844
N -0.012445 2.958366 -0.412524
H -1.029932 1.980076 -2.749729
H -2.138144 2.828858 -3.815665
H -3.918125 2.692030 -1.984580
H -2.806452 1.713988 -1.033825
H -0.697457 4.984400 -0.368580
H -0.644339 4.430315 -2.761829
H -2.291166 4.732783 -2.234866
H -3.881719 1.001357 -3.809917
S 0.317172 2.773573 1.314727
O 0.355190 4.073420 1.990533
O 1.457858 1.854686 1.392052
C -1.158662 1.899653 1.811010
C -1.304228 0.548838 1.478003
C -2.123334 2.570150 2.563223
C -2.445758 -0.126292 1.896021
H -0.533748 0.033742 0.914826
C -3.261911 1.874514 2.969665
H -1.974836 3.610935 2.830817
C -3.444973 0.525704 2.639136
H -2.560923 -1.177077 1.645480
H -4.018293 2.389705 3.555817
C -4.687893 -0.219908 3.057293
H -4.434398 -1.142508 3.592629
H -5.322328 0.387879 3.709127
H -5.278330 -0.508060 2.178773
H -1.893419 3.712763 -0.063036
C -4.647144 -0.028462 -2.083081
C -5.976516 0.414081 -2.144570
C -4.362890 -1.166893 -1.317490
C -6.989976 -0.255727 -1.458678
H -6.220406 1.290121 -2.742439
C -5.373569 -1.844319 -0.631502
H -3.340754 -1.535026 -1.262734
C -6.691999 -1.389050 -0.698017
H -8.014412 0.101982 -1.524492
H -5.126788 -2.733240 -0.056068
H -7.482447 -1.918086 -0.171981
H -2.683321 0.052371 -2.940628
C 3.792258 -1.482142 2.301513
C 2.410097 -1.176217 2.896543
C 1.410550 -2.343966 2.797050
C 0.916853 -2.583910 1.400598
C 4.709513 -0.250169 2.203682
N 4.364308 0.726300 1.166771
H 1.971883 -0.298984 2.407108
H 2.536234 -0.905216 3.953571
H 0.557646 -2.141319 3.461490
H 4.293268 -2.232428 2.930509
H 3.696228 -1.933395 1.306446
H 1.489435 -2.148429 0.583667
S 5.020208 0.678637 -0.357768
O 6.414195 0.248386 -0.205815
O 4.656594 1.948960 -0.989444
C 4.193101 -0.618435 -1.301238
C 2.929666 -0.379652 -1.848343
C 4.826228 -1.848164 -1.486740
C 2.299742 -1.389796 -2.573286
H 2.459113 0.590761 -1.726553
C 4.180289 -2.848778 -2.212990
H 5.820838 -2.003080 -1.082486
C 2.908536 -2.640713 -2.762444
H 1.324812 -1.197383 -3.016300
H 4.678354 -3.803594 -2.363908
C 2.203622 -3.736747 -3.526454
H 2.916124 -4.450374 -3.952283
H 1.527210 -4.302490 -2.871515
H 1.599572 -3.330588 -4.345047
H 5.741121 -0.553774 2.008656
C -0.230770 -3.335946 1.047804
C -1.055937 -3.993267 2.007981
C -0.609606 -3.459326 -0.323281
C -2.175507 -4.715973 1.616859
H -0.797798 -3.935450 3.061028
C -1.729478 -4.183816 -0.702271
H 0.004551 -2.969534 -1.075408
C -2.525334 -4.819324 0.263641
H -2.782124 -5.212291 2.370448
H -1.989522 -4.263687 -1.754866
H -3.397062 -5.394046 -0.036056
H 3.467123 1.206874 1.210156
Cl 1.518107 3.355214 -1.226710
H 4.715873 0.288516 3.160185
H 1.878916 -3.259567 3.200891
89
-3063.42924700
C -1.473930 4.011866 -2.161435
C -1.901573 2.661779 -2.757371
C -3.087630 1.985523 -2.057107
C -3.561106 0.719664 -2.801637
C -1.048715 3.988628 -0.689224
N -0.019766 2.954385 -0.415120
H -1.043357 1.978021 -2.752506
H -2.154877 2.828474 -3.813670
H -3.928059 2.689960 -1.974493
H -2.813409 1.707124 -1.032230
H -0.706157 4.979850 -0.367369
H -0.657098 4.427958 -2.761603
H -2.302705 4.730290 -2.230813
H -3.903701 1.006305 -3.805453
S 0.312933 2.767845 1.311440
O 0.351293 4.066950 1.988633
O 1.454366 1.849617 1.385685
C -1.161416 1.892371 1.809374
C -1.306746 0.541818 1.475190
C -2.125074 2.561463 2.564151
C -2.447088 -0.134468 1.894638

H -0.536966 0.027790 0.910083
C -3.262400 1.864672 2.972059
H -1.976709 3.602050 2.832584
C -3.445243 0.516101 2.640423
H -2.562163 -1.185052 1.643181
H -4.017955 2.378738 3.560262
C -4.686901 -0.230687 3.060224
H -5.279689 -0.516876 2.182643
H -4.431818 -1.154484 3.592719
H -5.319662 0.375497 3.715174
H -1.900627 3.706987 -0.061126
C -4.660500 -0.029565 -2.078497
C -4.372946 -1.169838 -1.316876
C -5.990047 0.413390 -2.132805
C -5.380612 -1.848648 -0.627841
H -3.350664 -1.538360 -1.267705
C -7.000494 -0.257828 -1.443856
H -6.236464 1.290886 -2.727491
C -6.699251 -1.392993 -0.687241
H -5.131377 -2.738908 -0.055543
H -8.025155 0.100207 -1.504134
H -7.487380 -1.923137 -0.158846
H -2.701514 0.052415 -2.947037
C 3.796281 -1.478857 2.302336
C 2.412592 -1.176971 2.895893
C 1.415626 -2.346656 2.792922
C 0.924104 -2.584816 1.395405
C 4.710924 -0.244655 2.207988
N 4.365201 0.732708 1.172070
H 1.973172 -0.299852 2.407347
H 2.536663 -0.907595 3.953586
H 0.561558 -2.147105 3.456755
H 4.298011 -2.229115 2.930788
H 3.702732 -1.928587 1.306350
H 1.498419 -2.148869 0.579943
S 5.023805 0.688670 -0.351471
O 6.418852 0.262556 -0.197606
O 4.657318 1.958536 -0.982371
C 4.202274 -0.609948 -1.297678
C 2.938906 -0.374552 -1.846369
C 4.839600 -1.837445 -1.483660
C 2.313326 -1.385857 -2.573455
H 2.464989 0.594150 -1.724064
C 4.197923 -2.839284 -2.211993
H 5.834097 -1.989666 -1.078106
C 2.926329 -2.634609 -2.763099
H 1.338530 -1.196006 -3.017803
H 4.699246 -3.792345 -2.363235
C 2.225975 -3.732062 -3.529256
H 1.552945 -4.302735 -2.875110
H 1.619223 -3.326797 -4.346295
H 2.941507 -4.441201 -3.957535
H 5.743546 -0.545580 2.014186
C -0.223552 -3.335437 1.039732
C -1.050997 -3.993274 1.997606
C -0.600179 -3.456610 -0.332170
C -2.170768 -4.714123 1.603622
H -0.794491 -3.937335 3.051151
C -1.720261 -4.179239 -0.714017
H 0.015815 -2.966515 -1.082599
C -2.518516 -4.815104 0.249688
H -2.779157 -5.210858 2.355510
H -1.978589 -4.257459 -1.767163
H -3.390501 -5.388249 -0.052250
H 3.466108 1.209857 1.213990
Cl 1.509158 3.354003 -1.231164
H 4.714481 0.292474 3.165385
H 1.885518 -3.262050 3.195479
89
-3063.42923851
C -1.467577 4.011983 -2.164326
C -1.894674 2.661271 -2.759241
C -3.081440 1.985836 -2.059386
C -3.554313 0.719172 -2.802881
C -1.043797 3.990302 -0.691670
N -0.015400 2.956075 -0.415468
H -1.036482 1.977491 -2.752817
H -2.146949 2.826845 -3.815962
H -3.921905 2.690424 -1.978395
H -2.808243 1.708603 -1.033918
H -0.701249 4.981792 -0.370629
H -0.650131 4.427399 -2.764124
H -2.296264 4.730353 -2.235293
H -3.895797 1.004603 -3.807417
S 0.315917 2.771370 1.311535
O 0.354508 4.071253 1.987227
O 1.456745 1.852558 1.387735
C -1.159318 1.897354 1.809422
C -1.305151 0.546492 1.476716
C -2.123155 2.567850 2.562711
C -2.446137 -0.128686 1.896159
H -0.535280 0.031394 0.912706
C -3.261175 1.872157 2.970594
H -1.974423 3.608672 2.830027
C -3.444503 0.523294 2.640431
H -2.561529 -1.179512 1.645884
H -4.016891 2.387335 3.557617
C -4.686847 -0.222373 3.060198
H -5.320221 0.385216 3.713249
H -5.278667 -0.510108 2.182471
H -4.432622 -1.145212 3.594771
H -1.896381 3.709615 -0.064058
C -4.654586 -0.029024 -2.079967
C -5.984083 0.413779 -2.136411
C -4.367833 -1.168241 -1.316455
C -6.995276 -0.256572 -1.447692
H -6.229893 1.290444 -2.732574
C -5.376231 -1.846195 -0.627660
H -3.345556 -1.536554 -1.265544
C -6.694834 -1.390692 -0.689202
H -8.019885 0.101333 -1.509632
H -5.127592 -2.735698 -0.053928
H -7.483543 -1.920177 -0.161008
H -2.694773 0.051457 -2.946496
C 3.792422 -1.481687 2.302750
C 2.409353 -1.176876 2.896239
C 1.410460 -2.345015 2.794632
C 0.918639 -2.584216 1.397391
C 4.709316 -0.249249 2.207378
N 4.365023 0.728214 1.171101
H 1.971340 -0.299507 2.406867
H 2.534025 -0.906609 3.953630
H 0.556633 -2.143200 3.458134
H 4.292886 -2.232392 2.931682
H 3.697904 -1.931990 1.307110
H 1.492324 -2.148306 0.581468
S 5.022916 0.682413 -0.352665
O 6.417088 0.253253 -0.199308
O 4.658962 1.952957 -0.983682
C 4.198095 -0.614578 -1.298224
C 2.934763 -0.376652 -1.845890
C 4.832828 -1.843342 -1.484736
C 2.306551 -1.386698 -2.572471
H 2.462909 0.593012 -1.723232
C 4.188594 -2.843857 -2.212608
H 5.827336 -1.997538 -1.079955
C 2.916947 -2.636643 -2.762672
H 1.331694 -1.194935 -3.015908
H 4.687911 -3.797898 -2.364321
C 2.213819 -3.732677 -3.528307
H 2.927702 -4.441714 -3.959495
H 1.542231 -4.303781 -2.873042
H 1.605062 -3.325876 -4.343063
H 5.741288 -0.552239 2.013317
C -0.228553 -3.335996 1.042641
C -1.055129 -3.993712 2.001339
C -0.605496 -3.458657 -0.329034
C -2.174277 -4.716036 1.608303
H -0.798413 -3.936515 3.054769
C -1.724955 -4.182771 -0.709934

H 0.009791 -2.968607 -1.080065
C -2.522262 -4.818628 0.254551
H -2.781986 -5.212684 2.360796
H -1.983523 -4.262118 -1.762933
H -3.393673 -5.393048 -0.046638
H 3.467179 1.207657 1.213433
Cl 1.514259 3.354216 -1.230803
H 4.714281 0.288383 3.164487
H 1.878730 -3.260686 3.198429
89
-3063.42923608
C 1.467498 4.017516 2.157374
C 1.895068 2.668299 2.755342
C 3.081062 1.990958 2.056030
C 3.555185 0.726652 2.802713
C 1.042974 3.992253 0.684983
N 0.014720 2.957130 0.411722
H 1.036814 1.984574 2.751540
H 2.148396 2.836593 3.811383
H 3.921310 2.695434 1.971826
H 2.806582 1.710623 1.031746
H 0.700073 4.982917 0.361766
H 0.650332 4.434298 2.756604
H 2.296155 4.736135 2.226156
H 3.898316 1.015259 3.805781
S -0.317580 2.768445 -1.314752
O -0.357123 4.066822 -1.993290
O -1.458049 1.848967 -1.388112
C 1.157677 1.893906 -1.811619
C 1.303967 0.543738 -1.476350
C 2.121068 2.563165 -2.566592
C 2.444953 -0.132002 -1.894902
H 0.534418 0.029567 -0.911064
C 3.259083 1.866930 -2.973561
H 1.971993 3.603427 -2.835898
C 3.442865 0.518742 -2.640842
H 2.560566 -1.182304 -1.642562
H 4.014461 2.381132 -3.561874
C 4.685155 -0.227478 -3.059800
H 4.430841 -1.150663 -3.593749
H 5.318746 0.379494 -3.713215
H 5.276773 -0.514666 -2.181768
H 1.895327 3.710256 0.057639
C 4.654396 -0.023682 2.080394
C 4.366411 -1.164434 1.319654
C 5.984129 0.418681 2.134557
C 5.373847 -1.844339 0.631383
H 3.343910 -1.532355 1.270412
C 6.994385 -0.253624 1.446346
H 6.230885 1.296559 2.728542
C 6.692722 -1.389290 0.690666
H 5.124275 -2.734972 0.059810
H 8.019198 0.104001 1.506511
H 7.480676 -1.920312 0.162887
H 2.695964 0.059295 2.949977
C -3.793405 -1.486056 -2.299508
C -2.410870 -1.181698 -2.894480
C -1.411667 -2.349504 -2.792075
C -0.918597 -2.586631 -1.394915
C -4.710752 -0.253862 -2.205364
N -4.366100 0.725488 -1.170961
H -1.972653 -0.303590 -2.406606
H -2.536411 -0.912881 -3.952140
H -0.558425 -2.148489 -3.456573
H -4.294039 -2.237921 -2.926920
H -3.697981 -1.934806 -1.303254
H -1.491527 -2.149519 -0.579102
S -5.022642 0.681626 0.353504
O -6.416674 0.251349 0.202007
O -4.658964 1.953386 0.982225
C -4.196090 -0.613361 1.300294
C -2.932113 -0.373935 1.845905
C -4.829974 -1.842087 1.489668
C -2.302426 -1.382461 2.573263
H -2.460858 0.595732 1.720973
C -4.184259 -2.841114 2.218338
H -5.824920 -1.997470 1.086416
C -2.911998 -2.632424 2.766335
H -1.326976 -1.189469 3.014908
H -4.682894 -3.795159 2.372229
C -2.207258 -3.726768 3.532908
H -1.603424 -3.318744 4.350724
H -2.919846 -4.439449 3.960169
H -1.530675 -4.293921 2.879361
H -5.742420 -0.557020 -2.009965
C 0.229023 -3.337759 -1.040158
C 1.054534 -3.997080 -1.998684
C 0.607569 -3.458044 0.331277
C 2.174140 -4.718706 -1.605710
H 0.796616 -3.941659 -3.051913
C 1.727513 -4.181464 0.712127
H -0.006821 -2.966683 1.082189
C 2.523715 -4.818959 -0.252184
H 2.781009 -5.216605 -2.358055
H 1.987310 -4.258956 1.764963
H 3.395449 -5.392891 0.049001
H -3.468254 1.204819 -1.214545
Cl -1.514640 3.356817 1.226962
H -4.716767 0.282207 -3.163346
H -1.880117 -3.265825 -3.194183
89
-3063.42923593
C -1.457751 4.016856 -2.164291
C -1.882531 2.665587 -2.759559
C -3.071144 1.989917 -2.063112
C -3.536652 0.719265 -2.804484
C -1.036754 3.995866 -0.690796
N -0.009705 2.961023 -0.412023
H -1.023999 1.982272 -2.749861
H -2.131508 2.830169 -3.817214
H -3.913683 2.692748 -1.988873
H -2.802438 1.716923 -1.035318
H -0.694057 4.987324 -0.369807
H -0.639350 4.432546 -2.762591
H -2.286737 4.734683 -2.237183
H -3.872525 0.999891 -3.812267
S 0.318701 2.777271 1.315506
O 0.356595 4.077547 1.990494
O 1.459101 1.858147 1.394243
C -1.157694 1.904057 1.811375
C -1.303186 0.552982 1.479434
C -2.122955 2.575367 2.562117
C -2.445192 -0.121606 1.897011
H -0.532292 0.037267 0.917385
C -3.262043 1.880283 2.968082
H -1.974521 3.616355 2.828959
C -3.445020 0.531206 2.638573
H -2.560220 -1.172602 1.647318
H -4.018905 2.396116 3.553052
C -4.688451 -0.213856 3.056202
H -5.277548 -0.503639 2.177320
H -4.435607 -1.135486 3.593525
H -5.323924 0.394966 3.706057
H -1.890688 3.716248 -0.064544
C -4.639509 -0.028865 -2.085501
C -4.355290 -1.166332 -1.318464
C -5.969218 0.412332 -2.149349
C -5.366325 -1.844102 -0.633337
H -3.332903 -1.533494 -1.261844
C -6.983040 -0.257812 -1.464319
H -6.213079 1.287586 -2.748380
C -6.685090 -1.390147 -0.702182
H -5.119531 -2.732287 -0.056776
H -8.007717 0.098866 -1.531963
H -7.475794 -1.919463 -0.176811
H -2.674227 0.053582 -2.939930
C 3.789459 -1.482395 2.302119
C 2.407931 -1.174302 2.897500
C 1.407064 -2.341017 2.799608
C 0.912756 -2.582061 1.403550

C 4.707868 -0.251459 2.202081
N 4.363005 0.723863 1.164004
H 1.970507 -0.297055 2.407380
H 2.534841 -0.902337 3.954184
H 0.554497 -2.136621 3.463992
H 4.290086 -2.232342 2.931822
H 3.692372 -1.934887 1.307714
H 1.484132 -2.146031 0.586064
S 5.017914 0.673673 -0.360816
O 6.411332 0.241433 -0.209277
O 4.655870 1.943893 -0.993621
C 4.188271 -0.623091 -1.302479
C 2.925292 -0.382603 -1.849982
C 4.818977 -1.854282 -1.486263
C 2.293430 -1.392477 -2.573563
H 2.456700 0.588936 -1.729571
C 4.171074 -2.854656 -2.211155
H 5.813281 -2.010621 -1.081807
C 2.899806 -2.644871 -2.760990
H 1.318964 -1.198681 -3.017004
H 4.667264 -3.810658 -2.360693
C 2.192834 -3.740432 -3.523788
H 2.903585 -4.460065 -3.942323
H 1.509486 -4.299175 -2.870055
H 1.595659 -3.334681 -4.347635
H 5.739065 -0.556353 2.006902
C -0.234027 -3.335912 1.051893
C -1.057441 -3.994062 2.012995
C -0.613699 -3.460480 -0.318848
C -2.176076 -4.718837 1.623030
H -0.798652 -3.935239 3.065830
C -1.732624 -4.187045 -0.696683
H -0.000918 -2.969989 -1.071641
C -2.526668 -4.823478 0.270111
H -2.781353 -5.215757 2.377299
H -1.993357 -4.267777 -1.749040
H -3.397574 -5.399914 -0.028702
H 3.466833 1.206276 1.207741
Cl 1.521514 3.356343 -1.225637
H 4.715283 0.288570 3.157816
H 1.874397 -3.256697 3.204441
89
-3063.42923439
C -1.466048 4.012090 -2.165318
C -1.892458 2.661031 -2.759923
C -3.079725 1.985722 -2.060813
C -3.550464 0.717663 -2.803292
C -1.043036 3.991047 -0.692426
N -0.014975 2.956764 -0.415209
H -1.034152 1.977407 -2.752371
H -2.143814 2.825997 -3.816957
H -3.920803 2.689815 -1.981974
H -2.807796 1.710035 -1.034588
H -0.700490 4.982633 -0.371681
H -0.648351 4.427432 -2.764823
H -2.294844 4.730259 -2.237031
H -3.890285 1.001370 -3.808883
S 0.315511 2.772845 1.312005
O 0.353654 4.073026 1.987153
O 1.456369 1.854163 1.389214
C -1.159916 1.898933 1.809513
C -1.305398 0.547883 1.477424
C -2.124303 2.569714 2.561844
C -2.446573 -0.127203 1.896500
H -0.535123 0.032572 0.914158
C -3.262516 1.874116 2.969349
H -1.975850 3.610689 2.828722
C -3.445494 0.525060 2.639770
H -2.561670 -1.178176 1.646711
H -4.018669 2.389522 3.555608
C -4.688038 -0.220516 3.059108
H -5.279121 -0.508988 2.181127
H -4.434072 -1.142924 3.594549
H -5.322024 0.387434 3.711226
H -1.895985 3.710800 -0.065111
C -4.651513 -0.030262 -2.081291
C -4.365533 -1.168878 -1.316605
C -5.981038 0.412202 -2.139831
C -5.374700 -1.846570 -0.628671
H -3.343252 -1.536947 -1.264111
C -6.992994 -0.257882 -1.451982
H -6.226253 1.288388 -2.736942
C -6.693313 -1.391396 -0.692280
H -5.126629 -2.735621 -0.053993
H -8.017599 0.099751 -1.515537
H -7.482608 -1.920667 -0.164748
H -2.690104 0.050451 -2.944269
C 3.792239 -1.480315 2.303154
C 2.409335 -1.175188 2.896872
C 1.410415 -2.343379 2.796176
C 0.918213 -2.583319 1.399196
C 4.708939 -0.247835 2.206441
N 4.364188 0.728648 1.169396
H 1.971183 -0.298085 2.407153
H 2.534302 -0.904333 3.954076
H 0.556771 -2.141215 3.459812
H 4.293015 -2.230451 2.932515
H 3.697427 -1.931442 1.307914
H 1.491562 -2.147670 0.582896
S 5.021708 0.681745 -0.354477
O 6.415950 0.252813 -0.201141
O 4.657508 1.951775 -0.986391
C 4.196787 -0.616043 -1.298857
C 2.933561 -0.378491 -1.846949
C 4.831336 -1.845109 -1.483984
C 2.305266 -1.389214 -2.572510
H 2.461883 0.591403 -1.725399
C 4.187014 -2.846310 -2.210848
H 5.825779 -1.999040 -1.078942
C 2.915470 -2.639484 -2.761275
H 1.330487 -1.197798 -3.016268
H 4.686182 -3.800601 -2.361463
C 2.212207 -3.736209 -3.525799
H 1.605609 -3.330368 -4.342651
H 2.925879 -4.447245 -3.954018
H 1.538507 -4.304834 -2.870553
H 5.740896 -0.550848 2.012340
C -0.228885 -3.335568 1.045134
C -1.055028 -3.993015 2.004379
C -0.606148 -3.459033 -0.326385
C -2.174070 -4.715871 1.611996
H -0.798077 -3.935190 3.057717
C -1.725485 -4.183672 -0.706630
H 0.008800 -2.969169 -1.077815
C -2.522360 -4.819272 0.258391
H -2.781454 -5.212278 2.364909
H -1.984320 -4.263619 -1.759517
H -3.393701 -5.394071 -0.042279
H 3.466483 1.208341 1.211781
Cl 1.515120 3.353962 -1.230153
H 4.714111 0.290648 3.163068
H 1.878787 -3.258842 3.200325

89
-3063.42865015
C -3.743918 2.726482 -2.387829
C -4.631729 1.673153 -1.713754
C -5.868725 2.282537 -1.043791
C -6.730277 1.261497 -0.270468
C -2.434946 2.159745 -2.948949
N -1.604808 1.554189 -1.855305
H -4.947100 0.922807 -2.454732
H -4.039708 1.134935 -0.964641
H -6.499495 2.769183 -1.800701
H -5.550027 3.078337 -0.356396
H -1.868651 2.921407 -3.497790
H -3.510821 3.531220 -1.684575
H -4.277656 3.188520 -3.231134
H -7.635086 1.772293 0.086518
S -0.428190 2.665434 -1.168893
O -1.236463 3.860355 -0.905949
O 0.786028 2.765566 -1.984358
C -0.036212 1.822274 0.352332
C -1.055686 1.477022 1.240693
C 1.305404 1.574162 0.655118
C -0.718227 0.844834 2.436619
H -2.093397 1.683704 1.001918
C 1.621822 0.947963 1.858397
H 2.085478 1.870490 -0.037502
C 0.617321 0.564670 2.762522
H -1.511382 0.560690 3.123308
H 2.663058 0.747559 2.093422
C 0.982224 -0.151628 4.038263
H 1.715994 0.422087 4.616573
H 0.105958 -0.319614 4.671844
H 1.436384 -1.121518 3.805217
H -2.650257 1.346141 -3.644738
C -6.026394 0.614528 0.907708
C -5.630337 1.380500 2.015172
C -5.758619 -0.760466 0.928830
C -4.986479 0.793171 3.104861
H -5.837872 2.448340 2.029333
C -5.118515 -1.355786 2.018782
H -6.064589 -1.373831 0.083991
C -4.727350 -0.580137 3.111116
H -4.698822 1.406103 3.955391
H -4.935632 -2.427568 2.018457
H -4.238102 -1.042257 3.964491
H -7.071770 0.479777 -0.961285
Cl -0.792888 0.063992 -2.394943
C 3.194150 -0.790937 -2.837468
C 4.729478 -0.687171 -2.817123
C 5.364832 -0.209578 -1.497867
C 5.017913 1.205939 -1.146765
C 2.587888 -1.956762 -2.043110
N 2.651637 -1.724984 -0.589950
H 5.033438 0.001111 -3.618295
H 5.153609 -1.665627 -3.080055
H 6.461089 -0.302032 -1.599667
H 5.083019 -0.891818 -0.688159
H 1.548723 -2.117862 -2.370212
H 2.738339 0.154412 -2.511976
H 2.873803 -0.929065 -3.878595
H 4.713733 1.861091 -1.962841
S 2.321097 -3.000295 0.453950
O 2.338682 -2.407762 1.796079
O 3.217002 -4.096349 0.079280
C 0.641134 -3.545912 0.116497
C 0.430015 -4.711433 -0.620623
C -0.438008 -2.781148 0.569327
C -0.876328 -5.110820 -0.903184
H 1.280214 -5.297656 -0.952364
C -1.734703 -3.191143 0.272918
H -0.267778 -1.880558 1.150600
C -1.975099 -4.361221 -0.464232
H -1.042847 -6.021050 -1.474222
H -2.572921 -2.590648 0.618488
C -3.387314 -4.811699 -0.753261
H -4.047568 -3.958719 -0.943933
H -3.427450 -5.474620 -1.623354
H -3.806887 -5.363974 0.098168
H 3.144201 -2.877673 -2.243134
C 5.140024 1.797136 0.135206
C 5.571779 1.073366 1.286765
C 4.830203 3.178726 0.317069
C 5.683103 1.695893 2.523051
H 5.820193 0.020856 1.194347
C 4.943461 3.788118 1.556835
H 4.498509 3.755359 -0.543452
C 5.371579 3.054206 2.672419
H 6.018511 1.120281 3.382294
H 4.699164 4.842115 1.662130
H 5.462280 3.534095 3.642777
H 2.162084 -0.886331 -0.278477
89
-3063.42393203
C 2.338733 -3.692657 -0.767803
C 3.151034 -2.417742 -0.492493
C 3.414877 -1.541344 -1.723606
C 4.238636 -0.268785 -1.419270
C 0.919512 -3.460859 -1.306775
N 0.127255 -2.553424 -0.411164
H 2.630857 -1.822363 0.268737
H 4.110071 -2.716890 -0.051964
H 2.462204 -1.220260 -2.165250
H 3.933349 -2.131178 -2.493484
H 0.395467 -4.410875 -1.469852
H 2.282001 -4.292562 0.143432
H 2.853263 -4.305920 -1.522383
H 4.252320 0.357551 -2.320773
S -0.772913 -3.446503 0.829415
O 0.283825 -4.215771 1.494232
O -1.938634 -4.121297 0.254797
C -1.308453 -2.123272 1.892512
C -0.427539 -1.642759 2.863868
C -2.593923 -1.595644 1.749117
C -0.843626 -0.600381 3.689223
H 0.554305 -2.089996 2.976629
C -2.983823 -0.548371 2.578807
H -3.271757 -1.994632 1.002646
C -2.117657 -0.027449 3.551892
H -0.169927 -0.225921 4.456065
H -3.975714 -0.122993 2.457544
C -2.539950 1.142676 4.403253
H -2.449912 2.069453 3.824131
H -3.584564 1.050120 4.719712
H -1.917150 1.236533 5.298523
H 0.967124 -2.950386 -2.269627
C 5.664651 -0.535301 -0.981589
C 6.663012 -0.799482 -1.930723
C 6.024848 -0.542574 0.373320
C 7.975802 -1.064429 -1.541006
H 6.407544 -0.790838 -2.988573
C 7.337934 -0.806571 0.769964
H 5.265540 -0.342339 1.126339
C 8.318609 -1.068919 -0.187013
H 8.733383 -1.262409 -2.295043
H 7.593136 -0.806193 1.826643
H 9.341573 -1.272061 0.118139
H 3.717040 0.311832 -0.647067
Cl -1.028956 -1.583077 -1.377788
C -1.407744 1.858508 -3.392065
C -2.841600 2.211137 -3.825169
C -3.969373 1.809133 -2.856755
C -4.102103 0.328012 -2.661647
C -0.819446 2.687213 -2.241922
N -1.445832 2.362100 -0.949829
H -3.028411 1.730938 -4.796069
H -2.900077 3.292900 -4.005128
H -4.916366 2.211149 -3.258897
H -3.819801 2.311574 -1.894126
H 0.270081 2.531678 -2.204143
H -1.335263 0.790404 -3.145376

H -0.741177 2.009804 -4.251620
H -3.647794 -0.321729 -3.409545
S -1.176298 3.374040 0.363656
O -1.870998 2.737047 1.486685
O -1.499065 4.730590 -0.083630
C 0.584912 3.339112 0.724679
C 1.409428 4.349020 0.228189
C 1.115278 2.294989 1.489445
C 2.777591 4.308143 0.499305
H 0.976708 5.166050 -0.339154
C 2.482546 2.271621 1.749806
H 0.462454 1.526842 1.892553
C 3.336848 3.273523 1.259136
H 3.419033 5.100090 0.120950
H 2.894169 1.468697 2.357260
C 4.817932 3.231283 1.550941
H 5.007612 3.120226 2.625171
H 5.299403 2.382413 1.049346
H 5.318119 4.144046 1.213798
H -0.996997 3.752001 -2.420087
C -4.866526 -0.299846 -1.646140
C -5.576155 0.433589 -0.650565
C -4.960177 -1.722255 -1.594145
C -6.327313 -0.217727 0.318890
H -5.531148 1.517995 -0.652222
C -5.709111 -2.362835 -0.618801
H -4.424325 -2.308794 -2.336343
C -6.402042 -1.617234 0.346876
H -6.866121 0.367527 1.060238
H -5.755851 -3.448463 -0.603918
H -6.992473 -2.120131 1.107521
H -1.367023 1.384398 -0.671603
89
-3063.42391305
C -2.334425 3.693815 -0.766628
C -3.148193 2.419327 -0.493667
C -3.414386 1.546346 -1.726706
C -4.238422 0.273428 -1.424591
C -0.915152 3.461271 -1.305145
N -0.124036 2.552843 -0.409553
H -2.628106 1.821422 0.265651
H -4.106433 2.718647 -0.051538
H -2.462593 1.225873 -2.170704
H -3.933660 2.138567 -2.494201
H -0.390352 4.411000 -1.467475
H -2.277458 4.292287 0.145535
H -2.847932 4.308800 -1.520502
H -4.254355 -0.350121 -2.327984
S 0.776632 3.444955 0.831501
O -0.279598 4.214944 1.496286
O 1.942990 4.118947 0.257225
C 1.310876 2.121015 1.894353
C 0.429177 1.640582 2.865041
C 2.596104 1.592706 1.751369
C 0.844224 0.597584 3.690126
H -0.552498 2.088278 2.977431
C 2.984962 0.544839 2.580796
H 3.274519 1.991603 1.005380
C 2.117984 0.023959 3.553171
H 0.169904 0.223139 4.456429
H 3.976663 0.118925 2.459841
C 2.539096 -1.146890 4.404117
H 1.915780 -1.240836 5.299019
H 2.448687 -2.073284 3.824438
H 3.583613 -1.055210 4.721151
H -0.962665 2.951360 -2.268305
C -5.663369 0.539569 -0.983240
C -6.663744 0.805947 -1.929620
C -6.020535 0.544379 0.372491
C -7.975570 1.070627 -1.536457
H -6.410643 0.799252 -2.988052
C -7.332628 0.808091 0.772577
H -5.259598 0.342450 1.123410
C -8.315350 1.072634 -0.181704
H -8.734756 1.270336 -2.288420
H -7.585470 0.805785 1.829821
H -9.337553 1.275560 0.126129
H -3.715638 -0.309910 -0.655246
Cl 1.031692 1.581727 -1.375998
C 1.403741 -1.857357 -3.392310
C 2.837367 -2.209012 -3.826963
C 3.965799 -1.807914 -2.858944
C 4.098782 -0.326982 -2.662545
C 0.816795 -2.687513 -2.242516
N 1.443604 -2.362680 -0.950528
H 3.023300 -1.727594 -4.797429
H 2.896000 -3.290539 -4.008285
H 4.912499 -2.209575 -3.262144
H 3.816907 -2.311261 -1.896683
H -0.272863 -2.533053 -2.204028
H 1.331062 -0.789557 -3.144376
H 0.736536 -2.008002 -4.251479
H 3.644119 0.323489 -3.409599
S 1.173607 -3.374735 0.362868
O 1.868133 -2.737985 1.486137
O 1.496242 -4.731296 -0.084467
C -0.587690 -3.339499 0.723422
C -1.412245 -4.349250 0.226693
C -1.118085 -2.295255 1.488019
C -2.780484 -4.308117 0.497412
H -0.979516 -5.166349 -0.340543
C -2.485425 -2.271621 1.747961
H -0.465215 -1.527227 1.891287
C -3.339767 -3.273396 1.257076
H -3.421967 -5.099932 0.118853
H -2.897089 -1.468605 2.355265
C -4.820888 -3.230991 1.548657
H -5.301736 -2.380541 1.049172
H -5.321548 -4.142602 1.209113
H -5.010703 -3.122594 2.623146
H 0.995229 -3.752007 -2.421568
C 4.864184 0.299826 -1.647105
C 5.574550 -0.434673 -0.652843
C 4.958218 1.722161 -1.593920
C 6.326752 0.215589 0.316509
H 5.529295 -1.519065 -0.655438
C 5.708207 2.361686 -0.618695
H 4.421781 2.309498 -2.335054
C 6.401851 1.615052 0.345671
H 6.866108 -0.370459 1.056831
H 5.755171 3.447289 -0.602855
H 6.993102 2.117124 1.106225
H 1.364495 -1.385013 -0.672191
89
-3063.42236127
C 0.895152 3.118293 -3.238349
C -0.581269 3.199459 -2.828867
C -1.015155 4.634912 -2.503218
C -2.492658 4.767519 -2.066374
C 1.354222 1.793559 -3.859669
N 1.377784 0.565602 -3.027087
H -1.208719 2.810984 -3.644062
H -0.762525 2.550144 -1.966172
H -0.861154 5.263755 -3.391895
H -0.369958 5.049981 -1.715959
H 2.382961 1.896196 -4.222439
H 1.537201 3.362340 -2.382593
H 1.103456 3.884898 -3.999745
H -2.770568 5.828335 -2.108628
S -0.095706 -0.320307 -2.785819
O -0.770029 -0.214762 -4.085539
O -0.798852 0.066659 -1.554632
C 0.535081 -1.976202 -2.568650
C 0.911393 -2.706738 -3.699638
C 0.617859 -2.522596 -1.288721
C 1.382568 -4.004442 -3.533959
H 0.828093 -2.265312 -4.687044
C 1.094018 -3.826077 -1.147008
H 0.319846 -1.955116 -0.414707
C 1.480580 -4.584879 -2.258322

H 1.677683 -4.579530 -4.408320
H 1.158667 -4.251972 -0.149321
C 1.967940 -6.004919 -2.098132
H 2.800930 -6.221451 -2.775985
H 2.300535 -6.200988 -1.074324
H 1.169000 -6.722268 -2.327751
H 0.728040 1.526661 -4.715929
C -2.789728 4.241362 -0.676317
C -2.674187 5.085506 0.437906
C -3.176011 2.910947 -0.460672
C -2.938423 4.620129 1.726333
H -2.383405 6.123958 0.290526
C -3.439498 2.438072 0.826319
H -3.269620 2.236432 -1.308128
C -3.325013 3.292765 1.924550
H -2.852111 5.296397 2.573372
H -3.735241 1.402461 0.970294
H -3.544217 2.927909 2.924799
H -3.125709 4.252089 -2.800965
Cl 2.304015 0.740854 -1.532184
C 0.847308 0.853725 3.795527
C 1.766971 2.021050 3.403192
C 2.301113 1.973659 1.945898
C 3.079509 0.733860 1.623716
C -0.461522 0.805994 2.997388
N -1.387608 -0.257150 3.428904
H 2.622890 2.037266 4.089459
H 1.232947 2.971743 3.544848
H 2.907775 2.871471 1.778294
H 1.453924 2.049706 1.252010
H -1.004904 1.754468 3.086140
H 1.374754 -0.099278 3.672768
H 0.604077 0.943234 4.864426
H 2.502323 -0.163191 1.408157
S -1.213427 -1.826371 2.832823
O -1.244840 -2.727296 3.991976
O -0.085085 -1.834761 1.886980
C -2.710615 -2.107869 1.887683
C -2.841822 -1.552977 0.610839
C -3.724799 -2.886896 2.441205
C -4.013864 -1.781555 -0.104412
H -2.045250 -0.963476 0.169119
C -4.890876 -3.105184 1.706084
H -3.593151 -3.323758 3.425161
C -5.056748 -2.556249 0.429166
H -4.113049 -1.357111 -1.100636
H -5.681660 -3.717205 2.132867
C -6.322876 -2.784721 -0.361606
H -6.982072 -3.503422 0.134657
H -6.883678 -1.850107 -0.489076
H -6.101905 -3.165288 -1.365755
H -0.271422 0.652533 1.933267
C 4.488733 0.588464 1.677383
C 5.378208 1.651142 2.014405
C 5.078145 -0.678537 1.385528
C 6.750972 1.452923 2.052449
H 4.976564 2.631969 2.249637
C 6.450236 -0.864785 1.425497
H 4.423561 -1.506695 1.124936
C 7.301697 0.198426 1.759198
H 7.404262 2.281686 2.314245
H 6.867542 -1.842291 1.197131
H 8.377518 0.050978 1.790786
H -1.516974 -0.317529 4.436511
89
-3063.41909248
C 5.774336 0.232521 -1.042361
C 5.679631 1.547526 -0.253402
C 6.957384 1.946733 0.508431
C 7.251351 1.172167 1.814555
C 4.627553 -0.053641 -2.018724
N 3.362548 -0.429563 -1.325133
H 5.438902 2.356673 -0.957620
H 4.840952 1.505807 0.451861
H 6.868734 3.007584 0.778262
H 7.825350 1.877731 -0.162796
H 4.895796 -0.931550 -2.613042
H 5.872394 -0.623710 -0.368546
H 6.691813 0.252672 -1.649090
H 8.015384 1.735909 2.368417
S 2.389085 -1.572773 -2.253278
O 3.336728 -2.663611 -2.510198
O 1.683291 -0.929321 -3.366383
C 1.201300 -2.076659 -1.023342
C 1.641435 -2.708637 0.141188
C -0.157929 -1.890478 -1.278270
C 0.695716 -3.138253 1.068140
H 2.701989 -2.850385 0.319553
C -1.089997 -2.335784 -0.343317
H -0.471240 -1.400552 -2.193501
C -0.678095 -2.961308 0.843442
H 1.029198 -3.620890 1.983469
H -2.150091 -2.189018 -0.532926
C -1.699048 -3.445352 1.844715
H -1.239764 -3.649717 2.817206
H -2.499504 -2.711547 1.985361
H -2.170720 -4.375200 1.500175
H 4.454233 0.783025 -2.707188
C 7.731790 -0.258156 1.659239
C 8.964685 -0.537077 1.050350
C 6.974445 -1.335072 2.138133
C 9.419131 -1.848231 0.912631
H 9.578112 0.283598 0.683795
C 7.424361 -2.650473 2.003032
H 6.019148 -1.139970 2.620808
C 8.648369 -2.912156 1.387221
H 10.377857 -2.040127 0.437295
H 6.817323 -3.469660 2.380029
H 9.000912 -3.934486 1.280162
H 6.350751 1.185960 2.442167
Cl 2.388443 1.003936 -0.917922
C -4.187698 2.831181 -1.277726
C -2.801608 2.569326 -1.889769
C -1.796086 1.770192 -1.019090
C -1.460795 2.414803 0.291247
C -5.039342 1.582834 -0.996122
N -4.638693 0.932174 0.263573
H -2.355086 3.540657 -2.138758
H -2.932753 2.035096 -2.841566
H -0.887293 1.623960 -1.617183
H -2.205019 0.773246 -0.827872
H -6.106171 1.851306 -0.974167
H -4.089733 3.421838 -0.356288
H -4.757104 3.457548 -1.976988
H -2.048475 2.114188 1.156047
S -5.023154 -0.677981 0.544864
O -4.547663 -0.949491 1.905265
O -4.529383 -1.425300 -0.616827
C -6.812605 -0.830217 0.553524
C -7.490375 -1.118597 -0.632583
C -7.511401 -0.637752 1.748520
C -8.881944 -1.203047 -0.616711
H -6.931917 -1.302519 -1.544223
C -8.900818 -0.727301 1.744701
H -6.968290 -0.450153 2.669136
C -9.608667 -1.003563 0.564873
H -9.410892 -1.438437 -1.536856
H -9.445582 -0.590853 2.675738
C -11.116993 -1.071519 0.569251
H -11.495576 -1.506749 1.500063
H -11.555598 -0.068996 0.477936
H -11.494097 -1.670612 -0.265369
H -4.898449 0.846618 -1.793146
C -0.455006 3.388090 0.505863
C 0.394764 3.874859 -0.532594
C -0.251429 3.927671 1.811169
C 1.370301 4.828505 -0.273437
H 0.275946 3.496033 -1.542972
C 0.723881 4.880308 2.057497
H -0.880495 3.571605 2.623956

C 1.545084 5.341681 1.018227
H 2.003347 5.178971 -1.084804
H 0.853638 5.270141 3.064124
H 2.310109 6.087839 1.213258
H -4.811305 1.485052 1.100448
89
-3063.41651019
C 0.538890 4.355561 0.127978
C 0.003543 4.318294 1.570227
C -1.283922 3.517022 1.838978
C -2.579716 4.065776 1.197570
C 0.943231 3.029032 -0.524088
N 2.150562 2.428083 0.122475
H 0.790287 3.934899 2.231189
H -0.166049 5.359331 1.882045
H -1.437245 3.510240 2.926112
H -1.151930 2.465082 1.560333
H 1.238405 3.238155 -1.556149
H 1.413843 5.016965 0.096865
H -0.212240 4.801988 -0.534684
H -3.426617 3.618920 1.736945
S 3.211105 1.620735 -1.027697
O 3.491684 2.673567 -2.009292
O 2.672504 0.326492 -1.466680
C 4.658715 1.341309 -0.023317
C 5.474352 2.429218 0.299745
C 4.981043 0.046056 0.378683
C 6.623462 2.206398 1.050693
H 5.214151 3.425823 -0.040165
C 6.140120 -0.154884 1.127117
H 4.343001 -0.787218 0.106826
C 6.972619 0.915264 1.478835
H 7.264970 3.046597 1.304703
H 6.399806 -1.163078 1.438981
C 8.209544 0.693359 2.315512
H 8.017144 0.933566 3.369477
H 8.541862 -0.348313 2.271834
H 9.036756 1.330523 1.984549
H 0.122964 2.305693 -0.552301
C -2.771389 3.792814 -0.283584
C -2.986666 4.834584 -1.194888
C -2.772715 2.475677 -0.772065
C -3.187397 4.577115 -2.553834
H -2.997302 5.861874 -0.836067
C -2.971388 2.214774 -2.129040
H -2.623150 1.649446 -0.080789
C -3.178440 3.265178 -3.027308
H -3.349389 5.404001 -3.240820
H -2.965187 1.186427 -2.483378
H -3.331148 3.061894 -4.083920
H -2.643207 5.147412 1.378094
Cl 1.700881 1.295785 1.419260
C -1.295344 -3.764926 -1.500769
C -0.592658 -4.300541 -0.236031
C 0.377709 -3.316388 0.474873
C 1.534592 -2.870277 -0.370730
C -2.641442 -3.052241 -1.303880
N -2.590203 -1.747894 -0.616483
H -0.013613 -5.187270 -0.521170
H -1.344408 -4.630815 0.490542
H 0.739171 -3.818440 1.383225
H -0.175793 -2.439871 0.826670
H -3.102543 -2.885012 -2.285462
H -0.613736 -3.111176 -2.063208
H -1.506579 -4.608609 -2.171855
H 1.476313 -1.894080 -0.846205
S -3.125278 -1.585038 0.967730
O -2.549083 -0.305786 1.409358
O -2.900239 -2.809121 1.749163
C -4.901060 -1.419549 0.775167
C -5.430106 -0.347725 0.048581
C -5.737060 -2.341508 1.401660
C -6.810168 -0.216216 -0.053719
H -4.774156 0.373110 -0.428032
C -7.120560 -2.193194 1.286869
H -5.304389 -3.157713 1.969571
C -7.677876 -1.135245 0.560742
H -7.223850 0.616079 -0.618234
H -7.774661 -2.912264 1.773520
C -9.174051 -0.976327 0.433074
H -9.500879 0.006988 0.792124
H -9.493660 -1.058266 -0.613269
H -9.709057 -1.738820 1.006934
H -3.323295 -3.696153 -0.738984
C 2.706349 -3.627757 -0.624262
C 2.953327 -4.905798 -0.041287
C 3.709007 -3.110921 -1.500279
C 4.117967 -5.608243 -0.318463
H 2.221233 -5.334960 0.636128
C 4.868313 -3.822786 -1.768800
H 3.544036 -2.139369 -1.959204
C 5.085378 -5.078191 -1.182468
H 4.279195 -6.580228 0.141737
H 5.611881 -3.403509 -2.442330
H 5.993624 -5.634817 -1.396261
H -1.731425 -1.215350 -0.731165
89
-3063.41276493
C 0.533785 4.052652 0.573283
C -0.030281 3.729017 1.970001
C -1.236484 2.773692 2.047050
C -2.600931 3.342630 1.595504
C 1.026618 2.889263 -0.295866
N 2.309190 2.304068 0.197464
H 0.773884 3.316292 2.589898
H -0.307456 4.683826 2.440587
H -1.352017 2.473660 3.096779
H -1.032483 1.842467 1.506871
H 1.266728 3.287936 -1.285531
H 1.367101 4.758017 0.683540
H -0.231264 4.566182 -0.020261
H -3.367493 2.619813 1.905643
S 3.294922 1.638032 -1.105487
O 3.438087 2.772392 -2.024018
O 2.776231 0.352654 -1.591229
C 4.841386 1.364193 -0.258419
C 5.647613 2.465134 0.042699
C 5.242987 0.064283 0.045165
C 6.869847 2.249908 0.670762
H 5.323850 3.466216 -0.220815
C 6.473444 -0.128601 0.671510
H 4.610041 -0.778997 -0.207448
C 7.300158 0.954033 0.998347
H 7.504766 3.100487 0.906069
H 6.793490 -1.140309 0.906769
C 8.616803 0.738486 1.704990
H 8.509924 0.893132 2.786801
H 8.990749 -0.279021 1.555089
H 9.380795 1.439349 1.351882
H 0.269004 2.109612 -0.424820
C -2.770750 3.609588 0.111587
C -3.052203 4.895551 -0.367609
C -2.672935 2.563941 -0.821793
C -3.220407 5.138451 -1.733501
H -3.139695 5.718518 0.338793
C -2.838801 2.802406 -2.186969
H -2.468896 1.556962 -0.465944
C -3.111919 4.092412 -2.649747
H -3.435477 6.146488 -2.079380
H -2.754545 1.979266 -2.892669
H -3.239348 4.278209 -3.712891
H -2.804511 4.267917 2.151258
Cl 2.024755 1.043053 1.422254
C -1.359207 -4.040555 -1.326656
C -0.665628 -4.342706 0.017984
C 0.234944 -3.213730 0.594220
C 1.387014 -2.825454 -0.286733
C -2.743868 -3.380156 -1.258848
N -2.763680 -2.000958 -0.742327
H -0.036029 -5.229961 -0.119144

H -1.420050 -4.601171 0.770599
H 0.603586 -3.562846 1.569418
H -0.369013 -2.326598 0.810904
H -3.184083 -3.364161 -2.264049
H -0.696781 -3.439327 -1.965646
H -1.507885 -4.983966 -1.869410
H 1.307123 -1.905667 -0.861571
S -3.346016 -1.643226 0.789112
O -2.672372 -0.383716 1.143389
O -3.266187 -2.817140 1.668215
C -5.089593 -1.316687 0.523341
C -5.482918 -0.180863 -0.191787
C -6.035010 -2.186505 1.062274
C -6.838577 0.068533 -0.371697
H -4.742013 0.502694 -0.593407
C -7.391950 -1.919996 0.869568
H -5.707799 -3.050740 1.629709
C -7.814886 -0.796161 0.151746
H -7.146831 0.952947 -0.924200
H -8.131588 -2.596657 1.290110
C -9.282246 -0.511078 -0.062190
H -9.544449 0.498735 0.275128
H -9.546827 -0.573177 -1.125295
H -9.912462 -1.221975 0.480463
H -3.408684 -3.976661 -0.626523
C 2.586140 -3.565417 -0.444832
C 2.869930 -4.759666 0.281016
C 3.579638 -3.116669 -1.367209
C 4.059484 -5.449633 0.091337
H 2.147597 -5.132196 1.001010
C 4.763844 -3.815648 -1.548142
H 3.389155 -2.207110 -1.931238
C 5.016264 -4.989351 -0.822813
H 4.248599 -6.356760 0.660389
H 5.498839 -3.450833 -2.261673
H 5.943528 -5.536555 -0.968405
H -1.936873 -1.439913 -0.925966

(C5-rad---N-Cl)$_{XAT,bi}$

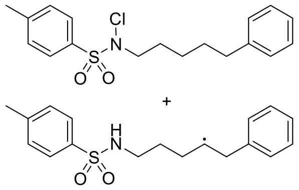

Name	E(B3LYP)	G(B3LYP)	E(RO-B2PLYP-D3)	G(RO-B2PLYP-D3)
Conformers				
Tosyl_NH_pentane_Ph_C5_radical_Tosyl_N_Cl_pentane_separate_no_hydrogen_bond_0009_B2PLYP	-3065.099370	-3064.314558	-3064.052484	-3063.41028179
Tosyl_NH_pentane_Ph_C5_radical_Tosyl_N_Cl_pentane_separate_no_hydrogen_bond_0003_B2PLYP	-3065.099370	-3064.314558	-3064.052466	-3063.41026123
Tosyl_NH_pentane_Ph_C5_radical_Tosyl_N_Cl_pentane_separate_no_hydrogen_bond_0018_B2PLYP	-3065.099370	-3064.314558	-3064.052466	-3063.41025727
Tosyl_NH_pentane_Ph_C5_radical_Tosyl_N_Cl_pentane_separate_no_hydrogen_bond_0008_B2PLYP	-3065.099370	-3064.314559	-3064.052470	-3063.41025651
Tosyl_NH_pentane_Ph_C5_radical_Tosyl_N_Cl_pentane_separate_no_hydrogen_bond_0011_B2PLYP	-3065.099370	-3064.314559	-3064.052470	-3063.41025283
Tosyl_NH_pentane_Ph_C5_radical_Tosyl_N_Cl_pentane_separate_no_hydrogen_bond_0017_B2PLYP	-3065.099370	-3064.314559	-3064.052475	-3063.41025155
Tosyl_NH_pentane_Ph_C5_radical_Tosyl_N_Cl_pentane_separate_no_hydrogen_bond_0014_B2PLYP	-3065.099370	-3064.314558	-3064.052465	-3063.41024726
Tosyl_NH_pentane_Ph_C5_radical_Tosyl_N_Cl_pentane_separate_no_hydrogen_bond_0002_B2PLYP	-3065.099370	-3064.314559	-3064.052466	-3063.41024660
Tosyl_NH_pentane_Ph_C5_radical_Tosyl_N_Cl_pentane_separate_no_hydrogen_bond_0007_B2PLYP	-3065.099370	-3064.314558	-3064.052468	-3063.41023286
Tosyl_NH_pentane_Ph_C5_radical_Tosyl_N_Cl_pentane_separate_no_hydrogen_bond_0006_B2PLYP	-3065.099370	-3064.314558	-3064.052470	-3063.41023211
Pre-reactive complexes for XAT				
Tosyl_NH_pentane_Ph_C5_H_Tosyl_N_pentane_TS_md_00030_IRC_forward_opt_B2PLYP_ric	-3065.099589	-3064.315496	-3064.046758	-3063.41175312
Tosyl_NH_pentane_Ph_C5_H_Tosyl_N_pentane_TS_md_00000_IRC_forward_opt_B2PLYP_ric	-3065.099801	-3064.315957	-3064.044073	-3063.41060281
Tosyl_NH_pentane_Ph_C5_H_Tosyl_N_pentane_TS_md_00100_IRC_reverse_opt_B2PLYP_ric	-3065.096588	-3064.312498	-3064.051037	-3063.41395083
Tosyl_NH_pentane_Ph_C5_H_Tosyl_N_pentane_TS_md_00350_IRC_reverse_opt_B2PLYP_ric	-3065.099037	-3064.314276	-3064.045424	-3063.40710229
Tosyl_NH_pentane_Ph_C5_H_Tosyl_N_pentane_TS_md_00016_IRC_reverse_opt_B2PLYP_ric	-3065.099030	-3064.314739	-3064.051046	-3063.41036832
Tosyl_NH_pentane_Ph_C5_H_Tosyl_N_pentane_TS_md_00012_IRC_forward_opt_B2PLYP_ric	-3065.099346	-3064.315247	-3064.049324	-3063.41207206
Tosyl_NH_pentane_Ph_C5_H_Tosyl_N_pentane_TS_md_00200_IRC_reverse_opt_B2PLYP_ric	-3065.098985	-3064.315131	-3064.049827	-3063.41191999
Tosyl_NH_pentane_Ph_C5_H_Tosyl_N_pentane_TS_md_00080_IRC_reverse_opt_B2PLYP_ric	-3065.099346	-3064.315246	-3064.049261	-3063.41160479
Tosyl_NH_pentane_Ph_C5_H_Tosyl_N_pentane_TS_md_00450_IRC_reverse_opt_B2PLYP_ric	-3065.098950	-3064.314875	-3064.051014	-3063.41348740
Tosyl_NH_pentane_Ph_C5_H_Tosyl_N_pentane_TS_md_00150_IRC_reverse_opt_B2PLYP_ric	-3065.096926	-3064.313003	-3064.053163	-3063.41459040

89
-3063.41028179
C 5.010323 -2.803430 0.752659
C 5.452604 -1.529373 0.016522
C 5.020088 -1.460888 -1.454354
C 5.697330 -0.323572 -2.251023
C 3.499050 -3.035841 0.847691
N 2.782723 -1.886598 1.455150

H 6.549574 -1.482085 0.068297
H 5.081724 -0.647835 0.551133
H 3.930834 -1.341917 -1.519564
H 5.260607 -2.412170 -1.951748
H 3.073836 -3.126253 -0.157731
H 5.423876 -3.687022 0.245906
H 5.434076 -2.805235 1.763087
H 6.784147 -0.477684 -2.233255
S 1.019550 -2.063472 1.369765
O 0.473076 -0.994557 2.202936
O 0.626254 -3.462788 1.590133
C 0.755422 -1.667602 -0.352574
C 0.514631 -2.700980 -1.261850
C 0.708337 -0.327824 -0.748382
C 0.223573 -2.381037 -2.586568
H 0.534644 -3.732870 -0.927215
C 0.411636 -0.032743 -2.076245
H 0.879403 0.465692 -0.028846
C 0.155223 -1.046671 -3.013573
H 0.025200 -3.179928 -3.296135
H 0.357206 1.006995 -2.388323
C -0.243147 -0.706342 -4.426422
H 0.011519 -1.511937 -5.122350
H -1.328179 -0.551962 -4.469859
H 0.241315 0.213405 -4.770695
H 3.281338 -3.964981 1.388830
C 5.382794 1.073408 -1.753052
C 6.340209 1.832218 -1.067395
C 4.118673 1.641860 -1.969267
C 6.048406 3.118761 -0.609709
H 7.328670 1.411902 -0.895125
C 3.820950 2.926313 -1.514534
H 3.360880 1.071516 -2.502350
C 4.787002 3.671269 -0.833106
H 6.808832 3.689722 -0.083141
H 2.835983 3.349728 -1.694188
H 4.559812 4.676135 -0.486713
H 5.390465 -0.410026 -3.302045
C -3.552463 3.359698 -0.625664
C -4.324984 3.301857 0.720399
C -3.642605 2.546594 1.816952
C -2.776384 3.208581 2.851682
C -3.297633 1.997651 -1.276401
N -4.574179 1.324720 -1.579619
H -5.307653 2.851713 0.523044
H -4.507540 4.336597 1.042527
H -4.110247 3.997167 -1.328341
H -2.579775 3.840948 -0.470683
H -3.186241 4.198068 3.097838
S -4.510003 -0.260197 -2.171174
O -3.287791 -0.480023 -2.962397
O -5.829675 -0.483775 -2.771762
C -4.355395 -1.214967 -0.665658
C -3.168135 -1.894482 -0.398447
C -5.435608 -1.275491 0.219938
C -3.057900 -2.634661 0.779624
H -2.346798 -1.840917 -1.103745
C -5.308367 -2.018716 1.388837
H -6.356488 -0.748378 -0.006916
C -4.120782 -2.707281 1.688439
H -2.129517 -3.156070 0.998441
H -6.143375 -2.065741 2.083771
C -4.001611 -3.525411 2.951734
H -4.523194 -3.048194 3.788405
H -4.446354 -4.520490 2.817660
H -2.954932 -3.668289 3.236409
H -2.742858 1.354112 -0.586129
C -1.321428 3.379091 2.411494
C -0.426074 2.301874 2.472605
C -0.855355 4.607727 1.924709
C 0.900750 2.452047 2.066369
H -0.758724 1.336373 2.844946
C 0.469433 4.759040 1.507681
H -1.533305 5.458188 1.883807
C 1.353422 3.681108 1.578292
H 1.579052 1.607106 2.146980
H 0.811374 5.723383 1.139361
H 2.387324 3.794454 1.263574
H -5.172898 1.852357 -2.215280
Cl 3.244574 -1.705757 3.166903
H -2.682054 2.116219 -2.178341
H -3.644891 1.459473 1.774821
H -2.793831 2.616882 3.776660
89
-3063.41026123
C 5.005980 -2.811564 0.747549
C 5.450896 -1.537220 0.013499
C 5.017925 -1.465112 -1.457069
C 5.697444 -0.327911 -2.251970
C 3.494211 -3.040591 0.842940
N 2.780908 -1.890739 1.452784
H 6.547993 -1.492566 0.064894
H 5.082294 -0.655774 0.549835
H 3.928919 -1.343534 -1.521610
H 5.256073 -2.416044 -1.956268
H 3.068276 -3.128244 -0.162421
H 5.417201 -3.695202 0.238981
H 5.430227 -2.816202 1.757763
H 6.783919 -0.484475 -2.234779
S 1.017285 -2.063252 1.367995
O 0.473729 -0.994334 2.203067
O 0.620742 -3.461959 1.586429
C 0.753345 -1.664095 -0.353602
C 0.508828 -2.695397 -1.264264
C 0.710240 -0.323622 -0.747430
C 0.217942 -2.372619 -2.588316
H 0.525899 -3.727834 -0.931153
C 0.413787 -0.025683 -2.074726
H 0.884251 0.468301 -0.026852
C 0.153557 -1.037410 -3.013350
H 0.016586 -3.169843 -3.298916
H 0.362660 1.014686 -2.385256
C -0.244546 -0.693949 -4.425512
H -1.329757 -0.540870 -4.469168
H 0.238981 0.227229 -4.767234
H 0.011409 -1.497499 -5.123357
H 3.274602 -3.970170 1.382560
C 5.386152 1.068886 -1.751458
C 4.123333 1.640633 -1.966587
C 6.345327 1.824243 -1.064453
C 3.828568 2.924926 -1.509485
H 3.364238 1.073012 -2.500719
C 6.056478 3.110600 -0.604371
H 7.332832 1.401353 -0.893001
C 4.796333 3.666400 -0.826696
H 2.844570 3.350932 -1.688319
H 6.818219 3.678844 -0.076768
H 4.571447 4.671135 -0.478424
H 5.390079 -0.411844 -3.303049
C -3.544821 3.359693 -0.628517
C -4.318399 3.306285 0.717097
C -3.639037 2.550728 1.815319
C -2.773119 3.212119 2.850661
C -3.293634 1.995900 -1.277000
N -4.572039 1.326218 -1.579632
H -5.302293 2.858773 0.519792
H -4.498087 4.342066 1.037469
H -4.100239 3.997718 -1.332563
H -2.570821 3.838325 -0.473625
H -3.182237 4.202111 3.096101
S -4.512200 -0.259167 -2.170607
O -3.290859 -0.482433 -2.962214
O -5.832690 -0.479521 -2.770558
C -4.359510 -1.213870 -0.664874
C -3.172423 -1.893132 -0.395975
C -5.440769 -1.274265 0.219347
C -3.063530 -2.632980 0.782364
H -2.350256 -1.839699 -1.100320
C -5.314874 -2.017247 1.388609
H -6.361453 -0.747390 -0.008846

C -4.127649 -2.705595 1.689831
H -2.135293 -3.154144 1.002445
H -6.150730 -2.064235 2.082511
C -4.009947 -3.523977 2.953090
H -2.964120 -3.655814 3.246229
H -4.543791 -3.053842 3.785964
H -4.442218 -4.523856 2.813897
H -2.740998 1.351836 -0.585502
C -1.317545 3.381133 2.411987
C -0.849882 4.609108 1.925048
C -0.423164 2.303199 2.474799
C 0.475552 4.759104 1.509604
H -1.527117 5.460077 1.882798
C 0.904294 2.452063 2.070175
H -0.757124 1.338165 2.847190
C 1.358577 3.680487 1.581998
H 0.818764 5.722947 1.141156
H 1.581816 1.606612 2.152033
H 2.392984 3.792839 1.268600
H -5.169354 1.855124 -2.215579
Cl 3.244039 -1.713997 3.164610
H -2.677358 2.111300 -2.178876
H -3.643897 1.463578 1.774542
H -2.792108 2.620777 3.775827
89
-3063.41025727
C 5.005043 -2.811978 0.748463
C 5.450150 -1.537771 0.014289
C 5.017400 -1.465854 -1.456358
C 5.697330 -0.329008 -2.251401
C 3.493248 -3.040992 0.843475
N 2.779807 -1.891113 1.453120
H 6.547242 -1.493179 0.065842
H 5.081523 -0.656221 0.550437
H 3.928435 -1.344017 -1.521072
H 5.255363 -2.416953 -1.955329
H 3.067572 -3.128658 -0.161992
H 5.416409 -3.695710 0.240174
H 5.429015 -2.816422 1.758791
H 6.783751 -0.485938 -2.234162
S 1.016191 -2.063567 1.367794
O 0.472444 -0.994759 2.202868
O 0.619570 -3.462301 1.585911
C 0.752720 -1.664123 -0.353805
C 0.508689 -2.695279 -1.264745
C 0.709521 -0.323565 -0.747377
C 0.218267 -2.372286 -2.588852
H 0.525774 -3.727778 -0.931829
C 0.413488 -0.025418 -2.074710
H 0.883131 0.468243 -0.026578
C 0.153823 -1.037017 -3.013639
H 0.017305 -3.169400 -3.299682
H 0.362283 1.015004 -2.385068
C -0.243639 -0.693265 -4.425908
H -1.328528 -0.537902 -4.469610
H 0.241859 0.226739 -4.768020
H 0.010581 -1.497623 -5.123436
H 3.273477 -3.970554 1.383056
C 5.386535 1.067988 -1.751123
C 6.346166 1.823301 -1.064702
C 4.123782 1.639995 -1.965932
C 6.057832 3.109868 -0.604896
H 7.333622 1.400201 -0.893492
C 3.829525 2.924502 -1.509093
H 3.364321 1.072420 -2.499590
C 4.797744 3.665933 -0.826903
H 6.819924 3.678072 -0.077756
H 2.845558 3.350692 -1.687662
H 4.573256 4.670824 -0.478828
H 5.389976 -0.412993 -3.302481
C -3.543931 3.360248 -0.628113
C -4.317630 3.306527 0.717434
C -3.638301 2.550791 1.815545
C -2.772370 3.212016 2.850999
C -3.292715 1.996601 -1.276905
N -4.571105 1.327071 -1.579904
H -5.301477 2.858997 0.519947
H -4.497400 4.342233 1.037998
H -4.099279 3.998452 -1.332053
H -2.569938 3.838820 -0.473012
H -3.181447 4.201988 3.096571
S -4.511322 -0.258257 -2.171033
O -3.289687 -0.481733 -2.962140
O -5.831616 -0.478293 -2.771555
C -4.359461 -1.213203 -0.665372
C -5.441027 -1.273340 0.218491
C -3.172711 -1.892952 -0.396192
C -5.315780 -2.016541 1.387687
H -6.361443 -0.746086 -0.009909
C -3.064461 -2.633003 0.782079
H -2.350305 -1.839722 -1.100273
C -4.128903 -2.705359 1.689196
H -6.151885 -2.063331 2.081303
H -2.136475 -3.154523 1.002381
C -4.011893 -3.523940 2.952396
H -4.546046 -3.053860 3.785102
H -4.444213 -4.523748 2.812846
H -2.966213 -3.655938 3.245989
H -2.740203 1.352340 -0.585492
C -1.316784 3.381005 2.412358
C -0.422486 2.302994 2.474890
C -0.849037 4.609056 1.925688
C 0.904971 2.451854 2.070250
H -0.756503 1.337900 2.847067
C 0.476394 4.759057 1.510244
H -1.526216 5.460079 1.883636
C 1.359339 3.680362 1.582353
H 1.582421 1.606324 2.151874
H 0.819654 5.722964 1.142008
H 2.393747 3.792703 1.268948
H -5.168269 1.856139 -2.215853
Cl 3.242408 -1.714471 3.165117
H -2.676307 2.112190 -2.178665
H -3.643093 1.463644 1.774570
H -2.791405 2.620526 3.776071
89
-3063.41025651
C 5.006229 -2.809625 0.749971
C 5.450600 -1.535441 0.015311
C 5.018080 -1.464489 -1.455451
C 5.697354 -0.327521 -2.250878
C 3.494575 -3.039629 0.844812
N 2.780223 -1.889953 1.453778
H 6.547651 -1.490069 0.067055
H 5.081262 -0.653904 0.550991
H 3.929040 -1.343459 -1.520426
H 5.256812 -2.415643 -1.953951
H 3.069150 -3.128060 -0.160696
H 5.418285 -3.693301 0.242144
H 5.430029 -2.813337 1.760375
H 6.783882 -0.483684 -2.233385
S 1.016728 -2.063743 1.368258
O 0.472089 -0.994934 2.202760
O 0.621090 -3.462665 1.586950
C 0.753287 -1.665267 -0.353574
C 0.510343 -2.697006 -1.264142
C 0.709026 -0.324905 -0.747715
C 0.219985 -2.374804 -2.588463
H 0.528216 -3.729355 -0.930798
C 0.413054 -0.027556 -2.075239
H 0.881774 0.467348 -0.027196
C 0.154514 -1.039765 -3.013823
H 0.019903 -3.172388 -3.299017
H 0.360982 1.012691 -2.386036
C -0.242847 -0.696906 -4.426341
H 0.011926 -1.501462 -5.123440
H -1.327796 -0.542081 -4.470310
H 0.242245 0.223186 -4.768795
H 3.275343 -3.969086 1.384790
C 5.385500 1.069484 -1.751280

C 6.344587 1.825890 -1.065303
C 4.122287 1.640399 -1.966296
C 6.055283 3.112467 -0.606134
H 7.332381 1.403640 -0.893937
C 3.827060 2.924908 -1.510089
H 3.363228 1.071962 -2.499611
C 4.794749 3.667438 -0.828340
H 6.816967 3.681529 -0.079329
H 2.842749 3.350239 -1.688802
H 4.569501 4.672331 -0.480758
H 5.390243 -0.412189 -3.301976
C -3.545723 3.360226 -0.627892
C -4.319542 3.305672 0.717558
C -3.639810 2.550162 1.815577
C -2.773762 3.211618 2.850791
C -3.293399 1.996873 -1.276870
N -4.571240 1.326466 -1.580256
H -5.303051 2.857506 0.519866
H -4.500059 4.341190 1.038327
H -4.101464 3.998140 -1.331785
H -2.572105 3.839494 -0.472596
H -3.182886 4.201592 3.096268
S -4.510169 -0.258838 -2.171269
O -3.288130 -0.481554 -2.961954
O -5.830107 -0.479803 -2.772235
C -4.358096 -1.213536 -0.665465
C -3.171531 -1.893797 -0.396716
C -5.439310 -1.273107 0.218854
C -3.063125 -2.633787 0.781557
H -2.349370 -1.840913 -1.101109
C -5.313905 -2.016266 1.388076
H -6.359569 -0.745408 -0.009152
C -4.127229 -2.705608 1.689127
H -2.135264 -3.155661 1.001557
H -6.149722 -2.062587 2.082068
C -4.009998 -3.524255 2.952257
H -4.439939 -4.524975 2.811868
H -2.964392 -3.654110 3.247097
H -4.546190 -3.055584 3.784433
H -2.740578 1.352894 -0.585439
C -1.318244 3.380663 2.411936
C -0.423886 2.302695 2.474339
C -0.850613 4.608749 1.925229
C 0.903517 2.451626 2.069534
H -0.757797 1.337581 2.846564
C 0.474758 4.758819 1.509623
H -1.527837 5.459741 1.883281
C 1.357766 3.680162 1.581602
H 1.581016 1.606128 2.151075
H 0.817934 5.722751 1.141368
H 2.392130 3.792562 1.268071
H -5.168576 1.855100 -2.216402
Cl 3.242425 -1.712165 3.165772
H -2.676885 2.113043 -2.178482
H -3.644340 1.463010 1.774653
H -2.792613 2.620271 3.775962
89
-3063.41025283
C 5.003991 -2.812432 0.749319
C 5.449501 -1.538190 0.015452
C 5.017393 -1.466107 -1.455377
C 5.697830 -0.329299 -2.250047
C 3.492146 -3.041370 0.843664
N 2.778495 -1.891482 1.453061
H 6.546575 -1.493686 0.067482
H 5.080715 -0.656662 0.551528
H 3.928472 -1.344110 -1.520550
H 5.255440 -2.417203 -1.954314
H 3.066886 -3.128966 -0.161988
H 5.415527 -3.696146 0.241136
H 5.427525 -2.816988 1.759831
H 6.784223 -0.486379 -2.232329
S 1.014901 -2.063997 1.367069
O 0.470786 -0.995268 2.202023
O 0.618257 -3.462767 1.584955
C 0.752136 -1.664430 -0.354612
C 0.508031 -2.695481 -1.265664
C 0.709513 -0.323854 -0.748148
C 0.218085 -2.372341 -2.589836
H 0.524704 -3.727997 -0.932778
C 0.413998 -0.025559 -2.075570
H 0.883182 0.467879 -0.027281
C 0.154231 -1.037024 -3.014595
H 0.017059 -3.169359 -3.300758
H 0.363274 1.014892 -2.385905
C -0.242648 -0.693108 -4.426992
H -1.327686 -0.539014 -4.471448
H 0.241976 0.227643 -4.768320
H 0.013060 -1.496826 -5.124723
H 3.272098 -3.970946 1.383107
C 5.386985 1.067702 -1.749816
C 6.346257 1.822696 -1.062546
C 4.124523 1.640024 -1.965506
C 6.057851 3.109254 -0.602749
H 7.333496 1.399360 -0.890659
C 3.830200 2.924524 -1.508694
H 3.365352 1.072695 -2.499846
C 4.798060 3.665632 -0.825637
H 6.819667 3.677204 -0.074936
H 2.846472 3.350971 -1.687965
H 4.573524 4.670522 -0.477582
H 5.390935 -0.413175 -3.301269
C -3.542076 3.360294 -0.629337
C -4.316588 3.307585 0.715778
C -3.638356 2.551847 1.814578
C -2.772293 3.212935 2.850009
C -3.291141 1.996226 -1.277344
N -4.569656 1.327104 -1.580722
H -5.300602 2.860594 0.517922
H -4.495886 4.343537 1.035829
H -4.096689 3.998457 -1.333896
H -2.567939 3.838458 -0.473884
H -3.181035 4.203112 3.095338
S -4.510189 -0.258504 -2.171083
O -3.288413 -0.482775 -2.961734
O -5.830378 -0.478411 -2.771890
C -4.359005 -1.212703 -0.664879
C -3.173024 -1.893787 -0.395720
C -5.440375 -1.271044 0.219354
C -3.065321 -2.633389 0.782899
H -2.350758 -1.841876 -1.100066
C -5.315680 -2.013785 1.388882
H -6.360199 -0.742735 -0.008998
C -4.129567 -2.703949 1.690374
H -2.137888 -3.155895 1.003189
H -6.151628 -2.059171 2.082785
C -4.013351 -3.521956 2.954013
H -2.967712 -3.657527 3.246098
H -4.544453 -3.049280 3.787215
H -4.449547 -4.520288 2.815986
H -2.739299 1.352046 -0.585318
C -1.316562 3.381338 2.411587
C -0.848169 4.609279 1.925253
C -0.422773 2.302899 2.474048
C 0.477408 4.758758 1.510086
H -1.524965 5.460608 1.883235
C 0.904838 2.451242 2.069700
H -0.757300 1.337876 2.845951
C 1.359855 3.679647 1.582147
H 0.821194 5.722575 1.142102
H 1.581885 1.605383 2.151278
H 2.394375 3.791606 1.268962
H -5.166280 1.856147 -2.217196
Cl 3.240451 -1.714868 3.165232
H -2.674187 2.111099 -2.178823
H -3.644251 1.464681 1.774298
H -2.791678 2.621638 3.775193
89
-3063.41025155
C 5.005288 2.810938 -0.749227

C 5.450095 1.536630 -0.015045
C 5.017595 1.464965 1.455689
C 5.697244 0.327898 2.250659
C 3.493549 3.040444 -0.843934
N 2.779578 1.890699 -1.453246
H 6.547162 1.491645 -0.066804
H 5.081048 0.655178 -0.551061
H 3.928595 1.343543 1.520614
H 5.256012 2.416003 1.954560
H 3.068128 3.128372 0.161620
H 5.417055 3.694568 -0.241089
H 5.429056 2.815158 -1.759642
H 6.783721 0.484427 2.233231
S 1.015984 2.063946 -1.367683
O 0.471658 0.995251 -2.202538
O 0.619953 3.462822 -1.585918
C 0.752712 1.664800 0.354020
C 0.509347 2.696130 1.264946
C 0.709111 0.324295 0.747719
C 0.219219 2.373359 2.589174
H 0.526736 3.728596 0.931935
C 0.413378 0.026375 2.075174
H 0.882189 -0.467646 0.026936
C 0.154417 1.038145 3.014104
H 0.018796 3.170611 3.300003
H 0.361800 -1.014001 2.385623
C -0.242615 0.694655 4.426565
H -1.327539 0.539713 4.470753
H 0.242656 -0.225523 4.768533
H 0.012251 1.498948 5.123935
H 3.273953 3.969999 -1.383591
C 5.385841 -1.068992 1.750458
C 4.122864 -1.640471 1.965357
C 6.345118 -1.824719 1.063998
C 3.828048 -2.924865 1.508555
H 3.363664 -1.072572 2.499042
C 6.056222 -3.111173 0.604227
H 7.332741 -1.402033 0.892726
C 4.795915 -3.666708 0.826317
H 2.843917 -3.350645 1.687197
H 6.818046 -3.679702 0.077051
H 4.570988 -4.671513 0.478273
H 5.390099 0.412022 3.301790
C -3.543531 -3.359854 0.629469
C -4.318088 -3.306248 -0.715597
C -3.639470 -2.550646 -1.814240
C -2.773749 -3.211942 -2.849832
C -3.291698 -1.996101 1.277792
N -4.569791 -1.326323 1.581506
H -5.301791 -2.858680 -0.517511
H -4.498075 -4.341992 -1.035935
H -4.098550 -3.997828 1.333873
H -2.569713 -3.838612 0.473855
H -3.182771 -4.202015 -3.095102
S -4.509525 0.259333 2.171520
O -3.287457 0.483328 2.961763
O -5.829452 0.479881 2.772662
C -4.358297 1.213139 0.665057
C -5.439547 1.271210 -0.219321
C -3.172325 1.894256 0.395897
C -5.314757 2.013700 -1.389020
H -6.359363 0.742867 0.008994
C -3.064525 2.633578 -0.782866
H -2.350143 1.842537 1.100350
C -4.128655 2.703866 -1.690506
H -6.150626 2.058872 -2.083030
H -2.137104 3.156103 -1.003166
C -4.012242 3.521620 -2.954283
H -2.966583 3.656008 -3.246873
H -4.544245 3.049435 -3.787185
H -4.447263 4.520445 -2.816130
H -2.739604 -1.352069 0.585830
C -1.317985 -3.380705 -2.411679
C -0.849834 -4.608743 -1.925372
C -0.423914 -2.302510 -2.474357
C 0.475775 -4.758559 -1.510411
H -1.526827 -5.459913 -1.883231
C 0.903711 -2.451181 -2.070204
H -0.758241 -1.337420 -2.846274
C 1.358485 -3.679681 -1.582652
H 0.819351 -5.722469 -1.142476
H 1.580973 -1.605512 -2.151948
H 2.393024 -3.791866 -1.269627
H -5.166602 -1.855005 2.218094
Cl 3.241780 1.713695 -3.165363
H -2.674686 -2.111540 2.179159
H -3.645010 -1.463483 -1.773920
H -2.793152 -2.620614 -3.774998
89
-3063.41024726
C -5.004359 2.813085 0.747617
C -5.449783 1.538744 0.013868
C -5.017039 1.466234 -1.456754
C -5.697305 0.329345 -2.251447
C -3.492507 3.041752 0.842575
N -2.779357 1.891911 1.452627
H -6.546887 1.494448 0.065427
H -5.081388 0.657278 0.550313
H -3.928109 1.344058 -1.521418
H -5.254716 2.417246 -1.956027
H -3.066792 3.128965 -0.162917
H -5.415491 3.696748 0.239019
H -5.428346 2.817984 1.757937
H -6.783679 0.486612 -2.234259
S -1.015678 2.063979 1.367289
O -0.472172 0.995362 2.202770
O -0.618781 3.462713 1.584911
C -0.752279 1.663854 -0.354164
C -0.507837 2.694618 -1.265444
C -0.709564 0.323151 -0.747281
C -0.217468 2.371072 -2.589424
H -0.524579 3.727235 -0.932876
C -0.413597 0.024447 -2.074510
H -0.883502 -0.468354 -0.026229
C -0.153498 1.035630 -3.013760
H -0.016182 3.167872 -3.300516
H -0.362799 -1.016100 -2.384520
C 0.243946 0.691294 -4.425895
H 1.328927 0.536599 -4.469635
H -0.241024 -0.229210 -4.767399
H -0.010867 1.495091 -5.123858
H -3.272519 3.971448 1.381835
C -5.386945 -1.067597 -1.750744
C -6.346836 -1.822430 -1.064161
C -4.124351 -1.640042 -1.965327
C -6.058911 -3.108954 -0.603976
H -7.334176 -1.398988 -0.893123
C -3.830503 -2.924510 -1.508113
H -3.364692 -1.072847 -2.499109
C -4.798979 -3.665459 -0.825762
H -6.821200 -3.676780 -0.076714
H -2.846655 -3.351045 -1.686514
H -4.574808 -4.670318 -0.477386
H -5.389925 0.412910 -3.302554
C 3.542200 -3.360280 -0.628364
C 4.316217 -3.307239 0.717026
C 3.637564 -2.551330 1.815435
C 2.771621 -3.212318 2.851038
C 3.291730 -1.996385 -1.276926
N 4.570492 -1.327641 -1.580055
H 5.300273 -2.860228 0.519400
H 4.495479 -4.343105 1.037360
H 4.097005 -3.998737 -1.332505
H 2.567940 -3.838266 -0.473134
H 3.180468 -4.202403 3.096537
S 4.511678 0.257783 -2.171007
O 3.290308 0.482076 -2.962282
O 5.832190 0.477132 -2.771299
C 4.360109 1.212611 -0.665241
C 5.441591 1.272099 0.218753

C 3.173653 1.892915 -0.396116
C 5.316575 2.015249 1.388020
H 6.361768 0.744401 -0.009583
C 3.065628 2.632884 0.782212
H 2.351299 1.840154 -1.100297
C 4.130022 2.704638 1.689451
H 6.152620 2.061546 2.081739
H 2.137854 3.154808 1.002471
C 4.013294 3.523267 2.952642
H 4.549019 3.054086 3.784835
H 4.444055 4.523654 2.812407
H 2.967760 3.653935 3.247366
H 2.739754 -1.351858 -0.585329
C 1.315914 -3.380899 2.412653
C 0.847717 -4.608833 1.926119
C 0.421947 -2.302619 2.475296
C -0.477835 -4.758460 1.510921
H 1.524637 -5.460057 1.883983
C -0.905626 -2.451104 2.070901
H 0.756318 -1.337606 2.847365
C -1.360449 -3.679499 1.583141
H -0.821452 -5.722285 1.142801
H -1.582816 -1.605374 2.152617
H -2.394943 -3.791550 1.269916
H 5.167255 -1.857038 -2.216106
Cl -3.242028 1.715977 3.164684
H 2.675078 -2.111459 -2.178588
H 3.642878 -1.464179 1.774639
H 2.790984 -2.620837 3.776109
89
-3063.41024660
C -5.005205 -2.811726 -0.748512
C -5.450156 -1.537494 -0.014285
C -5.017357 -1.465655 1.456350
C -5.697160 -0.328764 2.251435
C -3.493433 -3.040904 -0.843541
N -2.779856 -1.891063 -1.453089
H -6.547246 -1.492791 -0.065801
H -5.081458 -0.655968 -0.550420
H -3.928377 -1.343928 1.521032
H -5.255396 -2.416743 1.955305
H -3.067771 -3.128690 0.161924
H -5.416668 -3.695432 -0.240258
H -5.429189 -2.816083 -1.758836
H -6.783597 -0.485597 2.234237
S -1.016235 -2.063818 -1.367874
O -0.472361 -0.995072 -2.202951
O -0.619859 -3.462612 -1.586026
C -0.752606 -1.664441 0.353713
C -0.508849 -2.695653 1.264666
C -0.709075 -0.323890 0.747268
C -0.218399 -2.372719 2.588776
H -0.526166 -3.728151 0.931757
C -0.413025 -0.025802 2.074614
H -0.882459 0.467951 0.026448
C -0.153665 -1.037457 3.013561
H -0.017671 -3.169876 3.299626
H -0.361575 1.014607 2.384970
C 0.243756 -0.693832 4.425873
H -0.011415 -1.497825 5.123479
H 1.328777 -0.539460 4.469834
H -0.240964 0.226687 4.767696
H -3.273766 -3.970452 -1.383189
C -5.386264 1.068216 1.751170
C -6.345888 1.823654 1.064873
C -4.123430 1.640084 1.965868
C -6.057469 3.110206 0.605082
H -7.333406 1.400663 0.893749
C -3.829086 2.924577 1.509040
H -3.363971 1.072413 2.499426
C -4.797299 3.666132 0.826974
H -6.819556 3.678506 0.078038
H -2.845055 3.350657 1.687518
H -4.572739 4.671011 0.478908
H -5.389777 -0.412790 3.302504
C 3.543338 3.360258 0.628320
C 4.317279 3.306968 -0.717093
C 3.638431 2.551196 -1.815477
C 2.772445 3.212365 -2.850929
C 3.292344 1.996451 1.276872
N 4.570835 1.327124 1.579909
H 5.301237 2.859722 -0.519503
H 4.496795 4.342780 -1.037470
H 4.098423 3.998481 1.332450
H 2.569253 3.838625 0.473152
H 3.181413 4.202394 -3.096476
S 4.511214 -0.258261 2.170997
O 3.289673 -0.481856 2.962208
O 5.831566 -0.478207 2.771424
C 4.359330 -1.213126 0.665284
C 5.440798 -1.272988 -0.218724
C 3.172707 -1.893146 0.396241
C 5.315583 -2.016202 -1.387916
H 6.361114 -0.745512 0.009563
C 3.064485 -2.633203 -0.782029
H 2.350374 -1.840114 1.100423
C 4.128831 -2.705310 -1.689276
H 6.151608 -2.062784 -2.081642
H 2.136594 -3.154937 -1.002224
C 4.011894 -3.523929 -2.952454
H 4.545732 -3.053662 -3.785259
H 4.444607 -4.523577 -2.812982
H 2.966216 -3.656300 -3.245875
H 2.740041 1.352179 0.585300
C 1.316845 3.381150 -2.412288
C 0.848862 4.609148 -1.925712
C 0.422746 2.302960 -2.474720
C -0.476590 4.758918 -1.510236
H 1.525862 5.460319 -1.883766
C -0.904725 2.451586 -2.070051
H 0.756942 1.337911 -2.846856
C -1.359322 3.680039 -1.582220
H -0.820017 5.722799 -1.142079
H -1.582018 1.605927 -2.151642
H -2.393734 3.792188 -1.268759
H 5.167824 1.856236 2.215988
Cl -3.242494 -1.714195 -3.165074
H 2.675814 2.111786 2.178582
H 3.643498 1.464047 -1.774642
H 2.791578 2.620906 -3.776016
89
-3063.41023286
C 5.004671 -2.812917 0.746793
C 5.449971 -1.538386 0.013304
C 5.017042 -1.465515 -1.457244
C 5.697083 -0.328329 -2.251706
C 3.492836 -3.041654 0.841843
N 2.779709 -1.891952 1.452180
H 6.547081 -1.494071 0.064742
H 5.081616 -0.657064 0.550014
H 3.928092 -1.343432 -1.521740
H 5.254757 -2.416364 -1.956811
H 3.067039 -3.128684 -0.163629
H 5.415778 -3.696449 0.237946
H 5.428765 -2.818042 1.757068
H 6.783479 -0.485466 -2.234687
S 1.016034 -2.063997 1.366885
O 0.472569 -0.995564 2.202640
O 0.619146 -3.462789 1.584198
C 0.752551 -1.663467 -0.354468
C 0.508248 -2.694018 -1.266026
C 0.709589 -0.322665 -0.747211
C 0.217778 -2.370155 -2.589906
H 0.525170 -3.726728 -0.933752
C 0.413522 -0.023641 -2.074347
H 0.883420 0.468675 -0.025953
C 0.153558 -1.034603 -3.013868
H 0.016607 -3.166792 -3.301214
H 0.362536 1.016986 -2.384059
C -0.243992 -0.689946 -4.425896

H 0.240763 0.230769 -4.767141
H 0.010998 -1.493477 -5.124100
H -1.329007 -0.535495 -4.469584
H 3.272925 -3.971463 1.380939
C 5.386600 1.068434 -1.750585
C 6.346444 1.823180 -1.063842
C 4.123919 1.640788 -1.964909
C 6.058388 3.109528 -0.603244
H 7.333850 1.399811 -0.893001
C 3.829939 2.925076 -1.507281
H 3.364297 1.073658 -2.498813
C 4.798370 3.665938 -0.824769
H 6.820643 3.677289 -0.075863
H 2.846022 3.351539 -1.685468
H 4.574096 4.670662 -0.476070
H 5.389592 -0.411651 -3.302799
C -3.542933 3.359863 -0.628783
C -4.316816 3.306896 0.716685
C -3.638202 2.550804 1.814994
C -2.772435 3.211590 2.850861
C -3.292427 1.995923 -1.277245
N -4.571161 1.326891 -1.579853
H -5.300965 2.860051 0.519134
H -4.495892 4.342772 1.037085
H -4.097841 3.998231 -1.332921
H -2.568681 3.837915 -0.473692
H -3.181389 4.201584 3.096562
S -4.512202 -0.258592 -2.170679
O -3.291129 -0.482693 -2.962456
O -5.832920 -0.478313 -2.770391
C -4.359721 -1.213180 -0.664852
C -3.173221 -1.893737 -0.396565
C -5.440515 -1.272263 0.220011
C -3.064445 -2.633549 0.781791
H -2.351403 -1.841250 -1.101392
C -5.314753 -2.015254 1.389298
H -6.360739 -0.744356 -0.007659
C -4.128127 -2.704890 1.689893
H -2.136619 -3.155640 1.001427
H -6.150252 -2.061222 2.083697
C -4.010560 -3.523298 2.953146
H -4.442390 -4.523336 2.813709
H -2.964779 -3.654806 3.246615
H -4.544841 -3.053425 3.785881
H -2.740120 1.351596 -0.585728
C -1.316689 3.380433 2.412692
C -0.848664 4.608448 1.926188
C -0.422518 2.302337 2.475520
C 0.476923 4.758326 1.511203
H -1.525751 5.459532 1.883903
C 0.905099 2.451078 2.071355
H -0.756766 1.337270 2.847564
C 1.359749 3.679549 1.583626
H 0.820413 5.722204 1.143101
H 1.582442 1.605482 2.153193
H 2.394283 3.791809 1.270607
H -5.168257 1.856087 -2.215760
Cl 3.242497 -1.716344 3.164219
H -2.676080 2.110986 -2.179116
H -3.643565 1.463660 1.774020
H -2.791846 2.619882 3.775783
89
-3063.41023211
C 5.005412 -2.811467 0.747715
C 5.450165 -1.537057 0.013685
C 5.017225 -1.464987 -1.456897
C 5.696816 -0.327862 -2.251822
C 3.493681 -3.040857 0.842811
N 2.780003 -1.891227 1.452662
H 6.547253 -1.492240 0.065121
H 5.081420 -0.655665 0.550013
H 3.928225 -1.343382 -1.521456
H 5.255326 -2.415948 -1.956068
H 3.067955 -3.128489 -0.162639
H 5.416939 -3.695036 0.239272
H 5.429475 -2.815958 1.758006
H 6.783267 -0.484619 -2.234833
S 1.016420 -2.064085 1.367411
O 0.472497 -0.995593 2.202795
O 0.620152 -3.462972 1.585185
C 0.752769 -1.664238 -0.354083
C 0.509207 -2.695197 -1.265370
C 0.708913 -0.323568 -0.747201
C 0.218575 -2.371890 -2.589362
H 0.526790 -3.727808 -0.932820
C 0.412671 -0.025106 -2.074420
H 0.882178 0.468079 -0.026143
C 0.153436 -1.036503 -3.013687
H 0.017990 -3.168861 -3.300461
H 0.360942 1.015400 -2.384412
C -0.244386 -0.692439 -4.425788
H 0.240691 0.227870 -4.767680
H 0.010019 -1.496462 -5.123632
H -1.329342 -0.537492 -4.469189
H 3.274174 -3.970544 1.382280
C 5.385905 1.068981 -1.751186
C 6.345702 1.824447 -1.065168
C 4.122889 1.640690 -1.965249
C 6.057285 3.110882 -0.605043
H 7.333351 1.401568 -0.894518
C 3.828544 2.925059 -1.508078
H 3.363285 1.072994 -2.498575
C 4.796936 3.666650 -0.826306
H 6.819512 3.679212 -0.078234
H 2.844362 3.351005 -1.686036
H 4.572374 4.671438 -0.477977
H 5.389282 -0.411676 -3.302866
C -3.544165 3.359946 -0.628236
C -4.317741 3.306328 0.717377
C -3.638447 2.550440 1.815406
C -2.772565 3.211501 2.851007
C -3.293163 1.996270 -1.277049
N -4.571635 1.326784 -1.579804
H -5.301694 2.858981 0.519981
H -4.497306 4.342057 1.037982
H -4.099488 3.998226 -1.332126
H -2.570096 3.838402 -0.473230
H -3.181583 4.201489 3.096615
S -4.511945 -0.258621 -2.170735
O -3.290727 -0.482168 -2.962457
O -5.832510 -0.478885 -2.770580
C -4.359107 -1.213180 -0.664927
C -5.439552 -1.271955 0.220394
C -3.172748 -1.894190 -0.397208
C -5.313564 -2.015093 1.389561
H -6.359669 -0.743661 -0.006810
C -3.063743 -2.634141 0.781040
H -2.351192 -1.841895 -1.102353
C -4.127058 -2.705198 1.689580
H -6.148777 -2.060802 2.084322
H -2.136001 -3.156547 1.000265
C -4.009261 -3.523810 2.952674
H -4.542340 -3.053448 3.785906
H -4.442284 -4.523375 2.813531
H -2.963370 -3.656427 3.245243
H -2.740567 1.351985 -0.585722
C -1.316923 3.380389 2.412530
C -0.849033 4.608413 1.925923
C -0.422702 2.302317 2.475176
C 0.476470 4.758339 1.510679
H -1.526155 5.459476 1.883770
C 0.904822 2.451100 2.070720
H -0.756835 1.337241 2.847296
C 1.359342 3.679590 1.582913
H 0.819845 5.722238 1.142525
H 1.582215 1.605530 2.152431
H 2.393805 3.791870 1.269670
H -5.168826 1.855802 -2.215773
Cl 3.242690 -1.714699 3.164639
H -2.676908 2.111808 -2.178922

H -3.643311 1.463297 1.774336

89
-3063.41459040
C -1.018858 -2.414621 2.011123
C -2.333113 -3.213389 1.924781
C -3.557815 -2.410704 1.461123
C -4.839545 -3.266186 1.406332
C -0.256414 -2.231173 0.693403
N -1.028067 -1.447531 -0.303475
H -2.188018 -4.077281 1.259318
H -2.545972 -3.631381 2.919142
H -3.369145 -1.979498 0.473136
H -3.720055 -1.564017 2.142748
H 0.719208 -1.767105 0.881074
H -0.320061 -2.938393 2.677317
H -1.196624 -1.436203 2.474555
H -4.668989 -4.115065 0.729926
S -0.632083 -1.672265 -1.975800
O -1.578920 -0.851713 -2.726124
O -0.575996 -3.128765 -2.123619
C 1.011605 -1.016315 -2.267996
C 1.154587 0.292915 -2.731137
C 2.130409 -1.819604 -2.033629
C 2.435960 0.801092 -2.946580
H 0.273321 0.887908 -2.942896
C 3.401624 -1.295343 -2.254111
H 2.002434 -2.846203 -1.708058
C 3.577623 0.021624 -2.706070
H 2.549992 1.813785 -3.325984
H 4.273775 -1.919008 -2.076805
C 4.962499 0.586115 -2.914382
H 4.946489 1.448641 -3.587932
H 5.396617 0.915625 -1.961583
H 5.639560 -0.164046 -3.336415
H -0.106557 -3.207895 0.223722
C -6.051036 -2.484934 0.942685
C -6.267879 -2.245426 -0.422060
C -6.965451 -1.953613 1.861465
C -7.363967 -1.498320 -0.855397
H -5.570935 -2.651711 -1.152095
C -8.063336 -1.204358 1.434480
H -6.816250 -2.132094 2.924517
C -8.266927 -0.973738 0.073149
H -7.517293 -1.332550 -1.918921
H -8.761768 -0.804931 2.165708
H -9.124598 -0.396713 -0.262891
H -5.034714 -3.693960 2.399121
Cl -1.083362 0.281712 0.127139
C 1.351250 3.170951 1.410358
C 0.585316 3.318742 0.086985
C -0.903148 3.302149 0.244860
C -1.803067 3.593388 -0.921664
C 2.876305 3.104294 1.247129
N 3.413987 1.838402 0.706179
H 0.883509 2.522718 -0.616953
H 0.897992 4.259930 -0.404360
H -1.330160 3.315316 1.245273
H 3.221743 3.899228 0.573470
H 1.117025 4.031842 2.051281
H 1.005875 2.279457 1.947877
H -1.580546 4.608574 -1.301538
S 3.652827 0.547103 1.771665
O 2.672248 -0.517475 1.509422
O 3.784184 1.127444 3.112243
C 5.252169 -0.084802 1.257438
C 5.390396 -1.447613 1.002193
C 6.356283 0.770601 1.192535
C 6.647745 -1.954611 0.668267
H 4.522289 -2.094446 1.063345
C 7.600929 0.249205 0.855141
H 6.238249 1.830282 1.393890
C 7.769002 -1.120485 0.590711
H 6.756970 -3.017748 0.468574
H 8.459439 0.914171 0.797912
H -2.791749 2.619956 3.776041

C 9.132294 -1.674009 0.251521
H 9.064765 -2.688460 -0.152902
H 9.647024 -1.047406 -0.485620
H 9.772373 -1.714991 1.142263
H 3.365185 3.258885 2.210689
C -3.281834 3.491364 -0.601260
C -4.002468 2.324367 -0.885460
C -3.947263 4.557930 0.017780
C -5.353870 2.221622 -0.549370
H -3.501064 1.488478 -1.366585
C -5.298632 4.460569 0.351662
H -3.401878 5.473949 0.237601
C -6.004996 3.289379 0.070603
H -5.894725 1.304124 -0.765148
H -5.799310 5.299404 0.828953
H -7.056724 3.208765 0.332626
H 2.939740 1.500366 -0.130539
H -1.554756 2.918760 -1.755965
89
-3063.41395083
C 2.055734 -1.302439 -2.654985
C 3.492371 -1.847858 -2.757730
C 4.489353 -1.268354 -1.743799
C 5.905508 -1.853019 -1.919860
C 1.162027 -1.967370 -1.601585
N 1.634042 -1.750301 -0.213536
H 3.470589 -2.943973 -2.667448
H 3.864483 -1.636655 -3.770256
H 4.140448 -1.460554 -0.723944
H 4.533539 -0.176333 -1.854834
H 0.123641 -1.624325 -1.714962
H 1.540755 -1.464376 -3.611757
H 2.069494 -0.216667 -2.500125
H 5.859686 -2.940882 -1.770933
S 1.063657 -2.882960 0.979599
O 1.670340 -2.479285 2.242884
O 1.333406 -4.183483 0.361802
C -0.713588 -2.696599 1.132331
C -1.233627 -1.851515 2.116140
C -1.558907 -3.405740 0.275528
C -2.616015 -1.719878 2.233823
H -0.561526 -1.328601 2.787295
C -2.938585 -3.255902 0.404723
H -1.141475 -4.083025 -0.461694
C -3.490618 -2.417162 1.383932
H -3.025606 -1.076266 3.008772
H -3.598376 -3.810489 -0.257538
C -4.986826 -2.292056 1.549454
H -5.341777 -2.931879 2.367939
H -5.279022 -1.263979 1.787300
H -5.518150 -2.593110 0.641512
H 1.188346 -3.052214 -1.738900
C 6.920920 -1.252159 -0.970804
C 6.946004 -1.624277 0.381481
C 7.840398 -0.290399 -1.409714
C 7.858303 -1.050546 1.267731
H 6.243576 -2.372673 0.742299
C 8.757270 0.286113 -0.527758
H 7.840128 0.006877 -2.456431
C 8.768307 -0.091390 0.815997
H 7.861568 -1.355962 2.311012
H 9.465741 1.025984 -0.892156
H 9.481776 0.353683 1.504456
H 6.236448 -1.695774 -2.955327
Cl 1.378228 -0.061111 0.292722
C -1.632942 2.205339 0.602713
C -0.601009 2.980063 1.435741
C 0.759985 3.036366 0.814999
C 1.924892 3.580030 1.591664
C -3.000161 2.127251 1.290180
N -3.985066 1.249860 0.622538
H -0.527344 2.538987 2.443375
H -0.974855 4.009271 1.605154

H 0.835885 3.012188 -0.270639
H -2.890240 1.730170 2.307202
H -1.744697 2.674227 -0.382664
H -1.260654 1.188586 0.426184
H 1.725423 4.639305 1.844239
S -4.685012 1.831398 -0.814138
O -3.949693 1.343268 -1.990420
O -4.896942 3.268063 -0.606849
C -6.269044 0.991861 -0.791850
C -6.532096 0.001317 -1.735510
C -7.233349 1.362539 0.150113
C -7.775212 -0.634504 -1.724126
H -5.774944 -0.256140 -2.468349
C -8.465792 0.718751 0.146315
H -7.017611 2.145539 0.869675
C -8.757778 -0.288356 -0.789232
H -7.984946 -1.407114 -2.459719
H -9.218544 1.003328 0.877681
C -10.110680 -0.959055 -0.794836
H -10.107445 -1.865098 -1.408339
H -10.424884 -1.235011 0.218128
H -10.880074 -0.288625 -1.199068
H -3.458319 3.115940 1.376313
C 3.254005 3.485994 0.868081
C 4.122500 2.411854 1.097399
C 3.628264 4.461942 -0.065003
C 5.334254 2.309969 0.411398
H 3.846234 1.645516 1.817626
C 4.837353 4.364759 -0.755379
H 2.968222 5.307531 -0.249106
C 5.693487 3.287403 -0.518620
H 5.996968 1.471208 0.603577
H 5.112139 5.133401 -1.473561
H 6.637338 3.207916 -1.051219
H -3.597070 0.323963 0.436360
H 1.988957 3.066700 2.563171
89
-3063.41348740
C 1.404592 -3.216468 0.249521
C 2.810302 -3.778002 0.532006
C 3.972850 -2.964399 -0.054944
C 5.345650 -3.598844 0.246348
C 0.926187 -2.096141 1.179283
N 1.750876 -0.863142 1.074957
H 2.947054 -3.881906 1.618564
H 2.859003 -4.797617 0.123657
H 3.951206 -1.942512 0.337482
H 3.847355 -2.881893 -1.143756
H -0.132613 -1.876403 0.993931
H 0.664631 -4.019106 0.369091
H 1.324360 -2.891884 -0.795361
H 5.472564 -3.665455 1.335664
S 1.656453 0.238833 2.428850
O 2.520501 1.366126 2.091162
O 1.923628 -0.628520 3.578129
C -0.028780 0.833088 2.546135
C -0.389363 2.013088 1.889174
C -0.964478 0.104261 3.283118
C -1.710898 2.449897 1.965742
H 0.354000 2.589003 1.349278
C -2.280162 0.558097 3.346116
H -0.661285 -0.792116 3.812605
C -2.677357 1.727348 2.683639
H -1.990570 3.376512 1.470024
H -3.009155 -0.006561 3.921320
C -4.114422 2.188860 2.716346
H -4.648108 1.847348 1.819325
H -4.644236 1.792803 3.588465
H -4.185382 3.281472 2.741052
H 1.046363 -2.414601 2.219118
C 6.501199 -2.825977 -0.353649
C 7.003326 -1.680633 0.280763
C 7.075247 -3.213946 -1.571310
C 8.045058 -0.944985 -0.284339
H 6.572371 -1.363815 1.228260
C 8.118469 -2.481963 -2.141373
H 6.702110 -4.102833 -2.076336
C 8.607228 -1.343543 -1.499094
H 8.420109 -0.061421 0.226044
H 8.551330 -2.803919 -3.085393
H 9.421189 -0.773177 -1.939054
H 5.358254 -4.630314 -0.131471
Cl 1.451235 -0.023643 -0.460032
C -2.010581 1.239685 -2.909275
C -1.026479 2.228793 -2.258367
C 0.306010 2.267109 -2.938558
C 1.096764 3.533313 -3.105523
C -3.329765 1.079492 -2.139255
N -3.236033 0.396788 -0.834381
H -0.890185 1.956766 -1.195761
H -1.468061 3.236749 -2.241454
H 0.697132 1.342432 -3.357289
H -3.780092 2.060945 -1.943424
H -2.248445 1.587158 -3.923742
H -1.544481 0.253936 -3.014574
H 2.082193 3.289247 -3.522326
S -3.194966 -1.288690 -0.792639
O -2.574168 -1.613227 0.500307
O -2.663478 -1.846408 -2.043418
C -4.932841 -1.725224 -0.724776
C -5.671215 -1.439510 0.427823
C -5.520349 -2.372350 -1.809183
C -7.012507 -1.799757 0.479651
H -5.194474 -0.950286 1.270970
C -6.869799 -2.725312 -1.740091
H -4.923806 -2.600763 -2.685679
C -7.634537 -2.446733 -0.602274
H -7.590112 -1.580217 1.374566
H -7.331476 -3.229673 -2.585137
C -9.090014 -2.840810 -0.522530
H -9.247495 -3.619594 0.234284
H -9.719094 -1.987949 -0.241464
H -9.453847 -3.227969 -1.478862
H -4.052642 0.520209 -2.741231
C 1.274032 4.343789 -1.825681
C 0.709110 5.617378 -1.692370
C 2.009265 3.823796 -0.749541
C 0.871857 6.356620 -0.517339
H 0.140510 6.038551 -2.519231
C 2.173987 4.556670 0.426257
H 2.455324 2.835939 -0.827954
C 1.603357 5.827780 0.546015
H 0.427593 7.345709 -0.436572
H 2.742108 4.125917 1.246100
H 1.732224 6.400623 1.460657
H -2.517342 0.751068 -0.204422
H 0.607697 4.183157 -3.853056
89
-3063.41207206
C 3.110850 -1.322133 -2.632826
C 4.608391 -1.526719 -2.336678
C 5.149711 -0.735688 -1.137393
C 6.649340 -0.995650 -0.891564
C 2.147194 -2.177014 -1.801315
N 2.182651 -1.856619 -0.355109
H 4.804722 -2.599046 -2.189756
H 5.176938 -1.235876 -3.231324
H 4.584042 -0.993095 -0.236066
H 4.991359 0.337976 -1.307702
H 1.123661 -2.086534 -2.192022
H 2.910106 -1.594683 -3.678012
H 2.844487 -0.262052 -2.540292
H 6.793258 -2.066770 -0.691736
S 1.636433 -3.106733 0.727393
O 1.770541 -2.560887 2.073547
O 2.391668 -4.279778 0.280307
C -0.102403 -3.412740 0.420449
C -1.060841 -2.825269 1.250068
C -0.482459 -4.259922 -0.623946
C -2.409923 -3.092097 1.020834

H -0.743748 -2.200344 2.077723
C -1.836490 -4.501198 -0.846286
H 0.271997 -4.747698 -1.231730
C -2.822855 -3.922907 -0.034447
H -3.157216 -2.666467 1.687417
H -2.132945 -5.163441 -1.655381
C -4.288101 -4.177913 -0.289156
H -4.745236 -3.329757 -0.814669
H -4.435903 -5.071699 -0.902909
H -4.835558 -4.315363 0.650094
H 2.452946 -3.225387 -1.864005
C 7.214290 -0.184329 0.255713
C 8.042605 0.921556 0.021938
C 6.900695 -0.507728 1.584293
C 8.541875 1.685447 1.079994
H 8.305945 1.181901 -1.001431
C 7.396053 0.251683 2.644256
H 6.263309 -1.365304 1.788980
C 8.218751 1.352984 2.396027
H 9.187063 2.536114 0.874653
H 7.142065 -0.019242 3.665945
H 8.607291 1.943588 3.221486
H 7.212543 -0.773393 -1.807991
Cl 1.402859 -0.286018 -0.028462
C -1.993156 1.331244 0.212163
C -1.106034 2.363346 0.923440
C 0.211444 2.593681 0.252742
C 1.315608 3.308748 0.977294
C -3.333049 1.121894 0.924794
N -4.149060 0.020277 0.381576
H -0.939694 2.056469 1.968584
H -1.656465 3.322829 0.993697
H 0.264471 2.513458 -0.830920
H -3.168532 0.892408 1.985388
H -2.181925 1.639491 -0.822090
H -1.459906 0.372821 0.168079
H 0.900307 4.223595 1.441331
S -5.122091 0.300794 -0.968471
O -5.376089 -1.041468 -1.512230
O -4.580749 1.361770 -1.829088
C -6.622877 0.942387 -0.228330
C -7.338184 0.157676 0.681889
C -7.089050 2.197286 -0.613208
C -8.524586 0.649042 1.213274
H -6.964040 -0.819071 0.970132
C -8.283160 2.674571 -0.069169
H -6.521574 2.784049 -1.327242
C -9.017332 1.913549 0.846877
H -9.081862 0.043528 1.924162
H -8.648267 3.654680 -0.365272
C -10.314309 2.425108 1.426601
H -10.303698 2.386556 2.522312
H -10.507437 3.459429 1.127086
H -11.163643 1.816351 1.091877
H -3.941232 2.032434 0.894846
C 2.500346 3.687741 0.111011
C 3.751088 3.085634 0.289014
C 2.362613 4.659222 -0.890931
C 4.840155 3.444722 -0.509439
H 3.877855 2.328479 1.058887
C 3.444941 5.018019 -1.694054
H 1.398420 5.141682 -1.039136
C 4.688932 4.411578 -1.504261
H 5.803570 2.969243 -0.346097
H 3.318703 5.775229 -2.463890
H 5.534716 4.694386 -2.125674
H -3.615470 -0.830356 0.202156
H 1.657972 2.698647 1.828056
89
-3063.41191999
C 0.653642 -2.893605 0.303628
C 1.925819 -3.730045 0.532646
C 3.236004 -3.081281 0.063063
C 4.458640 -3.988693 0.307660
C 0.372948 -1.796896 1.337393
N 1.385502 -0.711452 1.315696
H 2.004086 -3.981788 1.600698
H 1.803733 -4.687897 0.006395
H 3.384040 -2.124029 0.572955
H 3.167050 -2.852833 -1.009878
H -0.636525 -1.391807 1.189600
H -0.222534 -3.554976 0.341448
H 0.657937 -2.459570 -0.704127
H 4.527966 -4.204936 1.382659
S 1.521676 0.243807 2.769289
O 2.553783 1.241475 2.497874
O 1.676155 -0.766898 3.818646
C -0.032426 1.093046 3.015538
C -0.180115 2.402456 2.555911
C -1.093811 0.423943 3.634050
C -1.412356 3.037860 2.708085
H 0.664059 2.914753 2.108103
C -2.316965 1.071617 3.767089
H -0.954681 -0.581196 4.016730
C -2.500116 2.382404 3.299928
H -1.528685 4.062490 2.363013
H -3.148450 0.550481 4.233232
C -3.847979 3.048786 3.419211
H -4.570160 2.549343 2.762595
H -4.236376 2.984028 4.442316
H -3.801358 4.106584 3.141685
H 0.441539 -2.223718 2.342595
C 5.758752 -3.378504 -0.171793
C 6.437747 -2.433182 0.610294
C 6.299612 -3.717509 -1.418874
C 7.619301 -1.844205 0.160049
H 6.034909 -2.157544 1.582675
C 7.481897 -3.131480 -1.874651
H 5.789354 -4.452761 -2.038153
C 8.146468 -2.191699 -1.085670
H 8.130539 -1.115662 0.784367
H 7.885162 -3.412444 -2.844520
H 9.068839 -1.736011 -1.436432
H 4.299447 -4.953446 -0.193325
Cl 1.163872 0.331332 -0.117431
C -1.701683 0.984054 -2.430890
C -1.110951 2.335386 -1.997902
C 0.374010 2.430182 -2.162863
C 1.128949 3.592979 -1.584895
C -3.235371 0.950705 -2.452670
N -3.917813 1.146902 -1.159870
H -1.368211 2.544567 -0.946760
H -1.595943 3.144202 -2.581970
H 0.854173 1.830848 -2.933879
H -3.621486 1.731430 -3.120680
H -1.357052 0.755978 -3.449422
H -1.328043 0.188482 -1.779637
H 0.671576 4.533382 -1.946328
S -4.103951 -0.147866 -0.098610
O -4.514525 0.479130 1.165274
O -2.957167 -1.069839 -0.127451
C -5.492925 -1.021122 -0.818790
C -6.734680 -0.384054 -0.911051
C -5.332186 -2.342293 -1.230472
C -7.816578 -1.084069 -1.431672
H -6.844844 0.644004 -0.582198
C -6.430429 -3.029347 -1.751202
H -4.361796 -2.818069 -1.140470
C -7.683730 -2.416604 -1.859119
H -8.783246 -0.591987 -1.508090
H -6.308097 -4.059281 -2.076444
C -8.874491 -3.164966 -2.408386
H -9.376510 -2.591974 -3.196663
H -8.582363 -4.132506 -2.827109
H -9.618344 -3.352264 -1.623637
H -3.587019 -0.004089 -2.857005
C 2.609355 3.602186 -1.912423
C 3.550997 3.074985 -1.019305
C 3.058991 4.121332 -3.133989
C 4.909130 3.064881 -1.343365

H 3.223454 2.663918 -0.067703
C 4.415581 4.112641 -3.460032
H 2.338961 4.540571 -3.834446
C 5.345733 3.582808 -2.563584
H 5.624721 2.649329 -0.639037
H 4.745909 4.523018 -4.411063
H 6.403348 3.575611 -2.813959
H -3.605490 1.952096 -0.620953
H 0.986482 3.612683 -0.492923
89
-3063.41175312
C 3.844505 1.117606 0.157759
C 4.663753 0.781821 -1.099925
C 5.764829 -0.268140 -0.894178
C 6.628393 -0.478574 -2.154761
C 3.022390 -0.069380 0.668929
N 2.218556 0.310790 1.852501
H 3.985775 0.449953 -1.900344
H 5.122974 1.711384 -1.462769
H 5.329223 -1.233388 -0.603208
H 6.413069 0.035934 -0.060504
H 2.384056 -0.478156 -0.128454
H 3.173595 1.953880 -0.067958
H 4.508450 1.452157 0.964593
H 5.976642 -0.771793 -2.988689
S 1.736512 -0.976797 2.922057
O 1.023272 -0.337822 4.022356
O 2.982497 -1.720227 3.122810
C 0.581568 -2.041304 2.058914
C -0.787558 -1.913990 2.310037
C 1.058940 -3.008980 1.172101
C -1.677462 -2.765498 1.658710
H -1.134093 -1.179461 3.028735
C 0.150560 -3.838943 0.516839
H 2.126054 -3.130998 1.020070
C -1.228447 -3.733625 0.743997
H -2.740615 -2.695611 1.880221
H 0.520936 -4.593358 -0.172188
C -2.210214 -4.634967 0.036768
H -2.874240 -4.056225 -0.616287
H -1.696100 -5.382588 -0.574430
H -2.844488 -5.166246 0.756831
H 3.687029 -0.859844 1.025256
C 7.711856 -1.519036 -1.965451
C 8.960675 -1.167200 -1.435043
C 7.481130 -2.864609 -2.281507
C 9.948495 -2.129510 -1.223053
H 9.161523 -0.126159 -1.189637
C 8.465331 -3.831631 -2.071794
H 6.520460 -3.156225 -2.701665
C 9.703471 -3.466821 -1.540520
H 10.911275 -1.834105 -0.813804
H 8.266356 -4.869257 -2.327747
H 10.472548 -4.217464 -1.379078
H 7.081663 0.480998 -2.437897
Cl 0.848650 1.351674 1.390275
C -2.697330 1.464861 0.056085
C -2.489317 2.898118 0.566728
C -1.175041 3.488338 0.162748
C -0.696471 4.777312 0.767576
C -4.031649 0.864143 0.507848
N -4.207218 -0.556574 0.152091
H -2.585993 2.924480 1.664204
H -3.314668 3.536593 0.193490
H -0.724811 3.159315 -0.771634
H -4.129256 0.927275 1.599042
H -2.648361 1.442421 -1.038257
H -1.880196 0.832069 0.426902
H -1.468199 5.555673 0.615627
S -4.777977 -0.955681 -1.385379
O -4.359578 -2.352687 -1.572252
O -4.431474 0.065015 -2.384210
C -6.554635 -0.887721 -1.161513
C -7.168060 -1.720285 -0.220006
C -7.310438 -0.042614 -1.970724
C -8.551283 -1.689847 -0.088984
H -6.566503 -2.374835 0.401905
C -8.699017 -0.025708 -1.825252
H -6.813168 0.588007 -2.699561
C -9.339517 -0.844123 -0.888612
H -9.031811 -2.332627 0.644813
H -9.291579 0.634885 -2.453015
C -10.842002 -0.833842 -0.739142
H -11.275924 -1.793343 -1.047529
H -11.137611 -0.668107 0.303581
H -11.300631 -0.049762 -1.348828
H -4.876182 1.419724 0.086208
C 0.622082 5.271448 0.206086
C 1.827756 5.034036 0.877686
C 0.661245 5.958347 -1.014784
C 3.041915 5.470591 0.343203
H 1.815195 4.502135 1.825670
C 1.872135 6.394996 -1.553379
H -0.267416 6.156423 -1.546362
C 3.067998 6.152048 -0.874732
H 3.966481 5.281529 0.882677
H 1.880953 6.929504 -2.499911
H 4.011866 6.495612 -1.289819
H -3.377149 -1.123829 0.324024
H -0.621354 4.666981 1.860522
89
-3063.41160479
C 3.098409 -1.283037 -2.637027
C 4.598671 -1.492764 -2.359383
C 5.153248 -0.724828 -1.151181
C 6.656553 -0.986531 -0.930238
C 2.141754 -2.144475 -1.804169
N 2.184241 -1.831084 -0.356492
H 4.796312 -2.567729 -2.234983
H 5.157801 -1.185125 -3.254386
H 4.599709 -1.001334 -0.247963
H 4.990669 0.351711 -1.297756
H 1.115786 -2.054482 -2.188498
H 2.886710 -1.545006 -3.682772
H 2.834672 -0.223519 -2.531478
H 6.805485 -2.061494 -0.756666
S 1.646508 -3.088353 0.722263
O 1.783086 -2.548270 2.070458
O 2.404825 -4.256233 0.266958
C -0.092114 -3.399941 0.419780
C -1.050289 -2.817553 1.253271
C -0.472056 -4.246734 -0.624999
C -2.399041 -3.089237 1.027642
H -0.733181 -2.192934 2.081152
C -1.825766 -4.493061 -0.843526
H 0.282498 -4.730439 -1.235904
C -2.811908 -3.920093 -0.027610
H -3.145960 -2.667584 1.697157
H -2.122098 -5.155187 -1.652764
C -4.276842 -4.180771 -0.278284
H -4.423047 -5.077956 -0.887469
H -4.822149 -4.315293 0.662612
H -4.737397 -3.336558 -0.807155
H 2.449518 -3.191826 -1.873851
C 7.234055 -0.200222 0.228051
C 6.952715 -0.565825 1.552797
C 8.041293 0.924202 0.009250
C 7.458702 0.170626 2.623875
H 6.331850 -1.438178 1.745510
C 8.551008 1.665316 1.078418
H 8.279852 1.217186 -1.011381
C 8.259964 1.290807 2.390685
H 7.229692 -0.132860 3.642306
H 9.179506 2.531043 0.884573
H 8.656871 1.863587 3.224683
H 7.207353 -0.741955 -1.848541
Cl 1.401834 -0.264680 -0.018276
C -2.005725 1.338937 0.218607
C -1.128548 2.379539 0.929830
C 0.189638 2.617611 0.263215

C 1.283302 3.347883 0.988481
C -3.346019 1.121596 0.928051
N -4.154016 0.014798 0.383417
H -0.963671 2.077092 1.976503
H -1.686354 3.335120 0.995206
H 0.246951 2.534988 -0.820085
H -3.182690 0.893765 1.989183
H -2.193905 1.643324 -0.816897
H -1.465281 0.384373 0.178368
H 0.861353 4.271576 1.428636
S -5.126213 0.289361 -0.968421
O -5.372230 -1.054496 -1.511893
O -4.589104 1.352674 -1.828814
C -6.631542 0.923481 -0.231059
C -7.344321 0.135434 0.678241
C -7.103407 2.175942 -0.616993
C -8.534022 0.620979 1.207636
H -6.965675 -0.839299 0.967419
C -8.300734 2.647367 -0.074956
H -6.537711 2.765400 -1.330220
C -9.032564 1.882859 0.840086
H -9.089310 0.012908 1.917894
H -8.670233 3.625606 -0.371802
C -10.333404 2.387779 1.416965
H -11.179938 1.780380 1.072704
H -10.328699 2.340476 2.512332
H -10.526685 3.424097 1.124509
H -3.959674 2.028395 0.896084
C 2.479392 3.710704 0.131000
C 2.359877 4.676204 -0.878949
C 3.722638 3.097967 0.324516
C 3.452756 5.018817 -1.674886
H 1.401776 5.166929 -1.039098
C 4.822225 3.440808 -0.466565
H 3.834713 2.345231 1.100960
C 4.689091 4.401900 -1.469593
H 3.340736 5.771712 -2.451124
H 5.779655 2.957452 -0.291420
H 5.543098 4.672222 -2.085286
H -3.614974 -0.832679 0.205314
H 1.614018 2.754781 1.855615
89
-3063.41060281
C -3.787211 0.812809 -1.149888
C -4.794403 0.138439 -2.091880
C -5.951089 -0.530583 -1.338486
C -6.955085 -1.236180 -2.272086
C -2.647024 1.479729 -1.925368
N -1.526675 2.063855 -1.141928
H -4.275642 -0.614781 -2.703600
H -5.198648 0.880110 -2.796780
H -6.484525 0.221518 -0.740919
H -5.549585 -1.260349 -0.622281
H -2.166940 0.767290 -2.604780
H -4.300257 1.563619 -0.537006
H -3.375414 0.068606 -0.458446
H -7.358864 -0.502510 -2.982672
S -1.982198 3.382826 -0.098656
O -2.517225 2.939021 1.194503
O -2.785324 4.239756 -0.978669
C -0.388854 4.142434 0.186413
C 0.137876 4.159611 1.475939
C 0.277040 4.757796 -0.877001
C 1.358774 4.797096 1.698026
H -0.404311 3.682638 2.284918
C 1.494348 5.385258 -0.635996
H -0.153376 4.741498 -1.872554
C 2.053234 5.417705 0.652427
H 1.775711 4.813685 2.701819
H 2.020188 5.862320 -1.459468
C 3.362190 6.128085 0.901192
H 3.223204 7.216984 0.905358
H 3.794465 5.847467 1.866447
H 4.095778 5.900278 0.119819
H -3.021282 2.311113 -2.531016
C -8.091626 -1.899102 -1.522903
C -7.979191 -3.220463 -1.068997
C -9.268434 -1.196021 -1.231258
C -9.008717 -3.821696 -0.343753
H -7.075653 -3.784892 -1.291092
C -10.301287 -1.791931 -0.506256
H -9.377177 -0.171072 -1.580452
C -10.174483 -3.108262 -0.059028
H -8.902305 -4.849399 -0.005478
H -11.207057 -1.229217 -0.294706
H -10.979084 -3.575618 0.502528
H -6.419770 -1.986355 -2.869518
Cl -0.641643 0.795166 -0.214730
C 2.686868 -0.949117 0.183164
C 2.006580 -0.699852 1.536894
C 0.580478 -1.150544 1.593721
C -0.251345 -0.869519 2.812729
C 4.141284 -0.466301 0.167258
N 4.837653 -0.640463 -1.118643
H 2.066052 0.369329 1.798243
H 2.583594 -1.217953 2.328227
H 0.259905 -1.946020 0.923606
H 4.190756 0.602981 0.409315
H 2.665773 -2.017795 -0.056242
H 2.125787 -0.427423 -0.603959
H 0.274908 -1.274908 3.698218
S 5.511502 -2.143943 -1.496374
O 5.562705 -2.162743 -2.962548
O 4.856813 -3.224333 -0.745085
C 7.177069 -1.996876 -0.848614
C 8.057171 -1.068336 -1.413098
C 7.589337 -2.840109 0.180327
C 9.356525 -0.984633 -0.926943
H 7.724621 -0.424582 -2.220814
C 8.898397 -2.741666 0.656554
H 6.893402 -3.562428 0.592795
C 9.799052 -1.818677 0.114323
H 10.043559 -0.262785 -1.362360
H 9.222820 -3.397409 1.460646
C 11.219388 -1.723981 0.618351
H 11.489959 -0.688874 0.857672
H 11.367053 -2.328702 1.518121
H 11.931318 -2.075275 -0.139356
H 4.728867 -0.987160 0.930851
C -1.654847 -1.441738 2.767015
C -2.766515 -0.615311 2.560927
C -1.862475 -2.819287 2.923989
C -4.054639 -1.154729 2.512323
H -2.625836 0.454661 2.429545
C -3.147075 -3.360898 2.871630
H -1.009082 -3.473361 3.092914
C -4.249466 -2.528386 2.665631
H -4.906111 -0.497416 2.356875
H -3.287589 -4.431495 2.997816
H -5.252146 -2.946117 2.629115
H 4.304633 -0.332413 -1.930273
H -0.296309 0.216308 2.984563
89
-3063.41036832
C 3.105008 -1.030304 -2.891362
C 4.584539 -1.457966 -2.933816
C 5.376971 -1.385457 -1.617151
C 5.486797 0.034653 -1.028841
C 2.191829 -1.890277 -2.006833
N 2.327090 -1.558193 -0.566503
H 4.635516 -2.488518 -3.313811
H 5.087688 -0.834573 -3.686943
H 6.389362 -1.768214 -1.804864
H 4.930171 -2.053178 -0.873126
H 1.143381 -1.793907 -2.319414
H 2.702347 -1.098033 -3.911054
H 3.005933 0.022169 -2.602737
H 5.946632 0.698266 -1.773938
S 1.959423 -2.822338 0.568119
O 2.188273 -2.248277 1.891011

O 2.739122 -3.954752 0.061754
C 0.220210 -3.230838 0.427975
C -0.693525 -2.673634 1.325450
C -0.201500 -4.120412 -0.564533
C -2.042018 -3.012508 1.216060
H -0.341540 -2.014540 2.111433
C -1.554318 -4.435128 -0.665570
H 0.523699 -4.582164 -1.225886
C -2.498018 -3.887198 0.216006
H -2.751848 -2.608710 1.934990
H -1.882931 -5.130260 -1.433619
C -3.964205 -4.218740 0.085074
H -4.479458 -3.461504 -0.519966
H -4.111049 -5.188975 -0.399680
H -4.454174 -4.248656 1.064322
H 2.483387 -2.941357 -2.091049
C 6.287250 0.084574 0.256094
C 7.607012 0.555508 0.270612
C 5.726632 -0.361617 1.463723
C 8.349633 0.583348 1.453429
H 8.058062 0.903868 -0.656731
C 6.466252 -0.336642 2.646325
H 4.705589 -0.736524 1.476460
C 7.780658 0.136795 2.646382
H 9.371328 0.955186 1.441199
H 6.013151 -0.687485 3.570109
H 8.355213 0.158101 3.568853
H 4.482594 0.429824 -0.839241
Cl 1.485644 -0.033480 -0.175055
C -1.956648 1.365087 0.266847
C -1.139866 2.446132 0.990678
C 0.176475 2.752296 0.348036
C 1.246948 3.459078 1.129430
C -3.297606 1.091238 0.956152
N -4.034562 -0.070962 0.427082
H -0.976441 2.149159 2.038724
H -1.744592 3.373255 1.048158
H 0.237729 2.748537 -0.738376
H -3.142512 0.898014 2.025444
H -2.139554 1.655266 -0.773595
H -1.373672 0.435652 0.240810
H 0.799559 4.345866 1.618047
S -4.985202 0.111172 -0.955292
O -5.155046 -1.260871 -1.455874
O -4.477067 1.168071 -1.840806
C -6.535322 0.699110 -0.274699
C -7.232491 -0.089520 0.646127
C -7.055265 1.913856 -0.715673
C -8.455713 0.357636 1.131170
H -6.816140 -1.034652 0.978504
C -8.285665 2.346989 -0.217623
H -6.500644 2.504199 -1.436827
C -9.003151 1.581072 0.707595
H -8.999094 -0.250793 1.850269
H -8.692706 3.295922 -0.557383
C -10.340007 2.043448 1.235716
H -10.365880 2.019409 2.331476
H -10.567084 3.064307 0.914569
H -11.150304 1.394160 0.880717
H -3.958952 1.962088 0.891429
C 2.448908 3.897735 0.316978
C 3.704724 3.309652 0.506526
C 2.323703 4.912880 -0.642337
C 4.810207 3.724328 -0.240298
H 3.823697 2.519356 1.243817
C 3.423470 5.327894 -1.392955
H 1.355854 5.385640 -0.798039
C 4.672205 4.734434 -1.192785
H 5.774792 3.252807 -0.072957
H 3.307008 6.118534 -2.130017
H 5.531100 5.060160 -1.773804
H -3.449623 -0.895326 0.289700
H 1.576280 2.821132 1.964545
89
-3063.40894642
C -2.205260 0.866996 1.541793
C -3.396618 -0.072819 1.803450
C -3.489434 -1.291129 0.873265
C -4.716858 -2.172558 1.179746
C -0.868371 0.439075 2.158131
N -0.340800 -0.819564 1.583221
H -3.368512 -0.410636 2.849924
H -4.320548 0.514113 1.701213
H -2.579447 -1.894419 0.953417
H -3.544448 -0.949889 -0.170132
H -0.130736 1.249071 2.064832
H -2.421742 1.853656 1.972668
H -2.084335 1.035277 0.464743
H -4.650038 -2.522249 2.219151
S 0.812436 -1.691845 2.560195
O 1.159378 -2.884365 1.792646
O 0.151113 -1.771505 3.865030
C 2.281069 -0.681949 2.728908
C 3.323735 -0.821731 1.808838
C 2.361456 0.241811 3.774628
C 4.452340 -0.015138 1.942195
H 3.257702 -1.557834 1.014992
C 3.498180 1.038789 3.888448
H 1.556487 0.315115 4.497964
C 4.558798 0.925091 2.977808
H 5.268835 -0.125459 1.232898
H 3.566484 1.755272 4.703260
C 5.801888 1.767902 3.131834
H 6.532229 1.271180 3.784111
H 6.291418 1.940940 2.168181
H 5.574592 2.740571 3.579986
H -1.013184 0.222127 3.220806
C -4.839477 -3.361378 0.249932
C -4.080136 -4.520326 0.465453
C -5.686221 -3.321674 -0.865396
C -4.162680 -5.605213 -0.407433
H -3.418200 -4.571890 1.327467
C -5.773402 -4.404669 -1.742057
H -6.287274 -2.432610 -1.046449
C -5.010681 -5.551144 -1.515771
H -3.566418 -6.494651 -0.220178
H -6.439961 -4.353340 -2.599439
H -5.078240 -6.396848 -2.195089
H -5.627115 -1.560736 1.115786
Cl 0.302572 -0.549646 -0.079519
C 2.977589 2.114511 -2.392453
C 2.949828 0.574211 -2.410237
C 1.630096 -0.080283 -2.701960
C 1.556812 -1.504127 -3.200224
C 2.392689 2.809452 -1.151210
N 0.926684 2.779970 -0.972456
H 3.364029 0.188184 -1.463461
H 3.675084 0.235382 -3.169489
H 0.785440 0.554139 -2.967062
H 2.806397 2.361888 -0.239180
H 4.029364 2.427557 -2.448496
H 2.495665 2.510251 -3.294605
H 0.567320 -1.916138 -2.962355
S -0.006636 3.653447 -2.078188
O -0.471951 2.795442 -3.181827
O 0.749445 4.877424 -2.367250
C -1.445196 4.056624 -1.086130
C -2.699988 3.605995 -1.491621
C -1.302561 4.861494 0.047941
C -3.823775 3.959581 -0.742602
H -2.786604 2.990088 -2.380166
C -2.432926 5.202852 0.782864
H -0.319639 5.209532 0.347963
C -3.710863 4.761386 0.399369
H -4.803777 3.607982 -1.055368
H -2.326217 5.826704 1.667188
C -4.932246 5.168814 1.188492
H -5.237127 6.193347 0.938024
H -5.782845 4.512934 0.979154
H -4.740130 5.144485 2.266788

H 2.679874 3.863627 -1.158230
C 2.642260 -2.446714 -2.707610
C 3.799933 -2.663249 -3.467153
C 2.513439 -3.122414 -1.484944
C 4.809618 -3.515882 -3.015443
H 3.908607 -2.165711 -4.428944
C 3.520566 -3.975730 -1.029763
H 1.619826 -2.990589 -0.882785
C 4.674483 -4.173006 -1.791929
H 5.696993 -3.671022 -3.624054
H 3.392521 -4.489906 -0.080861
H 5.456151 -4.841337 -1.440142
H 0.555133 1.835281 -0.878372
H 1.596257 -1.474631 -4.305014

$(C_5\text{-Cl---N-rad})_{pic,XAT}$

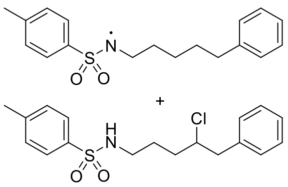

Name	E(B3LYP)	G(B3LYP)	E(RO-B2PLYP-D3)	G(RO-B2PLYP-D3)
Conformers				
Tosyl_NH_pentane_Ph_C5_Cl_Tosyl_N_radical_pentane_ separate_no_hydrogen_bond_0005_B2PLYP	-3065.151430	-3064.365334	-3064.103835	-3063.46475209
Tosyl_NH_pentane_Ph_C5_Cl_Tosyl_N_radical_pentane_ separate_no_hydrogen_bond_0012_B2PLYP	-3065.151430	-3064.365334	-3064.103833	-3063.46425364
Tosyl_NH_pentane_Ph_C5_Cl_Tosyl_N_radical_pentane_ separate_no_hydrogen_bond_0014_B2PLYP	-3065.151431	-3064.365368	-3064.103777	-3063.46395463
Tosyl_NH_pentane_Ph_C5_Cl_Tosyl_N_radical_pentane_ separate_no_hydrogen_bond_0010_B2PLYP	-3065.151431	-3064.365369	-3064.103783	-3063.46394556
Tosyl_NH_pentane_Ph_C5_Cl_Tosyl_N_radical_pentane_ separate_no_hydrogen_bond_0019_B2PLYP	-3065.151431	-3064.365336	-3064.103809	-3063.46379499
Tosyl_NH_pentane_Ph_C5_Cl_Tosyl_N_radical_pentane_ separate_no_hydrogen_bond_0002_B2PLYP	-3065.150804	-3064.364794	-3064.100992	-3063.46365277
Tosyl_NH_pentane_Ph_C5_Cl_Tosyl_N_radical_pentane_ separate_no_hydrogen_bond_0000_B2PLYP	-3065.150804	-3064.364795	-3064.100982	-3063.46355370
Tosyl_NH_pentane_Ph_C5_Cl_Tosyl_N_radical_pentane_ separate_no_hydrogen_bond_0006_B2PLYP	-3065.150804	-3064.364794	-3064.100985	-3063.46353652
Tosyl_NH_pentane_Ph_C5_Cl_Tosyl_N_radical_pentane_ separate_no_hydrogen_bond_0015_B2PLYP	-3065.150804	-3064.364794	-3064.100977	-3063.46351315
Tosyl_NH_pentane_Ph_C5_Cl_Tosyl_N_radical_pentane_ separate_no_hydrogen_bond_0007_B2PLYP	-3065.150804	-3064.364793	-3064.100979	-3063.46346121
Post-reactive complexes for XAT				
Tosyl_NH_pentane_Ph_C5_H_Tosyl_N_pentane_TS_md_ 00100_IRC_forward_opt_B2PLYP_pic	-3065.161523	-3064.375659	-3064.103816	-3063.46360205
Tosyl_NH_pentane_Ph_C5_H_Tosyl_N_pentane_TS_md_ 00030_IRC_reverse_opt_B2PLYP_pic	-3065.150360	-3064.364723	-3064.099979	-3063.46182485
Tosyl_NH_pentane_Ph_C5_H_Tosyl_N_pentane_TS_md_ 00016_IRC_forward_opt_B2PLYP_pic	-3065.154402	-3064.368519	-3064.096991	-3063.45904368
Tosyl_NH_pentane_Ph_C5_H_Tosyl_N_pentane_TS_md_ 00000_IRC_reverse_opt_B2PLYP_pic	-3065.152340	-3064.366273	-3064.091258	-3063.45790649
Tosyl_NH_pentane_Ph_C5_H_Tosyl_N_pentane_TS_md_ 00450_IRC_forward_opt_B2PLYP_pic	-3065.149099	-3064.363344	-3064.099826	-3063.45747405
Tosyl_NH_pentane_Ph_C5_H_Tosyl_N_pentane_TS_md_ 00200_IRC_forward_opt_B2PLYP_pic	-3065.149197	-3064.363583	-3064.097989	-3063.45711094
Tosyl_NH_pentane_Ph_C5_H_Tosyl_N_pentane_TS_md_ 00150_IRC_forward_opt_B2PLYP_pic	-3065.148509	-3064.362910	-3064.097121	-3063.45695091
Tosyl_NH_pentane_Ph_C5_H_Tosyl_N_pentane_TS_md_ 00080_IRC_forward_opt_B2PLYP_pic	-3065.146565	-3064.361005	-3064.093696	-3063.45596068
Tosyl_NH_pentane_Ph_C5_H_Tosyl_N_pentane_TS_md_ 00012_IRC_reverse_opt_B2PLYP_pic	-3065.146565	-3064.361005	-3064.093699	-3063.45588507
Tosyl_NH_pentane_Ph_C5_H_Tosyl_N_pentane_TS_md_ 00350_IRC_forward_opt_B2PLYP_pic	-3065.150173	-3064.364055	-3064.094875	-3063.45585861

89
-3063.46475209
C -5.158367 0.754479 -1.467161
C -5.320583 0.259278 -0.025815
C -6.011506 -1.106301 0.063606
C -6.143219 -1.648659 1.507398
C -4.620732 2.197177 -1.553170
N -3.238527 2.267955 -1.101714
H -5.901294 0.995163 0.549528
H -4.334150 0.216633 0.449138
H -5.468131 -1.839200 -0.548480
H -7.019424 -1.025937 -0.367903
H -4.590713 2.494851 -2.614135
H -4.489448 0.088731 -2.025233

H -6.128745 0.744277 -1.982841
H -6.578722 -0.865000 2.141637
S -2.968694 3.429898 0.111824
O -3.230120 2.730228 1.382139
O -3.674672 4.682290 -0.198025
C -1.215408 3.718986 -0.051595
C -0.309872 2.826814 0.526329
C -0.776111 4.841184 -0.755909
C 1.055262 3.061782 0.379033
H -0.663986 1.968479 1.086911
C 0.593009 5.061207 -0.888253
H -1.498398 5.528952 -1.182234
C 1.527107 4.179380 -0.326185
H 1.761856 2.363855 0.821172
H 0.941906 5.933446 -1.435469
C 3.007993 4.445263 -0.451796
H 3.246791 4.961253 -1.387322
H 3.363430 5.080948 0.370098
H 3.585203 3.515699 -0.418630
H -5.288982 2.899866 -1.039729
C -4.840536 -2.133492 2.111209
C -3.967561 -1.259860 2.777120
C -4.466873 -3.480688 1.997359
C -2.754357 -1.715275 3.298337
H -4.237509 -0.212712 2.890046
C -3.258172 -3.942725 2.522100
H -5.137586 -4.177484 1.498242
C -2.393967 -3.058426 3.171794
H -2.091669 -1.019700 3.805656
H -2.997037 -4.994536 2.432528
H -1.452984 -3.412765 3.583853
H -6.861176 -2.478265 1.502180
C 2.570656 -2.517416 -0.749184
C 1.346529 -1.660956 -1.096537
C 0.238325 -1.675721 -0.044908
C -1.068603 -0.989331 -0.478534
C 3.624656 -2.558015 -1.866877
N 4.306472 -1.292421 -2.150897
H 0.901985 -2.031664 -2.030531
H 1.636716 -0.616789 -1.276532
H 3.028063 -2.163171 0.180417
H 2.240857 -3.550366 -0.567698
H -0.856708 0.046690 -0.763695
S 5.792861 -0.882913 -1.505365
O 6.176934 0.343241 -2.208613
O 6.599405 -2.105180 -1.531779
C 5.575709 -0.441548 0.225061
C 5.296359 0.882937 0.567043
C 5.687015 -1.422616 1.212743
C 5.105363 1.216472 1.906527
H 5.265211 1.642369 -0.207655
C 5.489954 -1.072236 2.546994
H 5.950632 -2.438391 0.937427
C 5.187194 0.246250 2.915604
H 4.901810 2.250366 2.175176
H 5.583560 -1.834335 3.316802
C 4.945064 0.608623 4.361085
H 5.547897 -0.008266 5.035571
H 5.181879 1.659326 4.556931
H 3.892345 0.454262 4.632311
H 3.162068 -2.892590 -2.805009
C -1.750753 -1.717078 -1.621965
C -1.718251 -1.206413 -2.926890
C -2.425935 -2.925840 -1.393866
C -2.336689 -1.886212 -3.978860
H -1.214092 -0.261782 -3.116858
C -3.042408 -3.608344 -2.443472
H -2.483619 -3.325034 -0.383771
C -2.997978 -3.091770 -3.740522
H -2.302892 -1.471469 -4.982999
H -3.562927 -4.542154 -2.246969
H -3.480014 -3.622132 -4.557375
H 3.739740 -0.478262 -2.362204
Cl 0.843396 -0.853847 1.497430
H 4.408597 -3.282964 -1.632940
H 0.029042 -2.700672 0.273168
H -1.735656 -0.954719 0.389209
89
-3063.46425364
C -5.159849 0.750565 -1.468598
C -5.321887 0.256090 -0.026986
C -6.011773 -1.109969 0.063167
C -6.143466 -1.651418 1.507297
C -4.623367 2.193622 -1.555307
N -3.241298 2.265761 -1.103543
H -5.903215 0.991881 0.547849
H -4.335475 0.214467 0.448109
H -5.467639 -1.842859 -0.548256
H -7.019627 -1.030692 -0.368688
H -4.593317 2.490825 -2.616402
H -4.490304 0.085035 -2.026177
H -6.130138 0.739282 -1.984424
H -6.580017 -0.867747 2.140800
S -2.972815 3.429359 0.108680
O -3.236015 2.731580 1.379677
O -3.678237 4.681377 -0.203950
C -1.219280 3.718033 -0.052807
C -0.314668 2.826773 0.527812
C -0.778883 4.839087 -0.758356
C 1.050748 3.061380 0.382024
H -0.669614 1.969388 1.089319
C 0.590384 5.058759 -0.889162
H -1.500530 5.526221 -1.186783
C 1.523655 4.177719 -0.324344
H 1.756643 2.364091 0.826246
H 0.940122 5.930081 -1.437320
C 3.004688 4.443405 -0.448597
H 3.246156 4.946408 -1.390538
H 3.356605 5.091207 0.365303
H 3.582675 3.515028 -0.400313
H -5.292320 2.896030 -1.042390
C -4.840503 -2.134423 2.111955
C -4.465174 -3.481225 1.998936
C -3.968886 -1.259426 2.777844
C -3.256161 -3.941554 2.524460
H -5.134826 -4.179065 1.499852
C -2.755380 -1.713128 3.299855
H -4.240162 -0.212551 2.890128
C -2.393318 -3.055907 3.174128
H -2.993726 -4.993097 2.435531
H -2.093775 -1.016525 3.807176
H -1.452103 -3.408917 3.586792
H -6.860550 -2.481784 1.502418
C 2.571996 -2.516907 -0.746409
C 1.347863 -1.660655 -1.094236
C 0.239513 -1.675014 -0.042763
C -1.067337 -0.988750 -0.476857
C 3.625510 -2.558980 -1.864504
N 4.307395 -1.293844 -2.150404
H 0.903420 -2.031820 -2.028098
H 1.638034 -0.616563 -1.274684
H 3.029823 -2.161737 0.182633
H 2.242113 -3.549613 -0.563672
H -0.855308 0.047135 -0.762414
S 5.794276 -0.883880 -1.506350
O 6.178423 0.341052 -2.211662
O 6.600363 -2.106478 -1.531324
C 5.578245 -0.439775 0.223514
C 5.689052 -1.419499 1.212597
C 5.300265 0.885463 0.563664
C 5.492842 -1.066996 2.546404
H 5.951649 -2.435915 0.938679
C 5.110136 1.221133 1.902759
H 5.269494 1.643768 -0.212156
C 5.191462 0.252319 2.913211
H 5.586053 -1.828060 3.317285
H 4.907670 2.255605 2.169997
C 4.950346 0.616957 4.358297
H 3.898322 0.460837 4.631176
H 5.555352 0.002636 5.033204

H 5.185202 1.668521 4.551831
H 3.162447 -2.894635 -2.802014
C -1.749249 -1.716953 -1.620128
C -1.716696 -1.206700 -2.925209
C -2.424208 -2.925787 -1.391720
C -2.334851 -1.886974 -3.977035
H -1.212733 -0.262013 -3.115419
C -3.040390 -3.608766 -2.441179
H -2.481928 -3.324664 -0.381503
C -2.995901 -3.092601 -3.738392
H -2.301015 -1.472551 -4.981303
H -3.560730 -4.542626 -2.244443
H -3.477708 -3.623334 -4.555140
H 3.740706 -0.479833 -2.362449
Cl 0.844355 -0.852660 1.499430
H 4.409448 -3.283757 -1.630033
H 0.030156 -2.699849 0.275631
H -1.734544 -0.953743 0.390766
89
-3063.46395463
C -5.231412 0.662334 -1.449784
C -5.362301 0.150805 -0.010743
C -6.011526 -1.235340 0.073794
C -6.121985 -1.790292 1.514400
C -4.741828 2.121193 -1.529282
N -3.357435 2.231404 -1.090753
H -5.957660 0.864573 0.577255
H -4.369423 0.133010 0.452716
H -5.448923 -1.947886 -0.544921
H -7.023128 -1.182605 -0.353364
H -4.732805 2.431220 -2.587209
H -4.547177 0.022992 -2.020070
H -6.206599 0.623704 -1.955000
H -6.583665 -1.026080 2.153808
S -3.110360 3.435352 0.083788
O -3.425432 2.792669 1.371904
O -3.788060 4.681842 -0.303047
C -1.348543 3.695095 -0.028454
C -0.477598 2.809144 0.607889
C -0.867811 4.788886 -0.750814
C 0.895704 3.019769 0.501579
H -0.864612 1.973870 1.181396
C 0.507940 4.985481 -0.841101
H -1.564562 5.472957 -1.223099
C 1.408573 4.107746 -0.219893
H 1.575388 2.325132 0.988093
H 0.888835 5.835685 -1.401679
C 2.896793 4.347840 -0.304676
H 3.190254 4.710234 -1.295720
H 3.214312 5.105636 0.423737
H 3.460381 3.433509 -0.094704
H -5.427562 2.797242 -1.002923
C -4.802489 -2.232661 2.114108
C -4.376235 -3.562486 1.982410
C -3.965921 -1.335230 2.794759
C -3.151664 -3.984343 2.504162
H -5.018182 -4.277845 1.471852
C -2.737230 -1.750268 3.313398
H -4.277914 -0.301407 2.921868
C -2.324255 -3.076374 3.168951
H -2.849477 -5.023819 2.400750
H -2.103673 -1.037091 3.833450
H -1.371015 -3.399298 3.578506
H -6.810611 -2.644405 1.505053
C 2.618193 -2.462478 -0.762661
C 1.383473 -1.611559 -1.085324
C 0.279008 -1.663694 -0.030993
C -1.036390 -0.980796 -0.443976
C 3.657832 -2.483737 -1.894233
N 4.329234 -1.211402 -2.171624
H 0.939216 -1.965804 -2.025865
H 1.661651 -0.560473 -1.242438
H 3.084791 -2.115350 0.165124
H 2.298221 -3.499858 -0.588819
H -0.835707 0.064586 -0.701618
S 5.830157 -0.807972 -1.557928
O 6.200987 0.421818 -2.261800
O 6.633530 -2.031568 -1.609785
C 5.652717 -0.376815 0.179568
C 5.764692 -1.367939 1.157310
C 5.405084 0.949766 0.537133
C 5.599326 -1.025177 2.497670
H 6.004950 -2.386046 0.869450
C 5.246257 1.275575 1.882939
H 5.372279 1.715836 -0.230848
C 5.328694 0.295830 2.882456
H 5.693186 -1.795268 3.259470
H 5.067740 2.310893 2.163653
C 5.121529 0.650170 4.335380
H 5.739916 0.028994 4.991713
H 5.363970 1.699492 4.531484
H 4.075613 0.494631 4.631209
H 3.184531 -2.810198 -2.829942
C -1.712848 -1.684783 -1.605563
C -1.695920 -1.133954 -2.894260
C -2.367311 -2.910703 -1.411149
C -2.309267 -1.791241 -3.963380
H -1.208952 -0.175476 -3.057548
C -2.978666 -3.570765 -2.477959
H -2.413404 -3.341179 -0.413375
C -2.949765 -3.014129 -3.758782
H -2.287798 -1.345451 -4.954466
H -3.482958 -4.518510 -2.307561
H -3.427708 -3.527066 -4.589058
H 3.757666 -0.396590 -2.366265
Cl 0.879761 -0.874669 1.530178
H 4.448585 -3.207076 -1.679157
H 0.081601 -2.698450 0.261940
H -1.702166 -0.977005 0.425518
89
-3063.46394556
C -5.233620 0.659805 -1.446194
C -5.362763 0.147069 -0.007397
C -6.010844 -1.239657 0.076550
C -6.119541 -1.796099 1.516704
C -4.745661 2.119147 -1.525064
N -3.360993 2.230468 -1.087596
H -5.958155 0.859899 0.581703
H -4.369428 0.129676 0.455141
H -5.448301 -1.951197 -0.543377
H -7.022881 -1.187283 -0.349617
H -4.737899 2.430157 -2.582732
H -4.549202 0.021577 -2.017506
H -6.209210 0.620411 -1.950576
H -6.581217 -1.032898 2.157314
S -3.113887 3.436886 0.084322
O -3.430435 2.797344 1.373637
O -3.790455 4.682910 -0.305935
C -1.351813 3.695199 -0.027080
C -0.481956 2.809392 0.610973
C -0.869775 4.787748 -0.750418
C 0.891562 3.018916 0.505434
H -0.870006 1.975051 1.185141
C 0.506193 4.983280 -0.839906
H -1.565707 5.471706 -1.224069
C 1.405733 4.105688 -0.216943
H 1.570412 2.324362 0.993220
H 0.888099 5.832530 -1.401238
C 2.894191 4.344629 -0.300777
H 3.188606 4.706614 -1.291685
H 3.211775 5.102323 0.427718
H 3.456926 3.429910 -0.090199
H -5.431670 2.794098 -0.997634
C -4.799128 -2.238008 2.114705
C -4.371291 -3.567038 1.980119
C -3.963273 -1.340870 2.796586
C -3.145876 -3.988371 2.500292
H -5.012670 -4.282197 1.468568
C -2.733744 -1.755372 3.313672
H -4.276557 -0.307712 2.925930

C -2.319203 -3.080668 3.166369
H -2.842441 -5.027260 2.394667
H -2.100758 -1.042430 3.834735
H -1.365311 -3.403200 3.574706
H -6.807500 -2.650750 1.507139
C 2.618518 -2.460873 -0.764437
C 1.383771 -1.609695 -1.086318
C 0.279106 -1.663233 -0.032248
C -1.036210 -0.979827 -0.444666
C 3.658175 -2.481044 -1.896002
N 4.329454 -1.208398 -2.172236
H 0.939720 -1.962853 -2.027363
H 1.661875 -0.558392 -1.242104
H 3.085093 -2.114586 0.163675
H 2.298586 -3.498429 -0.591563
H -0.835492 0.065934 -0.700717
S 5.830568 -0.805511 -1.558720
O 6.201477 0.424561 -2.262048
O 6.633725 -2.029214 -1.611342
C 5.653604 -0.375153 0.179018
C 5.764906 -1.366879 1.156231
C 5.407079 0.951433 0.537310
C 5.599966 -1.024697 2.496784
H 6.004338 -2.385029 0.867827
C 5.248676 1.276659 1.883314
H 5.374782 1.717928 -0.230264
C 5.330452 0.296329 2.882302
H 5.693294 -1.795273 3.258160
H 5.071025 2.311971 2.164589
C 5.123788 0.650037 4.335451
H 5.743067 0.029169 4.991242
H 5.365456 1.699494 4.531754
H 4.078179 0.493490 4.631831
H 3.184926 -2.806740 -2.832004
C -1.712163 -1.682176 -1.607529
C -1.695457 -1.129092 -2.895254
C -2.365959 -2.908805 -1.415294
C -2.308362 -1.784860 -3.965561
H -1.209024 -0.170056 -3.056828
C -2.976858 -3.567348 -2.483294
H -2.411895 -3.341037 -0.418270
C -2.948178 -3.008462 -3.763145
H -2.287080 -1.337323 -4.955862
H -3.480619 -4.515679 -2.314597
H -3.425770 -3.520224 -4.594346
H 3.757873 -0.393464 -2.366300
Cl 0.879501 -0.876236 1.530026
H 4.449009 -3.204472 -1.681533
H 0.081683 -2.698385 0.259267
H -1.702344 -0.977321 0.424569
89
-3063.46379499
C -5.164341 0.743414 -1.469855
C -5.325103 0.249744 -0.027811
C -6.012460 -1.117497 0.063559
C -6.143332 -1.657910 1.508149
C -4.630612 2.187374 -1.557802
N -3.248632 2.262343 -1.106156
H -5.907580 0.984928 0.546631
H -4.338476 0.210243 0.447024
H -5.466913 -1.849930 -0.547150
H -7.020425 -1.040487 -0.368453
H -4.601206 2.483858 -2.619118
H -4.493639 0.078730 -2.027057
H -6.134726 0.729837 -1.985449
H -6.581714 -0.874603 2.140838
S -2.982092 3.429130 0.103281
O -3.248861 2.735369 1.375735
O -3.685888 4.680661 -0.214880
C -1.227957 3.716064 -0.054736
C -0.325552 2.826061 0.530928
C -0.784901 4.834673 -0.762731
C 1.040519 3.059236 0.387851
H -0.682539 1.970695 1.094210
C 0.584737 5.052947 -0.890727
H -1.504991 5.520982 -1.195085
C 1.515991 4.172876 -0.320747
H 1.744768 2.362825 0.835953
H 0.936487 5.922341 -1.440699
C 2.997427 4.437345 -0.442786
H 3.576594 3.512346 -0.358933
H 3.244966 4.908864 -1.399451
H 3.341953 5.113407 0.351017
H -5.300889 2.888933 -1.045430
C -4.839506 -2.137607 2.113595
C -4.461256 -3.483693 2.001861
C -3.969982 -1.260175 2.779026
C -3.251424 -3.940983 2.528165
H -5.129252 -4.183403 1.503176
C -2.755683 -1.710830 3.301817
H -4.243583 -0.213802 2.890361
C -2.390690 -3.052937 3.177348
H -2.986712 -4.992037 2.440218
H -2.095755 -1.012403 3.808829
H -1.448842 -3.403556 3.590603
H -6.858652 -2.489799 1.503834
C 2.574740 -2.515610 -0.742214
C 1.350355 -1.659860 -1.090394
C 0.241939 -1.674258 -0.038993
C -1.065065 -0.988438 -0.473283
C 3.627348 -2.559505 -1.861091
N 4.309334 -1.295006 -2.149327
H 0.906016 -2.031592 -2.024086
H 1.640219 -0.615768 -1.271311
H 3.033328 -2.159033 0.185921
H 2.244964 -3.548027 -0.557648
H -0.853374 0.047550 -0.758703
S 5.797480 -0.884869 -1.508485
O 6.182175 0.337631 -2.217707
O 6.602129 -2.108470 -1.531023
C 5.584161 -0.435559 0.220373
C 5.692999 -1.412966 1.211976
C 5.310452 0.891406 0.557224
C 5.498954 -1.056365 2.545001
H 5.952475 -2.430867 0.940599
C 5.122541 1.231179 1.895614
H 5.281125 1.647671 -0.220641
C 5.201809 0.264817 2.908563
H 5.590587 -1.815653 3.317818
H 4.923520 2.266980 2.160305
C 4.962994 0.633998 4.352878
H 5.201623 1.685235 4.543552
H 3.910624 0.482227 4.626905
H 5.566328 0.019314 5.028943
H 3.163430 -2.896445 -2.797726
C -1.746541 -1.716768 -1.616752
C -2.420738 -2.926085 -1.388677
C -1.714379 -1.206088 -2.921668
C -3.036560 -3.609131 -2.438312
H -2.478200 -3.325298 -0.378577
C -2.332169 -1.886425 -3.973671
H -1.211059 -0.261008 -3.111608
C -2.992461 -3.092539 -3.735369
H -3.556308 -4.543375 -2.241828
H -2.298645 -1.471668 -4.977812
H -3.473975 -3.623322 -4.552258
H 3.743043 -0.481174 -2.362992
Cl 0.846392 -0.851597 1.503208
H 4.411294 -3.284126 -1.626190
H 0.032826 -2.699109 0.279520
H -1.732455 -0.953815 0.394221
89
-3063.46365277
C -5.525848 -0.953988 -0.857475
C -5.211359 -1.349936 0.589428
C -5.252832 -2.864850 0.819287
C -4.828144 -3.292477 2.244313
C -5.664591 0.568703 -1.045844
N -4.389240 1.236759 -0.833785
H -5.927932 -0.863446 1.267051

H -4.226101 -0.952620 0.858604
H -4.608941 -3.370588 0.086002
H -6.273852 -3.228382 0.636306
H -5.924142 0.770511 -2.098278
H -4.745265 -1.330519 -1.529739
H -6.474412 -1.406108 -1.179009
H -5.400893 -2.707142 2.975626
S -4.462479 2.527581 0.272378
O -4.189517 1.913806 1.584379
O -5.680418 3.328049 0.075443
C -3.058729 3.506836 -0.228960
C -1.768731 3.084949 0.101515
C -3.273695 4.686318 -0.942573
C -0.683656 3.855985 -0.304308
H -1.613927 2.173995 0.669403
C -2.173872 5.447506 -1.334519
H -4.285496 4.998681 -1.177111
C -0.867211 5.047013 -1.024205
H 0.320899 3.522765 -0.057209
H -2.334304 6.368109 -1.890058
C 0.317715 5.890598 -1.429368
H 1.181391 5.269211 -1.688807
H 0.083491 6.526348 -2.289001
H 0.627859 6.550748 -0.608517
H -6.480176 0.971125 -0.431720
C -3.344761 -3.144205 2.516737
C -2.466378 -4.203393 2.241878
C -2.805907 -1.952695 3.024923
C -1.093712 -4.079413 2.463804
H -2.866574 -5.141043 1.860714
C -1.433288 -1.821141 3.244261
H -3.465779 -1.118851 3.251656
C -0.571675 -2.884805 2.966148
H -0.434809 -4.918622 2.254904
H -1.037356 -0.888142 3.635414
H 0.494846 -2.786306 3.151086
H -5.113992 -4.341338 2.392784
C 3.020270 -1.973563 -0.165747
C 1.923045 -1.384747 -1.061423
C 0.646133 -0.977101 -0.328144
C -0.513476 -0.570313 -1.253140
C 4.240530 -2.479034 -0.951916
N 5.020852 -1.454679 -1.650954
H 1.630393 -2.133707 -1.810368
H 2.297164 -0.512730 -1.615294
H 3.336731 -1.236442 0.579487
H 2.605767 -2.828165 0.387800
H -0.187851 0.260777 -1.889106
S 6.397889 -0.754372 -1.010252
O 6.928924 0.072566 -2.096168
O 7.158225 -1.841637 -0.389584
C 5.919087 0.364488 0.314159
C 5.917083 -0.086505 1.635675
C 5.551709 1.676768 0.008664
C 5.523827 0.782776 2.651628
H 6.245060 -1.095790 1.860676
C 5.162726 2.531971 1.036933
H 5.599182 2.026693 -1.017478
C 5.133030 2.099343 2.370568
H 5.530222 0.435249 3.681890
H 4.886420 3.556987 0.801596
C 4.678461 3.023884 3.474365
H 5.168743 2.786411 4.424053
H 4.890395 4.070813 3.233960
H 3.595751 2.938431 3.636270
H 3.921416 -3.211712 -1.705223
C -1.005237 -1.723011 -2.109257
C -0.800692 -1.732403 -3.494898
C -1.675220 -2.809264 -1.525069
C -1.252399 -2.794977 -4.280602
H -0.289321 -0.895055 -3.964595
C -2.123881 -3.874121 -2.307177
H -1.849212 -2.823036 -0.451482
C -1.914502 -3.870642 -3.688340
H -1.087318 -2.779790 -5.354757
H -2.638940 -4.707465 -1.836160
H -2.266437 -4.699035 -4.297228
H 4.531903 -0.829454 -2.282476
Cl 1.011298 0.438016 0.804915
H 4.938314 -2.999647 -0.290742
H 0.322579 -1.770665 0.350355
H -1.336066 -0.192823 -0.636171
89
-3063.46355370
C -5.522956 -0.956314 -0.856446
C -5.209634 -1.350519 0.591189
C -5.251150 -2.865179 0.822770
C -4.826203 -3.291150 2.248213
C -5.661915 0.566104 -1.046765
N -4.387143 1.234889 -0.833492
H -5.926757 -0.863294 1.267696
H -4.224610 -0.952817 0.860676
H -4.607347 -3.371776 0.090000
H -6.272191 -3.228901 0.640300
H -5.919939 0.766619 -2.099825
H -4.741681 -1.333464 -1.527560
H -6.471113 -1.409041 -1.178328
H -5.398742 -2.704933 2.978978
S -4.462652 2.527175 0.270845
O -4.190458 1.915319 1.583892
O -5.681086 3.326347 0.071721
C -3.059282 3.506932 -0.230550
C -1.769016 3.085329 0.099602
C -3.274747 4.686548 -0.943640
C -0.684330 3.856837 -0.306070
H -1.613809 2.174204 0.667110
C -2.175169 5.448320 -1.335444
H -4.286684 4.998651 -1.177933
C -0.868409 5.048152 -1.025543
H 0.320386 3.523865 -0.059239
H -2.335997 6.369117 -1.890525
C 0.316528 5.891670 -1.430829
H 0.076444 6.541587 -2.278102
H 0.639476 6.537295 -0.603418
H 1.173942 5.269689 -1.709229
H -6.478569 0.968974 -0.434359
C -3.342734 -3.142527 2.519988
C -2.464210 -4.201477 2.244621
C -2.803947 -1.950904 3.027944
C -1.091471 -4.077136 2.465859
H -2.864355 -5.139217 1.863621
C -1.431249 -1.818987 3.246601
H -3.463949 -1.117246 3.254995
C -0.569499 -2.882404 2.968003
H -0.432443 -4.916158 2.256598
H -1.035367 -0.885886 3.637559
H 0.497092 -2.783635 3.152395
H -5.112021 -4.339835 2.397997
C 3.019500 -1.973428 -0.164258
C 1.922855 -1.385865 -1.061480
C 0.645248 -0.977709 -0.329682
C -0.513708 -0.572288 -1.256103
C 4.240432 -2.479671 -0.948888
N 5.021098 -1.456035 -1.648621
H 1.630992 -2.135726 -1.809832
H 2.297220 -0.514377 -1.616016
H 3.335289 -1.235350 0.580314
H 2.604706 -2.827415 0.390022
H -0.187735 0.258115 -1.892792
S 6.397488 -0.754623 -1.007735
O 6.929275 0.070947 -2.094320
O 7.157492 -1.840922 -0.384975
C 5.917411 0.365945 0.314779
C 5.914470 -0.083272 1.636922
C 5.550031 1.677724 0.007232
C 5.520278 0.787292 2.651384
H 6.242461 -1.092194 1.863526
C 5.160084 2.534252 1.034065
H 5.598220 2.026287 -1.019341
C 5.129452 2.103429 2.368239

H 5.525946 0.441156 3.682123
H 4.883747 3.558889 0.797129
C 4.673946 3.029299 3.470543
H 3.591923 2.940324 3.635118
H 5.167168 2.796140 4.419795
H 4.881586 4.076325 3.226884
H 3.922007 -3.213322 -1.701538
C -1.004422 -1.726141 -2.111276
C -0.799159 -1.736891 -3.496798
C -1.674124 -2.812153 -1.526289
C -1.249887 -2.800543 -4.281613
H -0.287981 -0.899772 -3.967116
C -2.121795 -3.878083 -2.307499
H -1.848625 -2.824902 -0.452766
C -1.911703 -3.875946 -3.688559
H -1.084255 -2.786402 -5.355698
H -2.636650 -4.711215 -1.835881
H -2.262865 -4.705178 -4.296752
H 4.532492 -0.831733 -2.281323
Cl 1.009201 0.438757 0.802078
H 4.937815 -2.999341 -0.286554
H 0.321385 -1.770586 0.349471
H -1.336953 -0.194227 -0.640345
89
-3063.46353652
C -5.525111 -0.958611 -0.855500
C -5.209746 -1.353699 0.591446
C -5.249573 -2.868559 0.821951
C -4.823541 -3.295085 2.246904
C -5.665387 0.563882 -1.044349
N -4.390697 1.233261 -0.832529
H -5.926572 -0.867675 1.269136
H -4.224799 -0.955269 0.860117
H -4.605609 -3.373970 0.088503
H -6.270330 -3.233207 0.639738
H -5.925189 0.765105 -2.096835
H -4.744365 -1.334617 -1.527869
H -6.473330 -1.411771 -1.176586
H -5.396433 -2.710008 2.978309
S -4.465105 2.524365 0.273300
O -4.190476 1.911328 1.585292
O -5.684276 3.323081 0.076913
C -3.063039 3.505397 -0.229255
C -1.772207 3.085052 0.099957
C -3.280133 4.684699 -0.942514
C -0.688472 3.857471 -0.306794
H -1.615736 2.174210 0.667573
C -2.181614 5.447301 -1.335386
H -4.292539 4.995824 -1.176083
C -0.874174 5.048374 -1.026359
H 0.316728 3.525442 -0.060719
H -2.343703 6.367776 -1.890656
C 0.309349 5.893440 -1.432545
H 0.619918 6.553362 -0.611672
H 1.173314 5.273101 -1.693538
H 0.073334 6.529506 -2.291452
H -6.481336 0.965704 -0.430316
C -3.340132 -3.145079 2.518259
C -2.460730 -4.203274 2.242809
C -2.802285 -1.952922 3.025985
C -1.088056 -4.077717 2.463765
H -2.860123 -5.141396 1.861964
C -1.429660 -1.819789 3.244352
H -3.462946 -1.119806 3.253097
C -0.567034 -2.882489 2.965702
H -0.428340 -4.916177 2.254433
H -1.034521 -0.886311 3.635167
H 0.499502 -2.782778 3.149911
H -5.108170 -4.344180 2.396064
C 3.020610 -1.971647 -0.163910
C 1.924368 -1.384194 -1.061694
C 0.646791 -0.975074 -0.330396
C -0.511794 -0.569780 -1.257335
C 4.241554 -2.478711 -0.947989
N 5.022748 -1.455728 -1.648089
H 1.632318 -2.134401 -1.809631
H 2.299196 -0.513212 -1.616715
H 3.336456 -1.233285 0.580358
H 2.605407 -2.825209 0.390718
H -0.185221 0.259866 -1.894708
S 6.399149 -0.754350 -1.007175
O 6.931424 0.070562 -2.094018
O 7.158723 -1.840538 -0.383702
C 5.919022 0.366969 0.314679
C 5.915774 -0.081563 1.637038
C 5.551902 1.678660 0.006386
C 5.521537 0.789595 2.650991
H 6.243556 -1.090422 1.864224
C 5.161914 2.535770 1.032696
H 5.600326 2.026675 -1.020360
C 5.130976 2.105634 2.367101
H 5.526958 0.443988 3.681906
H 4.885780 3.560330 0.795184
C 4.675409 3.032211 3.468782
H 4.886152 4.078817 3.225947
H 3.592812 2.945889 3.630975
H 5.166025 2.797492 4.418989
H 3.923037 -3.212634 -1.700334
C -1.003108 -1.724086 -2.111549
C -0.797864 -1.736098 -3.497060
C -1.673440 -2.809215 -1.525656
C -1.249243 -2.800125 -4.280994
H -0.286194 -0.899672 -3.968076
C -2.121762 -3.875515 -2.305978
H -1.847943 -2.820978 -0.452128
C -1.911700 -3.874638 -3.687046
H -1.083617 -2.786967 -5.355092
H -2.637082 -4.707958 -1.833650
H -2.263372 -4.704158 -4.294550
H 4.534485 -0.831595 -2.281225
Cl 1.011022 0.441942 0.800555
H 4.938600 -2.998291 -0.285228
H 0.322476 -1.767420 0.349158
H -1.334894 -0.190697 -0.642013
89
-3063.46351315
C -5.524557 -0.960384 -0.855339
C -5.209556 -1.354598 0.591919
C -5.248706 -2.869361 0.823153
C -4.822620 -3.294982 2.248361
C -5.665260 0.561962 -1.045065
N -4.390912 1.231892 -0.832857
H -5.926861 -0.868585 1.269111
H -4.224902 -0.955556 0.860753
H -4.604392 -3.374821 0.090045
H -6.269258 -3.234602 0.640973
H -5.924512 0.762531 -2.097808
H -4.743450 -1.336500 -1.527230
H -6.472512 -1.414019 -1.176533
H -5.395906 -2.709872 2.979430
S -4.466292 2.523384 0.272417
O -4.191879 1.910968 1.584748
O -5.685735 3.321515 0.075302
C -3.064474 3.504805 -0.230066
C -1.773525 3.084698 0.098962
C -3.281894 4.684176 -0.943131
C -0.689993 3.857429 -0.307764
H -1.616781 2.173787 0.666394
C -2.183588 5.447091 -1.335961
H -4.294393 4.995073 -1.176601
C -0.876024 5.048407 -1.027105
H 0.315303 3.525571 -0.061862
H -2.345935 6.367612 -1.891084
C 0.307230 5.893877 -1.433233
H 1.172196 5.273924 -1.691777
H 0.071796 6.528051 -2.293707
H 0.615913 6.555745 -0.613211
H -6.481687 0.963839 -0.431707
C -3.339316 -3.144011 2.519764
C -2.459259 -4.201749 2.244674

C -2.802218 -1.951360 3.027133
C -1.086662 -4.075270 2.465619
H -2.858059 -5.140247 1.864131
C -1.429684 -1.817310 3.245485
H -3.463402 -1.118576 3.253943
C -0.566389 -2.879561 2.967184
H -0.426428 -4.913397 2.256578
H -1.035131 -0.883467 3.636023
H 0.500083 -2.779112 3.151356
H -5.106689 -4.344164 2.397984
C 3.020967 -1.971289 -0.163060
C 1.924919 -1.384535 -1.061533
C 0.647063 -0.975155 -0.330847
C -0.511318 -0.570623 -1.258363
C 4.242166 -2.478774 -0.946480
N 5.023422 -1.456198 -1.647089
H 1.633207 -2.135230 -1.809110
H 2.299779 -0.513825 -1.616955
H 3.336558 -1.232399 0.580794
H 2.605691 -2.824516 0.392027
H -0.184689 0.258688 -1.896143
S 6.399592 -0.754237 -1.006295
O 6.932003 0.070056 -2.093544
O 7.159157 -1.839942 -0.381963
C 5.919041 0.367863 0.314747
C 5.915656 -0.079806 1.637396
C 5.551831 1.679320 0.005546
C 5.521202 0.791981 2.650731
H 6.243508 -1.088484 1.865286
C 5.161628 2.537062 1.031242
H 5.600356 2.026668 -1.021422
C 5.130565 2.107795 2.365930
H 5.526520 0.447048 3.681871
H 4.885433 3.561442 0.793025
C 4.674779 3.035074 3.466931
H 5.164741 2.800572 4.417526
H 4.886124 4.081479 3.223741
H 3.592048 2.949319 3.628533
H 3.923912 -3.213260 -1.698389
C -1.002178 -1.725529 -2.112033
C -0.796678 -1.738243 -3.497499
C -1.672343 -2.810514 -1.525675
C -1.247641 -2.802811 -4.280940
H -0.285132 -0.901944 -3.968876
C -2.120246 -3.877353 -2.305502
H -1.847037 -2.821746 -0.452170
C -1.909929 -3.877172 -3.686532
H -1.081816 -2.790193 -5.355014
H -2.635444 -4.709673 -1.832823
H -2.261274 -4.707114 -4.293649
H 4.535236 -0.832494 -2.280709
Cl 1.010795 0.442636 0.799309
H 4.939091 -2.997830 -0.283180
H 0.322689 -1.767110 0.349135
H -1.334671 -0.191291 -0.643529
89
-3063.46346121
C -5.524721 -0.963311 -0.854004
C -5.208333 -1.357519 0.592955
C -5.246235 -2.872345 0.824003
C -4.819144 -3.297833 2.248950
C -5.666871 0.558947 -1.043336
N -4.392915 1.229939 -0.832152
H -5.925489 -0.872130 1.270749
H -4.223771 -0.957810 0.861133
H -4.601924 -3.377241 0.090502
H -6.266611 -3.238312 0.642294
H -5.927238 0.759490 -2.095810
H -4.743800 -1.338638 -1.526550
H -6.472532 -1.417696 -1.174567
H -5.392555 -2.713269 2.980356
S -4.468486 2.521034 0.273566
O -4.192832 1.908431 1.585555
O -5.688619 3.318337 0.077420
C -3.067668 3.503607 -0.229447
C -1.776232 3.084526 0.099061
C -3.286326 4.682827 -0.942341
C -0.693503 3.858142 -0.308040
H -1.618550 2.173725 0.666411
C -2.188773 5.446661 -1.335569
H -4.299164 4.992949 -1.175366
C -0.880787 5.049020 -1.027269
H 0.312160 3.527093 -0.062535
H -2.352081 6.367094 -1.890550
C 0.301729 5.895286 -1.433890
H 1.165149 5.275704 -1.698506
H 0.063716 6.533601 -2.290565
H 0.614157 6.552999 -0.611955
H -6.483106 0.959990 -0.429176
C -3.335838 -3.145749 2.519724
C -2.799442 -1.952786 3.027093
C -2.455087 -4.202770 2.244081
C -1.426922 -1.817745 3.244943
H -3.461162 -1.120542 3.254316
C -1.082507 -4.075301 2.464519
H -2.853330 -5.141492 1.863508
C -0.562941 -2.879297 2.966120
H -1.032924 -0.883679 3.635508
H -0.421720 -4.912887 2.255058
H 0.503524 -2.778096 3.149923
H -5.102326 -4.347255 2.398561
C 3.021310 -1.969588 -0.162960
C 1.925612 -1.383170 -1.062073
C 0.647627 -0.973189 -0.331960
C -0.510458 -0.569039 -1.260018
C 4.242703 -2.477610 -0.945722
N 5.024245 -1.455470 -1.646654
H 1.633978 -2.134244 -1.809301
H 2.300770 -0.512838 -1.617886
H 3.336740 -1.230350 0.580616
H 2.605750 -2.822482 0.392428
H -0.183526 0.259796 -1.898259
S 6.400353 -0.753337 -1.005921
O 6.932879 0.070617 -2.093372
O 7.159855 -1.838840 -0.381171
C 5.919612 0.369135 0.314725
C 5.915824 -0.078221 1.637489
C 5.552516 1.680513 0.005101
C 5.521063 0.793803 2.650485
H 6.243622 -1.086836 1.865733
C 5.162003 2.538510 1.030479
H 5.601344 2.027596 -1.021942
C 5.130506 2.109559 2.365243
H 5.526057 0.449121 3.681712
H 4.885886 3.562832 0.791926
C 4.674348 3.037056 3.465908
H 3.591809 2.950207 3.628204
H 5.165114 2.803692 4.416374
H 4.884418 4.083527 3.221925
H 3.924630 -3.212496 -1.697316
C -1.001318 -1.724415 -2.113052
C -0.795653 -1.737988 -3.498484
C -1.671660 -2.808977 -1.526119
C -1.246646 -2.802976 -4.281337
H -0.283960 -0.902030 -3.970307
C -2.119591 -3.876237 -2.305353
H -1.846453 -2.819554 -0.452625
C -1.909124 -3.876904 -3.686359
H -1.080698 -2.791021 -5.355399
H -2.634918 -4.708221 -1.832224
H -2.260494 -4.707170 -4.293019
H 4.536287 -0.832031 -2.280709
Cl 1.011226 0.445212 0.797475
H 4.939384 -2.996350 -0.281916
H 0.322981 -1.764717 0.348389
H -1.333892 -0.189164 -0.645624

89

-3063.46360205

C -0.552745 -0.917258 -3.588814
C 0.432513 -2.095994 -3.643223
C 0.853898 -2.653708 -2.279037
C 1.917980 -3.766418 -2.381758
C -1.916080 -1.234001 -2.945129
N -1.820309 -1.259505 -1.496554
H -0.006901 -2.905963 -4.243877
H 1.326228 -1.763040 -4.190079
H -0.022361 -3.061653 -1.761196
H 1.239860 -1.843931 -1.645334
H -2.594724 -0.392917 -3.164804
H -0.753831 -0.573522 -4.611034
H -0.110017 -0.066368 -3.057469
H 1.555677 -4.546203 -3.064498
S -2.899082 -2.332472 -0.726583
O -2.785428 -3.671700 -1.320476
O -4.198732 -1.638425 -0.696960
C -2.223639 -2.376392 0.924873
C -1.281514 -3.353927 1.252310
C -2.635370 -1.424402 1.859423
C -0.738292 -3.364953 2.534201
H -0.975883 -4.091397 0.518438
C -2.084217 -1.457581 3.139140
H -3.370042 -0.674199 1.588545
C -1.131104 -2.422757 3.496113
H 0.008343 -4.114370 2.783247
H -2.402438 -0.721951 3.873668
C -0.557713 -2.465374 4.892195
H -1.090030 -3.198870 5.512066
H 0.497986 -2.756325 4.881548
H -0.640443 -1.494550 5.390886
H -2.355258 -2.151210 -3.358653
C 2.244021 -4.382016 -1.037366
C 1.595010 -5.547060 -0.604392
C 3.169729 -3.778486 -0.174423
C 1.860338 -6.094011 0.653144
H 0.879709 -6.034741 -1.263645
C 3.438664 -4.320241 1.082954
H 3.685325 -2.874541 -0.491893
C 2.784524 -5.481125 1.502256
H 1.353668 -7.004527 0.963481
H 4.164514 -3.838261 1.732981
H 3.002038 -5.911064 2.476708
H 2.828581 -3.351347 -2.834006
Cl 3.911599 0.645019 -0.130002
C 0.895635 1.891758 0.118468
C 2.031170 2.275695 1.073460
C 3.419058 2.329440 0.432455
C 4.493202 2.888937 1.378958
C -0.466857 1.871088 0.824426
N -1.586796 1.473742 -0.036256
H 2.061783 1.589492 1.930593
H 1.832486 3.278444 1.480580
H 3.390307 2.917214 -0.489317
H -0.449682 1.163203 1.662737
H 0.848853 2.606640 -0.711541
H 1.092577 0.902309 -0.311246
H 4.093257 3.834942 1.770706
S -2.317717 2.594955 -1.045939
O -2.540850 1.910765 -2.329040
O -1.574321 3.860721 -0.977084
C -3.923579 2.880399 -0.297239
C -4.864919 1.845707 -0.265162
C -4.220785 4.137605 0.222615
C -6.109664 2.085848 0.307056
H -4.632583 0.867890 -0.676268
C -5.477385 4.358964 0.790297
H -3.478514 4.926970 0.175957
C -6.437940 3.343176 0.841762
H -6.842363 1.282775 0.336048
H -5.712604 5.339849 1.196237
C -7.803101 3.588033 1.439619
H -8.075338 2.799383 2.150728
H -7.845196 4.547014 1.965107
H -8.578129 3.600826 0.662538
H -0.696849 2.857029 1.239195
C 5.841857 3.145943 0.740709
C 6.913295 2.264854 0.928484
C 6.040740 4.279923 -0.059070
C 8.150541 2.506068 0.329214
H 6.774756 1.381002 1.545818
C 7.274467 4.523739 -0.662190
H 5.222853 4.982962 -0.206476
C 8.334602 3.635707 -0.469084
H 8.970666 1.810546 0.487135
H 7.409563 5.409635 -1.277206
H 9.297984 3.825471 -0.934745
H -1.481236 0.577442 -0.520925
H 4.595356 2.211243 2.234335
89
-3063.46182485
C 1.925391 -2.274955 -1.873717
C 0.843164 -1.447615 -2.588111
C -0.508993 -2.157995 -2.747921
C -1.550733 -1.298267 -3.492563
C 1.604530 -2.538847 -0.400478
N 2.669474 -3.292292 0.229864
H 0.689594 -0.499907 -2.050892
H 1.220236 -1.173607 -3.582920
H -0.922423 -2.422460 -1.765703
H -0.366099 -3.105946 -3.285979
H 1.464062 -1.584152 0.136039
H 2.887662 -1.752084 -1.923603
H 2.063153 -3.237200 -2.383157
H -1.682714 -0.356931 -2.944982
S 2.492479 -3.739467 1.853297
O 3.561815 -3.035837 2.569844
O 2.422677 -5.205407 1.831402
C 0.924595 -3.138404 2.496115
C 0.881496 -1.929431 3.191247
C -0.235901 -3.892699 2.297424
C -0.344613 -1.471113 3.678851
H 1.799185 -1.377518 3.367539
C -1.450885 -3.412981 2.777894
H -0.177630 -4.850209 1.789749
C -1.529311 -2.193496 3.468512
H -0.377927 -0.547921 4.254130
H -2.354527 -3.996113 2.620684
C -2.860817 -1.669722 3.948093
H -3.402683 -1.200223 3.117209
H -3.489867 -2.476984 4.338115
H -2.737752 -0.921527 4.737836
H 0.649256 -3.076834 -0.291281
C -2.887116 -1.994999 -3.641697
C -3.206209 -2.703246 -4.807837
C -3.823745 -1.974993 -2.597221
C -4.423688 -3.374930 -4.932228
H -2.493078 -2.724784 -5.629831
C -5.041506 -2.645754 -2.717908
H -3.598728 -1.427630 -1.684252
C -5.346565 -3.348582 -3.885608
H -4.651854 -3.915357 -5.847720
H -5.755787 -2.614534 -1.898708
H -6.296710 -3.868126 -3.980342
H -1.156491 -1.039276 -4.484690
Cl 3.929497 0.716824 0.792447
C 0.888525 1.778174 0.851668
C 2.067817 2.756812 0.852152
C 3.291682 2.279844 0.066385
C 4.414664 3.329417 0.023274
C -0.334391 2.341748 1.584849
N -1.460060 1.399376 1.717152
H 2.371921 2.988131 1.881990
H 1.751331 3.707169 0.396595
H 3.001544 2.004184 -0.951608
H -0.062942 2.645493 2.603701
H 0.601802 1.530228 -0.176331
H 1.193530 0.843664 1.337838
H 3.943658 4.273605 -0.285073
S -2.509422 1.183126 0.416457

O -3.198551 -0.081780 0.714884
O -1.832593 1.359293 -0.879085
C -3.640880 2.558646 0.605282
C -4.345905 2.719691 1.802479
C -3.852842 3.415377 -0.473080
C -5.262149 3.758996 1.912864
H -4.168865 2.045878 2.634122
C -4.777892 4.452635 -0.343611
H -3.299069 3.267379 -1.393643
C -5.495427 4.640334 0.843103
H -5.808515 3.891409 2.843609
H -4.943473 5.124812 -1.181566
C -6.508593 5.751764 0.975566
H -7.529947 5.351206 1.003682
H -6.360136 6.319050 1.901590
H -6.449695 6.450985 0.136275
H -0.713641 3.239654 1.084519
C 5.552238 3.008992 -0.923147
C 6.750115 2.454205 -0.456939
C 5.419072 3.257546 -2.296216
C 7.786826 2.149650 -1.340234
H 6.868301 2.253911 0.604619
C 6.451955 2.953122 -3.182806
H 4.499256 3.700125 -2.674751
C 7.640583 2.397170 -2.705828
H 8.708774 1.717960 -0.959961
H 6.330937 3.155347 -4.243854
H 8.448100 2.161529 -3.393767
H -1.192827 0.475155 2.058645
H 4.791087 3.481618 1.041365
89
-3063.45904368
C 3.135074 -4.458116 0.623406
C 3.145196 -4.272149 2.151685
C 1.991879 -3.458833 2.761497
C 1.905862 -2.005733 2.255172
C 1.903225 -5.187563 0.069658
N 0.790386 -4.278852 -0.144888
H 3.154307 -5.265889 2.622063
H 4.099610 -3.800446 2.425498
H 2.121428 -3.447740 3.852016
H 1.036111 -3.964505 2.576881
H 2.138027 -5.609403 -0.924209
H 4.020101 -5.045774 0.346977
H 3.230013 -3.494914 0.108709
H 2.886696 -1.523671 2.361164
S -0.731718 -5.051233 -0.205881
O -1.311812 -4.824146 1.130542
O -0.618257 -6.415352 -0.741920
C -1.591146 -4.043251 -1.399574
C -2.307530 -2.921532 -0.973857
C -1.537230 -4.394611 -2.749730
C -2.968002 -2.141782 -1.920162
H -2.355251 -2.678331 0.082436
C -2.196368 -3.596831 -3.682568
H -0.996093 -5.282843 -3.058431
C -2.917912 -2.461511 -3.287866
H -3.533523 -1.269314 -1.605293
H -2.153086 -3.862973 -4.735664
C -3.649592 -1.611051 -4.296841
H -3.634217 -0.556288 -4.007297
H -3.212236 -1.711410 -5.295398
H -4.702488 -1.915568 -4.368190
H 1.628966 -6.055811 0.688163
C 0.862665 -1.180567 2.981547
C 1.228794 -0.066070 3.748593
C -0.499204 -1.518343 2.912244
C 0.269925 0.696113 4.421609
H 2.280195 0.204542 3.823330
C -1.459075 -0.761352 3.585215
H -0.807187 -2.389838 2.338953
C -1.079400 0.351384 4.341006
H 0.579882 1.555182 5.011648
H -2.507149 -1.043538 3.522017
H -1.828152 0.939628 4.865054
H 1.678818 -2.014639 1.182150
Cl 3.634879 0.569561 -0.757104
C 0.573218 1.691722 -0.472607
C 1.748147 2.281820 0.315730
C 3.081450 2.295855 -0.433793
C 4.182245 3.055285 0.324460
C -0.723959 1.720240 0.344959
N -1.902280 1.174911 -0.354689
H 1.874307 1.749534 1.267471
H 1.518482 3.328009 0.568493
H 2.953201 2.723390 -1.432289
H -0.612305 1.138816 1.266577
H 0.423189 2.252246 -1.402285
H 0.800885 0.654671 -0.748699
H 3.753452 4.031523 0.591705
S -2.757055 2.159939 -1.417745
O -3.558190 1.221291 -2.220480
O -1.897651 3.148180 -2.085327
C -3.831304 3.067986 -0.306512
C -4.665704 2.377365 0.578149
C -3.869800 4.458242 -0.389136
C -5.535686 3.096496 1.389336
H -4.620765 1.295035 0.635811
C -4.750571 5.163728 0.432693
H -3.215497 4.972219 -1.084759
C -5.594723 4.499400 1.329309
H -6.181665 2.563166 2.082732
H -4.779960 6.248750 0.373450
C -6.555937 5.262015 2.209338
H -6.448102 4.973352 3.261497
H -6.395944 6.341874 2.137083
H -7.596350 5.059197 1.925529
H -0.971288 2.744231 0.646558
C 5.464785 3.271575 -0.450464
C 6.597583 2.483971 -0.213683
C 5.538787 4.271787 -1.429860
C 7.772837 2.685684 -0.939051
H 6.555868 1.703976 0.542154
C 6.710092 4.475346 -2.159180
H 4.671828 4.902138 -1.619631
C 7.832412 3.681312 -1.914983
H 8.642131 2.064236 -0.740451
H 6.748252 5.257354 -2.913082
H 8.747524 3.840432 -2.479104
H -1.745391 0.275451 -0.808223
H 4.385169 2.531716 1.265747
89
-3063.45790649
C -4.519491 0.887127 -1.060750
C -5.601818 0.152750 -1.861395
C -6.166161 -1.053528 -1.099052
C -7.205849 -1.852939 -1.910240
C -4.026050 2.161504 -1.801211
N -2.841824 2.718631 -1.177374
H -5.183201 -0.182015 -2.821518
H -6.419859 0.846060 -2.105626
H -6.629340 -0.711111 -0.163472
H -5.345680 -1.721729 -0.805683
H -3.734588 1.891937 -2.822719
H -4.904039 1.179201 -0.077636
H -3.663575 0.227358 -0.882277
H -8.020964 -1.181485 -2.210966
S -3.155434 3.652939 0.209751
O -3.077435 2.714051 1.344688
O -4.351479 4.494134 0.043832
C -1.718691 4.712077 0.219376
C -0.444631 4.150763 0.353545
C -1.895429 6.091128 0.122794
C 0.659471 4.995411 0.383732
H -0.322932 3.074360 0.420747
C -0.774498 6.920504 0.161577
H -2.894809 6.499358 0.018425
C 0.514485 6.389926 0.292509
H 1.654410 4.566959 0.478796
H -0.905546 7.997017 0.086776

C 1.724801 7.290759 0.351504
H 2.120388 7.352141 1.373727
H 2.533963 6.914947 -0.284870
H 1.484128 8.308309 0.029187
H -4.843056 2.894614 -1.841624
C -7.771064 -3.027022 -1.138996
C -7.094777 -4.254741 -1.103958
C -8.960877 -2.904522 -0.409136
C -7.590156 -5.327009 -0.361175
H -6.172320 -4.371672 -1.669308
C -9.461403 -3.973893 0.335346
H -9.502838 -1.961077 -0.428111
C -8.776602 -5.189782 0.362365
H -7.052395 -6.271775 -0.352053
H -10.388555 -3.857842 0.890934
H -9.166097 -6.024914 0.938516
H -6.736846 -2.209160 -2.837371
Cl 0.088136 0.169569 -0.369617
C 3.251459 -0.631462 -0.245216
C 2.392579 -0.467368 1.013875
C 0.935430 -0.907741 0.859309
C 0.160064 -0.884804 2.186230
C 4.695975 -0.170668 -0.015018
N 5.574390 -0.287663 -1.190358
H 2.414150 0.574307 1.362337
H 2.823255 -1.076581 1.822400
H 0.886808 -1.906013 0.415158
H 4.716375 0.882904 0.291746
H 3.261935 -1.681216 -0.558791
H 2.813899 -0.049820 -1.066349
H 0.781235 -1.427281 2.913464
S 6.288811 -1.780806 -1.537747
O 6.550234 -1.733242 -2.980368
O 5.524494 -2.884798 -0.939520
C 7.843185 -1.683661 -0.649709
C 8.790386 -0.723692 -1.019623
C 8.108868 -2.596660 0.367976
C 10.007009 -0.679658 -0.349229
H 8.572927 -0.024280 -1.820202
C 9.336627 -2.537422 1.030640
H 7.364460 -3.341102 0.628438
C 10.300987 -1.584524 0.685452
H 10.745339 0.066808 -0.632521
H 9.546425 -3.247507 1.826588
C 11.634720 -1.530961 1.391478
H 11.862899 -0.515869 1.737028
H 11.656353 -2.196540 2.259564
H 12.448844 -1.832590 0.720239
H 5.159417 -0.741171 0.796864
C -1.214716 -1.518135 2.137686
C -2.374228 -0.734002 2.104295
C -1.345305 -2.914276 2.127327
C -3.634210 -1.335462 2.061289
H -2.298536 0.350072 2.103755
C -2.602332 -3.516086 2.075467
H -0.453396 -3.537592 2.166715
C -3.753780 -2.726114 2.043143
H -4.523137 -0.710934 2.044585
H -2.683133 -4.600091 2.069087
H -4.735402 -3.191223 2.006820
H 5.169479 0.065779 -2.055753
H 0.092846 0.150852 2.538180
89
-3063.45747405
C 1.730340 -0.211445 -2.264576
C 2.661724 -1.372293 -1.875009
C 4.092992 -0.954818 -1.511205
C 4.932785 -2.123268 -0.960948
C 1.254457 0.637945 -1.073505
N 2.304643 1.490106 -0.554437
H 2.205013 -1.920028 -1.037607
H 2.697895 -2.083477 -2.712452
H 4.074517 -0.149687 -0.768455
H 4.588718 -0.533473 -2.396707
H 0.461508 1.322597 -1.430326
H 0.824634 -0.619530 -2.729530
H 2.211886 0.440197 -3.004581
H 4.425404 -2.527366 -0.072667
S 2.327882 1.912360 1.079758
O 2.204063 3.375522 1.103980
O 3.520351 1.251494 1.631003
C 0.899874 1.233769 1.937774
C -0.314352 1.923908 1.904116
C 1.025592 0.037305 2.644385
C -1.412640 1.395567 2.581324
H -0.391202 2.872752 1.384474
C -0.082670 -0.474330 3.317275
H 1.982267 -0.472682 2.674842
C -1.316847 0.187929 3.292450
H -2.349609 1.948280 2.578337
H 0.015225 -1.404036 3.871742
C -2.523390 -0.391134 3.992246
H -3.143474 -0.960432 3.286789
H -2.231506 -1.070989 4.798933
H -3.154952 0.394352 4.421226
H 0.792170 -0.005393 -0.313438
C 6.351013 -1.730889 -0.598129
C 6.597392 -0.811742 0.434130
C 7.447128 -2.265208 -1.286458
C 7.900953 -0.442775 0.764785
H 5.762443 -0.373954 0.976941
C 8.754227 -1.898967 -0.957313
H 7.274478 -2.978899 -2.089718
C 8.985590 -0.985722 0.071199
H 8.069632 0.271912 1.566456
H 9.589671 -2.328115 -1.505306
H 10.001259 -0.697803 0.330510
H 4.955566 -2.938171 -1.697090
Cl -1.794685 2.552081 -3.170566
C -3.904150 0.443700 -1.762314
C -3.641466 1.860644 -1.234724
C -3.404439 2.891725 -2.339390
C -3.481485 4.364541 -1.891416
C -4.130773 -0.601515 -0.658642
N -2.944146 -1.014461 0.111315
H -2.789403 1.881142 -0.544603
H -4.515228 2.199862 -0.657591
H -4.133688 2.747827 -3.142014
H -4.863700 -0.236982 0.072536
H -4.806694 0.464279 -2.389931
H -3.081245 0.111445 -2.401414
H -3.222481 4.990030 -2.752897
S -1.885867 -2.146875 -0.559186
O -0.654310 -2.009666 0.230976
O -1.845319 -2.049740 -2.025590
C -2.675749 -3.704378 -0.158129
C -2.744558 -4.118496 1.175681
C -3.178935 -4.501619 -1.183981
C -3.335074 -5.340674 1.474588
H -2.334024 -3.492886 1.961454
C -3.770122 -5.725392 -0.864172
H -3.098282 -4.167778 -2.212738
C -3.857316 -6.163706 0.461835
H -3.390715 -5.667325 2.510272
H -4.165077 -6.349619 -1.661598
C -4.477199 -7.497211 0.804044
H -3.710718 -8.219327 1.113273
H -5.187084 -7.406487 1.634131
H -5.008408 -7.924432 -0.051654
H -4.557024 -1.511600 -1.092552
C -2.660298 4.768768 -0.683142
C -3.271300 4.909890 0.571010
C -1.287490 5.039829 -0.789333
C -2.535237 5.305316 1.690160
H -4.339505 4.725858 0.669132
C -0.545154 5.425929 0.327975
H -0.796799 4.947623 -1.753442
C -1.168219 5.561752 1.571454
H -3.031805 5.420834 2.650326
H 0.521266 5.605138 0.232353

H -0.589342 5.865154 2.439227
H -2.394349 -0.264426 0.529491
H -4.542819 4.554462 -1.679017
89
-3063.45711094
C 1.064410 -1.416218 -2.017064
C 1.792677 -2.752368 -1.789702
C 3.311006 -2.628869 -1.600895
C 3.974769 -3.959071 -1.197099
C 0.850016 -0.583193 -0.740278
N 2.088432 -0.024365 -0.242423
H 1.348501 -3.256621 -0.917509
H 1.591001 -3.410554 -2.646601
H 3.536391 -1.872414 -0.840969
H 3.764882 -2.262132 -2.531798
H 0.220655 0.284074 -1.009374
H 0.061955 -1.604489 -2.419026
H 1.601559 -0.808904 -2.755988
H 3.514158 -4.308505 -0.261164
S 2.355236 0.198818 1.406140
O 2.400819 1.656942 1.600320
O 3.524296 -0.634611 1.718729
C 0.977518 -0.428697 2.371225
C 0.011494 0.456304 2.852397
C 0.882396 -1.799197 2.627181
C -1.071594 -0.046299 3.574052
H 0.130250 1.521770 2.687640
C -0.211774 -2.283925 3.337455
H 1.661048 -2.471046 2.280869
C -1.212748 -1.421671 3.807168
H -1.813502 0.641950 3.973866
H -0.293593 -3.350941 3.527035
C -2.426090 -1.970125 4.514933
H -3.156377 -2.328120 3.777857
H -2.168641 -2.817215 5.159417
H -2.915709 -1.208228 5.129862
H 0.286825 -1.161890 0.004077
C 5.475175 -3.852663 -1.012190
C 6.014350 -3.067557 0.019106
C 6.357805 -4.521876 -1.868730
C 7.394687 -2.959139 0.185440
H 5.349332 -2.529721 0.691443
C 7.740836 -4.416426 -1.704494
H 5.956683 -5.134955 -2.673465
C 8.264179 -3.634120 -0.675153
H 7.791985 -2.345110 0.989755
H 8.407060 -4.946286 -2.381031
H 9.339918 -3.549600 -0.543736
H 3.751891 -4.725114 -1.952049
Cl -0.055402 3.078619 -1.880630
C -2.990635 2.432451 -0.734779
C -1.883208 2.950039 0.190005
C -0.877487 3.904861 -0.459949
C 0.179578 4.403472 0.538008
C -4.058946 1.598893 -0.009899
N -3.617673 0.353463 0.644884
H -1.326998 2.109463 0.624927
H -2.334960 3.498423 1.031716
H -1.399360 4.752032 -0.914863
H -4.537408 2.194308 0.778208
H -3.504981 3.286382 -1.198493
H -2.556244 1.841723 -1.544884
H -0.379136 4.835059 1.382321
S -3.275707 -1.018458 -0.271873
O -2.645293 -1.940944 0.682458
O -2.589229 -0.697161 -1.534222
C -4.916165 -1.597990 -0.699042
C -5.804677 -1.970035 0.314938
C -5.267027 -1.722778 -2.041931
C -7.057811 -2.462248 -0.030919
H -5.516418 -1.868214 1.355944
C -6.529686 -2.219405 -2.369808
H -4.558456 -1.434205 -2.810597
C -7.440455 -2.597197 -1.376564
H -7.753587 -2.748990 0.753963
H -6.808243 -2.314656 -3.416133
C -8.797566 -3.154130 -1.733661
H -9.589785 -2.691282 -1.134400
H -9.032440 -2.993003 -2.789968
H -8.840629 -4.234607 -1.545367
H -4.853547 1.323200 -0.711427
C 1.146308 5.434545 -0.004506
C 2.481717 5.094198 -0.252155
C 0.725962 6.746270 -0.263248
C 3.374178 6.042698 -0.754847
H 2.819661 4.082341 -0.043945
C 1.614831 7.695022 -0.768256
H -0.305517 7.031291 -0.061563
C 2.943638 7.344119 -1.016502
H 4.407738 5.762512 -0.940405
H 1.272495 8.708515 -0.961797
H 3.639176 8.082545 -1.406757
H -2.883430 0.452833 1.344602
H 0.735156 3.542546 0.925325
89
-3063.45695091
C -1.209383 -1.107968 2.251733
C -1.675448 -2.569078 2.127238
C -3.129719 -2.740642 1.667172
C -3.499101 -4.208820 1.382291
C -0.888389 -0.427932 0.909315
N -2.079481 -0.174920 0.129000
H -1.001784 -3.100148 1.436667
H -1.547697 -3.060559 3.101906
H -3.313132 -2.145075 0.765774
H -3.804392 -2.336597 2.434282
H -0.457998 0.566416 1.127833
H -0.282034 -1.066629 2.834931
H -1.958001 -0.512180 2.788202
H -2.817368 -4.596527 0.610664
S -2.038855 -0.194332 -1.553209
O -2.241530 1.204563 -1.962307
O -2.992039 -1.239019 -1.950230
C -0.422445 -0.697026 -2.165380
C 0.494205 0.275818 -2.569278
C -0.110258 -2.054723 -2.262523
C 1.738107 -0.120675 -3.060033
H 0.214840 1.323240 -2.533458
C 1.139871 -2.433306 -2.749026
H -0.846052 -2.801698 -1.982865
C 2.085310 -1.477574 -3.149312
H 2.443120 0.633636 -3.402332
H 1.379098 -3.490415 -2.834469
C 3.444495 -1.896597 -3.655365
H 3.848882 -1.169466 -4.366752
H 4.161295 -1.977839 -2.827546
H 3.403437 -2.872006 -4.150782
H -0.111676 -0.987205 0.373266
C -4.932998 -4.392466 0.927274
C -5.367464 -3.862812 -0.297975
C -5.859623 -5.081262 1.719356
C -6.688812 -4.020672 -0.714896
H -4.669122 -3.314365 -0.926619
C -7.183607 -5.242235 1.304606
H -5.539793 -5.498350 2.672268
C -7.602621 -4.712255 0.084367
H -7.005391 -3.601637 -1.666709
H -7.885407 -5.781965 1.935781
H -8.632098 -4.835498 -0.242032
H -3.319122 -4.813954 2.281027
Cl -0.431891 3.433228 1.770715
C 2.669815 3.402439 0.770694
C 1.490764 3.477030 -0.205965
C 0.273699 4.263041 0.289181
C -0.804520 4.412418 -0.795391
C 3.861769 2.594448 0.235074
N 3.625934 1.154594 0.022496
H 1.152485 2.468440 -0.477138
H 1.823921 3.958650 -1.138327
H 0.578049 5.246532 0.659184

H 4.195837 2.993232 -0.731477
H 3.026432 4.419999 0.984942
H 2.349168 2.973895 1.725175
H -0.304008 4.882923 -1.655254
S 3.607388 0.107232 1.347274
O 2.309754 -0.585402 1.346996
O 4.084355 0.861689 2.510300
C 4.852339 -1.120159 0.946548
C 4.481341 -2.287129 0.277408
C 6.179764 -0.896173 1.315205
C 5.459972 -3.229790 -0.035797
H 3.440525 -2.457680 0.022815
C 7.143296 -1.849289 0.993924
H 6.446788 0.004103 1.858387
C 6.802248 -3.028305 0.315175
H 5.173982 -4.143270 -0.551694
H 8.177748 -1.678005 1.281602
C 7.849951 -4.070219 0.003964
H 7.562590 -4.684911 -0.854999
H 8.819760 -3.610923 -0.214225
H 7.995597 -4.746948 0.856240
H 4.702236 2.679548 0.926932
C -2.010721 5.238994 -0.402873
C -3.256812 4.630024 -0.212135
C -1.905639 6.626021 -0.231148
C -4.370596 5.389545 0.150456
H -3.351503 3.556731 -0.355416
C -3.015860 7.386904 0.134173
H -0.947454 7.118076 -0.390735
C -4.253289 6.768767 0.327166
H -5.330854 4.900998 0.293255
H -2.916782 8.461866 0.262180
H -5.120646 7.360151 0.608671
H 2.851439 0.924858 -0.596398
H -1.126616 3.416865 -1.119839
89
-3063.45596068
C 2.628500 -1.019652 -2.281650
C 4.156573 -1.186639 -2.393325
C 4.946919 -0.643359 -1.193659
C 6.469201 -0.826713 -1.355637
C 1.895823 -2.125978 -1.513107
N 2.316868 -2.219559 -0.129400
H 4.399674 -2.248383 -2.550134
H 4.495425 -0.664425 -3.299056
H 4.614263 -1.133380 -0.272577
H 4.724152 0.425352 -1.069802
H 0.806970 -1.958049 -1.560683
H 2.188705 -1.008894 -3.288142
H 2.389899 -0.051780 -1.825447
H 6.692437 -1.899900 -1.432441
S 1.709522 -3.533794 0.773118
O 1.773627 -3.117159 2.173653
O 2.467627 -4.695343 0.280778
C -0.021308 -3.776759 0.368543
C -0.997105 -3.131223 1.134945
C -0.381689 -4.627569 -0.678101
C -2.341113 -3.341789 0.836171
H -0.697170 -2.503973 1.967891
C -1.732396 -4.816258 -0.968288
H 0.385344 -5.154853 -1.235737
C -2.733451 -4.177834 -0.223709
H -3.104971 -2.873629 1.454452
H -2.014784 -5.481902 -1.779699
C -4.194150 -4.377526 -0.544355
H -4.335423 -5.174566 -1.280355
H -4.762439 -4.643012 0.355109
H -4.634732 -3.458225 -0.948968
H 2.070061 -3.102761 -1.997649
C 7.260437 -0.221455 -0.215211
C 7.363780 -0.882894 1.016872
C 7.880172 1.028309 -0.349163
C 8.060317 -0.312624 2.082570
H 6.892410 -1.855514 1.141119
C 8.579979 1.603850 0.713957
H 7.818573 1.552839 -1.300696
C 8.671111 0.934628 1.935486
H 8.128457 -0.844542 3.028151
H 9.060379 2.570768 0.584542
H 9.217407 1.378177 2.763803
H 6.790289 -0.374134 -2.303751
Cl 1.216254 1.000425 0.769134
C -1.973829 1.308361 0.370774
C -1.129313 2.430064 0.986244
C 0.282086 2.541308 0.404779
C 1.064925 3.754026 0.936279
C -3.353049 1.184012 1.026644
N -4.153200 0.049057 0.530687
H -1.055897 2.302264 2.074666
H -1.626015 3.396419 0.814235
H 0.238116 2.580283 -0.687621
H -3.249613 1.047006 2.110349
H -2.105695 1.479012 -0.703730
H -1.443689 0.354682 0.485192
H 0.403598 4.624584 0.824376
S -5.062152 0.243399 -0.879959
O -5.282836 -1.128942 -1.357460
O -4.479226 1.257359 -1.770056
C -6.599957 0.916091 -0.253879
C -7.345718 0.189848 0.680214
C -7.063120 2.131875 -0.751629
C -8.560288 0.701423 1.121097
H -6.973161 -0.756704 1.057478
C -8.285895 2.629857 -0.297383
H -6.471550 2.673100 -1.481884
C -9.051098 1.927739 0.640122
H -9.141328 0.141980 1.850399
H -8.649105 3.579598 -0.681585
C -10.378071 2.461517 1.123807
H -11.203603 1.810942 0.809004
H -10.410776 2.514750 2.218488
H -10.574954 3.463436 0.731160
H -3.943889 2.094590 0.880989
C 2.370690 4.024046 0.217586
C 2.381392 4.778582 -0.963581
C 3.584103 3.512863 0.695828
C 3.570929 5.011037 -1.654751
H 1.449283 5.193478 -1.343329
C 4.776711 3.739729 0.006544
H 3.593936 2.925182 1.609465
C 4.772288 4.489502 -1.171432
H 3.559336 5.602319 -2.566804
H 5.706158 3.325825 0.388142
H 5.700408 4.670064 -1.707418
H -3.619650 -0.814307 0.429777
H 1.233395 3.619199 2.010978
89
-3063.45588507
C 2.627743 -1.018196 -2.281570
C 4.155697 -1.185324 -2.394470
C 4.947028 -0.643477 -1.194818
C 6.469187 -0.826647 -1.358226
C 1.895394 -2.124362 -1.512494
N 2.316639 -2.217513 -0.128824
H 4.398533 -2.246918 -2.552768
H 4.493914 -0.662099 -3.299862
H 4.615126 -1.134507 -0.274002
H 4.724373 0.425094 -1.069562
H 0.806523 -1.956491 -1.559931
H 2.187284 -1.007454 -3.287774
H 2.389611 -0.050265 -1.825250
H 6.692401 -1.899756 -1.436163
S 1.709481 -3.531605 0.774105
O 1.773253 -3.114415 2.174491
O 2.467948 -4.693144 0.282320
C -0.021235 -3.775228 0.369374
C -0.997354 -3.129704 1.135375
C -0.381182 -4.626607 -0.676954
C -2.341252 -3.340880 0.836514
H -0.697750 -2.501993 1.968093

C -1.731784 -4.815883 -0.967251
H 0.386104 -5.153862 -1.234268
C -2.733156 -4.177507 -0.223066
H -3.105361 -2.872755 1.454514
H -2.013828 -5.481954 -1.778431
C -4.193751 -4.377800 -0.543816
H -4.634531 -3.458820 -0.948942
H -4.334686 -5.175242 -1.279448
H -4.762085 -4.643008 0.355698
H 2.069558 -3.101268 -1.996818
C 7.261309 -0.222325 -0.217920
C 7.880799 1.027622 -0.351310
C 7.365705 -0.884786 1.013523
C 8.581375 1.602349 0.711739
H 7.818381 1.552953 -1.302348
C 8.063010 -0.315319 2.079159
H 6.894534 -1.857557 1.137341
C 8.673545 0.932119 1.932638
H 9.061553 2.569436 0.582764
H 8.131914 -0.848052 3.024225
H 9.220404 1.375065 2.760906
H 6.789472 -0.373214 -2.306204
Cl 1.215641 1.000066 0.770075
C -1.974247 1.308201 0.371171
C -1.129849 2.429858 0.986908
C 0.281702 2.541102 0.405799
C 1.064476 3.753673 0.937738
C -3.353572 1.183787 1.026792
N -4.153607 0.048801 0.530736
H -1.056689 2.301974 2.075338
H -1.626488 3.396235 0.814853
H 0.238009 2.580285 -0.686606
H -3.250330 1.046818 2.110521
H -2.105921 1.478950 -0.703339
H -1.444096 0.354534 0.485606
H 0.403257 4.624293 0.825703
S -5.062196 0.242984 -0.880154
O -5.282532 -1.129393 -1.357715
O -4.479172 1.257032 -1.770083
C -6.600302 0.915449 -0.254552
C -7.345890 0.189484 0.679875
C -7.063835 2.130820 -0.753006
C -8.560695 0.700887 1.120357
H -6.972968 -0.756646 1.057836
C -8.286819 2.628620 -0.299172
H -6.472301 2.671926 -1.483378
C -9.051931 1.926698 0.638577
H -9.141542 0.141720 1.850022
H -8.650258 3.578088 -0.683839
C -10.379567 2.459899 1.121074
H -10.417035 2.503895 2.215969
H -10.572221 3.465550 0.735924
H -11.205381 1.814181 0.797014
H -3.944427 2.094338 0.881022
C 2.370537 4.023720 0.219596
C 3.583667 3.511956 0.697937
C 2.381814 4.778904 -0.961157
C 4.776550 3.738870 0.009139
H 3.593071 2.923788 1.611263
C 3.571625 5.011415 -1.651831
H 1.449930 5.194252 -1.340963
C 4.772695 4.489286 -1.168424
H 5.705774 3.324511 0.390789
H 3.560483 5.603201 -2.563562
H 5.701030 4.669868 -1.704026
H -3.620136 -0.814645 0.430118
H 1.232517 3.618684 2.012488
89
-3063.45585861
C 3.647524 -0.518793 -2.278193
C 4.709776 -1.616538 -2.094352
C 6.060438 -1.123454 -1.558353
C 7.030513 -2.277436 -1.240894
C 2.997288 -0.032444 -0.970334
N 3.894316 0.801393 -0.198482
H 4.308422 -2.391229 -1.422890
H 4.867056 -2.113192 -3.062342
H 5.909645 -0.528708 -0.651996
H 6.521150 -0.448382 -2.292622
H 2.133913 0.605692 -1.232498
H 2.832833 -0.902156 -2.906267
H 4.073991 0.344205 -2.804063
H 6.548392 -2.945226 -0.512494
S 3.854896 0.717430 1.494207
O 4.022228 2.095920 1.958402
O 4.837202 -0.326923 1.833596
C 2.244995 0.150858 2.044517
C 1.205898 1.076815 2.186271
C 2.047893 -1.198237 2.342872
C -0.041042 0.636005 2.619549
H 1.384915 2.128088 1.984994
C 0.791643 -1.619991 2.778703
H 2.874572 -1.895674 2.257129
C -0.270990 -0.717752 2.919310
H -0.848324 1.354825 2.735846
H 0.637231 -2.668316 3.021403
C -1.632403 -1.177748 3.379967
H -1.613357 -2.225371 3.696056
H -1.986000 -0.576453 4.225916
H -2.375077 -1.076291 2.579364
H 2.590111 -0.881292 -0.401273
C 8.360473 -1.806274 -0.688738
C 8.432435 -1.206424 0.578328
C 9.541347 -1.942235 -1.428497
C 9.651154 -0.758013 1.086506
H 7.525473 -1.083840 1.166591
C 10.763978 -1.495213 -0.922202
H 9.503374 -2.406124 -2.412277
C 10.822633 -0.901355 0.338525
H 9.685919 -0.295587 2.069746
H 11.668921 -1.612641 -1.513475
H 11.772295 -0.552697 0.736259
H 7.199228 -2.877028 -2.145716
Cl 0.037031 1.557339 -3.319409
C -2.756726 0.287089 -2.216277
C -2.643457 1.758846 -2.637115
C -1.292306 2.435161 -2.384486
C -0.860667 2.559403 -0.911140
C -4.187259 -0.261765 -2.329829
N -5.175753 0.299027 -1.389629
H -3.379989 2.368901 -2.095885
H -2.883666 1.852621 -3.703540
H -1.307549 3.429951 -2.836087
H -4.587057 -0.086959 -3.336506
H -2.109093 -0.318975 -2.860650
H -2.410721 0.138396 -1.189547
H -0.775502 1.563334 -0.465449
S -5.236775 -0.266724 0.196052
O -5.945500 0.783302 0.937553
O -3.914548 -0.732072 0.653544
C -6.277630 -1.716501 0.055072
C -7.621852 -1.562349 -0.299629
C -5.748987 -2.974052 0.337453
C -8.432855 -2.687997 -0.377925
H -8.021209 -0.575256 -0.508169
C -6.579304 -4.093341 0.252111
H -4.707031 -3.068179 0.622787
C -7.926722 -3.970682 -0.104305
H -9.478322 -2.573351 -0.653854
H -6.170332 -5.076600 0.469766
C -8.827150 -5.179835 -0.185087
H -9.333754 -5.234487 -1.155871
H -8.266301 -6.108390 -0.044004
H -9.609008 -5.141321 0.583697
H -4.185578 -1.346026 -2.181053
C -1.819746 3.418467 -0.108134
C -1.751186 4.817215 -0.179210
C -2.811544 2.837102 0.695027
C -2.653921 5.613287 0.526834
H -0.978573 5.286890 -0.784898

C -3.722210 3.630381 1.396805
H -2.873948 1.754965 0.777624
C -3.644959 5.021637 1.313410
H -2.580799 6.695939 0.464952
H -4.491742 3.152698 1.996271
H -4.349673 5.641929 1.860657
H -5.230983 1.315156 -1.362088
H 0.141242 3.002313 -0.895072

$(C_6\text{-Cl---N-rad})_{pic,XAT}$

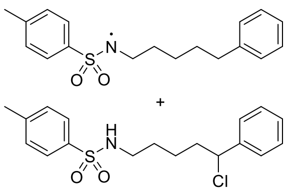

Name	E(B3LYP)	G(B3LYP)	E(RO-B2PLYP-D3)	G(RO-B2PLYP-D3)
Conformers				
Tosyl_NH_pentane_Ph_C6_Cl_Tosyl_N_radical_pentane_ separate_no_hydrogen_bond_0018_B2PLYP	-3065.153056	-3064.366902	-3064.096238	-3063.45526261
Tosyl_NH_pentane_Ph_C6_Cl_Tosyl_N_radical_pentane_ separate_no_hydrogen_bond_0015_B2PLYP	-3065.152714	-3064.366178	-3064.096252	-3063.45448392
Tosyl_NH_pentane_Ph_C6_Cl_Tosyl_N_radical_pentane_ separate_no_hydrogen_bond_0003_B2PLYP	-3065.152714	-3064.366179	-3064.096241	-3063.45443420
Tosyl_NH_pentane_Ph_C6_Cl_Tosyl_N_radical_pentane_ separate_no_hydrogen_bond_0009_B2PLYP	-3065.152714	-3064.366179	-3064.096248	-3063.45433622
Tosyl_NH_pentane_Ph_C6_Cl_Tosyl_N_radical_pentane_ separate_no_hydrogen_bond_0012_B2PLYP	-3065.152714	-3064.366177	-3064.096240	-3063.45433382
Tosyl_NH_pentane_Ph_C6_Cl_Tosyl_N_radical_pentane_ separate_no_hydrogen_bond_0014_B2PLYP	-3065.152714	-3064.366179	-3064.096248	-3063.45432832
Tosyl_NH_pentane_Ph_C6_Cl_Tosyl_N_radical_pentane_ separate_no_hydrogen_bond_0017_B2PLYP	-3065.152714	-3064.366179	-3064.096248	-3063.45432777
Tosyl_NH_pentane_Ph_C6_Cl_Tosyl_N_radical_pentane_ separate_no_hydrogen_bond_0006_B2PLYP	-3065.152714	-3064.366179	-3064.096249	-3063.45432758
Tosyl_NH_pentane_Ph_C6_Cl_Tosyl_N_radical_pentane_ separate_no_hydrogen_bond_0010_B2PLYP	-3065.152714	-3064.366179	-3064.096246	-3063.45432608
Tosyl_NH_pentane_Ph_C6_Cl_Tosyl_N_radical_pentane_ separate_no_hydrogen_bond_0019_B2PLYP	-3065.152714	-3064.366179	-3064.096252	-3063.45432515
Post-reactive complexes for XAT				
Tosyl_NH_pentane_Ph_C6_H_Tosyl_N_pentane_TS_md_ 00450_IRC_forward_opt_B2PLYP_pic	-3065.149745	-3064.363299	-3064.097452	-3063.45658419
Tosyl_NH_pentane_Ph_C6_H_Tosyl_N_pentane_TS_md_ 00250_IRC_forward_opt_B2PLYP_pic	-3065.154723	-3064.368338	-3064.093384	-3063.45429222
Tosyl_NH_pentane_Ph_C6_H_Tosyl_N_pentane_TS_md_ 00200_IRC_forward_opt_B2PLYP_pic	-3065.154811	-3064.368311	-3064.096848	-3063.45404621
Tosyl_NH_pentane_Ph_C6_H_Tosyl_N_pentane_TS_md_ 00000_IRC_reverse_opt_B2PLYP_pic	-3065.153486	-3064.367225	-3064.094131	-3063.45335823
Tosyl_NH_pentane_Ph_C6_H_Tosyl_N_pentane_TS_md_ 00650_IRC_reverse_opt_B2PLYP_pic	-3065.150522	-3064.363873	-3064.091641	-3063.44964078

89
-3063.45526261
C 4.955015 3.518656 0.282526
C 5.330121 2.154874 -0.307300
C 6.691732 2.162766 -1.011134
C 7.084199 0.800399 -1.628093
C 3.595486 3.506129 1.010443
N 2.506713 3.397182 0.052664
H 4.551404 1.840037 -1.014597
H 5.334638 1.404759 0.494224
H 7.472861 2.473743 -0.302820
H 6.681767 2.915259 -1.812276
H 3.570896 2.744623 1.799953
H 5.712796 3.834926 1.012043
H 4.926476 4.283815 -0.503236
H 6.296923 0.489373 -2.327307
S 1.404698 2.144469 0.369220
O 1.136328 2.004967 1.809728
O 1.922039 0.984468 -0.386826
C -0.070900 2.743537 -0.427856
C -0.175111 2.685373 -1.818526
C -1.120672 3.231489 0.356983
C -1.349180 3.127873 -2.424708
H 0.641267 2.287193 -2.411695
C -2.289360 3.660248 -0.267370
H -1.019425 3.260609 1.436846
C -2.422058 3.617808 -1.665958
H -1.438492 3.080989 -3.506903
H -3.121841 4.011882 0.334812
C -3.697357 4.088338 -2.319238
H -3.809332 5.175398 -2.217324
H -3.713075 3.850965 -3.387387
H -4.566029 3.625315 -1.839826
H 3.450066 4.484694 1.497233
C 7.321892 -0.295812 -0.609238
C 6.302712 -1.187983 -0.247938
C 8.569156 -0.428632 0.017564
C 6.517739 -2.178633 0.712160
H 5.327574 -1.102737 -0.722372
C 8.791277 -1.417699 0.976336

H 9.377146 0.247908 -0.254605
C 7.764774 -2.296822 1.327731
H 5.709638 -2.856849 0.973702
H 9.768055 -1.505122 1.445546
H 7.936806 -3.069259 2.072613
H 7.993758 0.941244 -2.225911
C -2.758594 -1.039035 2.405014
C -1.331241 -0.626187 2.012255
C -0.394620 -1.807546 1.664241
C -0.059373 -1.962563 0.177786
C -3.639809 0.147972 2.819887
N -3.959001 1.096138 1.742140
H -0.890500 -0.053831 2.837764
H -1.361692 0.063268 1.158426
H -0.801323 -2.751314 2.047962
H -3.234286 -1.591369 1.587011
H -2.705175 -1.724836 3.262721
H 0.311176 -1.013624 -0.212364
S -5.452790 1.135369 0.998524
O -5.362393 2.256008 0.053048
O -6.454993 1.093240 2.065434
C -5.664776 -0.360809 0.025389
C -6.428964 -1.410802 0.534804
C -5.055762 -0.463877 -1.228682
C -6.571810 -2.576793 -0.217877
H -6.919784 -1.301624 1.495839
C -5.209064 -1.634075 -1.965312
H -4.482470 0.367018 -1.626426
C -5.962439 -2.710925 -1.471321
H -7.172846 -3.393214 0.174871
H -4.737535 -1.713916 -2.941707
C -6.097175 -3.983365 -2.272860
H -6.887685 -4.626852 -1.874742
H -6.327944 -3.771910 -3.323124
H -5.161709 -4.557237 -2.257462
H -3.143420 0.716227 3.618080
C 0.945006 -3.051115 -0.112507
C 2.195142 -2.701704 -0.638012
C 0.678600 -4.398322 0.169524
C 3.163644 -3.680630 -0.873775
H 2.407945 -1.657736 -0.854801
C 1.644115 -5.374789 -0.066424
H -0.296072 -4.683871 0.555933
C 2.890952 -5.018603 -0.587886
H 4.128470 -3.397296 -1.286227
H 1.422847 -6.416153 0.152431
H 3.642051 -5.781411 -0.775216
H -3.210841 1.336193 1.097985
Cl -1.617379 -2.251703 -0.796172
H -4.592961 -0.193379 3.232925
H 0.573179 -1.665521 2.161975
89
-3063.45448392
C 4.861024 2.843235 -0.302193
C 6.262242 2.653143 0.294494
C 7.338910 2.321047 -0.751986
C 7.176359 0.954454 -1.459046
C 3.810829 3.123419 0.802773
N 2.512645 3.367730 0.192518
H 6.234147 1.856923 1.049555
H 6.555082 3.571508 0.821601
H 8.319483 2.344085 -0.258521
H 7.359498 3.109549 -1.517317
H 3.806980 2.306464 1.535087
H 4.856896 3.676039 -1.015971
H 4.541707 1.949688 -0.846593
H 6.226257 0.930173 -2.005885
S 1.462872 2.041385 0.316027
O 1.191999 1.774109 1.738664
O 2.013979 0.962856 -0.531896
C -0.025117 2.657227 -0.445291
C -0.178139 2.562529 -1.828329
C -1.029278 3.206671 0.358477
C -1.353546 3.035718 -2.410233
H 0.601696 2.113509 -2.434130
C -2.199375 3.665578 -0.240915
H -0.894314 3.260468 1.433790
C -2.378798 3.592557 -1.633594
H -1.479789 2.961947 -3.487248
H -2.996575 4.068824 0.376685
C -3.651968 4.102875 -2.260899
H -3.702649 3.854258 -3.325433
H -4.525303 3.678150 -1.755350
H -3.720520 5.194394 -2.169445
H 4.081655 4.052556 1.321368
C 7.253691 -0.236124 -0.524323
C 8.494319 -0.695072 -0.057810
C 6.096909 -0.896043 -0.086588
C 8.578032 -1.775787 0.820071
H 9.405534 -0.203220 -0.393114
C 6.174568 -1.978539 0.793127
H 5.122797 -0.561014 -0.437388
C 7.416560 -2.421826 1.249351
H 9.550850 -2.117490 1.164586
H 5.263202 -2.476299 1.113445
H 7.479990 -3.265969 1.930858
H 7.965976 0.873065 -2.217300
C -2.808332 -1.045530 2.387468
C -1.372647 -0.641743 2.016190
C -0.444662 -1.822102 1.645628
C -0.133222 -1.977458 0.153659
C -3.670545 0.141634 2.840027
N -3.966483 1.132205 1.794458
H -0.932025 -0.099377 2.861818
H -1.384617 0.072996 1.183045
H -0.843046 -2.766846 2.035788
H -3.290182 -1.561400 1.549784
H -2.769886 -1.760891 3.221527
H 0.233700 -1.029660 -0.243084
S -5.456758 1.227488 1.050028
O -5.336674 2.369989 0.134594
O -6.463092 1.181682 2.112842
C -5.702516 -0.237946 0.038396
C -6.511419 -1.271736 0.510070
C -5.075858 -0.333575 -1.207722
C -6.682575 -2.414069 -0.272651
H -7.013214 -1.167827 1.465975
C -5.257721 -1.479918 -1.974499
H -4.465138 0.484286 -1.575888
C -6.057554 -2.540305 -1.518984
H -7.318076 -3.217770 0.090965
H -4.771968 -1.554103 -2.944358
C -6.225519 -3.786541 -2.354753
H -5.299881 -4.375920 -2.371085
H -7.021079 -4.427959 -1.963559
H -6.468738 -3.540447 -3.394784
H -3.168687 0.672345 3.660360
C 0.868413 -3.066389 -0.145941
C 2.123699 -2.713740 -0.656990
C 0.597084 -4.415609 0.120462
C 3.094061 -3.691209 -0.891252
H 2.339074 -1.668620 -0.865466
C 1.563502 -5.391069 -0.116705
H -0.381323 -4.703130 0.495788
C 2.816286 -5.031460 -0.621210
H 4.064694 -3.402889 -1.285707
H 1.338496 -6.434270 0.089309
H 3.568674 -5.793390 -0.806976
H -3.211637 1.380180 1.161211
Cl -1.702050 -2.264410 -0.801060
H -4.631911 -0.197073 3.235914
H 0.532185 -1.679265 2.125127
89
-3063.45443420
C 4.862918 2.842982 -0.302131
C 6.263390 2.652539 0.296183
C 7.341166 2.319812 -0.748954
C 7.178822 0.953355 -1.456345
C 3.811638 3.124051 0.801595
N 2.514018 3.367896 0.189975

H 6.234122 1.856516 1.051406
H 6.555977 3.570929 0.823390
H 8.321114 2.342280 -0.254223
H 7.363174 3.108332 -1.514229
H 3.807010 2.307700 1.534574
H 4.859883 3.675504 -1.016245
H 4.543867 1.949352 -0.846542
H 6.229533 0.929694 -2.004611
S 1.464221 2.041541 0.312990
O 1.193974 1.772668 1.735448
O 2.015002 0.963920 -0.536336
C -0.024146 2.658189 -0.446946
C -0.177266 2.566143 -1.830173
C -1.028593 3.205307 0.358021
C -1.353076 3.039614 -2.410996
H 0.602815 2.118878 -2.436959
C -2.199133 3.664517 -0.240320
H -0.893529 3.257075 1.433420
C -2.378674 3.594099 -1.633109
H -1.479422 2.967861 -3.488136
H -2.996562 4.065940 0.378182
C -3.652329 4.104703 -2.259212
H -3.704203 3.855956 -3.323657
H -4.525236 3.680291 -1.752709
H -3.720421 5.196268 -2.167873
H 4.082013 4.053594 1.319705
C 7.254033 -0.237388 -0.521650
C 6.096222 -0.896784 -0.085815
C 8.493697 -0.697046 -0.053270
C 6.171973 -1.979436 0.793879
H 5.122808 -0.561208 -0.438037
C 8.575495 -1.777920 0.824595
H 9.405687 -0.205622 -0.387094
C 7.413028 -2.423420 1.251979
H 5.259862 -2.476785 1.112702
H 9.547602 -2.120164 1.170581
H 7.474962 -3.267684 1.933475
H 7.969537 0.871582 -2.213416
C -2.806469 -1.044620 2.386878
C -1.371544 -0.640276 2.013279
C -0.443058 -1.820329 1.642915
C -0.133385 -1.977267 0.150753
C -3.668683 0.142359 2.839927
N -3.966163 1.132438 1.794274
H -0.930195 -0.096573 2.857667
H -1.385136 0.073426 1.179263
H -0.840145 -2.764944 2.034707
H -3.289243 -1.561274 1.550213
H -2.766470 -1.759427 3.221337
H 0.232540 -1.029743 -0.247582
S -5.457459 1.227258 1.051800
O -5.339044 2.369811 0.136231
O -6.462323 1.181026 2.115974
C -5.703987 -0.238171 0.040349
C -6.511795 -1.272388 0.512923
C -5.078827 -0.333387 -1.206571
C -6.683364 -2.414742 -0.269703
H -7.012467 -1.168823 1.469457
C -5.261043 -1.479755 -1.973209
H -4.469063 0.484865 -1.575462
C -6.059772 -2.540598 -1.516786
H -7.318071 -3.218755 0.094614
H -4.776396 -1.553594 -2.943653
C -6.227912 -3.786962 -2.352322
H -6.470368 -3.541055 -3.392566
H -5.302539 -4.376793 -2.367915
H -7.023991 -4.427899 -1.961395
H -3.166123 0.673602 3.659486
C 0.868465 -3.066047 -0.148737
C 2.123278 -2.713290 -0.660868
C 0.597833 -4.415172 0.118871
C 3.093870 -3.690569 -0.895024
H 2.338129 -1.668255 -0.870298
C 1.564465 -5.390437 -0.118216
H -0.380224 -4.702770 0.495047
C 2.816783 -5.030726 -0.623816
H 4.064139 -3.402154 -1.290305
H 1.339993 -6.433574 0.088705
H 3.569333 -5.792512 -0.809515
H -3.212177 1.380002 1.159849
Cl -1.703239 -2.266214 -0.801655
H -4.629476 -0.196579 3.236997
H 0.534236 -1.675965 2.121046
89
-3063.45433622
C 4.859121 2.844023 -0.302193
C 6.260411 2.654579 0.294538
C 7.337315 2.323299 -0.751969
C 7.175711 0.956689 -1.459210
C 3.808746 3.123733 0.802727
N 2.510546 3.367901 0.192472
H 6.232729 1.858216 1.049462
H 6.552716 3.573020 0.821808
H 8.317862 2.346930 -0.258486
H 7.357378 3.111924 -1.517187
H 3.804983 2.306646 1.534894
H 4.854648 3.676862 -1.015931
H 4.540234 1.950364 -0.846665
H 6.225565 0.931765 -2.005937
S 1.461175 2.041130 0.315015
O 1.190764 1.772256 1.737440
O 2.012378 0.963633 -0.534181
C -0.027215 2.657340 -0.445226
C -0.180624 2.564028 -1.828315
C -1.031341 3.205546 0.359428
C -1.356407 3.037337 -2.409359
H 0.599215 2.115961 -2.434818
C -2.201824 3.664591 -0.239115
H -0.896053 3.258308 1.434752
C -2.381655 3.592918 -1.631812
H -1.482960 2.964643 -3.486411
H -2.999004 4.066851 0.379154
C -3.655284 4.103283 -2.258175
H -3.706754 3.854742 -3.322685
H -4.528214 3.678486 -1.752007
H -3.723784 5.194794 -2.166582
H 4.079342 4.052833 1.321516
C 7.254059 -0.233965 -0.524660
C 8.495089 -0.691764 -0.058073
C 6.097884 -0.895090 -0.087153
C 8.579774 -1.772517 0.819659
H 9.405855 -0.198966 -0.393207
C 6.176518 -1.977630 0.792431
H 5.123474 -0.560967 -0.437990
C 7.418892 -2.419758 1.248728
H 9.552890 -2.113310 1.164237
H 5.265606 -2.476316 1.112596
H 7.483081 -3.263928 1.930130
H 7.965301 0.875995 -2.217568
C -2.808245 -1.048466 2.387235
C -1.372863 -0.643029 2.016569
C -0.443215 -1.822052 1.645967
C -0.132998 -1.978452 0.153837
C -3.671427 0.137421 2.841288
N -3.967684 1.129431 1.797151
H -0.933136 -0.100558 2.862605
H -1.385249 0.072062 1.183721
H -0.839388 -2.767120 2.037599
H -3.289658 -1.563700 1.548929
H -2.769268 -1.764811 3.220425
H 0.232214 -1.030517 -0.244167
S -5.457788 1.225018 1.052394
O -5.338320 2.369469 0.139315
O -6.464432 1.176243 2.114765
C -5.701924 -0.238570 0.037698
C -6.510254 -1.274018 0.506949
C -5.074861 -0.331097 -1.208362
C -6.680248 -2.414788 -0.278146
H -7.012705 -1.172321 1.462749
C -5.255555 -1.476073 -1.977609

H -4.464957 0.488144 -1.574823
C -6.054419 -2.538086 -1.524475
H -7.315553 -3.219577 0.083437
H -4.769641 -1.547732 -2.947561
C -6.219285 -3.784041 -2.361250
H -7.036632 -4.410685 -1.991490
H -6.427189 -3.537712 -3.408701
H -5.303787 -4.389309 -2.347965
H -3.170225 0.667234 3.662604
C 0.870044 -3.066224 -0.145440
C 2.124486 -2.712276 -0.657657
C 0.600789 -4.415583 0.122360
C 3.096049 -3.688609 -0.891712
H 2.338275 -1.667050 -0.867207
C 1.568405 -5.389908 -0.114589
H -0.376962 -4.704135 0.498602
C 2.820339 -5.029008 -0.620280
H 4.066005 -3.399292 -1.287107
H 1.344993 -6.433236 0.092513
H 3.573663 -5.790052 -0.805875
H -3.212801 1.378684 1.164453
Cl -1.702032 -2.268767 -0.799338
H -4.632703 -0.202556 3.236300
H 0.533888 -1.676784 2.124204
89
-3063.45433382
C 4.862696 2.842683 -0.301642
C 6.263424 2.651517 0.295855
C 7.340380 2.318341 -0.749993
C 7.177055 0.951821 -1.457019
C 3.812127 3.123911 0.802727
N 2.514324 3.368735 0.191856
H 6.234241 1.855443 1.051027
H 6.556757 3.569721 0.822967
H 8.320692 2.340582 -0.255978
H 7.362077 3.106748 -1.515391
H 3.807391 2.307223 1.535329
H 4.859647 3.675396 -1.015530
H 4.542961 1.949334 -0.846112
H 6.227235 0.928294 -2.004373
S 1.464273 2.042467 0.313997
O 1.193794 1.773176 1.736338
O 2.014921 0.965015 -0.535637
C -0.023851 2.659671 -0.445955
C -0.176748 2.568195 -1.829241
C -1.028358 3.206623 0.359053
C -1.352382 3.042079 -2.410080
H 0.603373 2.121052 -2.436067
C -2.198727 3.666230 -0.239315
H -0.893483 3.257931 1.434494
C -2.378038 3.596401 -1.632156
H -1.478550 2.970786 -3.487269
H -2.996190 4.067544 0.379219
C -3.651497 4.107430 -2.258302
H -4.524602 3.682835 -1.752285
H -3.719522 5.198954 -2.166423
H -3.703092 3.859232 -3.322886
H 4.083268 4.053076 1.321095
C 7.252888 -0.238786 -0.522195
C 6.095369 -0.897939 -0.085241
C 8.492902 -0.698575 -0.054865
C 6.171744 -1.980516 0.794497
H 5.121687 -0.562249 -0.436608
C 8.575320 -1.779355 0.823052
H 9.404665 -0.207328 -0.389565
C 7.413134 -2.424636 1.251539
H 5.259840 -2.477703 1.114163
H 9.547682 -2.121705 1.168214
H 7.475551 -3.268843 1.933060
H 7.967013 0.869708 -2.214842
C -2.806002 -1.043181 2.386235
C -1.371255 -0.638542 2.012294
C -0.442363 -1.818358 1.642333
C -0.133568 -1.976456 0.150094
C -3.668568 0.143857 2.838451
N -3.966392 1.133014 1.792046
H -0.930010 -0.094346 2.856412
H -1.385156 0.074833 1.178001
H -0.838562 -2.762853 2.035315
H -3.288657 -1.560615 1.549992
H -2.765680 -1.757353 3.221224
H 0.231805 -1.029106 -0.249165
S -5.457719 1.226861 1.049546
O -5.339484 2.368531 0.132840
O -6.462513 1.181519 2.113828
C -5.704114 -0.239621 0.039584
C -6.509958 -1.274498 0.514326
C -5.081176 -0.334938 -1.208337
C -6.681561 -2.417540 -0.267118
H -7.009374 -1.170677 1.471490
C -5.263475 -1.482114 -1.973911
H -4.473375 0.483953 -1.579026
C -6.059871 -2.543612 -1.515235
H -7.314912 -3.221924 0.098766
H -4.780896 -1.555919 -2.945371
C -6.226735 -3.791761 -2.348339
H -6.442988 -3.548141 -3.394754
H -5.309242 -4.394075 -2.340529
H -7.039435 -4.420234 -1.971523
H -3.166128 0.675881 3.657577
C 0.868592 -3.065082 -0.148965
C 2.123093 -2.712261 -0.661807
C 0.598555 -4.414100 0.119766
C 3.093959 -3.689360 -0.895566
H 2.337524 -1.667299 -0.872019
C 1.565458 -5.389194 -0.116923
H -0.379256 -4.701771 0.496524
C 2.817463 -5.029414 -0.623241
H 4.063990 -3.400890 -1.291387
H 1.341440 -6.432253 0.090883
H 3.570236 -5.791059 -0.808605
H -3.212510 1.380371 1.157420
Cl -1.703709 -2.266795 -0.801292
H -4.629223 -0.195079 3.235860
H 0.535223 -1.672920 2.119536
89
-3063.45432832
C 4.859690 2.844131 -0.301802
C 6.260965 2.654393 0.294876
C 7.337799 2.323107 -0.751700
C 7.175964 0.956645 -1.459182
C 3.809317 3.123445 0.803214
N 2.511096 3.367769 0.193051
H 6.233179 1.857915 1.049674
H 6.553418 3.572706 0.822286
H 8.318354 2.346486 -0.258220
H 7.357994 3.111855 -1.516789
H 3.805584 2.306107 1.535102
H 4.855262 3.677245 -1.015219
H 4.540780 1.950696 -0.846623
H 6.225862 0.932019 -2.005999
S 1.461823 2.040901 0.315089
O 1.191250 1.771664 1.737416
O 2.013194 0.963629 -0.534300
C -0.026516 2.657189 -0.445200
C -0.179820 2.564027 -1.828312
C -1.030710 3.205300 0.359435
C -1.355571 3.037383 -2.409399
H 0.600067 2.116031 -2.434804
C -2.201156 3.664387 -0.239147
H -0.895504 3.257943 1.434776
C -2.380887 3.592861 -1.631869
H -1.482045 2.964788 -3.486466
H -2.998389 4.066583 0.379098
C -3.654477 4.103298 -2.258250
H -3.722769 5.194848 -2.166932
H -3.706088 3.854514 -3.322698
H -4.527422 3.678767 -1.751892
H 4.079881 4.052376 1.322318
C 7.253977 -0.234194 -0.524842

C 6.097611 -0.895046 -0.087426
C 8.494881 -0.692447 -0.058363
C 6.175941 -1.977761 0.791970
H 5.123296 -0.560569 -0.438186
C 8.579261 -1.773376 0.819182
H 9.405786 -0.199863 -0.393435
C 7.418193 -2.420341 1.248164
H 5.264888 -2.476231 1.112068
H 9.552282 -2.114523 1.163679
H 7.482141 -3.264647 1.929420
H 7.965606 0.875925 -2.217483
C -2.808634 -1.047882 2.387253
C -1.373150 -0.642682 2.016704
C -0.443646 -1.821836 1.646200
C -0.133420 -1.978401 0.154096
C -3.671670 0.138194 2.841086
N -3.967849 1.130029 1.796748
H -0.933425 -0.100298 2.862798
H -1.385331 0.072429 1.183872
H -0.839911 -2.766820 2.037938
H -3.290038 -1.563145 1.548963
H -2.769841 -1.764136 3.220531
H 0.231939 -1.030554 -0.243978
S -5.457935 1.225512 1.051948
O -5.338457 2.369829 0.138705
O -6.464588 1.176892 2.114324
C -5.702060 -0.238222 0.037458
C -6.510256 -1.273680 0.506916
C -5.075078 -0.330859 -1.208633
C -6.680180 -2.414584 -0.278003
H -7.012658 -1.171892 1.462731
C -5.255708 -1.475958 -1.977706
H -4.465292 0.488400 -1.575249
C -6.054418 -2.537996 -1.524357
H -7.315377 -3.219388 0.083740
H -4.769870 -1.547710 -2.947691
C -6.219175 -3.784106 -2.360923
H -6.426742 -3.537974 -3.408486
H -5.303744 -4.389469 -2.347240
H -7.036696 -4.410589 -1.991272
H -3.170377 0.668127 3.662268
C 0.869408 -3.066365 -0.145133
C 2.123815 -2.712709 -0.657638
C 0.599984 -4.415636 0.122951
C 3.095178 -3.689239 -0.891698
H 2.337743 -1.667554 -0.867399
C 1.567398 -5.390158 -0.114006
H -0.377746 -4.703967 0.499419
C 2.819298 -5.029547 -0.619988
H 4.065113 -3.400143 -1.287305
H 1.343854 -6.433415 0.093314
H 3.572465 -5.790745 -0.805589
H -3.212931 1.379087 1.164012
Cl -1.702519 -2.268490 -0.799151
H -4.632976 -0.201570 3.236206
H 0.533485 -1.676621 2.124398
89
-3063.45432777
C 4.859738 2.844207 -0.301866
C 6.260976 2.654521 0.294910
C 7.337898 2.323308 -0.751597
C 7.176162 0.956877 -1.459168
C 3.809279 3.123563 0.803074
N 2.511084 3.367667 0.192798
H 6.233166 1.858023 1.049687
H 6.553354 3.572832 0.822365
H 8.318415 2.346680 -0.258039
H 7.358135 3.112094 -1.516644
H 3.805603 2.306322 1.535070
H 4.855332 3.677277 -1.015333
H 4.540880 1.950731 -0.846651
H 6.226109 0.932253 -2.006073
S 1.461896 2.040726 0.315064
O 1.191390 1.771634 1.737431
O 2.013332 0.963404 -0.534218
C -0.026498 2.656846 -0.445248
C -0.179870 2.563461 -1.828340
C -1.030623 3.205142 0.359342
C -1.355615 3.036791 -2.409450
H 0.599972 2.115321 -2.434785
C -2.201069 3.664205 -0.239265
H -0.895375 3.257952 1.434669
C -2.380857 3.592476 -1.631965
H -1.482146 2.964032 -3.486500
H -2.998248 4.066551 0.378954
C -3.654420 4.102942 -2.258385
H -3.722504 5.194534 -2.167403
H -3.706178 3.853836 -3.322749
H -4.527383 3.678723 -1.751802
H 4.079734 4.052590 1.322062
C 7.254135 -0.234011 -0.524888
C 8.495036 -0.692432 -0.058577
C 6.097735 -0.894735 -0.087349
C 8.579392 -1.773400 0.818929
H 9.405961 -0.199952 -0.393747
C 6.176043 -1.977482 0.792004
H 5.123419 -0.560148 -0.437999
C 7.418299 -2.420229 1.248037
H 9.552416 -2.114676 1.163289
H 5.264971 -2.475855 1.112204
H 7.482220 -3.264563 1.929261
H 7.965878 0.876227 -2.217398
C -2.808619 -1.047893 2.387323
C -1.373141 -0.642733 2.016715
C -0.443672 -1.821932 1.646248
C -0.133383 -1.978497 0.154158
C -3.671646 0.138195 2.841145
N -3.967856 1.129988 1.796788
H -0.933383 -0.100306 2.862764
H -1.385340 0.072327 1.183838
H -0.840016 -2.766904 2.037939
H -3.290059 -1.563169 1.549061
H -2.769811 -1.764127 3.220618
H 0.232012 -1.030658 -0.243904
S -5.457993 1.225603 1.052136
O -5.338579 2.370015 0.139003
O -6.464564 1.176885 2.114585
C -5.702216 -0.238015 0.037503
C -6.510583 -1.273413 0.506790
C -5.075143 -0.330613 -1.208548
C -6.680601 -2.414214 -0.278263
H -7.013035 -1.171663 1.462583
C -5.255861 -1.475608 -1.977750
H -4.465215 0.488598 -1.575039
C -6.054759 -2.537583 -1.524579
H -7.315933 -3.218969 0.083349
H -4.769934 -1.547324 -2.947693
C -6.219633 -3.783578 -2.361295
H -7.037231 -4.410013 -1.991734
H -6.427153 -3.537298 -3.408833
H -5.304271 -4.389046 -2.347666
H -3.170355 0.668144 3.662319
C 0.869445 -3.066479 -0.145028
C 2.123910 -2.712825 -0.657396
C 0.599964 -4.415754 0.122969
C 3.095272 -3.689366 -0.891409
H 2.337880 -1.667666 -0.867093
C 1.567382 -5.390287 -0.113937
H -0.377809 -4.704081 0.499326
C 2.819338 -5.029681 -0.619783
H 4.065245 -3.400275 -1.286924
H 1.343795 -6.433547 0.093317
H 3.572506 -5.790887 -0.805347
H -3.212966 1.379128 1.164054
Cl -1.702445 -2.268568 -0.799132
H -4.632943 -0.201583 3.236274
H 0.533431 -1.676788 2.124528
89
-3063.45432758
C 4.859553 2.844040 -0.302041

C 6.260814 2.654424 0.294695
C 7.337725 2.323194 -0.751822
C 7.176031 0.956708 -1.459295
C 3.809153 3.123512 0.802924
N 2.510956 3.367762 0.192703
H 6.233059 1.857967 1.049516
H 6.553177 3.572775 0.822089
H 8.318252 2.346649 -0.258291
H 7.357902 3.111935 -1.516918
H 3.805417 2.306286 1.534936
H 4.855111 3.677044 -1.015585
H 4.540659 1.950514 -0.846726
H 6.225955 0.931989 -2.006153
S 1.461615 2.040961 0.315045
O 1.191223 1.771834 1.737427
O 2.012835 0.963610 -0.534341
C -0.026793 2.657283 -0.445078
C -0.180155 2.564343 -1.828206
C -1.030953 3.205246 0.359686
C -1.355929 3.037783 -2.409161
H 0.599715 2.116454 -2.434798
C -2.201438 3.664415 -0.238771
H -0.895701 3.257727 1.435029
C -2.381222 3.593126 -1.631490
H -1.482454 2.965380 -3.486235
H -2.998642 4.066502 0.379584
C -3.654857 4.103639 -2.257725
H -4.527738 3.678792 -1.751525
H -3.723304 5.195143 -2.165992
H -3.706418 3.855238 -3.322263
H 4.079727 4.052520 1.321888
C 7.254112 -0.234103 -0.524925
C 6.097785 -0.894986 -0.087450
C 8.495048 -0.692292 -0.058467
C 6.176183 -1.977661 0.791989
H 5.123445 -0.560577 -0.438210
C 8.579497 -1.773183 0.819119
H 9.405923 -0.199686 -0.393588
C 7.418466 -2.420172 1.248166
H 5.265160 -2.476155 1.112135
H 9.552541 -2.114279 1.163598
H 7.482468 -3.264447 1.929457
H 7.965714 0.876043 -2.217558
C -2.808504 -1.047969 2.387458
C -1.373080 -0.642616 2.016844
C -0.443475 -1.821698 1.646363
C -0.133333 -1.978325 0.154246
C -3.671705 0.138019 2.841210
N -3.968028 1.129715 1.796790
H -0.933385 -0.100148 2.862900
H -1.385364 0.072452 1.183977
H -0.839624 -2.766696 2.038188
H -3.289860 -1.563364 1.549220
H -2.769590 -1.764150 3.220794
H 0.231992 -1.030488 -0.243884
S -5.458157 1.225068 1.052084
O -5.338972 2.369591 0.139064
O -6.464744 1.176011 2.114503
C -5.702005 -0.238499 0.037290
C -6.509974 -1.274234 0.506533
C -5.075013 -0.330740 -1.208821
C -6.679661 -2.415004 -0.278629
H -7.012383 -1.172763 1.462379
C -5.255396 -1.475716 -1.978137
H -4.465404 0.488723 -1.575275
C -6.053877 -2.538019 -1.525015
H -7.314685 -3.220020 0.082943
H -4.769526 -1.547147 -2.948129
C -6.218375 -3.783995 -2.361833
H -7.035924 -4.410601 -1.992451
H -6.425749 -3.537697 -3.409394
H -5.302906 -4.389300 -2.348067
H -3.170495 0.668082 3.662361
C 0.869512 -3.066278 -0.144986
C 2.123899 -2.712601 -0.657527
C 0.600124 -4.415550 0.123121
C 3.095277 -3.689111 -0.891604
H 2.337791 -1.667445 -0.867319
C 1.567555 -5.390055 -0.113849
H -0.377589 -4.703898 0.499619
C 2.819433 -5.029422 -0.619870
H 4.065192 -3.399999 -1.287247
H 1.344038 -6.433314 0.093488
H 3.572610 -5.790608 -0.805486
H -3.213150 1.378919 1.164069
Cl -1.702456 -2.268490 -0.798901
H -4.632961 -0.201865 3.236342
H 0.533669 -1.676354 2.124501
89
-3063.45432608
C 4.859650 2.844016 -0.301319
C 6.260955 2.654107 0.295227
C 7.337683 2.323013 -0.751519
C 7.175771 0.956676 -1.459225
C 3.809377 3.123411 0.803802
N 2.511158 3.367871 0.193727
H 6.233198 1.857469 1.049857
H 6.553482 3.572297 0.822811
H 8.318298 2.346306 -0.258153
H 7.357782 3.111899 -1.516467
H 3.805626 2.306044 1.535658
H 4.855243 3.677165 -1.014694
H 4.540595 1.950637 -0.846145
H 6.225502 0.932056 -2.005753
S 1.461875 2.040955 0.315548
O 1.191033 1.771776 1.737836
O 2.013416 0.963662 -0.533692
C -0.026314 2.657197 -0.445067
C -0.179298 2.564025 -1.828217
C -1.030711 3.205263 0.359338
C -1.354937 3.037316 -2.409567
H 0.600747 2.116059 -2.434528
C -2.201051 3.664284 -0.239512
H -0.895753 3.257923 1.434708
C -2.380463 3.592733 -1.632267
H -1.481168 2.964716 -3.486663
H -2.998439 4.066440 0.378558
C -3.653947 4.103068 -2.258956
H -3.705252 3.854315 -3.323425
H -4.526982 3.678429 -1.752844
H -3.722374 5.194606 -2.167612
H 4.080094 4.052299 1.322897
C 7.254220 -0.234339 -0.525149
C 6.098062 -0.895202 -0.087189
C 8.495358 -0.692755 -0.059461
C 6.176837 -1.978078 0.791968
H 5.123549 -0.560632 -0.437313
C 8.580181 -1.773850 0.817842
H 9.406101 -0.200166 -0.394965
C 7.419323 -2.420820 1.247374
H 5.265937 -2.476548 1.112504
H 9.553383 -2.115110 1.161714
H 7.483605 -3.265254 1.928441
H 7.965186 0.876185 -2.217786
C -2.808946 -1.047773 2.387143
C -1.373398 -0.642567 2.016836
C -0.443905 -1.821748 1.646383
C -0.133342 -1.978079 0.154322
C -3.672097 0.138293 2.840785
N -3.968085 1.130059 1.796336
H -0.933784 -0.100246 2.863028
H -1.385436 0.072595 1.184047
H -0.840382 -2.766763 2.037839
H -3.290191 -1.563057 1.548770
H -2.770281 -1.764014 3.220437
H 0.232202 -1.030200 -0.243506
S -5.458121 1.225666 1.051449
O -5.338355 2.369723 0.137912
O -6.464845 1.177531 2.113776
C -5.702480 -0.238291 0.037341

C -6.511287 -1.273258 0.506817
C -5.075067 -0.331585 -1.208488
C -6.681423 -2.414326 -0.277827
H -7.013972 -1.170973 1.462431
C -5.255893 -1.476843 -1.977271
H -4.464772 0.487285 -1.575126
C -6.055252 -2.538390 -1.523906
H -7.317098 -3.218746 0.083928
H -4.769635 -1.549110 -2.947006
C -6.220269 -3.784657 -2.360187
H -6.427957 -3.538714 -3.407772
H -5.304908 -4.390126 -2.346519
H -7.037815 -4.410964 -1.990295
H -3.170988 0.668257 3.662060
C 0.869460 -3.066083 -0.144876
C 2.124019 -2.712432 -0.657016
C 0.599855 -4.415381 0.122876
C 3.095352 -3.688998 -0.891044
H 2.338079 -1.667255 -0.866530
C 1.567243 -5.389940 -0.114048
H -0.377987 -4.703704 0.499058
C 2.819292 -5.029336 -0.619664
H 4.065401 -3.399908 -1.286374
H 1.343559 -6.433219 0.093011
H 3.572437 -5.790563 -0.805240
H -3.213110 1.378963 1.163609
Cl -1.702252 -2.267849 -0.799293
H -4.633468 -0.201503 3.235722
H 0.533102 -1.676738 2.124899
89
-3063.45432515
C 4.859615 2.844173 -0.301914
C 6.260921 2.654525 0.294715
C 7.337742 2.323249 -0.751876
C 7.175937 0.956777 -1.459348
C 3.809284 3.123556 0.803151
N 2.511030 3.367774 0.193068
H 6.233200 1.858082 1.049553
H 6.553346 3.572878 0.822072
H 8.318306 2.346656 -0.258412
H 7.357910 3.111993 -1.516969
H 3.805662 2.306267 1.535097
H 4.855120 3.677227 -1.015400
H 4.540692 1.950677 -0.846629
H 6.225853 0.932129 -2.006197
S 1.461803 2.040867 0.315356
O 1.191158 1.772064 1.737746
O 2.013260 0.963374 -0.533679
C -0.026501 2.656870 -0.445224
C -0.179812 2.563013 -1.828296
C -1.030666 3.205439 0.359128
C -1.355537 3.036119 -2.409619
H 0.600064 2.114661 -2.434540
C -2.201097 3.664269 -0.239688
H -0.895488 3.258596 1.434448
C -2.380832 3.592044 -1.632367
H -1.482031 2.962975 -3.486648
H -2.998309 4.066814 0.378360
C -3.654377 4.102215 -2.259045
H -3.722421 5.193861 -2.168650
H -3.706140 3.852544 -3.323278
H -4.527382 3.678311 -1.752264
H 4.079868 4.052535 1.322160
C 7.253936 -0.234053 -0.524989
C 8.494832 -0.692384 -0.058568
C 6.097547 -0.894815 -0.087499
C 8.579179 -1.773302 0.818999
H 9.405757 -0.199877 -0.393698
C 6.175839 -1.977509 0.791926
H 5.123252 -0.560263 -0.438236
C 7.418086 -2.420168 1.248065
H 9.552193 -2.114513 1.163451
H 5.264766 -2.475896 1.112098
H 7.482010 -3.264460 1.929342
H 7.965606 0.876057 -2.217620
C -2.808486 -1.047402 2.387556
C -1.373113 -0.642301 2.016473
C -0.443721 -1.821638 1.646259
C -0.133434 -1.978389 0.154177
C -3.671465 0.138790 2.841180
N -3.967740 1.130358 1.796613
H -0.933171 -0.099473 2.862166
H -1.385593 0.072371 1.183272
H -0.840177 -2.766528 2.038024
H -3.290056 -1.562994 1.549556
H -2.769460 -1.763350 3.221087
H 0.232087 -1.030628 -0.243953
S -5.457873 1.225736 1.051959
O -5.338604 2.370064 0.138692
O -6.464431 1.177020 2.114416
C -5.701905 -0.238019 0.037473
C -6.509864 -1.273627 0.507013
C -5.075075 -0.330520 -1.208702
C -6.679701 -2.414540 -0.277912
H -7.012160 -1.171948 1.462896
C -5.255612 -1.475631 -1.977782
H -4.465479 0.488848 -1.575388
C -6.054082 -2.537815 -1.524356
H -7.314718 -3.219457 0.083894
H -4.769905 -1.547268 -2.947844
C -6.218730 -3.783950 -2.360908
H -6.425860 -3.537856 -3.408563
H -5.303406 -4.389470 -2.346833
H -7.036487 -4.410279 -1.991515
H -3.170084 0.668930 3.662175
C 0.869221 -3.066538 -0.144940
C 2.123730 -2.713106 -0.657357
C 0.599560 -4.415758 0.123164
C 3.094952 -3.689797 -0.891327
H 2.337845 -1.667990 -0.867125
C 1.566834 -5.390446 -0.113701
H -0.378240 -4.703924 0.499575
C 2.818827 -5.030055 -0.619609
H 4.064961 -3.400862 -1.286870
H 1.343103 -6.433660 0.093630
H 3.571875 -5.791387 -0.805153
H -3.212853 1.379423 1.163848
Cl -1.702580 -2.268280 -0.799038
H -4.632735 -0.200861 3.236477
H 0.533381 -1.676524 2.124539

89
-3063.45658419
C -4.077453 0.791078 2.644901
C -4.823503 0.539779 1.330369
C -5.426636 -0.865327 1.234728
C -6.192129 -1.123584 -0.085096
C -3.534467 2.230564 2.755089
N -2.464812 2.435417 1.788421
H -5.621862 1.287641 1.216711
H -4.133394 0.703639 0.495784
H -6.123501 -1.020963 2.070780
H -4.633202 -1.617205 1.346357
H -3.061859 2.355634 3.742043
H -3.240389 0.090096 2.745388
H -4.745521 0.627284 3.501858
H -6.740410 -2.069370 0.010306
S -2.672117 3.812062 0.825335
O -2.707055 5.009491 1.679151
O -3.794126 3.528108 -0.086893
C -1.153140 3.824596 -0.113331
C -0.199599 4.806647 0.150337
C -0.955078 2.873541 -1.116864
C 0.974278 4.827750 -0.602013
H -0.386061 5.543657 0.923943
C 0.225556 2.908768 -1.852729
H -1.711293 2.122636 -1.319436

C 1.208413 3.879992 -1.607325
H 1.716210 5.599100 -0.409276
H 0.385869 2.172060 -2.636056
C 2.497621 3.886784 -2.393457
H 3.248249 3.242119 -1.917111
H 2.348111 3.515082 -3.412520
H 2.922961 4.893685 -2.457792
H -4.348995 2.962820 2.682997
C -5.311213 -1.187698 -1.316657
C -4.780680 -2.412566 -1.746987
C -4.989896 -0.035488 -2.049437
C -3.949627 -2.487651 -2.866722
H -5.034435 -3.321421 -1.204369
C -4.157804 -0.103953 -3.168494
H -5.393608 0.925873 -1.741659
C -3.633215 -1.330232 -3.581495
H -3.561434 -3.451196 -3.187793
H -3.928314 0.802587 -3.722863
H -2.994620 -1.385973 -4.459313
H -6.948092 -0.337411 -0.213248
Cl -0.652129 -0.559358 0.869975
C 2.373637 -3.377525 -1.555853
C 0.905844 -3.197427 -1.983330
C 0.146797 -2.041966 -1.297818
C 0.187533 -2.088549 0.235630
C 3.254656 -2.144427 -1.800637
N 4.619023 -2.241358 -1.252328
H 0.376924 -4.138313 -1.790854
H 0.854225 -3.033140 -3.068874
H -0.895563 -2.051211 -1.636024
H 0.557690 -1.076399 -1.614922
H 3.356691 -1.949945 -2.875706
H 2.424199 -3.638539 -0.494821
H 2.799094 -4.228679 -2.106340
H 1.211428 -1.973263 0.591683
S 4.856958 -1.915197 0.390356
O 6.107741 -2.602412 0.726698
O 3.627518 -2.163153 1.162909
C 5.128051 -0.144373 0.407059
C 4.117415 0.707627 0.850023
C 6.363466 0.358861 -0.010718
C 4.348334 2.084619 0.861764
H 3.171865 0.300269 1.191178
C 6.577194 1.732652 0.013502
H 7.143858 -0.319951 -0.338545
C 5.576501 2.617012 0.450049
H 3.560450 2.750838 1.203883
H 7.538080 2.128326 -0.306636
C 5.837412 4.103516 0.503707
H 6.430297 4.365326 1.389780
H 6.399853 4.444286 -0.372527
H 4.904386 4.673171 0.554087
H 2.805525 -1.245399 -1.368581
C -0.417732 -3.317900 0.866813
C -1.720532 -3.740529 0.562763
C 0.345494 -4.067641 1.771361
C -2.242957 -4.891686 1.149308
H -2.332404 -3.159850 -0.122490
C -0.177977 -5.222929 2.356035
H 1.353819 -3.742311 2.016589
C -1.472989 -5.637685 2.046512
H -3.254707 -5.207942 0.908247
H 0.426810 -5.794211 3.055224
H -1.883417 -6.534874 2.502384
H 5.103669 -3.111915 -1.465577
89
-3063.45429222
C -0.254834 5.115957 1.348409
C -1.225179 4.086646 0.746948
C -1.136702 3.940672 -0.777993
C -2.171356 2.953477 -1.364608
C 1.228799 4.795516 1.122265
N 1.573783 3.532553 1.758450
H -1.040995 3.110605 1.216337
H -2.246387 4.374487 1.025223
H -0.137526 3.586710 -1.062423
H -1.269904 4.924179 -1.251995
H 1.855971 5.567600 1.600249
H -0.432393 5.191706 2.428049
H -0.448800 6.110261 0.922695
H -1.939082 2.804472 -2.427598
S 2.935679 2.793487 1.063594
O 4.105668 3.654831 1.296498
O 2.567527 2.416334 -0.316415
C 3.099348 1.314122 2.043113
C 4.003092 1.300860 3.105789
C 2.337967 0.187636 1.723393
C 4.133547 0.137982 3.863072
H 4.599317 2.180695 3.322383
C 2.486760 -0.964363 2.489299
H 1.651637 0.193105 0.884987
C 3.378766 -1.006614 3.571644
H 4.838827 0.118066 4.690137
H 1.904577 -1.843110 2.226137
C 3.509697 -2.257064 4.407649
H 2.655001 -2.364392 5.088157
H 3.536092 -3.154857 3.780369
H 4.417863 -2.240074 5.018139
H 1.489472 4.806691 0.055357
C -3.612967 3.401034 -1.227175
C -4.130952 4.397119 -2.068632
C -4.459143 2.847098 -0.256824
C -5.450532 4.830602 -1.943235
H -3.492539 4.833148 -2.834897
C -5.781728 3.278157 -0.127796
H -4.080334 2.068047 0.401140
C -6.282003 4.271888 -0.969567
H -5.831925 5.601193 -2.608697
H -6.420378 2.833415 0.631265
H -7.311486 4.606472 -0.871709
H -2.042271 1.978541 -0.876375
Cl -3.151597 -0.583426 1.865030
C -2.037213 -2.805452 -2.279103
C -3.479322 -2.453845 -1.873820
C -3.632410 -1.481191 -0.685463
C -2.865879 -1.895840 0.577585
C -1.169061 -1.592547 -2.638966
N 0.235894 -1.903972 -2.973268
H -4.003886 -3.388176 -1.641810
H -4.005165 -2.011048 -2.730996
H -4.697725 -1.387719 -0.445565
H -3.298132 -0.477089 -0.969182
H -1.575891 -1.069378 -3.512937
H -1.553504 -3.365214 -1.471188
H -2.070548 -3.488137 -3.139622
H -1.789845 -1.841816 0.406163
S 1.226349 -2.461986 -1.711601
O 1.342970 -3.927238 -1.756797
O 0.724106 -1.804130 -0.490570
C 2.819248 -1.773659 -2.139642
C 3.036569 -0.399164 -2.005085
C 3.835089 -2.627162 -2.567079
C 4.290657 0.117522 -2.308973
H 2.252679 0.261807 -1.654060
C 5.084682 -2.087381 -2.875177
H 3.649156 -3.692946 -2.643945
C 5.331806 -0.714656 -2.753052
H 4.459451 1.183565 -2.183633
H 5.881555 -2.747832 -3.207840
C 6.683879 -0.132099 -3.088058
H 6.624344 0.524529 -3.965210
H 7.070235 0.474012 -2.260392
H 7.416635 -0.914937 -3.306150
H -1.137395 -0.869648 -1.821564
C -3.217478 -3.251234 1.138449
C -4.539153 -3.601410 1.451542
C -2.202519 -4.196677 1.333486
C -4.837141 -4.870830 1.941989
H -5.334062 -2.870771 1.328322
C -2.501205 -5.470917 1.820682

H -1.173813 -3.932305 1.099748
C -3.818777 -5.810817 2.125742
H -5.864964 -5.127621 2.184474
H -1.702527 -6.193859 1.962955
H -4.053508 -6.800705 2.507904
H 0.320858 -2.556070 -3.752651
89
-3063.45404621
C 0.174513 2.866333 -3.622758
C -0.089096 3.631961 -2.321046
C 1.075580 4.554214 -1.940161
C 0.818926 5.414275 -0.680992
C -1.032422 2.029520 -4.099247
N -1.213094 0.856505 -3.262244
H -1.003157 4.234103 -2.436436
H -0.290434 2.919756 -1.512921
H 1.281398 5.234874 -2.778987
H 1.985194 3.957055 -1.791581
H -0.797678 1.630115 -5.099293
H 1.039929 2.203732 -3.506250
H 0.411737 3.572616 -4.430488
H 1.598990 6.184257 -0.625882
S -2.744166 0.714003 -2.544900
O -3.822558 1.175890 -3.432758
O -2.592559 1.341482 -1.214952
C -2.871550 -1.055428 -2.368154
C -3.919415 -1.721823 -3.002636
C -1.953827 -1.742565 -1.566848
C -4.045092 -3.100022 -2.828902
H -4.620880 -1.165020 -3.614292
C -2.097800 -3.115860 -1.406166
H -1.142677 -1.223423 -1.069930
C -3.141572 -3.816125 -2.033520
H -4.861858 -3.625484 -3.317367
H -1.391288 -3.646051 -0.773060
C -3.272707 -5.309723 -1.856980
H -4.229934 -5.677514 -2.238940
H -2.474735 -5.840712 -2.391905
H -3.194601 -5.592954 -0.801231
H -1.934117 2.647530 -4.195796
C 0.803594 4.642176 0.622708
C 1.990506 4.444816 1.343195
C -0.381887 4.103361 1.142020
C 1.995104 3.736089 2.545520
H 2.920114 4.861755 0.961118
C -0.383490 3.388790 2.341474
H -1.314895 4.245632 0.602016
C 0.805349 3.204962 3.049817
H 2.925334 3.611364 3.094862
H -1.313902 2.977203 2.722752
H 0.801781 2.661577 3.991205
H -0.133612 5.946953 -0.804805
Cl 3.248160 0.955907 -1.675374
C 3.076809 -1.375244 2.592832
C 4.269955 -0.538817 2.102449
C 4.007217 0.390031 0.899001
C 3.408598 -0.313915 -0.326846
C 1.883142 -0.556051 3.098279
N 0.758126 -1.383753 3.569891
H 5.083942 -1.227664 1.847772
H 4.643269 0.084590 2.927139
H 4.955440 0.859805 0.612497
H 3.333797 1.207873 1.179358
H 2.182522 0.080745 3.940247
H 2.732432 -2.043168 1.796640
H 3.423839 -2.024054 3.410283
H 2.375657 -0.604830 -0.130138
S -0.339019 -2.015990 2.448117
O -0.597456 -3.393710 2.887084
O 0.144831 -1.732052 1.083912
C -1.834525 -1.064529 2.697266
C -2.174340 -0.049193 1.805779
C -2.661672 -1.379355 3.780305
C -3.361655 0.658038 2.003630
H -1.541643 0.185865 0.958970
C -3.833959 -0.655482 3.968023
H -2.395465 -2.187455 4.453636
C -4.205082 0.370722 3.082986
H -3.631471 1.427143 1.285754
H -4.479360 -0.896669 4.809462
C -5.501996 1.119410 3.277592
H -5.671975 1.359289 4.333246
H -5.513801 2.053800 2.708587
H -6.356859 0.518286 2.941040
H 1.495702 0.111787 2.325512
C 4.176190 -1.506202 -0.841864
C 5.537726 -1.423328 -1.168732
C 3.520372 -2.735925 -0.981704
C 6.226884 -2.547325 -1.618781
H 6.054162 -0.470594 -1.087648
C 4.211433 -3.863788 -1.429720
H 2.463489 -2.807742 -0.735392
C 5.565839 -3.772248 -1.748895
H 7.280776 -2.468142 -1.872251
H 3.688465 -4.810891 -1.531747
H 6.105005 -4.647757 -2.100776
H 1.036425 -2.148843 4.181555
89
-3063.45335823
C -2.930143 -2.788502 -0.981889
C -4.391168 -2.319833 -1.097411
C -4.851032 -1.364877 0.012352
C -6.268669 -0.811573 -0.228339
C -1.887280 -1.737286 -1.399525
N -1.772540 -0.674097 -0.423718
H -5.040226 -3.206765 -1.098254
H -4.537335 -1.842705 -2.078988
H -4.153046 -0.525661 0.100883
H -4.820403 -1.886561 0.978641
H -0.894560 -2.221801 -1.426451
H -2.710103 -3.120430 0.039673
H -2.773832 -3.655812 -1.636334
H -6.281121 -0.296121 -1.199632
S -1.405538 0.895634 -0.932110
O -0.318010 1.335843 -0.038822
O -2.691518 1.609070 -0.924127
C -0.776732 0.902921 -2.608045
C 0.580352 0.656028 -2.838867
C -1.658079 1.162413 -3.661215
C 1.045811 0.667448 -4.151615
H 1.271172 0.489153 -2.017803
C -1.167740 1.167687 -4.965477
H -2.700696 1.379485 -3.454415
C 0.185682 0.916039 -5.231980
H 2.102726 0.491371 -4.334607
H -1.845847 1.379561 -5.788289
C 0.706939 0.899937 -6.648699
H 0.039153 1.437028 -7.329294
H 1.700609 1.355783 -6.713509
H 0.798516 -0.129091 -7.020546
H -2.085193 -1.373056 -2.418231
C -6.731272 0.141636 0.854831
C -6.113936 1.391978 1.015304
C -7.769990 -0.203847 1.727750
C -6.526664 2.267743 2.018949
H -5.298966 1.677727 0.353577
C -8.186678 0.670999 2.733653
H -8.260323 -1.169008 1.616794
C -7.565854 1.911183 2.882174
H -6.035840 3.231783 2.126681
H -8.996747 0.382408 3.398976
H -7.888046 2.595120 3.663166
H -6.979231 -1.644826 -0.312609
Cl 0.274580 -2.884178 1.394542
C 1.391887 -0.572501 3.675282
C 2.227236 -1.855859 3.862567
C 2.796934 -2.549017 2.606480
C 1.914707 -3.594988 1.919432
C 2.114675 0.596445 2.985959
N 2.149737 0.424206 1.523857

H 1.629565 -2.577107 4.438041
H 3.081140 -1.598787 4.504466
H 3.693027 -3.113028 2.903510
H 3.134960 -1.806711 1.882446
H 1.620203 1.543153 3.245542
H 0.460913 -0.784367 3.141235
H 1.100245 -0.223821 4.675581
H 1.620048 -4.360499 2.641639
S 3.423939 1.010540 0.631374
O 3.121406 0.635060 -0.759402
O 4.656113 0.550737 1.281152
C 3.412198 2.806830 0.736632
C 2.314927 3.527234 0.252127
C 4.504881 3.461858 1.302473
C 2.326019 4.915616 0.342181
H 1.460475 3.012555 -0.176450
C 4.498648 4.855398 1.379148
H 5.343442 2.882013 1.672298
C 3.415826 5.602581 0.901552
H 1.471630 5.477913 -0.027382
H 5.350669 5.368114 1.818966
C 3.417333 7.111289 0.970208
H 4.220240 7.482905 1.614226
H 3.560092 7.551849 -0.025071
H 2.466467 7.494671 1.357722
H 3.150947 0.657968 3.332858
C 2.573658 -4.245256 0.726831
C 3.066622 -3.479927 -0.341305
C 2.723537 -5.636329 0.689820
C 3.700300 -4.100114 -1.416167
H 2.948946 -2.399619 -0.337960
C 3.360979 -6.257269 -0.386165
H 2.339641 -6.238775 1.510255
C 3.851337 -5.489505 -1.442032
H 4.080845 -3.495239 -2.234839
H 3.471510 -7.338416 -0.397789
H 4.348397 -5.969054 -2.281219
H 1.257866 0.563030 1.043825
89
-3063.44964078
C 5.354430 1.731998 2.379222
C 5.897353 0.558374 1.557533
C 7.368524 0.754605 1.170595
C 7.985717 -0.434597 0.403513
C 3.861304 1.594430 2.785920
N 2.991142 1.565485 1.618717
H 5.792405 -0.371610 2.137047
H 5.292898 0.430150 0.652564
H 7.966494 0.926940 2.076828
H 7.462323 1.666223 0.564683
H 3.595487 2.395366 3.486489
H 5.473436 2.672186 1.830220
H 5.922149 1.829340 3.314813
H 9.052101 -0.223697 0.244579
S 2.325859 3.090628 1.249825
O 3.408882 3.853261 0.603054
O 1.646627 3.632620 2.436401
C 1.096396 2.677051 0.028795
C 1.487748 2.467829 -1.295484
C -0.245390 2.596493 0.407420
C 0.516025 2.160974 -2.244254
H 2.531549 2.559291 -1.576836
C -1.203145 2.290507 -0.557639
H -0.529142 2.788496 1.436669
C -0.839960 2.063559 -1.894739
H 0.813069 1.999808 -3.277314
H -2.249925 2.232773 -0.270006
C -1.880767 1.698508 -2.923449
H -2.829982 2.208233 -2.726383
H -1.552222 1.958852 -3.934817
H -2.075187 0.619648 -2.895176
H 3.721789 0.629013 3.290044
C 7.328807 -0.731747 -0.930399
C 7.420677 0.178875 -1.993687
C 6.614994 -1.919102 -1.138461
C 6.812586 -0.085122 -3.220605
H 7.978834 1.103297 -1.860347
C 6.004212 -2.189538 -2.365439
H 6.545778 -2.645900 -0.331582
C 6.099533 -1.271235 -3.410970
H 6.899107 0.634132 -4.031091
H 5.457048 -3.118592 -2.503392
H 5.626629 -1.478523 -4.367121
H 7.941089 -1.330686 1.036210
Cl -4.447002 0.942097 1.660261
C -4.441807 -2.325104 2.611795
C -5.896668 -2.077789 2.161255
C -6.159726 -1.197865 0.921503
C -6.124130 0.324064 1.085346
C -3.509763 -2.993443 1.590259
N -3.228426 -2.090745 0.463019
H -6.453238 -1.671833 3.018050
H -6.347603 -3.057189 1.950743
H -7.187932 -1.398184 0.586426
H -5.506680 -1.500229 0.100740
H -2.582400 -3.297104 2.100968
H -3.982530 -1.396829 2.965899
H -4.478592 -2.993817 3.482053
H -6.771389 0.629257 1.910722
S -2.271044 -2.596595 -0.814730
O -2.293167 -1.475739 -1.762378
O -2.741229 -3.933964 -1.182365
C -0.593243 -2.754791 -0.197514
C -0.104137 -4.013296 0.152095
C 0.195928 -1.609484 -0.043204
C 1.190760 -4.119929 0.661971
H -0.722458 -4.892111 0.003538
C 1.482302 -1.733077 0.472103
H -0.184955 -0.637233 -0.339509
C 1.998358 -2.989237 0.832734
H 1.579338 -5.100571 0.925732
H 2.093612 -0.841596 0.592934
C 3.397105 -3.106094 1.389495
H 3.691022 -4.152002 1.521808
H 4.127033 -2.626686 0.726752
H 3.476448 -2.610920 2.365759
H -3.969466 -3.895868 1.175785
C -6.500910 1.068228 -0.171547
C -5.832470 0.836323 -1.383957
C -7.553293 1.990251 -0.141429
C -6.219790 1.512271 -2.539321
H -5.001215 0.137194 -1.424839
C -7.942879 2.664679 -1.300246
H -8.072199 2.182330 0.795050
C -7.276673 2.426604 -2.501887
H -5.696607 1.322108 -3.472506
H -8.763216 3.376179 -1.260797
H -7.576298 2.950836 -3.405327
H -3.020342 -1.128275 0.724193

N-H

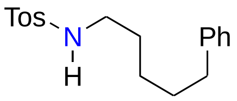

Name	E(B3LYP)	G(B3LYP)	E(RO-B2PLYP-D3)	G(RO-B2PLYP-D3)
NH.conf15	-1303.10330570	-1302.778657	-1302.5831192072	-1302.2584705072
NH.conf08	-1303.10257964	-1302.777386	-1302.5831872962	-1302.2579936562
NH.conf73	-1303.10493032	-1302.782663	-1302.5798361191	-1302.2575687991
NH.conf03	-1303.10277256	-1302.779294	-1302.5806747738	-1302.2571962138
NH.conf48	-1303.10345681	-1302.780640	-1302.5797214200	-1302.2569046100
NH.conf61	-1303.10277105	-1302.778582	-1302.5806793849	-1302.2564903349
NH.conf94	-1303.10406537	-1302.781520	-1302.5786777677	-1302.2561323977
NH.conf84	-1303.10427140	-1302.781333	-1302.5786506532	-1302.2557122532
NH.conf28	-1303.10302977	-1302.778440	-1302.5797963369	-1302.2552065669
NH.conf59	-1303.10182835	-1302.775976	-1302.5804735078	-1302.2546211578

45
-1302.25847050720000 NH.conf15
H 1.370625 -2.076475 1.152152
C 2.092476 -2.425601 -0.820792
H 3.049714 -2.192621 -1.295165
H 2.044751 -3.518341 -0.728351
N 2.167212 -1.902447 0.547934
S 2.930734 -0.465503 0.937328
O 4.199387 -0.458331 0.205194
O 2.855505 -0.392931 2.398183
C 1.947843 0.897220 0.292265
C 0.740967 1.231958 0.913472
H 0.416180 0.700707 1.802750
C -0.034201 2.261079 0.387853
H -0.980821 2.507080 0.861769
C 0.383528 2.977840 -0.744772
C 1.603599 2.632483 -1.339441
H 1.947453 3.181164 -2.213002
C 2.388466 1.596696 -0.830886
H 3.336200 1.333252 -1.288026
C -0.455279 4.110146 -1.287872
H -0.326197 5.019935 -0.687011
H -0.180233 4.355817 -2.318354
H -1.521877 3.860282 -1.269518
C 0.920001 -1.925285 -1.680865
H 1.034785 -0.851773 -1.874701
H 0.992274 -2.426992 -2.656634
C -0.462033 -2.188287 -1.069147
H -0.563167 -3.258959 -0.831999
H -0.550079 -1.647558 -0.117739
C -1.615993 -1.764294 -1.985942
H -1.481935 -0.713045 -2.276130
H -1.573926 -2.350432 -2.914395
C -3.018132 -1.937339 -1.358085
H -3.769735 -1.778410 -2.141962
H -3.135194 -2.976277 -1.022673
C -3.296992 -0.994470 -0.204609
C -3.157432 -1.405681 1.128018
H -2.871701 -2.433614 1.340961
C -3.389454 -0.521580 2.184125
H -3.278184 -0.864540 3.209560
C -3.774197 0.794576 1.924135
H -3.965608 1.481421 2.744249
C -3.925750 1.217178 0.600935
H -4.241718 2.235420 0.387306
C -3.687146 0.330452 -0.449440
H -3.814600 0.666681 -1.476589

45
-1302.25799365620000 NH.conf08
H -0.890018 -1.439114 -0.551454
C -0.088841 -2.650734 1.019383
H -0.999876 -3.254601 1.102098
H 0.747814 -3.354109 1.031637
N -0.115659 -2.055569 -0.320672
S 1.257936 -1.703538 -1.199477
O 0.774188 -1.368943 -2.540627
O 2.201809 -2.795669 -0.946331
C 2.002238 -0.205518 -0.532725
C 3.137884 -0.296821 0.272031
H 3.576294 -1.268934 0.470891
C 3.700548 0.868731 0.792710
H 4.588195 0.798856 1.416933
C 3.147347 2.126330 0.519581
C 2.006832 2.189587 -0.295792
H 1.564758 3.156848 -0.522946
C 1.434049 1.037249 -0.827453
H 0.563120 1.101622 -1.472459
C 3.778894 3.387235 1.060199
H 4.437326 3.849401 0.312818
H 3.021822 4.132181 1.328199
H 4.385432 3.183501 1.948325
C 0.017012 -1.672592 2.205500
H -0.105034 -2.260925 3.126412
H 1.030019 -1.253678 2.237477
C -0.989972 -0.511712 2.180363
H -0.790024 0.148374 3.035321
H -0.798354 0.098176 1.288818
C -2.468656 -0.928029 2.205313
H -2.646502 -1.744886 1.493595
H -2.724128 -1.331636 3.194469
C -3.435469 0.227601 1.863439
H -4.466739 -0.130858 1.983772
H -3.303362 1.041582 2.587637
C -3.243244 0.767141 0.458430
C -2.782506 2.070771 0.236071
H -2.587712 2.718256 1.088168
C -2.568220 2.549739 -1.059088
H -2.214742 3.567316 -1.205651
C -2.806435 1.726510 -2.160051
H -2.636886 2.094091 -3.168228
C -3.272256 0.424764 -1.956001
H -3.467913 -0.222862 -2.806283
C -3.492013 -0.045414 -0.660416

H -3.871972 -1.055144 -0.516512

45
-1302.25756879910000 NH.conf73
H 1.158769 -3.162016 0.155846
C 0.096376 -1.705843 1.254035
H 0.265115 -2.099311 2.264240
H 0.183635 -0.616592 1.325938
N 1.216031 -2.180038 0.420858
S 1.633802 -1.306640 -0.964435
O 0.498415 -0.514536 -1.462342
O 2.304744 -2.285918 -1.826731
C 2.842728 -0.141227 -0.337173
C 4.072293 -0.612853 0.133503
H 4.282041 -1.677444 0.135833
C 5.016653 0.298539 0.590367
H 5.974446 -0.064075 0.956130
C 4.757428 1.680136 0.583845
C 3.521755 2.123507 0.100865
H 3.305130 3.188599 0.082047
C 2.561078 1.222352 -0.363186
H 1.607113 1.565954 -0.747754
C 5.795268 2.654092 1.088382
H 6.742807 2.540146 0.548143
H 5.463876 3.690085 0.970911
H 6.010063 2.489351 2.151612
C -1.296239 -2.093854 0.744775
H -1.384716 -1.751033 -0.290044
H -1.383062 -3.191126 0.727025
C -2.418356 -1.499933 1.606877
H -2.325375 -1.878111 2.634940
H -2.288522 -0.410999 1.666639
C -3.834657 -1.808308 1.095260
H -3.970704 -2.897294 1.030914
H -4.561000 -1.450047 1.836826
C -4.188033 -1.189818 -0.278531
H -3.512536 -1.583348 -1.046931
H -5.194425 -1.532635 -0.552501
C -4.151937 0.325487 -0.301497
C -3.062734 1.021578 -0.844867
H -2.228927 0.470572 -1.274538
C -3.033684 2.418210 -0.843657
H -2.181346 2.937389 -1.274733
C -4.093814 3.144063 -0.299725
H -4.073343 4.230791 -0.302988
C -5.185573 2.463046 0.243501
H -6.020601 3.018229 0.663666
C -5.211286 1.068624 0.240367
H -6.070355 0.546244 0.657471

45
-1302.25719621380000 NH.conf03
H -0.655947 0.613191 0.367025
C -0.249764 -1.077666 1.582306
H 0.348568 -1.966795 1.359922
H 0.053159 -0.717594 2.573462
N 0.094214 -0.036280 0.597511
S 0.964920 -0.379252 -0.791281
O 0.545692 0.646496 -1.757611
O 0.881957 -1.809759 -1.117590
C 2.666602 -0.055869 -0.323191
C 3.580628 -1.105572 -0.284279
H 3.253635 -2.110334 -0.528478
C 4.907376 -0.840213 0.062064
H 5.622843 -1.658167 0.093096
C 5.331811 0.457868 0.364271
C 4.388931 1.498816 0.309872
H 4.701279 2.515671 0.536296
C 3.064557 1.252715 -0.032284
H 2.344953 2.063677 -0.078871
C 6.767219 0.745641 0.735102
H 7.231273 1.435662 0.019571
H 6.836385 1.214713 1.724220
H 7.367322 -0.168974 0.754840
C -1.745885 -1.421011 1.588746
H -1.963179 -1.975639 2.512398
H -2.315748 -0.486068 1.663724
C -2.222847 -2.244424 0.379445
H -1.743009 -1.880056 -0.536237
H -1.862111 -3.273656 0.502539
C -3.755529 -2.251463 0.192238
H -4.071535 -3.226929 -0.198833
H -4.250594 -2.142530 1.167612
C -4.294958 -1.176558 -0.783528
H -3.886300 -1.381537 -1.781019
H -5.383366 -1.300757 -0.861973
C -3.980230 0.256950 -0.406661
C -4.713295 0.916007 0.591547
H -5.537662 0.397931 1.077956
C -4.403175 2.223380 0.969005
H -4.987321 2.714531 1.743249
C -3.349592 2.901348 0.351242
H -3.108742 3.920244 0.642744
C -2.616911 2.263378 -0.652011
H -1.799430 2.777111 -1.151089
C -2.934694 0.954862 -1.028720
H -2.355352 0.475268 -1.813610

45
-1302.25690461000000 NH.conf48
H 0.199710 -2.511676 1.196475
C 0.568993 -2.671504 -0.866113
H 1.212400 -2.120925 -1.560754
H 0.962592 -3.694622 -0.806330
N 0.643085 -1.991814 0.440224
S 2.157715 -1.498345 1.011478
O 3.233202 -2.340670 0.470858
O 1.969959 -1.363191 2.460866
C 2.324591 0.123081 0.267111
C 1.348789 1.098542 0.497749
H 0.465878 0.863738 1.083135
C 1.517865 2.365602 -0.048604
H 0.756183 3.122853 0.121141
C 2.651820 2.682291 -0.816094
C 3.613195 1.687667 -1.026621
H 4.495995 1.913060 -1.619652
C 3.458999 0.408202 -0.489401
H 4.202597 -0.364330 -0.652608
C 2.823994 4.068984 -1.388670
H 2.983001 4.807740 -0.592885
H 3.681927 4.120125 -2.065769
H 1.933630 4.383829 -1.945593
C -0.877160 -2.664273 -1.376015
H -0.947917 -3.362090 -2.221527
H -1.528782 -3.075901 -0.591926
C -1.360617 -1.272211 -1.809510
H -1.086780 -0.538038 -1.043208
H -0.813322 -0.986261 -2.718313
C -2.869762 -1.183140 -2.088021
H -3.074110 -0.261447 -2.648335
H -3.174602 -2.013182 -2.741327
C -3.766418 -1.192052 -0.826688
H -4.813304 -1.247346 -1.152827
H -3.580887 -2.103138 -0.244292
C -3.583180 0.022008 0.062502
C -2.844817 -0.047307 1.251425
H -2.409259 -0.996467 1.554548
C -2.660522 1.081264 2.054243
H -2.089521 0.999655 2.975712
C -3.216639 2.305025 1.678847
H -3.080319 3.183473 2.304036
C -3.959086 2.389222 0.498633
H -4.404453 3.335402 0.201580
C -4.139213 1.258500 -0.297908
H -4.725972 1.333247 -1.211492

45
-1302.25649033490000 NH.conf61
H -0.654412 -0.611433 0.367380
C -0.250540 1.081372 1.580955

H 0.052843 0.722335 2.572351
H 0.346729 1.971003 1.357803
N 0.094498 0.039818 0.596797
S 0.965190 0.382242 -0.792046
O 0.883612 1.813120 -1.116938
O 0.544585 -0.642503 -1.758820
C 2.666813 0.056477 -0.325237
C 3.065744 -1.254164 -0.045932
H 2.347541 -2.065696 -0.102973
C 4.390292 -1.502145 0.294883
H 4.703971 -2.521017 0.509979
C 5.331730 -0.460807 0.360776
C 4.906741 0.839410 0.067811
H 5.622030 1.657332 0.104073
C 3.580139 1.106611 -0.276984
H 3.253060 2.112768 -0.515267
C 6.763498 -0.747146 0.746438
H 7.179454 -1.570053 0.153505
H 7.401114 0.129990 0.600631
H 6.838179 -1.041167 1.801279
C -1.747047 1.423049 1.587228
H -2.315890 0.487572 1.663163
H -1.964802 1.978334 2.510377
C -2.224953 2.244788 0.377159
H -1.865561 3.274596 0.499362
H -1.744562 1.880202 -0.538163
C -3.757606 2.249745 0.189705
H -4.252681 2.141575 1.165155
H -4.074683 3.224286 -0.202790
C -4.295596 1.172864 -0.784686
H -5.384094 1.295897 -0.863771
H -3.886718 1.376813 -1.782297
C -3.979657 -0.259798 -0.405660
C -4.712031 -0.917910 0.593665
H -5.536688 -0.399739 1.079483
C -4.400869 -2.224493 0.973012
H -4.984504 -2.714912 1.748104
C -3.346920 -2.902601 0.356047
H -3.105266 -3.920890 0.648994
C -2.614903 -2.265560 -0.648286
H -1.797185 -2.779489 -1.146780
C -2.933699 -0.957843 -1.026876
H -2.354790 -0.478931 -1.812496

45
-1302.25613239770000 NH.conf94
H 1.275409 -2.861438 -0.897470
C 0.683614 -2.167048 0.993472
H 1.124300 -2.958955 1.613140
H 0.823931 -1.223153 1.530508
N 1.380454 -2.040985 -0.299884
S 3.036306 -1.679294 -0.263209
O 3.528500 -2.078082 -1.586440
O 3.664035 -2.194258 0.961326
C 3.025705 0.108785 -0.145026
C 3.495031 0.722882 1.014050
H 3.850293 0.112956 1.837598
C 3.505740 2.117129 1.087184
H 3.870376 2.600101 1.990287
C 3.059740 2.901968 0.017875
C 2.595775 2.254626 -1.140103
H 2.248065 2.849035 -1.981739
C 2.578336 0.867396 -1.231154
H 2.227748 0.373233 -2.131264
C 3.092824 4.409958 0.089936
H 3.893854 4.816647 -0.540761
H 2.152189 4.847940 -0.262991
H 3.267480 4.759312 1.112034
C -0.808904 -2.438991 0.771809
H -0.918574 -3.411005 0.268953
H -1.269446 -2.560292 1.761897
C -1.572150 -1.370159 -0.027125
H -1.129405 -1.278704 -1.027547
H -2.600745 -1.723305 -0.171865
C -1.602786 0.012640 0.635923
H -1.974425 -0.081417 1.666681
H -0.583769 0.414106 0.705548
C -2.469630 1.045539 -0.119556
H -2.315126 2.029176 0.343448
H -2.105071 1.129032 -1.151711
C -3.951532 0.727578 -0.129842
C -4.729606 0.926324 1.020609
H -4.262828 1.330228 1.917016
C -6.090603 0.622592 1.030517
H -6.674668 0.788748 1.932224
C -6.703774 0.112929 -0.116487
H -7.764914 -0.121530 -0.111635
C -5.944361 -0.087037 -1.269449
H -6.411677 -0.478649 -2.169434
C -4.581613 0.219027 -1.273137
H -3.998199 0.065431 -2.178453

45
-1302.25571225320000 NH.conf84
H -1.603125 2.662709 0.145220
C -0.266275 2.293897 -1.444845
H 0.143630 1.365873 -1.848376
H -0.763528 2.813733 -2.273208
N -1.321389 1.915280 -0.485258
S -1.253440 0.461455 0.368178
O -1.066599 0.750185 1.797347
O -0.329411 -0.418022 -0.360549
C -2.910365 -0.197290 0.179846
C -3.806306 -0.119890 1.243944
H -3.493043 0.327991 2.180789
C -5.093199 -0.637069 1.086724
H -5.794593 -0.578752 1.915282
C -5.493302 -1.234252 -0.114130
C -4.565817 -1.304198 -1.166917
H -4.856479 -1.769810 -2.105695
C -3.279763 -0.793729 -1.029037
H -2.564778 -0.863874 -1.842232
C -6.878917 -1.812862 -0.274393
H -6.851123 -2.910160 -0.268766
H -7.542604 -1.495060 0.535415
H -7.332073 -1.507510 -1.224439
C 0.838435 3.195956 -0.870669
H 1.468905 3.514951 -1.713299
H 0.369277 4.113326 -0.484433
C 1.716935 2.574528 0.227002
H 2.343694 3.368779 0.656190
H 1.080521 2.218037 1.047963
C 2.615756 1.428081 -0.255405
H 2.007709 0.651342 -0.731322
H 3.313893 1.797444 -1.020362
C 3.412337 0.778805 0.893569
H 4.065096 1.529545 1.358587
H 2.701858 0.458793 1.667588
C 4.237139 -0.408582 0.443272
C 3.613170 -1.627521 0.136121
H 2.533510 -1.713488 0.241339
C 4.358290 -2.721511 -0.302798
H 3.856528 -3.658311 -0.532454
C 5.744601 -2.617790 -0.442918
H 6.326064 -3.471308 -0.781983
C 6.377134 -1.411561 -0.140982
H 7.455744 -1.320305 -0.243533
C 5.626834 -0.318312 0.297941
H 6.127619 0.618299 0.535345

45
-1302.25520656690000 NH.conf28
H 0.700870 -0.065961 0.429746
C 0.277436 -1.788844 1.598183
H 0.553106 -1.299670 2.540883
H -0.640942 -2.351216 1.787439
N -0.059584 -0.698411 0.668517
S -1.017060 -0.978044 -0.681173
O -1.292616 -2.416678 -0.777195
O -0.391500 -0.229185 -1.781721

C -2.574408 -0.170523 -0.302189
C -3.647897 -0.929526 0.161184
H -3.542375 -2.003746 0.267719
C -4.850898 -0.291368 0.464107
H -5.690019 -0.881284 0.824679
C -4.998747 1.092013 0.305471
C -3.902875 1.829759 -0.169386
H -3.999975 2.904389 -0.304906
C -2.695399 1.210202 -0.476524
H -1.858210 1.784535 -0.859285
C -6.311867 1.774263 0.607155
H -6.875354 1.969616 -0.314707
H -6.944512 1.158627 1.254093
H -6.157295 2.739736 1.101355
C 1.399697 -2.746588 1.163843
H 1.536387 -3.456232 1.991132
H 1.057659 -3.333270 0.303492
C 2.741354 -2.051617 0.841364
H 2.818462 -1.119393 1.419825
H 3.573910 -2.678129 1.187560
C 2.947708 -1.753246 -0.654572
H 3.150009 -2.695748 -1.180742
H 2.026081 -1.360462 -1.098028
C 4.096002 -0.763126 -0.938692
H 5.029657 -1.146548 -0.506403
H 4.254054 -0.717818 -2.024770
C 3.834914 0.635804 -0.411311
C 2.752777 1.391895 -0.894430
H 2.102026 0.976893 -1.660881
C 2.501154 2.674734 -0.404840
H 1.662109 3.243904 -0.797332
C 3.326677 3.230377 0.576007
H 3.131684 4.230249 0.954595
C 4.405154 2.491504 1.061909
H 5.056239 2.912664 1.823877
C 4.652936 1.206764 0.571808
H 5.497099 0.638495 0.956610

45
-1302.25462115780000 NH.conf59
H 2.748335 1.577005 1.738397
C 2.955045 2.123577 -0.277586
H 2.983441 3.128455 0.160146
H 3.977692 1.867032 -0.579025
N 2.502894 1.230311 0.813407
S 3.002960 -0.397650 0.800829
O 3.181014 -0.733113 2.219352
O 4.085259 -0.580135 -0.174947
C 1.573003 -1.286016 0.190668
C 1.610604 -1.869534 -1.073442
H 2.511108 -1.795694 -1.673659
C 0.482080 -2.546298 -1.541127
H 0.506316 -3.003124 -2.527372
C -0.678210 -2.644303 -0.764420
C -0.681532 -2.056993 0.512119
H -1.576348 -2.115797 1.126307
C 0.435344 -1.386442 0.996510
H 0.427143 -0.940949 1.985762
C -1.903660 -3.365192 -1.272118
H -2.793617 -2.731262 -1.183980
H -1.793156 -3.656539 -2.321089
H -2.096339 -4.276034 -0.691167
C 2.050110 2.138676 -1.516084
H 2.593608 2.717050 -2.275816
H 1.964097 1.122042 -1.921191
C 0.648979 2.754753 -1.339263
H 0.733939 3.690505 -0.765535
H 0.277495 3.047228 -2.331993
C -0.391864 1.837211 -0.683599
H -0.458297 0.900523 -1.253431
H -0.063181 1.556235 0.321811
C -1.794519 2.473448 -0.605512
H -1.740434 3.391863 -0.004829
H -2.110802 2.778936 -1.612043
C -2.824837 1.534816 -0.013346
C -3.637293 0.743884 -0.836981
H -3.552989 0.840089 -1.917553
C -4.555530 -0.156755 -0.293114
H -5.184902 -0.750384 -0.951812
C -4.675679 -0.284304 1.092331
H -5.394151 -0.979478 1.518595
C -3.874766 0.499760 1.925852
H -3.967472 0.416622 3.005858
C -2.961166 1.399832 1.375528
H -2.345918 2.011162 2.032770

45
-1302.25357580010000 NH.conf63
H 0.718730 0.379390 0.285727
C 0.461362 -1.224635 1.672423
H 0.200585 -0.744568 2.625021
H -0.140852 -2.133848 1.589378
N 0.051398 -0.328147 0.583201
S -0.917140 -0.820793 -0.681045
O -1.015910 -2.285663 -0.661205
O -0.428552 -0.091806 -1.860826
C -2.553581 -0.185849 -0.304184
C -2.865344 1.140365 -0.612780
H -2.130043 1.769919 -1.102882
C -4.129710 1.631358 -0.301051
H -4.374954 2.663082 -0.542095
C -5.095033 0.817606 0.312131
C -4.757264 -0.509664 0.604540
H -5.493369 -1.158167 1.073395
C -3.495215 -1.018893 0.298459
H -3.244186 -2.052889 0.509003
C -6.472560 1.355733 0.618328
H -6.427061 2.385762 0.988724
H -7.101801 1.363283 -0.281436
H -6.983349 0.746286 1.370360
C 1.954747 -1.586334 1.690925
H 2.551303 -0.668007 1.742260
H 2.120436 -2.099740 2.648814
C 2.464351 -2.510591 0.565034
H 3.387758 -2.989272 0.922380
H 1.736681 -3.322275 0.430924
C 2.762971 -1.909507 -0.823267
H 2.989647 -2.753203 -1.488413
H 1.873377 -1.436502 -1.249220
C 3.961210 -0.934062 -0.906012
H 4.819043 -1.368814 -0.375557
H 4.260653 -0.870438 -1.961631
C 3.724326 0.477778 -0.401008
C 2.728855 1.287799 -0.974423
H 2.111699 0.897115 -1.780030
C 2.517559 2.592193 -0.522232
H 1.743706 3.200721 -0.983580
C 3.301707 3.118237 0.508094
H 3.138221 4.134040 0.857815
C 4.300801 2.329018 1.077439
H 4.922207 2.727146 1.875681
C 4.507691 1.023395 0.624406
H 5.290480 0.416761 1.074978

45
-1302.25341800910000 NH.conf65
H 0.718012 0.378792 0.286707
C 0.462770 -1.226919 1.671885
H 0.202177 -0.747697 2.624964
H -0.138935 -2.136428 1.588454
N 0.051641 -0.329919 0.583529
S -0.917163 -0.822030 -0.680684
O -1.016637 -2.286842 -0.660407
O -0.428335 -0.093523 -1.860667
C -2.553359 -0.186208 -0.304160
C -2.866303 1.138184 -0.618095
H -2.132190 1.765956 -1.112233
C -4.130574 1.630053 -0.306042
H -4.376726 2.660587 -0.551036
C -5.094159 0.818772 0.312397

C -4.755011 -0.507208 0.610518
H -5.489776 -1.153854 1.084109
C -3.493581 -1.017080 0.304309
H -3.241566 -2.049941 0.519239
C -6.472612 1.355359 0.617096
H -7.123462 1.292916 -0.265108
H -6.955328 0.788814 1.419735
H -6.435674 2.407950 0.917702
C 1.956385 -1.587692 1.689389
H 2.552401 -0.669047 1.741303
H 2.122787 -2.101868 2.646741
C 2.466067 -2.510583 0.562411
H 3.389906 -2.989059 0.918907
H 1.738820 -3.322570 0.427817
C 2.763754 -1.907945 -0.825416
H 2.990787 -2.750831 -1.491472
H 1.873683 -1.435126 -1.250578
C 3.961287 -0.931584 -0.907628
H 4.819595 -1.366243 -0.377865
H 4.260337 -0.866732 -1.963283
C 3.723601 0.479609 -0.401168
C 2.727366 1.289511 -0.973438
H 2.110215 0.899194 -1.779224
C 2.515338 2.593314 -0.519891
H 1.740925 3.201783 -0.980380
C 3.299482 3.118886 0.510686
H 3.135403 4.134226 0.861472
C 4.299325 2.329800 1.078889
H 4.920742 2.727564 1.877304
C 4.506959 1.024766 0.624488
H 5.290341 0.418240 1.074177

TS-1,2'-HAT$_{bi}$ pathway

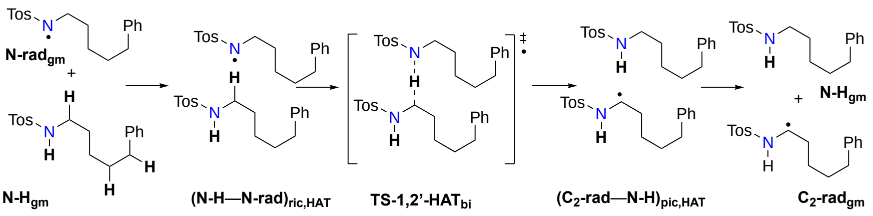

Name	E(B3LYP)	G(B3LYP)	E(RO-B2PLYP-D3)	G(RO-B2PLYP-D3)
**N-H$_{gm}$**				
NH.conf15	-1303.10330570	-1302.778657	-1302.5831192072	-1302.2584705072
**N-rad$_{gm}$**				
N-rad_0007	-1302.44553888	-1302.137918	-1301.9129157256	-1301.6052948456
**C$_2$-rad$_{gm}$**				
TScrad15crad.C2rad.conf18	-1302.44649895	-1302.138121	-1301.9250302632	-1301.6166523132
**N-H$_{gm}$ + N-rad$_{gm}$**				
Summed	-2605.54884458	-2604.916575	-2604.4960349328	-2603.8637653528
**(N-H—N-rad)$_{ric,HAT}$**				
fullTos_Nrad_PhC2.bimol.conf24_orc_fwd	-2605.55695983	-2604.908179	-2604.50652408350000	-2603.85774325350000
fullTos_Nrad_PhC2.bimol.conf11_orc_fwd	-2605.55475717	-2604.904873	-2604.50643188330000	-2603.85654771330000
fullTos_Nrad_PhC2.bimol.conf26_orc_rev	-2605.55360791	-2604.902026	-2604.50730662510000	-2603.85572471510000
**TS-1,2'-HAT$_{bi}$**				
fullTos_Nrad_PhC2.bimol.conf26	-2605.53098795	-2604.877601	-2604.5014046881	-2603.8480177381
fullTos_Nrad_PhC2.bimol.conf19	-2605.53103932	-2604.878829	-2604.4979082519	-2603.8456979319
**(C2-rad—N-H)$_{pic,HAT}$**				
fullTos_Nrad_PhC2.bimol.conf19_orc_rev	-2605.55384724	-2604.898949	-2604.5235527056	-2603.8686544656
fullTos_Nrad_PhC2.bimol.conf26_orc_fwd	-2605.55484440	-2604.895423	-2604.5264417824	-2603.8670203824
**N-H$_{gm}$ + C2-rad$_{gm}$**				
Summed	-2605.54980465	-2604.916778	-2604.5081494704	-2603.8751228204

45
-1302.25847050720000 NH.conf15
H 1.370625 -2.076475 1.152152
C 2.092476 -2.425601 -0.820792
H 3.049714 -2.192621 -1.295165
H 2.044751 -3.518341 -0.728351
N 2.167212 -1.902447 0.547934
S 2.930734 -0.465503 0.937328
O 4.199387 -0.458331 0.205194
O 2.855505 -0.392931 2.398183
C 1.947843 0.897220 0.292265
C 0.740967 1.231958 0.913472
H 0.416180 0.700707 1.802750
C -0.034201 2.261079 0.387853
H -0.980821 2.507080 0.861769
C 0.383528 2.977840 -0.744772
C 1.603599 2.632483 -1.339441
H 1.947453 3.181164 -2.213002
C 2.388466 1.596696 -0.830886
H 3.336200 1.333252 -1.288026
C -0.455279 4.110146 -1.287872
H -0.326197 5.019935 -0.687011
H -0.180233 4.355817 -2.318354
H -1.521877 3.860282 -1.269518
C 0.920001 -1.925285 -1.680865
H 1.034785 -0.851773 -1.874701
H 0.992274 -2.426992 -2.656634
C -0.462033 -2.188287 -1.069147
H -0.563167 -3.258959 -0.831999
H -0.550079 -1.647558 -0.117739
C -1.615993 -1.764294 -1.985942
H -1.481935 -0.713045 -2.276130
H -1.573926 -2.350432 -2.914395
C -3.018132 -1.937339 -1.358085
H -3.769735 -1.778410 -2.141962
H -3.135194 -2.976277 -1.022673
C -3.296992 -0.994470 -0.204609
C -3.157432 -1.405681 1.128018
H -2.871701 -2.433614 1.340961
C -3.389454 -0.521580 2.184125
H -3.278184 -0.864540 3.209560
C -3.774197 0.794576 1.924135
H -3.965608 1.481421 2.744249
C -3.925750 1.217178 0.600935
H -4.241718 2.235420 0.387306
C -3.687146 0.330452 -0.449440
H -3.814600 0.666681 -1.476589

44
-1301.65239562 N-rad,gm
C -0.238134 -3.358884 -0.417824
N -0.609805 -1.951523 -0.427561
S -1.998149 -1.648411 0.514591
O -3.116568 -2.419032 -0.049923

O -1.610544 -1.810507 1.924776
C -2.288903 0.084461 0.198270
C -1.674282 1.042582 1.005501
C -3.142903 0.455088 -0.841819
C -1.915949 2.391813 0.753519
H -1.027868 0.734372 1.820167
C -3.370678 1.808502 -1.078048
H -3.625058 -0.306996 -1.444639
C -2.764524 2.795810 -0.287270
H -4.033993 2.103661 -1.887281
C 1.173473 -3.585353 -0.970638
H 1.253130 -4.650754 -1.223993
H 1.275072 -3.035891 -1.914106
C 2.316188 -3.234365 -0.000627
H 3.257217 -3.592444 -0.442056
H 2.176940 -3.813917 0.923155
C 2.475679 -1.750723 0.370010
C 2.848198 -0.839306 -0.817181
H 2.052581 -0.880181 -1.569952
H -1.441914 3.142714 1.380595
C -3.046908 4.258777 -0.531825
H -3.961317 4.573371 -0.011710
H -2.230884 4.891502 -0.168779
H -3.192030 4.466214 -1.597146
H 3.258369 -1.667856 1.136055
C 3.067829 0.600089 -0.402369
C 1.993901 1.499617 -0.349664
C 4.338101 1.060256 -0.030292
C 2.183324 2.818769 0.063777
H 1.001561 1.160040 -0.637279
C 4.532637 2.378875 0.383906
H 5.185156 0.378103 -0.070592
C 3.454259 3.263996 0.432560
H 1.336560 3.500339 0.092063
H 5.527566 2.715985 0.663918
H 3.604257 4.292370 0.750875
H -0.976728 -3.882605 -1.048841
H 1.555928 -1.374761 0.835108
H 3.758117 -1.229318 -1.294077
H -0.344582 -3.792423 0.589237

44
-1301.61665231320000 C2-rad,gm
S 1.523155 -1.824933 0.956840
N 0.048561 -2.040482 0.127851
C 2.208544 -0.266812 0.396304
C 1.876305 0.911870 1.069454
H 1.249864 0.871247 1.954439
C 2.370505 2.124698 0.596642
H 2.119377 3.043068 1.121952
C 3.189646 2.182308 -0.541170
C 3.745489 3.500566 -1.024661
C 3.502497 0.984982 -1.199034
H 4.138654 1.009697 -2.080519
C 3.020022 -0.239511 -0.739788
H 3.283510 -1.165702 -1.238640
O 1.136345 -1.672318 2.360621
O 2.402732 -2.895350 0.487717
C 0.002226 -2.300203 -1.245393
C -1.206182 -1.856550 -2.005235
H 4.668821 3.758187 -0.488997
H 3.036895 4.319264 -0.859978
H 3.986256 3.467493 -2.092020
H -1.206642 -2.341617 -2.988216
C -1.296649 -0.319661 -2.183669
H -2.117216 -2.216155 -1.496241
H -4.031632 -1.054173 -1.390509
H 0.689980 -3.057810 -1.598783
C -2.690257 0.205847 -2.561230
C -3.781467 0.009535 -1.478401
H -0.572156 -0.014431 -2.948616
H -0.977300 0.173654 -1.257742
H -3.035077 -0.269236 -3.490287
H -2.603593 1.278266 -2.780711
H -0.675162 -1.431849 0.508677
H -4.696768 0.507578 -1.824824
C -3.393435 0.542706 -0.113210
C -3.111228 -0.327854 0.950231
H -3.227302 -1.400596 0.806287
C -2.704631 0.158098 2.196608
H -2.488372 -0.537266 3.003208
C -2.581959 1.533022 2.402550
H -2.270065 1.915353 3.370602
C -2.874868 2.412989 1.358000
H -2.794353 3.486076 1.512251
C -3.274649 1.921406 0.114926
H -3.500190 2.617970 -0.689966

89
-2603.86865446560000
fullTos_Nrad_PhC2.bimol.conf19_orc_rev
S -0.525643 0.366101 -1.152912
N -0.559609 -0.388371 0.349465
C -1.789665 1.636922 -1.038701
C -2.783067 1.698044 -2.013291
H -2.815516 0.944408 -2.792367
C -3.722467 2.730533 -1.965033
H -4.497126 2.781030 -2.726307
C -3.682302 3.702049 -0.957772
C -4.683471 4.832078 -0.922634
C -2.669788 3.614691 0.012746
H -2.622604 4.358964 0.804234
C -1.722279 2.597091 -0.024722
H -0.939895 2.542914 0.725306
O -0.952573 -0.554868 -2.218154
O 0.784526 1.030368 -1.215505
C -1.352821 -1.624411 0.532182
H -0.807248 -2.207771 1.282707
C -2.785535 -1.373467 1.014374
H -4.231871 5.768303 -1.275762
H -5.047128 5.013721 0.094858
H -5.548084 4.622565 -1.559687
H -1.359182 -2.211261 -0.392965
H -3.272940 -2.354294 1.106944
H -3.344457 -0.835296 0.241137
S 4.013264 -2.179263 1.186284
N 3.457654 -0.665020 1.727962
C 3.007242 -2.603542 -0.233360
C 3.140607 -1.870430 -1.416845
H 3.866030 -1.066261 -1.491677
C 2.323301 -2.180908 -2.497592
H 2.409083 -1.600488 -3.412079
C 1.376166 -3.214970 -2.422925
C 0.456466 -3.489924 -3.585753
C 1.282773 -3.947923 -1.233125
H 0.567437 -4.763699 -1.162233
C 2.085878 -3.645755 -0.132527
H 2.014258 -4.212280 0.789620
O 5.380536 -1.915900 0.735727
O 3.705194 -3.132924 2.252586
C 2.185723 -0.503865 2.296109
H 1.845502 -1.389317 2.827130
C 1.778150 0.843042 2.835150
H -0.315744 -2.712656 -3.636092
H -0.040924 -4.459946 -3.486949
H 0.998859 -3.479149 -4.537878
H 3.830543 0.092091 1.157167
H 0.681250 0.849735 2.877852
H 2.112483 0.941717 3.884714
H 0.407548 -0.465397 0.681267
C 2.279442 2.066008 2.037040
H 2.259153 1.836346 0.964008
H 1.571009 2.894044 2.172056
C 3.677059 2.551280 2.467739
H 3.591232 3.077507 3.428032
H 4.334447 1.693999 2.660987
C 4.357522 3.480320 1.440594
H 3.699887 4.332486 1.225355
H 5.266242 3.896871 1.895665
C -2.859485 -0.627466 2.353450

H -2.156732 -1.101177 3.055654
H -2.497330 0.399302 2.213249
C -4.239776 -0.590564 3.031780
H -4.583728 -1.616866 3.220552
H -4.108859 -0.131208 4.020717
C -5.350578 0.194630 2.293970
H -6.110536 0.473245 3.037696
H -4.934786 1.139003 1.918945
C -6.048191 -0.534936 1.159700
C -6.038668 -0.028145 -0.146120
H -5.501233 0.893895 -0.355485
C -6.702900 -0.689617 -1.181593
H -6.677741 -0.278044 -2.187573
C -7.395348 -1.873454 -0.927547
H -7.913171 -2.390060 -1.731283
C -7.420661 -2.387075 0.371148
H -7.963087 -3.305046 0.583260
C -6.755867 -1.722312 1.401260
H -6.793222 -2.128463 2.409978
C 4.718121 2.778751 0.144311
C 4.082222 3.100431 -1.061772
H 3.320379 3.876223 -1.072789
C 4.405996 2.435695 -2.246144
H 3.895118 2.700333 -3.167928
C 5.373872 1.430139 -2.244061
H 5.629656 0.913785 -3.165668
C 6.014083 1.092145 -1.048510
H 6.760718 0.303728 -1.028363
C 5.689505 1.764110 0.131580
H 6.205041 1.498228 1.052179

89
-2603.86702038240000
fullTos_Nrad_PhC2.bimol.conf26_orc_fwd
S 1.237421 1.690192 1.144363
N 1.392070 0.401275 0.068695
C 2.542404 2.823265 0.659343
C 3.588693 3.086313 1.539990
H 3.615684 2.589228 2.503529
C 4.581887 3.993828 1.165487
H 5.399654 4.200647 1.851278
C 4.540578 4.643637 -0.072821
C 5.611912 5.628721 -0.475777
C 3.468515 4.365275 -0.937827
H 3.415722 4.865600 -1.902040
C 2.470545 3.465533 -0.580613
H 1.639306 3.265651 -1.248928
O 1.536470 1.268507 2.522614
O -0.053435 2.307162 0.808434
C 2.204743 -0.774321 0.453397
H 1.748959 -1.624714 -0.060476
C 3.684042 -0.662919 0.070004
H 5.194208 6.633476 -0.615899
H 6.080804 5.340911 -1.424524
H 6.399040 5.697000 0.280998
H 2.115266 -0.965173 1.528690
H 4.159756 -1.602237 0.378060
H 4.154282 0.125892 0.671783
S -2.817885 -2.304174 -0.339074
N -2.468786 -1.016083 -1.398514
C -2.114019 -1.857567 1.244893
C -2.712696 -0.843352 1.997836
H -3.622982 -0.362798 1.653020
C -2.123365 -0.455706 3.197040
H -2.577322 0.342881 3.777807
C -0.948141 -1.065023 3.662576
C -0.274981 -0.582020 4.922080
C -0.389242 -2.100036 2.900908
H 0.511077 -2.597517 3.252892
C -0.955456 -2.495897 1.689898
H -0.511376 -3.290360 1.100301
O -4.277019 -2.287975 -0.228986
O -2.081811 -3.461254 -0.852940
C -1.158626 -0.738398 -1.813930
H -0.552585 -1.632828 -1.932166
C -0.899698 0.429155 -2.728920
H 0.272271 -1.388507 5.421493
H -0.997322 -0.165575 5.631817
H 0.444715 0.207171 4.671455
H -3.091111 -0.227792 -1.228490
H 0.165330 0.676498 -2.634152
H -1.035962 0.119750 -3.782146
H 0.445280 0.151176 -0.234793
C -1.755031 1.688340 -2.467757
H -1.916882 1.803824 -1.388402
H -1.184582 2.575841 -2.769916
C -3.092382 1.689158 -3.232019
H -2.892971 1.884906 -4.294302
H -3.551798 0.692540 -3.200898
C -4.109879 2.725199 -2.711460
H -3.650643 3.722107 -2.714655
H -4.952271 2.769943 -3.414985
C 3.978376 -0.374309 -1.411162
H 3.597567 0.626127 -1.650768
H 5.069801 -0.321090 -1.533895
C 3.419612 -1.352344 -2.461231
H 2.322379 -1.356615 -2.426659
H 3.680687 -0.944953 -3.446979
C 3.939892 -2.810339 -2.400983
H 3.851233 -3.238392 -3.409127
H 5.013576 -2.802669 -2.171273
C 3.220358 -3.735361 -1.435711
C 1.857610 -4.024162 -1.610575
H 1.318185 -3.582218 -2.446049
C 1.179968 -4.880349 -0.740995
H 0.121980 -5.072949 -0.895301
C 1.863111 -5.475934 0.323385
H 1.341340 -6.147590 0.999974
C 3.221088 -5.209919 0.504006
H 3.763812 -5.674276 1.323615
C 3.890229 -4.348360 -0.368787
H 4.950244 -4.151092 -0.221939
C -4.633589 2.413906 -1.321704
C -4.355339 3.249685 -0.232303
H -3.753424 4.142321 -0.385336
C -4.837125 2.953076 1.044413
H -4.608190 3.617737 1.873237
C -5.605005 1.806856 1.255923
H -5.985080 1.576819 2.247899
C -5.885699 0.957885 0.181634
H -6.473941 0.057287 0.332267
C -5.406048 1.262985 -1.093560
H -5.646372 0.600870 -1.922943

89
-2603.85774325350000
fullTos_Nrad_PhC2.bimol.conf24_orc_fwd
S -0.603659 -0.894259 -0.707604
N -2.221166 -0.732497 -0.218338
C -0.513028 0.297140 -2.028989
C 0.351122 0.023754 -3.091298
H 0.914427 -0.901230 -3.116185
C 0.494254 0.974791 -4.098892
H 1.168108 0.768556 -4.926161
C -0.206270 2.187994 -4.062443
C -0.018542 3.220273 -5.147751
C -1.066912 2.431875 -2.981044
H -1.626383 3.363091 -2.941064
C -1.221816 1.500299 -1.957965
H -1.898305 1.690534 -1.132543
O -0.348387 -2.236988 -1.250607
O 0.198720 -0.440910 0.453344
C -2.661802 -1.823014 0.635416
H -1.934837 -1.975085 1.451480
C -4.063481 -1.552468 1.185677
H -0.900612 3.860268 -5.251317
H 0.180398 2.751072 -6.116686
H 0.834897 3.872929 -4.921339
H -2.640657 -2.751968 0.042170
H -4.400725 -2.460219 1.704495

H -4.748242 -1.399435 0.346360
S 3.958097 -1.187384 -0.730652
N 3.216489 -0.183645 0.374366
C 3.927974 -2.857136 -0.060338
C 2.763669 -3.625054 -0.168727
H 1.890071 -3.232115 -0.679562
C 2.746313 -4.905693 0.376285
H 1.843406 -5.505947 0.290038
C 3.870177 -5.438662 1.027511
C 3.839582 -6.840500 1.589089
C 5.022429 -4.648974 1.119206
H 5.905621 -5.045382 1.614500
C 5.060720 -3.362995 0.578439
H 5.961919 -2.761417 0.628733
O 3.087043 -1.173926 -1.912710
O 5.366793 -0.787200 -0.807700
C 3.812003 0.030515 1.706518
H 4.284056 -0.894071 2.069501
C 4.826003 1.181131 1.771854
H 3.922294 -7.588141 0.789330
H 2.901089 -7.039149 2.118948
H 4.665746 -7.011908 2.286208
H 2.201864 -0.302641 0.360141
H 5.328498 1.097092 2.746373
H 5.594276 1.015480 1.008421
H 2.971290 0.234073 2.379808
C 4.246412 2.601544 1.649990
H 3.424023 2.722837 2.369509
H 5.026375 3.310547 1.965937
C 3.769209 3.003090 0.247072
H 4.582243 2.830443 -0.471027
H 2.947028 2.354071 -0.071058
C 3.331084 4.480556 0.143929
H 4.166597 5.127811 0.442746
H 3.128632 4.705980 -0.912083
C -4.102769 -0.359699 2.151612
H -3.358434 -0.533447 2.942400
H -3.771191 0.543974 1.621563
C -5.459557 -0.088781 2.825518
H -5.845046 -1.019853 3.264385
H -5.283612 0.590881 3.669686
C -6.551984 0.557233 1.941555
H -7.338045 0.933226 2.611322
H -6.128905 1.441081 1.446444
C -7.197750 -0.338881 0.901650
C -7.074571 -0.068618 -0.467548
H -6.496346 0.794891 -0.789460
C -7.679043 -0.889369 -1.422600
H -7.567382 -0.659811 -2.479199
C -8.424976 -1.997891 -1.021570
H -8.897688 -2.637660 -1.761910
C -8.564360 -2.275307 0.340433
H -9.150339 -3.131761 0.664331
C -7.958317 -1.452454 1.289302
H -8.082327 -1.674115 2.347393
C 2.107949 4.830702 0.968707
C 2.205493 5.633294 2.112754
H 3.178276 6.020531 2.408300
C 1.077504 5.946412 2.874701
H 1.179510 6.572795 3.757504
C -0.175438 5.459181 2.502807
H -1.055738 5.702325 3.092222
C -0.288990 4.658250 1.363832
H -1.261497 4.275172 1.063683
C 0.840344 4.349243 0.606462
H 0.738230 3.726491 -0.279980

89
-2603.85654771330000
fullTos_Nrad_PhC2.bimol.conf11_orc_fwd
S -0.453622 1.086359 -0.938768
N -1.900418 1.071420 -0.046996
C -0.171410 2.832694 -1.140868
C -1.040016 3.578230 -1.944204
H -1.880953 3.098992 -2.434386
C -0.804756 4.938835 -2.104264
H -1.477304 5.526468 -2.724172
C 0.288810 5.565720 -1.483307
C 0.544344 7.039078 -1.689648
C 1.143928 4.789035 -0.692039
H 1.996809 5.254519 -0.205393
C 0.925224 3.422935 -0.514284
H 1.598956 2.824313 0.088844
O -0.786057 0.480960 -2.239803
O 0.655620 0.531994 -0.138220
C -2.170476 -0.209767 0.584773
H -1.347967 -0.446682 1.280311
C -3.520360 -0.186464 1.304936
H 0.985906 7.225269 -2.677305
H 1.235184 7.435633 -0.939652
H -0.384832 7.617159 -1.637690
H -2.143572 -1.004026 -0.180880
H -3.726413 -1.204649 1.662399
H -4.303227 0.055317 0.580301
S 2.743521 -2.906541 -0.863041
N 2.574320 -1.741658 0.319754
C 1.100307 -3.558377 -1.203722
C 0.209194 -2.806954 -1.975835
H 0.510413 -1.853728 -2.396597
C -1.073222 -3.299121 -2.205273
H -1.763008 -2.712654 -2.807623
C -1.482320 -4.537071 -1.683361
C -2.863159 -5.076590 -1.973000
C -0.568769 -5.270253 -0.915764
H -0.865143 -6.232967 -0.505827
C 0.719156 -4.790695 -0.672056
H 1.432052 -5.366857 -0.091913
O 3.189207 -2.185530 -2.058907
O 3.525228 -4.000272 -0.276048
C 2.168448 -2.115505 1.686834
H 1.462797 -2.959320 1.673380
C 3.341883 -2.428847 2.625483
H -2.896216 -5.564939 -2.955932
H -3.612363 -4.277458 -1.985373
H -3.168751 -5.819251 -1.228989
H 2.101942 -0.916509 -0.048196
H 2.904990 -2.616714 3.616304
H 3.814064 -3.365779 2.309398
H 1.616512 -1.249384 2.068906
C 4.398319 -1.306686 2.714079
H 3.918755 -0.336734 2.524307
H 4.792899 -1.253517 3.738531
C 5.573588 -1.496556 1.743112
H 6.154607 -2.375415 2.054926
H 5.187275 -1.731096 0.747597
C 6.523817 -0.287438 1.661152
H 6.920154 -0.062229 2.660714
H 7.391825 -0.574564 1.050091
C -3.545964 0.792412 2.487588
H -2.711862 0.540488 3.159108
H -3.340654 1.806982 2.119337
C -4.840258 0.801942 3.320426
H -5.108318 -0.227605 3.596605
H -4.626460 1.320218 4.264476
C -6.063778 1.502368 2.684459
H -6.800343 1.668191 3.483082
H -5.760629 2.500009 2.340967
C -6.748427 0.767092 1.547759
C -6.788279 1.304611 0.254528
H -6.309507 2.262309 0.061713
C -7.429608 0.630933 -0.787604
H -7.446114 1.067918 -1.782787
C -8.049125 -0.596459 -0.551302
H -8.550764 -1.122060 -1.359395
C -8.025185 -1.141208 0.734851
H -8.512213 -2.092886 0.932151
C -7.383142 -0.464153 1.771263
H -7.379864 -0.894511 2.770684
C 5.913918 0.975422 1.076947
C 6.022772 2.199590 1.750295

H 6.525134 2.231358 2.714997
C 5.508401 3.377014 1.203055
H 5.613526 4.314541 1.743982
C 4.866997 3.348244 -0.035591
H 4.478100 4.263767 -0.475076
C 4.739566 2.133044 -0.714465
H 4.240937 2.096728 -1.679764
C 5.257193 0.960137 -0.164338
H 5.140300 0.025554 -0.706399

89
-2603.85572471510000
fullTos_Nrad_PhC2.bimol.conf26_orc_rev
S 2.316780 1.659528 0.590628
N 3.744493 0.894703 0.075475
C 2.643635 3.362867 0.169029
C 3.231706 4.201695 1.116309
H 3.474186 3.820623 2.102495
C 3.489138 5.527724 0.775837
H 3.948281 6.186218 1.508893
C 3.163703 6.027871 -0.493026
C 3.417804 7.474760 -0.841683
C 2.569706 5.160826 -1.421922
H 2.308912 5.532440 -2.409739
C 2.304149 3.832056 -1.100744
H 1.833665 3.167350 -1.817240
O 2.230195 1.554464 2.057605
O 1.219513 1.169198 -0.261495
C 3.771749 -0.525596 0.384645
H 2.899196 -1.018368 -0.074354
C 5.083774 -1.160107 -0.083531
H 2.541507 8.093809 -0.607977
H 3.628054 7.598994 -1.908911
H 4.263284 7.880853 -0.277319
H 3.641055 -0.658323 1.471117
H 5.084328 -2.201595 0.254236
H 5.910663 -0.657283 0.434092
S -2.683803 -2.438210 0.584798
N -2.728721 -1.461734 -0.785610
C -1.999715 -1.442928 1.913071
C -2.825175 -0.548339 2.596757
H -3.886999 -0.509202 2.375765
C -2.267283 0.288340 3.562391
H -2.906796 0.989147 4.093576
C -0.897065 0.249947 3.852382
C -0.279688 1.181250 4.867097
C -0.094711 -0.671483 3.163964
H 0.970705 -0.708382 3.371695
C -0.633517 -1.515058 2.197194
H -0.007616 -2.225337 1.667784
O -4.089150 -2.716041 0.897407
O -1.726189 -3.514029 0.307832
C -1.476685 -0.842045 -1.261000
H -0.702831 -1.613205 -1.215554
C -1.602910 -0.330478 -2.703178
H 0.130780 0.626120 5.720071
H -1.011395 1.896753 5.255033
H 0.547068 1.742035 4.415967
H -3.501995 -0.804599 -0.683375
H -0.648428 0.158264 -2.935253
H -1.698426 -1.183618 -3.387055
H -1.160685 -0.019069 -0.605063
C -2.769823 0.654888 -2.934652
H -2.959476 1.217712 -2.009311
H -2.477572 1.407378 -3.678829
C -4.065427 -0.029678 -3.411053
H -3.958809 -0.292018 -4.472322
H -4.197758 -0.981104 -2.883345
C -5.334503 0.828883 -3.232002
H -5.204624 1.791412 -3.744043
H -6.170351 0.323069 -3.734667
C 5.331622 -1.077494 -1.599501
H 5.350728 -0.016698 -1.882084
H 6.340320 -1.464027 -1.802424
C 4.335625 -1.802370 -2.523947
H 3.315131 -1.436311 -2.349305
H 4.580099 -1.508268 -3.553334
C 4.345096 -3.348633 -2.468877
H 3.878311 -3.715139 -3.393736
H 5.385155 -3.700020 -2.496769
C 3.628339 -3.986694 -1.292656
C 2.241441 -3.837431 -1.134300
H 1.678996 -3.258368 -1.864645
C 1.562821 -4.438810 -0.072252
H 0.486889 -4.317997 0.020849
C 2.270315 -5.205777 0.857978
H 1.745774 -5.678473 1.683997
C 3.647935 -5.371551 0.711689
H 4.204437 -5.974841 1.424766
C 4.317309 -4.769592 -0.356644
H 5.390359 -4.912607 -0.468974
C -5.700841 1.076840 -1.779772
C -5.730267 2.371911 -1.246113
H -5.493806 3.217221 -1.888865
C -6.061357 2.593991 0.092772
H -6.079754 3.608949 0.481849
C -6.364610 1.518327 0.928062
H -6.623587 1.689318 1.969667
C -6.337387 0.219427 0.413282
H -6.567737 -0.630961 1.049079
C -6.012479 0.004242 -0.927438
H -6.006751 -1.013140 -1.312108

89
-2603.84801773810000 fullTos_Nrad_PhC2.bimol.conf26
S -0.555386 1.376042 0.966418
N 0.592784 1.154365 -0.246154
C -0.956687 3.120816 0.816454
C -0.541360 4.007797 1.808054
H 0.012990 3.634484 2.662343
C -0.859095 5.361519 1.687767
H -0.538278 6.054714 2.461563
C -1.586618 5.840994 0.592261
C -1.946967 7.302772 0.474041
C -1.994227 4.924272 -0.391119
H -2.563014 5.277778 -1.248122
C -1.687436 3.571817 -0.286858
H -2.015700 2.868808 -1.045213
O 0.003380 1.178989 2.315853
O -1.745977 0.609531 0.549918
C 1.991362 0.990599 0.185144
H 2.425280 0.222212 -0.462978
C 2.814998 2.283433 0.049020
H -3.013243 7.464586 0.679004
H -1.752613 7.682360 -0.535608
H -1.378585 7.914545 1.181240
H 2.057423 0.620084 1.213531
H 3.836632 2.038015 0.366157
H 2.439044 3.022944 0.768007
S -0.136614 -3.310842 -0.230661
N -0.806294 -1.975203 -1.035168
C 0.382236 -2.742444 1.382630
C -0.580538 -2.528920 2.373988
H -1.628316 -2.726512 2.173194
C -0.173671 -2.057781 3.616596
H -0.918748 -1.878189 4.387114
C 1.177541 -1.795535 3.888220
C 1.592455 -1.225488 5.221818
C 2.121430 -2.039903 2.882964
H 3.174903 -1.855915 3.078701
C 1.735286 -2.506549 1.627109
H 2.469817 -2.691025 0.850802
O -1.265265 -4.225124 -0.049232
O 1.057390 -3.677966 -0.996067
C -0.027369 -0.989672 -1.689008
H 0.967177 -1.390453 -1.900723
C -0.671912 -0.332648 -2.918666
H 2.656965 -1.388741 5.417560
H 1.020890 -1.668221 6.044820
H 1.411285 -0.143131 5.243456

H -1.700810 -1.681976 -0.642599
H -0.158684 0.625088 -3.066310
H -0.432061 -0.952378 -3.796101
H 0.207464 -0.006616 -0.913618
C -2.197362 -0.117849 -2.849538
H -2.488018 0.142020 -1.824354
H -2.460808 0.753284 -3.464457
C -3.001200 -1.332468 -3.350138
H -2.908484 -1.393380 -4.443349
H -2.558559 -2.255711 -2.958965
C -4.496675 -1.294950 -2.975331
H -4.944996 -0.362344 -3.342933
H -5.006086 -2.110756 -3.506696
C 2.826590 2.928299 -1.345838
H 1.811573 3.275660 -1.574126
H 3.454821 3.829552 -1.296316
C 3.303759 2.068151 -2.531163
H 2.623307 1.219156 -2.676542
H 3.201338 2.684348 -3.434210
C 4.763674 1.551356 -2.476748
H 5.097459 1.384130 -3.510292
H 5.413251 2.340281 -2.075353
C 4.987571 0.265919 -1.699914
C 4.367520 -0.927373 -2.104523
H 3.726790 -0.925668 -2.984176
C 4.563717 -2.119255 -1.405477
H 4.060071 -3.025405 -1.731122
C 5.401913 -2.142915 -0.286892
H 5.563897 -3.071008 0.254913
C 6.038161 -0.969141 0.119193
H 6.699798 -0.977530 0.981791
C 5.831008 0.221150 -0.582319
H 6.333997 1.130062 -0.258674
C -4.758066 -1.426516 -1.485733
C -5.387972 -0.404405 -0.764852
H -5.698296 0.499717 -1.284443
C -5.621675 -0.526278 0.606509
H -6.108384 0.283226 1.144297
C -5.225710 -1.678709 1.284755
H -5.405325 -1.774722 2.352295
C -4.595110 -2.707701 0.580794
H -4.281419 -3.613787 1.092519
C -4.366227 -2.582218 -0.790389
H -3.875466 -3.395418 -1.319524

89
-2603.84569793190000 fullTos_Nrad_PhC2.bimol.conf19
S -0.196842 0.186420 -0.640012
N -0.449638 -0.153475 0.988075
C -1.218790 1.639747 -0.910511
C -2.191980 1.610382 -1.907376
H -2.342365 0.702087 -2.480581
C -2.954114 2.755258 -2.149603
H -3.709376 2.736700 -2.931723
C -2.760107 3.926735 -1.407696
C -3.570215 5.170214 -1.686687
C -1.774065 3.926505 -0.406875
H -1.609496 4.826457 0.181122
C -1.000821 2.797722 -0.157267
H -0.238699 2.809963 0.614702
O -0.709104 -0.870989 -1.529146
O 1.212793 0.612793 -0.755850
C -1.299734 -1.315823 1.298494
H -0.918430 -1.713923 2.248192
C -2.782215 -0.940623 1.482873
H -2.976425 5.911586 -2.237208
H -3.901848 5.648874 -0.758436
H -4.456366 4.945718 -2.288182
H -1.205538 -2.104617 0.543378
H -3.317995 -1.869265 1.726795
H -3.191849 -0.598579 0.526982
S 3.585665 -2.284967 0.938899
N 2.983672 -0.738786 1.296470
C 2.465576 -3.027627 -0.240011
C 2.506067 -2.619558 -1.576447
H 3.220624 -1.870204 -1.899675
C 1.614394 -3.185420 -2.479937
H 1.633082 -2.863147 -3.517571
C 0.680893 -4.150626 -2.073783
C -0.316525 -4.710428 -3.057521
C 0.677348 -4.555385 -0.732778
H -0.025764 -5.317194 -0.405135
C 1.560410 -3.998027 0.191267
H 1.566765 -4.319567 1.227135
O 4.855875 -2.024409 0.259772
O 3.499543 -3.043440 2.188474
C 1.904278 -0.511534 2.184326
H 1.749750 -1.391723 2.813557
C 1.966982 0.788558 3.000436
H -1.136199 -3.997627 -3.214870
H -0.754355 -5.647684 -2.700004
H 0.143508 -4.898078 -4.033921
H 3.084626 -0.086155 0.518268
H 0.941993 1.006631 3.323101
H 2.543819 0.582695 3.914747
H 0.820855 -0.384634 1.528081
C 2.574579 2.007742 2.277803
H 2.298593 1.986734 1.216097
H 2.126129 2.923285 2.686650
C 4.104047 2.097973 2.432049
H 4.339549 2.384989 3.466258
H 4.547592 1.106597 2.285668
C 4.776919 3.097215 1.469459
H 4.329655 4.091760 1.599401
H 5.832516 3.195707 1.758472
C -3.017810 0.101368 2.584274
H -2.450636 -0.202945 3.476879
H -2.589204 1.061820 2.271327
C -4.480756 0.310337 3.012288
H -4.903715 -0.643890 3.356062
H -4.476169 0.970186 3.890268
C -5.424991 0.940952 1.961261
H -6.279242 1.374685 2.500276
H -4.913039 1.783950 1.479136
C -5.967571 0.005125 0.895996
C -5.728095 0.235869 -0.464772
H -5.120785 1.088435 -0.760240
C -6.250850 -0.612552 -1.443477
H -6.048459 -0.414334 -2.493147
C -7.029694 -1.710115 -1.076947
H -7.438207 -2.371570 -1.836319
C -7.283676 -1.948994 0.275628
H -7.895432 -2.796963 0.573362
C -6.759362 -1.098395 1.248527
H -6.974391 -1.290506 2.297689
C 4.691187 2.694001 0.008434
C 4.040531 3.503253 -0.931001
H 3.593471 4.440797 -0.606750
C 3.955701 3.126361 -2.273053
H 3.441984 3.770251 -2.982375
C 4.521163 1.925385 -2.700113
H 4.452891 1.628291 -3.743260
C 5.174249 1.106548 -1.775630
H 5.621426 0.167492 -2.090907
C 5.260223 1.489534 -0.436504
H 5.773850 0.837806 0.265949

TS-1,5’-HAT$_{bi}$ pathway

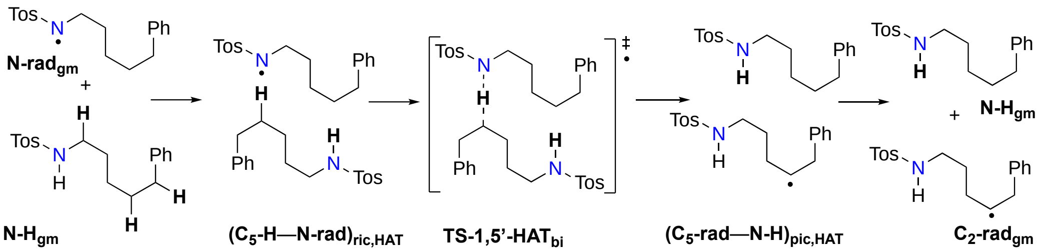

Name	E(B3LYP)	G(B3LYP)	E(RO-B2PLYP-D3)	G(RO-B2PLYP-D3)
**N-H$_{gm}$**				
NH.conf15	-1303.10330570	-1302.778657	-1302.5831192072	-1302.2584705072
**N-rad$_{gm}$**				
N-rad_0007	-1302.44553888	-1302.137918	-1301.9129157256	-1301.6052948456
**C$_5$-rad$_{gm}$**				
C5-rad_0023	-1302.43760047	-1302.128919	-1301.9165611576	-1301.6078796876
**N-H$_{gm}$ + N-rad$_{gm}$**				
Summed	-2605.54884458	-2604.916575	-2604.4960349328	-2603.8637653528
**(C5-H—N-rad)$_{ric,HAT}$**				
fullTos_Nrad_PhC5.bimol.conf44_orc_fwd	-2605.56128203	-2604.906741	-2604.5211794534	-2603.8666384234
fullTos_Nrad_PhC5.bimol.conf39_orc_fwd	-2605.56266321	-2604.906754	-2604.5211766009	-2603.8652673909
**TS-1,5’-HAT$_{bi}$**				
fullTos_Nrad_PhC5.bimol.conf76	-2605.53171820	-2604.876326	-2604.5002932431	-2603.8449010431
fullTos_Nrad_PhC5.bimol.conf38	-2605.53004634	-2604.877507	-2604.4955458706	-2603.8430065306
fullTos_Nrad_PhC5.bimol.conf39	-2605.53004647	-2604.877398	-2604.4955549867	-2603.8429065167
fullTos_Nrad_PhC5.bimol.conf44	-2605.52744318	-2604.874028	-2604.4938478135	-2603.8404326335
**(C5-rad—N-H)$_{pic,HAT}$**				
fullTos_Nrad_PhC5.bimol.conf44_orc_rev	-2605.55835809	-2604.905408	-2604.5263782815	-2603.8734281915
fullTos_Nrad_PhC5.bimol.conf38_orc_rev	-2605.55667600	-2604.907088	-2604.5226396613	-2603.8730516613

45
-1302.25847050720000 NH.conf15
H 1.370625 -2.076475 1.152152
C 2.092476 -2.425601 -0.820792
H 3.049714 -2.192621 -1.295165
H 2.044751 -3.518341 -0.728351
N 2.167212 -1.902447 0.547934
S 2.930734 -0.465503 0.937328
O 4.199387 -0.458331 0.205194
O 2.855505 -0.392931 2.398183
C 1.947843 0.897220 0.292265
C 0.740967 1.231958 0.913472
H 0.416180 0.700707 1.802750
C -0.034201 2.261079 0.387853
H -0.980821 2.507080 0.861769
C 0.383528 2.977840 -0.744772
C 1.603599 2.632483 -1.339441
H 1.947453 3.181164 -2.213002
C 2.388466 1.596696 -0.830886
H 3.336200 1.333252 -1.288026
C -0.455279 4.110146 -1.287872
H -0.326197 5.019935 -0.687011
H -0.180233 4.355817 -2.318354
H -1.521877 3.860282 -1.269518
C 0.920001 -1.925285 -1.680865
H 1.034785 -0.851773 -1.874701
H 0.992274 -2.426992 -2.656634
C -0.462033 -2.188287 -1.069147
H -0.563167 -3.258959 -0.831999
H -0.550079 -1.647558 -0.117739
C -1.615993 -1.764294 -1.985942
H -1.481935 -0.713045 -2.276130
H -1.573926 -2.350432 -2.914395
C -3.018132 -1.937339 -1.358085
H -3.769735 -1.778410 -2.141962
H -3.135194 -2.976277 -1.022673
C -3.296992 -0.994470 -0.204609
C -3.157432 -1.405681 1.128018
H -2.871701 -2.433614 1.340961
C -3.389454 -0.521580 2.184125
H -3.278184 -0.864540 3.209560
C -3.774197 0.794576 1.924135
H -3.965608 1.481421 2.744249
C -3.925750 1.217178 0.600935
H -4.241718 2.235420 0.387306
C -3.687146 0.330452 -0.449440
H -3.814600 0.666681 -1.476589

44
-1301.65239562 N-rad,gm
C -0.237876 -3.358936 -0.417876
N -0.609695 -1.951609 -0.427701
S -1.997870 -1.648523 0.514714
O -3.116351 -2.419288 -0.049482

O -1.609961 -1.810426 1.924839
C -2.288844 0.084289 0.198276
C -1.674248 1.042541 1.005371
C -3.142985 0.454737 -0.841761
C -1.916087 2.391725 0.753303
H -1.027722 0.734461 1.819996
C -3.370923 1.808107 -1.078083
H -3.625121 -0.307446 -1.444471
C -2.764800 2.795545 -0.287442
H -4.034342 2.103130 -1.887280
C 1.173711 -3.585278 -0.970798
H 1.253450 -4.650668 -1.224179
H 1.275200 -3.035791 -1.914262
C 2.316453 -3.234217 -0.000849
H 3.257489 -3.592155 -0.442378
H 2.177347 -3.813862 0.922896
C 2.475782 -1.750581 0.369903
C 2.848163 -0.839024 -0.817225
H 2.052514 -0.879914 -1.569960
H -1.442076 3.142729 1.380275
C -3.047363 4.258461 -0.532094
H -3.961782 4.572991 -0.011959
H -2.231392 4.891306 -0.169137
H -3.192558 4.465800 -1.597424
H 3.258492 -1.667694 1.135923
C 3.067687 0.600357 -0.402312
C 1.993684 1.499789 -0.349503
C 4.337935 1.060612 -0.030260
C 2.183012 2.818934 0.064005
H 1.001358 1.160144 -0.637087
C 4.532376 2.379223 0.384006
H 5.185044 0.378530 -0.070629
C 3.453924 3.264249 0.432757
H 1.336188 3.500427 0.092365
H 5.527286 2.716400 0.664002
H 3.603851 4.292621 0.751116
H -0.976469 -3.882775 -1.048794
H 1.556009 -1.374762 0.835074
H 3.758097 -1.228911 -1.294194
H -0.344186 -3.792412 0.589224

44
-1301.66270851 C5-rad,gm
C -0.636714 -1.868853 -0.529744
N 0.084166 -1.992110 0.745741
S 1.765757 -2.004662 0.748524
O 2.147291 -2.083999 2.159882
O 2.173535 -3.005736 -0.239072
C 2.315908 -0.403142 0.138292
C 2.503838 0.648239 1.038800
C 2.528189 -0.215217 -1.228988
C 2.899855 1.894916 0.558790
H 2.362246 0.479485 2.101385
C 2.921287 1.039656 -1.692590
H 2.408424 -1.049104 -1.912599
C 3.113867 2.112027 -0.810817
H 3.090741 1.185417 -2.756799
C -2.122026 -2.162924 -0.310554
H -2.234040 -3.197250 0.035774
H -2.507673 -1.513753 0.485102
C -2.976076 -1.950052 -1.591217
H -4.004944 -2.254987 -1.346631
H -2.621742 -2.644444 -2.365330
C -2.984687 -0.554753 -2.137029
C -3.799775 0.549597 -1.523717
H -4.804374 0.176034 -1.277624
H 3.057180 2.710985 1.260307
C 3.578953 3.456904 -1.316689
H 3.196239 4.274023 -0.696303
H 4.674855 3.523877 -1.302334
H 3.256223 3.631593 -2.348176
H -0.258376 -1.369526 1.475692
H -2.274993 -0.295067 -2.918895
C -3.190711 1.148514 -0.253244
C -3.873180 1.095984 0.968100
C -1.923245 1.749473 -0.282365
C -3.305562 1.621137 2.132091
H -4.859125 0.637886 1.009303
C -1.350804 2.272152 0.877227
H -1.378226 1.804505 -1.222037
C -2.040333 2.207497 2.091430
H -3.853026 1.569047 3.069708
H -0.363935 2.725297 0.832621
H -1.595904 2.613927 2.995982
H -0.511604 -0.868800 -0.970312
H -0.201197 -2.600327 -1.216618
H -3.937334 1.351483 -2.261916

89
-2603.87342819150000
fullTos_Nrad_PhC5.bimol.conf44_orc_rev
C 1.337648 0.687560 -3.609953
H 1.457179 5.916575 -2.182034
C 1.434248 4.868977 -2.470684
C 0.231048 4.159076 -2.449490
H -0.686764 4.653333 -2.141758
C 0.197936 2.815379 -2.823478
H -0.742934 2.270162 -2.797008
C 1.370493 2.156137 -3.223667
C 2.570821 2.877581 -3.240536
H 3.486775 2.382464 -3.556827
C 2.605574 4.223960 -2.868265
H 3.547164 4.766847 -2.893208
H 0.616617 0.553607 -4.439777
H 2.311556 0.389445 -4.022540
C 3.159581 1.589011 0.923550
H 2.950246 2.641077 0.683450
H 3.352238 1.522577 1.996778
N 1.994227 0.737382 0.647731
S 0.782946 0.551555 1.770377
O 1.430116 0.250862 3.051810
O -0.164055 -0.398600 1.160364
C -0.083117 2.111892 1.964832
C -1.100552 2.456562 1.069971
H -1.405282 1.776060 0.280461
C -1.744090 3.682355 1.226228
H -2.546314 3.946577 0.541505
C -1.388119 4.571238 2.251325
H -0.090194 4.860740 3.948650
C 0.286817 2.971460 3.001391
H 1.056461 2.671055 3.704213
C -2.077210 5.908064 2.387346
H -3.130063 5.847744 2.092391
H -2.031595 6.280998 3.415570
H -1.603081 6.661914 1.744761
C 4.385980 1.108824 0.131998
H 5.235977 1.731342 0.445583
H 4.225632 1.325446 -0.932804
C 0.966900 -0.219216 -2.470877
C 1.255110 -1.690195 -2.506373
H 0.177016 0.131464 -1.811436
H 1.644822 0.742726 -0.311831
H 2.090039 -1.875130 -3.194730
C 0.046873 -2.569136 -2.946437
H 1.591437 -2.038370 -1.517269
C -0.907309 -2.971953 -1.811589
N -1.500826 -1.882438 -1.028556
S -2.740992 -0.943318 -1.597635
O -3.112853 -1.437209 -2.929221
C -4.110956 -1.277879 -0.486107
C -3.984863 -0.985054 0.876059
H -3.056542 -0.580955 1.269166
C -5.060832 -1.230717 1.721380
H -4.965284 -1.008531 2.781761
C -6.267385 -1.759677 1.231768
C -6.364572 -2.038527 -0.135285
H -7.290123 -2.446585 -0.534031
C -5.293414 -1.802130 -1.000128
H -5.368423 -2.018315 -2.060336
C -7.423094 -2.021632 2.168297

H -8.301057 -2.389100 1.628444
H -7.158875 -2.769760 2.926136
H -7.715283 -1.110908 2.705170
H 0.429621 -3.504104 -3.379590
H -0.521042 -2.064213 -3.735598
H -0.370311 -3.592574 -1.084429
H -1.721890 -3.577643 -2.219184
H -0.916372 -1.404039 -0.342893
O -2.371943 0.471389 -1.395023
C -0.368877 4.193996 3.136193
C 4.722641 -0.381586 0.299014
H 3.906605 -0.979110 -0.121963
H 5.613705 -0.604218 -0.305261
C 4.967157 -0.824166 1.746981
H 5.815390 -0.264782 2.167818
H 4.095636 -0.574421 2.362609
C 5.257168 -2.332916 1.888394
H 5.489899 -2.540473 2.942576
H 6.163324 -2.584756 1.321562
C 4.124951 -3.241184 1.440568
C 2.837776 -3.118833 1.987670
H 2.632098 -2.350507 2.728380
C 1.804153 -3.964058 1.582900
H 0.815639 -3.846271 2.019033
C 2.037154 -4.954988 0.625028
H 1.234436 -5.621287 0.318750
C 3.311312 -5.086605 0.071371
H 3.506592 -5.852533 -0.675229
C 4.341154 -4.234518 0.476337
H 5.331503 -4.344104 0.039032

89
-2603.87305166130000
fullTos_Nrad_PhC5.bimol.conf38_orc_rev
C 1.527734 -0.149198 -3.352746
H 1.169151 5.214198 -4.059966
C 1.242291 4.138667 -3.921218
C 2.485810 3.507667 -3.962906
H 3.388135 4.088865 -4.136469
C 2.574535 2.124451 -3.786444
H 3.547011 1.637616 -3.826208
C 1.427626 1.351235 -3.567978
C 0.182215 1.996405 -3.528027
H -0.719423 1.414993 -3.348500
C 0.091382 3.377430 -3.702342
H -0.882091 3.859984 -3.669114
H 0.865852 -0.658632 -4.079326
H 2.543377 -0.493534 -3.592665
C 2.849350 2.620895 0.563793
H 2.776103 3.469415 -0.127183
H 2.887778 3.022403 1.580289
N 1.616415 1.832836 0.448052
S 0.725781 1.333695 1.752736
O 1.527804 1.458767 2.974835
O 0.147616 0.032857 1.363350
C -0.631581 2.498941 1.892547
C -0.656051 3.389805 2.963666
H 0.136272 3.363281 3.703664
C -1.714736 4.293228 3.072209
H -1.738341 4.986738 3.909272
C -2.746617 4.316805 2.127250
H -3.494940 3.394176 0.323290
C -1.652216 2.492002 0.935788
H -1.644490 1.786107 0.110799
C -3.880367 5.308786 2.234229
H -3.903682 5.787610 3.218014
H -3.782738 6.102254 1.481891
H -4.850514 4.826843 2.067792
C 4.120721 1.814202 0.262389
H 4.968374 2.514339 0.291186
H 4.067164 1.446144 -0.772712
C 1.161365 -0.572645 -1.958433
C 1.571466 -1.902016 -1.401221
H 0.304297 -0.072550 -1.514904
H 1.534031 1.200165 -0.347571
H 2.519975 -2.208885 -1.860709
C 0.531154 -3.040224 -1.623713
H 1.764253 -1.818814 -0.322006
C -0.572612 -3.124072 -0.557375
N -1.354862 -1.907035 -0.309656
S -2.515724 -1.358351 -1.354271
O -2.455641 -2.146942 -2.591432
C -4.082260 -1.742449 -0.562891
C -4.875532 -2.767762 -1.072195
H -4.549177 -3.312097 -1.951668
C -6.088608 -3.064716 -0.447647
H -6.711381 -3.861523 -0.846885
C -6.518519 -2.352518 0.677186
C -5.701591 -1.318462 1.164277
H -6.022558 -0.747099 2.032289
C -4.491267 -1.007747 0.554341
H -3.874236 -0.199090 0.933436
C -7.823415 -2.684473 1.361409
H -8.435532 -1.787389 1.511436
H -8.411036 -3.400140 0.778441
H -7.650447 -3.124788 2.351809
H 1.057941 -4.004558 -1.601716
H 0.074137 -2.946872 -2.614121
H -0.126754 -3.379701 0.410885
H -1.267707 -3.930446 -0.813296
H -0.910587 -1.162262 0.228276
O -2.400557 0.111921 -1.378929
C -2.698578 3.400267 1.063798
C 4.375642 0.643021 1.217601
H 4.399779 1.013926 2.251579
H 3.532995 -0.056217 1.169023
C 5.677104 -0.107068 0.910721
H 5.668056 -0.452026 -0.133206
H 6.526618 0.585030 0.999700
C 5.936014 -1.317123 1.837507
H 5.903103 -0.977803 2.881107
H 6.957441 -1.677736 1.659740
C 4.966044 -2.466138 1.646285
C 5.174278 -3.409328 0.628864
H 6.051179 -3.318842 -0.009507
C 4.284623 -4.465668 0.431626
H 4.472317 -5.190368 -0.356806
C 3.163147 -4.599594 1.254035
H 2.474890 -5.428581 1.110350
C 2.940900 -3.667667 2.269443
H 2.074563 -3.762838 2.918990
C 3.835715 -2.612605 2.462226
H 3.654305 -1.894243 3.258160

89
-2603.86663842340000
fullTos_Nrad_PhC5.bimol.conf44_orc_fwd
C -2.737894 -1.649767 -2.931593
H -7.444952 -1.585037 -0.237445
C -6.499043 -1.590367 -0.773598
C -5.371984 -2.193093 -0.210818
H -5.437192 -2.666711 0.766368
C -4.159781 -2.210387 -0.905559
H -3.287733 -2.679736 -0.456966
C -4.053262 -1.633613 -2.179491
C -5.194115 -1.034112 -2.732909
H -5.133745 -0.589703 -3.724763
C -6.404715 -1.008108 -2.039527
H -7.277210 -0.542801 -2.492016
H -2.112627 -2.455939 -2.534799
H -2.924444 -1.883257 -3.990094
C -0.263455 4.016558 -0.455723
H -1.304480 4.387746 -0.480731
H 0.260616 4.590581 0.317806
N -0.354888 2.606475 -0.138028
S -0.058677 2.190811 1.502302
O 0.520819 3.306345 2.266790
O 0.674466 0.912105 1.432888
C -1.729776 1.908115 2.040480
C -2.370927 0.708106 1.708773

H -1.846561 -0.087265 1.189619
C -3.700254 0.539773 2.081844
H -4.207821 -0.381168 1.810066
C -4.394849 1.539486 2.780397
H -4.242959 3.505797 3.656009
C -2.392978 2.922746 2.739056
H -1.868476 3.837307 2.994856
C -5.832745 1.326950 3.185086
H -5.896577 0.698068 4.082895
H -6.333062 2.273945 3.409959
H -6.394267 0.818806 2.394002
C 0.355655 4.222411 -1.851584
H 0.302005 5.296915 -2.074184
H -0.271688 3.710014 -2.590453
C -1.972642 -0.312621 -2.845380
C -0.652556 -0.293586 -3.639840
H -1.782470 -0.072847 -1.795211
H -2.617653 0.487423 -3.235898
H -0.887214 -0.456221 -4.702254
C 0.431878 -1.314161 -3.241867
H -0.223869 0.718046 -3.579181
C 1.165351 -1.047642 -1.921293
N 0.310487 -1.138425 -0.720794
S 0.063570 -2.597551 0.038941
O -0.176121 -3.601255 -1.005350
C 1.590954 -3.053473 0.871907
C 1.947466 -2.400682 2.056741
H 1.299222 -1.637978 2.476250
C 3.141083 -2.742687 2.685143
H 3.418288 -2.241285 3.609624
C 3.989803 -3.727709 2.152434
C 3.608533 -4.362818 0.964306
H 4.249255 -5.131376 0.538712
C 2.414124 -4.034074 0.320017
H 2.109649 -4.540136 -0.589784
C 5.266869 -4.106567 2.864240
H 5.935492 -4.677391 2.212512
H 5.808127 -3.220552 3.214491
H 5.057265 -4.725944 3.746109
H 1.203792 -1.310908 -4.025115
H 0.023184 -2.329077 -3.221813
H 1.571782 -0.030597 -1.914859
H 2.028333 -1.722756 -1.836541
H 0.443915 -0.391404 -0.034917
O -0.936398 -2.332565 1.083724
C -3.720290 2.725979 3.107938
C 1.804021 3.725239 -1.978983
H 1.822864 2.641936 -1.805924
H 2.128304 3.875385 -3.018173
C 2.804051 4.407814 -1.036270
H 2.778471 5.495191 -1.197078
H 2.507039 4.246931 0.007748
C 4.256489 3.920147 -1.222002
H 4.900505 4.493047 -0.540153
H 4.592249 4.165758 -2.237972
C 4.457884 2.435381 -0.978610
C 4.119218 1.852518 0.252413
H 3.705058 2.467705 1.047790
C 4.296442 0.486858 0.472229
H 4.011478 0.054157 1.426639
C 4.826828 -0.325483 -0.533330
H 4.962272 -1.389641 -0.359330
C 5.173379 0.241090 -1.760454
H 5.587535 -0.379093 -2.551662
C 4.985305 1.608054 -1.978615
H 5.254277 2.040871 -2.940019

89
-2603.86526739090000
fullTos_Nrad_PhC5.bimol.conf39_orc_fwd
C -2.612561 -1.659714 -2.907830
H -7.482294 -2.126876 -0.565717
C -6.504864 -2.025178 -1.031034
C -6.374967 -1.377029 -2.261762
H -7.251744 -0.966279 -2.756789
C -5.121963 -1.267799 -2.866028
H -5.032449 -0.771775 -3.830785
C -3.973972 -1.795165 -2.256775
C -4.116985 -2.440127 -1.019462
H -3.239492 -2.854357 -0.529028
C -5.370571 -2.557689 -0.413364
H -5.460288 -3.077008 0.537991
H -1.946422 -2.425567 -2.498039
H -2.700480 -1.856266 -3.986380
C 0.074143 4.191302 -0.088372
H -0.852502 4.689998 -0.426398
H 0.410348 4.710544 0.818785
N -0.291816 2.811917 0.155690
S -0.456822 2.356821 1.803165
O -0.121987 3.451652 2.725099
O 0.287187 1.083565 1.887884
C -2.206330 2.046911 1.872711
C -3.054348 3.084363 2.277628
H -2.637910 4.034798 2.594500
C -4.428295 2.866370 2.280272
H -5.094639 3.666004 2.594077
C -4.966524 1.632077 1.882979
H -4.487282 -0.351353 1.178137
C -2.711132 0.801300 1.483837
H -2.051087 -0.010918 1.198797
C -6.456660 1.395100 1.894899
H -6.771851 0.944517 2.845587
H -7.014297 2.329594 1.775410
H -6.752914 0.708720 1.095241
C 1.119845 4.285347 -1.214577
H 1.208945 5.343725 -1.494778
H 0.743056 3.751125 -2.095678
C -1.982183 -0.265568 -2.706081
C -0.610861 -0.092858 -3.386270
H -1.897273 -0.060944 -1.634900
H -2.665651 0.491787 -3.115526
H -0.741268 -0.242733 -4.468323
C 0.532631 -1.015725 -2.920846
H -0.291433 0.953100 -3.264555
C 1.141372 -0.705137 -1.547610
N 0.218539 -0.893818 -0.409202
S 0.035568 -2.390208 0.298866
O -0.079942 -3.378222 -0.781397
C 1.548473 -2.763082 1.199002
C 2.408233 -3.753367 0.724736
H 2.148497 -4.301740 -0.174325
C 3.580343 -4.032144 1.429303
H 4.248667 -4.809326 1.065956
C 3.904818 -3.335819 2.600819
C 3.023687 -2.338766 3.050599
H 3.258911 -1.786334 3.957266
C 1.848649 -2.048107 2.362813
H 1.175928 -1.275509 2.722190
C 5.153756 -3.669700 3.382040
H 4.932436 -4.377197 4.192134
H 5.915078 -4.130952 2.744890
H 5.589975 -2.777184 3.843191
H 1.357420 -0.925317 -3.642368
H 0.221638 -2.064559 -2.943839
H 1.440677 0.347624 -1.504634
H 2.059043 -1.292572 -1.409648
H 0.264668 -0.165829 0.307579
O -1.034699 -2.224115 1.291923
C -4.089443 0.608884 1.493111
C 2.490813 3.733739 -0.804987
H 2.831485 4.250908 0.104427
H 2.388419 2.674603 -0.535329
C 3.548215 3.894248 -1.903304
H 3.205372 3.395016 -2.820426
H 3.649694 4.960499 -2.150866
C 4.943315 3.347638 -1.523028
H 5.264763 3.815797 -0.583319
H 5.659055 3.669083 -2.290455
C 5.003929 1.839402 -1.388421
C 4.892717 1.211828 -0.140114

H 4.781449 1.820052 0.754850
C 4.926275 -0.179628 -0.027891
H 4.828770 -0.646364 0.948364
C 5.079565 -0.970286 -1.167961
H 5.108473 -2.052878 -1.079714
C 5.195703 -0.359485 -2.419071
H 5.322839 -0.965841 -3.312398
C 5.156704 1.031111 -2.524742
H 5.255036 1.499142 -3.502442

89
-2603.84490104310000 fullTos_Nrad_PhC5.bimol.conf76
C -2.119174 -2.748340 -2.492840
H -6.586485 -3.675699 0.432936
C -5.689014 -3.488140 -0.150806
C -5.786868 -2.968407 -1.442092
H -6.761169 -2.751354 -1.872606
C -4.630983 -2.733654 -2.188378
H -4.716750 -2.340505 -3.199707
C -3.361343 -3.008695 -1.661042
C -3.276242 -3.539392 -0.366543
H -2.306662 -3.771955 0.063792
C -4.430582 -3.773255 0.382193
H -4.342304 -4.183250 1.384768
H -1.415877 -3.585540 -2.386840
H -2.408413 -2.747229 -3.555672
C 1.381305 -2.940927 -1.198837
H 1.205086 -3.883647 -0.667097
H 0.972031 -3.070576 -2.209464
N 0.636065 -1.827424 -0.574320
S 0.097401 -2.098601 1.014374
O -0.255801 -3.509953 1.251316
O -0.921432 -1.051387 1.237596
C 1.528483 -1.730998 2.026239
C 1.817483 -0.407672 2.376801
H 1.178901 0.412828 2.068092
C 2.937697 -0.154018 3.163624
H 3.160718 0.872938 3.441229
C 3.771538 -1.192052 3.608122
H 4.074333 -3.326906 3.594657
C 2.330775 -2.787178 2.464809
H 2.070283 -3.808667 2.209578
C 4.969571 -0.893836 4.476691
H 5.535581 -0.037441 4.093896
H 4.659267 -0.643483 5.499413
H 5.646614 -1.751411 4.536946
C 2.886996 -2.641582 -1.311964
H 3.388404 -3.584695 -1.574117
H 3.269117 -2.351996 -0.326539
C -1.373800 -1.429946 -2.246085
C -2.196475 -0.205805 -1.874293
H -0.659079 -1.238129 -3.058672
H -0.465161 -1.611885 -1.301770
H -3.167013 -0.283209 -2.387403
C -1.574587 1.151718 -2.243098
H -2.447176 -0.241655 -0.806799
C -0.226009 1.484259 -1.586484
N -0.205192 1.489162 -0.121405
S -0.565039 2.826488 0.792830
O -0.036831 3.993820 0.074572
C -2.353465 3.007468 0.866473
C -3.107264 2.062035 1.570446
H -2.616989 1.235446 2.076198
C -4.491398 2.195469 1.612479
H -5.079999 1.459192 2.154611
C -5.140089 3.263839 0.971581
C -4.359976 4.197466 0.279300
H -4.843579 5.033518 -0.220203
C -2.970189 4.077622 0.221081
H -2.364497 4.805011 -0.308796
C -6.641684 3.406070 1.049380
H -6.955529 3.739093 2.047216
H -7.013561 4.137666 0.325412
H -7.144177 2.451374 0.856420
H -2.297990 1.939490 -1.999666
H -1.425922 1.194396 -3.332072
H 0.121649 2.465886 -1.917583
H 0.537264 0.760609 -1.892788
H -0.464200 0.618140 0.348027
O -0.120272 2.490878 2.151550
C 3.448389 -2.507917 3.249320
C 3.234540 -1.580111 -2.364026
H 2.907713 -1.948045 -3.347381
H 2.663945 -0.663183 -2.171029
C 4.736443 -1.249952 -2.424680
H 5.311202 -2.186138 -2.377542
H 4.970233 -0.795228 -3.395634
C 5.235455 -0.296390 -1.311383
H 6.331744 -0.346670 -1.283729
H 4.886607 -0.652022 -0.333647
C 4.812010 1.145050 -1.514983
C 5.525145 1.967768 -2.400157
H 6.397898 1.567966 -2.913765
C 5.138353 3.288685 -2.624388
H 5.709510 3.909811 -3.310261
C 4.024944 3.813422 -1.963940
H 3.722564 4.843425 -2.134201
C 3.305060 3.010560 -1.077953
H 2.437064 3.410008 -0.560611
C 3.699942 1.687755 -0.856859
H 3.135666 1.073000 -0.159291

89
-2603.84300653060000 fullTos_Nrad_PhC5.bimol.conf38
C 1.528750 -1.702129 2.861610
H -1.023790 -6.476219 2.640477
C -0.512301 -5.518046 2.680628
C 0.881135 -5.464203 2.662949
H 1.463370 -6.380686 2.609192
C 1.533001 -4.230278 2.716554
H 2.620789 -4.196295 2.709996
C 0.808713 -3.034003 2.787098
C -0.592763 -3.100021 2.806433
H -1.179135 -2.185710 2.855406
C -1.245652 -4.331194 2.752128
H -2.332080 -4.362618 2.768639
H 1.281874 -1.211767 3.820159
H 2.614104 -1.866822 2.890677
C 2.868266 -2.491410 -0.764483
H 2.886361 -3.427289 -0.193838
H 2.962468 -2.748761 -1.826521
N 1.522843 -1.934202 -0.559922
S 0.836480 -0.987398 -1.766044
O 1.694419 -0.934379 -2.960861
O 0.380918 0.278458 -1.151777
C -0.629986 -1.923808 -2.204390
C -0.694739 -2.526741 -3.459084
H 0.136441 -2.424906 -4.148158
C -1.840827 -3.241203 -3.811039
H -1.896370 -3.708059 -4.791449
C -2.918791 -3.362876 -2.926726
H -3.654776 -2.814587 -0.971741
C -1.693099 -2.021320 -1.300549
H -1.649316 -1.541028 -0.328406
C -4.147204 -4.158404 -3.299212
H -4.186279 -4.357202 -4.374735
H -4.160526 -5.127381 -2.783233
H -5.065964 -3.631954 -3.016914
C 4.072825 -1.627983 -0.341129
H 4.962404 -2.268235 -0.435218
H 3.983949 -1.393651 0.728885
C 1.189773 -0.706338 1.759418
C 1.905971 0.636551 1.830963
H 0.110913 -0.607872 1.612331
H 1.438331 -1.281560 0.640382
H 2.902203 0.488274 2.267011
C 1.134889 1.702951 2.654996
H 2.067656 1.030155 0.820603
C 0.211103 2.605245 1.820589
N -0.830017 1.935374 1.027054

S -2.209489 1.343252 1.746780
O -2.248950 1.856810 3.121732
C -3.551277 2.107066 0.832228
C -4.209717 3.206182 1.381115
H -3.920265 3.572956 2.359985
C -5.247617 3.803292 0.665739
H -5.768639 4.655363 1.095532
C -5.634095 3.321367 -0.591039
C -4.958636 2.207979 -1.114482
H -5.252185 1.810868 -2.083370
C -3.923862 1.595536 -0.413376
H -3.419989 0.725586 -0.822249
C -6.739410 3.988731 -1.374575
H -7.380496 3.251676 -1.870660
H -7.370402 4.608478 -0.729887
H -6.329623 4.639682 -2.158130
H 1.860075 2.366178 3.145682
H 0.554839 1.230316 3.456917
H 0.809892 3.172579 1.098927
H -0.285617 3.325409 2.475733
H -0.503249 1.343296 0.260155
O -2.297375 -0.107095 1.482171
C -2.826140 -2.738691 -1.671804
C 4.288474 -0.336772 -1.137484
H 4.342431 -0.572309 -2.208177
H 3.419737 0.320004 -1.020447
C 5.555458 0.415847 -0.713021
H 5.525337 0.617222 0.367537
H 6.432567 -0.224683 -0.883423
C 5.771012 1.749495 -1.465857
H 5.743941 1.554327 -2.545960
H 6.780533 2.116371 -1.240623
C 4.764302 2.827307 -1.116714
C 4.985635 3.676872 -0.022524
H 5.900243 3.569893 0.557753
C 4.059913 4.660841 0.325364
H 4.257471 5.314040 1.171762
C 2.887890 4.814001 -0.419619
H 2.169669 5.586871 -0.158086
C 2.652274 3.974480 -1.510388
H 1.744519 4.083506 -2.097810
C 3.583937 2.993028 -1.855192
H 3.389647 2.346204 -2.707279

89
-2603.84290651670000 fullTos_Nrad_PhC5.bimol.conf39
C 1.530790 -1.699202 2.862055
H -1.016715 -6.476036 2.641892
C -0.506235 -5.517317 2.681871
C 0.887143 -5.462005 2.664169
H 1.470345 -6.377884 2.610574
C 1.537708 -4.227382 2.717543
H 2.625460 -4.192254 2.710972
C 0.812159 -3.031858 2.787875
C -0.589246 -3.099355 2.807255
H -1.176587 -2.185659 2.856059
C -1.240836 -4.331227 2.753178
H -2.327230 -4.363792 2.769707
H 1.283394 -1.208856 3.820478
H 2.616318 -1.862725 2.891149
C 2.869926 -2.489395 -0.763697
H 2.888459 -3.425139 -0.192849
H 2.964267 -2.746898 -1.825685
N 1.524283 -1.932669 -0.559246
S 0.837323 -0.986896 -1.765830
O 1.695417 -0.933340 -2.960515
O 0.380456 0.278686 -1.151974
C -0.628148 -1.924844 -2.204219
C -0.692356 -2.527602 -3.459050
H 0.138677 -2.424745 -4.148150
C -1.837711 -3.243154 -3.811081
H -1.892853 -3.709825 -4.791606
C -2.915506 -3.366131 -2.926701
H -3.652017 -2.818852 -0.971666
C -1.691110 -2.023583 -1.300365
H -1.647802 -1.543359 -0.328168
C -4.142843 -4.163305 -3.299209
H -5.062150 -3.641768 -3.009787
H -4.185646 -4.355498 -4.375802
H -4.150750 -5.135633 -2.789417
C 4.074041 -1.625230 -0.340550
H 4.964007 -2.264944 -0.434601
H 3.985087 -1.390759 0.729427
C 1.190696 -0.704070 1.759608
C 1.905519 0.639579 1.830682
H 0.111714 -0.606787 1.612631
H 1.439629 -1.279388 0.640718
H 2.901856 0.492473 2.266887
C 1.133290 1.705503 2.654255
H 2.066920 1.032985 0.820196
C 0.208342 2.606280 1.819489
N -0.832034 1.934777 1.026383
S -2.210828 1.341443 1.746351
O -2.250549 1.854954 3.121315
C -3.553466 2.104066 0.832039
C -4.212983 3.202474 1.381172
H -3.923847 3.569323 2.360107
C -5.251521 3.798625 0.666021
H -5.773423 4.650051 1.096047
C -5.637615 3.316513 -0.590865
C -4.961178 2.203866 -1.114474
H -5.254505 1.806540 -2.083331
C -3.925676 1.592338 -0.413542
H -3.421058 0.722882 -0.822550
C -6.743210 3.983556 -1.374273
H -7.375062 3.246996 -1.882664
H -7.383289 4.591222 -0.726999
H -6.333548 4.646548 -2.147771
H 1.857772 2.369848 3.144471
H 0.553924 1.232599 3.456510
H 0.806414 3.173929 1.097485
H -0.289143 3.326213 2.474308
H -0.504653 1.342806 0.259665
O -2.297385 -0.108974 1.481714
C -2.823455 -2.742048 -1.671711
C 4.288713 -0.334021 -1.137161
H 4.342891 -0.569742 -2.207802
H 3.419416 0.322037 -1.020292
C 5.555011 0.419830 -0.712852
H 5.524674 0.621474 0.367650
H 6.432716 -0.219932 -0.883064
C 5.769322 1.753479 -1.466051
H 5.742546 1.557962 -2.546099
H 6.778452 2.121431 -1.240827
C 4.761491 2.830383 -1.117323
C 3.580853 2.994452 -1.855742
H 3.387159 2.346997 -2.707485
C 2.648147 3.975039 -1.511295
H 1.740195 4.082770 -2.098653
C 2.882976 4.815352 -0.420963
H 2.163939 5.587561 -0.159718
C 4.055258 4.663845 0.323949
H 4.252201 5.317671 1.170006
C 4.982019 3.680721 -0.023571
H 5.896811 3.575025 0.556651

89
-2603.84043263350000 fullTos_Nrad_PhC5.bimol.conf44
C 1.519864 -1.449712 3.040324
H 0.098110 -6.609164 2.196410
C 0.383473 -5.573501 2.361665
C -0.591546 -4.574302 2.412524
H -1.640552 -4.830319 2.287526
C -0.229354 -3.244866 2.627543
H -1.000130 -2.478550 2.658454
C 1.118193 -2.890224 2.792994
C 2.086424 -3.900441 2.741933
H 3.134993 -3.643045 2.878288
C 1.725574 -5.232477 2.527250
H 2.493596 -6.001112 2.492535

H 1.045913 -1.099587 3.974544
H 2.600760 -1.388655 3.222229
C 3.281876 -1.839766 -0.583361
H 3.320420 -2.910258 -0.347587
H 3.538964 -1.717224 -1.640805
N 1.881154 -1.427669 -0.392418
S 1.123786 -0.521800 -1.578176
O 2.050388 -0.146856 -2.662139
O 0.346078 0.539706 -0.907471
C -0.052975 -1.684048 -2.273904
C -1.161914 -2.082630 -1.519497
H -1.324397 -1.698911 -0.517261
C -2.069932 -2.974477 -2.081474
H -2.934794 -3.283715 -1.499354
C -1.891365 -3.480272 -3.379818
H -0.623780 -3.429336 -5.121255
C 0.148328 -2.163578 -3.566866
H 1.007228 -1.828367 -4.137816
C -2.875105 -4.468069 -3.960262
H -3.909721 -4.166573 -3.761251
H -2.752583 -4.568057 -5.043176
H -2.740011 -5.464142 -3.518789
C 4.335495 -1.126338 0.288893
H 5.281944 -1.660860 0.121632
H 4.079762 -1.294483 1.343046
C 1.121736 -0.459238 1.952940
C 1.521197 0.990854 2.189998
H 0.072661 -0.569967 1.665739
H 1.612125 -0.881055 0.848910
H 2.486397 1.017577 2.710968
C 0.469462 1.794680 3.003190
H 1.673642 1.496632 1.229492
C -0.545355 2.562500 2.140450
N -1.351586 1.767221 1.202863
S -2.631602 0.845934 1.737865
O -2.882939 1.202257 3.139943
C -4.027250 1.415352 0.764324
C -4.183198 0.972528 -0.551558
H -3.476194 0.270001 -0.981103
C -5.264392 1.433388 -1.297148
H -5.389677 1.088916 -2.320980
C -6.198518 2.326647 -0.749962
C -6.023661 2.742338 0.575853
H -6.744843 3.422078 1.023124
C -4.943804 2.295388 1.337260
H -4.816875 2.608554 2.367938
C -7.352890 2.836532 -1.579670
H -8.157843 3.227766 -0.949670
H -7.031516 3.648912 -2.244835
H -7.770521 2.046784 -2.213753
H 0.989901 2.541900 3.617515
H -0.068557 1.141436 3.700528
H -0.017028 3.296754 1.520727
H -1.234625 3.111633 2.786926
H -0.841388 1.315601 0.440797
O -2.395618 -0.558397 1.346933
C -0.774741 -3.058081 -4.110537
C 4.552460 0.375649 0.056122
H 3.616936 0.917237 0.231564
H 5.253125 0.732817 0.825227
C 5.096245 0.753405 -1.327657
H 6.000301 0.165066 -1.542779
H 4.361741 0.490415 -2.095704
C 5.459343 2.249430 -1.450920
H 5.811076 2.433520 -2.476274
H 6.312629 2.466930 -0.794734
C 4.335953 3.218547 -1.123424
C 3.081972 3.121033 -1.746748
H 2.892835 2.327823 -2.464302
C 2.057895 4.017144 -1.437636
H 1.092515 3.913737 -1.925946
C 2.268278 5.036534 -0.505205
H 1.472038 5.738814 -0.271171
C 3.511212 5.146990 0.119460
H 3.689340 5.934827 0.847418
C 4.530352 4.242625 -0.186753
H 5.494911 4.333680 0.308798

TS-1,6'-HAT$_{bi}$ pathway

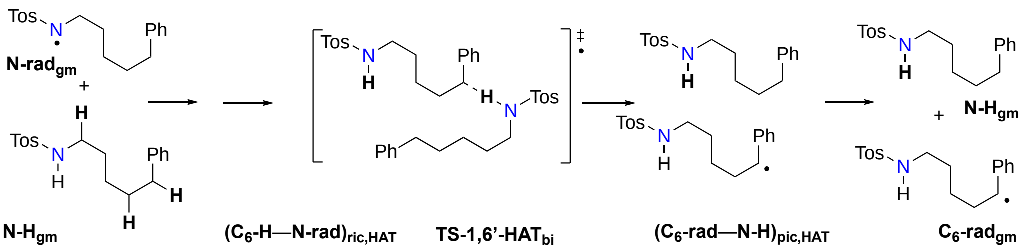

Name	E(B3LYP)	G(B3LYP)	E(RO-B2PLYP-D3)	G(RO-B2PLYP-D3)
**N-H$_{gm}$**				
NH.conf15	-1303.10330570	-1302.778657	-1302.5831192072	-1302.2584705072
**N-rad$_{gm}$**				
N-rad_0007	-1302.44553888	-1302.137918	-1301.9129157	-1301.6052948456
**C$_6$-rad$_{gm}$**				
TScrad15crad.C6.conf059	-1302.45907388	-1302.147252	-1301.9349086884	-1301.6230868084
**N-H$_{gm}$ + N-rad$_{gm}$**				
Summed	-2605.54884458	-2604.916575	-2604.4960349328	-2603.8637653528
**(C6-H—N-rad)$_{ric,HAT}$**				
fullTos_Nrad_PhC6.bimol.conf95_orc_rev	-2605.56567565	-2604.914618	-2604.5158559677	-2603.8647983177
fullTos_Nrad_PhC6.bimol.conf05_orc_fwd	-2605.56242949	-2604.904726	-2604.5210089479	-2603.8633054579
fullTos_Nrad_PhC6.bimol.conf52_orc_rev	-2605.56386476	-2604.916860	-2604.5093310126	-2603.86232625260
fullTos_Nrad_PhC6.bimol.conf54_orc_rev	-2605.56386484	-2604.916219	-2604.5093324497	-2603.8616866097
**TS-1,6'-HAT$_{bi}$**				
fullTos_Nrad_PhC6.bimol.conf82	-2605.53606815	-2604.880260	-2604.5012053803	-2603.8453972303
fullTos_Nrad_PhC6.bimol.conf52	-2605.53606811	-2604.879875	-2604.5012098306	-2603.8450167206
fullTos_Nrad_PhC6.bimol.conf54	-2605.53606738	-2604.877429	-2604.5012125609	-2603.8425741809
**(C6-rad—N-H)$_{pic,HAT}$**				
fullTos_Nrad_PhC6.bimol.conf94_orc_fwd	-2605.58688458	-2604.933710	-2604.5445847742	-2603.8914101942
fullTos_Nrad_PhC6.bimol.conf54_orc_fwd	-2605.58688879	-2604.933167	-2604.5445899986	-2603.8908682086
fullTos_Nrad_PhC6.bimol.conf82_orc_fwd	-2605.58499237	-2604.933642	-2604.5413363954	-2603.8899860254
fullTos_Nrad_PhC6.bimol.conf95_orc_fwd	-2605.58688502	-2604.931031	-2604.5445824588	-2603.8887284388

45
-1302.25847050720000 NH.conf15
H 1.370625 -2.076475 1.152152
C 2.092476 -2.425601 -0.820792
H 3.049714 -2.192621 -1.295165
H 2.044751 -3.518341 -0.728351
N 2.167212 -1.902447 0.547934
S 2.930734 -0.465503 0.937328
O 4.199387 -0.458331 0.205194
O 2.855505 -0.392931 2.398183
C 1.947843 0.897220 0.292265
C 0.740967 1.231958 0.913472
H 0.416180 0.700707 1.802750
C -0.034201 2.261079 0.387853
H -0.980821 2.507080 0.861769
C 0.383528 2.977840 -0.744772
C 1.603599 2.632483 -1.339441
H 1.947453 3.181164 -2.213002
C 2.388466 1.596696 -0.830886
H 3.336200 1.333252 -1.288026
C -0.455279 4.110146 -1.287872
H -0.326197 5.019935 -0.687011
H -0.180233 4.355817 -2.318354
H -1.521877 3.860282 -1.269518
C 0.920001 -1.925285 -1.680865
H 1.034785 -0.851773 -1.874701
H 0.992274 -2.426992 -2.656634
C -0.462033 -2.188287 -1.069147
H -0.563167 -3.258959 -0.831999
H -0.550079 -1.647558 -0.117739
C -1.615993 -1.764294 -1.985942
H -1.481935 -0.713045 -2.276130
H -1.573926 -2.350432 -2.914395
C -3.018132 -1.937339 -1.358085
H -3.769735 -1.778410 -2.141962
H -3.135194 -2.976277 -1.022673
C -3.296992 -0.994470 -0.204609
C -3.157432 -1.405681 1.128018
H -2.871701 -2.433614 1.340961
C -3.389454 -0.521580 2.184125
H -3.278184 -0.864540 3.209560
C -3.774197 0.794576 1.924135
H -3.965608 1.481421 2.744249
C -3.925750 1.217178 0.600935

H -4.241718 2.235420 0.387306
C -3.687146 0.330452 -0.449440
H -3.814600 0.666681 -1.476589
44
-1301.65239562 N-rad,gm
C -0.237876 -3.358936 -0.417876
N -0.609695 -1.951609 -0.427701
S -1.997870 -1.648523 0.514714
O -3.116351 -2.419288 -0.049482
O -1.609961 -1.810426 1.924839
C -2.288844 0.084289 0.198276
C -1.674248 1.042541 1.005371
C -3.142985 0.454737 -0.841761
C -1.916087 2.391725 0.753303
H -1.027722 0.734461 1.819996
C -3.370923 1.808107 -1.078083
H -3.625121 -0.307446 -1.444471
C -2.764800 2.795545 -0.287442
H -4.034342 2.103130 -1.887280
C 1.173711 -3.585278 -0.970798
H 1.253450 -4.650668 -1.224179
H 1.275200 -3.035791 -1.914262
C 2.316453 -3.234217 -0.000849
H 3.257489 -3.592155 -0.442378
H 2.177347 -3.813862 0.922896
C 2.475782 -1.750581 0.369903
C 2.848163 -0.839024 -0.817225
H 2.052514 -0.879914 -1.569960
H -1.442076 3.142729 1.380275
C -3.047363 4.258461 -0.532094
H -3.961782 4.572991 -0.011959
H -2.231392 4.891306 -0.169137
H -3.192558 4.465800 -1.597424
H 3.258492 -1.667694 1.135923
C 3.067687 0.600357 -0.402312
C 1.993684 1.499789 -0.349503
C 4.337935 1.060612 -0.030260
C 2.183012 2.818934 0.064005
H 1.001358 1.160144 -0.637087
C 4.532376 2.379223 0.384006
H 5.185044 0.378530 -0.070629
C 3.453924 3.264249 0.432757
H 1.336188 3.500427 0.092365
H 5.527286 2.716400 0.664002
H 3.603851 4.292621 0.751116
H -0.976469 -3.882775 -1.048794
H 1.556009 -1.374762 0.835074
H 3.758097 -1.228911 -1.294194
H -0.344186 -3.792412 0.589224

44
-1301.62308680840000 TScrad15crad.C6.conf059
S 1.346150 -1.435409 -0.962068
N 0.581517 -2.096293 0.377700
C 2.492765 -0.182300 -0.374786
C 2.054143 1.130983 -0.190616
H 1.035944 1.404759 -0.446496
C 2.945897 2.082681 0.301329
H 2.606482 3.105742 0.443904
C 4.272297 1.747174 0.606297
C 5.240804 2.793162 1.104545
C 4.687659 0.422453 0.406136
H 5.714930 0.144489 0.629457
C 3.810251 -0.542986 -0.081836
H 4.142074 -1.561507 -0.255200
O 2.136456 -2.530677 -1.527404
O 0.306183 -0.739344 -1.726776
C -0.256114 -1.255455 1.245844
C -1.359070 -2.075357 1.926383
H 5.789928 3.248787 0.269928
H 5.983267 2.361433 1.783805
H 4.723194 3.600181 1.632920
H -0.904854 -2.786287 2.631365
C -2.284634 -2.842162 0.968084
H -1.946153 -1.373994 2.533720
H 0.347967 -0.736115 2.004508
H -0.707511 -0.490999 0.610044
C -2.940617 -1.981117 -0.137870
C -3.757712 -0.842231 0.388131
H -3.069350 -3.325078 1.566438
H -1.720738 -3.646450 0.481883
H -3.590190 -2.651682 -0.724756
H -2.169173 -1.636290 -0.832095
H 1.200956 -2.745003 0.857317
H -4.522601 -1.100367 1.121687
C -3.659063 0.524766 0.032470
C -4.530893 1.475513 0.646103
H -5.256424 1.119982 1.374510
C -4.470660 2.823714 0.333508
H -5.150272 3.519734 0.819203
C -3.541253 3.293491 -0.605912
H -3.496608 4.350545 -0.852765
C -2.674663 2.383255 -1.225789
H -1.955529 2.736345 -1.961037
C -2.723606 1.029375 -0.922460
H -2.038651 0.352781 -1.423080

89
-2603.89141019420000
fullTos_Nrad_PhC6.bimol.conf94_orc_fwd
C 5.210981 1.099564 0.264623
H 6.911948 5.929871 -1.253919
C 6.574648 4.942742 -0.950143
C 7.334591 3.808152 -1.264093
H 8.266758 3.917587 -1.813398
C 6.912969 2.543143 -0.880048
H 7.523839 1.681228 -1.131091
C 5.697131 2.359512 -0.157096
C 4.942310 3.533243 0.150754
H 4.009181 3.418310 0.697138
C 5.375019 4.790203 -0.239150
H 4.778160 5.664572 0.009193
H -0.016621 0.574622 -0.672730
H 4.284962 1.100923 0.837629
C -0.226269 2.714887 -0.531407
H 0.300997 3.456517 0.074616
H 0.042070 2.913288 -1.576559
N 0.352352 1.404983 -0.199402
S 0.941202 1.057632 1.302643
O 1.702506 2.220245 1.772651
O 1.602853 -0.259335 1.161616
C -0.424464 0.817661 2.447614
C -0.543318 1.655466 3.554756
H 0.189560 2.438304 3.716707
C -1.604483 1.462832 4.441178
H -1.696928 2.111600 5.308631
C -2.548311 0.450513 4.233652
C -2.401621 -0.378962 3.109057
H -3.122851 -1.173636 2.933073
C -1.349944 -0.206299 2.215047
H -1.252519 -0.852325 1.347144
C -3.708605 0.259105 5.180991
H -3.563877 0.817852 6.110573
H -4.647642 0.604533 4.729487
H -3.844868 -0.797618 5.437564
C -1.746217 2.835631 -0.347292
H -2.006299 2.618623 0.696864
H -2.017341 3.886986 -0.520978
C 5.836187 -0.244806 0.045978
C 4.803558 -1.310384 -0.382248
H 6.305092 -0.586940 0.984448
H 6.645457 -0.200207 -0.693631
H 5.251735 -2.309817 -0.296997
H 3.969904 -1.282976 0.331117
C -2.565205 1.941000 -1.288021
H -2.325101 2.199841 -2.330237
H -2.273093 0.889753 -1.161862
C -4.077526 2.068653 -1.066664
H -4.322277 1.789202 -0.032331
H -4.383817 3.118381 -1.178895

C -4.904111 1.196050 -2.032872
H -4.668729 1.487679 -3.065125
H -4.588234 0.150074 -1.926819
C -6.395892 1.305801 -1.799185
C -7.148689 2.314862 -2.415746
H -6.656474 3.004512 -3.098727
C -8.516730 2.442657 -2.172480
H -9.081748 3.229953 -2.665243
C -9.159733 1.558131 -1.304463
H -10.226014 1.653127 -1.116814
C -8.423751 0.546550 -0.685104
H -8.916026 -0.152031 -0.013019
C -7.055650 0.424074 -0.932273
H -6.489979 -0.371382 -0.451040
C 4.291858 -1.111454 -1.818529
H 5.100114 -1.347396 -2.523156
H 4.030039 -0.058938 -1.984391
C 3.079046 -1.979003 -2.176374
H 2.922581 -1.997960 -3.258030
H 3.246611 -3.015757 -1.843387
N 1.866513 -1.404107 -1.568646
H 1.965971 -0.994864 -0.634510
S 0.408540 -2.169023 -1.734006
O -0.568707 -1.278414 -1.066342
O 0.241126 -2.535765 -3.141029
C 0.465103 -3.692108 -0.780196
C 0.677476 -3.637503 0.602410
H 0.814057 -2.683281 1.103394
C 0.717455 -4.824864 1.325836
H 0.881919 -4.786773 2.400192
C 0.548831 -6.069560 0.696168
C 0.338429 -6.092446 -0.687106
H 0.203007 -7.045419 -1.192530
C 0.298720 -4.912373 -1.432036
H 0.133844 -4.929044 -2.503923
C 0.609790 -7.347601 1.498370
H 1.629843 -7.543358 1.853104
H -0.033261 -7.293706 2.384472
H 0.294404 -8.210072 0.903531

89
-2603.89086820860000
fullTos_Nrad_PhC6.bimol.conf54_orc_fwd
C 5.211518 1.097985 0.265121
H 6.913590 5.927496 -1.254757
C 6.576063 4.940525 -0.950718
C 7.335581 3.805647 -1.264640
H 8.267648 3.914692 -1.814192
C 6.913674 2.540835 -0.880253
H 7.524231 1.678694 -1.131280
C 5.697961 2.357699 -0.156968
C 4.943571 3.531727 0.150842
H 4.010533 3.417173 0.697460
C 5.376560 4.788482 -0.239401
H 4.780024 5.663078 0.008919
H -0.016171 0.574069 -0.672017
H 4.285635 1.099775 0.838343
C -0.224778 2.714497 -0.531227
H 0.302689 3.455979 0.074803
H 0.043898 2.912612 -1.576347
N 0.353130 1.404375 -0.198855
S 0.941907 1.057108 1.303222
O 1.703902 2.219406 1.772890
O 1.602777 -0.260289 1.162535
C -0.423828 0.818252 2.448330
C -0.542139 1.656397 3.555204
H 0.191142 2.438913 3.716881
C -1.603507 1.464704 4.441677
H -1.695658 2.113885 5.308842
C -2.547901 0.452936 4.234473
C -2.401856 -0.376835 3.109935
H -3.123780 -1.170902 2.933985
C -1.350071 -0.205135 2.215954
H -1.253163 -0.851290 1.348086
C -3.707575 0.261269 5.182523
H -4.650361 0.585943 4.723646
H -3.829675 -0.792983 5.456213
H -3.572363 0.836848 6.103191
C -1.744711 2.835947 -0.347479
H -2.005134 2.619124 0.696632
H -2.015330 3.887409 -0.521304
C 5.836221 -0.246613 0.046469
C 4.803181 -1.311781 -0.381813
H 6.304976 -0.588959 0.984932
H 6.645515 -0.202301 -0.693130
H 5.250963 -2.311392 -0.296616
H 3.969527 -1.284093 0.331538
C -2.563865 1.941616 -1.288352
H -2.323400 2.200293 -2.330524
H -2.272238 0.890250 -1.162062
C -4.076187 2.069924 -1.067376
H -4.321314 1.790632 -0.033089
H -4.382008 3.119773 -1.179740
C -4.902881 1.197610 -2.033753
H -4.667099 1.489086 -3.065958
H -4.587464 0.151509 -1.927562
C -6.394683 1.307978 -1.800486
C -7.146864 2.317428 -2.417166
H -6.654149 3.006940 -3.099924
C -8.514922 2.445781 -2.174298
H -9.079455 3.233372 -2.667146
C -9.158566 1.561440 -1.306566
H -10.224862 1.656874 -1.119228
C -8.423204 0.549481 -0.687093
H -8.915980 -0.148957 -0.015225
C -7.055079 0.426447 -0.933865
H -6.489891 -0.369301 -0.452544
C 4.291598 -1.112531 -1.818090
H 5.099815 -1.348635 -2.522710
H 4.030101 -0.059917 -1.983845
C 3.078558 -1.979674 -2.176124
H 2.922091 -1.998346 -3.257785
H 3.245834 -3.016536 -1.843339
N 1.866171 -1.404584 -1.568241
H 1.965859 -0.995766 -0.633935
S 0.408089 -2.169368 -1.733450
O -0.568905 -1.278827 -1.065305
O 0.240427 -2.535719 -3.140548
C 0.463811 -3.692702 -0.779967
C 0.674300 -3.638401 0.602758
H 0.810982 -2.684362 1.104056
C 0.718281 -4.826369 1.325309
H 0.886055 -4.788819 2.399139
C 0.552518 -6.070878 0.694944
C 0.348570 -6.093738 -0.689500
H 0.223276 -7.047162 -1.196741
C 0.304880 -4.913342 -1.433348
H 0.146753 -4.930004 -2.506250
C 0.580404 -7.347796 1.500706
H 1.411356 -7.351706 2.215140
H -0.343731 -7.468233 2.080908
H 0.682298 -8.226568 0.856709

89
-2603.88998602540000
fullTos_Nrad_PhC6.bimol.conf82_orc_fwd
C 5.594791 1.185718 0.711321
H 9.329735 4.693444 -0.796741
C 8.574107 3.977423 -0.485797
C 8.850405 3.054907 0.531629
H 9.825804 3.057009 1.012326
C 7.890826 2.136694 0.935134
H 8.124233 1.432163 1.727980
C 6.601113 2.105180 0.328609
C 6.346768 3.056368 -0.703145
H 5.370785 3.053690 -1.182913
C 7.312441 3.968296 -1.098359
H 7.088733 4.680732 -1.888634
H -0.464579 -0.294769 1.539266
H 4.635053 1.252914 0.201617

C -1.325216 0.624264 3.285147
H -1.657187 0.201917 4.237361
H -0.456369 1.257051 3.506052
N -0.823980 -0.495948 2.476519
S -1.473513 -2.013018 2.580328
O -1.719484 -2.303996 3.994260
O -0.567294 -2.868185 1.780853
C -3.067164 -2.042736 1.745590
C -4.208760 -2.371438 2.474413
H -4.121751 -2.606014 3.529681
C -5.444942 -2.398974 1.826520
H -6.335993 -2.659932 2.392160
C -5.556558 -2.098422 0.464183
C -4.388460 -1.772227 -0.244945
H -4.454044 -1.539900 -1.305364
C -3.146235 -1.742315 0.380951
H -2.253164 -1.483691 -0.180451
C -6.898136 -2.104604 -0.229096
H -7.661054 -2.601478 0.377896
H -7.245582 -1.081929 -0.425295
H -6.846157 -2.617601 -1.196120
C -2.436699 1.467158 2.642194
H -3.297512 0.825064 2.415166
H -2.780857 2.189924 3.395980
C 5.735158 0.144924 1.783945
C 4.547574 -0.830060 1.842292
H 5.841817 0.633036 2.768044
H 6.669960 -0.423128 1.646352
H 4.640790 -1.462019 2.736385
H 3.618896 -0.258943 1.960816
C -2.000729 2.223528 1.379779
H -1.162227 2.890509 1.630903
H -1.614441 1.519435 0.630367
C -3.135052 3.044791 0.754257
H -3.966038 2.377861 0.485134
H -3.537924 3.749273 1.495640
C -2.692780 3.828743 -0.497967
H -1.870021 4.502417 -0.223668
H -2.283884 3.123665 -1.233429
C -3.817319 4.625835 -1.124198
C -4.622932 4.069078 -2.126839
H -4.410349 3.062682 -2.482237
C -5.684466 4.787410 -2.678976
H -6.293109 4.337699 -3.459589
C -5.959776 6.082211 -2.235861
H -6.783347 6.645195 -2.667086
C -5.164512 6.651030 -1.239265
H -5.366112 7.661131 -0.891511
C -4.104411 5.927810 -0.691323
H -3.485486 6.380896 0.080675
C 4.435122 -1.718220 0.597478
H 5.299387 -2.396070 0.545076
H 4.466161 -1.101154 -0.305947
C 3.169945 -2.582499 0.566125
H 3.189774 -3.250646 -0.307335
H 3.127998 -3.223970 1.453281
N 1.918015 -1.797926 0.603825
H 1.153638 -2.210861 1.148967
S 1.320422 -1.093452 -0.774459
O 0.029105 -0.494773 -0.367170
O 2.362062 -0.250869 -1.368274
C 0.960609 -2.405496 -1.950373
C 0.071611 -3.428476 -1.598954
H -0.390424 -3.446960 -0.615802
C -0.205606 -4.427389 -2.526489
H -0.894781 -5.224468 -2.257465
C 0.386724 -4.426315 -3.800598
C 1.271881 -3.391216 -4.122257
H 1.740198 -3.372211 -5.103150
C 1.566629 -2.380964 -3.205078
H 2.253183 -1.578912 -3.453327
C 0.080256 -5.527400 -4.787565
H -1.000671 -5.661693 -4.911901
H 0.509767 -5.315560 -5.771267
H 0.486010 -6.488299 -4.446451

89
-2603.88872843880000
fullTos_Nrad_PhC6.bimol.conf95_orc_fwd
C 5.211720 1.095504 0.260438
H 6.924408 5.924643 -1.248588
C 6.584728 4.937744 -0.946718
C 7.343510 3.802234 -1.260120
H 8.277174 3.910706 -1.807068
C 6.918842 2.537507 -0.878488
H 7.528876 1.674839 -1.128986
C 5.700975 2.355132 -0.158657
C 4.947414 3.529785 0.148748
H 4.012838 3.415826 0.692862
C 5.383164 4.786444 -0.238728
H 4.787205 5.661547 0.009191
H -0.017583 0.574512 -0.675036
H 4.284535 1.097732 0.831539
C -0.226564 2.714993 -0.536017
H 0.301230 3.457158 0.068891
H 0.041131 2.912269 -1.581548
N 0.352044 1.405326 -0.203017
S 0.941960 1.059586 1.298993
O 1.704164 2.222435 1.766936
O 1.602946 -0.257848 1.159075
C -0.422859 0.821659 2.445396
C -0.540282 1.660804 3.551665
H 0.193141 2.443454 3.712043
C -1.600793 1.469788 4.439233
H -1.692168 2.119644 5.305981
C -2.545343 0.457747 4.233719
C -2.400136 -0.373064 3.109906
H -3.122013 -1.167480 2.935396
C -1.349141 -0.202033 2.214794
H -1.252892 -0.849069 1.347510
C -3.704672 0.267700 5.182514
H -3.561788 0.832559 6.108670
H -4.645167 0.606664 4.729199
H -3.836755 -0.787989 5.445570
C -1.746370 2.836174 -0.351036
H -2.005820 2.620146 0.693480
H -2.017425 3.887420 -0.525490
C 5.835620 -0.249499 0.041870
C 4.801842 -1.314390 -0.385143
H 6.305125 -0.591672 0.980048
H 6.644326 -0.205792 -0.698435
H 5.249206 -2.314165 -0.299700
H 3.968644 -1.285939 0.328717
C -2.566123 1.940860 -1.290453
H -2.326730 2.198835 -2.333047
H -2.274051 0.889680 -1.163649
C -4.078273 2.068860 -1.068124
H -4.322323 1.790181 -0.033418
H -4.384520 3.118543 -1.180894
C -4.905630 1.195652 -2.033123
H -4.670952 1.486536 -3.065749
H -4.589776 0.149723 -1.926561
C -6.397237 1.305697 -1.798459
C -7.150255 2.314811 -2.414671
H -6.658362 3.004280 -3.098066
C -8.518112 2.442882 -2.170535
H -9.083311 3.230212 -2.663038
C -9.160712 1.558588 -1.301979
H -10.226854 1.653801 -1.113655
C -8.424511 0.546963 -0.682961
H -8.916472 -0.151439 -0.010460
C -7.056588 0.424210 -0.931001
H -6.490746 -0.371281 -0.450025
C 4.289420 -1.115733 -1.821203
H 5.097083 -1.352581 -2.526205
H 4.028297 -0.063090 -1.987394
C 3.075758 -1.982544 -2.177938
H 2.918667 -2.001938 -3.259495
H 3.242716 -3.019250 -1.844519
N 1.864021 -1.406436 -1.569752

H 1.964472 -0.995991 -0.636250
S 0.405529 -2.170747 -1.733040
O -0.570743 -1.278738 -1.065785
O 0.236895 -2.539217 -3.139465
C 0.461797 -3.692571 -0.777230
C 0.674537 -3.636131 0.605382
H 0.811738 -2.681247 1.104930
C 0.715231 -4.822437 1.330226
H 0.880901 -4.782948 2.404370
C 0.546395 -6.068098 0.702178
C 0.336609 -6.092858 -0.681000
H 0.202606 -7.046585 -1.185336
C 0.296143 -4.913627 -1.427493
H 0.132039 -4.931839 -2.499473
C 0.600970 -7.344246 1.507866
H -0.109487 -7.318801 2.342728
H 0.366069 -8.217670 0.892268
H 1.597964 -7.497907 1.939611

89
-2603.86479831770000
fullTos_Nrad_PhC6.bimol.conf95_orc_rev
C 4.395984 -1.833776 0.540633
H 9.807289 -2.126514 0.454303
C 8.721973 -2.068618 0.467038
C 8.075263 -0.892757 0.083775
H 8.655627 -0.029521 -0.232474
C 6.681010 -0.821758 0.096615
H 6.186489 0.096625 -0.213731
C 5.906859 -1.921318 0.491403
C 6.570263 -3.097480 0.869644
H 5.986256 -3.965487 1.169946
C 7.963217 -3.173191 0.859884
H 8.456419 -4.096645 1.153444
H 4.049351 -1.044346 -0.138532
H 3.963553 -2.770594 0.168511
C -1.526670 3.137911 2.315963
H -2.016557 3.884300 2.965094
H -1.238808 2.305590 2.977065
N -2.514243 2.731434 1.329864
S -3.645633 1.616164 1.919466
O -4.278896 2.115221 3.147836
O -2.953195 0.305712 1.960296
C -4.847180 1.607343 0.604183
C -6.158160 1.979841 0.896991
H -6.421918 2.284997 1.903767
C -7.109002 1.950023 -0.122898
H -8.134412 2.235950 0.096936
C -6.763867 1.560157 -1.422892
C -5.434002 1.188875 -1.681808
H -5.149366 0.879189 -2.684303
C -4.467458 1.205622 -0.681227
H -3.443769 0.912803 -0.896628
C -7.789042 1.554696 -2.531275
H -8.807494 1.626364 -2.137643
H -7.635939 2.402648 -3.211321
H -7.719681 0.642634 -3.134584
C -0.305241 3.743944 1.618821
H -0.646751 4.543801 0.949306
H 0.323858 4.217247 2.385151
C 3.861107 -1.545432 1.960413
C 2.344756 -1.301724 2.028424
H 4.379289 -0.660481 2.353634
H 4.137295 -2.376629 2.625164
H 2.083642 -1.001063 3.054249
H 2.082936 -0.454745 1.382310
C 0.509608 2.707932 0.834739
H 0.859140 1.926879 1.525900
H -0.142702 2.200042 0.112232
C 1.712774 3.318139 0.106734
H 1.366725 4.069751 -0.616477
H 2.350313 3.855774 0.823500
C 2.561912 2.261456 -0.628696
H 2.902841 1.515314 0.101423
H 1.925918 1.724612 -1.343538
C 3.758083 2.852328 -1.343711
C 4.962843 3.077947 -0.661690
H 5.041723 2.798109 0.387284
C 6.058827 3.653057 -1.306845
H 6.983851 3.815892 -0.759260
C 5.968469 4.014830 -2.652381
H 6.821436 4.459484 -3.158277
C 4.776135 3.795317 -3.344169
H 4.697270 4.067590 -4.393723
C 3.683587 3.219136 -2.694018
H 2.760272 3.044077 -3.242250
C 1.485338 -2.506379 1.627517
H 1.723776 -3.365478 2.271949
H 1.709915 -2.807085 0.599962
C -0.022595 -2.253835 1.747682
H -0.576927 -3.178151 1.526538
H -0.273970 -1.974312 2.777025
N -0.506601 -1.132158 0.916684
H -1.273975 -0.594018 1.323384
S -0.780488 -1.347250 -0.715031
O -1.413105 -0.098274 -1.166362
O 0.460381 -1.822392 -1.334332
C -1.987633 -2.674978 -0.877972
C -3.247258 -2.549835 -0.281739
H -3.497810 -1.668857 0.301788
C -4.173683 -3.576920 -0.433786
H -5.151176 -3.484249 0.033776
C -3.869766 -4.729063 -1.177148
C -2.600738 -4.829258 -1.759279
H -2.343325 -5.716145 -2.333181
C -1.656642 -3.811714 -1.613866
H -0.672375 -3.889302 -2.062775
C -4.897104 -5.821866 -1.353712
H -5.654990 -5.534649 -2.094366
H -4.437148 -6.753139 -1.698320
H -5.425519 -6.029872 -0.416625

89
-2603.86330545790000
fullTos_Nrad_PhC6.bimol.conf05_orc_fwd
C 2.444636 0.042291 -2.833734
H 7.246771 -2.003633 -1.364918
C 6.285498 -1.587055 -1.655386
C 6.212840 -0.614683 -2.653044
H 7.118832 -0.266676 -3.143582
C 4.974914 -0.085936 -3.025832
H 4.926884 0.671406 -3.806084
C 3.790317 -0.512209 -2.411923
C 3.877363 -1.489621 -1.407727
H 2.971928 -1.838634 -0.916969
C 5.111582 -2.022800 -1.035465
H 5.155118 -2.785678 -0.261530
H 1.881480 0.346185 -1.943553
H 2.597654 0.949950 -3.434632
C 0.844712 3.851651 0.072836
H 0.372882 4.363562 0.919014
H 1.914807 4.122280 0.067140
N 0.809215 2.408794 0.192062
S 0.214091 1.787949 1.678333
O -0.577993 0.603869 1.295094
O -0.411422 2.829449 2.509961
C 1.739351 1.273906 2.434465
C 2.359726 2.129514 3.350506
H 1.888899 3.068130 3.622924
C 3.570169 1.741067 3.917634
H 4.056291 2.394745 4.637123
C 4.170045 0.518950 3.578770
C 3.517928 -0.319496 2.660239
H 3.963673 -1.272957 2.390242
C 2.307031 0.043297 2.080962
H 1.806832 -0.630230 1.392635
C 5.487409 0.103975 4.186184
H 6.248314 -0.042636 3.410061
H 5.861874 0.853786 4.889212
H 5.391372 -0.846718 4.724420

C 0.240595 4.304570 -1.276206
H 0.417821 5.385928 -1.354633
H 0.804890 3.826876 -2.085463
C 1.611080 -0.967930 -3.647402
C 0.269156 -0.418289 -4.161214
H 1.441050 -1.866286 -3.044826
H 2.203330 -1.287887 -4.515756
H 0.464689 0.447571 -4.811299
H -0.188162 -1.181056 -4.807975
C -1.256065 4.000278 -1.443757
H -1.557735 4.332472 -2.447093
H -1.406729 2.913783 -1.425704
C -2.168668 4.658821 -0.400140
H -1.914359 4.296303 0.603204
H -1.990749 5.743943 -0.388387
C -3.671195 4.417066 -0.656296
H -4.240202 4.947200 0.120477
H -3.953004 4.881212 -1.610503
C -4.086695 2.956813 -0.676844
C -4.744311 2.415712 -1.789352
H -4.954249 3.053732 -2.645381
C -5.136506 1.075452 -1.815910
H -5.649701 0.680535 -2.689271
C -4.867713 0.247046 -0.725299
H -5.167408 -0.797624 -0.740625
C -4.207433 0.771463 0.389122
H -3.979848 0.131898 1.237104
C -3.826325 2.112949 0.413990
H -3.310632 2.500628 1.289107
C -0.775056 0.005952 -3.111297
H -1.653091 0.395955 -3.643624
H -0.399009 0.839380 -2.503780
C -1.268252 -1.107551 -2.172392
H -2.268265 -0.859247 -1.792254
H -1.337629 -2.054353 -2.716322
N -0.331074 -1.307579 -1.052331
H -0.364747 -0.575310 -0.337952
S -0.166834 -2.806801 -0.344661
O -0.034527 -3.786524 -1.427762
O 0.888875 -2.637069 0.666696
C -1.689415 -3.187879 0.535592
C -2.571576 -4.128826 0.006089
H -2.317501 -4.643044 -0.914551
C -3.761439 -4.403598 0.681839
H -4.448506 -5.140252 0.272379
C -4.082080 -3.752660 1.879253
C -3.176060 -2.807758 2.388292
H -3.406353 -2.292969 3.318312
C -1.983884 -2.520955 1.729301
H -1.294085 -1.786098 2.130930
C -5.356451 -4.073477 2.623640
H -6.080529 -4.581810 1.979332
H -5.157597 -4.732157 3.479256
H -5.829244 -3.167179 3.018059

89
-2603.86232625260000
fullTos_Nrad_PhC6.bimol.conf52_orc_rev
C 1.469800 -3.302417 -0.153156
H 6.552558 -5.070846 -0.809018
C 5.532493 -4.717891 -0.682321
C 5.133569 -3.503278 -1.242319
H 5.841739 -2.904204 -1.809213
C 3.819972 -3.056265 -1.080646
H 3.513392 -2.114199 -1.530421
C 2.882306 -3.807882 -0.360015
C 3.296972 -5.029520 0.190111
H 2.582122 -5.635351 0.743537
C 4.607464 -5.481402 0.033281
H 4.905514 -6.433414 0.465517
H 1.245683 -2.524055 -0.894037
H 0.757877 -4.116534 -0.336767
C 1.345749 2.139950 2.528204
H 1.079922 2.578553 3.504752
H 1.004226 1.092534 2.567560
N 0.615290 2.858035 1.495964
S -1.072642 2.715353 1.667301
O -1.445882 3.421847 2.902312
O -1.431314 1.292700 1.501163
C -1.670385 3.630714 0.261886
C -1.661002 5.028086 0.315752
H -1.302315 5.540100 1.202505
C -2.134952 5.744879 -0.777372
H -2.133701 6.831597 -0.742911
C -2.624981 5.088713 -1.918736
C -2.635761 3.688710 -1.932379
H -3.030123 3.159186 -2.795453
C -2.158701 2.947567 -0.850996
H -2.195830 1.864065 -0.875841
C -3.115192 5.880577 -3.107079
H -2.274451 6.211095 -3.731274
H -3.778449 5.283348 -3.740124
H -3.658075 6.779056 -2.794104
C 2.854153 2.219324 2.283791
H 3.151744 3.274972 2.242514
H 3.359166 1.782556 3.156250
C 1.242583 -2.729274 1.262351
C -0.125001 -2.056287 1.463959
H 2.033048 -1.995226 1.471000
H 1.374023 -3.532609 2.001283
H -0.153732 -1.618292 2.472866
H -0.214600 -1.213591 0.765780
C 3.300803 1.497277 1.007176
H 2.975996 0.446807 1.053749
H 2.782656 1.941063 0.146227
C 4.816575 1.547493 0.783608
H 5.152115 2.593201 0.742713
H 5.333024 1.094405 1.641374
C 5.254015 0.823390 -0.505645
H 4.895241 -0.213176 -0.467521
H 4.756877 1.296425 -1.363483
C 6.753435 0.831596 -0.714766
C 7.389650 1.924066 -1.320549
H 6.790599 2.761157 -1.673958
C 8.775221 1.949631 -1.482281
H 9.247874 2.805014 -1.958531
C 9.552266 0.877118 -1.039834
H 10.631366 0.893570 -1.168073
C 8.932081 -0.218788 -0.437742
H 9.527295 -1.061583 -0.095216
C 7.545386 -0.239250 -0.277891
H 7.067816 -1.100509 0.184857
C -1.326377 -2.994876 1.290840
H -1.212450 -3.860444 1.961042
H -1.365594 -3.383519 0.268920
C -2.680375 -2.342867 1.614899
H -3.482897 -3.082653 1.519130
H -2.695767 -1.997278 2.655818
N -3.056355 -1.175415 0.804995
H -2.500397 -0.327835 0.920315
S -3.593750 -1.348471 -0.760317
O -3.408039 -0.026488 -1.380085
O -3.037154 -2.550006 -1.398529
C -5.352904 -1.645738 -0.558741
C -6.140984 -0.707408 0.115581
H -5.683211 0.174797 0.551347
C -7.509239 -0.924752 0.226469
H -8.123835 -0.197960 0.752694
C -8.111871 -2.066771 -0.328941
C -7.300591 -2.986614 -1.001392
H -7.748149 -3.875595 -1.438858
C -5.924052 -2.783702 -1.122793
H -5.294317 -3.494732 -1.646300
C -9.599831 -2.287370 -0.194298
H -10.165989 -1.448692 -0.617483
H -9.917975 -3.199838 -0.707404
H -9.894947 -2.374669 0.858786

89

-2603.86168660970000
fullTos_Nrad_PhC6.bimol.conf54_orc_rev
C 1.468839 -3.301608 -0.152845
H 6.551173 -5.070972 -0.809499
C 5.531193 -4.717831 -0.682643
C 5.132529 -3.502931 -1.242207
H 5.840822 -2.903830 -1.808920
C 3.819045 -3.055665 -1.080325
H 3.512660 -2.113378 -1.529768
C 2.881231 -3.807306 -0.359910
C 3.295630 -5.029238 0.189765
H 2.580666 -5.635104 0.743003
C 4.606011 -5.481373 0.032725
H 4.903850 -6.433608 0.464616
H 1.245099 -2.522447 -0.893008
H 0.756700 -4.115326 -0.337397
C 1.346649 2.139010 2.527893
H 1.080922 2.577487 3.504528
H 1.005059 1.091613 2.567128
N 0.616152 2.857308 1.495830
S -1.071805 2.715048 1.667511
O -1.444579 3.421819 2.902505
O -1.430865 1.292452 1.501673
C -1.669683 3.630364 0.262127
C -1.660670 5.027679 0.316119
H -1.302233 5.539712 1.202960
C -2.134804 5.744469 -0.777003
H -2.133920 6.831173 -0.742430
C -2.624566 5.088313 -1.918406
C -2.635060 3.688242 -1.932149
H -3.029408 3.158668 -2.795210
C -2.157841 2.947173 -0.850869
H -2.194809 1.863670 -0.875762
C -3.114426 5.879796 -3.107144
H -2.274718 6.194849 -3.740701
H -3.791083 5.287820 -3.730943
H -3.642286 6.787395 -2.795016
C 2.855050 2.218346 2.283460
H 3.152711 3.273986 2.242491
H 3.360027 1.781298 3.155802
C 1.241479 -2.729714 1.263150
C -0.125973 -2.056546 1.465051
H 2.032088 -1.996055 1.472622
H 1.372554 -3.533742 2.001390
H -0.154694 -1.619105 2.474197
H -0.215317 -1.213440 0.767336
C 3.301706 1.496643 1.006658
H 2.976779 0.446194 1.052866
H 2.783707 1.940761 0.145794
C 4.817516 1.546758 0.783287
H 5.153192 2.592439 0.742808
H 5.333794 1.093299 1.640960
C 5.255032 0.823065 -0.506168
H 4.896059 -0.213447 -0.468476
H 4.758126 1.296509 -1.363911
C 6.754486 0.831068 -0.715032
C 7.546258 -0.239660 -0.277522
H 7.068513 -1.100674 0.185505
C 8.932981 -0.219393 -0.437101
H 9.528054 -1.062091 -0.094089
C 9.553385 0.876196 -1.039555
H 10.632512 0.892493 -1.167586
C 8.776527 1.948579 -1.482631
H 9.249349 2.803710 -1.959164
C 7.390917 1.923208 -1.321167
H 6.792011 2.760199 -1.675059
C -1.327557 -2.994751 1.291336
H -1.214116 -3.860473 1.961420
H -1.366560 -3.383216 0.269337
C -2.681469 -2.342380 1.615011
H -3.484150 -3.081978 1.519139
H -2.697035 -1.996623 2.655871
N -3.056931 -1.174988 0.804780
H -2.500837 -0.327496 0.920045
S -3.594235 -1.348204 -0.760510
O -3.407966 -0.026433 -1.380568
O -3.037995 -2.550075 -1.398404
C -5.353498 -1.644879 -0.558981
C -6.141206 -0.706516 0.115688
H -5.683104 0.175379 0.551737
C -7.509641 -0.923132 0.226065
H -8.124041 -0.196068 0.752134
C -8.112729 -2.064597 -0.329928
C -7.301858 -2.984284 -1.003137
H -7.749886 -3.872467 -1.441751
C -5.925192 -2.782102 -1.124026
H -5.295805 -3.492916 -1.648245
C -9.600248 -2.286483 -0.192583
H -9.886731 -2.416968 0.858583
H -10.167535 -1.429667 -0.575564
H -9.925105 -3.176554 -0.739724

89
-2603.84539723030000 fullTos_Nrad_PhC6.bimol.conf82
C 0.439546 2.923153 -0.179756
H -3.875523 3.660066 -3.317871
C -3.009233 3.514521 -2.678274
C -2.745801 4.411325 -1.637907
H -3.408017 5.256353 -1.468438
C -1.632881 4.229678 -0.820783
H -1.435115 4.937615 -0.020646
C -0.754286 3.146031 -1.025020
C -1.035511 2.251988 -2.079311
H -0.354080 1.424254 -2.260119
C -2.146990 2.435703 -2.897210
H -2.338158 1.743383 -3.712716
H 0.119153 2.281790 0.840193
H 1.124338 2.206724 -0.645274
C -1.401017 2.127520 2.751908
H -1.256135 2.222075 3.837282
H -1.601160 3.140291 2.379007
N -0.082430 1.784903 2.205996
S 0.657460 0.338841 2.557498
O 0.525553 0.150010 4.011442
O 1.996243 0.425040 1.944024
C -0.138070 -1.072437 1.768607
C -0.883946 -1.957711 2.550250
H -0.967217 -1.790512 3.618809
C -1.490702 -3.056019 1.944551
H -2.065275 -3.750019 2.553501
C -1.366335 -3.285137 0.566207
C -0.593064 -2.392084 -0.189236
H -0.459189 -2.566887 -1.253931
C 0.026523 -1.288921 0.398055
H 0.653290 -0.632834 -0.198688
C -2.061045 -4.457613 -0.084057
H -2.028320 -5.347439 0.554077
H -3.119593 -4.230973 -0.268474
H -1.604825 -4.711338 -1.046128
C -2.615827 1.225580 2.483894
H -2.429664 0.228681 2.898578
H -3.449405 1.640944 3.068728
C 1.164648 4.139710 0.406826
C 2.586110 3.796197 0.881448
H 0.587933 4.550787 1.245829
H 1.205930 4.934245 -0.353434
H 2.950514 4.589714 1.546044
H 2.527222 2.891708 1.496824
C -3.035085 1.111326 1.014192
H -3.316598 2.103690 0.635765
H -2.178089 0.788272 0.410338
C -4.192712 0.128049 0.800548
H -3.887968 -0.872985 1.133976
H -5.048003 0.413458 1.429787
C -4.649347 0.051618 -0.670443
H -4.997322 1.044583 -0.986062
H -3.781406 -0.182950 -1.299556
C -5.737055 -0.974824 -0.905462
C -5.439072 -2.228021 -1.457329
H -4.414676 -2.447852 -1.751597

C -6.434767 -3.188772 -1.645726
H -6.181072 -4.151977 -2.081596
C -7.753286 -2.910319 -1.283313
H -8.531219 -3.654620 -1.431248
C -8.066622 -1.664211 -0.736015
H -9.091995 -1.434422 -0.457274
C -7.067525 -0.708243 -0.551536
H -7.322903 0.262174 -0.130331
C 3.586141 3.607669 -0.280198
H 4.054205 4.571858 -0.521438
H 3.065642 3.293304 -1.193241
C 4.697653 2.587121 0.005137
H 5.460143 2.625570 -0.775851
H 5.191892 2.809660 0.958529
N 4.249943 1.184911 0.102958
H 3.535676 1.010472 0.814656
S 3.845559 0.381821 -1.314474
O 2.376058 0.271999 -1.446346
O 4.621849 0.988697 -2.402796
C 4.469169 -1.272698 -1.018170
C 3.917881 -2.058459 -0.001250
H 3.128660 -1.668224 0.633905
C 4.407642 -3.345545 0.194203
H 3.986181 -3.958566 0.987498
C 5.431842 -3.867600 -0.613086
C 5.956868 -3.059159 -1.627148
H 6.747350 -3.446319 -2.265401
C 5.484929 -1.761825 -1.835088
H 5.893096 -1.133349 -2.619176
C 5.956419 -5.264096 -0.376485
H 5.140318 -5.994562 -0.326917
H 6.640338 -5.575644 -1.171863
H 6.501380 -5.326436 0.574149

89
-2603.84501672060000 fullTos_Nrad_PhC6.bimol.conf52
C -0.440384 -2.923805 -0.180475
H 3.874394 -3.661380 -3.318838
C 3.008160 -3.515709 -2.679194
C 2.744422 -4.412710 -1.639074
H 3.406345 -5.258016 -1.469845
C 1.631568 -4.230905 -0.821894
H 1.433554 -4.939005 -0.021963
C 0.753357 -3.146888 -1.025818
C 1.034889 -2.252645 -2.079858
H 0.353757 -1.424608 -2.260408
C 2.146292 -2.436528 -2.897822
H 2.337700 -1.744044 -3.713131
H -0.119652 -2.282899 0.839670
H -1.124891 -2.206949 -0.645749
C 1.401308 -2.129791 2.750741
H 1.256865 -2.225141 3.836104
H 1.601181 -3.142302 2.376992
N 0.082553 -1.786607 2.205592
S -0.656945 -0.340607 2.558153
O -0.524685 -0.152749 4.012191
O -1.995884 -0.426174 1.944926
C 0.138575 1.071128 1.770055
C 0.884690 1.955785 2.552159
H 0.968213 1.787804 3.620576
C 1.491305 3.054544 1.947125
H 2.066046 3.748077 2.556446
C 1.366567 3.284714 0.568996
C 0.593081 2.392234 -0.186918
H 0.458922 2.567839 -1.251447
C -0.026363 1.288641 0.399701
H -0.653243 0.632986 -0.197396
C 2.061122 4.457650 -0.080598
H 3.119209 4.230489 -0.267028
H 1.603584 4.713141 -1.041577
H 2.030078 5.346538 0.558920
C 2.616090 -1.227770 2.482879
H 2.430232 -0.231206 2.898509
H 3.449932 -1.643706 3.066926
C -1.165980 -4.140275 0.405680
C -2.587184 -3.796286 0.880750
H -0.589299 -4.552060 1.244358
H -1.207816 -4.934404 -0.354975
H -2.951822 -4.589875 1.545129
H -2.527741 -2.892057 1.496452
C 3.034568 -1.112282 1.013051
H 3.315980 -2.104303 0.633657
H 2.177222 -0.788807 0.409924
C 4.191955 -0.128676 0.799620
H 3.887220 0.872038 1.134016
H 5.047603 -0.414465 1.428199
C 4.647812 -0.050946 -0.671541
H 4.995752 -1.043596 -0.988195
H 3.779507 0.184047 -1.299993
C 5.735235 0.975869 -0.906269
C 5.436916 2.229122 -1.457824
H 4.412468 2.448737 -1.752070
C 6.432346 3.190207 -1.645947
H 6.178387 4.153452 -2.081575
C 7.750931 2.912033 -1.283575
H 8.528659 3.656591 -1.431293
C 8.064605 1.665866 -0.736598
H 9.090036 1.436292 -0.457897
C 7.065775 0.709570 -0.552387
H 7.321423 -0.260882 -0.131428
C -3.587363 -3.606828 -0.280633
H -4.056145 -4.570636 -0.521995
H -3.066838 -3.292623 -1.193717
C -4.698076 -2.585520 0.005121
H -5.460776 -2.623293 -0.775692
H -5.192242 -2.807807 0.958608
N -4.249230 -1.183676 0.103037
H -3.534700 -1.009918 0.814639
S -3.844520 -0.380708 -1.314335
O -2.374955 -0.271415 -1.446018
O -4.620914 -0.987259 -2.402763
C -4.467522 1.274075 -1.018156
C -3.916603 2.059391 -0.000789
H -3.128002 1.668739 0.634879
C -4.405569 3.346930 0.194181
H -3.984129 3.959776 0.987606
C -5.428722 3.869653 -0.613868
C -5.953250 3.061669 -1.628659
H -6.742571 3.449565 -2.267919
C -5.482133 1.764038 -1.836115
H -5.889764 1.135981 -2.620819
C -5.954646 5.265482 -0.376359
H -5.137740 5.991407 -0.290108
H -6.611140 5.590414 -1.189345
H -6.530727 5.317649 0.556499

89
-2603.84257418090000 fullTos_Nrad_PhC6.bimol.conf54
C -0.439883 -2.923366 -0.181027
H 3.875946 -3.660784 -3.318000
C 3.009501 -3.515141 -2.678636
C 2.745494 -4.412104 -1.638555
H 3.407416 -5.257361 -1.469077
C 1.632377 -4.230328 -0.821726
H 1.434168 -4.938400 -0.021820
C 0.754150 -3.146387 -1.025976
C 1.035960 -2.252189 -2.079978
H 0.354834 -1.424206 -2.260802
C 2.147632 -2.436027 -2.897585
H 2.339241 -1.743578 -3.712878
H -0.119623 -2.282271 0.839140
H -1.124414 -2.206728 -0.646624
C 1.400048 -2.128978 2.751329
H 1.254755 -2.223828 3.836619
H 1.599948 -3.141706 2.378177
N 0.081764 -1.785710 2.205088
S -0.657540 -0.339344 2.556610
O -0.525361 -0.150479 4.010526
O -1.996426 -0.425064 1.943300
C 0.138419 1.071575 1.767517

C 0.884538 1.956725 2.549063
H 0.967756 1.789618 3.617641
C 1.491578 3.054820 1.943257
H 2.066317 3.748739 2.552140
C 1.367286 3.283824 0.564893
C 0.593781 2.390888 -0.190461
H 0.459955 2.565625 -1.255175
C -0.026121 1.287965 0.396939
H -0.653144 0.632035 -0.199707
C 2.062322 4.456046 -0.085474
H 3.120576 4.228732 -0.270768
H 1.605563 4.710390 -1.047128
H 2.030675 5.345674 0.552980
C 2.615292 -1.227417 2.484007
H 2.429291 -0.230508 2.898741
H 3.448444 -1.643162 3.069177
C -1.165451 -4.139843 0.405129
C -2.586686 -3.795834 0.880077
H -0.588790 -4.551586 1.243840
H -1.207228 -4.934003 -0.355500
H -2.951293 -4.589230 1.544709
H -2.527338 -2.891382 1.495456
C 3.035281 -1.113180 1.014507
H 3.317284 -2.105514 0.636358
H 2.178512 -0.790469 0.410149
C 4.192750 -0.129619 0.801315
H 3.887614 0.871362 1.134534
H 5.047817 -0.414790 1.430964
C 4.650007 -0.053232 -0.669486
H 4.999158 -1.045959 -0.984577
H 3.782125 0.180182 -1.299104
C 5.736761 0.974241 -0.904447
C 7.067027 0.709932 -0.548033
H 7.322995 -0.259455 -0.124823
C 8.065199 1.666849 -0.732573
H 9.090427 1.438821 -0.451862
C 7.751143 2.911650 -1.282436
H 8.528367 3.656683 -1.430410
C 6.432840 3.187827 -1.647348
H 6.178602 4.149975 -2.085233
C 5.438060 2.226133 -1.458872
H 4.413843 2.444188 -1.755070
C -3.586820 -3.606884 -0.281377
H -4.054532 -4.571089 -0.523241
H -3.066453 -3.291717 -1.194218
C -4.698654 -2.586897 0.004704
H -5.461262 -2.625179 -0.776177
H -5.192664 -2.810120 0.958054
N -4.251461 -1.184554 0.103209
H -3.537103 -1.010324 0.814868
S -3.847158 -0.380888 -1.313980
O -2.377663 -0.271837 -1.446571
O -4.624357 -0.986714 -2.402235
C -4.469345 1.273943 -1.016595
C -3.916186 2.059160 -0.000149
H -3.126990 1.667908 0.634406
C -4.406542 3.345529 0.197847
H -3.985599 3.957033 0.992610
C -5.431978 3.868242 -0.607628
C -5.960730 3.059440 -1.619324
H -6.755361 3.445421 -2.253099
C -5.488179 1.762633 -1.829791
H -5.900065 1.133459 -2.611368
C -5.941291 5.272229 -0.382235
H -6.260510 5.418159 0.656674
H -5.160137 6.015701 -0.585403
H -6.793075 5.498612 -1.030716

N-Cl, TS-1,5'-HAT$_{bimol}$

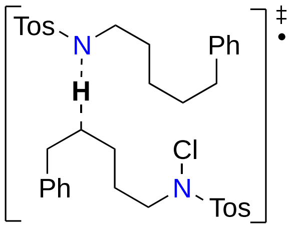

89

C -0.247597 -0.667291 3.365981
H -5.668607 -0.740317 3.331154
C -4.581570 -0.732879 3.322431
C -3.868371 -1.899202 3.601785
H -4.396865 -2.823174 3.822428
C -2.471150 -1.881323 3.605202
H -1.922706 -2.793161 3.833262
C -1.765135 -0.702810 3.334505
C -2.492146 0.462879 3.046324
H -1.957630 1.386097 2.836661
C -3.887347 0.447264 3.040179
H -4.433627 1.362203 2.825048
H 0.068914 0.073345 4.125680
H 0.142426 -1.629274 3.732623
C -0.355460 -3.902005 0.441682
H -1.053132 -4.208561 1.232622
H -0.645952 -4.437274 -0.468082
N -0.537144 -2.474164 0.193311
S -0.843666 -1.803190 -1.278460
O -0.168202 -2.555822 -2.343264
O -0.590810 -0.363387 -1.110001
C -2.605474 -2.045300 -1.504658
C -3.069470 -2.664726 -2.662067
H -2.359780 -3.007199 -3.407319
C -4.443337 -2.837508 -2.835554
H -4.809785 -3.322493 -3.737117
C -5.355794 -2.399610 -1.869090
H -5.549767 -1.422689 0.048907
C -3.493848 -1.590284 -0.524996
H -3.125197 -1.110720 0.376251
C -6.839415 -2.616976 -2.046567
H -7.090133 -2.841747 -3.087937
H -7.191798 -3.457464 -1.434136
H -7.413004 -1.734497 -1.741727
C 1.080461 -4.281196 0.831889
H 1.083446 -5.351872 1.083842
H 1.355504 -3.751826 1.756272
C 0.390919 -0.320098 2.051347
C 1.876944 -0.361513 1.877927
H -0.177622 0.294949 1.362579
H -0.220617 -1.791245 0.891897
H 2.284891 -1.222704 2.425168
C 2.602437 0.922422 2.386729
H 2.123293 -0.510383 0.819123
C 3.014009 1.915254 1.293029
N 1.903476 2.362472 0.393189
S 0.816714 3.534631 1.152736
O 1.530298 4.749720 1.559446
C -0.292271 3.919324 -0.192925
C -0.527746 5.262491 -0.483095
H 0.009150 6.035338 0.055927
C -1.449588 5.582729 -1.478893
H -1.638283 6.627457 -1.712537
C -2.127898 4.582516 -2.186918
C -1.858992 3.240124 -1.874521
H -2.361263 2.446578 -2.421868
C -0.952980 2.894398 -0.876748
H -0.745887 1.849837 -0.670710
C -3.133600 4.933430 -3.257254
H -2.976123 4.336412 -4.162603
H -4.157671 4.735278 -2.915195
H -3.075199 5.990840 -3.532681
H 3.550513 0.634724 2.860599
H 2.005927 1.418322 3.155924
H 3.713516 1.412910 0.620807
H 3.525681 2.782509 1.731101
O 0.115536 2.737746 2.169089
C -4.859083 -1.771097 -0.715138
C 2.114883 -3.993255 -0.260083
H 1.794264 -4.470836 -1.196301
H 2.129357 -2.917793 -0.465846
C 3.525909 -4.473224 0.095123
H 3.839514 -4.039824 1.055725
H 3.513166 -5.563315 0.237211
C 4.584589 -4.132008 -0.979802
H 4.223925 -4.484716 -1.955113
H 5.498481 -4.698983 -0.761923
C 4.928222 -2.658404 -1.062245
C 4.218618 -1.783531 -1.898247
H 3.413534 -2.168625 -2.519462
C 4.530098 -0.422980 -1.943170
H 3.964785 0.237685 -2.594574
C 5.565409 0.087071 -1.156172
H 5.817344 1.143378 -1.202783
C 6.281449 -0.772776 -0.319711
H 7.094901 -0.388376 0.290822
C 5.962361 -2.130220 -0.275610
H 6.529831 -2.795232 0.372755
Cl 2.656656 3.140321 -1.035605

89

C -0.250899 -0.722902 3.240919
H -5.666698 -0.719973 3.375150
C -4.580109 -0.723382 3.343697
C -3.873292 -1.896089 3.612994
H -4.406353 -2.813148 3.850512
C -2.477116 -1.893422 3.582276
H -1.932592 -2.810344 3.798372
C -1.765853 -0.725596 3.280974
C -2.485511 0.449823 3.015577
H -1.946826 1.366820 2.789702
C -3.880577 0.449462 3.045343
H -4.422194 1.369919 2.842770
H 0.120063 0.088599 3.888896
H 0.143489 -1.653491 3.670800
C -0.345903 -3.791485 0.537359
H -1.007850 -4.063541 1.370560
H -0.704753 -4.338382 -0.344353
N -0.514186 -2.366580 0.265947
S -0.817971 -1.752231 -1.220966
O -0.139119 -2.519132 -2.278812
O -0.591966 -0.300338 -1.121980
C -2.582112 -2.031021 -1.453582
C -3.030905 -2.655556 -2.616024
H -2.311138 -2.994852 -3.352957
C -4.402058 -2.834862 -2.808814
H -4.752939 -3.322624 -3.715220
C -5.330821 -2.399409 -1.856826
H -5.552891 -1.424424 0.058687
C -3.488482 -1.582631 -0.487790
H -3.134183 -1.103697 0.419118
C -6.812464 -2.615635 -2.055759
H -7.049825 -2.829894 -3.102359
H -7.173771 -3.462142 -1.456617

H -7.389471 -1.735949 -1.749360
C 1.090496 -4.234266 0.859210
H 1.054180 -5.306903 1.100581
H 1.422388 -3.729121 1.778428
C 0.344190 -0.497589 1.857935
C 1.853823 -0.458154 1.737818
H -0.157696 0.282919 1.286244
H -0.065534 -1.502845 1.158875
H 2.280326 -1.333815 2.243268
C 2.499946 0.829067 2.333170
H 2.127715 -0.537747 0.680013
C 2.966041 1.857686 1.293600
N 1.886590 2.351309 0.381282
S 0.806951 3.522517 1.147267
O 1.520941 4.731942 1.566824
C -0.297018 3.915633 -0.197582
C -0.529499 5.259796 -0.486652
H 0.008036 6.031544 0.053261
C -1.449254 5.581527 -1.483491
H -1.635377 6.626543 -1.717794
C -2.128244 4.582215 -2.192711
C -1.861253 3.239447 -1.881680
H -2.361742 2.446761 -2.431760
C -0.957711 2.891766 -0.882753
H -0.752457 1.846339 -0.679115
C -3.139493 4.934830 -3.256954
H -2.984200 4.341016 -4.164412
H -4.162322 4.733138 -2.911789
H -3.085285 5.993491 -3.527811
H 3.415178 0.548356 2.870492
H 1.842447 1.298745 3.066871
H 3.681728 1.371930 0.627002
H 3.473243 2.700203 1.782615
O 0.103741 2.715198 2.155783
C -4.850767 -1.769890 -0.696474
C 2.105203 -3.976447 -0.258126
H 1.768063 -4.463981 -1.182955
H 2.123266 -2.904332 -0.479049
C 3.516337 -4.462868 0.091747
H 3.835399 -4.025880 1.049092
H 3.497011 -5.552031 0.240094
C 4.580463 -4.131643 -0.981031
H 4.227662 -4.489302 -1.957258
H 5.491487 -4.698790 -0.751522
C 4.928895 -2.659232 -1.064402
C 4.217260 -1.782614 -1.897250
H 3.410180 -2.166482 -2.516719
C 4.526767 -0.421641 -1.938190
H 3.960016 0.240238 -2.587133
C 5.561226 0.087902 -1.149089
H 5.810483 1.145041 -1.191399
C 6.279195 -0.773360 -0.315851
H 7.092329 -0.389559 0.295505
C 5.963105 -2.132056 -0.277235
H 6.531900 -2.798265 0.368793
Cl 2.666927 3.137587 -1.025787

89

C 0.259445 0.271355 3.450815
H -5.126791 0.862855 3.337133
C -4.046895 0.737023 3.348708
C -3.481389 -0.434003 3.856394
H -4.119021 -1.227942 4.237184
C -2.093027 -0.583854 3.885543
H -1.658692 -1.494802 4.292902
C -1.248389 0.428213 3.412572
C -1.827374 1.599209 2.901431
H -1.181197 2.394751 2.539414
C -3.214130 1.751886 2.868529
H -3.644929 2.670260 2.477462
H 0.697806 1.211862 3.829386
H 0.534382 -0.496310 4.190592
C -1.302182 -3.691730 0.872476
H -1.974835 -3.818479 1.730596
H -1.759111 -4.214950 0.026437
N -1.258014 -2.263459 0.539622
S -1.506196 -1.693389 -1.000457
O -1.038668 -2.652698 -2.010726
O -0.983443 -0.317694 -0.983498
C -3.293891 -1.629574 -1.143694
C -3.914352 -2.240695 -2.231430
H -3.315251 -2.782799 -2.954919
C -5.300466 -2.144151 -2.366444
H -5.787370 -2.621172 -3.213510
C -6.072237 -1.444348 -1.431878
H -5.998800 -0.282997 0.387485
C -4.038285 -0.918304 -0.197450
H -3.548178 -0.446525 0.648087
C -7.574183 -1.360992 -1.566590
H -7.904890 -1.661989 -2.565379
H -8.073025 -2.017304 -0.841508
H -7.936550 -0.343477 -1.381145
C 0.065215 -4.310595 1.193821
H -0.117234 -5.337825 1.542005
H 0.507293 -3.776995 2.048492
C 0.892993 -0.058780 2.127599
C 2.353667 -0.388120 2.067264
H 0.403341 0.288390 1.221751
H -0.628103 -1.664055 1.073497
H 2.595694 -1.077037 2.890023
C 3.311162 0.834768 2.201032
H 2.587937 -0.916912 1.134092
C 3.708235 1.514133 0.884067
N 2.564739 2.028862 0.065038
S 1.887326 3.538754 0.691453
O 2.872410 4.625199 0.681711
C 0.618539 3.876637 -0.517183
C 0.549959 5.159242 -1.059402
H 1.295277 5.900858 -0.793565
C -0.479645 5.455838 -1.951717
H -0.540306 6.452912 -2.380498
C -1.427736 4.489596 -2.312449
C -1.322689 3.205194 -1.755100
H -2.031431 2.429507 -2.032444
C -0.313581 2.890277 -0.851546
H -0.243622 1.884838 -0.454064
C -2.544720 4.816979 -3.274189
H -2.651411 4.039349 -4.038880
H -3.506959 4.887380 -2.750603
H -2.372669 5.771459 -3.780938
H 4.265002 0.484122 2.619318
H 2.906762 1.561708 2.908422
H 4.181694 0.766925 0.242649
H 4.437707 2.314397 1.067652
O 1.276419 3.131078 1.964606
C -5.418018 -0.833183 -0.349186
C 1.054961 -4.326830 0.023897
H 0.578203 -4.795106 -0.848005
H 1.281181 -3.298151 -0.277534
C 2.354262 -5.071269 0.353314
H 2.826896 -4.625256 1.240119
H 2.114564 -6.110421 0.620833
C 3.377849 -5.093221 -0.805966
H 2.875977 -5.457857 -1.712139
H 4.157636 -5.827018 -0.566117
C 4.033411 -3.756207 -1.087325
C 3.457576 -2.827229 -1.966923
H 2.526279 -3.071716 -2.472179
C 4.063542 -1.592022 -2.203979
H 3.597815 -0.885498 -2.885248
C 5.264984 -1.265767 -1.570431
H 5.744860 -0.310851 -1.768563
C 5.850070 -2.180831 -0.691949
H 6.789380 -1.941079 -0.199562
C 5.236672 -3.411432 -0.454481
H 5.703114 -4.122571 0.224695
Cl 3.177445 2.337147 -1.592214

89

C 0.262449 0.269981 3.451336
H -5.123836 0.861449 3.340195
C -4.043935 0.735615 3.351262
C -3.478183 -0.435331 3.858836
H -4.115621 -1.229207 4.240081
C -2.089802 -0.585173 3.887325
H -1.655265 -1.496040 4.294654
C -1.245396 0.426813 3.413773
C -1.824635 1.597734 2.902727
H -1.178638 2.393226 2.540285
C -3.211401 1.750408 2.870509
H -3.642398 2.668728 2.479531
H 0.700974 1.210393 3.829962
H 0.537703 -0.497859 4.190821
C -1.304042 -3.691611 0.871889
H -1.976732 -3.818488 1.729962
H -1.760944 -4.214627 0.025714
N -1.259775 -2.263320 0.539267
S -1.508017 -1.692553 -1.000484
O -1.041084 -2.651824 -2.011050
O -0.984700 -0.317084 -0.983093
C -3.295682 -1.627704 -1.143653
C -3.916553 -2.238719 -2.231110
H -3.317861 -2.781418 -2.954492
C -5.302732 -2.141582 -2.365899
H -5.790021 -2.618751 -3.212644
C -6.073976 -1.441170 -1.431492
H -5.999747 -0.279980 0.388062
C -4.039655 -0.915991 -0.197297
H -3.549212 -0.444561 0.648244
C -7.575729 -1.355098 -1.566700
H -7.909121 -1.683663 -2.555816
H -8.076721 -1.986822 -0.821660
H -7.933029 -0.330662 -1.411233
C 0.063328 -4.310566 1.193162
H -0.119193 -5.337790 1.541338
H 0.505481 -3.777009 2.047819
C 0.895417 -0.059887 2.127772
C 2.356104 -0.388966 2.066691
H 0.405200 0.287111 1.222159
H -0.630702 -1.663714 1.073838
H 2.598663 -1.077825 2.889343
C 3.313462 0.834089 2.199945
H 2.589972 -0.917770 1.133426
C 3.709768 1.513487 0.882781
N 2.565815 2.028319 0.064432
S 1.888694 3.538074 0.691423
O 2.873827 4.624477 0.681604
C 0.619495 3.876360 -0.516659
C 0.551174 5.158917 -1.059066
H 1.296898 5.900279 -0.793656
C -0.478616 5.455721 -1.951059
H -0.539020 6.452720 -2.380061
C -1.427213 4.489741 -2.311261
C -1.322372 3.205385 -1.753821
H -2.031396 2.429877 -2.030911
C -0.313048 2.890267 -0.850549
H -0.243226 1.884854 -0.452977
C -2.544839 4.817764 -3.272037
H -2.660437 4.034574 -4.029654
H -3.504637 4.900472 -2.745701
H -2.367052 5.766598 -3.787368
H 4.267571 0.483613 2.617756
H 2.909307 1.560979 2.907529
H 4.182810 0.766296 0.241038
H 4.439361 2.313732 1.065961
O 1.278182 3.130125 1.964678
C -5.419298 -0.830288 -0.348800
C 1.052998 -4.326890 0.023180
H 0.576117 -4.795105 -0.848692
H 1.279332 -3.298235 -0.278248
C 2.352199 -5.071542 0.352511
H 2.825088 -4.625469 1.239150
H 2.112316 -6.110591 0.620263
C 3.375586 -5.093970 -0.806937
H 2.873452 -5.458646 -1.712949
H 4.155212 -5.827938 -0.567086
C 4.031483 -3.757205 -1.088674
C 3.455685 -2.828148 -1.968207
H 2.524132 -3.072367 -2.473120
C 4.062028 -1.593201 -2.205665
H 3.596328 -0.886616 -2.886892
C 5.263809 -1.267289 -1.572588
H 5.743967 -0.312582 -1.771037
C 5.848852 -2.182427 -0.694151
H 6.788422 -1.942948 -0.202126
C 5.235081 -3.412762 -0.456281
H 5.701497 -4.123972 0.222837
Cl 3.177718 2.336842 -1.593084

89

C -0.239711 -0.670238 3.232659
H -5.655586 -0.667270 3.366941
C -4.568996 -0.670658 3.335529
C -3.862165 -1.843351 3.604823
H -4.395213 -2.760427 3.842305
C -2.465986 -1.840676 3.574141
H -1.921479 -2.757626 3.790159
C -1.754722 -0.672854 3.272839
C -2.474380 0.502557 3.007466
H -1.935701 1.419553 2.781563
C -3.869457 0.502183 3.037185
H -4.411067 1.422637 2.834585
H 0.131170 0.141160 3.880914
H 0.154783 -1.600771 3.662614
C -0.334995 -3.738957 0.529158
H -0.996579 -4.011548 1.362465
H -0.693715 -4.285905 -0.352487
N -0.503937 -2.313552 0.258512
S -0.806990 -1.699649 -1.229351
O -0.127875 -2.466379 -2.287156
O -0.580684 -0.247741 -1.130136
C -2.571023 -1.978259 -1.461922
C -3.019848 -2.602803 -2.624279
H -2.300107 -2.942171 -3.361207
C -4.391004 -2.782138 -2.816999
H -4.741923 -3.270014 -3.723326
C -5.319707 -2.346663 -1.865009
H -5.541718 -1.371740 0.050567
C -3.477337 -1.529887 -0.496040
H -3.122976 -1.051060 0.410891
C -6.801347 -2.562906 -2.063920
H -7.038704 -2.777205 -3.110505
H -7.162649 -3.409408 -1.464756
H -7.378366 -1.683226 -1.757531
C 1.101725 -4.181253 0.851067
H 1.065583 -5.253850 1.092672
H 1.433699 -3.675857 1.770128
C 0.355617 -0.444653 1.850029
C 1.865012 -0.405592 1.729845
H -0.147209 0.333648 1.276241
H -0.057211 -1.453457 1.149308
H 2.291583 -1.281085 2.235515
C 2.511189 0.881988 2.325165
H 2.139070 -0.485169 0.672081
C 2.977179 1.910549 1.285483
N 1.897691 2.404042 0.373112
S 0.818050 3.575276 1.139105
O 1.532049 4.784670 1.558639
C -0.285900 3.968381 -0.205757
C -0.518409 5.312556 -0.494814
H 0.019153 6.084305 0.045067
C -1.438171 5.634278 -1.491635
H -1.624236 6.679291 -1.725996
C -2.117135 4.634955 -2.200877

C -1.850171 3.292195 -1.889816
H -2.350594 2.499519 -2.439968
C -0.946614 2.944510 -0.890905
H -0.741332 1.899068 -0.687297
C -3.128379 4.987561 -3.265128
H -2.973015 4.393702 -4.172527
H -4.151278 4.785923 -2.920067
H -3.074140 6.046217 -3.535975
H 3.426416 0.601344 2.862525
H 1.853632 1.351646 3.058820
H 3.692852 1.424748 0.618931
H 3.484340 2.753149 1.774401
O 0.114843 2.767968 2.147617
C -4.839616 -1.717145 -0.704645
C 2.116427 -3.923634 -0.266304
H 1.779292 -4.411180 -1.191129
H 2.134551 -2.851533 -0.487299
C 3.527516 -4.410179 0.083553
H 3.846714 -3.973226 1.040868
H 3.508045 -5.499348 0.231833
C 4.591564 -4.078911 -0.989253
H 4.238818 -4.436593 -1.965487
H 5.502626 -4.645992 -0.759724
C 4.940000 -2.606495 -1.072556
C 4.228373 -1.729876 -1.905427
H 3.421274 -2.113756 -2.524870
C 4.537873 -0.368911 -1.946375
H 3.971128 0.292970 -2.595320
C 5.572345 0.140634 -1.157273
H 5.821603 1.197773 -1.199583
C 6.290309 -0.720619 -0.324026
H 7.103473 -0.336821 0.287292
C 5.974201 -2.079322 -0.285390
H 6.542990 -2.745529 0.360647
Cl 2.678032 3.190311 -1.033940

N-Cl, TS-1,6'-HAT$_{bimol}$

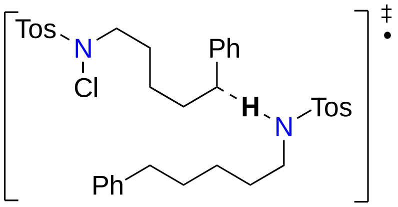

89

C 0.025917 2.880849 0.033006
H -4.608223 5.056875 -1.715411
C -3.681611 4.621130 -1.353088
C -3.046760 5.153043 -0.228799
H -3.481195 6.005072 0.287710
C -1.849553 4.601642 0.231364
H -1.359842 5.032548 1.100878
C -1.260876 3.512035 -0.427624
C -1.914730 2.985897 -1.551927
H -1.466657 2.145340 -2.076812
C -3.111764 3.529721 -2.012398
H -3.592958 3.111517 -2.892985
H -0.217135 2.107127 0.797888
H 0.453242 2.323435 -0.791567
C -1.983860 1.660053 3.051692
H -1.932246 1.629213 4.155173
H -2.212853 2.699910 2.785280
N -0.606117 1.472264 2.630869
S 0.203788 -0.016774 2.722201
O 0.015814 -0.459136 4.114028
O 1.543337 0.270052 2.196871
C -0.492813 -1.264519 1.634118
C -1.273298 -2.280740 2.191870
H -1.466245 -2.285934 3.259322
C -1.763596 -3.289785 1.368904
H -2.363233 -4.086528 1.802317
C -1.488237 -3.301289 -0.007533
C -0.684947 -2.280786 -0.535975
H -0.436320 -2.285942 -1.593983
C -0.176476 -1.263028 0.272240
H 0.476382 -0.505201 -0.149646
C -2.054466 -4.390032 -0.887055
H -1.915388 -5.379386 -0.436577
H -3.133875 -4.252308 -1.032647
H -1.582899 -4.396430 -1.874181
C -3.123425 0.738644 2.586694
H -2.905622 -0.296487 2.868051
H -4.006285 1.029532 3.172909
C 1.075845 3.808841 0.669653
C 2.500956 3.256224 0.510555
H 0.852661 3.939083 1.735978
H 1.018804 4.805955 0.210020
H 3.193547 3.840634 1.132227
H 2.536324 2.228550 0.884364
C -3.465750 0.825097 1.093652
H -3.738957 1.857724 0.839468
H -2.578924 0.588558 0.492500
C -4.605656 -0.127272 0.709223
H -4.313002 -1.160263 0.941257
H -5.490585 0.085562 1.325978
C -4.997043 -0.040241 -0.779798
H -5.318859 0.985672 -1.003472
H -4.105870 -0.224764 -1.393306
C -6.086818 -1.020107 -1.162555
C -7.429783 -0.759527 -0.853753
H -7.691952 0.174829 -0.361242
C -8.432553 -1.675107 -1.173723
H -9.467438 -1.450114 -0.928366
C -8.110261 -2.873713 -1.813994
H -8.890776 -3.586052 -2.067749
C -6.779015 -3.145407 -2.132147
H -6.517741 -4.071246 -2.638712
C -5.779876 -2.225380 -1.808119
H -4.745141 -2.438655 -2.069716
C 2.965564 3.311267 -0.951187
H 3.012791 4.360363 -1.278502
H 2.245140 2.818332 -1.605898
C 4.361597 2.741957 -1.210726
H 4.634327 2.824133 -2.270980
H 5.094214 3.309441 -0.631402
N 4.534195 1.333243 -0.730810
S 3.740442 0.124512 -1.750988
O 2.307524 0.396204 -1.564187
O 4.301649 0.102094 -3.105869
C 4.173549 -1.383285 -0.901351
C 3.698631 -1.602094 0.395050
H 3.104308 -0.854046 0.909955
C 4.009655 -2.806323 1.019621
H 3.648260 -2.984056 2.029372
C 4.773400 -3.790483 0.372352
C 5.221311 -3.541380 -0.931813
H 5.808509 -4.295034 -1.450379
C 4.929926 -2.340413 -1.576398
H 5.279941 -2.141426 -2.583397
C 5.120069 -5.078944 1.079053
H 5.441248 -5.851235 0.373299
H 5.938077 -4.924761 1.794848
H 4.266095 -5.466644 1.645206
Cl 6.290093 0.988026 -0.705371

89

C 0.074929 3.032214 -0.075403
H -4.598869 5.012939 -1.706451
C -3.667198 4.589458 -1.342299
C -3.019324 5.157080 -0.237057
H -3.458101 6.016609 0.263408
C -1.811552 4.642621 0.215115
H -1.310113 5.104867 1.060623
C -1.207620 3.525080 -0.429108
C -1.916131 2.930293 -1.512771
H -1.477335 2.069698 -2.011930
C -3.109094 3.466385 -1.970259
H -3.609828 3.017550 -2.824150
H -0.546579 1.772747 1.514118
H 0.409733 2.129741 -0.584510
C -1.983108 1.645555 3.076615
H -1.849520 1.538311 4.156504
H -2.190568 2.703563 2.877837
N -0.674864 1.396480 2.453538
S 0.206291 0.006916 2.695758
O -0.019673 -0.398029 4.085597
O 1.552038 0.304404 2.183672
C -0.447714 -1.292336 1.633714
C -1.244583 -2.293485 2.191766
H -1.436110 -2.292885 3.259610
C -1.759416 -3.292839 1.369528
H -2.371357 -4.078954 1.805850
C -1.491940 -3.309379 -0.007530
C -0.679176 -2.298889 -0.539600
H -0.438633 -2.305043 -1.599752
C -0.149762 -1.292616 0.268582
H 0.512281 -0.545839 -0.158741
C -2.069123 -4.394123 -0.885442
H -1.928890 -5.385221 -0.438934
H -3.149131 -4.254978 -1.025130
H -1.603084 -4.399917 -1.875492

C -3.162138 0.779199 2.609639
H -2.974414 -0.262537 2.896008
H -4.042005 1.095141 3.188637
C 1.088376 3.753782 0.768426
C 2.511480 3.216463 0.538822
H 0.851361 3.663327 1.840472
H 1.048657 4.831454 0.544402
H 3.214086 3.776924 1.169988
H 2.551855 2.176556 0.874457
C -3.487469 0.848701 1.112406
H -3.764106 1.876578 0.840104
H -2.592707 0.606381 0.524976
C -4.613604 -0.116802 0.718584
H -4.310317 -1.145339 0.956417
H -5.506002 0.086959 1.327406
C -4.995629 -0.039915 -0.773412
H -5.317201 0.984572 -1.005063
H -4.100944 -0.228162 -1.380910
C -6.084404 -1.020470 -1.158297
C -7.427834 -0.759476 -0.851279
H -7.689982 0.174224 -0.357467
C -8.430938 -1.673811 -1.173579
H -9.465933 -1.448386 -0.928953
C -8.108761 -2.871950 -1.814837
H -8.889409 -3.583597 -2.070196
C -6.777237 -3.144141 -2.131261
H -6.515703 -4.069885 -2.637880
C -5.777669 -2.225270 -1.804777
H -4.742657 -2.439841 -2.063986
C 2.946442 3.310837 -0.929686
H 2.985086 4.365518 -1.240396
H 2.214570 2.825844 -1.577917
C 4.341322 2.745628 -1.213854
H 4.595672 2.827614 -2.278702
H 5.081536 3.317088 -0.648205
N 4.526202 1.337927 -0.733074
S 3.736693 0.124550 -1.750218
O 2.303776 0.400558 -1.564481
O 4.298274 0.102609 -3.104725
C 4.170116 -1.382293 -0.899933
C 3.694238 -1.602447 0.396058
H 3.096613 -0.857225 0.911854
C 4.007095 -2.806716 1.019743
H 3.644000 -2.985961 2.028543
C 4.772848 -3.789317 0.372540
C 5.221542 -3.538739 -0.931182
H 5.809893 -4.291295 -1.449995
C 4.928827 -2.337920 -1.575243
H 5.279023 -2.138212 -2.582012
C 5.119897 -5.078016 1.078555
H 5.442078 -5.849621 0.372525
H 5.937230 -4.923625 1.795078
H 4.265645 -5.466387 1.643731
Cl 6.282761 0.999564 -0.706536

89

C -0.320843 2.863110 0.009288
H -5.497951 3.721757 1.375816
C -4.462646 3.539317 1.099975
C -3.500000 3.316456 2.087217
H -3.784159 3.323272 3.136636
C -2.170312 3.086594 1.729856
H -1.424987 2.918367 2.504930
C -1.772935 3.073114 0.383661
C -2.751327 3.295463 -0.594795
H -2.465459 3.285084 -1.644554
C -4.083102 3.527910 -0.243835
H -4.823360 3.701932 -1.020912
H 0.127909 2.112583 0.672036
H -0.265528 2.449825 -1.004861
C -2.120100 -0.957409 2.252832
H -1.784339 -1.157499 3.285293
H -1.787475 0.067419 2.022247
N -1.460052 -1.898630 1.364803
S 0.236687 -1.795879 1.417848
O 0.648113 -2.547477 2.617804
O 0.678248 -0.398214 1.255751
C 0.690046 -2.709055 -0.043711
C 0.491026 -4.092881 -0.076399
H 0.051769 -4.604115 0.773872
C 0.875770 -4.799802 -1.210190
H 0.725062 -5.876097 -1.243373
C 1.466345 -4.149967 -2.307291
C 1.665606 -2.765951 -2.237088
H 2.141756 -2.242141 -3.061343
C 1.276262 -2.035837 -1.114394
H 1.447440 -0.966977 -1.071486
C 1.861639 -4.929255 -3.538479
H 0.995789 -5.092259 -4.193902
H 2.619137 -4.397580 -4.122323
H 2.260075 -5.915798 -3.278072
C -3.641105 -1.076939 2.138527
H -3.937564 -2.106902 2.375838
H -4.085562 -0.432854 2.909449
C 0.504612 4.166006 0.085971
C 2.015249 3.970608 -0.119108
H 0.342290 4.624914 1.070243
H 0.115153 4.883809 -0.650387
H 2.518839 4.931471 0.061683
H 2.398341 3.276090 0.638995
C -4.175015 -0.676579 0.757890
H -3.860323 0.352141 0.534038
H -3.710427 -1.317691 -0.003760
C -5.701079 -0.774625 0.653908
H -6.025686 -1.803016 0.866184
H -6.165675 -0.142567 1.423781
C -6.234596 -0.351002 -0.729594
H -5.905351 0.676437 -0.933366
H -5.773129 -0.984843 -1.498534
C -7.742250 -0.436753 -0.835464
C -8.551470 0.647997 -0.470421
H -8.083315 1.573259 -0.140023
C -9.942878 0.560179 -0.529627
H -10.550910 1.415520 -0.245649
C -10.552586 -0.620369 -0.958146
H -11.635978 -0.690069 -1.008629
C -9.759723 -1.708598 -1.326865
H -10.224176 -2.630690 -1.667351
C -8.368708 -1.614516 -1.265419
H -7.757672 -2.465650 -1.559554
C 2.406826 3.458301 -1.511316
H 2.037437 4.153753 -2.280017
H 1.948877 2.487587 -1.716294
C 3.921781 3.349076 -1.715922
H 4.173554 3.096870 -2.753134
H 4.393537 4.307570 -1.490101
N 4.528143 2.359608 -0.761579
S 4.692511 0.754681 -1.462501
O 3.329444 0.474791 -1.936830
O 5.817730 0.669837 -2.397230
C 5.050090 -0.227181 -0.019174
C 4.047509 -0.434718 0.932493
H 3.069538 0.021265 0.825789
C 4.317754 -1.258215 2.020629
H 3.533529 -1.439791 2.751132
C 5.571650 -1.872082 2.173199
C 6.553503 -1.645122 1.199556
H 7.526649 -2.119145 1.299154
C 6.303252 -0.827155 0.098705
H 7.058255 -0.657563 -0.660977
C 5.852358 -2.746813 3.371028
H 6.736163 -3.372827 3.214149
H 6.032567 -2.137879 4.266561
H 5.002536 -3.401342 3.592861
Cl 6.119831 2.915021 -0.180067

89

C 0.200119 3.189921 -0.160954
H -4.797249 4.884393 -1.002563
C -3.783235 4.538995 -0.822183
C -2.986331 5.162056 0.147812
H -3.387228 5.992269 0.724060
C -1.686067 4.734115 0.378031
H -1.082663 5.231795 1.131256
C -1.127494 3.647945 -0.359041
C -1.963202 3.028247 -1.335451
H -1.559132 2.203583 -1.918148
C -3.257438 3.469810 -1.562629
H -3.865788 2.985677 -2.322242
H -0.693782 1.495175 1.552865
H 0.527067 2.334118 -0.749236
C -2.047203 1.104882 3.173665
H -1.832755 0.840241 4.212142
H -2.308823 2.169694 3.157980
N -0.776571 1.007619 2.442331
S 0.263754 -0.281014 2.591796
O 0.104689 -0.788740 3.957290
O 1.557983 0.198215 2.087173
C -0.256228 -1.585516 1.465064
C -0.981294 -2.672114 1.956777
H -1.195534 -2.738898 3.018257
C -1.395398 -3.670104 1.075585
H -1.952294 -4.521708 1.459150
C -1.096582 -3.599851 -0.292172
C -0.350685 -2.506026 -0.755606
H -0.085161 -2.444814 -1.808168
C 0.074756 -1.499717 0.109402
H 0.682220 -0.680122 -0.262753
C -1.575580 -4.668558 -1.245701
H -1.691947 -5.633560 -0.741826
H -2.552179 -4.405413 -1.673873
H -0.880634 -4.801027 -2.081357
C -3.222960 0.267639 2.648886
H -2.977421 -0.798307 2.734203
H -4.069759 0.440064 3.329057
C 1.212519 3.836350 0.741662
C 2.629624 3.261413 0.581032
H 0.909772 3.719850 1.795324
H 1.227950 4.924438 0.563098
H 3.290550 3.735278 1.319319
H 2.606446 2.195475 0.823925
C -3.649274 0.585215 1.211558
H -3.898745 1.652652 1.127035
H -2.804370 0.415158 0.530210
C -4.842418 -0.259931 0.747563
H -4.590520 -1.326626 0.826771
H -5.696162 -0.097735 1.420584
C -5.277972 0.047398 -0.699397
H -5.526687 1.114060 -0.776309
H -4.425029 -0.121126 -1.370188
C -6.456046 -0.787643 -1.153927
C -7.771454 -0.363026 -0.920931
H -7.943453 0.596231 -0.436564
C -8.859609 -1.147551 -1.305103
H -9.871362 -0.796535 -1.118131
C -8.650255 -2.376538 -1.933489
H -9.496239 -2.987394 -2.237176
C -7.345936 -2.811805 -2.174248
H -7.172046 -3.764352 -2.668584
C -6.261881 -2.022661 -1.787384
H -5.247830 -2.365996 -1.983675
C 3.204558 3.467447 -0.826168
H 3.274882 4.543273 -1.045404
H 2.535375 3.041928 -1.577037
C 4.623802 2.917966 -1.018343
H 4.967374 3.060704 -2.051213
H 5.309384 3.456064 -0.358373
N 4.775760 1.486397 -0.604159
S 4.008818 0.337362 -1.709603
O 2.572363 0.603724 -1.543168
O 4.606787 0.392931 -3.047731
C 4.416030 -1.216790 -0.935875
C 3.891301 -1.513702 0.325420
H 3.268177 -0.802105 0.858116
C 4.184496 -2.752024 0.888914
H 3.782801 -2.991822 1.870141
C 4.978313 -3.693332 0.214678
C 5.475578 -3.365747 -1.053842
H 6.085886 -4.085773 -1.593069
C 5.203445 -2.129427 -1.636730
H 5.590809 -1.869946 -2.615900
C 5.302096 -5.021367 0.855785
H 5.660195 -5.747185 0.119165
H 6.085750 -4.908106 1.616248
H 4.426087 -5.446758 1.357516
Cl 6.527732 1.124627 -0.530670

89

C 0.035853 2.827130 0.196919
H -4.457482 5.057483 -1.776468
C -3.552541 4.609392 -1.375304
C -2.944203 5.151089 -0.238627
H -3.377196 6.021868 0.246648
C -1.780077 4.582512 0.272256
H -1.312228 5.016445 1.151906
C -1.196270 3.453022 -0.337819
C -1.826870 2.918785 -1.478732
H -1.387774 2.052123 -1.966940
C -2.986192 3.492193 -1.995544
H -3.446839 3.070921 -2.885086
H -0.281678 2.124494 1.184518
H 0.434085 2.072681 -0.488649
C -1.877783 1.709029 2.984110
H -1.776236 1.677931 4.078123
H -2.116145 2.748457 2.725577
N -0.522974 1.497742 2.462872
S 0.274039 0.043240 2.656957
O 0.041117 -0.366779 4.052035
O 1.637452 0.284017 2.161729
C -0.399218 -1.258062 1.606373
C -1.182204 -2.264984 2.176267
H -1.371722 -2.256047 3.244378
C -1.686073 -3.279337 1.365304
H -2.289217 -4.067319 1.810509
C -1.421139 -3.306081 -0.012394
C -0.614218 -2.296023 -0.553840
H -0.371706 -2.312539 -1.613461
C -0.095282 -1.275533 0.243144
H 0.562142 -0.528771 -0.190573
C -1.997590 -4.397156 -0.883036
H -1.858532 -5.385643 -0.430411
H -3.077463 -4.257975 -1.024180
H -1.531264 -4.409182 -1.872920
C -3.036997 0.792024 2.563879
H -2.816674 -0.239005 2.861214
H -3.907781 1.096279 3.162680
C 1.128672 3.745766 0.769309
C 2.553109 3.214531 0.538688
H 0.969154 3.874936 1.847475
H 1.030357 4.744647 0.319996
H 3.253084 3.805901 1.145291
H 2.616294 2.186274 0.905327
C -3.404314 0.843945 1.077192
H -3.690721 1.869785 0.807974
H -2.520878 0.604610 0.471684
C -4.537531 -0.121480 0.708433
H -4.234753 -1.150512 0.944232
H -5.422199 0.087571 1.326746
C -4.931326 -0.041463 -0.780479
H -5.254933 0.983755 -1.006422
H -4.039638 -0.226463 -1.393392
C -6.020033 -1.020891 -1.164530
C -7.363130 -0.759452 -0.857417
H -7.625010 0.174381 -0.363710

C -8.365974 -1.674096 -1.179301
H -9.401037 -1.449061 -0.934620
C -8.043370 -2.872321 -1.820156
H -8.823891 -3.584268 -2.075047
C -6.711893 -3.144538 -2.136904
H -6.450429 -4.070451 -2.643226
C -5.712605 -2.225473 -1.810902
H -4.677441 -2.439678 -2.069780
C 2.980331 3.286491 -0.933990
H 3.003266 4.337178 -1.259543
H 2.253774 2.779876 -1.572620
C 4.386569 2.741488 -1.214116
H 4.643651 2.830005 -2.277851
H 5.115428 3.323130 -0.643859
N 4.587900 1.335971 -0.736412
S 3.804095 0.122724 -1.756009
O 2.370380 0.395832 -1.574041
O 4.368552 0.103854 -3.109513
C 4.238450 -1.382469 -0.905378
C 3.761693 -1.601535 0.390437
H 3.162400 -0.856910 0.905230
C 4.074939 -2.805318 1.014769
H 3.711173 -2.984065 2.023411
C 4.841142 -3.787920 0.368184
C 5.290463 -3.538092 -0.935478
H 5.879168 -4.290904 -1.453546
C 4.997800 -2.337741 -1.580189
H 5.348312 -2.138155 -2.586878
C 5.188018 -5.076386 1.074622
H 5.509797 -5.848393 0.368810
H 6.005581 -4.922022 1.790904
H 4.333800 -5.464281 1.640182
Cl 6.347266 1.006343 -0.712365

N-Cl, TS-1,2'-HAT$_{bimol}$

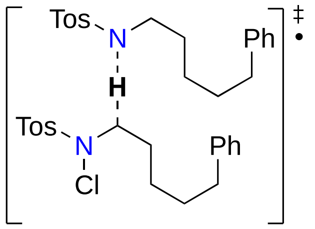

89

S 0.055258 0.000889 1.185698
N 0.668800 -0.809483 -0.190083
C 1.589574 0.788585 1.633164
C 2.317172 0.281022 2.712026
H 1.924740 -0.555302 3.280261
C 3.521685 0.887273 3.054140
H 4.083609 0.514486 3.906790
C 4.013749 1.982038 2.327368
C 5.326096 2.625550 2.702682
C 3.265311 2.461828 1.240256
H 3.621253 3.320829 0.677939
C 2.055486 1.874900 0.884086
H 1.467430 2.270024 0.063340
O -0.336605 -0.908978 2.274423
O -0.899604 0.997207 0.691284
C 1.064139 -2.192641 -0.029684
H 0.325586 -2.789380 -0.588393
C 2.467882 -2.407288 -0.632854
H 5.316281 3.701549 2.500462
H 6.155722 2.194987 2.126073
H 5.554695 2.479322 3.763143
H 1.008825 -2.538343 1.009293
H 2.714116 -3.470334 -0.507094
H 3.189796 -1.849127 -0.029963
S -3.344598 -2.335620 -0.467768
N -2.918463 -0.879748 -1.414237
C -4.420517 -1.647616 0.776559
C -5.802214 -1.755244 0.617207
H -6.210838 -2.255455 -0.253701
C -6.634094 -1.219405 1.598549
H -7.712184 -1.298883 1.484009
C -6.103947 -0.584012 2.729539
C -7.010033 0.026490 3.771745
C -4.709239 -0.511983 2.867189
H -4.281197 -0.037729 3.746822
C -3.857006 -1.038623 1.901031
H -2.780328 -0.988746 2.022539
O -4.097020 -3.297584 -1.277874
O -2.071757 -2.726402 0.150648
C -1.677126 -1.115165 -2.201616
H -1.728406 -2.078460 -2.729121
C -1.273761 -0.040434 -3.219585
H -7.097202 1.111453 3.627138
H -6.621666 -0.134008 4.783317
H -8.019608 -0.393332 3.722991
H -0.377006 -0.466618 -3.691663
H -2.028937 0.010369 -4.013683
H -0.901395 -1.170039 -1.441270
C -0.920004 1.384439 -2.751928
H -0.181356 1.336370 -1.945396
H -0.418185 1.866635 -3.604563
C -2.108352 2.259607 -2.326033
H -2.966942 2.045197 -2.974206
H -2.414089 1.992767 -1.308877
C -1.843317 3.777742 -2.428238
H -1.611180 4.023347 -3.473449
H -2.782371 4.299161 -2.195365
C 2.581261 -2.015470 -2.111936
H 1.815130 -2.564031 -2.679487
H 2.339654 -0.951293 -2.225658
C 3.948108 -2.295436 -2.760538
H 4.205692 -3.356981 -2.641258
H 3.842098 -2.130112 -3.840735
C 5.127945 -1.420674 -2.269361
H 5.903891 -1.443369 -3.047058
H 4.795567 -0.376320 -2.205129
C 5.767851 -1.829575 -0.954917
C 5.741226 -0.988295 0.165202
H 5.233439 -0.028389 0.098673
C 6.346550 -1.366995 1.365727
H 6.305541 -0.699690 2.222860
C 6.998203 -2.596198 1.466382
H 7.471627 -2.892292 2.398689
C 7.039441 -3.442493 0.355992
H 7.549277 -4.400452 0.419961
C 6.431398 -3.060409 -0.839464
H 6.478227 -3.725382 -1.699456
C -0.749495 4.339807 -1.541729
C 0.421674 4.877426 -2.092124
H 0.557318 4.860657 -3.171479
C 1.405532 5.451446 -1.282401
H 2.299413 5.873077 -1.736023
C 1.231557 5.497574 0.101206
H 1.985869 5.957467 0.734840
C 0.074185 4.953582 0.666238
H -0.069431 4.979270 1.743394
C -0.902361 4.379978 -0.146837
H -1.795690 3.956954 0.305073
Cl -4.289937 -0.484042 -2.477601

89

S 0.050309 0.010673 1.165442
N 0.512159 -0.826682 -0.216791
C 1.590372 0.782993 1.619672
C 2.305577 0.280487 2.702845
H 1.907868 -0.552504 3.272078
C 3.513653 0.882925 3.048770
H 4.070980 0.507066 3.903217
C 4.009433 1.975923 2.327045
C 5.319298 2.621799 2.708663
C 3.266680 2.454748 1.235687
H 3.625480 3.313758 0.674824
C 2.060637 1.865337 0.872851
H 1.480222 2.259848 0.046352
O -0.332172 -0.899099 2.255146
O -0.898554 1.031595 0.699177
C 1.009176 -2.214438 -0.113194
H 0.324615 -2.867167 -0.665450
C 2.438188 -2.348634 -0.640113
H 5.305866 3.699276 2.513661
H 6.152866 2.198820 2.132217
H 5.545750 2.471023 3.769045
H 0.942350 -2.538579 0.929471
H 2.760761 -3.386339 -0.474335
H 3.100878 -1.719729 -0.037804
S -3.304711 -2.384405 -0.426793
N -2.729926 -0.973773 -1.380224
C -4.394559 -1.637490 0.772739
C -5.777765 -1.747141 0.603657
H -6.180627 -2.236448 -0.275594
C -6.615586 -1.219999 1.585305
H -7.692538 -1.302749 1.463837
C -6.093909 -0.584997 2.721820
C -7.004816 0.020188 3.763554
C -4.700371 -0.506909 2.864430
H -4.277007 -0.032734 3.746150

C -3.840409 -1.031943 1.903499
H -2.764854 -0.983641 2.038828
O -4.078327 -3.274956 -1.288162
O -2.072983 -2.846154 0.220188
C -1.678787 -1.117455 -2.113968
H -1.157513 -2.081693 -2.097337
C -1.189579 -0.100534 -3.123074
H -7.095666 1.104683 3.618303
H -6.616281 -0.138838 4.775205
H -8.012553 -0.403576 3.713223
H -0.272877 -0.519708 -3.553330
H -1.946006 -0.108861 -3.922206
H -0.252313 -0.703223 -0.867645
C -0.899203 1.379795 -2.737079
H -0.129918 1.411942 -1.958754
H -0.439735 1.810303 -3.638320
C -2.099462 2.244390 -2.326986
H -2.953290 2.014668 -2.972928
H -2.405775 1.984152 -1.308746
C -1.837807 3.765497 -2.432451
H -1.607159 4.012388 -3.477693
H -2.782750 4.276327 -2.202234
C 2.574968 -2.000276 -2.127758
H 1.821014 -2.572678 -2.691324
H 2.328333 -0.939341 -2.273798
C 3.947132 -2.290655 -2.758996
H 4.196911 -3.351745 -2.621732
H 3.853546 -2.140226 -3.842759
C 5.126785 -1.417367 -2.266357
H 5.903240 -1.440437 -3.043555
H 4.796320 -0.372432 -2.201174
C 5.765340 -1.828809 -0.952127
C 5.737981 -0.989020 0.168631
H 5.228138 -0.030135 0.104590
C 6.345322 -1.367986 1.367932
H 6.303188 -0.701697 2.225701
C 6.998814 -2.596327 1.466612
H 7.473414 -2.892732 2.398253
C 7.040024 -3.441724 0.355519
H 7.551096 -4.399149 0.418092
C 6.430580 -3.058975 -0.839033
H 6.478021 -3.722753 -1.700012
C -0.749055 4.337231 -1.543755
C 0.420760 4.878216 -2.094094
H 0.556726 4.862947 -3.173573
C 1.403797 5.453344 -1.284053
H 2.297212 5.876066 -1.737389
C 1.229985 5.497653 0.099522
H 1.984831 5.955743 0.733610
C 0.073057 4.952810 0.664194
H -0.069917 4.976442 1.741328
C -0.903202 4.378638 -0.148974
H -1.795333 3.954022 0.303629
Cl -4.418930 -0.372554 -2.555717

89

S 1.730606 1.505506 1.568624
N 2.313896 0.407399 0.414053
C 2.442360 3.033430 0.979499
C 3.492322 3.611891 1.689243
H 3.867120 3.129906 2.585690
C 4.040181 4.809521 1.228843
H 4.859113 5.266254 1.778752
C 3.554642 5.431626 0.072346
C 4.136573 6.738216 -0.411437
C 2.496089 4.822597 -0.621696
H 2.109431 5.289791 -1.524310
C 1.931986 3.631674 -0.176039
H 1.106894 3.174670 -0.712682
O 2.305309 1.197480 2.887116
O 0.264164 1.582109 1.409726
C 1.989141 -0.978719 0.706191
H 0.908996 -1.115231 0.520549
C 2.796316 -1.924992 -0.189673
H 3.472252 7.578087 -0.169605
H 4.271961 6.737138 -1.498692
H 5.106765 6.941515 0.051925
H 2.151014 -1.197248 1.772826
H 2.581377 -2.950210 0.141262
H 3.863046 -1.755858 -0.014306
S -2.233172 -2.703042 -0.093169
N -3.673601 -2.009236 -0.856247
C -2.392774 -2.113420 1.579902
C -2.858121 -2.977186 2.571414
H -3.146415 -3.991221 2.317685
C -2.926928 -2.513717 3.884209
H -3.286056 -3.179671 4.664598
C -2.529142 -1.211436 4.216691
C -2.612745 -0.712414 5.638931
C -2.050615 -0.373280 3.197010
H -1.704633 0.629172 3.432793
C -1.982398 -0.810798 1.878185
H -1.589590 -0.158098 1.106877
O -2.266462 -4.167796 -0.099919
O -1.138255 -1.978662 -0.754546
C -3.608826 -2.086977 -2.341501
H -3.626021 -3.133171 -2.675103
C -4.711966 -1.290124 -3.045477
H -3.449040 -0.011710 5.759896
H -1.700687 -0.177636 5.925998
H -2.764198 -1.533899 6.345707
H -4.555341 -1.479938 -4.116262
H -5.691613 -1.721692 -2.805060
H -2.631105 -1.662658 -2.588230
C -4.730232 0.231109 -2.807514
H -3.708253 0.627437 -2.874379
H -5.287820 0.691315 -3.636105
C -5.374486 0.674582 -1.486121
H -6.369298 0.215405 -1.400868
H -4.788297 0.294115 -0.643338
C -5.540073 2.203147 -1.358611
H -6.160139 2.569283 -2.187878
H -6.106526 2.409191 -0.439587
C 2.447024 -1.782666 -1.677946
H 1.363810 -1.926295 -1.784767
H 2.653483 -0.753018 -2.000815
C 3.158423 -2.766538 -2.624123
H 3.066624 -3.788846 -2.230931
H 2.616440 -2.763758 -3.579208
C 4.641397 -2.466620 -2.945956
H 4.920811 -3.075316 -3.817631
H 4.727721 -1.420274 -3.267030
C 5.642259 -2.733549 -1.837903
C 6.388685 -1.694665 -1.266459
H 6.237233 -0.676124 -1.617427
C 7.317657 -1.945404 -0.253933
H 7.882898 -1.121898 0.175236
C 7.520329 -3.247525 0.203754
H 8.242963 -3.446132 0.990803
C 6.789472 -4.295151 -0.361777
H 6.944007 -5.314756 -0.017569
C 5.863281 -4.038616 -1.372176
H 5.305498 -4.864104 -1.809703
C -4.241406 2.986240 -1.324922
C -3.932788 3.920913 -2.321610
H -4.634744 4.079762 -3.137648
C -2.745949 4.655980 -2.278590
H -2.531742 5.380324 -3.060770
C -1.841709 4.462489 -1.234115
H -0.920571 5.038630 -1.192109
C -2.129788 3.528294 -0.235237
H -1.427751 3.356362 0.575265
C -3.319704 2.802369 -0.283115
H -3.536783 2.082947 0.503682
Cl -5.124283 -2.849756 -0.249421

89

S 0.824800 1.590369 1.333051
N 1.269081 0.038694 0.830091
C 2.423047 2.376009 1.530224
C 2.684480 3.092133 2.696625
H 1.947783 3.106570 3.492782
C 3.896256 3.772660 2.818075
H 4.105529 4.329216 3.728172
C 4.847395 3.748733 1.791027
C 6.169526 4.462924 1.939409
C 4.553703 3.024755 0.623762
H 5.277667 3.001251 -0.187070
C 3.348732 2.343121 0.482714
H 3.130184 1.778908 -0.417454
O 0.169997 1.598087 2.653718
O 0.104742 2.165988 0.186230
C 1.774805 -0.885012 1.875376
H 0.947743 -1.251789 2.494436
C 2.553174 -2.046977 1.249165
H 6.471875 4.944335 1.003196
H 6.967216 3.760266 2.214719
H 6.126281 5.230345 2.718293
H 2.444575 -0.310264 2.523315
H 2.961983 -2.644950 2.075899
H 3.410177 -1.647066 0.698580
S -2.857493 -1.947992 1.089054
N -3.534267 -1.857251 -0.530240
C -3.767383 -0.688965 1.951982
C -5.114657 -0.918431 2.252392
H -5.602089 -1.832216 1.933277
C -5.812758 0.053972 2.961863
H -6.860782 -0.112373 3.198578
C -5.187273 1.239053 3.380743
C -5.963536 2.299342 4.124390
C -3.830882 1.425608 3.078405
H -3.321492 2.326618 3.409706
C -3.109462 0.471833 2.363205
H -2.054639 0.623090 2.161171
O -3.223554 -3.272908 1.586137
O -1.437416 -1.552919 1.002005
C -2.722040 -1.749565 -1.513002
H -1.655054 -1.657130 -1.289272
C -3.149490 -1.750937 -2.944485
H -6.357069 3.053680 3.428730
H -5.331185 2.823621 4.848717
H -6.817891 1.871773 4.660389
H -2.647828 -2.600353 -3.432765
H -4.227890 -1.913242 -2.994163
H 0.446965 -0.329663 0.347446
C -2.740621 -0.449882 -3.677441
H -1.647817 -0.359339 -3.701064
H -3.064813 -0.553060 -4.721610
C -3.360021 0.813847 -3.065010
H -4.443909 0.670432 -2.957100
H -2.971353 0.961627 -2.048068
C -3.111190 2.090647 -3.894491
H -3.589786 1.975591 -4.876422
H -3.626260 2.923597 -3.396820
C 1.700545 -2.952564 0.346634
H 0.781495 -3.214694 0.887803
H 1.377561 -2.390459 -0.542079
C 2.373773 -4.259935 -0.112552
H 2.812379 -4.770260 0.756510
H 1.586747 -4.927339 -0.487432
C 3.436896 -4.141145 -1.231185
H 3.609824 -5.151708 -1.627136
H 3.016831 -3.558382 -2.062054
C 4.776136 -3.551574 -0.830712
C 5.232952 -2.341905 -1.371372
H 4.608563 -1.808661 -2.085823
C 6.474292 -1.812650 -1.009965
H 6.806500 -0.872830 -1.444860
C 7.285924 -2.489168 -0.098290
H 8.253616 -2.081545 0.183085
C 6.847716 -3.700310 0.443701
H 7.475610 -4.240905 1.148069
C 5.607956 -4.224466 0.077530
H 5.282099 -5.173906 0.498703
C -1.649287 2.450210 -4.091720
C -1.086274 2.497235 -5.373112
H -1.708307 2.273956 -6.238035
C 0.256799 2.835585 -5.558360
H 0.670104 2.870482 -6.563665
C 1.061463 3.128894 -4.457015
H 2.106449 3.393559 -4.597977
C 0.513524 3.080844 -3.172365
H 1.123287 3.303167 -2.300928
C -0.828070 2.746710 -2.992401
H -1.227197 2.717782 -1.981928
Cl -5.967139 -2.207978 -0.958685

89

S 0.520277 -0.761238 -1.170845
N 1.521739 0.419076 -0.515466
C 1.687263 -2.053181 -1.591544
C 1.776778 -2.491668 -2.910798
H 1.165130 -2.023244 -3.674221
C 2.653767 -3.532290 -3.221842
H 2.726038 -3.878011 -4.249822
C 3.438575 -4.138131 -2.234188
C 4.398820 -5.252504 -2.574491
C 3.323474 -3.677612 -0.910991
H 3.924939 -4.137443 -0.130436
C 2.454095 -2.643374 -0.581386
H 2.370185 -2.291679 0.441458
O -0.125156 -0.327557 -2.424310
O -0.327703 -1.215527 -0.052717
C 2.317891 1.216586 -1.474276
H 1.667249 1.802309 -2.138514
C 3.300157 2.129513 -0.733019
H 4.268860 -6.109263 -1.903308
H 5.439978 -4.920166 -2.474383
H 4.259083 -5.603001 -3.601349
H 2.877106 0.510792 -2.097826
H 3.959706 2.578569 -1.488571
H 3.935960 1.515988 -0.087470
S -2.062304 2.671291 0.790253
N -3.060272 1.521627 1.647114
C -2.957421 2.873248 -0.734081
C -4.010503 3.791618 -0.771583
H -4.272668 4.357275 0.115019
C -4.706457 3.959426 -1.964463
H -5.525485 4.672922 -2.004916
C -4.369915 3.225233 -3.112105
C -5.155367 3.396016 -4.389916
C -3.304983 2.316600 -3.039330
H -3.023098 1.745603 -3.920046
C -2.591422 2.128416 -1.857331
H -1.766556 1.424141 -1.823781
O -2.116172 3.900549 1.573439
O -0.762173 2.035972 0.505707
C -2.579552 0.382355 1.971841
H -1.571125 0.101575 1.643115
C -3.352577 -0.587060 2.813385
H -6.066834 2.784028 -4.371939
H -4.572156 3.089623 -5.263845
H -5.466935 4.436122 -4.532989
H -2.957717 -0.474632 3.836058
H -4.398418 -0.268490 2.842786
H 0.920141 1.001299 0.073409
C -3.212265 -2.059127 2.383309
H -2.172138 -2.386948 2.495947
H -3.802181 -2.658240 3.089489
C -3.685097 -2.330103 0.947849
H -4.627471 -1.795965 0.763780
H -2.952374 -1.916408 0.243617
C -3.924060 -3.823768 0.645087
H -4.790209 -4.165986 1.226701

H -4.215957 -3.914009 -0.411119
C 2.621524 3.252895 0.067653
H 1.909853 3.765330 -0.595073
H 2.015930 2.831593 0.881574
C 3.569672 4.313189 0.657118
H 4.253924 4.670829 -0.125349
H 2.961796 5.180216 0.946232
C 4.382320 3.894259 1.904172
H 4.789146 4.810035 2.355676
H 3.694127 3.471634 2.647662
C 5.527439 2.926417 1.673668
C 5.531902 1.654030 2.260084
H 4.691608 1.352572 2.881865
C 6.594985 0.770363 2.059843
H 6.575185 -0.211357 2.526478
C 7.679654 1.147669 1.267924
H 8.509561 0.463516 1.111985
C 7.693437 2.416040 0.682636
H 8.537409 2.723951 0.070388
C 6.629426 3.294043 0.886591
H 6.656987 4.282788 0.433177
C -2.756874 -4.754885 0.923229
C -2.937313 -5.900260 1.709880
H -3.917839 -6.103927 2.135555
C -1.884630 -6.785176 1.952271
H -2.051450 -7.667711 2.564971
C -0.623562 -6.534127 1.411753
H 0.199245 -7.219463 1.599120
C -0.426917 -5.392661 0.631046
H 0.552537 -5.181564 0.209081
C -1.482185 -4.512839 0.388465
H -1.305098 -3.622185 -0.207531
Cl -5.354979 2.233458 2.264681

89

S -0.030259 -0.022138 1.135369
N 0.401439 -0.840137 -0.291308
C 1.532718 0.737822 1.585366
C 2.243083 0.242988 2.680385
H 1.846680 -0.594313 3.244261
C 3.440880 0.856331 3.045075
H 3.989116 0.482497 3.906431
C 3.941682 1.953043 2.329727
C 5.252208 2.597424 2.715202
C 3.202037 2.432094 1.236572
H 3.557922 3.297019 0.682914
C 2.003136 1.834782 0.857918
H 1.427309 2.232869 0.029954
O -0.399363 -0.924939 2.241290
O -0.972177 1.025614 0.718465
C 0.907622 -2.215524 -0.130959
H 0.252524 -2.874743 -0.714668
C 2.355047 -2.350054 -0.635304
H 5.241810 3.674716 2.518912
H 6.085386 2.171724 2.140085
H 5.476951 2.446455 3.775971
H 0.814558 -2.552384 0.906966
H 2.666078 -3.388900 -0.453915
H 3.010620 -1.720765 -0.025582
S -3.395868 -2.388374 -0.437439
N -2.910779 -0.946670 -1.370310
C -4.470610 -1.672499 0.786901
C -5.852241 -1.777352 0.622053
H -6.259440 -2.280013 -0.248087
C -6.684983 -1.238477 1.600321
H -7.762836 -1.317646 1.484313
C -6.155691 -0.602913 2.731940
C -7.063352 0.003867 3.774624
C -4.761021 -0.527985 2.870519
H -4.334248 -0.050752 3.748954
C -3.906265 -1.057902 1.908506
H -2.829309 -1.006914 2.032846
O -4.159068 -3.310957 -1.278203
O -2.138183 -2.807657 0.183824
C -1.620378 -1.068952 -1.984659
H -1.371358 -2.104341 -2.255495
C -1.234207 -0.096788 -3.093046
H -7.152307 1.088842 3.631584
H -6.674517 -0.157498 4.785769
H -8.071932 -0.418023 3.724786
H -0.315270 -0.517021 -3.520026
H -1.991436 -0.156347 -3.887837
H -0.731840 -0.875260 -1.061750
C -0.950218 1.382071 -2.750713
H -0.174540 1.425946 -1.979599
H -0.510036 1.819843 -3.658833
C -2.152804 2.234404 -2.326341
H -3.007825 2.011230 -2.975435
H -2.451760 1.965243 -1.307577
C -1.899084 3.755014 -2.424299
H -1.670851 4.005746 -3.469146
H -2.842104 4.267374 -2.188310
C 2.512406 -2.014992 -2.124044
H 1.760694 -2.588167 -2.688519
H 2.267427 -0.956067 -2.280395
C 3.887357 -2.313654 -2.746679
H 4.135819 -3.374299 -2.602735
H 3.795414 -2.170054 -3.831585
C 5.068430 -1.439270 -2.259350
H 5.843890 -1.464390 -3.037570
H 4.738446 -0.394145 -2.195549
C 5.708005 -1.849534 -0.945684
C 5.680560 -1.009911 0.175079
H 5.170342 -0.051245 0.110963
C 6.288333 -1.388813 1.374198
H 6.246034 -0.722748 2.232174
C 6.942080 -2.617000 1.472731
H 7.416746 -2.913397 2.404372
C 6.983264 -3.462400 0.361593
H 7.494381 -4.419837 0.424134
C 6.373612 -3.079633 -0.832739
H 6.420638 -3.743309 -1.693810
C -0.806828 4.319852 -1.536930
C 0.363445 4.858621 -2.087878
H 0.499009 4.842561 -3.167332
C 1.347180 5.432533 -1.278032
H 2.241062 5.854255 -1.731426
C 1.173504 5.477073 0.105589
H 1.928702 5.934938 0.739481
C 0.017074 4.931778 0.670762
H -0.124866 4.953901 1.748066
C -0.959648 4.358541 -0.142203
H -1.850743 3.932196 0.310671
Cl -4.276741 -0.477820 -2.453187

TS-1,5-HAT$_{CC,uni}$ pathway

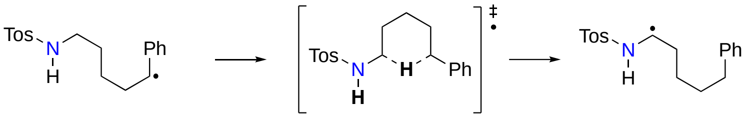

C_6-rad$_{gm}$ TS-1,5-HAT$_{CC,uni}$ C_2-rad$_{gm}$

Name	E(B3LYP)	G(B3LYP)	E(RO-B2PLYP-D3)	G(RO-B2PLYP-D3)
**N-rad$_{gm}$**				
-1302.44553888	-1302.137918	-1301.9129157	-1301.6052948456	-1302.44553888
**C_6-rad$_{gm}$**				
TScrad15crad.C6.conf059	-1302.45907388	-1302.147252	-1301.9349086884	-1301.6230868084
TScrad15crad.C6.conf033	-1302.45627173	-1302.145546	-1301.9333656230	-1301.6226398930
TScrad15crad.C6.conf073	-1302.45760453	-1302.147502	-1301.9324668510	-1301.6223643210
TScrad15crad.C6.conf067	-1302.45591863	-1302.144403	-1301.9334490403	-1301.6219334103
TScrad15crad.C6.conf065	-1302.45676546	-1302.146196	-1301.9321549375	-1301.6215854775
**C_6-rad$_{pic}$**				
TScrad15crad.TS.conf055_orc_fwd	-1302.45759135	-1302.147527	-1301.9324541234	-1301.6223897734
TScrad15crad.TS.conf195_orc_rev	-1302.45621893	-1302.147054	-1301.9303057205	-1301.6211407905
TScrad15crad.TS.conf009_orc_rev	-1302.45463835	-1302.145406	-1301.9298046610	-1301.6205723110
TScrad15crad.TS.conf020_orc_rev	-1302.45528506	-1302.146490	-1301.9269731960	-1301.6181781360
**TS-1,5-HAT$_{CC,uni}$**				
TScrad15crad.TS.conf003	-1302.41866363	-1302.111532	-1301.8962848298	-1301.5891531998
TScrad15crad.TS.conf009	-1302.41823982	-1302.111225	-1301.8950906838	-1301.5880758638
TScrad15crad.TS.conf055	-1302.41800174	-1302.111067	-1301.8944767021	-1301.5875419621
TScrad15crad.TS.conf195	-1302.41780788	-1302.111705	-1301.8934098601	-1301.5873069801
**C_2-rad$_{ric}$**				
TScrad15crad.TS.conf195_orc_fwd	-1302.44520930	-1302.134464	-1301.9241821385	-1301.6134368385
TScrad15crad.TS.conf009_orc_fwd	-1302.44450941	-1302.135377	-1301.9209060440	-1301.6117736340
TScrad15crad.TS.conf055_orc_rev	-1302.44430202	-1302.135430	-1301.9197333150	-1301.6108612950
TScrad15crad.TS.conf020_orc_fwd	-1302.44388444	-1302.135740	-1301.9184597151	-1301.6103152751
**C_2-rad$_{gm}$**				
TScrad15crad.C2rad.conf18	-1302.44649895	-1302.138121	-1301.9250302632	-1301.6166523132
TScrad15crad.C2rad.conf98	-1302.44716843	-1302.139527	-1301.9236714961	-1301.6160300661
TScrad15crad.C2rad.conf06	-1302.44620010	-1302.135658	-1301.9261142783	-1301.6155721783
TScrad15crad.C2rad.conf19	-1302.44578106	-1302.135972	-1301.9251068918	-1301.6152978318
TScrad15crad.C2rad.conf60	-1302.44700150	-1302.137840	-1301.9242044514	-1301.6150429514

44
-1302.44553888 Nrad,gm
C -0.238134 -3.358884 -0.417824
N -0.609805 -1.951523 -0.427561
S -1.998149 -1.648411 0.514591
O -3.116568 -2.419032 -0.049923
O -1.610544 -1.810507 1.924776
C -2.288903 0.084461 0.198270
C -1.674282 1.042582 1.005501
C -3.142903 0.455088 -0.841819
C -1.915949 2.391813 0.753519
H -1.027868 0.734372 1.820167
C -3.370678 1.808502 -1.078048
H -3.625058 -0.306996 -1.444639
C -2.764524 2.795810 -0.287270
H -4.033993 2.103660 -1.887281
C 1.173473 -3.585353 -0.970638
H 1.253131 -4.650754 -1.223993
H 1.275072 -3.035891 -1.914106
C 2.316188 -3.234365 -0.000627
H 3.257217 -3.592444 -0.442056
H 2.176940 -3.813917 0.923155
C 2.475679 -1.750723 0.370010
C 2.848198 -0.839306 -0.817181
H 2.052581 -0.880181 -1.569952
H -1.441914 3.142714 1.380595
C -3.046909 4.258777 -0.531825
H -3.961318 4.573370 -0.011710

H -2.230885 4.891502 -0.168779
H -3.192031 4.466214 -1.597146
H 3.258369 -1.667856 1.136055
C 3.067829 0.600089 -0.402369
C 1.993901 1.499617 -0.349664
C 4.338101 1.060257 -0.030292
C 2.183324 2.818769 0.063777
H 1.001561 1.160040 -0.637279
C 4.532637 2.378876 0.383906
H 5.185156 0.378104 -0.070592
C 3.454259 3.263996 0.432560
H 1.336559 3.500339 0.092063
H 5.527566 2.715986 0.663918
H 3.604256 4.292370 0.750875
H -0.976728 -3.882605 -1.048841
H 1.555928 -1.374761 0.835108
H 3.758117 -1.229318 -1.294077
H -0.344582 -3.792423 0.589237
44
-1301.62308680840000 TScrad15crad.C6.conf059
S 1.346150 -1.435409 -0.962068
N 0.581517 -2.096293 0.377700
C 2.492765 -0.182300 -0.374786
C 2.054143 1.130983 -0.190616
H 1.035944 1.404759 -0.446496
C 2.945897 2.082681 0.301329
H 2.606482 3.105742 0.443904
C 4.272297 1.747174 0.606297
C 5.240804 2.793162 1.104545
C 4.687659 0.422453 0.406136
H 5.714930 0.144489 0.629457
C 3.810251 -0.542986 -0.081836
H 4.142074 -1.561507 -0.255200
O 2.136456 -2.530677 -1.527404
O 0.306183 -0.739344 -1.726776
C -0.256114 -1.255455 1.245844
C -1.359070 -2.075357 1.926383
H 5.789928 3.248787 0.269928
H 5.983267 2.361433 1.783805
H 4.723194 3.600181 1.632920
H -0.904854 -2.786287 2.631365
C -2.284634 -2.842162 0.968084
H -1.946153 -1.373994 2.533720
H 0.347967 -0.736115 2.004508
H -0.707511 -0.490999 0.610044
C -2.940617 -1.981117 -0.137870
C -3.757712 -0.842231 0.388131
H -3.069350 -3.325078 1.566438
H -1.720738 -3.646450 0.481883
H -3.590190 -2.651682 -0.724756
H -2.169173 -1.636290 -0.832095
H 1.200956 -2.745003 0.857317
H -4.522601 -1.100367 1.121687
C -3.659063 0.524766 0.032470
C -4.530893 1.475513 0.646103
H -5.256424 1.119982 1.374510
C -4.470660 2.823714 0.333508
H -5.150272 3.519734 0.819203
C -3.541253 3.293491 -0.605912
H -3.496608 4.350545 -0.852765
C -2.674663 2.383255 -1.225789
H -1.955529 2.736345 -1.961037
C -2.723606 1.029375 -0.922460
H -2.038651 0.352781 -1.423080

44
-1301.62263989300000 TScrad15crad.C6.conf033
S 2.364444 -0.981747 -1.087129
N 2.141930 -2.203067 0.035747
C 1.970169 0.595909 -0.318133
C 0.680312 1.118741 -0.427033
H -0.074014 0.595016 -1.004222
C 0.381246 2.328033 0.199355
H -0.625601 2.729019 0.115969
C 1.353052 3.029189 0.926370
C 1.029281 4.355411 1.572439
C 2.643573 2.486092 1.011281
H 3.413783 3.020196 1.562858
C 2.959865 1.278254 0.393776
H 3.966463 0.875711 0.442142
O 3.805722 -0.973683 -1.347235
O 1.367651 -1.211624 -2.136017
C 0.823937 -2.703275 0.433454
C 0.248261 -2.083896 1.716187
H 1.238984 5.188048 0.887855
H 1.627256 4.519433 2.475078
H -0.028569 4.419555 1.847292
H 1.001643 -2.169583 2.512289
C -1.044354 -2.770698 2.187337
H 0.071920 -1.012095 1.566020
H 0.165462 -2.547495 -0.424705
H 0.918431 -3.790476 0.560344
C -2.278510 -2.535710 1.282356
C -2.768439 -1.121108 1.305920
H -1.283397 -2.420852 3.200306
H -0.870533 -3.852858 2.271344
H -3.082267 -3.196602 1.647113
H -2.064573 -2.869177 0.261488
H 2.872680 -2.210705 0.740779
H -2.981803 -0.714863 2.295008
C -3.009938 -0.264292 0.206296
C -3.513387 1.052316 0.440275
H -3.706117 1.359269 1.465786
C -3.762446 1.930303 -0.602611
H -4.151673 2.922843 -0.389231
C -3.520144 1.544203 -1.929477
H -3.713996 2.233185 -2.746351
C -3.026810 0.258555 -2.189939
H -2.833733 -0.049568 -3.214068
C -2.778119 -0.630800 -1.153635
H -2.397236 -1.619485 -1.388550

44
-1301.62236432100000 TScrad15crad.C6.conf073
S -1.306633 -1.190819 -1.158894
N -0.634253 -2.088346 0.092841
C -2.502846 -0.060836 -0.436603
C -2.095610 1.208087 -0.019038
H -1.070092 1.526327 -0.174598
C -3.026914 2.060550 0.571949
H -2.712165 3.049643 0.895683
C -4.361409 1.669421 0.747586
C -5.371277 2.612744 1.356420
C -4.744487 0.391617 0.313559
H -5.777298 0.073340 0.433691
C -3.827697 -0.474664 -0.277057
H -4.134188 -1.453511 -0.631478
O -0.226111 -0.371226 -1.714640
O -2.044551 -2.166610 -1.963954
C 0.079315 -1.424393 1.194375
C 1.032265 -2.407622 1.889967
H -5.885643 3.192291 0.578443
H -4.894530 3.328386 2.033866
H -6.139352 2.070570 1.917821
H 1.448847 -1.897426 2.769755
C 2.173753 -2.965756 1.022225
H 0.439706 -3.246959 2.282723
H 0.644492 -0.596996 0.760156
H -0.622766 -1.004122 1.930487
C 3.314235 -1.972514 0.707165
C 2.931060 -0.872356 -0.235646
H 1.757830 -3.355238 0.084836
H 2.609299 -3.824386 1.549307
H 4.144414 -2.556641 0.271341
H 3.707008 -1.566453 1.649240
H -1.280964 -2.820163 0.379031
H 2.236603 -1.135022 -1.030945
C 3.391900 0.467020 -0.210321
C 2.870976 1.402297 -1.156364
H 2.120265 1.057990 -1.862816

C 3.289667 2.723213 -1.168076
H 2.873415 3.411793 -1.899436
C 4.244187 3.176728 -0.245637
H 4.572838 4.212215 -0.259139
C 4.771411 2.279871 0.692778
H 5.514620 2.622399 1.408954
C 4.359316 0.954719 0.717165
H 4.791798 0.277433 1.447570

44
-1301.62193341030000 TScrad15crad.C6.conf067
S 1.085244 1.550764 1.407441
N -0.344675 1.745313 0.545407
C 2.089190 0.346667 0.523573
C 3.036842 0.781042 -0.404795
H 3.194213 1.843660 -0.556252
C 3.788729 -0.161897 -1.104812
H 4.529869 0.175985 -1.825229
C 3.613718 -1.535261 -0.888537
C 4.457653 -2.549702 -1.623102
C 2.657760 -1.944770 0.053389
H 2.514735 -3.006235 0.242359
C 1.896798 -1.016933 0.761023
H 1.171694 -1.340623 1.500476
O 1.791743 2.831911 1.336878
O 0.686584 0.937707 2.675331
C -0.307251 2.318160 -0.806902
C -1.625757 3.025797 -1.149655
H 5.345773 -2.820290 -1.036814
H 4.808280 -2.159887 -2.584119
H 3.901068 -3.473699 -1.812393
H -1.722774 3.892930 -0.485231
C -2.914221 2.195056 -1.039940
H -1.532567 3.417636 -2.172753
H 0.497004 3.058565 -0.814707
H -0.064644 1.553589 -1.558589
C -3.039180 0.984201 -2.007097
C -2.185024 -0.206488 -1.680832
H -3.757543 2.868907 -1.240426
H -3.043509 1.851625 -0.005956
H -2.809348 1.324479 -3.024993
H -4.096893 0.683401 -2.019396
H -0.923623 0.912436 0.637073
H -1.289429 -0.369161 -2.276936
C -2.447735 -1.151430 -0.656403
C -1.540224 -2.230884 -0.432579
H -0.653214 -2.303231 -1.057378
C -1.769448 -3.173746 0.556956
H -1.061640 -3.986142 0.701291
C -2.908668 -3.087766 1.370194
H -3.084971 -3.826359 2.146651
C -3.816855 -2.039817 1.172468
H -4.702497 -1.967287 1.798354
C -3.599180 -1.089890 0.184441
H -4.320664 -0.290698 0.046053

44
-1301.62158547750000 TScrad15crad.C6.conf065
S -1.431907 -1.904178 0.879648
N -0.946151 -2.657964 -0.556241
C -2.073602 -0.340898 0.285336
C -1.322791 0.820957 0.456379
H -0.358204 0.777316 0.950375
C -1.835557 2.033122 -0.009279
H -1.245369 2.937133 0.115439
C -3.088138 2.099427 -0.629771
C -3.652007 3.416099 -1.108650
C -3.825086 0.912544 -0.780997
H -4.802036 0.946594 -1.257611
C -3.328620 -0.305191 -0.329425
H -3.904062 -1.218289 -0.440859
O -2.536981 -2.738574 1.365066
O -0.288244 -1.595493 1.750053
C 0.354785 -2.313161 -1.153968
C 1.578521 -2.950839 -0.482841
H -4.086306 3.324874 -2.110774
H -2.882596 4.193511 -1.141334
H -4.450346 3.768622 -0.442684
H 1.620153 -2.610789 0.556266
C 2.905809 -2.646649 -1.198446
H 1.437582 -4.040966 -0.453701
H 0.296695 -2.609215 -2.209717
H 0.429302 -1.221188 -1.142432
C 3.240925 -1.145702 -1.349041
C 3.246612 -0.399983 -0.049713
H 2.903340 -3.102222 -2.198500
H 3.715895 -3.137063 -0.642330
H 2.541577 -0.681322 -2.055375
H 4.230504 -1.071824 -1.833132
H -1.112562 -3.657339 -0.446767
H 3.586682 -0.949050 0.827320
C 2.864513 0.949004 0.150444
C 2.864459 1.496069 1.469257
H 3.159459 0.855985 2.297070
C 2.487391 2.807657 1.709219
H 2.494212 3.191424 2.726168
C 2.099386 3.641043 0.649250
H 1.812125 4.671575 0.838290
C 2.096088 3.132716 -0.656632
H 1.805516 3.773619 -1.485538
C 2.467017 1.818767 -0.908015
H 2.465582 1.453851 -1.930449

44
-1301.62238977340000 TScrad15crad.TS.conf055_orc_fwd
S 1.306659 -1.194075 1.158545
N 0.636050 -2.087736 -0.096892
C 2.501536 -0.060433 0.439708
C 2.093612 1.210529 0.029393
H 1.068954 1.528742 0.190581
C 3.025443 2.068406 -0.553126
H 2.711786 3.061795 -0.864393
C 4.359846 1.678731 -0.732020
C 5.356747 2.604581 -1.387267
C 4.745313 0.401399 -0.298296
H 5.781026 0.089092 -0.408608
C 3.828176 -0.470175 0.283749
H 4.137043 -1.447187 0.641149
O 0.224771 -0.377941 1.716713
O 2.045350 -2.171924 1.960381
C -0.077978 -1.420423 -1.196089
C -1.029965 -2.401918 -1.895442
H 5.069231 3.653801 -1.266547
H 5.427872 2.406920 -2.465162
H 6.359802 2.475425 -0.967399
H -1.446749 -1.888900 -2.773491
C -2.171210 -2.963971 -1.029912
H -0.436661 -3.239385 -2.291062
H -0.643939 -0.595077 -0.758969
H 0.623863 -0.996846 -1.930537
C -3.312893 -1.972853 -0.712298
C -2.930995 -0.874897 0.233569
H -1.755240 -3.355849 -0.093547
H -2.605675 -3.821448 -1.559758
H -4.142393 -2.559144 -0.278108
H -3.705985 -1.564705 -1.653310
H 1.283844 -2.817590 -0.385635
H -2.238099 -1.139713 1.029527
C -3.390637 0.464921 0.210157
C -2.870650 1.397767 1.159130
H -2.121588 1.051315 1.866283
C -3.288154 2.719042 1.172798
H -2.872655 3.405715 1.906377
C -4.240517 3.175352 0.249509
H -4.568246 4.211111 0.264542
C -4.766803 2.280919 -0.691750
H -5.508357 2.625611 -1.408603
C -4.355881 0.955444 -0.718116
H -4.787632 0.280080 -1.450730

44
-1301.62114079050000 TScrad15crad.TS.conf195_orc_rev
S 2.820053 -0.801438 -0.756989
N 1.877132 -1.957996 0.047577
C 2.121298 0.743300 -0.175713
C 1.554724 1.625399 -1.092102
H 1.509255 1.353009 -2.140894
C 1.060205 2.850914 -0.638970
H 0.620895 3.543638 -1.352347
C 1.125919 3.203768 0.713854
C 0.606797 4.535590 1.201053
C 1.703724 2.292777 1.615686
H 1.766071 2.551196 2.670144
C 2.205701 1.071195 1.181608
H 2.661670 0.378139 1.881008
O 4.162348 -0.966582 -0.187201
O 2.604348 -0.859530 -2.208666
C 0.637499 -2.416112 -0.610456
C -0.047855 -3.479163 0.257174
H 0.085695 5.078199 0.406714
H 1.426912 5.171867 1.556913
H -0.089484 4.410386 2.038561
H -0.891211 -3.885928 -0.317155
C -0.537956 -3.015659 1.641505
H 0.653529 -4.316510 0.388308
H -0.013860 -1.546028 -0.735929
H 0.842225 -2.821898 -1.609655
C -1.874660 -2.242173 1.642976
C -1.814226 -0.870329 1.044036
H 0.241121 -2.409439 2.120626
H -0.669546 -3.903804 2.272570
H -2.203539 -2.160484 2.694532
H -2.637805 -2.854677 1.146073
H 2.510074 -2.723344 0.282375
H -0.928422 -0.281231 1.279409
C -2.818617 -0.234619 0.273119
C -2.604915 1.094251 -0.203731
H -1.665437 1.586918 0.034808
C -3.558914 1.753204 -0.962117
H -3.363843 2.764058 -1.311813
C -4.771717 1.125946 -1.281348
H -5.518793 1.644206 -1.875693
C -5.009720 -0.176857 -0.824503
H -5.948307 -0.670269 -1.064696
C -4.061521 -0.847504 -0.065241
H -4.276138 -1.853707 0.280881

44
-1301.62057231100000 TScrad15crad.TS.conf009_orc_rev
S -2.575204 -1.463515 0.820172
N -1.512624 -2.344092 -0.144288
C -2.477113 0.251869 0.287304
C -3.355402 0.717816 -0.695239
H -4.119722 0.056814 -1.090835
C -3.257257 2.038112 -1.126054
H -3.949360 2.405271 -1.880345
C -2.290653 2.906357 -0.595111
C -2.183351 4.330145 -1.087471
C -1.428268 2.416772 0.394503
H -0.683752 3.077219 0.832035
C -1.514889 1.098026 0.841377
H -0.862664 0.735757 1.628847
O -3.908230 -1.955468 0.466252
O -2.043818 -1.526510 2.182436
C -0.073203 -2.045690 -0.086817
C 0.711644 -3.136584 -0.822610
H -1.691927 4.373552 -2.068387
H -1.600218 4.949770 -0.399322
H -3.171710 4.788714 -1.202504
H 0.437530 -4.100134 -0.376788
C 2.240073 -2.967249 -0.764373
H 0.390627 -3.179952 -1.874829
H 0.156205 -1.053216 -0.498666
H 0.206652 -2.038359 0.970349
C 2.848781 -1.910406 -1.727839
C 2.498169 -0.477351 -1.455052
H 2.697750 -3.933097 -1.014601
H 2.544038 -2.740141 0.265941
H 2.539850 -2.164274 -2.750484
H 3.940473 -2.037058 -1.701320
H -1.891112 -2.427002 -1.085879
H 1.706761 -0.029564 -2.052985
C 3.103918 0.351862 -0.478374
C 2.655821 1.695762 -0.308892
H 1.845051 2.056695 -0.937652
C 3.228202 2.535192 0.633469
H 2.867060 3.555483 0.736903
C 4.271222 2.077684 1.451608
H 4.718591 2.736709 2.190074
C 4.731162 0.762437 1.306903
H 5.539769 0.400678 1.936986
C 4.165658 -0.086768 0.366096
H 4.541049 -1.101080 0.270643

44
-1301.61817813600000 TScrad15crad.TS.conf020_orc_rev
S -1.998991 -1.489867 -1.108586
N -1.128062 -2.116295 0.205096
C -2.780601 -0.047630 -0.389132
C -3.832869 -0.217785 0.515424
H -4.164935 -1.216243 0.780079
C -4.445743 0.905746 1.058442
H -5.264827 0.778865 1.762446
C -4.028380 2.201683 0.710536
C -4.719641 3.410454 1.294933
C -2.975258 2.341264 -0.200434
H -2.640343 3.335976 -0.483568
C -2.348443 1.224768 -0.756885
H -1.540652 1.331700 -1.472777
O -1.093545 -1.017062 -2.165141
O -3.019624 -2.505524 -1.390892
C 0.217623 -1.562031 0.450748
C 0.840323 -2.268063 1.658401
H -4.841044 3.315588 2.380095
H -5.723341 3.534980 0.868430
H -4.159256 4.328816 1.095393
H 0.121748 -2.213437 2.485850
C 2.184055 -1.688781 2.131219
H 0.968335 -3.337412 1.429852
H 0.854183 -1.642196 -0.437208
H 0.098119 -0.494404 0.668131
C 3.410715 -1.958617 1.215505
C 3.451187 -1.197485 -0.077024
H 2.405701 -2.126433 3.113551
H 2.081376 -0.607451 2.290884
H 3.451294 -3.034871 1.001923
H 4.311259 -1.733338 1.804554
H -1.131697 -3.130662 0.093883
H 3.144789 -1.717732 -0.981921
C 3.850857 0.154345 -0.223382
C 3.812811 0.771489 -1.508964
H 3.467735 0.185329 -2.357415
C 4.199552 2.089668 -1.689793
H 4.158261 2.531008 -2.682340
C 4.642541 2.855287 -0.601610
H 4.946038 3.888405 -0.745149
C 4.691235 2.274766 0.672380
H 5.035684 2.861224 1.520617
C 4.305235 0.955631 0.865176
H 4.356015 0.526645 1.861398

44
-1301.58915319980000 TScrad15crad.TS.conf003
S 2.739240 0.720087 0.631627
N 1.795050 1.932512 -0.057093
C 2.008076 -0.863204 0.208727
C 2.418299 -1.519193 -0.954537
H 3.191578 -1.082075 -1.577589
C 1.845179 -2.746445 -1.280130
H 2.167508 -3.262464 -2.181337

C 0.870589 -3.333442 -0.459799
C 0.283147 -4.682598 -0.799458
C 0.477906 -2.652795 0.701624
H -0.278104 -3.088990 1.349160
C 1.040300 -1.424990 1.044049
H 0.747467 -0.915072 1.955333
O 4.017969 0.809878 -0.075388
O 2.613481 0.887263 2.079628
C 0.517443 2.322417 0.453651
C 0.038395 3.667961 -0.073110
H 0.305923 -4.871224 -1.877643
H -0.754567 -4.764831 -0.459619
H 0.848220 -5.489903 -0.315001
H -0.707080 4.063686 0.628459
C -0.612195 3.548586 -1.466837
H 0.870190 4.386188 -0.092405
H -0.513251 1.514199 -0.062179
H 0.497665 2.203608 1.536369
C -1.840294 2.621007 -1.430575
C -1.491808 1.217216 -0.936988
H 0.120452 3.168716 -2.195528
H -0.905499 4.543121 -1.823017
H -2.294236 2.565195 -2.430982
H -2.598022 3.074949 -0.779827
H 1.960394 2.026549 -1.054813
H -0.916236 0.656339 -1.682052
C -2.523466 0.380154 -0.292053
C -2.571202 -1.005811 -0.547362
H -1.859343 -1.436116 -1.247565
C -3.513020 -1.825576 0.069739
H -3.533157 -2.888622 -0.158822
C -4.435117 -1.286726 0.970547
H -5.172100 -1.923989 1.451532
C -4.398568 0.082957 1.244858
H -5.106910 0.515251 1.947286
C -3.458629 0.903155 0.625348
H -3.445898 1.963357 0.863786

44
-1301.58807586380000 TScrad15crad.TS.conf009
S -1.999386 -1.798713 0.804610
N -0.787109 -2.459841 -0.160350
C -2.266237 -0.106946 0.270248
C -3.242487 0.159973 -0.694452
H -3.845669 -0.650431 -1.089992
C -3.449846 1.473420 -1.105441
H -4.217132 1.684647 -1.846574
C -2.698001 2.530252 -0.568529
C -2.923973 3.948534 -1.035589
C -1.731993 2.234356 0.402082
H -1.147042 3.038432 0.840648
C -1.508863 0.924624 0.826315
H -0.773295 0.709354 1.593587
O -3.200872 -2.556829 0.451585
O -1.452294 -1.754548 2.160550
C 0.602990 -2.193481 0.009080
C 1.508401 -3.224328 -0.644887
H -2.469827 4.117171 -2.020904
H -2.486093 4.673557 -0.342754
H -3.992232 4.172370 -1.131249
H 1.520445 -4.150895 -0.052364
C 2.931736 -2.673481 -0.821123
H 1.102458 -3.493935 -1.632329
H 1.106229 -1.026390 -0.610476
H 0.822953 -2.000337 1.060268
C 2.959460 -1.429109 -1.737455
C 2.016504 -0.307719 -1.285157
H 3.577801 -3.452425 -1.243441
H 3.344980 -2.428258 0.166299
H 2.669024 -1.747691 -2.747151
H 3.990056 -1.057675 -1.817793
H -1.118013 -2.704361 -1.088902
H 1.401220 0.088252 -2.097951
C 2.519496 0.769897 -0.408012
C 1.881334 2.028155 -0.408386
H 1.042174 2.191326 -1.080448
C 2.308322 3.059443 0.423347
H 1.803324 4.022099 0.390363
C 3.389668 2.866812 1.288332
H 3.725736 3.671010 1.937026
C 4.036341 1.629459 1.303294
H 4.881669 1.466286 1.967245
C 3.609090 0.596836 0.469803
H 4.134841 -0.352974 0.497688

44
-1301.58754196210000 TScrad15crad.TS.conf055
S 1.243809 -1.327966 1.214425
N 0.486888 -2.171369 -0.030469
C 2.325194 -0.122289 0.441328
C 1.835192 1.147302 0.126491
H 0.816560 1.416214 0.386532
C 2.681059 2.065546 -0.494435
H 2.304584 3.057043 -0.733598
C 4.008397 1.738311 -0.803116
C 4.910072 2.730784 -1.497603
C 4.477097 0.460174 -0.463327
H 5.510025 0.195267 -0.676105
C 3.646653 -0.472327 0.152919
H 4.019336 -1.451793 0.434456
O 0.208693 -0.577382 1.927654
O 2.078002 -2.332898 1.878763
C -0.267225 -1.513489 -1.057959
C -1.042800 -2.477614 -1.933835
H 4.594307 3.761331 -1.307008
H 4.896475 2.580344 -2.585336
H 5.949449 2.626208 -1.169137
H -1.312750 -1.957452 -2.861891
C -2.326523 -2.989801 -1.243681
H -0.413350 -3.333421 -2.231416
H -1.378299 -0.874650 -0.402979
H 0.256069 -0.701480 -1.575037
C -3.268187 -1.825691 -0.882607
C -2.586411 -0.839454 0.059449
H -2.053336 -3.542827 -0.335704
H -2.839941 -3.696611 -1.906488
H -4.188374 -2.218759 -0.426291
H -3.573898 -1.328404 -1.812132
H 1.036799 -2.988772 -0.293924
H -2.387480 -1.279933 1.040814
C -3.025949 0.570261 0.164231
C -2.625204 1.325689 1.288126
H -2.024333 0.843319 2.054399
C -2.972767 2.667103 1.416426
H -2.653576 3.222748 2.294805
C -3.732928 3.298143 0.426565
H -4.009128 4.344295 0.528349
C -4.135393 2.567928 -0.692993
H -4.729596 3.045154 -1.468637
C -3.784700 1.224517 -0.825495
H -4.115732 0.680915 -1.705420

44
-1301.58730698010000 TScrad15crad.TS.conf195
S 2.276044 -1.429552 -0.802749
N 1.081785 -2.201270 0.111830
C 2.219983 0.277924 -0.265924
C 1.472482 1.205421 -0.990466
H 0.937533 0.894382 -1.881024
C 1.438070 2.532039 -0.560070
H 0.855083 3.256862 -1.122394
C 2.143825 2.945042 0.577287
C 2.131050 4.389203 1.018783
C 2.890850 1.988973 1.284021
H 3.449761 2.292106 2.166184
C 2.936163 0.661006 0.871164
H 3.529753 -0.070532 1.409223
O 3.532463 -2.017987 -0.326765
O 1.887091 -1.455464 -2.215795
C -0.248482 -2.389523 -0.390031

C -0.993713 -3.524085 0.288400
H 1.279098 4.930767 0.596191
H 3.043303 4.907138 0.694780
H 2.082105 4.474418 2.109877
H -1.841265 -3.805415 -0.349183
C -1.524584 -3.124148 1.681867
H -0.355218 -4.419520 0.366193
H -1.036153 -1.315057 0.069234
H -0.304964 -2.351916 -1.479227
C -2.490184 -1.929105 1.587719
C -1.825883 -0.709565 0.952331
H -0.681746 -2.869474 2.339562
H -2.033057 -3.980532 2.140576
H -2.865323 -1.675096 2.590060
H -3.367694 -2.235480 1.004326
H 1.501550 -2.964545 0.638495
H -1.058868 -0.279896 1.605561
C -2.636391 0.330004 0.286953
C -2.232561 1.680308 0.348554
H -1.344071 1.937427 0.920264
C -2.948922 2.682532 -0.300331
H -2.617604 3.715579 -0.225313
C -4.093006 2.367253 -1.038110
H -4.654885 3.147981 -1.543467
C -4.505934 1.034929 -1.117813
H -5.392127 0.774479 -1.691359
C -3.789799 0.032160 -0.468057
H -4.128636 -0.996535 -0.554577

44
-1301.61343683850000 TScrad15crad.TS.conf195_orc_fwd
S 3.314259 0.228063 -0.232396
N 2.798827 -1.339042 0.184779
C 1.884627 1.299102 -0.100632
C 1.056634 1.484028 -1.210673
H 1.323553 1.040138 -2.163574
C -0.091313 2.261639 -1.078225
H -0.737523 2.408669 -1.939959
C -0.429123 2.857538 0.145874
C -1.703274 3.654175 0.287000
C 0.432993 2.673968 1.236881
H 0.201529 3.149586 2.186802
C 1.584914 1.897565 1.124999
H 2.257427 1.772584 1.967542
O 4.253677 0.590434 0.829741
O 3.691743 0.159246 -1.643305
C 1.930398 -2.085761 -0.623955
C 1.340431 -3.338818 -0.051190
H -2.560770 2.984578 0.434703
H -1.906216 4.248384 -0.610413
H -1.660126 4.333835 1.143919
H 0.820444 -3.867418 -0.859590
C 0.368110 -3.174914 1.144653
H 2.154551 -4.014282 0.271045
H -0.536899 -1.097962 -0.576374
H 2.137446 -2.001073 -1.685014
C -1.050085 -2.692279 0.797497
C -1.147361 -1.228195 0.324078
H 0.806743 -2.505065 1.900224
H 0.284145 -4.153434 1.634784
H -1.687167 -2.815615 1.683807
H -1.476379 -3.350401 0.026912
H 2.716609 -1.446427 1.192109
H -0.705996 -0.575561 1.088714
C -2.570339 -0.791207 0.048292
C -3.336111 -0.158455 1.037765
H -2.889557 0.039599 2.010240
C -4.657703 0.220926 0.793753
H -5.232562 0.710552 1.575954
C -5.239333 -0.027999 -0.450812
H -6.267056 0.267708 -0.644050
C -4.489129 -0.657287 -1.446136
H -4.931143 -0.852224 -2.420016
C -3.168510 -1.032755 -1.196509
H -2.589309 -1.516954 -1.980337

44
-1301.61177363400000 TScrad15crad.TS.conf009_orc_fwd
S -2.046666 -1.761656 0.845038
N -1.116273 -2.321636 -0.468045
C -2.530828 -0.077830 0.466446
C -3.725862 0.152853 -0.220521
H -4.375405 -0.679891 -0.469748
C -4.081368 1.459460 -0.544485
H -5.017592 1.642509 -1.066341
C -3.260548 2.543705 -0.198165
C -3.643218 3.953445 -0.580399
C -2.073294 2.284127 0.499928
H -1.434132 3.112334 0.795610
C -1.701400 0.983181 0.835960
H -0.797260 0.793805 1.403839
O -3.253625 -2.588098 0.818690
O -1.129284 -1.722545 1.983721
C 0.204839 -1.903192 -0.671294
C 0.820508 -2.146705 -2.012072
H -3.316579 4.186169 -1.602705
H -3.180928 4.689087 0.085245
H -4.728309 4.097276 -0.546643
H 0.923648 -3.235461 -2.182081
C 2.209419 -1.504280 -2.206477
H 0.142624 -1.792257 -2.806394
H 0.978442 0.655126 -0.777475
H 0.814961 -1.910172 0.225112
C 2.222088 0.016832 -2.439545
C 1.954229 0.908492 -1.205984
H 2.669873 -1.982111 -3.080395
H 2.850951 -1.756512 -1.351899
H 1.482596 0.264621 -3.214629
H 3.200178 0.290156 -2.857409
H -1.689733 -2.484561 -1.291816
H 1.879313 1.947601 -1.556398
C 3.021647 0.827018 -0.130511
C 2.750590 0.279152 1.130490
H 1.753197 -0.094311 1.350261
C 3.739845 0.209389 2.114812
H 3.503371 -0.220220 3.084831
C 5.022791 0.689792 1.854199
H 5.793824 0.637647 2.618291
C 5.307530 1.243733 0.603547
H 6.302168 1.628216 0.391808
C 4.316421 1.310967 -0.374745
H 4.548297 1.753198 -1.341760

44
-1301.61086129500000 TScrad15crad.TS.conf055_orc_rev
S 1.308208 0.335980 1.219468
N 0.589185 -1.118950 0.720280
C 2.902281 0.435377 0.408238
C 2.984507 0.919175 -0.899750
H 2.095949 1.288223 -1.400068
C 4.225121 0.952050 -1.532577
H 4.294521 1.336761 -2.547098
C 5.386108 0.511327 -0.881101
C 6.721411 0.527270 -1.585794
C 5.274871 0.042135 0.435917
H 6.166899 -0.284865 0.964583
C 4.042847 -0.003395 1.084359
H 3.962740 -0.350145 2.109275
O 0.458936 1.395326 0.672323
O 1.539230 0.160376 2.653394
C 0.132277 -1.302280 -0.594816
C -0.387817 -2.647727 -0.984682
H 6.773452 1.329846 -2.328542
H 6.897642 -0.418644 -2.115124
H 7.546311 0.662676 -0.878837
H -0.469833 -2.665807 -2.079257
C -1.765967 -3.053984 -0.387537
H 0.346749 -3.424961 -0.720397
H -2.126579 -0.568109 0.736291
H -0.269887 -0.402177 -1.046049

C -2.900315 -2.044520 -0.661011
C -3.092758 -1.006671 0.468343
H -1.663664 -3.207943 0.695355
H -2.024519 -4.032911 -0.812304
H -3.845867 -2.582586 -0.804503
H -2.709730 -1.520844 -1.607975
H 0.982739 -1.924193 1.200994
H -3.448428 -1.536573 1.363473
C -4.058817 0.101611 0.106465
C -3.591269 1.396324 -0.156872
H -2.527758 1.606934 -0.063849
C -4.473540 2.416989 -0.517341
H -4.090775 3.415748 -0.711996
C -5.841073 2.159157 -0.620237
H -6.529252 2.953330 -0.898054
C -6.320784 0.874346 -0.355205
H -7.385572 0.665332 -0.424185
C -5.436320 -0.142143 0.005410
H -5.820667 -1.137996 0.218220

44
-1301.61031527510000 TScrad15crad.TS.conf020_orc_fwd
S -1.476592 -1.261235 -1.156870
N -0.688961 -1.942079 0.195898
C -2.694022 -0.183030 -0.408697
C -3.877623 -0.731365 0.092267
H -4.058393 -1.798263 0.013363
C -4.818423 0.110837 0.675545
H -5.741695 -0.310772 1.065542
C -4.599187 1.495150 0.765201
C -5.642307 2.399029 1.377434
C -3.405864 2.016139 0.250268
H -3.220362 3.085791 0.306499
C -2.451242 1.188045 -0.340783
H -1.535831 1.596156 -0.755103
O -0.542932 -0.414482 -1.906395
O -2.165752 -2.397225 -1.776680
C 0.470532 -1.348032 0.731366
C 1.243006 -2.116146 1.753871
H -6.089574 1.947086 2.269520
H -6.458622 2.592770 0.669307
H -5.217770 3.366571 1.662244
H 0.691963 -2.134408 2.713517
C 2.656305 -1.557189 2.019376
H 1.321946 -3.171893 1.446047
H 2.614787 -1.438511 -0.873644
H 0.454675 -0.263612 0.763277
C 3.715696 -1.874631 0.949493
C 3.568228 -1.160692 -0.411656
H 3.004540 -1.976600 2.972055
H 2.595403 -0.471307 2.170698
H 3.725435 -2.959382 0.769689
H 4.701667 -1.634467 1.369253
H -0.673672 -2.954757 0.090934
H 4.356332 -1.546903 -1.074558
C 3.667049 0.353817 -0.358916
C 2.644998 1.156599 -0.885175
H 1.770221 0.684933 -1.326399
C 2.742955 2.550151 -0.860711
H 1.940684 3.152406 -1.280330
C 3.864330 3.167861 -0.306699
H 3.941446 4.251930 -0.287367
C 4.891780 2.379958 0.217127
H 5.774677 2.849227 0.644352
C 4.792623 0.988933 0.188118
H 5.606813 0.389370 0.589788

44
-1301.61665231320000 TScrad15crad.C2rad.conf18
S 1.523155 -1.824933 0.956840
N 0.048561 -2.040482 0.127851
C 2.208544 -0.266812 0.396304
C 1.876305 0.911870 1.069454
H 1.249864 0.871247 1.954439
C 2.370505 2.124698 0.596642
H 2.119377 3.043068 1.121952
C 3.189646 2.182308 -0.541170
C 3.745489 3.500566 -1.024661
C 3.502497 0.984982 -1.199034
H 4.138654 1.009697 -2.080519
C 3.020022 -0.239511 -0.739788
H 3.283510 -1.165702 -1.238640
O 1.136345 -1.672318 2.360621
O 2.402732 -2.895350 0.487717
C 0.002226 -2.300203 -1.245393
C -1.206182 -1.856550 -2.005235
H 4.668821 3.758187 -0.488997
H 3.036895 4.319264 -0.859978
H 3.986256 3.467493 -2.092020
H -1.206642 -2.341617 -2.988216
C -1.296649 -0.319661 -2.183669
H -2.117216 -2.216155 -1.496241
H -4.031632 -1.054173 -1.390509
H 0.689980 -3.057810 -1.598783
C -2.690257 0.205847 -2.561230
C -3.781467 0.009535 -1.478401
H -0.572156 -0.014431 -2.948616
H -0.977300 0.173654 -1.257742
H -3.035077 -0.269236 -3.490287
H -2.603593 1.278266 -2.780711
H -0.675162 -1.431849 0.508677
H -4.696768 0.507578 -1.824824
C -3.393435 0.542706 -0.113210
C -3.111228 -0.327854 0.950231
H -3.227302 -1.400596 0.806287
C -2.704631 0.158098 2.196608
H -2.488372 -0.537266 3.003208
C -2.581959 1.533022 2.402550
H -2.270065 1.915353 3.370602
C -2.874868 2.412989 1.358000
H -2.794353 3.486076 1.512251
C -3.274649 1.921406 0.114926
H -3.500190 2.617970 -0.689966

44
-1301.61603006610000 TScrad15crad.C2rad.conf98
S 2.963887 -0.879254 0.936312
N 1.802064 -2.104947 0.648759
C 2.307814 0.638220 0.244808
C 1.591291 1.514092 1.063879
H 1.486495 1.298963 2.122148
C 1.041613 2.667141 0.507750
H 0.496040 3.358040 1.146151
C 1.189539 2.958612 -0.856748
C 0.623040 4.230462 -1.441924
C 1.908829 2.058533 -1.656380
H 2.039903 2.270870 -2.714741
C 2.472337 0.903528 -1.117457
H 3.052721 0.227019 -1.735234
O 2.997394 -0.738518 2.392811
O 4.133318 -1.260011 0.145012
C 1.539162 -2.593643 -0.629459
C 0.142766 -2.931189 -1.037158
H -0.277769 4.552182 -0.909160
H 0.368348 4.107497 -2.499624
H 1.350381 5.050445 -1.375578
H 0.185812 -3.490850 -1.979376
C -0.786427 -1.705115 -1.208124
H -0.321528 -3.614274 -0.303305
H -4.103311 -1.235270 -2.219450
H 2.408827 -2.780113 -1.245158
C -2.207852 -2.088605 -1.633752
C -3.172728 -0.886637 -1.753421
H -0.346936 -1.024042 -1.949265
H -0.824769 -1.144704 -0.265035
H -2.624445 -2.811327 -0.917868
H -2.168378 -2.602807 -2.604323
H 1.014745 -2.016632 1.286748
H -2.739087 -0.147929 -2.440257
C -3.496899 -0.224216 -0.429465

C -4.476679 -0.767572 0.414552
H -5.021500 -1.654391 0.096398
C -4.768109 -0.185702 1.648249
H -5.534979 -0.621551 2.283533
C -4.081684 0.957573 2.063381
H -4.309072 1.414471 3.022803
C -3.105722 1.510831 1.233861
H -2.567163 2.402359 1.545341
C -2.818569 0.924901 -0.000813
H -2.056996 1.365668 -0.640306

44
-1301.61557217830000 TScrad15crad.C2rad.conf06
S 1.088238 2.198430 -0.067445
N -0.426040 1.767822 0.572325
C 2.042024 0.689969 -0.240315
C 2.106588 0.055127 -1.483481
H 1.628849 0.511264 -2.344182
C 2.808369 -1.142806 -1.600694
H 2.868818 -1.632232 -2.569909
C 3.445440 -1.723241 -0.493518
C 4.229307 -3.005774 -0.638523
C 3.364516 -1.065341 0.741810
H 3.858081 -1.495899 1.609756
C 2.673329 0.137611 0.877447
H 2.640529 0.653777 1.830617
O 0.794987 2.697888 -1.412214
O 1.735854 3.013563 0.960236
C -0.578526 1.339090 1.894007
C -1.812786 0.596117 2.301252
H 4.274850 -3.557442 0.305954
H 5.263056 -2.801675 -0.947581
H 3.788474 -3.662119 -1.396199
H -2.292151 1.133253 3.133591
C -1.567786 -0.861401 2.774798
H -2.541113 0.601271 1.482674
H -2.885125 -2.841631 1.256269
H 0.048379 1.851978 2.613815
C -1.019970 -1.842758 1.723187
C -2.043036 -2.402354 0.705557
H -2.508234 -1.256021 3.186866
H -0.860478 -0.826457 3.614063
H -0.597560 -2.706223 2.254184
H -0.178743 -1.378397 1.192337
H -1.025834 1.337936 -0.131360
H -1.559797 -3.234151 0.174348
C -2.576265 -1.420629 -0.319684
C -1.727491 -0.874058 -1.296379
H -0.682556 -1.176146 -1.326446
C -2.203888 0.048645 -2.230615
H -1.527773 0.465358 -2.972688
C -3.547199 0.436152 -2.212406
H -3.918190 1.155347 -2.937120
C -4.405988 -0.114208 -1.261209
H -5.454198 0.172589 -1.242512
C -3.922509 -1.033701 -0.326419
H -4.600309 -1.454270 0.413294

44
-1301.61529783180000 TScrad15crad.C2rad.conf19
S 1.367009 -2.187300 0.056766
N -0.182319 -1.860580 0.692086
C 2.174034 -0.610591 -0.220508
C 2.097852 -0.009967 -1.480576
H 1.612169 -0.533971 -2.297205
C 2.674588 1.243179 -1.673219
H 2.626522 1.706515 -2.655910
C 3.325744 1.912734 -0.625681
C 3.975424 3.256554 -0.854783
C 3.387339 1.287697 0.627466
H 3.894539 1.787388 1.449313
C 2.820767 0.031572 0.838573
H 2.896331 -0.457409 1.803708
O 2.094395 -2.873544 1.124631
O 1.109826 -2.794524 -1.251083
C -0.397719 -1.395571 1.986927
C -1.419853 -0.335311 2.228606
H 4.996826 3.138732 -1.240375
H 4.042228 3.833693 0.073038
H 3.419805 3.851446 -1.587412
H -2.279160 -0.499647 1.564228
C -0.906367 1.120363 2.029612
H -1.806492 -0.432964 3.251190
H -1.571537 2.956880 -0.071455
H 0.257534 -1.797631 2.748283
C -2.037833 2.160818 1.883253
C -2.430380 2.481207 0.419224
H -0.256715 1.158133 1.146220
H -0.266254 1.378281 2.882106
H -1.735003 3.105927 2.351817
H -2.925557 1.820933 2.434691
H -0.823312 -1.535477 -0.030859
H -3.233528 3.229828 0.433855
C -2.869287 1.286672 -0.403200
C -1.991492 0.666979 -1.304806
H -0.994968 1.079646 -1.447714
C -2.377182 -0.469451 -2.022945
H -1.680250 -0.935205 -2.715427
C -3.657846 -1.001321 -1.856062
H -3.960095 -1.883987 -2.412571
C -4.549211 -0.382596 -0.976816
H -5.552203 -0.781346 -0.849033
C -4.157074 0.749239 -0.260761
H -4.859598 1.222416 0.422449

44
-1301.61504295140000 TScrad15crad.C2rad.conf60
S -1.089103 -1.167158 -1.507536
N 0.219964 -1.518419 -0.468617
C -2.250017 -0.172720 -0.572071
C -2.099588 1.216801 -0.544996
H -1.320039 1.688048 -1.134779
C -2.974700 1.978465 0.224364
H -2.868797 3.060663 0.236577
C -3.996120 1.376649 0.975560
C -4.920483 2.212568 1.828315
C -4.126806 -0.017900 0.925269
H -4.923682 -0.500698 1.485817
C -3.262510 -0.798114 0.158773
H -3.384001 -1.874471 0.101668
O -0.510860 -0.321319 -2.554446
O -1.728428 -2.452701 -1.789433
C 0.179448 -2.479153 0.536357
C 0.626266 -2.197909 1.941935
H -5.136352 3.177720 1.357827
H -4.470739 2.422944 2.807886
H -5.871535 1.701635 2.008795
H -0.219757 -1.812151 2.542142
C 1.793408 -1.201599 2.075934
H 0.899430 -3.156304 2.404602
H 5.014328 -0.925743 0.718318
H -0.413013 -3.354299 0.299270
C 3.087683 -1.647133 1.376266
C 4.109438 -0.506289 1.179238
H 1.989675 -1.032524 3.142766
H 1.478804 -0.225738 1.682343
H 2.844239 -2.082420 0.398973
H 3.558030 -2.454635 1.953006
H 0.839533 -0.717553 -0.366421
H 4.416175 -0.113608 2.157287
C 3.583347 0.634646 0.326698
C 3.144971 0.407575 -0.988814
H 3.218412 -0.589410 -1.417788
C 2.614459 1.442373 -1.761790
H 2.261780 1.234198 -2.767698
C 2.529222 2.734619 -1.237174
H 2.120737 3.542418 -1.838412
C 2.975297 2.979174 0.061923
H 2.917031 3.981305 0.479325
C 3.490775 1.936447 0.835688

H 3.825396 2.135259 1.851586

44
-1301.61472285540000 TScrad15crad.C2rad.conf21
S 1.528471 1.826182 0.951418
N 0.053927 2.042519 0.122390
C 2.209012 0.264505 0.395076
C 1.872591 -0.912078 1.071748
H 1.247698 -0.866434 1.957588
C 2.365757 -2.126548 0.605026
H 2.115563 -3.042095 1.135992
C 3.186074 -2.189901 -0.532994
C 3.705008 -3.518400 -1.029552
C 3.506727 -0.995707 -1.190489
H 4.150293 -1.023917 -2.066283
C 3.024660 0.232148 -0.736728
H 3.296430 1.156520 -1.234509
O 2.410235 2.893080 0.478466
O 1.141878 1.678986 2.355843
C 0.007487 2.298221 -1.251514
C -1.201823 1.854367 -2.009761
H 4.413406 -3.389888 -1.853407
H 2.886289 -4.154368 -1.389566
H 4.213302 -4.071279 -0.230707
H -2.112120 2.216508 -1.501272
C -1.294571 0.317142 -2.184301
H -1.202037 2.336960 -2.993955
H -4.695693 -0.504307 -1.821570
H 0.697561 3.052290 -1.607872
C -2.689144 -0.207310 -2.559787
C -3.779499 -0.006697 -1.476890
H -0.975413 -0.174248 -1.257280
H -0.570934 0.009002 -2.948897
H -2.604173 -1.280411 -2.776621
H -3.033771 0.265917 -3.489859
H -0.671107 1.436875 0.505468
H -4.028109 1.057583 -1.391575
C -3.391526 -0.536957 -0.110548
C -3.107514 0.335878 0.950542
H -3.222140 1.408418 0.803951
C -2.701014 -0.147512 2.197948
H -2.483381 0.549570 3.002694
C -2.580233 -1.522089 2.407310
H -2.268440 -1.902427 3.376178
C -2.874945 -2.404273 1.365138
H -2.795944 -3.477083 1.522085
C -3.274637 -1.915250 0.121025
H -3.501668 -2.613512 -0.681981

N-rad,N-rad-1,2'-HAT self reaction

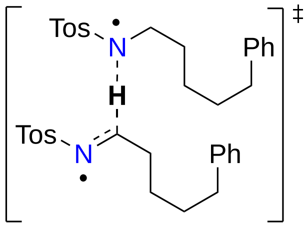

88

S -0.567121 1.367690 0.965393
N 0.582736 1.152233 -0.246699
C -0.976148 3.110785 0.816718
C -0.565374 3.998745 1.809338
H -0.009853 3.627161 2.663625
C -0.889107 5.351133 1.690059
H -0.571846 6.045073 2.464645
C -1.618131 5.828302 0.594545
C -1.984960 7.288556 0.477396
C -2.021057 4.910630 -0.389877
H -2.590918 5.262341 -1.246899
C -1.708270 3.559477 -0.286624
H -2.032936 2.855670 -1.045782
O -0.008258 1.171959 2.314976
O -1.754021 0.596219 0.547524
C 1.981785 0.994352 0.185265
H 2.419520 0.228485 -0.463274
C 2.799702 2.290979 0.050761
H -3.052063 7.445416 0.681874
H -1.791716 7.669901 -0.531807
H -1.419728 7.902254 1.185464
H 2.048907 0.623228 1.213369
H 3.822237 2.049870 0.368284
H 2.420014 3.028164 0.770179
S -0.126646 -3.316212 -0.235551
N -0.801837 -1.982879 -1.039282
C 0.388714 -2.746912 1.378540
C -0.575589 -2.538580 2.369523
H -1.622356 -2.740686 2.167937
C -0.171557 -2.066715 3.612784
H -0.917882 -1.891143 4.383015
C 1.178306 -1.798662 3.885424
C 1.589882 -1.227932 5.219766
C 2.123866 -2.037906 2.880503
H 3.176387 -1.849370 3.077020
C 1.740551 -2.505177 1.624018
H 2.476351 -2.685671 0.847977
O -1.251303 -4.235699 -0.055584
O 1.069432 -3.677315 -1.000584
C -0.026961 -0.993294 -1.691801
H 0.969498 -1.389427 -1.903281
C -0.673724 -0.338088 -2.921262
H 2.655009 -1.386582 5.415986
H 1.019834 -1.673942 6.042043
H 1.403851 -0.146414 5.242249
H -0.164705 0.622072 -3.067761
H -0.430589 -0.955965 -3.799094
H 0.203011 -0.009884 -0.915412
C -2.200161 -0.130186 -2.852836
H -2.492579 0.127477 -1.827589
H -2.467155 0.740295 -3.467146
C -2.998259 -1.347955 -3.354971
H -2.904632 -1.407487 -4.448180
H -2.551714 -2.269546 -2.964352
C -4.494100 -1.317466 -2.981004
H -4.946391 -0.386551 -3.348048
H -4.999550 -2.135082 -3.513387
C 2.809213 2.937121 -1.343526
H 1.792779 3.280130 -1.572105
H 3.433369 3.841133 -1.292848
C 3.290921 2.080161 -2.529320
H 2.614367 1.228258 -2.675843
H 3.186266 2.696690 -3.431889
C 4.753101 1.569870 -2.474509
H 5.088238 1.405046 -3.508000
H 5.398905 2.361339 -2.072037
C 4.982307 0.284763 -1.698666
C 4.367846 -0.910937 -2.104685
H 3.727627 -0.911334 -2.984718
C 4.568979 -2.102540 -1.406575
H 4.069584 -3.010661 -1.733305
C 5.406617 -2.123428 -0.287516
H 5.572449 -3.051269 0.253564
C 6.037364 -0.947176 0.119965
H 6.698533 -0.953360 0.982944
C 5.825290 0.242790 -0.580622
H 6.324013 1.153660 -0.255881
C -4.755773 -1.451516 -1.491673
C -5.390666 -0.432873 -0.770265
H -5.704743 0.470314 -1.289244
C -5.624615 -0.556995 0.600850
H -6.115260 0.249853 1.139065
C -5.223889 -1.708235 1.278321
H -5.403697 -1.805988 2.345661
C -4.588275 -2.733770 0.573818
H -4.270825 -3.638895 1.084937
C -4.359162 -2.606059 -0.797117
H -3.864456 -3.416587 -1.326673

88

S 2.954943 1.223435 1.176356
N 1.811240 0.698443 0.052226
C 4.530201 0.751041 0.461769
C 5.361314 -0.125676 1.154735
H 5.033975 -0.539155 2.102377
C 6.606590 -0.452017 0.613786
H 7.257519 -1.135973 1.152431
C 7.031903 0.088128 -0.604739
C 8.376342 -0.269074 -1.192144
C 6.175657 0.976920 -1.277097
H 6.491757 1.412431 -2.222084
C 4.933107 1.314297 -0.752946
H 4.282371 2.008327 -1.274725
O 2.822927 0.487603 2.443820
O 2.860185 2.690941 1.149600
C 1.178479 -0.630490 0.230618
H 0.212182 -0.562937 -0.278479
C 2.011917 -1.779533 -0.347373
H 8.978640 0.627769 -1.380431
H 8.264296 -0.788140 -2.152236
H 8.945373 -0.921993 -0.523760
H 0.967688 -0.819288 1.288918
H 1.490401 -2.712738 -0.109278
H 2.969761 -1.822122 0.186639
S -4.468201 -0.467077 0.966053
N -3.831240 0.177545 -0.509738
C -3.025022 -0.375282 2.011485
C -2.331723 0.833541 2.140611
H -2.620355 1.700131 1.555267
C -1.258383 0.902204 3.022264
H -0.709630 1.835669 3.119122
C -0.872971 -0.208930 3.793535

C 0.286255 -0.105758 4.754004
C -1.587790 -1.403489 3.640093
H -1.303332 -2.274598 4.224641
C -2.660175 -1.497941 2.752522
H -3.211554 -2.424300 2.635971
O -5.470226 0.505246 1.414393
O -4.845863 -1.884237 0.808010
C -4.146785 -0.468517 -1.566317
H -4.722198 -1.401921 -1.514030
C -3.738578 -0.005431 -2.935988
H 0.430207 -1.039075 5.307034
H 0.120034 0.695915 5.484086
H 1.214846 0.125763 4.220063
H -3.157813 -0.828071 -3.382647
H -4.653900 0.048047 -3.546519
H 1.142932 1.461239 -0.056002
C -2.953236 1.310266 -3.004923
H -2.129366 1.272712 -2.283418
H -2.496943 1.384940 -4.001443
C -3.806988 2.559779 -2.748621
H -4.598268 2.618261 -3.509578
H -4.306401 2.469784 -1.777737
C -2.998087 3.875533 -2.781885
H -2.486049 3.961458 -3.749656
H -3.704471 4.714219 -2.729736
C 2.295485 -1.685054 -1.856917
H 2.772287 -0.716605 -2.051357
H 3.041430 -2.448051 -2.120926
C 1.089793 -1.832645 -2.805353
H 0.258938 -1.197320 -2.469236
H 1.384176 -1.434818 -3.785864
C 0.577738 -3.273000 -3.039305
H -0.097818 -3.248036 -3.906471
H 1.426434 -3.902423 -3.337747
C -0.150792 -3.934573 -1.884456
C -1.366845 -3.418136 -1.411956
H -1.780800 -2.523760 -1.873949
C -2.058350 -4.029997 -0.366172
H -2.998840 -3.609203 -0.019762
C -1.540011 -5.183372 0.228888
H -2.076531 -5.666504 1.041170
C -0.334258 -5.713531 -0.230689
H 0.074858 -6.612786 0.223044
C 0.350796 -5.093893 -1.278687
H 1.287944 -5.517317 -1.634029
C -1.985886 4.003204 -1.660708
C -0.617039 3.812248 -1.888275
H -0.270314 3.596395 -2.896907
C 0.308010 3.904896 -0.844124
H 1.368457 3.766964 -1.037028
C -0.122833 4.193584 0.452707
H 0.603133 4.260809 1.257146
C -1.485084 4.398710 0.691382
H -1.832364 4.644065 1.692130
C -2.403698 4.302790 -0.354692
H -3.461062 4.467360 -0.157725

88

S 3.596354 -0.819016 1.006879
N 2.381769 -1.059231 -0.149070
C 4.743335 0.224062 0.117586
C 5.592973 -0.346025 -0.833510
H 5.556804 -1.412960 -1.027103
C 6.487039 0.471988 -1.517082
H 7.147919 0.033887 -2.261088
C 6.555416 1.850338 -1.258481
C 7.550808 2.718868 -1.989871
C 5.698338 2.391256 -0.291785
H 5.742899 3.455131 -0.072513
C 4.792079 1.587755 0.400860
H 4.137128 2.004230 1.158473
O 4.245700 -2.115711 1.262906
O 3.040671 -0.042593 2.129399
C 1.313250 -1.935450 0.308672
H 0.383378 -1.500755 -0.076122
C 1.490512 -3.371777 -0.266143
H 8.565193 2.570438 -1.597348
H 7.309454 3.781096 -1.886009
H 7.580644 2.479399 -3.058624
H 1.240456 -1.994708 1.400940
H 0.688036 -3.972714 0.172803
H 2.438460 -3.763619 0.117579
S -1.856917 1.196079 -2.007260
N -0.979762 2.509872 -1.378628
C -3.527116 1.630620 -1.555437
C -4.344360 2.259611 -2.493887
H -3.964128 2.470375 -3.487634
C -5.650293 2.590258 -2.136375
H -6.295755 3.073522 -2.865430
C -6.146618 2.304722 -0.857228
C -7.552789 2.692608 -0.467365
C -5.301036 1.664018 0.061411
H -5.674660 1.422572 1.053516
C -3.994408 1.321520 -0.275793
H -3.351114 0.817068 0.438079
O -1.726190 1.248266 -3.471679
O -1.489394 -0.030760 -1.274751
C 0.459056 2.312195 -1.417409
H 0.767928 2.486935 -2.463956
C 1.197015 3.289732 -0.493995
H -8.002314 1.950917 0.201268
H -8.198496 2.796649 -1.344886
H -7.561347 3.654493 0.062200
H 2.263827 3.191667 -0.733269
H 0.907706 4.317471 -0.749733
H 0.736767 1.272305 -1.188930
C 0.993190 3.031588 1.010751
H 1.071846 1.954342 1.204987
H 1.826615 3.495871 1.557825
C -0.319778 3.581703 1.585595
H -0.322927 4.674611 1.466969
H -1.173880 3.211419 1.005956
C -0.535610 3.245849 3.080530
H 0.377408 3.492943 3.639030
H -1.324457 3.901470 3.470670
C 1.474566 -3.477025 -1.800005
H 2.271067 -2.836899 -2.199180
H 1.751604 -4.507364 -2.067154
C 0.158631 -3.117180 -2.516367
H -0.220385 -2.150688 -2.164523
H 0.389320 -2.970094 -3.579966
C -0.965946 -4.175831 -2.446308
H -1.749459 -3.868744 -3.153551
H -0.576629 -5.131518 -2.822668
C -1.606079 -4.404242 -1.089178
C -2.262932 -3.361094 -0.416581
H -2.301898 -2.371258 -0.863995
C -2.857095 -3.575803 0.827219
H -3.351431 -2.750621 1.333790
C -2.815450 -4.839309 1.422361
H -3.280571 -5.005968 2.390655
C -2.173685 -5.886568 0.761026
H -2.135862 -6.875574 1.211048
C -1.576405 -5.667255 -0.482608
H -1.077463 -6.489810 -0.991032
C -0.922983 1.804018 3.343063
C 0.040957 0.815644 3.584689
H 1.094837 1.078181 3.600796
C -0.331208 -0.513829 3.796239
H 0.437692 -1.259092 3.981516
C -1.677538 -0.880277 3.776193
H -1.966646 -1.914309 3.943575
C -2.650347 0.095252 3.544406
H -3.704037 -0.174821 3.545060
C -2.273367 1.422169 3.329909
H -3.037593 2.178749 3.160378

S11. EPR calculations of PBN adducts

All calculations were performed on B3LYP/6-31G(d) optimized geometries with properties calculated with mixed basis set: EPR-III for C, H, O atoms, def2-QZVP for S-atom, and 6-31G(d) for N-atom (see above). Additional options: ultrafine grid, tight SCF convergence, PCM solvation using toluene, and empirical dispersion D3-BJ were used in these calculations. Only the lowest 10 structures are shown here. For more details, please consult experimental repository for this article on Zenodo.

N-PBN

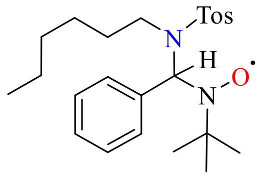

Name	E(B3LYP)	H(B3LYP)	g-factor	α_N	$\alpha_{N'}$	α_H
Tosyl_N_radical_pentane_Ph_PBN_adduct_0264_nmr_three_basis_sets	-1861.453295	-1859.967290	2.00611310	13.889680	1.478330	3.005310
Tosyl_N_radical_pentane_Ph_PBN_adduct_0273_nmr_three_basis_sets	-1861.453295	-1859.967290	2.00611333	13.889970	1.477920	3.003300
Tosyl_N_radical_pentane_Ph_PBN_adduct_0785_nmr_three_basis_sets	-1861.452967	-1859.966859	2.00611337	13.865610	1.505300	2.974810
Tosyl_N_radical_pentane_Ph_PBN_adduct_0816_nmr_three_basis_sets	-1861.452969	-1859.966859	2.00611303	13.866490	1.504870	2.977920
Tosyl_N_radical_pentane_Ph_PBN_adduct_0919_nmr_three_basis_sets	-1861.452968	-1859.966859	2.00611320	13.865680	1.505380	2.976010
Tosyl_N_radical_pentane_Ph_PBN_adduct_0788_nmr_three_basis_sets	-1861.452969	-1859.966858	2.00611310	13.865580	1.505410	2.976410
Tosyl_N_radical_pentane_Ph_PBN_adduct_0789_nmr_three_basis_sets	-1861.452972	-1859.966858	2.00611313	13.866540	1.505610	2.975180
Tosyl_N_radical_pentane_Ph_PBN_adduct_0808_nmr_three_basis_sets	-1861.452962	-1859.966854	2.00611103	13.858490	1.499020	3.001780
Tosyl_N_radical_pentane_Ph_PBN_adduct_0376_nmr_three_basis_sets	-1861.453088	-1859.966845	2.00611320	13.887990	1.481610	2.999420
Tosyl_N_radical_pentane_Ph_PBN_adduct_0371_nmr_three_basis_sets	-1861.453090	-1859.966843	2.00611333	13.890090	1.482130	2.996040
Boltzman averaged for 298.15 K			2.0061220880	13.8207426884	2.8515973977	1.5391163007

72
-1859.966368
C -1.267193 1.656567 -0.388616
C 0.370638 3.041598 -1.699631
H -0.246719 2.846223 -2.573489
C 1.521713 3.818697 -1.822221
H 1.791406 4.234516 -2.789287
C 2.321647 4.064221 -0.703720
H 3.218495 4.670697 -0.796736
C 1.960376 3.531489 0.534312
H 2.576251 3.720411 1.409444
C 0.806508 2.754609 0.659167
H 0.523455 2.348233 1.624599
C 0.005328 2.498243 -0.460011
N -1.953769 1.795975 0.923168
C -3.014010 2.818952 1.233725
C -3.472808 3.548726 -0.035988
C -2.422991 3.818548 2.245624
C -4.197790 2.059109 1.859375
H -3.900791 2.858916 -0.770127
H -2.658446 4.112974 -0.503482
H -4.250764 4.267990 0.240828
H -3.192908 4.531637 2.559892
H -1.593048 4.380209 1.804008
H -2.054707 3.288382 3.127820
H -3.868187 1.503073 2.740561
H -4.628684 1.355170 1.139588
H -4.977458 2.766638 2.161567
O -1.498970 1.104181 1.906534
C 0.149991 -0.503984 -0.268417
N -1.033196 0.255014 -0.727077
S -2.193886 -0.553544 -1.635546
O -3.252217 0.430718 -1.914354
O -1.543072 -1.248386 -2.752684
C -2.849643 -1.841023 -0.560891
C -2.998703 -1.644830 0.813260
C -3.270135 -3.028771 -1.165257
C -3.583680 -2.652945 1.578895
H -2.630907 -0.741226 1.288803
C -3.860197 -4.018673 -0.383318
H -3.121972 -3.174345 -2.229744
C -4.032183 -3.846574 0.997487
H -4.185087 -4.944027 -0.852954
C 1.303848 -0.580112 -1.275695
H 0.931632 -1.019367 -2.207711
H 1.647193 0.433329 -1.513687
C 2.474086 -1.408023 -0.728009
H 2.121833 -2.421011 -0.479350
H 2.831004 -0.967784 0.213162
C 3.640885 -1.512928 -1.717568
C 4.830302 -2.358093 -1.210029
H 5.541357 -2.484858 -2.037240
H -3.686410 -2.508540 2.651703
C -4.696741 -4.914433 1.833170
H -5.786551 -4.779970 1.847787
H -4.498988 -5.915780 1.436553
H -4.351294 -4.884332 2.871605
H -1.961559 2.033415 -1.132650
H 3.997425 -0.504083 -1.968296
C 5.554385 -1.762359 -0.019258
C 5.370468 -2.266788 1.274754
C 6.423848 -0.674087 -0.186655
C 6.028750 -1.701932 2.369349
H 4.706616 -3.115368 1.425460
C 7.084273 -0.105864 0.902248
H 6.588781 -0.272477 -1.184633
C 6.888127 -0.618244 2.186878
H 5.871662 -2.112212 3.363762

H 7.757229 0.733924 0.747662
H 7.404635 -0.179122 3.036343
H 0.469405 -0.046745 0.670112
H -0.182556 -1.516094 -0.008670
H 4.470893 -3.363575 -0.954463
H 3.278169 -1.953742 -2.656385
 72
-1859.966368
C -1.267299 1.656617 -0.388539
C 0.370548 3.041758 -1.699419
H -0.246715 2.846310 -2.573328
C 1.521561 3.818961 -1.821921
H 1.791300 4.234788 -2.788970
C 2.321372 4.064581 -0.703351
H 3.218165 4.671149 -0.796297
C 1.960040 3.531837 0.534656
H 2.575815 3.720833 1.409843
C 0.806234 2.754846 0.659423
H 0.523131 2.348466 1.624839
C 0.005178 2.498387 -0.459819
N -1.953941 1.795891 0.923207
C -3.014418 2.818642 1.233738
C -2.423699 3.818231 2.245823
C -4.198145 2.058522 1.859141
C -3.473161 3.548478 -0.035962
H -1.593773 4.380055 1.804381
H -2.055472 3.288040 3.128027
H -3.193773 4.531177 2.560033
H -4.977965 2.765881 2.161341
H -3.868553 1.502427 2.740296
H -4.628841 1.354612 1.139209
H -3.900910 2.858667 -0.770237
H -2.658826 4.112926 -0.503266
H -4.251290 4.267571 0.240815
O -1.499085 1.104124 1.906573
C 0.150082 -0.503823 -0.268516
N -1.033168 0.255110 -0.727124
S -2.193878 -0.553524 -1.635510
O -3.252329 0.430649 -1.914160
O -1.543087 -1.248228 -2.752746
C -2.849407 -1.841158 -0.560887
C -2.998423 -1.645077 0.813280
C -3.269782 -3.028924 -1.165315
C -3.583218 -2.653333 1.578881
H -2.630763 -0.741440 1.288863
C -3.859657 -4.018959 -0.383412
H -3.121690 -3.174392 -2.229826
C -4.031561 -3.846992 0.997423
H -4.184476 -4.944312 -0.853105
C 1.303888 -0.579846 -1.275858
H 0.931642 -1.019071 -2.207878
H 1.647164 0.433627 -1.513819
C 2.474196 -1.407725 -0.728278
H 2.122024 -2.420766 -0.479717
H 2.831099 -0.967562 0.212936
C 3.640998 -1.512437 -1.717856
C 4.830431 -2.357610 -1.210390
H 5.541548 -2.484224 -2.037576
H -3.685943 -2.509002 2.651698
C -4.695753 -4.915108 1.833078
H -5.785766 -4.782200 1.846130
H -4.496047 -5.916498 1.437523
H -4.351694 -4.883711 2.871931
H -1.961637 2.033496 -1.132583
H 3.997477 -0.503539 -1.968454
C 5.554409 -1.762042 -0.019465
C 5.371182 -2.267345 1.274298
C 6.423110 -0.673094 -0.186466
C 6.029399 -1.702702 2.369046
H 4.707907 -3.116432 1.424693
C 7.083465 -0.105082 0.902587
H 6.587508 -0.270785 -1.184251
C 6.888014 -0.618346 2.186973
H 5.872847 -2.113664 3.363262
H 7.755822 0.735244 0.748310
H 7.404469 -0.179379 3.036552
H 0.469515 -0.046581 0.670005
H -0.182379 -1.515962 -0.008771
H 4.471060 -3.363148 -0.954998
H 3.278314 -1.953145 -2.656735
 72
-1859.966368
C -1.267352 1.656567 -0.388613
C 0.370558 3.041755 -1.699383
H -0.246617 2.846265 -2.573345
C 1.521545 3.819016 -1.821798
H 1.791338 4.234848 -2.788830
C 2.321251 4.064685 -0.703169
H 3.218016 4.671304 -0.796045
C 1.959852 3.531918 0.534812
H 2.575554 3.720945 1.410042
C 0.806079 2.754869 0.659489
H 0.522936 2.348458 1.624880
C 0.005112 2.498376 -0.459816
N -1.954145 1.795940 0.923052
C -3.014514 2.818837 1.233450
C -2.423798 3.818427 2.245524
C -4.198386 2.058853 1.858769
C -3.473086 3.548655 -0.036318
H -1.593761 4.380129 1.804136
H -2.055723 3.288282 3.127821
H -3.193821 4.531490 2.559601
H -4.978206 2.766286 2.160791
H -3.868945 1.502820 2.740017
H -4.629001 1.354899 1.138831
H -3.900882 2.858869 -0.770586
H -2.658645 4.112967 -0.503607
H -4.251126 4.267874 0.240378
O -1.499455 1.104179 1.906497
C 0.150071 -0.503830 -0.268405
N -1.033191 0.255056 -0.727041
S -2.193842 -0.553673 -1.635460
O -3.252342 0.430436 -1.914145
O -1.542995 -1.248345 -2.752686
C -2.849299 -1.841341 -0.560834
C -2.998631 -1.645122 0.813284
C -3.269315 -3.029259 -1.165200
C -3.583391 -2.653390 1.578885
H -2.631217 -0.741374 1.288841
C -3.859165 -4.019315 -0.383296
H -3.120956 -3.174822 -2.229660
C -4.031415 -3.847197 0.997475
H -4.183675 -4.944803 -0.852932
C 1.303927 -0.579754 -1.275685
H 0.931683 -1.018795 -2.207794
H 1.647301 0.433728 -1.513457
C 2.474163 -1.407807 -0.728199
H 2.121921 -2.420886 -0.479896
H 2.831005 -0.967861 0.213138
C 3.641025 -1.512348 -1.717722
C 4.830509 -2.357531 -1.210351
H 5.541587 -2.484035 -2.037582
H -3.686328 -2.508964 2.651669
C -4.695761 -4.915235 1.833098
H -4.497098 -5.916579 1.436924
H -4.350986 -4.884564 2.871736
H -5.785671 -4.781500 1.846978
H -1.961619 2.033406 -1.132750
H 3.997487 -0.503407 -1.968171
C 5.554518 -1.762001 -0.019436
C 5.370532 -2.266651 1.274481
C 6.423973 -0.673686 -0.186596
C 6.028733 -1.701965 2.369212
H 4.706693 -3.115274 1.425004
C 7.084319 -0.105631 0.902444
H 6.588958 -0.271909 -1.184498
C 6.888099 -0.618228 2.186976
H 5.871589 -2.112414 3.363546
H 7.757269 0.734195 0.748042
H 7.404547 -0.179238 3.036547

H 0.469413 -0.046640 0.670175
H -0.182357 -1.516006 -0.008755
H 4.471187 -3.363106 -0.955028
H 3.278394 -1.952952 -2.656671
72
-1859.966357
C -1.268447 1.656379 -0.387176
C 0.368033 3.042733 -1.698512
H -0.249991 2.847876 -2.572016
C 1.518877 3.820121 -1.821470
H 1.787717 4.236690 -2.788452
C 2.319674 4.064975 -0.703439
H 3.216334 4.671686 -0.796742
C 1.959491 3.531297 0.534509
H 2.576032 3.719715 1.409283
C 0.805860 2.754127 0.659733
H 0.523616 2.347063 1.625115
C 0.003831 2.498410 -0.458990
N -1.954163 1.794973 0.925161
C -3.014218 2.817715 1.237013
C -4.197252 2.057588 1.863759
C -3.474467 3.547638 -0.032106
C -2.422400 3.817264 2.248499
H -4.976759 2.764954 2.166737
H -3.866699 1.501553 2.744589
H -4.628746 1.353614 1.144362
H -3.902800 2.857850 -0.766066
H -2.660762 4.112352 -0.500179
H -4.252470 4.266507 0.245597
H -2.053126 3.287018 3.130238
H -3.192185 4.530100 2.563674
H -1.593034 4.379224 1.806186
O -1.498476 1.102803 1.907860
C 0.149229 -0.503972 -0.268965
N -1.034403 0.255050 -0.726502
S -2.195016 -0.552937 -1.635648
O -3.252752 0.431782 -1.914997
O -1.543931 -1.247986 -2.752515
C -2.850742 -1.840789 -0.561430
C -3.281041 -3.024079 -1.168035
C -2.997921 -1.645936 0.812964
C -3.876379 -4.011661 -0.387483
H -3.142845 -3.165035 -2.234500
C -3.588311 -2.652029 1.577382
H -2.631133 -0.742065 1.288754
C -4.039350 -3.844044 0.995129
H -3.696490 -2.505700 2.649397
C 1.302852 -0.578632 -1.276622
H 0.930601 -1.017235 -2.208926
H 1.645666 0.435178 -1.513786
C 2.473625 -1.406478 -0.729975
H 2.121969 -2.419936 -0.482361
H 2.830428 -0.967061 0.211626
C 3.640358 -1.509674 -1.719796
C 4.830295 -2.354731 -1.213311
H 5.541365 -2.480163 -2.040715
H -4.215859 -4.930318 -0.860029
C -4.653879 -4.935763 1.838378
H -5.104071 -4.532174 2.750940
H -5.428622 -5.479405 1.287419
H -3.897148 -5.670399 2.144410
H -1.963385 2.033414 -1.130580
H 3.996309 -0.500357 -1.969449
C 5.554117 -1.759939 -0.021904
C 5.370889 -2.266205 1.271484
C 6.422635 -0.670715 -0.188056
C 6.028921 -1.702229 2.366690
H 4.707774 -3.115536 1.421218
C 7.082804 -0.103362 0.901454
H 6.587025 -0.267662 -1.185542
C 6.887350 -0.617583 2.185460
H 5.872379 -2.113936 3.360600
H 7.755023 0.737193 0.747831
H 7.403662 -0.179134 3.035392
H 0.468703 -0.047564 0.669949
H -0.182872 -1.516469 -0.010194
H 4.471488 -3.360714 -0.958861
H 3.277772 -1.949643 -2.659061
72
-1859.966356
C -1.268481 1.656359 -0.387243
C 0.367973 3.042632 -1.698636
H -0.250079 2.847742 -2.572111
C 1.518813 3.820013 -1.821657
H 1.787629 4.236528 -2.788667
C 2.319631 4.064922 -0.703655
H 3.216293 4.671622 -0.797005
C 1.959472 3.531305 0.534321
H 2.576033 3.719761 1.409072
C 0.805846 2.754139 0.659609
H 0.523645 2.347092 1.625009
C 0.003791 2.498371 -0.459080
N -1.954212 1.794889 0.925071
C -3.014144 2.817799 1.236985
C -2.421892 3.817580 2.247984
C -4.196961 2.057879 1.864359
C -3.474771 3.547341 -0.032197
H -3.191464 4.530639 2.563165
H -1.592571 4.379266 1.805238
H -2.052427 3.287559 3.129779
H -3.866041 1.501915 2.745095
H -4.628892 1.353878 1.145255
H -4.976231 2.765374 2.167641
H -2.661236 4.111988 -0.500651
H -4.252757 4.266226 0.245512
H -3.903261 2.857325 -0.765851
O -1.498438 1.102824 1.907801
C 0.149157 -0.503995 -0.268883
N -1.034410 0.255018 -0.726605
S -2.194992 -0.552980 -1.635686
O -3.252743 0.431716 -1.915082
O -1.543943 -1.248093 -2.752523
C -2.850746 -1.840772 -0.561424
C -2.998225 -1.645720 0.812911
C -3.280784 -3.024208 -1.167925
C -3.588679 -2.651736 1.577368
H -2.631567 -0.741760 1.288625
C -3.876191 -4.011719 -0.387336
H -3.142337 -3.165311 -2.234336
C -4.039496 -3.843881 0.995205
H -4.215445 -4.930507 -0.859785
C 1.302809 -0.578896 -1.276486
H 0.930561 -1.017645 -2.208723
H 1.645667 0.434860 -1.513822
C 2.473536 -1.406684 -0.729662
H 2.121809 -2.420037 -0.481727
H 2.830433 -0.967012 0.211784
C 3.640203 -1.510278 -1.719517
C 4.830115 -2.355243 -1.212818
H 5.541099 -2.481042 -2.040239
H -3.697041 -2.505269 2.649346
C -4.654241 -4.935446 1.838502
H -3.897355 -5.669241 2.146130
H -5.105975 -4.531482 2.750141
H -5.427849 -5.480094 1.286946
H -1.963434 2.033398 -1.130633
H 3.996193 -0.501068 -1.969543
C 5.554102 -1.760077 -0.021703
C 6.422597 -0.670909 -0.188315
C 5.371074 -2.265956 1.271865
C 7.082940 -0.103237 0.900923
H 6.586835 -0.268152 -1.185944
C 6.029279 -1.701659 2.366796
H 4.707974 -3.115236 1.421954
C 6.887686 -0.617073 2.185108
H 7.755136 0.737271 0.746943
H 5.872887 -2.113065 3.360854
H 7.404134 -0.178375 3.034828
H 0.468622 -0.047420 0.669953
H -0.183009 -1.516423 -0.009926

H 4.471241 -3.361097 -0.957960
H 3.277533 -1.950555 -2.658602
72
-1859.966356
C -1.268447 1.656348 -0.387303
C 0.368200 3.042452 -1.698637
H -0.249743 2.847470 -2.572170
C 1.519063 3.819806 -1.821598
H 1.788006 4.236216 -2.788619
C 2.319743 4.064831 -0.703521
H 3.216420 4.671514 -0.796827
C 1.959423 3.531353 0.534467
H 2.575870 3.719900 1.409278
C 0.805772 2.754209 0.659694
H 0.523443 2.347278 1.625105
C 0.003856 2.498328 -0.459068
N -1.954286 1.794955 0.924938
C -3.014251 2.817864 1.236695
C -2.422118 3.817663 2.247754
C -4.197162 2.057981 1.863936
C -3.474718 3.547388 -0.032558
H -1.592740 4.379337 1.805100
H -2.052762 3.287644 3.129596
H -3.191728 4.530725 2.562837
H -4.976472 2.765499 2.167063
H -3.866384 1.502059 2.744752
H -4.628995 1.353943 1.144809
H -3.903101 2.857356 -0.766259
H -2.661124 4.112036 -0.500909
H -4.252742 4.266275 0.245039
O -1.498616 1.102918 1.907739
C 0.149158 -0.504026 -0.268772
N -1.034370 0.254988 -0.726603
S -2.194922 -0.553049 -1.635680
O -3.252650 0.431645 -1.915189
O -1.543816 -1.248237 -2.752440
C -2.850747 -1.840769 -0.561377
C -2.998371 -1.645601 0.812929
C -3.280701 -3.024264 -1.167817
C -3.588890 -2.651557 1.577411
H -2.631770 -0.741596 1.288602
C -3.876173 -4.011719 -0.387202
H -3.142138 -3.165464 -2.234201
C -4.039635 -3.843760 0.995302
H -4.215351 -4.930557 -0.859610
C 1.302832 -0.579029 -1.276340
H 0.930592 -1.017822 -2.208560
H 1.645733 0.434701 -1.513727
C 2.473519 -1.406822 -0.729435
H 2.121731 -2.420117 -0.481345
H 2.830475 -0.967039 0.211935
C 3.640139 -1.510637 -1.719322
C 4.830056 -2.355548 -1.212535
H 5.540979 -2.481521 -2.039981
H -3.697362 -2.505003 2.649365
C -4.654502 -4.935233 1.838631
H -5.106145 -4.531192 2.750282
H -5.428220 -5.479764 1.287111
H -3.897720 -5.669142 2.146236
H -1.963312 2.033378 -1.130778
H 3.996158 -0.501486 -1.969548
C 5.554141 -1.760169 -0.021587
C 5.370924 -2.265561 1.272146
C 6.422906 -0.671267 -0.188527
C 6.029205 -1.701043 2.366918
H 4.707623 -3.114637 1.422490
C 7.083327 -0.103375 0.900551
H 6.587294 -0.268894 -1.186287
C 6.887880 -0.616723 2.184902
H 5.872667 -2.112072 3.361108
H 7.755734 0.736917 0.746317
H 7.404386 -0.177855 3.034500
H 0.468602 -0.047391 0.670041
H -0.183044 -1.516427 -0.009755
H 4.471167 -3.361342 -0.957461

H 3.277407 -1.951073 -2.658309
72
-1859.966356
C -1.268934 1.656461 -0.387109
C 0.367327 3.042834 -1.698671
H -0.250546 2.847514 -2.572177
C 1.517951 3.820520 -1.821743
H 1.786782 4.236850 -2.788829
C 2.318540 4.065986 -0.703691
H 3.215032 4.672930 -0.797086
C 1.958369 3.532601 0.534378
H 2.574744 3.721484 1.409168
C 0.804951 2.755121 0.659716
H 0.522754 2.348243 1.625188
C 0.003130 2.498819 -0.459011
N -1.954539 1.794675 0.925292
C -3.015016 2.817013 1.237257
C -2.423446 3.816662 2.248792
C -4.197745 2.056432 1.863999
C -3.475553 3.546874 -0.031778
H -1.594239 4.378859 1.806481
H -2.053991 3.286488 3.130498
H -3.193408 4.529281 2.564021
H -4.977447 2.763536 2.167086
H -3.866969 1.500430 2.744764
H -4.629086 1.352384 1.144583
H -3.903617 2.857012 -0.765824
H -2.662082 4.111962 -0.499812
H -4.253842 4.265402 0.246010
O -1.498546 1.102568 1.907891
C 0.149234 -0.503519 -0.268842
N -1.034408 0.255285 -0.726790
S -2.194870 -0.553047 -1.635777
O -3.253041 0.431285 -1.914878
O -1.543699 -1.247754 -2.752794
C -2.850028 -1.841239 -0.561631
C -3.279261 -3.024856 -1.168159
C -2.997929 -1.646222 0.812750
C -3.874359 -4.012714 -0.387616
H -3.140541 -3.165936 -2.234540
C -3.587987 -2.652451 1.577065
H -2.631825 -0.742052 1.288493
C -4.038050 -3.844941 0.994789
H -3.696690 -2.505984 2.649022
C 1.302846 -0.578542 -1.276489
H 0.930594 -1.017417 -2.208664
H 1.645662 0.435200 -1.513954
C 2.473612 -1.406239 -0.729608
H 2.121911 -2.419587 -0.481614
H 2.830499 -0.966499 0.211809
C 3.640270 -1.509869 -1.719476
C 4.830204 -2.354789 -1.212741
H 5.541161 -2.480641 -2.040177
H -4.213043 -4.931678 -0.860101
C -4.652038 -4.936625 1.838484
H -5.410299 -5.495797 1.280329
H -3.890791 -5.657883 2.164499
H -5.122518 -4.530240 2.739573
H -1.964092 2.033388 -1.130365
H 3.996233 -0.500673 -1.969597
C 5.554235 -1.759540 -0.021692
C 5.371367 -2.265432 1.271894
C 6.422634 -0.670306 -0.188387
C 6.029632 -1.701084 2.366764
H 4.708346 -3.114763 1.422044
C 7.083039 -0.102585 0.900789
H 6.586756 -0.267539 -1.186032
C 6.887942 -0.616435 2.184994
H 5.873365 -2.112502 3.360837
H 7.755158 0.737973 0.746746
H 7.404432 -0.177694 3.034667
H 0.468695 -0.046638 0.669842
H -0.182814 -1.515907 -0.009578
H 4.471339 -3.360625 -0.957796
H 3.277597 -1.950243 -2.658514

72
-1859.966356
C -1.268559 1.656423 -0.387141
C 0.367784 3.042681 -1.698707
H -0.250180 2.847518 -2.572183
C 1.518511 3.820212 -1.821802
H 1.787330 4.236583 -2.788874
C 2.319217 4.065470 -0.703791
H 3.215789 4.672293 -0.797207
C 1.959062 3.532040 0.534264
H 2.575530 3.720763 1.409021
C 0.805543 2.754716 0.659626
H 0.523352 2.347808 1.625087
C 0.003607 2.498611 -0.459066
N -1.954136 1.794770 0.925230
C -3.014457 2.817254 1.237233
C -2.422642 3.816970 2.248558
C -4.197134 2.056829 1.864257
C -3.475131 3.546984 -0.031828
H -1.593407 4.378989 1.806069
H -2.053165 3.286858 3.130290
H -3.192466 4.529743 2.563771
H -4.976674 2.764022 2.167556
H -3.866194 1.500793 2.744941
H -4.628734 1.352834 1.144950
H -3.903383 2.857047 -0.765695
H -2.661685 4.111942 -0.500060
H -4.253317 4.265615 0.245977
O -1.498171 1.102714 1.907884
C 0.149317 -0.503787 -0.268937
N -1.034264 0.255169 -0.726749
S -2.194822 -0.552954 -1.635757
O -3.252603 0.431684 -1.915263
O -1.543678 -1.248114 -2.752511
C -2.850599 -1.840736 -0.561483
C -2.997748 -1.645871 0.812885
C -3.280979 -3.024038 -1.168073
C -3.588230 -2.651920 1.577333
H -2.630833 -0.742056 1.288679
C -3.876392 -4.011535 -0.387525
H -3.142753 -3.165022 -2.234530
C -4.039389 -3.843866 0.995106
H -4.215890 -4.930203 -0.860045
C 1.302994 -0.578643 -1.276511
H 0.930758 -1.017324 -2.208784
H 1.645883 0.435122 -1.513768
C 2.473687 -1.406494 -0.729707
H 2.121932 -2.419863 -0.481874
H 2.830549 -0.966910 0.211792
C 3.640392 -1.510024 -1.719525
C 4.830268 -2.355077 -1.212874
H 5.541263 -2.480819 -2.040293
H -3.696316 -2.505604 2.649355
C -4.654287 -4.935500 1.838203
H -3.898920 -5.673453 2.139536
H -5.099664 -4.532525 2.753379
H -5.432998 -5.475219 1.288940
H -1.963628 2.033427 -1.130442
H 3.996426 -0.500801 -1.969440
C 5.554254 -1.760035 -0.021695
C 5.371042 -2.265895 1.271856
C 6.422927 -0.670997 -0.188228
C 6.029236 -1.701698 2.366846
H 4.707810 -3.115082 1.421884
C 7.083261 -0.103425 0.901069
H 6.587314 -0.268263 -1.185843
C 6.887819 -0.617237 2.185236
H 5.872701 -2.113087 3.360888
H 7.755597 0.736981 0.747150
H 7.404256 -0.178617 3.035004
H 0.468721 -0.047171 0.669898
H -0.182789 -1.516232 -0.009968
H 4.471357 -3.360943 -0.958113
H 3.277742 -1.950200 -2.658666
72
-1859.966356
C -1.268538 1.656411 -0.387271
C 0.368129 3.042508 -1.698594
H -0.249752 2.847450 -2.572153
C 1.518959 3.819913 -1.821520
H 1.787945 4.236288 -2.788543
C 2.319557 4.065034 -0.703403
H 3.216209 4.671760 -0.796684
C 1.959189 3.531598 0.534587
H 2.575573 3.720219 1.409426
C 0.805567 2.754404 0.659780
H 0.523205 2.347500 1.625193
C 0.003733 2.498428 -0.459018
N -1.954373 1.794960 0.924966
C -3.014540 2.817677 1.236690
C -3.474675 3.547548 -0.032483
C -2.422768 3.817235 2.248198
C -4.197577 2.057520 1.863363
H -3.902927 2.857737 -0.766469
H -2.660946 4.112274 -0.500503
H -4.252733 4.266397 0.245119
H -3.192515 4.530163 2.563246
H -1.593300 4.379083 1.805930
H -2.053626 3.287008 3.130000
H -3.867021 1.501400 2.744139
H -4.629128 1.353638 1.143915
H -4.977042 2.764891 2.166440
O -1.498770 1.102838 1.907733
C 0.149146 -0.503921 -0.268779
N -1.034407 0.255051 -0.726623
S -2.194931 -0.553018 -1.635675
O -3.252775 0.431598 -1.915029
O -1.543845 -1.248061 -2.752538
C -2.850556 -1.840892 -0.561433
C -3.280229 -3.024443 -1.167892
C -2.998323 -1.645749 0.812892
C -3.875578 -4.012033 -0.387296
H -3.141600 -3.165608 -2.234272
C -3.588688 -2.651793 1.577319
H -2.631948 -0.741662 1.288583
C -4.039150 -3.844120 0.995166
H -3.697290 -2.505233 2.649265
C 1.302807 -0.578931 -1.276359
H 0.930557 -1.017748 -2.208563
H 1.645699 0.434795 -1.513774
C 2.473510 -1.406706 -0.729456
H 2.121749 -2.420023 -0.481417
H 2.830426 -0.966950 0.211942
C 3.640152 -1.510438 -1.719323
C 4.830091 -2.355348 -1.212578
H 5.541009 -2.481262 -2.040035
H -4.214569 -4.930926 -0.859719
C -4.653644 -4.935680 1.838652
H -5.113056 -4.530637 2.745993
H -5.421023 -5.486273 1.284362
H -3.894957 -5.664398 2.153843
H -1.963425 2.033419 -1.130738
H 3.996155 -0.501263 -1.969481
C 5.554167 -1.759998 -0.021613
C 5.370698 -2.265211 1.272156
C 6.423163 -0.671285 -0.188571
C 6.028954 -1.700695 2.366942
H 4.707222 -3.114148 1.422515
C 7.083562 -0.103396 0.900523
H 6.587748 -0.269058 -1.186358
C 6.887860 -0.616559 2.184908
H 5.872220 -2.111583 3.361160
H 7.756152 0.736747 0.746274
H 7.404349 -0.177697 3.034519
H 0.468586 -0.047250 0.670016
H -0.183029 -1.516325 -0.009733
H 4.471227 -3.361164 -0.957550
H 3.277447 -1.950826 -2.658343
72
-1859.966356

C -1.268624 1.656428 -0.387160
C 0.805489 2.754518 0.659859
H 0.523249 2.347486 1.625254
C 1.959034 3.531827 0.534634
H 2.575478 3.720414 1.409440
C 2.319243 4.065424 -0.703334
H 3.215841 4.672227 -0.796636
C 1.518556 3.820356 -1.821401
H 1.787412 4.236856 -2.788407
C 0.367801 3.042842 -1.698445
H -0.250146 2.847825 -2.571967
C 0.003577 2.498597 -0.458892
N -1.954414 1.794726 0.925093
C -3.014776 2.817189 1.236970
C -2.422963 3.817121 2.248116
C -4.197371 2.056787 1.864158
C -3.475582 3.546720 -0.032156
H -2.053457 3.287187 3.129943
H -3.192782 4.529961 2.563184
H -1.593733 4.379032 1.805475
H -4.976919 2.763984 2.167432
H -3.866353 1.500862 2.744882
H -4.628996 1.352708 1.144946
H -3.903706 2.856634 -0.765961
H -2.662254 4.111791 -0.500458
H -4.253891 4.265240 0.245600
O -1.498486 1.102757 1.907834
C 0.149269 -0.503775 -0.268896
N -1.034312 0.255164 -0.726739
S -2.194857 -0.552973 -1.635731
O -3.252744 0.431590 -1.915082
O -1.543751 -1.247997 -2.752587
C -2.850454 -1.840867 -0.561479
C -2.997759 -1.645959 0.812899
C -3.280527 -3.024262 -1.168046
C -3.588107 -2.652060 1.577322
H -2.631066 -0.742035 1.288662
C -3.875835 -4.011857 -0.387486
H -3.142155 -3.165242 -2.234485
C -4.038976 -3.844158 0.995087
H -4.215135 -4.930601 -0.859990
C 1.302989 -0.578498 -1.276434
H 0.930766 -1.017068 -2.208767
H 1.645867 0.435301 -1.513552
C 2.473683 -1.406427 -0.729745
H 2.121909 -2.419820 -0.482037
H 2.830583 -0.966965 0.211797
C 3.640352 -1.509864 -1.719615
C 4.830305 -2.354836 -1.213007
H 5.541291 -2.480504 -2.040447
H -3.696344 -2.505718 2.649330
C -4.653547 -4.935834 1.838373
H -5.105163 -4.531977 2.750118
H -5.427206 -5.480480 1.286885
H -3.896574 -5.669618 2.145820
H -1.963563 2.033488 -1.130551
H 3.996302 -0.500616 -1.969547
C 5.554277 -1.759798 -0.021819
C 6.422807 -0.670637 -0.188304
C 5.371199 -2.265795 1.271699
C 7.083143 -0.103090 0.901005
H 6.587076 -0.267782 -1.185890
C 6.029398 -1.701625 2.366701
H 4.708067 -3.115067 1.421688
C 6.887846 -0.617049 2.185137
H 7.755366 0.737414 0.747122
H 5.872972 -2.113123 3.360716
H 7.404291 -0.178451 3.034912
H 0.468582 -0.047239 0.670012
H -0.182824 -1.516256 -0.010058
H 4.471465 -3.360735 -0.958273
H 3.277702 -1.950076 -2.658739

C6-PBN

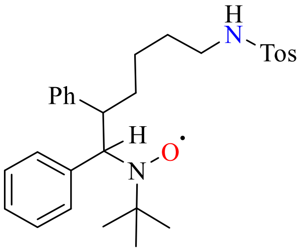

Name	E(B3LYP)	H(B3LYP)	g-factor	α_N	α_H
Tosyl_NH_pentane_Ph_C6_radical_PBN_adduct_0016_nmr_three_basis_sets	-1860.628486	-1859.981153	2.00582937	14.629260	3.891600
Tosyl_NH_pentane_Ph_C6_radical_PBN_adduct_0014_nmr_three_basis_sets	-1860.628486	-1859.981150	2.00582920	14.630100	3.894560
Tosyl_NH_pentane_Ph_C6_radical_PBN_adduct_0043_nmr_three_basis_sets	-1860.628486	-1859.981144	2.00582937	14.628830	3.890550
Tosyl_NH_pentane_Ph_C6_radical_PBN_adduct_0150_nmr_three_basis_sets	-1860.628499	-1859.980209	2.00582900	14.629750	3.887990
Tosyl_NH_pentane_Ph_C6_radical_PBN_adduct_0015_nmr_three_basis_sets	-1860.628499	-1859.980208	2.00582893	14.629820	3.890820
Tosyl_NH_pentane_Ph_C6_radical_PBN_adduct_0017_nmr_three_basis_sets	-1860.628499	-1859.980208	2.00582897	14.629760	3.890480
Tosyl_NH_pentane_Ph_C6_radical_PBN_adduct_0021_nmr_three_basis_sets	-1860.628499	-1859.980208	2.00582897	14.629910	3.890880
Tosyl_NH_pentane_Ph_C6_radical_PBN_adduct_0022_nmr_three_basis_sets	-1860.628499	-1859.980208	2.00582900	14.629650	3.890800
Boltzman averaged for 298.15 K			2.0059156059	14.7812016268	3.6719681444

72
-1859.980209
C -2.271375 0.576280 0.524126
C -2.583403 1.840399 -1.668548
H -1.538149 1.650610 -1.892216
C -3.374259 2.553936 -2.569988
H -2.940896 2.906494 -3.502420
C -4.714382 2.817733 -2.277355
H -5.328909 3.373745 -2.980311
C -5.257691 2.365616 -1.073905
H -6.298386 2.567093 -0.834105
C -4.464906 1.654780 -0.171291
H -4.897610 1.303758 0.762247
C -3.122006 1.381243 -0.458217
N -0.963209 1.246421 0.746676
C -0.722438 2.199619 1.889537
C -1.940985 3.123282 2.073233
C 0.513580 3.046080 1.557137
C -0.471132 1.375443 3.168584
H -2.846969 2.579410 2.356384
H -2.150835 3.691728 1.162503
H -1.722546 3.832775 2.878258
H 0.656071 3.780992 2.356473
H 0.378665 3.583161 0.613225
H 1.412585 2.431977 1.491978
H -0.306757 2.052015 4.014685
H 0.418717 0.749556 3.056039
H -1.327306 0.737017 3.414137
O -0.014584 1.003351 -0.089726
C 1.961672 -2.357556 -0.024176
N 1.963032 -1.110634 0.761411
S 3.403968 -0.585071 1.416627
O 3.085529 0.657914 2.133974
O 4.000175 -1.730279 2.115011
C 4.501095 -0.157335 0.059123
C 4.360560 1.080875 -0.576666
C 5.461643 -1.073641 -0.370453
C 5.191945 1.393385 -1.647612
H 3.617858 1.791652 -0.229120
C 6.287145 -0.742712 -1.446153
H 5.570288 -2.022654 0.143234
C 6.167933 0.489915 -2.099803
H 7.038551 -1.454627 -1.778170
C 0.574060 -2.688935 -0.587374
H 0.273911 -1.897942 -1.286566
H 0.709349 -3.589639 -1.199884
C -0.540755 -2.957967 0.443519
H -0.130217 -3.577494 1.251976
H -1.318666 -3.563599 -0.039520
C -1.201158 -1.711206 1.063297
H -0.427802 -1.078969 1.501683
C -2.049332 -0.896234 0.054849
H -1.492677 -0.815769 -0.882691
H 5.086076 2.357275 -2.139540
C 7.077605 0.855237 -3.248259
H 7.826216 1.594846 -2.936300
H 7.614650 -0.019000 -3.628185
H 6.514880 1.297706 -4.077907
H -2.778898 0.560138 1.493312
H 1.424015 -0.330754 0.368399
H -1.842490 -2.032685 1.893509
C -3.372334 -1.587239 -0.255142
C -3.688584 -1.957599 -1.568895
C -4.301259 -1.884608 0.754671
C -4.889894 -2.603246 -1.869469
H -2.985854 -1.733349 -2.367790
C -5.505056 -2.525339 0.460057
H -4.085659 -1.615799 1.786304
C -5.804072 -2.888990 -0.855058
H -5.109594 -2.881393 -2.896987
H -6.208738 -2.743667 1.259299
H -6.740022 -3.391012 -1.085045
H 2.673825 -2.299262 -0.862255
H 2.298830 -3.158890 0.640023

72
-1859.980208
C -2.271383 0.576256 0.524197
C -2.583177 1.840407 -1.668471
H -1.537900 1.650615 -1.892028
C -3.373936 2.553931 -2.569998
H -2.940476 2.906497 -3.502379
C -4.714097 2.817706 -2.277517
H -5.328547 3.373711 -2.980544
C -5.257534 2.365590 -1.074129
H -6.298256 2.567057 -0.834443
C -4.464844 1.654766 -0.171423
H -4.897647 1.303753 0.762070
C -3.121913 1.381232 -0.458208
N -0.963226 1.246424 0.746796
C -0.722556 2.199689 1.889561
C -0.471128 1.375629 3.168667
C -1.941222 3.123194 2.073205
C 0.513354 3.046298 1.557085
H -1.327242 0.737160 3.414309
H -0.306740 2.052271 4.014704
H 0.418758 0.749806 3.056108
H -2.847170 2.579211 2.356243
H -2.151052 3.691655 1.162479
H -1.722927 3.832668 2.878281
H 1.412451 2.432317 1.492038
H 0.655713 3.781359 2.356306
H 0.378382 3.583210 0.613085
O -0.014497 1.003227 -0.089450
C 1.961676 -2.357629 -0.023885
N 1.962963 -1.110703 0.761673
S 3.403915 -0.584937 1.416653
O 3.085486 0.658146 2.133826
O 4.000310 -1.729996 2.115120
C 4.500936 -0.157302 0.059035
C 5.461695 -1.073514 -0.370347
C 4.360135 1.080710 -0.577018
C 6.287104 -0.742666 -1.446104
H 5.570501 -2.022412 0.143516
C 5.191462 1.393158 -1.648060
H 3.617249 1.791383 -0.229659
C 6.167658 0.489838 -2.100004
H 5.085338 2.356880 -2.140251
C 0.574108 -2.689090 -0.587133
H 0.274000 -1.898201 -1.286454
H 0.709475 -3.589881 -1.199496
C -0.540788 -2.958011 0.443690
H -0.130334 -3.577553 1.252178
H -1.318704 -3.563599 -0.039394
C -1.201156 -1.711216 1.063429
H -0.427804 -1.079003 1.501850
C -2.049277 -0.896241 0.054924
H -1.492559 -0.815749 -0.882574
H 7.038619 -1.454526 -1.778000
C 7.077635 0.855143 -3.248216
H 6.516331 1.304983 -4.074809
H 7.831271 1.588630 -2.933850
H 7.608882 -0.020479 -3.633108
H -2.778961 0.560108 1.493355
H 1.423790 -0.330904 0.368711
H -1.842544 -2.032669 1.893607
C -3.372246 -1.587274 -0.255150
C -3.688423 -1.957588 -1.568935
C -4.301223 -1.884701 0.754596
C -4.889699 -2.603248 -1.869599
H -2.985661 -1.733281 -2.367785
C -5.504988 -2.525447 0.459893
H -4.085693 -1.615914 1.786247
C -5.803924 -2.889054 -0.855249
H -5.109339 -2.881358 -2.897139
H -6.208708 -2.743816 1.259087
H -6.739845 -3.391094 -1.085307
H 2.673816 -2.299360 -0.861974
H 2.298840 -3.158945 0.640335
72

-1859.980208
C -2.271388 0.576214 0.524168
C -2.583237 1.840420 -1.668458
H -1.537973 1.650607 -1.892057
C -3.374008 2.553999 -2.569929
H -2.940570 2.906588 -3.502312
C -4.714149 2.817804 -2.277389
H -5.328609 3.373855 -2.980371
C -5.257557 2.365656 -1.074001
H -6.298265 2.567143 -0.834271
C -4.464856 1.654775 -0.171350
H -4.897637 1.303732 0.762143
C -3.121944 1.381217 -0.458191
N -0.963234 1.246378 0.746755
C -0.722533 2.199608 1.889561
C 0.513367 3.046215 1.557063
C -0.471083 1.375524 3.168642
C -1.941191 3.123115 2.073253
H 1.412454 2.432226 1.491939
H 0.655781 3.781233 2.356313
H 0.378355 3.583176 0.613097
H 0.418803 0.749701 3.056085
H -1.327195 0.737052 3.414288
H -0.306688 2.052157 4.014685
H -1.722866 3.832591 2.878319
H -2.847128 2.579134 2.356332
H -2.151060 3.691577 1.162537
O -0.014525 1.003226 -0.089524
C 1.961703 -2.357617 -0.024000
N 1.962985 -1.110710 0.761588
S 3.403918 -0.584967 1.416630
O 3.085451 0.658093 2.133826
O 4.000267 -1.730057 2.115084
C 4.500980 -0.157303 0.059051
C 5.461699 -1.073541 -0.370358
C 4.360264 1.080753 -0.576937
C 6.287157 -0.742674 -1.446076
H 5.570451 -2.022470 0.143456
C 5.191637 1.393220 -1.647932
H 3.617402 1.791443 -0.229560
C 6.167797 0.489870 -2.099905
H 5.085589 2.356981 -2.140063
C 0.574132 -2.689022 -0.587266
H 0.274026 -1.898064 -1.286509
H 0.709477 -3.589758 -1.199713
C -0.540755 -2.958021 0.443553
H -0.130279 -3.577581 1.252015
H -1.318652 -3.563619 -0.039550
C -1.201156 -1.711266 1.063330
H -0.427806 -1.079037 1.501738
C -2.049315 -0.896286 0.054871
H -1.492632 -0.815796 -0.882647
H 7.038640 -1.454556 -1.777995
C 7.077802 0.855208 -3.248086
H 7.609638 -0.020269 -3.632489
H 6.516400 1.304356 -4.074993
H 7.830931 1.589285 -2.933890
H -2.778951 0.560050 1.493332
H 1.423861 -0.330893 0.368599
H -1.842499 -2.032750 1.893529
C -3.372311 -1.587279 -0.255171
C -3.688562 -1.957519 -1.568959
C -4.301249 -1.884726 0.754604
C -4.889870 -2.603128 -1.869598
H -2.985828 -1.733199 -2.367829
C -5.505047 -2.525421 0.459924
H -4.085670 -1.615996 1.786259
C -5.804056 -2.888956 -0.855221
H -5.109565 -2.881180 -2.897142
H -6.208736 -2.743809 1.259141
H -6.740005 -3.390952 -1.085258
H 2.673860 -2.299328 -0.862075
H 2.298848 -3.158953 0.640204
72
-1859.980208
C 2.271368 0.576222 -0.524188
C 2.583161 1.840453 1.668425
H 1.537884 1.650668 1.891985
C 3.373912 2.554026 2.569919
H 2.940446 2.906640 3.502278
C 4.714072 2.817793 2.277429
H 5.328516 3.373840 2.980426
C 5.257518 2.365610 1.074072
H 6.298242 2.567066 0.834383
C 4.464838 1.654733 0.171400
H 4.897649 1.303660 -0.762067
C 3.121906 1.381219 0.458188
N 0.963231 1.246400 -0.746805
C 0.722559 2.199604 -1.889655
C 1.941231 3.123088 -2.073338
C -0.513341 3.046222 -1.557191
C 0.471126 1.375481 -3.168710
H 2.847167 2.579089 -2.356384
H 2.151086 3.691570 -1.162632
H 1.722935 3.832550 -2.878425
H -1.412433 2.432243 -1.492092
H -0.655723 3.781242 -2.356445
H -0.378341 3.583183 -0.613224
H 1.327248 0.737018 -3.414338
H 0.306722 2.052087 -4.014774
H -0.418752 0.749652 -3.056136
O 0.014485 1.003239 0.089428
C -1.961686 -2.357705 0.023827
N -1.962984 -1.110729 -0.761657
S -3.403925 -0.584941 -1.416633
O -3.085462 0.658126 -2.133816
O -4.000340 -1.729997 -2.115083
C -4.500921 -0.157269 -0.059002
C -4.360107 1.080730 0.577053
C -5.461691 -1.073477 0.370381
C -5.191435 1.393189 1.648094
H -3.617194 1.791390 0.229723
C -6.287096 -0.742624 1.446134
H -5.570499 -2.022370 -0.143488
C -6.167644 0.489887 2.100031
H -7.038612 -1.454478 1.778038
C -0.574133 -2.689134 0.587127
H -0.274064 -1.898221 1.286438
H -0.709484 -3.589916 1.199505
C 0.540796 -2.958054 -0.443663
H 0.130374 -3.577616 -1.252149
H 1.318707 -3.563623 0.039454
C 1.201161 -1.711256 -1.063395
H 0.427803 -1.079038 -1.501807
C 2.049269 -0.896276 -0.054895
H 1.492541 -0.815783 0.882597
H -5.085298 2.356911 2.140283
C -7.077672 0.855232 3.248191
H -6.516515 1.305729 4.074518
H -7.831716 1.588202 2.933577
H -7.608449 -0.020482 3.633508
H 2.778952 0.560045 -1.493341
H -1.423826 -0.330943 -0.368650
H 1.842533 -2.032697 -1.893587
C 3.372250 -1.587269 0.255205
C 3.688456 -1.957476 1.569013
C 4.301216 -1.884749 -0.754533
C 4.889750 -2.603084 1.869706
H 2.985697 -1.733133 2.367855
C 5.505000 -2.525444 -0.459799
H 4.085672 -1.616043 -1.786203
C 5.803966 -2.888945 0.855364
H 5.109413 -2.881109 2.897264
H 6.208712 -2.743860 -1.258987
H 6.739904 -3.390941 1.085444
H -2.673869 -2.299482 0.861882
H -2.298800 -3.159004 -0.640436
72
-1859.980208
C 2.271437 0.576240 -0.524152

C 2.583352 1.840378 1.668505
H 1.538085 1.650592 1.892111
C 3.374156 2.553905 2.569987
H 2.940742 2.906482 3.502385
C 4.714304 2.817675 2.277439
H 5.328788 3.373687 2.980429
C 5.257682 2.365543 1.074032
H 6.298394 2.567001 0.834295
C 4.464947 1.654712 0.171371
H 4.897707 1.303681 -0.762136
C 3.122030 1.381191 0.458220
N 0.963307 1.246460 -0.746708
C 0.722625 2.199727 -1.889488
C -0.513202 3.046413 -1.556915
C 0.471062 1.375683 -3.168572
C 1.941332 3.123159 -2.073215
H -0.655619 3.781444 -2.356152
H -0.378097 3.583362 -0.612955
H -1.412323 2.432483 -1.491734
H 0.306695 2.052341 -4.014601
H -0.418871 0.749931 -3.055994
H 1.327113 0.737144 -3.414256
H 2.847219 2.579131 -2.356362
H 2.151285 3.691579 -1.162492
H 1.723010 3.832675 -2.878247
O 0.014588 1.003304 0.089558
C -1.961728 -2.357559 0.024058
N -1.963031 -1.110677 -0.761569
S -3.403976 -0.584992 -1.416633
O -3.085541 0.658054 -2.133869
O -4.000290 -1.730117 -2.115059
C -4.501051 -0.157317 -0.059070
C -5.461789 -1.073543 0.370322
C -4.360326 1.080735 0.576921
C -6.287260 -0.742665 1.446026
H -5.570541 -2.022472 -0.143493
C -5.191712 1.393213 1.647905
H -3.617443 1.791412 0.229563
C -6.167895 0.489880 2.099856
H -5.085652 2.356969 2.140044
C -0.574138 -2.688958 0.587284
H -0.274005 -1.897996 1.286511
H -0.709463 -3.589690 1.199741
C 0.540712 -2.957960 -0.443574
H 0.130201 -3.577494 -1.252036
H 1.318613 -3.563581 0.039495
C 1.201125 -1.711204 -1.063341
H 0.427780 -1.078953 -1.501725
C 2.049316 -0.896259 -0.054881
H 1.492650 -0.815764 0.882647
H -7.038755 -1.454539 1.777936
C -7.077937 0.855229 3.248004
H -6.516644 1.304867 4.074716
H -7.831390 1.588901 2.933633
H -7.609397 -0.020334 3.632730
H 2.778991 0.560083 -1.493321
H -1.423926 -0.330838 -0.368604
H 1.842449 -2.032687 -1.893556
C 3.372292 -1.587307 0.255128
C 3.688534 -1.957615 1.568898
C 4.301211 -1.884751 -0.754666
C 4.889819 -2.603284 1.869503
H 2.985814 -1.733302 2.367782
C 5.504985 -2.525505 -0.460020
H 4.085633 -1.615972 -1.786309
C 5.803988 -2.889104 0.855108
H 5.109510 -2.881387 2.897034
H 6.208661 -2.743889 -1.259250
H 6.739919 -3.391147 1.085119
H -2.673853 -2.299237 0.862157
H -2.298904 -3.158911 -0.640111
72
-1859.980208
C 2.271309 0.576171 -0.524235
C 2.583146 1.840513 1.668315
H 1.537902 1.650644 1.891959
C 3.373896 2.554194 2.569724
H 2.940465 2.906800 3.502103
C 4.714013 2.818073 2.277128
H 5.328455 3.374205 2.980061
C 5.257414 2.365893 1.073749
H 6.298101 2.567435 0.833974
C 4.464731 1.654908 0.171161
H 4.897508 1.303845 -0.762324
C 3.121845 1.381282 0.458055
N 0.963139 1.246294 -0.746887
C 0.722441 2.199431 -1.889775
C -0.513541 3.045986 -1.557424
C 0.471128 1.375235 -3.168806
C 1.941052 3.123002 -2.073452
H -1.412599 2.431951 -1.492352
H -0.655929 3.780955 -2.356726
H -0.378634 3.583008 -0.613479
H -0.418731 0.749374 -3.056262
H 1.327290 0.736787 -3.414339
H 0.306764 2.051793 -4.014916
H 1.722733 3.832408 -2.878580
H 2.847040 2.579056 -2.356435
H 2.150827 3.691541 -1.162762
O 0.014435 1.003213 0.089417
C -1.961672 -2.357713 0.024296
N -1.962938 -1.110939 -0.761501
S -3.403891 -0.585276 -1.416571
O -3.085450 0.657656 -2.134006
O -4.000298 -1.730467 -2.114808
C -4.500862 -0.157364 -0.058998
C -4.359827 1.080644 0.577026
C -5.461863 -1.073331 0.370332
C -5.191187 1.393331 1.647964
H -3.616720 1.791123 0.229735
C -6.287310 -0.742244 1.445996
H -5.570828 -2.022238 -0.143481
C -6.167652 0.490260 2.099843
H -7.039017 -1.453925 1.777841
C -0.574098 -2.689045 0.587601
H -0.274004 -1.898002 1.286754
H -0.709432 -3.589721 1.200137
C 0.540793 -2.958123 -0.443189
H 0.130326 -3.577795 -1.251569
H 1.318720 -3.563630 0.039984
C 1.201150 -1.711432 -1.063144
H 0.427792 -1.079294 -1.501671
C 2.049286 -0.896289 -0.054791
H 1.492619 -0.815713 0.882729
H -5.084887 2.357038 2.140145
C -7.077588 0.855833 3.247999
H -7.610168 -0.019386 3.631956
H -6.515980 1.304184 4.075201
H -7.830071 1.590639 2.933970
H 2.778872 0.559941 -1.493398
H -1.423766 -0.331071 -0.368677
H 1.842517 -2.033036 -1.893278
C 3.372310 -1.587207 0.255295
C 3.688553 -1.957406 1.569096
C 4.301277 -1.884640 -0.754459
C 4.889882 -2.602964 1.869768
H 2.985795 -1.733102 2.367950
C 5.505094 -2.525284 -0.459747
H 4.085697 -1.615949 -1.786125
C 5.804096 -2.888777 0.855412
H 5.109572 -2.880985 2.897321
H 6.208804 -2.743667 -1.258947
H 6.740062 -3.390730 1.085475
H -2.673804 -2.299260 0.862379
H -2.298844 -3.159149 -0.639770
72
-1859.980208
C 2.271388 0.576241 -0.524154
C 2.583204 1.840369 1.668521
H 1.537933 1.650563 1.892091

C 3.373969 2.553900 2.570037
H 2.940519 2.906455 3.502426
C 4.714120 2.817699 2.277532
H 5.328573 3.373710 2.980550
C 5.257542 2.365600 1.074132
H 6.298255 2.567088 0.834424
C 4.464846 1.654767 0.171436
H 4.897638 1.303771 -0.762069
C 3.121928 1.381205 0.458248
N 0.963241 1.246416 -0.746737
C 0.722568 2.199670 -1.889530
C 0.471131 1.375600 -3.168623
C 1.941245 3.123156 -2.073194
C -0.513324 3.046294 -1.557042
H -0.418803 0.749839 -3.056110
H 1.327211 0.737065 -3.414210
H 0.306835 2.052233 -4.014688
H 1.722926 3.832666 -2.878232
H 2.847164 2.579160 -2.356308
H 2.151136 3.691582 -1.162462
H -0.655729 3.781300 -2.356305
H -0.378304 3.583269 -0.613084
H -1.412414 2.432314 -1.491904
O 0.014535 1.003293 0.089554
C -1.961728 -2.357549 0.023899
N -1.962983 -1.110608 -0.761626
S -3.403901 -0.584831 -1.416674
O -3.085419 0.658271 -2.133786
O -4.000223 -1.729875 -2.115222
C -4.500970 -0.157274 -0.059068
C -5.461796 -1.073477 0.370179
C -4.360154 1.080680 0.577095
C -6.287227 -0.742695 1.445943
H -5.570644 -2.022310 -0.143794
C -5.191503 1.393064 1.648136
H -3.617227 1.791356 0.229828
C -6.167741 0.489730 2.099973
H -5.085383 2.356751 2.140396
C -0.574167 -2.689033 0.587151
H -0.274036 -1.898137 1.286456
H -0.709538 -3.589812 1.199530
C 0.540723 -2.957989 -0.443673
H 0.130249 -3.577505 -1.252168
H 1.318614 -3.563610 0.039411
C 1.201149 -1.711221 -1.063403
H 0.427817 -1.078968 -1.501808
C 2.049296 -0.896277 -0.054906
H 1.492592 -0.815800 0.882602
H -7.038809 -1.454536 1.777729
C -7.077699 0.854941 3.248230
H -7.830417 1.589567 2.934356
H -7.609998 -0.020479 3.632124
H -6.516146 1.303354 4.075440
H 2.778960 0.560114 -1.493314
H -1.423834 -0.330808 -0.368643
H 1.842502 -2.032696 -1.893599
C 3.372272 -1.587308 0.255128
C 3.688482 -1.957639 1.568902
C 4.301225 -1.884719 -0.754643
C 4.889768 -2.603294 1.869530
H 2.985735 -1.733348 2.367770
C 5.505002 -2.525462 -0.459973
H 4.085678 -1.615920 -1.786287
C 5.803972 -2.889082 0.855156
H 5.109434 -2.881414 2.897062
H 6.208705 -2.743817 -1.259186
H 6.739903 -3.391115 1.085185
H -2.673874 -2.299278 0.861984
H -2.298897 -3.158848 -0.640340
72
-1859.980208
C -2.271371 0.576222 0.524213
C -2.583189 1.840478 -1.668383
H -1.537908 1.650718 -1.891949
C -3.373959 2.554047 -2.569863
H -2.940503 2.906686 -3.502217
C -4.714125 2.817780 -2.277366
H -5.328581 3.373824 -2.980356
C -5.257556 2.365572 -1.074011
H -6.298283 2.566999 -0.834311
C -4.464854 1.654703 -0.171350
H -4.897656 1.303617 0.762117
C -3.121921 1.381213 -0.458153
N -0.963215 1.246391 0.746771
C -0.722530 2.199665 1.889508
C -0.471155 1.375666 3.168668
C -1.941152 3.123250 2.073086
C 0.513423 3.046202 1.557020
H -0.306763 2.052361 4.014663
H 0.418720 0.749813 3.056183
H -1.327288 0.737236 3.414342
H -2.847143 2.579337 2.356114
H -2.150920 3.691685 1.162328
H -1.722838 3.832745 2.878138
H 1.412489 2.432175 1.491963
H 0.655827 3.781256 2.356239
H 0.378474 3.583123 0.613021
O -0.014539 1.003298 -0.089568
C 1.961615 -2.357623 -0.024083
N 1.962889 -1.110723 0.761491
S 3.403803 -0.584975 1.416563
O 3.085318 0.658112 2.133710
O 4.000143 -1.730036 2.115073
C 4.500919 -0.157346 0.059011
C 5.461809 -1.073498 -0.370199
C 4.360098 1.080626 -0.577111
C 6.287348 -0.742621 -1.445847
H 5.570613 -2.022375 0.143699
C 5.191552 1.393102 -1.648048
H 3.617076 1.791236 -0.229913
C 6.167900 0.489855 -2.099802
H 5.085413 2.356785 -2.140309
C 0.574015 -2.689152 -0.587212
H 0.273823 -1.898301 -1.286541
H 0.709348 -3.589965 -1.199548
C -0.540797 -2.958058 0.443711
H -0.130263 -3.577545 1.252199
H -1.318722 -3.563700 -0.039292
C -1.201167 -1.711254 1.063422
H -0.427803 -1.079027 1.501807
C -2.049292 -0.896289 0.054924
H -1.492567 -0.815787 -0.882568
H 7.038964 -1.454435 -1.777613
C 7.078046 0.855195 -3.247869
H 7.608954 -0.020504 -3.633058
H 6.516941 1.305554 -4.074311
H 7.831975 1.588258 -2.933218
H -2.778934 0.560068 1.493378
H 1.423755 -0.330902 0.368526
H -1.842523 -2.032689 1.893628
C -3.372267 -1.587285 -0.255163
C -3.688497 -1.957465 -1.568976
C -4.301214 -1.884792 0.754583
C -4.889794 -2.603070 -1.869662
H -2.985754 -1.733100 -2.367825
C -5.505003 -2.525486 0.459856
H -4.085656 -1.616105 1.786254
C -5.803992 -2.888958 -0.855310
H -5.109474 -2.881073 -2.897222
H -6.208699 -2.743920 1.259053
H -6.739932 -3.390954 -1.085384
H 2.673681 -2.299301 -0.862235
H 2.298884 -3.158928 0.640097
72
-1859.980206
C -2.271375 0.576283 0.524115
C -2.583285 1.840405 -1.668564
H -1.538022 1.650608 -1.892176
C -3.374097 2.553919 -2.570055
H -2.940688 2.906478 -3.502461

C -4.714240 2.817694 -2.277503
H -5.328732 3.373689 -2.980500
C -5.257612 2.365585 -1.074083
H -6.298322 2.567050 -0.834345
C -4.464871 1.654773 -0.171413
H -4.897626 1.303764 0.762104
C -3.121955 1.381244 -0.458268
N -0.963219 1.246408 0.746674
C -0.722551 2.199676 1.889464
C -0.471524 1.375638 3.168660
C -1.941072 3.123437 2.072823
C 0.513583 3.046003 1.557168
H -1.327758 0.737270 3.414137
H -0.307275 2.052306 4.014704
H 0.418323 0.749711 3.056370
H -2.847167 2.579653 2.355765
H -2.150640 3.691840 1.162002
H -1.722774 3.832957 2.877859
H 1.412536 2.431813 1.492126
H 0.656066 3.780942 2.356479
H 0.378824 3.583056 0.613217
O -0.014569 1.003348 -0.089695
C 1.961712 -2.357464 -0.024403
N 1.962961 -1.110700 0.761446
S 3.403936 -0.585157 1.416646
O 3.085517 0.657813 2.134020
O 4.000129 -1.730420 2.114956
C 4.501103 -0.157389 0.059205
C 5.461307 -1.073905 -0.370719
C 4.360988 1.081070 -0.576166
C 6.286834 -0.742940 -1.446377
H 5.569703 -2.023096 0.142687
C 5.192417 1.393627 -1.647068
H 3.618585 1.791996 -0.228301
C 6.168021 0.489941 -2.099628
H 5.086887 2.357730 -2.138645
C 0.574106 -2.688802 -0.587606
H 0.273928 -1.897746 -1.286705
H 0.709378 -3.589455 -1.200191
C -0.540666 -2.957916 0.443317
H -0.130057 -3.577459 1.251723
H -1.318572 -3.563558 -0.039717
C -1.201085 -1.711224 1.063198
H -0.427731 -1.078984 1.501581
C -2.049353 -0.896237 0.054829
H -1.492763 -0.815746 -0.882746
H 7.037966 -1.455011 -1.778673
C 7.077668 0.855229 -3.248111
H 7.614783 -0.019005 -3.627940
H 6.514897 1.297540 -4.077812
H 7.826197 1.594943 -2.936229
H -2.778931 0.560162 1.493282
H 1.423954 -0.330787 0.368490
H -1.842353 -2.032786 1.893424
C -3.372369 -1.587241 -0.255069
C -3.688710 -1.957599 -1.568801
C -4.301237 -1.884602 0.754798
C -4.890033 -2.603249 -1.869297
H -2.986036 -1.733345 -2.367742
C -5.505050 -2.525331 0.460261
H -4.085584 -1.615774 1.786412
C -5.804145 -2.888991 -0.854830
H -5.109798 -2.881397 -2.896800
H -6.208683 -2.743653 1.259544
H -6.740106 -3.391018 -1.084755
H 2.673818 -2.298968 -0.862502
H 2.298924 -3.158896 0.639648
72
-1859.980206
C -2.271294 0.576220 0.524227
C -2.583014 1.840578 -1.668336
H -1.537760 1.650709 -1.891930
C -3.373728 2.554247 -2.569789
H -2.940252 2.906857 -3.502146
C -4.713859 2.818117 -2.277262
H -5.328274 3.374233 -2.980232
C -5.257315 2.365943 -1.073905
H -6.298017 2.567478 -0.834186
C -4.464672 1.654972 -0.171274
H -4.897493 1.303910 0.762193
C -3.121766 1.381348 -0.458100
N -0.963088 1.246258 0.746903
C -0.722315 2.199314 1.889858
C -1.940859 3.122949 2.073635
C 0.513703 3.045799 1.557504
C -0.471005 1.375033 3.168839
H -1.722452 3.832318 2.878775
H -2.846862 2.579049 2.356661
H -2.150658 3.691533 1.162980
H 1.412694 2.431684 1.492271
H 0.656230 3.780657 2.356882
H 0.378768 3.582943 0.613630
H -1.327179 0.736592 3.414350
H -0.306615 2.051541 4.014984
H 0.418841 0.749158 3.056256
O -0.014384 1.003105 -0.089373
C 1.961709 -2.357756 -0.023940
N 1.963028 -1.110877 0.761723
S 3.404043 -0.585241 1.416711
O 3.085651 0.657703 2.134141
O 4.000472 -1.730453 2.114893
C 4.500921 -0.157343 0.059079
C 4.360598 1.081168 -0.576198
C 5.461112 -1.073745 -0.371048
C 5.191788 1.393836 -1.647223
H 3.618256 1.792042 -0.228092
C 6.286423 -0.742655 -1.446866
H 5.569735 -2.022921 0.142338
C 6.167346 0.490200 -2.100063
H 7.037600 -1.454610 -1.779294
C 0.574167 -2.689005 -0.587361
H 0.274158 -1.897953 -1.286543
H 0.709508 -3.589682 -1.199895
C -0.540817 -2.958074 0.443332
H -0.130441 -3.577775 1.251735
H -1.318709 -3.563556 -0.039924
C -1.201214 -1.711374 1.063228
H -0.427862 -1.079215 1.501739
C -2.049312 -0.896265 0.054825
H -1.492610 -0.815730 -0.882679
H 5.086151 2.357989 -2.138689
C 7.076311 0.855504 -3.249086
H 7.619014 -0.017310 -3.624182
H 6.511899 1.290487 -4.081588
H 7.819943 1.601219 -2.939868
H -2.778882 0.560019 1.493375
H 1.423927 -0.331017 0.368799
H -1.842597 -2.032942 1.893362
C -3.372337 -1.587164 -0.255288
C -4.301352 -1.884550 0.754436
C -3.688536 -1.957396 -1.569092
C -5.505170 -2.525180 0.459694
H -4.085818 -1.615823 1.786102
C -4.889863 -2.602945 -1.869794
H -2.985749 -1.733112 -2.367926
C -5.804122 -2.888713 -0.855465
H -6.208919 -2.743520 1.258871
H -5.109516 -2.880994 -2.897347
H -6.740087 -3.390659 -1.085552
H 2.673953 -2.299477 -0.861938
H 2.298715 -3.159143 0.640269

C5-PBN

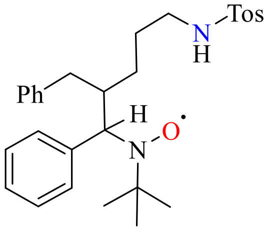

Name	E(B3LYP)	H(B3LYP)	g-factor	α_N	α_H
Tosyl_NH_pentane_Ph_C5_radical_PBN_adduct_0097_nmr_three_basis_sets	-1860.629614	-1859.981174	2.00600737	15.405290	2.649270
Tosyl_NH_pentane_Ph_C5_radical_PBN_adduct_0106_nmr_three_basis_sets	-1860.629614	-1859.981174	2.00600737	15.405880	2.649410
Tosyl_NH_pentane_Ph_C5_radical_PBN_adduct_0147_nmr_three_basis_sets	-1860.629614	-1859.981174	2.00600737	15.406030	2.649500
Tosyl_NH_pentane_Ph_C5_radical_PBN_adduct_0145_nmr_three_basis_sets	-1860.629614	-1859.981173	2.00600730	15.405780	2.649030
Tosyl_NH_pentane_Ph_C5_radical_PBN_adduct_0146_nmr_three_basis_sets	-1860.629614	-1859.981173	2.00600740	15.406190	2.649040
Tosyl_NH_pentane_Ph_C5_radical_PBN_adduct_0150_nmr_three_basis_sets	-1860.629614	-1859.981173	2.00600737	15.405700	2.648720
Tosyl_NH_pentane_Ph_C5_radical_PBN_adduct_0154_nmr_three_basis_sets	-1860.629614	-1859.981173	2.00600737	15.405400	2.649310
Tosyl_NH_pentane_Ph_C5_radical_PBN_adduct_0124_nmr_three_basis_sets	-1860.628101	-1859.979667	2.00602480	15.321720	2.828560
Boltzman averaged for 298.15 K			2.0059906848	15.3232198075	2.3727837366

72
-1859.981174
C -2.287331 -0.830513 -0.476434
C -2.083512 -1.295312 2.026409
H -1.008492 -1.175661 1.935549
C -2.652985 -1.593913 3.264255
H -2.015824 -1.689096 4.139551
C -4.034047 -1.772773 3.382514
H -4.474289 -2.005108 4.348334
C -4.843390 -1.654275 2.252252
H -5.918223 -1.793125 2.331788
C -4.272576 -1.358063 1.012343
H -4.910570 -1.263134 0.136290
C -2.889793 -1.171814 0.884501
N -1.229911 -1.817182 -0.833519
C -1.477511 -3.168270 -1.454925
C -1.355955 -4.232772 -0.347080
C -0.388224 -3.377708 -2.522539
C -2.864289 -3.231425 -2.107365
H -1.455505 -5.232932 -0.782184
H -2.135990 -4.105665 0.410126
H -0.380304 -4.159414 0.140152
H -0.487513 -2.646220 -3.332207
H -0.486111 -4.379337 -2.953748
H 0.610286 -3.279266 -2.091373
H -3.672557 -3.126374 -1.377155
H -2.977247 -4.210708 -2.583316
H -2.984437 -2.471422 -2.887087
O -0.067685 -1.638506 -0.306919
C 1.304272 1.439064 -1.433907
N 1.728826 0.086820 -1.837676
S 3.331061 -0.351351 -1.649837
O 3.396338 -1.760297 -2.060154
O 4.148813 0.670344 -2.315977
C 3.714287 -0.280867 0.105635
C 4.525003 0.741160 0.598379
C 3.177533 -1.246047 0.964673
C 4.804577 0.791244 1.964780
H 4.936759 1.476892 -0.083828
C 3.464521 -1.178053 2.324772
H 2.543978 -2.036189 0.574481
C 4.283937 -0.163791 2.846853
H 3.049069 -1.927368 2.994365
C 0.014736 1.848314 -2.158404
H -0.256499 2.848927 -1.806844
H 0.240556 1.935335 -3.228359
C -1.179457 0.882972 -2.013027
H -0.894557 -0.068283 -2.476388
H -2.004866 1.271156 -2.625653
C -1.710342 0.615475 -0.579627
C -2.794341 1.638876 -0.151334
H -3.203091 1.332096 0.817619
H 5.440051 1.585209 2.349170
C 4.611926 -0.123065 4.320304
H 4.966202 0.866468 4.625040
H 3.739400 -0.377825 4.931756
H 5.400838 -0.845714 4.566693
H -3.070659 -0.929894 -1.231579
H 1.134751 -0.647866 -1.437614
H -0.884016 0.672557 0.135988
C -2.311790 3.071000 -0.035964
C -2.726495 4.054763 -0.942934
C -1.444743 3.447791 1.001543
C -2.278663 5.373437 -0.829558
H -3.407702 3.785576 -1.747204
C -0.993828 4.762509 1.118674
H -1.124003 2.702993 1.727062
C -1.407796 5.731339 0.200400
H -2.612725 6.119803 -1.545674
H -0.322939 5.032718 1.930131
H -1.058384 6.756247 0.291245
H 1.178761 1.504330 -0.343578
H 2.100678 2.132934 -1.715343
H -3.626284 1.593488 -0.867187
72
-1859.981174

C 2.287209 0.830659 -0.476549
C 2.083078 1.295560 2.026247
H 1.008084 1.175751 1.935285
C 2.652385 1.594303 3.264134
H 2.015122 1.689436 4.139361
C 4.033409 1.773367 3.382523
H 4.473521 2.005810 4.348375
C 4.842882 1.654929 2.252348
H 5.917687 1.793933 2.331984
C 4.272235 1.358575 1.012396
H 4.910333 1.263691 0.136413
C 2.889491 1.172120 0.884426
N 1.229722 1.817195 -0.833823
C 1.477281 3.168291 -1.455224
C 2.864043 3.231487 -2.107692
C 1.355702 4.232802 -0.347390
C 0.387966 3.377691 -2.522819
H 2.976941 4.210750 -2.583696
H 2.984221 2.471450 -2.887375
H 3.672329 3.126524 -1.377491
H 0.380058 4.159421 0.139854
H 1.455211 5.232958 -0.782512
H 2.135749 4.105738 0.409811
H 0.485821 4.379316 -2.954043
H -0.610528 3.279233 -2.091623
H 0.487255 2.646193 -3.332477
O 0.067461 1.638436 -0.307333
C -1.304126 -1.439420 -1.433926
N -1.728903 -0.087294 -1.837864
S -3.331179 0.350678 -1.649885
O -3.396757 1.759513 -2.060521
O -4.148869 -0.671302 -2.315658
C -3.714133 0.280535 0.105665
C -3.177158 1.245799 0.964457
C -4.524985 -0.741260 0.598681
C -3.464042 1.178128 2.324603
H -2.543492 2.035769 0.574095
C -4.804440 -0.791027 1.965113
H -4.936879 -1.477078 -0.083348
C -4.283559 0.164098 2.846963
H -5.440002 -1.584812 2.349730
C -0.014521 -1.848559 -2.158360
H 0.256824 -2.849109 -1.806705
H -0.240307 -1.935714 -3.228313
C 1.179542 -0.883055 -2.013048
H 0.894541 0.068130 -2.476491
H 2.005006 -1.271182 -2.625635
C 1.710367 -0.615394 -0.579664
C 2.794466 -1.638654 -0.151290
H 3.203209 -1.331738 0.817624
H -3.048405 1.927533 2.993982
C -4.611478 0.123726 4.320438
H -4.964268 -0.866168 4.625739
H -3.739335 0.380134 4.931745
H -5.401474 0.845327 4.566418
H 3.070601 0.930049 -1.231623
H -1.134860 0.647522 -1.438004
H 0.884029 -0.672514 0.135936
C 2.312068 -3.070816 -0.035752
C 1.445006 -3.447563 1.001758
C 2.726960 -4.054663 -0.942546
C 0.994264 -4.762325 1.119067
H 1.124116 -2.702697 1.727141
C 2.279302 -5.373380 -0.828989
H 3.408176 -3.785508 -1.746819
C 1.408423 -5.731242 0.200973
H 0.323357 -5.032495 1.930523
H 2.613509 -6.119813 -1.544969
H 1.059147 -6.756184 0.291957
H -1.178620 -1.504542 -0.343587
H -2.100421 -2.133448 -1.715284
H 3.626386 -1.593256 -0.867167
72
-1859.981174
C -2.287322 -0.830252 -0.476616
C -2.083054 -1.294741 2.026243
H -1.008052 -1.175089 1.935185
C -2.652305 -1.593199 3.264224
H -2.014987 -1.688263 4.139418
C -4.033342 -1.772063 3.382751
H -4.473408 -2.004287 4.348677
C -4.842887 -1.653709 2.252620
H -5.917704 -1.792557 2.332363
C -4.272297 -1.357637 1.012575
H -4.910451 -1.262815 0.136627
C -2.889540 -1.171391 0.884464
N -1.230123 -1.817109 -0.833869
C -1.478103 -3.168249 -1.455022
C -2.864894 -3.231134 -2.107454
C -1.356810 -4.232592 -0.347000
C -0.388866 -3.378144 -2.522595
H -2.978068 -4.210415 -2.583355
H -2.984872 -2.471142 -2.887211
H -3.673143 -3.125877 -1.377253
H -0.381135 -4.159398 0.140212
H -1.456610 -5.232798 -0.781939
H -2.136806 -4.105170 0.410192
H -0.487005 -4.379819 -2.953634
H 0.609658 -3.279882 -2.091423
H -0.487964 -2.646765 -3.332384
O -0.067760 -1.638569 -0.307538
C 1.304463 1.439061 -1.434471
N 1.729034 0.086776 -1.838131
S 3.331205 -0.351444 -1.649777
O 3.396641 -1.760316 -2.060308
O 4.149183 0.670360 -2.315469
C 3.713777 -0.281218 0.105855
C 3.176554 -1.246400 0.964579
C 4.524546 0.740582 0.599004
C 3.463109 -1.178644 2.324793
H 2.542957 -2.036369 0.574108
C 4.803668 0.790436 1.965496
H 4.936626 1.476348 -0.082970
C 4.282531 -0.164613 2.847284
H 5.439154 1.584237 2.350210
C 0.014913 1.848238 -2.158974
H -0.256264 2.848912 -1.807544
H 0.240692 1.935109 -3.228950
C -1.179318 0.882965 -2.013427
H -0.894522 -0.068349 -2.476728
H -2.004746 1.271138 -2.626035
C -1.710119 0.615636 -0.579972
C -2.793912 1.639236 -0.151657
H -3.202810 1.332452 0.817234
H 3.047272 -1.927972 2.994131
C 4.610152 -0.124124 4.320821
H 5.401071 -0.844729 4.566760
H 4.961639 0.866162 4.626378
H 3.738251 -0.381755 4.931943
H -3.070818 -0.929547 -1.231595
H 1.134795 -0.647866 -1.438237
H -0.883717 0.672642 0.135560
C -2.311048 3.071240 -0.036081
C -1.443816 3.447661 1.001406
C -2.725686 4.055266 -0.942793
C -0.992668 4.762277 1.118769
H -1.123122 2.702656 1.726733
C -2.277620 5.373840 -0.829182
H -3.407023 3.786368 -1.747048
C -1.406580 5.731375 0.200754
H -0.321637 5.032194 1.930205
H -2.611637 6.120415 -1.545101
H -1.056986 6.756204 0.291781
H 1.178983 1.504435 -0.344146
H 2.100872 2.132890 -1.715994
H -3.625814 1.594113 -0.867573
72
-1859.981173
C -2.287354 -0.830381 -0.476505
C -2.083120 -1.294979 2.026340

H -1.008122 -1.175268 1.935314
C -2.652384 -1.593532 3.264292
H -2.015080 -1.688611 4.139495
C -4.033413 -1.772475 3.382778
H -4.473490 -2.004772 4.348681
C -4.842938 -1.654105 2.252633
H -5.917749 -1.793014 2.332345
C -4.272335 -1.357940 1.012618
H -4.910472 -1.263107 0.136659
C -2.889584 -1.171612 0.884549
N -1.230064 -1.817143 -0.833745
C -1.477889 -3.168249 -1.455049
C -2.864722 -3.231274 -2.107374
C -1.356358 -4.232690 -0.347148
C -0.388704 -3.377862 -2.522727
H -2.977810 -4.210549 -2.583310
H -2.984861 -2.471269 -2.887093
H -3.672923 -3.126148 -1.377102
H -0.380652 -4.159424 0.139986
H -1.456074 -5.232865 -0.782177
H -2.136306 -4.105438 0.410125
H -0.486709 -4.379512 -2.953856
H 0.609842 -3.279481 -2.091630
H -0.487986 -2.646422 -3.332439
O -0.067743 -1.638545 -0.307344
C 1.304303 1.439088 -1.434198
N 1.728901 0.086828 -1.837937
S 3.331108 -0.351321 -1.649838
O 3.396528 -1.760217 -2.060299
O 4.148954 0.670465 -2.315719
C 3.713987 -0.280986 0.105721
C 3.176686 -1.245965 0.964653
C 4.525109 0.740638 0.598601
C 3.463528 -1.178156 2.324787
H 2.542821 -2.035816 0.574375
C 4.804531 0.790543 1.965047
H 4.937249 1.476229 -0.083526
C 4.283327 -0.164268 2.847020
H 5.440314 1.584203 2.349553
C 0.014763 1.848283 -2.158703
H -0.256456 2.848926 -1.807215
H 0.240563 1.935225 -3.228668
C -1.179436 0.882959 -2.013238
H -0.894574 -0.068326 -2.476561
H -2.004853 1.271120 -2.625868
C -1.710281 0.615560 -0.579815
C -2.794192 1.639058 -0.151539
H -3.203060 1.332263 0.817361
H 3.047643 -1.927319 2.994283
C 4.611090 -0.123732 4.320523
H 3.738008 -0.376855 4.931869
H 5.398722 -0.847651 4.567252
H 4.966937 0.865290 4.625086
H -3.070821 -0.929730 -1.231506
H 1.134742 -0.647849 -1.437985
H -0.883921 0.672637 0.135760
C -2.311473 3.071115 -0.036013
C -1.444340 3.447673 1.001505
C -2.726137 4.055049 -0.942813
C -0.993311 4.762335 1.118816
H -1.123629 2.702741 1.726899
C -2.278190 5.373667 -0.829255
H -3.407400 3.786043 -1.747094
C -1.407246 5.731340 0.200715
H -0.322355 5.032360 1.930278
H -2.612224 6.120169 -1.545242
H -1.057746 6.756205 0.291700
H 1.178802 1.504375 -0.343872
H 2.100709 2.132951 -1.715653
H -3.626083 1.593823 -0.867460
72
-1859.981173
C -2.287497 -0.829925 -0.476578
C -2.083386 -1.294250 2.026327
H -1.008358 -1.174813 1.935290
C -2.652731 -1.592498 3.264315
H -2.015457 -1.687619 4.139535
C -4.033806 -1.771086 3.382816
H -4.473945 -2.003148 4.348748
C -4.843295 -1.652672 2.252651
H -5.918140 -1.791313 2.332373
C -4.272612 -1.356809 1.012599
H -4.910719 -1.261952 0.136621
C -2.889815 -1.170831 0.884515
N -1.230485 -1.817030 -0.833719
C -1.478730 -3.168168 -1.454758
C -2.865555 -3.230855 -2.107138
C -1.357596 -4.232461 -0.346668
C -0.389579 -3.378352 -2.522363
H -2.978918 -4.210150 -2.582966
H -2.985423 -2.470895 -2.886943
H -3.673757 -3.125404 -1.376914
H -0.381896 -4.159403 0.140512
H -1.457584 -5.232680 -0.781535
H -2.137550 -4.104849 0.410535
H -0.487954 -4.380024 -2.953357
H 0.608981 -3.280287 -2.091231
H -0.488548 -2.646984 -3.332178
O -0.068105 -1.638690 -0.307356
C 1.304612 1.438760 -1.434757
N 1.728948 0.086314 -1.838088
S 3.331049 -0.352145 -1.649746
O 3.396265 -1.761081 -2.060082
O 4.149190 0.669440 -2.315570
C 3.713693 -0.281770 0.105861
C 3.176330 -1.246755 0.964732
C 4.524658 0.739943 0.598843
C 3.462945 -1.178877 2.324919
H 2.542585 -2.036665 0.574380
C 4.803842 0.789920 1.965327
H 4.936849 1.475546 -0.083238
C 4.282572 -0.164914 2.847251
H 5.439493 1.583649 2.349913
C 0.015121 1.847983 -2.159345
H -0.255907 2.848768 -1.808117
H 0.240902 1.934600 -3.229342
C -1.179236 0.882905 -2.013587
H -0.894582 -0.068514 -2.476759
H -2.004652 1.271089 -2.626206
C -1.709993 0.615835 -0.580069
C -2.793514 1.639722 -0.151749
H -3.202443 1.333077 0.817172
H 3.047005 -1.928050 2.994369
C 4.610176 -0.124308 4.320788
H 4.964052 0.865300 4.625757
H 3.737581 -0.379416 4.931992
H 5.399266 -0.846730 4.567259
H -3.070991 -0.929128 -1.231570
H 1.134582 -0.648147 -1.438050
H -0.883520 0.672696 0.135392
C -2.310296 3.071614 -0.036226
C -1.442887 3.447833 1.001186
C -2.724782 4.055737 -0.942903
C -0.991418 4.762342 1.118507
H -1.122308 2.702754 1.726488
C -2.276396 5.374205 -0.829332
H -3.406251 3.786999 -1.747099
C -1.405181 5.731536 0.200527
H -0.320253 5.032103 1.929884
H -2.610299 6.120858 -1.545223
H -1.055338 6.756284 0.291523
H 1.179142 1.504430 -0.344446
H 2.101136 2.132388 -1.716450
H -3.625465 1.594785 -0.867621
72
-1859.981173
C -2.287655 -0.829967 -0.476402
C -2.083775 -1.294100 2.026560
H -1.008733 -1.174734 1.935600
C -2.653240 -1.592216 3.264526

H -2.016044 -1.687302 4.139807
C -4.034335 -1.770715 3.382927
H -4.474569 -2.002670 4.348841
C -4.843723 -1.652348 2.252684
H -5.918583 -1.790923 2.332327
C -4.272920 -1.356622 1.012655
H -4.910948 -1.261805 0.136615
C -2.890101 -1.170732 0.884671
N -1.230613 -1.817098 -0.833345
C -1.478759 -3.168258 -1.454365
C -2.865552 -3.231038 -2.106809
C -1.357631 -4.232499 -0.346223
C -0.389543 -3.378431 -2.521907
H -2.978872 -4.210385 -2.582541
H -2.985398 -2.471165 -2.886703
H -3.673796 -3.125523 -1.376638
H -0.381953 -4.159389 0.140993
H -1.457571 -5.232739 -0.781052
H -2.137620 -4.104876 0.410945
H -0.487824 -4.380142 -2.952833
H 0.608997 -3.280261 -2.090750
H -0.488534 -2.647132 -3.331782
O -0.068286 -1.638717 -0.306870
C 1.304622 1.438342 -1.434573
N 1.728835 0.085849 -1.837871
S 3.330931 -0.352698 -1.649746
O 3.395895 -1.761775 -2.059659
O 4.149021 0.668591 -2.316089
C 3.713986 -0.281790 0.105749
C 4.525368 0.739846 0.598159
C 3.176538 -1.246318 0.965108
C 4.804919 0.790220 1.964570
H 4.937623 1.475069 -0.084293
C 3.463501 -1.178039 2.325185
H 2.542478 -2.036189 0.575185
C 4.283577 -0.164122 2.846955
H 3.047501 -1.926851 2.995009
C 0.015192 1.847689 -2.159204
H -0.255760 2.848497 -1.807979
H 0.241020 1.934291 -3.229193
C -1.179258 0.882721 -2.013489
H -0.894649 -0.068748 -2.476590
H -2.004591 1.270946 -2.626194
C -1.710145 0.615786 -0.579989
C -2.793699 1.639718 -0.151875
H -3.202695 1.333202 0.817060
H 5.440917 1.583880 2.348716
C 4.611397 -0.123116 4.320434
H 5.396950 -0.849041 4.567894
H 4.969991 0.865187 4.624080
H 3.737574 -0.373225 4.931978
H -3.071087 -0.929245 -1.231449
H 1.134492 -0.648563 -1.437712
H -0.883735 0.672720 0.135540
C -2.310479 3.071623 -0.036524
C -1.443145 3.447989 1.000898
C -2.724880 4.055613 -0.943384
C -0.991672 4.762512 1.118057
H -1.122629 2.703016 1.726337
C -2.276488 5.374094 -0.829978
H -3.406284 3.786759 -1.747597
C -1.405352 5.731572 0.199898
H -0.320568 5.032387 1.929448
H -2.610325 6.120641 -1.546011
H -1.055506 6.756330 0.290767
H 1.179136 1.504032 -0.344267
H 2.101214 2.131900 -1.716255
H -3.625601 1.594684 -0.867797
72
-1859.981173
C -2.287412 -0.830304 -0.476471
C -2.083373 -1.294879 2.026396
H -1.008355 -1.175304 1.935422
C -2.652745 -1.593325 3.264325
H -2.015504 -1.688464 4.139566
C -4.033805 -1.772085 3.382738
H -4.473966 -2.004301 4.348622
C -4.843252 -1.653644 2.252544
H -5.918085 -1.792416 2.332198
C -4.272541 -1.357586 1.012551
H -4.910617 -1.262707 0.136553
C -2.889758 -1.171437 0.884556
N -1.230171 -1.817154 -0.833605
C -1.478044 -3.168253 -1.454862
C -2.864877 -3.231240 -2.107199
C -1.356577 -4.232681 -0.346935
C -0.388865 -3.377965 -2.522535
H -2.978013 -4.210533 -2.583085
H -2.984967 -2.471266 -2.886957
H -3.673078 -3.126030 -1.376938
H -0.380871 -4.159451 0.140205
H -1.456338 -5.232859 -0.781948
H -2.136525 -4.105374 0.410327
H -0.486950 -4.379616 -2.953648
H 0.609687 -3.279653 -2.091439
H -0.488091 -2.646531 -3.332259
O -0.067859 -1.638603 -0.307153
C 1.304386 1.438935 -1.434281
N 1.728872 0.086615 -1.837857
S 3.331060 -0.351645 -1.649812
O 3.396338 -1.760588 -2.060132
O 4.148948 0.670013 -2.315836
C 3.714047 -0.281170 0.105714
C 3.176921 -1.246170 0.964737
C 4.525038 0.740606 0.598489
C 3.463811 -1.178230 2.324850
H 2.543160 -2.036140 0.574531
C 4.804513 0.790642 1.964924
H 4.937057 1.476205 -0.083701
C 4.283489 -0.164185 2.846981
H 5.440202 1.584416 2.349348
C 0.014853 1.848159 -2.158801
H -0.256299 2.848838 -1.807366
H 0.240644 1.935029 -3.228775
C -1.179396 0.882912 -2.013265
H -0.894598 -0.068409 -2.476549
H -2.004813 1.271089 -2.625885
C -1.710224 0.615599 -0.579811
C -2.794040 1.639185 -0.151530
H -3.202874 1.332460 0.817407
H 3.048065 -1.927410 2.994416
C 4.611297 -0.123529 4.320471
H 5.398364 -0.848002 4.567379
H 4.967900 0.865294 4.624785
H 3.738023 -0.375839 4.931885
H -3.070835 -0.929621 -1.231523
H 1.134676 -0.647992 -1.437835
H -0.883836 0.672617 0.135738
C -2.311241 3.071222 -0.036093
C -1.444055 3.447790 1.001379
C -2.725882 4.055132 -0.942931
C -0.992953 4.762435 1.118607
H -1.123358 2.702878 1.726799
C -2.277864 5.373733 -0.829456
H -3.407184 3.786119 -1.747178
C -1.406868 5.731414 0.200468
H -0.321958 5.032467 1.930034
H -2.611883 6.120216 -1.545470
H -1.057311 6.756266 0.291390
H 1.178906 1.504383 -0.343959
H 2.100826 2.132715 -1.715842
H -3.625979 1.593968 -0.867398
72
-1859.979667
C -2.699159 -0.645103 -0.460631
C -3.518731 -0.703666 1.956464
H -2.484184 -0.772168 2.277700
C -4.546228 -0.695442 2.899884
H -4.303483 -0.739614 3.958322
C -5.881176 -0.634265 2.490716

H -6.679174 -0.628243 3.228167
C -6.182808 -0.584979 1.129354
H -7.217197 -0.539710 0.799255
C -5.153764 -0.596169 0.184935
H -5.396788 -0.553816 -0.874651
C -3.812044 -0.651824 0.585174
N -1.805590 -1.820724 -0.269496
C -2.084427 -3.211263 -0.781233
C -3.161600 -3.197597 -1.873291
C -2.541981 -4.072563 0.411826
C -0.763146 -3.749885 -1.358336
H -4.129630 -2.851104 -1.498646
H -3.296848 -4.221803 -2.235609
H -2.867780 -2.583103 -2.731233
H -3.488353 -3.706891 0.822736
H -1.787064 -4.052694 1.201721
H -2.683252 -5.109058 0.087117
H -0.457365 -3.174904 -2.239505
H -0.893889 -4.794084 -1.661574
H 0.040232 -3.695833 -0.621079
O -0.899836 -1.724528 0.640176
C 1.341276 1.006785 -0.123531
N 1.666702 -0.415879 0.069765
S 2.861358 -1.150140 -0.825404
O 2.668546 -2.594329 -0.615255
O 2.941647 -0.627063 -2.201460
C 4.358372 -0.623941 0.012333
C 4.529825 -0.919685 1.369096
C 5.359069 0.023656 -0.708693
C 5.712969 -0.552192 1.999607
H 3.742551 -1.423754 1.920118
C 6.541071 0.386650 -0.059244
H 5.208763 0.239296 -1.760721
C 6.737813 0.106059 1.297417
H 7.321154 0.895525 -0.619690
C 0.533198 1.375520 -1.379532
H 0.327203 2.449948 -1.325237
H 1.166259 1.214687 -2.258353
C -0.770965 0.583625 -1.593833
H -0.497005 -0.464519 -1.755102
H -1.221348 0.917746 -2.538967
C -1.846916 0.661225 -0.479855
C -2.781188 1.888827 -0.640612
H -3.601729 1.801184 0.080093
H 5.847322 -0.778248 3.054732
C 8.017912 0.492691 1.998761
H 7.816656 1.119801 2.875568
H 8.556064 -0.394196 2.355601
H 8.687769 1.046130 1.334136
H -3.155017 -0.758872 -1.447147
H 0.863936 -1.040219 0.201601
H -1.360616 0.726512 0.498575
C -2.116192 3.236279 -0.439924
C -1.668101 3.625676 0.832071
C -1.952774 4.134110 -1.502768
C -1.060990 4.865638 1.031424
H -1.803003 2.951935 1.675770
C -1.346618 5.377913 -1.308350
H -2.303144 3.855721 -2.494251
C -0.895391 5.746961 -0.040421
H -0.722206 5.146981 2.025278
H -1.229443 6.057667 -2.148446
H -0.424310 6.714003 0.113751
H 0.806307 1.310341 0.782682
H 2.282950 1.566935 -0.120829
H -3.240540 1.856005 -1.637828
72
-1859.979495
C -2.233906 -1.026528 -0.410228
C -2.052921 -1.465811 2.101186
H -0.984254 -1.291711 2.029543
C -2.630799 -1.781856 3.331500
H -2.005822 -1.838135 4.218930
C -4.002930 -2.026299 3.426565
H -4.449866 -2.271283 4.386203
C -4.795288 -1.956599 2.279872
H -5.863467 -2.147091 2.340363
C -4.215786 -1.645312 1.048402
H -4.841036 -1.593268 0.159515
C -2.841226 -1.394065 0.943207
N -1.071007 -1.901601 -0.717494
C -1.183937 -3.175413 -1.517898
C 0.178928 -3.878738 -1.492612
C -1.562779 -2.828328 -2.971272
C -2.254925 -4.087590 -0.888501
H 0.106314 -4.793608 -2.089782
H 0.465712 -4.151620 -0.473910
H 0.970108 -3.253046 -1.913135
H -1.659677 -3.753404 -3.549438
H -0.789783 -2.213943 -3.443150
H -2.518724 -2.298498 -3.037360
H -2.014343 -4.312855 0.154826
H -2.294246 -5.030287 -1.445055
H -3.252557 -3.640230 -0.921493
O 0.040991 -1.616194 -0.132989
C 1.181399 1.499427 -1.293705
N 1.684357 0.182324 -1.724312
S 3.322494 -0.136657 -1.659939
O 3.454249 -1.538260 -2.081557
O 4.012338 0.939415 -2.382523
C 3.833644 -0.038520 0.060775
C 4.631085 1.022216 0.488356
C 3.411105 -1.024412 0.959400
C 5.012327 1.091348 1.829271
H 4.953582 1.773437 -0.224287
C 3.798015 -0.936919 2.293066
H 2.788110 -1.846017 0.620049
C 4.606270 0.117364 2.749698
H 3.470653 -1.701749 2.993247
C -0.126868 1.849818 -2.016965
H -0.472029 2.814463 -1.630845
H 0.104325 1.994178 -3.079582
C -1.247751 0.794773 -1.925997
H -0.867516 -0.123513 -2.383886
H -2.075747 1.121824 -2.569583
C -1.810640 0.475412 -0.516142
C -3.009784 1.387133 -0.146009
H -3.423263 1.054495 0.811895
H 5.637443 1.915896 2.162802
C 5.041098 0.180804 4.194396
H 5.414013 1.175299 4.457529
H 4.215690 -0.065270 4.871624
H 5.846735 -0.537731 4.393805
H -2.981469 -1.226390 -1.184974
H 1.175123 -0.593860 -1.287223
H -1.026306 0.621349 0.233979
C -2.679824 2.862495 -0.038742
C -3.137773 3.783400 -0.989829
C -1.914419 3.342815 1.035381
C -2.829803 5.142093 -0.883061
H -3.742989 3.433277 -1.823158
C -1.603168 4.697898 1.146126
H -1.563146 2.646803 1.794435
C -2.058419 5.603842 0.184185
H -3.195272 5.838409 -1.633451
H -1.009596 5.048440 1.986601
H -1.818177 6.660112 0.270062
H 1.048823 1.527478 -0.203123
H 1.936336 2.246204 -1.553589
H -3.803220 1.246937 -0.892728
72
-1859.979495
C -2.233843 -1.026600 -0.410223
C -2.052878 -1.466054 2.101162
H -0.984221 -1.291874 2.029555
C -2.630758 -1.782232 3.331441
H -2.005794 -1.838532 4.218879
C -4.002873 -2.026781 3.426460
H -4.449812 -2.271868 4.386070
C -4.795212 -1.957053 2.279754

H	-5.863378	-2.147626	2.340211
C	-4.215707	-1.645634	1.048320
H	-4.840943	-1.593569	0.159425
C	-2.841163	-1.394280	0.943171
N	-1.070873	-1.901568	-0.717527
C	-1.183692	-3.175318	-1.518057
C	-2.254687	-4.087591	-0.888818
C	0.179198	-3.878590	-1.492743
C	-1.562443	-2.828114	-2.971425
H	-2.293917	-5.030247	-1.445448
H	-3.252339	-3.640278	-0.921862
H	-2.014182	-4.312926	0.154511
H	0.970377	-3.252829	-1.913165
H	0.106656	-4.793410	-2.089997
H	0.465925	-4.151552	-0.474046
H	-2.518384	-2.298280	-3.037528
H	-1.659304	-3.753143	-3.549676
H	-0.789418	-2.213690	-3.443203
O	0.041081	-1.616135	-0.132951
C	1.181356	1.499477	-1.293488
N	1.684338	0.182426	-1.724278
S	3.322518	-0.136455	-1.659944
O	3.454357	-1.538028	-2.081634
O	4.012274	0.939694	-2.382497
C	3.833685	-0.038359	0.060767
C	4.631171	1.022347	0.488344
C	3.411137	-1.024249	0.959386
C	5.012445	1.091450	1.829249
H	4.953663	1.773573	-0.224297
C	3.798077	-0.936783	2.293047
H	2.788100	-1.845828	0.620047
C	4.606379	0.117464	2.749673

H	3.470698	-1.701608	2.993225
C	-0.126913	1.849939	-2.016705
H	-0.472080	2.814542	-1.630482
H	0.104273	1.994414	-3.079308
C	-1.247784	0.794874	-1.925844
H	-0.867533	-0.123362	-2.383817
H	-2.075780	1.121976	-2.569405
C	-1.810683	0.475377	-0.516025
C	-3.009914	1.386971	-0.145856
H	-3.423318	1.054294	0.812066
H	5.637589	1.915978	2.162778
C	5.041278	0.180853	4.194353
H	4.216120	-0.066044	4.871582
H	5.847494	-0.537107	4.393494
H	5.413455	1.175564	4.457720
H	-2.981375	-1.226459	-1.184998
H	1.175189	-0.593797	-1.287151
H	-1.026385	0.621330	0.234130
C	-2.680104	2.862367	-0.038621
C	-1.914835	3.342801	1.035547
C	-3.138045	3.783189	-0.989792
C	-1.603703	4.697914	1.146254
H	-1.563573	2.646853	1.794665
C	-2.830195	5.141912	-0.883064
H	-3.743165	3.432977	-1.823155
C	-2.058942	5.603774	0.184228
H	-1.010234	5.048546	1.986764
H	-3.195657	5.838162	-1.633517
H	-1.818794	6.660069	0.270075
H	1.048782	1.527370	-0.202903
H	1.936283	2.246300	-1.553270
H	-3.803364	1.246682	-0.892544

C2-PBN

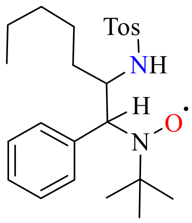

Name	E(B3LYP)	H(B3LYP)	g-factor	α_N	α_H
Tosyl_NH_pentane_Ph_C2_radical_PBN_adduct_0193_nmr_three_basis_sets	-1860.630642	-1859.982628	2.00609913	14.107510	6.139690
Tosyl_NH_pentane_Ph_C2_radical_PBN_adduct_0277_nmr_three_basis_sets	-1860.630642	-1859.982628	2.00609917	14.106950	6.139100
Tosyl_NH_pentane_Ph_C2_radical_PBN_adduct_0326_nmr_three_basis_sets	-1860.630642	-1859.982628	2.00609917	14.107080	6.137940
Tosyl_NH_pentane_Ph_C2_radical_PBN_adduct_0192_nmr_three_basis_sets	-1860.630642	-1859.982627	2.00609920	14.107040	6.138430
Tosyl_NH_pentane_Ph_C2_radical_PBN_adduct_0194_nmr_three_basis_sets	-1860.630642	-1859.982627	2.00609927	14.107130	6.136020
Tosyl_NH_pentane_Ph_C2_radical_PBN_adduct_0195_nmr_three_basis_sets	-1860.630642	-1859.982627	2.00609920	14.107140	6.137950
Tosyl_NH_pentane_Ph_C2_radical_PBN_adduct_0200_nmr_three_basis_sets	-1860.630642	-1859.982627	2.00609917	14.106970	6.139090
Tosyl_NH_pentane_Ph_C2_radical_PBN_adduct_0290_nmr_three_basis_sets	-1860.630642	-1859.982627	2.00609920	14.106780	6.138900
Boltzman averaged for 298.15 K			2.0061077527	14.0575603467	5.6905732500

72
-1859.982628
C -2.592355 -0.106658 -0.248795
C -4.209932 1.701083 0.575432
H -3.879870 1.508961 1.591720
C -5.170859 2.681281 0.323870
H -5.588678 3.248361 1.151486
C -5.594843 2.934497 -0.983029
H -6.344121 3.697316 -1.176347
C -5.053947 2.198371 -2.038402
H -5.380808 2.383370 -3.058142
C -4.097219 1.213260 -1.785975
H -3.686246 0.636836 -2.612171
C -3.662245 0.956388 -0.478836
N -2.782642 -0.850902 1.011748
C -3.726676 -2.012101 1.192186
C -4.881701 -1.546690 2.099422
C -2.928761 -3.136382 1.878032
C -4.268037 -2.505174 -0.156017
H -5.483933 -0.775533 1.608365
H -4.486155 -1.140049 3.034166
H -5.533549 -2.394910 2.334878
H -3.588881 -3.986674 2.080234
H -2.511178 -2.784454 2.824500
H -2.112574 -3.474570 1.232052
H -4.830508 -1.728588 -0.684369
H -4.954520 -3.337283 0.032343
H -3.465187 -2.874621 -0.801206
O -2.208138 -0.401947 2.074437
C -1.132483 0.456056 -0.369713
N -0.120362 -0.570351 -0.043622
S 0.183867 -1.797131 -1.156353
O -0.717256 -2.954418 -0.978830
O 0.231415 -1.145696 -2.474726
C 1.820997 -2.310378 -0.651091
C 2.045409 -3.649432 -0.336363
C 2.864592 -1.378496 -0.624859
C 3.332773 -4.055821 0.018815
H 1.223660 -4.356202 -0.366907
C 4.138460 -1.801485 -0.263807
H 2.682058 -0.338612 -0.873791
C 4.394179 -3.144319 0.063677
H 4.945730 -1.074309 -0.229603
C -0.795366 1.733485 0.421138
H -1.630242 2.435555 0.321236
H -0.718061 1.489652 1.485092
C 0.493109 2.402219 -0.075381
H 0.366374 2.697945 -1.127824
H 1.311018 1.673411 -0.054790
C 0.883991 3.632126 0.751737
C 2.135861 4.369356 0.225529
H 2.274913 5.281819 0.820271
H 3.511129 -5.098899 0.266599
C 5.783635 -3.582066 0.459730
H 6.106289 -3.083140 1.381912
H 5.831653 -4.661891 0.627709
H 6.515281 -3.326838 -0.316056
H -2.684366 -0.842913 -1.049585
H -0.267950 -0.955109 0.890903
H 1.052772 3.331653 1.795372
C 3.409736 3.548669 0.267913
C 4.077796 3.329010 1.482460
C 3.950911 2.983270 -0.894877
C 5.246380 2.569006 1.534927
H 3.680098 3.765914 2.396301
C 5.122201 2.222124 -0.849699
H 3.453980 3.148223 -1.848279
C 5.774712 2.012368 0.366851
H 5.749833 2.417649 2.486436
H 5.528194 1.802839 -1.766904
H 6.692421 1.431000 0.403989
H -1.005894 0.680582 -1.433497
H 1.948412 4.696035 -0.805533
H 0.048001 4.345457 0.767905

72
-1859.982628
C -2.592393 -0.106630 -0.248765
C -4.209929 1.701289 0.575175
H -3.880015 1.509188 1.591515
C -5.170741 2.681560 0.323443
H -5.588621 3.248710 1.150979
C -5.594528 2.934756 -0.983524
H -6.343716 3.697631 -1.176974
C -5.053552 2.198539 -2.038794
H -5.380263 2.383521 -3.058586
C -4.096941 1.213358 -1.786198
H -3.685908 0.636862 -2.612313
C -3.662164 0.956504 -0.478987
N -2.782844 -0.850741 1.011826
C -3.726912 -2.011912 1.192286
C -4.268272 -2.505030 -0.155901
C -4.881950 -1.546424 2.099472
C -2.929044 -3.136183 1.878200
H -3.465437 -2.874591 -0.801044
H -4.830662 -1.728431 -0.684322
H -4.954829 -3.337071 0.032492
H -4.486418 -1.139748 3.034205
H -5.533828 -2.394615 2.334948
H -5.484143 -0.775273 1.608357
H -3.589186 -3.986453 2.080426
H -2.511481 -2.784216 2.824664
H -2.112849 -3.474414 1.232255
O -2.208397 -0.401732 2.074523
C -1.132472 0.455949 -0.369631
N -0.120447 -0.570535 -0.043490
S 0.183722 -1.797377 -1.156194
O -0.717356 -2.954673 -0.978505
O 0.231143 -1.146008 -2.474603
C 1.820918 -2.310555 -0.651085
C 2.864500 -1.378647 -0.625138
C 2.045395 -3.649556 -0.336190
C 4.138423 -1.801556 -0.264203
H 2.681910 -0.338799 -0.874183
C 3.332820 -4.055866 0.018872
H 1.223649 -4.356339 -0.366516
C 4.394215 -3.144338 0.063448
H 3.511242 -5.098900 0.266790
C -0.795301 1.733360 0.421232
H -1.630111 2.435496 0.321244
H -0.718117 1.489535 1.485197
C 0.493274 2.402000 -0.075161
H 0.366690 2.697690 -1.127633
H 1.311146 1.673154 -0.054429
C 0.884094 3.631923 0.751968
C 2.135915 4.369245 0.225771
H 2.274938 5.281680 0.820563
H 4.945677 -1.074351 -0.230214
C 5.783747 -3.581963 0.459378
H 6.515269 -3.326905 -0.316579
H 6.106546 -3.082789 1.381381
H 5.831809 -4.661741 0.627620
H -2.684417 -0.842944 -1.049499
H -0.268092 -0.955279 0.891031
H 1.052900 3.331443 1.795597
C 3.409837 3.548620 0.268060
C 4.077798 3.328686 1.482612
C 3.951163 2.983575 -0.894831
C 5.246436 2.568757 1.534985
H 3.679985 3.765317 2.396534
C 5.122510 2.222508 -0.849747
H 3.454310 3.148749 -1.848235
C 5.774924 2.012478 0.366809
H 5.749808 2.417180 2.486502
H 5.528621 1.803495 -1.767025
H 6.692674 1.431170 0.403877
H -1.005813 0.680455 -1.433410
H 1.948421 4.695974 -0.805266
H 0.048057 4.345200 0.768152
72
-1859.982628
C -2.592385 -0.106624 -0.248759
C -4.209970 1.701212 0.575263
H -3.880110 1.509017 1.591602
C -5.170769 2.681505 0.323567
H -5.588697 3.248577 1.151133
C -5.594483 2.934823 -0.983399
H -6.343660 3.697715 -1.176820
C -5.053446 2.198706 -2.038708
H -5.380098 2.383784 -3.058501
C -4.096849 1.213502 -1.786150
H -3.685768 0.637085 -2.612296
C -3.662146 0.956526 -0.478939
N -2.782889 -0.850845 1.011760
C -3.726958 -2.012037 1.192072
C -2.929136 -3.136333 1.878000
C -4.268184 -2.505089 -0.156193
C -4.882082 -1.546614 2.099178
H -2.511642 -2.784404 2.824508
H -2.112891 -3.474539 1.232101
H -3.589288 -3.986614 2.080142
H -3.465279 -2.874574 -0.801292
H -4.830562 -1.728479 -0.684610
H -4.954724 -3.337169 0.032089
H -4.486643 -1.139994 3.033977
H -5.533978 -2.394823 2.334539
H -5.484235 -0.775435 1.608057
O -2.208543 -0.401883 2.074531
C -1.132456 0.455964 -0.369509
N -0.120452 -0.570548 -0.043399
S 0.183812 -1.797268 -1.156206
O -0.717272 -2.954585 -0.978696
O 0.231321 -1.145758 -2.474544
C 1.820978 -2.310491 -0.651042
C 2.045412 -3.649501 -0.336172
C 2.864579 -1.378599 -0.624999
C 3.332813 -4.055849 0.018954
H 1.223661 -4.356274 -0.366564
C 4.138469 -1.801544 -0.264006
H 2.682015 -0.338740 -0.874016
C 4.394219 -3.144349 0.063610
H 4.945740 -1.074362 -0.229940
C -0.795305 1.733318 0.421456
H -1.630168 2.435414 0.321634
H -0.717991 1.489387 1.485388
C 0.493178 2.402072 -0.075019
H 0.366433 2.697907 -1.127430
H 1.311069 1.673239 -0.054511
C 0.884102 3.631890 0.752214
C 2.135865 4.369252 0.225935
H 2.274950 5.281652 0.820764
H 3.511186 -5.098894 0.266861
C 5.783737 -3.582021 0.459528
H 5.831640 -4.661722 0.628321
H 6.515151 -3.327521 -0.316721
H 6.106812 -3.082412 1.381192
H -2.684370 -0.842870 -1.049560
H -0.268158 -0.955392 0.891070
H 1.053041 3.331286 1.795785
C 3.409788 3.548621 0.268051
C 3.950874 2.983444 -0.894888
C 4.077985 3.328800 1.482493
C 5.122214 2.222358 -0.849957
H 3.453836 3.148528 -1.848212
C 5.246622 2.568858 1.534714
H 3.680360 3.765532 2.396448
C 5.774865 2.012443 0.366492
H 5.528135 1.803242 -1.767272
H 5.750182 2.417376 2.486147
H 6.692613 1.431124 0.403438
H -1.005756 0.680546 -1.433266
H 1.948265 4.696037 -0.805065
H 0.048073 4.345172 0.768587
72
-1859.982627

C -2.592356 -0.106606 -0.248740
C -4.209957 1.701242 0.575232
H -3.880143 1.509036 1.591584
C -5.170729 2.681552 0.323502
H -5.588687 3.248622 1.151054
C -5.594376 2.934893 -0.983481
H -6.343532 3.697798 -1.176929
C -5.053298 2.198781 -2.038773
H -5.379896 2.383877 -3.058579
C -4.096728 1.213559 -1.786180
H -3.685617 0.637146 -2.612313
C -3.662095 0.956560 -0.478951
N -2.782907 -0.850834 1.011765
C -3.727010 -2.012004 1.192047
C -4.268273 -2.505002 -0.156223
C -4.882119 -1.546581 2.099174
C -2.929231 -3.136354 1.877936
H -3.465393 -2.874499 -0.801345
H -4.830634 -1.728359 -0.684609
H -4.954838 -3.337064 0.032049
H -4.486662 -1.139993 3.033979
H -5.534030 -2.394783 2.334519
H -5.484260 -0.775379 1.608077
H -2.112999 -3.474566 1.232027
H -3.589415 -3.986619 2.080047
H -2.511726 -2.784474 2.824457
O -2.208569 -0.401897 2.074551
C -1.132430 0.455983 -0.369447
N -0.120419 -0.570530 -0.043347
S 0.183800 -1.797251 -1.156176
O -0.717333 -2.954535 -0.978693
O 0.231356 -1.145727 -2.474506
C 1.820932 -2.310544 -0.650981
C 2.045315 -3.649587 -0.336194
C 2.864562 -1.378696 -0.624879
C 3.332696 -4.056003 0.018901
H 1.223532 -4.356323 -0.366632
C 4.138441 -1.801711 -0.263912
H 2.682042 -0.338811 -0.873822
C 4.394140 -3.144539 0.063615
H 4.945736 -1.074559 -0.229804
C -0.795265 1.733308 0.421565
H -1.630158 2.435384 0.321865
H -0.717849 1.489315 1.485475
C 0.493154 2.402128 -0.074985
H 0.366281 2.698080 -1.127348
H 1.311055 1.673301 -0.054652
C 0.884152 3.631856 0.752342
C 2.135884 4.369279 0.226068
H 2.274968 5.281630 0.820973
H 3.511035 -5.099070 0.266740
C 5.783629 -3.582310 0.459528
H 6.515111 -3.327582 -0.316580
H 6.106613 -3.082971 1.381370
H 5.831520 -4.662058 0.628020
H -2.684328 -0.842845 -1.049548
H -0.268145 -0.955393 0.891113
H 1.053151 3.331148 1.795874
C 3.409821 3.548671 0.268072
C 4.078195 3.328961 1.482435
C 3.950748 2.983403 -0.894899
C 5.246845 2.569032 1.534553
H 3.680699 3.765772 2.396408
C 5.122100 2.222334 -0.850071
H 3.453578 3.148410 -1.848167
C 5.774926 2.012524 0.366303
H 5.750543 2.417636 2.485926
H 5.527900 1.803156 -1.767412
H 6.692683 1.431216 0.403167
H -1.005710 0.680599 -1.433196
H 1.948240 4.696151 -0.804897
H 0.048131 4.345143 0.768836
72
-1859.982627
C -2.592367 -0.106639 -0.248770
C -4.210033 1.701082 0.575369
H -3.880213 1.508788 1.591703
C -5.170841 2.681379 0.323730
H -5.588817 3.248360 1.151333
C -5.594500 2.934822 -0.983232
H -6.343686 3.697717 -1.176609
C -5.053397 2.198827 -2.038590
H -5.380003 2.384001 -3.058380
C -4.096789 1.213616 -1.786087
H -3.685656 0.637296 -2.612276
C -3.662145 0.956513 -0.478884
N -2.782885 -0.850994 1.011654
C -3.726908 -2.012233 1.191816
C -4.882144 -1.546931 2.098849
C -2.929104 -3.136537 1.877749
C -4.267987 -2.505234 -0.156529
H -5.534024 -2.395189 2.334077
H -5.484282 -0.775739 1.607733
H -4.486808 -1.140370 3.033717
H -2.112796 -3.474683 1.231898
H -3.589245 -3.986852 2.079789
H -2.511696 -2.784651 2.824310
H -4.830400 -1.728632 -0.684924
H -4.954462 -3.337393 0.031635
H -3.464994 -2.874584 -0.801594
O -2.208676 -0.402047 2.074511
C -1.132460 0.456022 -0.369408
N -0.120401 -0.570459 -0.043362
S 0.183923 -1.797097 -1.156262
O -0.717152 -2.954440 -0.978912
O 0.231526 -1.145480 -2.474533
C 1.821054 -2.310355 -0.651020
C 2.864671 -1.378469 -0.624912
C 2.045444 -3.649364 -0.336156
C 4.138526 -1.801441 -0.263855
H 2.682131 -0.338606 -0.873936
C 3.332825 -4.055745 0.019033
H 1.223687 -4.356129 -0.366591
C 4.394235 -3.144263 0.063760
H 3.511161 -5.098800 0.266922
C -0.795380 1.733310 0.421701
H -1.630318 2.435341 0.322046
H -0.717959 1.489250 1.485595
C 0.492999 2.402226 -0.074815
H 0.366053 2.698349 -1.127122
H 1.310908 1.673402 -0.054653
C 0.884069 3.631841 0.752656
C 2.135792 4.369257 0.226359
H 2.274998 5.281539 0.821341
H 4.945819 -1.074289 -0.229720
C 5.783719 -3.581949 0.459782
H 6.106539 -3.082687 1.381725
H 5.831699 -4.661714 0.628154
H 6.515270 -3.327054 -0.316206
H -2.684302 -0.842792 -1.049661
H -0.268070 -0.955373 0.891082
H 1.053126 3.330995 1.796137
C 3.409696 3.548575 0.268149
C 3.950606 2.983590 -0.894964
C 4.078055 3.328524 1.482461
C 5.121935 2.222467 -0.850327
H 3.453445 3.148856 -1.848192
C 5.246678 2.568542 1.534388
H 3.680566 3.765104 2.396548
C 5.774747 2.012319 0.365995
H 5.527727 1.803515 -1.767774
H 5.750366 2.416881 2.485724
H 6.692488 1.430975 0.402708
H -1.005726 0.680724 -1.433135
H 1.948065 4.696245 -0.804555
H 0.048073 4.345154 0.769286
72
-1859.982627
C -2.592378 -0.106616 -0.248764
C -4.209982 1.701132 0.575384

H -3.880087 1.508898 1.591704
C -5.170815 2.681413 0.323766
H -5.588720 3.248445 1.151370
C -5.594585 2.934770 -0.983173
H -6.343786 3.697652 -1.176539
C -5.053573 2.198706 -2.038532
H -5.380277 2.383814 -3.058302
C -4.096940 1.213520 -1.786051
H -3.685871 0.637146 -2.612234
C -3.662180 0.956503 -0.478867
N -2.782838 -0.850919 1.011703
C -3.726854 -2.012166 1.191935
C -4.881984 -1.546858 2.099087
C -2.928958 -3.136472 1.877759
C -4.268053 -2.505123 -0.156375
H -5.533834 -2.395115 2.334401
H -5.484182 -0.775679 1.608021
H -4.486556 -1.140277 3.033907
H -2.112704 -3.474575 1.231817
H -3.589058 -3.986809 2.079833
H -2.511465 -2.784607 2.824291
H -4.830499 -1.728505 -0.684710
H -4.954523 -3.337278 0.031829
H -3.465123 -2.874476 -0.801518
O -2.208517 -0.401995 2.074506
C -1.132461 0.456029 -0.369477
N -0.120429 -0.570463 -0.043414
S 0.183896 -1.797094 -1.156307
O -0.717235 -2.954402 -0.978994
O 0.231554 -1.145481 -2.474584
C 1.820996 -2.310415 -0.651027
C 2.045333 -3.649467 -0.336261
C 2.864645 -1.378587 -0.624819
C 3.332678 -4.055919 0.018939
H 1.223557 -4.356204 -0.366794
C 4.138483 -1.801636 -0.263757
H 2.682162 -0.338695 -0.873762
C 4.394132 -3.144480 0.063767
H 4.945796 -1.074506 -0.229582
C -0.795327 1.733344 0.421550
H -1.630233 2.435406 0.321861
H -0.717903 1.489333 1.485456
C 0.493090 2.402180 -0.074980
H 0.366233 2.698143 -1.127340
H 1.310997 1.673358 -0.054648
C 0.884086 3.631891 0.752378
C 2.135826 4.369310 0.226129
H 2.274934 5.281630 0.821078
H 3.510966 -5.099000 0.266757
C 5.783578 -3.582280 0.459793
H 6.515175 -3.327308 -0.316126
H 6.106373 -3.083168 1.381823
H 5.831502 -4.662071 0.628016
H -2.684328 -0.842818 -1.049613
H -0.268124 -0.955376 0.891026
H 1.053073 3.331152 1.795903
C 3.409749 3.548676 0.268082
C 3.950763 2.983634 -0.894957
C 4.078018 3.328715 1.482457
C 5.122100 2.222538 -0.850185
H 3.453659 3.148816 -1.848229
C 5.246657 2.568767 1.534517
H 3.680447 3.765343 2.396484
C 5.774828 2.012487 0.366198
H 5.527960 1.803524 -1.767572
H 5.750279 2.417175 2.485899
H 6.692578 1.431165 0.403022
H -1.005770 0.680662 -1.433226
H 1.948178 4.696239 -0.804816
H 0.048074 4.345188 0.768884
72
-1859.982627
C -2.592313 -0.106663 -0.248759
C -4.210036 1.700923 0.575544
H -3.880043 1.508709 1.591836
C -5.170988 2.681107 0.324013
H -5.588902 3.248072 1.151659
C -5.594874 2.934453 -0.982893
H -6.344171 3.697259 -1.176187
C -5.053854 2.198472 -2.038305
H -5.380639 2.383572 -3.058051
C -4.097103 1.213375 -1.785910
H -3.686038 0.637062 -2.612137
C -3.662227 0.956374 -0.478762
N -2.782585 -0.850956 1.011752
C -3.726495 -2.012271 1.192131
C -4.881464 -1.547068 2.099549
C -2.928416 -3.136574 1.877748
C -4.267981 -2.505205 -0.156071
H -5.533232 -2.395365 2.334946
H -5.483792 -0.775883 1.608653
H -4.485857 -1.140527 3.034310
H -2.112297 -3.474645 1.231623
H -3.588448 -3.986931 2.079969
H -2.510727 -2.784703 2.824193
H -4.954438 -3.337340 0.032268
H -3.465200 -2.874583 -0.801384
H -4.830508 -1.728562 -0.684283
O -2.208115 -0.402008 2.074465
C -1.132464 0.456104 -0.369661
N -0.120292 -0.570285 -0.043654
S 0.183989 -1.796941 -1.156538
O -0.717239 -2.954198 -0.979312
O 0.231754 -1.145319 -2.474810
C 1.821009 -2.310385 -0.651119
C 2.045287 -3.649525 -0.336655
C 2.864667 -1.378583 -0.624561
C 3.332576 -4.056073 0.018612
H 1.223496 -4.356232 -0.367488
C 4.138459 -1.801735 -0.263431
H 2.682249 -0.338635 -0.873318
C 4.394037 -3.144646 0.063835
H 4.945782 -1.074627 -0.229019
C -0.795388 1.733494 0.421282
H -1.630324 2.435509 0.321511
H -0.717982 1.489565 1.485207
C 0.493004 2.402356 -0.075278
H 0.366153 2.698217 -1.127669
H 1.310942 1.673575 -0.054867
C 0.883924 3.632172 0.751960
C 2.135706 4.369523 0.225697
H 2.274778 5.281937 0.820505
H 3.510837 -5.099220 0.266169
C 5.783342 -3.582536 0.460268
H 5.831948 -4.662820 0.625092
H 6.515785 -3.324393 -0.313782
H 6.104435 -3.086184 1.384397
H -2.684316 -0.842887 -1.049577
H -0.267902 -0.955179 0.890812
H 1.052862 3.331567 1.795530
C 3.409612 3.548873 0.267890
C 3.950508 2.983287 -0.894940
C 4.077955 3.329403 1.482313
C 5.121800 2.222135 -0.849915
H 3.453359 3.148101 -1.848252
C 5.246550 2.569401 1.534626
H 3.680476 3.766458 2.396177
C 5.774596 2.012566 0.366517
H 5.527568 1.802689 -1.767146
H 5.750230 2.418198 2.486040
H 6.692308 1.431195 0.403528
H -1.005889 0.680714 -1.433429
H 1.948136 4.696272 -0.805321
H 0.047899 4.345458 0.768324
72
-1859.982627
C -2.592377 -0.106659 -0.248736
C -4.209981 1.701164 0.575280
H -3.880089 1.509005 1.591616
C -5.170804 2.681432 0.323584

H -5.588725 3.248515 1.151146
C -5.594553 2.934711 -0.983379
H -6.343750 3.697583 -1.176800
C -5.053524 2.198582 -2.038683
H -5.380201 2.383631 -3.058473
C -4.096901 1.213403 -1.786123
H -3.685827 0.636976 -2.612266
C -3.662167 0.956464 -0.478916
N -2.782841 -0.850850 1.011807
C -3.726861 -2.012071 1.192172
C -4.268209 -2.505110 -0.156049
C -4.881914 -1.546697 2.099396
C -2.928955 -3.136362 1.878008
H -3.465368 -2.874603 -0.801224
H -4.830624 -1.728491 -0.684412
H -4.954740 -3.337185 0.032288
H -5.533760 -2.394930 2.334808
H -5.484136 -0.775532 1.608338
H -4.486398 -1.140075 3.034160
H -2.112764 -3.474545 1.232032
H -3.589074 -3.986656 2.080205
H -2.511380 -2.784435 2.824483
O -2.208439 -0.401884 2.074546
C -1.132471 0.455974 -0.369549
N -0.120408 -0.570485 -0.043433
S 0.183847 -1.797214 -1.156249
O -0.717262 -2.954516 -0.978766
O 0.231378 -1.145699 -2.474582
C 1.820997 -2.310462 -0.651059
C 2.864577 -1.378562 -0.624856
C 2.045450 -3.649522 -0.336392
C 4.138470 -1.801538 -0.263888
H 2.682016 -0.338672 -0.873748
C 3.332843 -4.055899 0.018705
H 1.223714 -4.356307 -0.366942
C 4.394230 -3.144375 0.063555
H 3.511245 -5.098983 0.266425
C -0.795345 1.733375 0.421354
H -1.630229 2.435443 0.321507
H -0.718011 1.489489 1.485294
C 0.493116 2.402140 -0.075163
H 0.366351 2.697936 -1.127583
H 1.311020 1.673325 -0.054643
C 0.884026 3.631996 0.752021
C 2.135832 4.369309 0.225773
H 2.274897 5.281736 0.820566
H 4.945730 -1.074350 -0.229730
C 5.783692 -3.582040 0.459677
H 5.832127 -4.662072 0.626184
H 6.515629 -3.325347 -0.315340
H 6.105618 -3.084260 1.382744
H -2.684368 -0.842929 -1.049513
H -0.268114 -0.955336 0.891034
H 1.052906 3.331446 1.795617
C 3.409744 3.548669 0.268000
C 4.077977 3.329044 1.482455
C 3.950776 2.983271 -0.894860
C 5.246594 2.569077 1.534769
H 3.680394 3.765945 2.396347
C 5.122094 2.222160 -0.849834
H 3.453712 3.148202 -1.848196
C 5.774780 2.012438 0.366630
H 5.750181 2.417748 2.486212
H 5.527971 1.802872 -1.767090
H 6.692510 1.431097 0.403649
H -1.005805 0.680520 -1.433319
H 1.948291 4.696046 -0.805253
H 0.048011 4.345296 0.768309
72
-1859.982595
C 2.423517 0.266574 0.316308
C 3.270663 2.658978 -0.040924
H 3.457366 2.420050 -1.083260
C 3.544798 3.937754 0.445721
H 3.948586 4.691906 -0.224635
C 3.302632 4.250054 1.785786
H 3.517879 5.246312 2.162416
C 2.787920 3.272863 2.639240
H 2.602189 3.503040 3.684967
C 2.520378 1.991321 2.153972
H 2.130016 1.230885 2.827089
C 2.753448 1.671253 0.809504
N 3.363245 -0.213509 -0.714851
C 4.678933 -0.892776 -0.435006
C 5.804654 0.065355 -0.869200
C 4.712774 -2.176441 -1.285577
C 4.820900 -1.243419 1.051952
H 5.669165 0.357201 -1.914052
H 6.775861 -0.430722 -0.767125
H 5.812245 0.969146 -0.251135
H 4.593612 -1.935059 -2.344546
H 3.912215 -2.864360 -0.992777
H 5.672517 -2.685471 -1.147277
H 5.792884 -1.725077 1.200802
H 4.048251 -1.944513 1.383206
H 4.793457 -0.353377 1.689048
O 3.107569 0.066755 -1.946691
C 0.933649 0.099613 -0.158325
N 0.719911 -1.216294 -0.788798
S 0.581858 -2.609210 0.132913
O 1.074271 -3.691960 -0.732227
O 1.159981 -2.411853 1.474869
C -1.185918 -2.801679 0.352442
C -1.737529 -2.704921 1.628535
C -1.988726 -3.075617 -0.759345
C -3.113300 -2.878172 1.788631
H -1.097092 -2.500038 2.479118
C -3.357618 -3.245559 -0.580662
H -1.547380 -3.156371 -1.747467
C -3.942298 -3.152383 0.694133
H -3.984769 -3.459215 -1.442592
C 0.367543 1.191615 -1.082742
H 0.628264 2.167926 -0.659447
H 0.864593 1.129611 -2.055989
C -1.155005 1.101118 -1.246489
H -1.633699 1.174336 -0.260246
H -1.421503 0.114082 -1.645623
C -1.714670 2.190442 -2.169062
C -3.241669 2.103794 -2.391132
H -3.486445 1.110551 -2.789738
H -3.546049 -2.800905 2.782708
C -5.424830 -3.372627 0.876563
H -5.999025 -2.936826 0.052020
H -5.660093 -4.444659 0.902989
H -5.783048 -2.935136 1.813473
H 2.523478 -0.416234 1.159087
H 1.340677 -1.384016 -1.580045
H -1.220613 2.123073 -3.148391
C -4.067098 2.367681 -1.147830
C -4.601654 1.317166 -0.389128
C -4.302778 3.681712 -0.715963
C -5.345299 1.568921 0.766234
H -4.432472 0.290523 -0.706701
C -5.045035 3.939254 0.437028
H -3.903810 4.512225 -1.295125
C -5.569993 2.881609 1.183850
H -5.750944 0.737930 1.337933
H -5.218511 4.965890 0.749734
H -6.151571 3.079927 2.080290
H 0.342218 0.108574 0.764489
H -3.519459 2.828324 -3.167649
H -1.465719 3.182019 -1.765312
72
-1859.982574
C 2.428971 0.263364 0.317278
C 3.281600 2.657314 -0.015189
H 3.468301 2.428449 -1.059772
C 3.558282 3.930506 0.484480
H 3.964031 4.690522 -0.178021
C 3.316164 4.229749 1.827529

H 3.533389 5.221676 2.214317
C 2.798970 3.245071 2.670831
H 2.613298 3.465048 3.718763
C 2.528885 1.969089 2.172496
H 2.136610 1.202698 2.837716
C 2.761830 1.662176 0.824940
N 3.367723 -0.207932 -0.718881
C 4.682248 -0.892394 -0.445953
C 4.712944 -2.168619 -1.307753
C 4.824577 -1.256623 1.037689
C 5.809571 0.067260 -0.872520
H 4.591882 -1.918034 -2.364361
H 3.912385 -2.858329 -1.019147
H 5.672384 -2.679910 -1.175831
H 4.798715 -0.372330 1.682799
H 5.796023 -1.740882 1.181557
H 4.051110 -1.959648 1.362787
H 5.674044 0.368541 -1.914679
H 6.779846 -0.431620 -0.775389
H 5.819341 0.965569 -0.246542
O 3.112551 0.085195 -1.947839
C 0.938710 0.104850 -0.159136
N 0.721501 -1.203425 -0.804297
S 0.581971 -2.605822 0.102655
O 1.049463 -3.682388 -0.783682
O 1.182683 -2.434362 1.438391
C -1.184760 -2.776911 0.346928
C -1.711613 -2.714287 1.635098
C -2.010983 -3.009390 -0.757708
C -3.086623 -2.876964 1.815455
H -1.052820 -2.547816 2.480002
C -3.377901 -3.169418 -0.558930
H -1.587845 -3.070709 -1.755144
C -3.939032 -3.102868 0.728638
H -4.022137 -3.356720 -1.414475
C 0.375670 1.209220 -1.070784
H 0.631121 2.179417 -0.630489
H 0.879440 1.163458 -2.041459
C -1.145405 1.117334 -1.246710
H -1.631224 1.166471 -0.262498
H -1.405247 0.138467 -1.669470
C -1.703146 2.224777 -2.148690
C -3.225722 2.127976 -2.396142
H -3.452486 1.144248 -2.827721
H -3.499376 -2.830744 2.819827
C -5.427740 -3.262275 0.924057
H -5.815175 -4.114654 0.354903
H -5.679630 -3.413288 1.977880
H -5.968096 -2.371980 0.576971
H 2.527695 -0.428431 1.152782
H 1.341668 -1.363857 -1.597598
H -1.194526 2.187981 -3.122185
C -4.073121 2.345020 -1.158624
C -4.347168 3.643056 -0.701974
C -4.590554 1.265357 -0.429373
C -5.110143 3.857067 0.446486
H -3.962015 4.495562 -1.258064
C -5.354443 1.473491 0.721355
H -4.391227 0.250379 -0.766404
C -5.617573 2.770880 1.163773
H -5.313348 4.872076 0.778615
H -5.745620 0.620489 1.270505
H -6.215084 2.935239 2.056586
H 0.346883 0.104932 0.763567
H -3.500399 2.872000 -3.155046
H -1.470564 3.207971 -1.715756

S12. Kinetic modeling

The photochemical initiation reaction is an extremely fast process and is considered to occur instantaneously. This corresponds to the formation of the **N-rad** by photochemical cleavage of the **N-Cl** bond. The produced N radical can then undergo either of the following reactions to form **C_5-rad** or **C_6-rad** radicals, as shown in eq 1 and eq 2.

$$\text{N-rad} \underset{k_{1b}}{\overset{k_{1f}}{\rightleftharpoons}} \text{C}_5\text{-rad} \tag{1}$$

$$\text{N-rad} \underset{k_{2b}}{\overset{k_{2f}}{\rightleftharpoons}} \text{C}_6\text{-rad} \tag{2}$$

The reaction rate coefficients are shown above and below the reaction arrows. The notation k_{xy} is used where *x* presents the reaction number and *y* indicates the direction, either forward (denoted by 'f') or backward (denoted by 'b'). The radicals **C_5-rad** and **C_6-rad** react with **N-Cl** to generate the final products while regenerating the **N-rad** in the process, as shown in eqs 3-5.

$$\text{C}_5\text{-rad} + \text{N-Cl} \underset{k_{3b}}{\overset{k_{3f}}{\rightleftharpoons}} \text{C}_5\text{-Cl} + \text{N-rad} \tag{3}$$

$$\text{C}_6\text{-rad} + \text{N-Cl} \underset{k_{4b}}{\overset{k_{4f}}{\rightleftharpoons}} \text{C}_2\text{-Cl} + \text{N-rad} \tag{4}$$

$$\text{C}_6\text{-rad} + \text{N-Cl} \underset{k_{5b}}{\overset{k_{5f}}{\rightleftharpoons}} \text{C}_6\text{-Cl} + \text{N-rad} \tag{5}$$

For these reactions, a set of differential equations describing the temporal concentration changes of each reaction constituent is provided in eqs 6-12

$$\frac{d[\text{N-rad}]}{dt} = -(k_{1f} + k_{2f} + k_{3b}[\text{C}_5\text{-Cl}] + k_{4b}[\text{C}_2\text{-Cl}] + k_{5b}[\text{C}_6\text{-Cl}])[\text{N-rad}] + (k_{3f}[\text{C}_5\text{-rad}] + k_{4f}[\text{C}_6\text{-rad}] + k_{5f}[\text{C}_6\text{-rad}])[\text{N-Cl}] + k_{1b}[\text{C}_5\text{-rad}] + k_{2b}[\text{C}_6\text{-rad}] \quad (6)$$

$$\frac{d[\text{C}_5\text{-rad}]}{dt} = -[\text{C}_5\text{-rad}](k_{3f}[\text{N-Cl}] + k_{1b}) + [\text{N-rad}](k_{3b}[\text{C}_5\text{-Cl}] + k_{1f}) \quad (7)$$

$$\frac{d[\text{C}_6\text{-rad}]}{dt} = -[\text{C}_6\text{-rad}](k_{4f}[\text{N-Cl}] + k_{5f}[\text{N-Cl}] + k_{2b}) + [\text{N-rad}](k_{4b}[\text{C}_2\text{-Cl}] + k_{5b}[\text{C}_6\text{-Cl}] + k_{2f}) \quad (8)$$

$$\frac{d[\text{N-Cl}]}{dt} = -[\text{N-Cl}](k_{3f}[\text{C}_5\text{-rad}] + k_{4f}[\text{C}_6\text{-rad}] + k_{5f}[\text{C}_6\text{-rad}]) + [\text{N-rad}](k_{3b}[\text{C}_5\text{-Cl}] + k_{4b}[\text{C}_2\text{-Cl}] + k_{5b}[\text{C}_6\text{-Cl}]) \quad (9)$$

$$\frac{d[\text{C}_5\text{-Cl}]}{dt} = k_{3f}[\text{C}_5\text{-rad}][\text{N-Cl}] - k_{3b}[\text{C}_5\text{-Cl}][\text{N-rad}] \quad (10)$$

$$\frac{d[\text{C}_2\text{-Cl}]}{dt} = k_{4f}[\text{C}_6\text{-rad}][\text{N-Cl}] - k_{4b}[\text{C}_2\text{-Cl}][\text{N-rad}] \quad (11)$$

$$\frac{d[\text{C}_6\text{-Cl}]}{dt} = k_{5f}[\text{C}_6\text{-rad}][\text{N-Cl}] - k_{5b}[\text{C}_6\text{-Cl}][\text{N-rad}] \quad (12)$$

Steady-state approximation

Without further assumptions, the given system of differential equations is exceedingly complicated and likely impossible to solve analytically. This complexity arises because the equations are nonlinear, with each species' concentration explicitly depending on the concentrations of others, thus creating interdependencies.[34–37] In addition, any attempts to numerically solve the system of differential equations using methods like Runge-Kutta

or other iterative approaches may prove challenging due to uncertainty in the amount of radicals formed (which serve as the initial condition in the model).[38,39] This uncertainty in the initial conditions can generally contribute to numerical instability.[36,40,41]

In chemical reaction systems, faulty or poorly defined initial conditions may lead to non-unique solutions. This may occur when the differential equations governing the chemical reactions do not satisfy the Lipschitz criterion, particularly when the system has a large Lipschitz constant, leading to increased sensitivity to initial conditions.[36,42] Even if the initial concentration of **N-Cl** is very precisely determined, accurate estimation of the number of radical species generated after the laser pulse is difficult, if not impossible.

These complexities make finding an explicit analytical solution highly challenging, if not impossible, without simplifications. A reasonable assumption is that the **N-rad** achieves a steady-state, as it is regenerated after each reaction cycle. Interestingly, the regenerated radical at the end of a reaction cycle may be viewed as acting similarly to a catalyst, continuously facilitating the reaction without being consumed (under the assumption of insignificant secondary reactions). Graph theory, specifically the concept of spanning trees, can potentially be useful for solving the kinetics of catalytic networks in a steady-state regime, which bears resemblance to the hereby-presented issue of regenerated radicals.[43] With all things considered, it can be assumed that the concentration of **N-rad** remains approximately constant throughout the course of the reaction. It should be emphasized that smaller radicals are known as excellent examples of steady-state intermediates, particularly when they disappear much faster than they are formed, according to J.M. Perez-Benito.[44] However, the assumed steady-state of radicals may be challenging to confirm experimentally without the use of a spin-trap due to their rapid reactivity and short lifetimes.[45]

Therefore, for the studied system, the steady-state approximation can be expressed *via* eq 13, as showcased below.

$$\frac{d\text{N-rad}}{dt} = 0 = -(k_{1\text{f}} + k_{2\text{f}} + k_{3\text{b}}[\text{C}_5\text{-Cl}] + k_{4\text{b}}[\text{C}_2\text{-Cl}] + k_{5\text{b}}[\text{C}_6\text{-Cl}])[\text{N}] + (k_{3\text{f}}[\text{C}_5\text{-rad}] + k_{4\text{f}}[\text{C}_6\text{-rad}] + k_{5\text{f}}[\text{C}_6\text{-rad}])[\text{N-Cl}] + k_{1\text{b}}[\text{C}_5\text{-rad}] + k_{2\text{b}}[\text{C}_6\text{-rad}] \quad (13)$$

The expression for the concentration of **N-rad** (eq 14) can be obtained by rearranging the previous equation.

$$[\text{N-rad}] = \frac{(k_{3\text{f}}[\text{C}_5\text{-rad}]+k_{4\text{f}}[\text{C}_6\text{-rad}] + k_{5\text{f}}[\text{C}_6\text{-rad}])[\text{N-Cl}]+k_{1\text{b}}[\text{C}_5\text{-rad}]+k_{2\text{b}}[\text{C}_6\text{-rad}]}{k_{1\text{f}}+k_{2\text{f}}+k_{3\text{b}}[\text{C}_5\text{-Cl}]+k_{4\text{b}}[\text{C}_2\text{-Cl}]+k_{5\text{b}}[\text{C}_6\text{-Cl}]} \quad (14)$$

Substituting eq 14 into eqs 7-12 yields the following set of differential equations, which are provided below.

$$\frac{d[\text{C}_5\text{-rad}]}{dt} = -[\text{C}_5\text{-rad}](k_{3\text{f}}[\text{N-Cl}] + k_{1\text{b}}) + \frac{(k_{3\text{f}}[\text{C}_5\text{-rad}]+k_{4\text{f}}[\text{C}_6\text{-rad}] + k_{5\text{f}}[\text{C}_6\text{-rad}])[\text{N-Cl}]+k_{1\text{b}}[\text{C}_5\text{-rad}]+k_{2\text{b}}[\text{C}_6\text{-rad}]}{k_{1\text{f}}+k_{2\text{f}}+k_{3\text{b}}[\text{C}_5\text{-Cl}]+k_{4\text{b}}[\text{C}_2\text{-Cl}]+k_{5\text{b}}[\text{C}_6\text{-Cl}]}(k_{3\text{b}}[\text{C}_5\text{-Cl}] + k_{1\text{f}}) \quad (15)$$

$$\frac{d[\text{C}_6\text{-rad}]}{dt} = -[\text{C}_6\text{-rad}](k_{4\text{f}}[\text{N-Cl}] + k_{5\text{f}}[\text{N-Cl}] + k_{2\text{b}}) + \frac{(k_{3\text{f}}[\text{C}_5\text{-rad}]+k_{4\text{f}}[\text{C}_6\text{-rad}] + k_{5\text{f}}[\text{C}_6\text{-rad}])[\text{N-Cl}]+k_{1\text{b}}[\text{C}_5\text{-rad}]+k_{2\text{b}}[\text{C}_6\text{-rad}]}{k_{1\text{f}}+k_{2\text{f}}+k_{3\text{b}}[\text{C}_5\text{-Cl}]+k_{4\text{b}}[\text{C}_2\text{-Cll}]+k_{5\text{b}}[\text{C}_6\text{-Cl}]}(k_{4\text{b}}[\text{C}_2\text{-Cl}] + k_{5\text{b}}[\text{C}_6\text{-Cl}] + k_{2\text{f}}) \quad (16)$$

$$\frac{d[\text{N-Cl}]}{dt} = -[\text{N-Cl}](k_{3\text{f}}[\text{C}_5\text{-rad}] + k_{4\text{f}}[\text{C}_6\text{-rad}] + k_{5\text{f}}[\text{C}_6\text{-rad}]) + \frac{(k_{3\text{f}}[\text{C}_5\text{-rad}]+k_{4\text{f}}[\text{C}_6\text{-rad}] + k_{5\text{f}}[\text{C}_6\text{-rad}])[\text{N-Cl}]+k_{1\text{b}}[\text{C}_5\text{-rad}]+k_{2\text{b}}[\text{C}_6\text{-rad}]}{k_{1\text{f}}+k_{2\text{f}}+k_{3\text{b}}[\text{C}_5\text{-Cl}]+k_{4\text{b}}[\text{C}_2\text{-Cl}]+k_{5\text{b}}[\text{C}_6\text{-Cl}]}(k_{3\text{b}}[\text{C}_5\text{-Cl}] + k_{4\text{b}}[\text{C}_2\text{-Cl}] + k_{5\text{b}}[\text{C}_6\text{-Cl}]) \quad (17)$$

$$\frac{d[\text{C}_5\text{-Cl}]}{dt} = k_{3\text{f}}[\text{C}_5\text{-rad}][\text{N-Cl}] - (k_{3\text{b}}[\text{C}_5\text{-Cll}])\frac{(k_{3\text{f}}[\text{C}_5\text{-rad}]+k_{4\text{f}}[\text{C}_6\text{-rad}] + k_{5\text{f}}[\text{C}_6\text{-rad}])[\text{N-Cl}]+k_{1\text{b}}[\text{C}_5\text{-rad}]+k_{2\text{b}}[\text{C}_6\text{-rad}]}{k_{1\text{f}}+k_{2\text{f}}+k_{3\text{b}}[\text{C}_5\text{-Cl}]+k_{4\text{b}}[\text{C}_2\text{-Cl}]+k_{5\text{b}}[\text{C}_6\text{-Cl}]} \quad (18)$$

$$\frac{d[\text{C}_2\text{-Cl}]}{dt} = k_{4\text{f}}[\text{C}_6\text{-rad}][\text{N-Cl}] - (k_{4\text{b}}[\text{C}_2\text{-Cl}])\frac{(k_{3\text{f}}[\text{C}_5\text{-rad}]+k_{4\text{f}}[\text{C}_6\text{-rad}] + k_{5\text{f}}[\text{C}_6\text{-rad}])[\text{N-Cl}]+k_{1\text{b}}[\text{C}_5\text{-rad}]+k_{2\text{b}}[\text{C}_6\text{-rad}]}{k_{1\text{f}}+k_{2\text{f}}+k_{3\text{b}}[\text{C}_5\text{-Cl}]+k_{4\text{b}}[\text{C}_2\text{-Cl}]+k_{5\text{b}}[\text{C}_6\text{-Cl}]} \quad (19)$$

$$\frac{d[\text{C}_6\text{-Cl}]}{dt} = k_{5\text{f}}[\text{C}_6\text{-rad}][\text{N-Cl}] - (k_{5\text{b}}[\text{C}_6\text{-Cl}])\frac{(k_{3\text{f}}[\text{C}_5\text{-rad}]+k_{4\text{f}}[\text{C}_6\text{-rad}] + k_{5\text{f}}[\text{C}_6\text{-rad}])[\text{N-Cl}]+k_{1\text{b}}[\text{C}_5\text{-rad}]+k_{2\text{b}}[\text{C}_6\text{-rad}]}{k_{1\text{f}}+k_{2\text{f}}+k_{3\text{b}}[\text{C}_5\text{-Cl}]+k_{4\text{b}}[\text{C}_2\text{-Cl}]+k_{5\text{b}}[\text{C}_6\text{-Cl}]} \quad (20)$$

Additional approximations

Considering that **C_6-Cl** accounts for over 99% of the final product, its reaction pathway is most likely the predominant factor in the consumption and production of N radicals. Therefore, it may be reasonable to assume that secondary pathways have only a minor influence on the relative change in **N-Cl** reactant concentration over time. Moreover, for all reactions involving the formation of chlorinated products from **C_5-rad** or **C_6-rad** radicals (eqs 3-5), it can be assumed that only the forward reactions occur significantly, while the backward reactions are negligible. This approximation is necessary to obtain a system of differential equations that can be solved analytically. We consider this approach reasonable, as quantum chemical calculations indicate that the backward reactions are much less likely to occur due to significantly higher energy barriers (please see reaction coordinate diagrams provided in Figures 5-7). Therefore, disregarding these backward reactions should not introduce significant errors into the model, while simplifying it substantially. The simplified model, considering only the **C_6-Cl** product formation, is given in the equations below.

$$\text{N}-\text{rad} \underset{k_{2\text{b}}}{\overset{k_{2\text{f}}}{\rightleftharpoons}} \text{C}_6\text{-rad} \tag{21}$$

$$\text{C}_6\text{-rad} + \text{N-Cl} \xrightarrow{k_{5\text{f}}} \text{C}_6\text{-Cl} + \text{N-rad} \tag{22}$$

The simplified model is described by the set of differential equations (eqs 23-26) that are shown below.

$$\frac{d[\text{N-rad}]}{dt} = -k_{2\text{f}}[\text{N-rad}] + k_{2\text{b}}[\text{C}_6\text{-rad}] + k_{5\text{f}}[\text{C}_6\text{-rad}][\text{N-Cl}] \tag{23}$$

$$\frac{d[\text{C}_6\text{-rad}]}{dt} = k_{2\text{f}}[\text{N-rad}] - k_{2\text{b}}[\text{C}_6\text{-rad}] - k_{5\text{f}}[\text{C}_6\text{-rad}][\text{N-Cl}] \tag{24}$$

$$\frac{d[\text{C}_6\text{-Cl}]}{dt} = k_{5\text{f}}[\text{C}_6\text{-rad}][\text{N-Cl}] \tag{25}$$

$$\frac{d[\text{N-Cl}]}{dt} = -k_{5\text{f}}[\text{C}_6\text{-rad}][\text{N-Cl}] \tag{26}$$

Steady-state condition is approximated as given in eq 27.

$$\frac{d[\text{N-rad}]}{dt} = 0 = -k_{2\text{f}}[\text{N-rad}] + k_{2\text{b}}[\text{C}_6\text{-rad}] + k_{5\text{f}}[\text{C}_6\text{-rad}][\text{N-Cl}] \tag{27}$$

The [N-rad] can be expressed as shown in eq 28.

$$[\text{N-rad}] = \frac{k_{2\text{b}}[\text{C}_6\text{-rad}] + k_{5\text{f}}[\text{C}_6\text{-rad}][\text{N-Cl}]}{k_{2\text{f}}} \tag{28}$$

Substituting the [N-rad] in the eq 24 provides the following expression.

$$\frac{d[\text{C}_6\text{-rad}]}{dt} = k_{2\text{f}}\left(\frac{k_{2\text{b}}[\text{C}_6\text{-rad}] + k_{5\text{f}}[\text{C}_6\text{-rad}][\text{N-Cl}]}{k_{2\text{f}}}\right) - k_{2\text{b}}[\text{C}_6\text{-rad}] - k_{5\text{f}}[\text{C}_6\text{-rad}][\text{N-Cl}] = 0 \tag{29}$$

This indicates that there is no temporal change in the concentration of the **C6-rad** radical, which is sensible given that a steady-state is presumed for [N-rad]. Due to the mechanism and approximations used, a steady-state for the **C6-rad** radical is also obtained, as confirmed by the cancellation of $k_{2\text{f}}$ terms in eq 29, leading to no net change in [C_6-rad] over time. The formation of final product **C6-Cl** is described by eq 30.

$$\frac{d[\text{C}_6\text{-Cl}]}{dt} = k_{5\text{f}}[\text{C}_6\text{-rad}]_0[\text{N-Cl}] \tag{30}$$

Here, the notation [C_6-rad]$_0$ is used because the concentration of the **C6-rad** radical is not treated as a variable; it is assumed to be constant under the steady-state conditions. The expenditure of reactant N-Cl, shown in eq 31, is therefore pseudo-first-order reaction.

$$\frac{d[\text{N-Cl}]}{dt} = -k_{5\text{f}}[\text{C}_6\text{-rad}]_0[\text{N-Cl}] \tag{31}$$

This differential equation is trivial to solve by separation of variables and subsequent integration, as shown below.

$$\int_{[\text{N-Cl}]_0}^{[\text{N-Cl}](t)} \frac{d[\text{N-Cl}]}{[\text{N-Cl}]} = -k_{5\text{f}}[\text{C}_6\text{-rad}]_0 \int_0^t dt \tag{32}$$

$$[\text{N-Cl}](t) = [\text{N-Cl}]_0 e^{-k_{5\text{f}}[\text{C}_6\text{-rad}]_0 t} \tag{33}$$

Exponential decay is in accordance with the measurements obtained in photolysis experiments. The solution for time-dependent concentration of main product **C_6-Cl** is obtained as follows.

$$\frac{d[\mathrm{C_6\text{-}Cl}]}{dt} = k_{5\mathrm{f}}[\mathrm{C_6\text{-}rad}]_0[\mathrm{N\text{-}Cl}]_0 e^{-k_{5\mathrm{f}}[\mathrm{C_6\text{-}rad}]_0 t} \tag{34}$$

$$[\mathrm{C_6\text{-}Cl}](t) = k_{5\mathrm{f}}[\mathrm{C_6\text{-}rad}]_0[\mathrm{N\text{-}Cl}]_0 \int_0^t e^{-k_{5\mathrm{f}}[\mathrm{C_6\text{-}rad}]_0 t} dt \tag{35}$$

$$[\mathrm{C_6\text{-}Cl}](t) = [\mathrm{N\text{-}Cl}]_0\left(1 - e^{-k_{5\mathrm{f}}[\mathrm{C_6\text{-}rad}]_0 t}\right) \tag{36}$$

Now, we assume that the [N-Cl](t) concentration is primarily governed by the **C_6-Cl** pathway. The solutions for the kinetics involving the **C_2-Cl** and **C_5-Cl** pathways (secondary products) are derived under this assumption. The amounts of these secondary products are low (< 1%), while the main product, **C_6-Cl**, constitutes > 99% of the total products. Therefore, the reaction time is dictated by the formation of the main product, as it is the primary driver of reactant consumption, with all products originating from the same initial compound.

The kinetic model for the formation of **C_5-Cl** secondary product is described *via* eq 37 and eq 38, while the formation of the **C_2-Cl** product is described by eq 39 and eq 40. As before, the reverse reactions in eq 38 and eq 40 are not considered, based on the Gibbs energy diagrams (please see Figures 5-7), which indicate that these reverse reactions are unlikely to occur.

$$\mathrm{N-rad} \underset{k_{1\mathrm{b}}}{\overset{k_{1\mathrm{f}}}{\rightleftharpoons}} \mathrm{C_5\text{-}rad} \tag{37}$$

$$\mathrm{C_5\text{-}rad} + \mathrm{N\text{-}Cl} \xrightarrow{k_{3\mathrm{f}}} \mathrm{C_5\text{-}Cl} + \mathrm{N\text{-}rad} \tag{38}$$

$$\mathrm{N-rad} \underset{k_{2\mathrm{b}}}{\overset{k_{2\mathrm{f}}}{\rightleftharpoons}} \mathrm{C_6\text{-}rad} \tag{39}$$

$$\mathrm{C_6\text{-}rad} + \mathrm{N\text{-}Cl} \xrightarrow{k_{4\mathrm{f}}} \mathrm{C_2\text{-}Cl} + \mathrm{N\text{-}rad} \tag{40}$$

The differential equations corresponding to the kinetics of the proposed mechanism for the **C_5-Cl** product are provided below.

$$\frac{d[\text{N-rad}]}{dt} = -k_{1\text{f}}[\text{N-rad}] + k_{1\text{b}}[\text{C}_5\text{-rad}] + k_{3\text{f}}[\text{C}_5\text{-rad}][\text{N-Cl}] \quad (41)$$

$$\frac{d[\text{C}_5\text{-rad}]}{dt} = k_{1\text{f}}[\text{N-rad}] - k_{1\text{b}}[\text{C}_5\text{-rad}] - k_{3\text{f}}[\text{C}_5\text{-rad}][\text{N-Cl}] \quad (42)$$

$$\frac{d[\text{C}_5\text{-Cl}]}{dt} = k_{3\text{f}}[\text{C}_5\text{-rad}][\text{N-Cl}] \quad (43)$$

Given that [N-Cl] is affected primarily by the **C_6-rad** pathway, we use the time-dependent [N-Cl](t) concentration obtained previously in eq 33. By substituting the expression for [N-Cl] in the eqs 41-43, the following expressions are obtained.

$$\frac{d[\text{N-rad}]}{dt} = -k_{1\text{f}}[\text{N-rad}] + k_{1\text{b}}[\text{C}_5\text{-rad}] + k_{3\text{f}}[\text{C}_5\text{-rad}][\text{N-Cl}]_0 e^{-k_{5\text{f}}[\text{C}_6\text{-rad}]_0 t} \quad (44)$$

$$\frac{d[\text{C}_5\text{-rad}]}{dt} = k_{1\text{f}}[\text{N-rad}] - k_{1\text{b}}[\text{C}_5\text{-rad}] - k_{3\text{f}}[\text{C}_5\text{-rad}][\text{N-Cl}]_0 e^{-k_{5\text{f}}[\text{C}_6\text{-rad}]_0 t} \quad (45)$$

$$\frac{d[\text{C}_5\text{-Cl}]}{dt} = k_{3\text{f}}[\text{C}_5\text{-rad}][\text{N-Cl}]_0 e^{-k_{5\text{f}}[\text{C}_6\text{-rad}]_0 t} \quad (46)$$

Under the assumption of steady-state, the [N-rad] can be expressed as shown in eq 47.

$$[\text{N-rad}] = \frac{k_{1\text{b}}[\text{C}_5\text{-rad}] + k_{3\text{f}}[\text{C}_5\text{-rad}][\text{N-Cl}]_0 e^{-k_{5\text{f}}[\text{C}_6\text{-rad}]_0 t}}{k_{1\text{f}}} \quad (47)$$

The obtained steady-state expression for **N-rad** (eq 47) reflects the balance between its consumption in forming **C_5-rad** and its regeneration *via* the **C_5-Cl** pathway, with **C_6-rad** having no direct influence on the steady-state of **N-rad**. The role of **C_6-rad** is implicit in maintaining the overall balance of N through the **C_6-Cl** pathway, which permits an independent focus on the **C_5-rad** pathway. In a system where only **C_5-rad** and **C_6-rad** products are formed, steady-states for both **N-rad** and **C_6-rad** necessitate a steady-state for **C_5-rad**, as the sum of

[C_5-rad] and [C_6-rad] must remain constant to maintain the overall balance. For example, if **C_5-rad** concentration were to fluctuate while **C_6-rad** is at steady-state, then **N-rad** must also fluctuate (because it is linearly dependent).

Substituting the expression for [N-rad] given in eq 47 into eq 45 yields the following equation.

$$\frac{d[\mathrm{C_5\text{-}rad}]}{dt} = k_{1\mathrm{f}}\left(\frac{k_{1\mathrm{b}}[\mathrm{C_5\text{-}rad}]+k_{3\mathrm{f}}[\mathrm{C_5\text{-}rad}][\mathrm{N\text{-}Cl}]_0 e^{-k_{5\mathrm{f}}[\mathrm{C_6\text{-}rad}]_0 t}}{k_{1\mathrm{f}}}\right) - k_{1\mathrm{b}}[\mathrm{C_5\text{-}rad}] - k_{3\mathrm{f}}[\mathrm{C_5\text{-}rad}][\mathrm{N\text{-}Cl}]_0 e^{-k_{5\mathrm{f}}[\mathrm{C_6\text{-}rad}]_0 t} = 0 \qquad (48)$$

Expectingly, eq 48 shows that [C_5-rad] is invariant over time, indicating that it is at steady-state. This allows eq 46 to be solved analytically, by separation of variables and integration, as expressed below.

$$\int_0^{[\mathrm{C_5\text{-}Cl}](t)} d[\mathrm{C_5\text{-}Cl}] = k_{3\mathrm{f}}[\mathrm{C_5\text{-}rad}]_0[\mathrm{N\text{-}Cl}]_0 \int_0^t e^{-k_{5\mathrm{f}}[\mathrm{C_6\text{-}rad}]_0 t}\, dt \qquad (49)$$

$$[\mathrm{C_5\text{-}Cl}](t) = \frac{k_{3\mathrm{f}}[\mathrm{C_5\text{-}rad}]_0[\mathrm{N\text{-}Cl}]_0}{k_{5\mathrm{f}}[\mathrm{C_6\text{-}rad}]_0}\left(1 - e^{-k_{5\mathrm{f}}[\mathrm{C_6\text{-}rad}]_0 t}\right) \qquad (50)$$

In an analogous manner, the differential equations describing the **C_2-Cl** secondary pathway can be inferred from the model given in eq 39 and eq 40. The differential equations provided are derived under the assumption that the **C_6-Cl** pathway is the primary route for the consumption of **N-Cl** and **C_6-rad**. As a result, the rate constant $k_{5\mathrm{f}}$ is used to describe the decay of **N-Cl** over time in the **C_2-Cl** pathway.

$$\frac{d[\mathrm{N\text{-}rad}]}{dt} = -k_{2\mathrm{f}}[\mathrm{N\text{-}rad}] + k_{2\mathrm{b}}[\mathrm{C_6\text{-}rad}] + k_{5\mathrm{f}}[\mathrm{C_6\text{-}rad}][\mathrm{N\text{-}Cl}] \qquad (51)$$

$$\frac{d[\mathrm{C_6\text{-}rad}]}{dt} = k_{2\mathrm{f}}[\mathrm{N\text{-}rad}] - k_{2\mathrm{b}}[\mathrm{C_6\text{-}rad}] - k_{5\mathrm{f}}[\mathrm{C_6\text{-}rad}][\mathrm{N\text{-}Cl}] \qquad (52)$$

$$\frac{d[\mathrm{C_2\text{-}Cl}]}{dt} = k_{4\mathrm{f}}[\mathrm{C_6\text{-}rad}][\mathrm{N\text{-}Cl}] \qquad (53)$$

Again, the [N-Cl](t) is taken from eq 33, and the following expressions are obtained.

$$\frac{d[\text{C}_6\text{-rad}]}{dt} = k_{2\text{f}}[\text{N-rad}] - k_{2\text{b}}[\text{C}_6\text{-rad}] - k_{5\text{f}}[\text{C}_6\text{-rad}][\text{N-Cl}]_0 e^{-k_{5\text{f}}[\text{C}_6\text{-rad}]_0 t} \tag{54}$$

$$\frac{d[\text{C}_2\text{-Cl}]}{dt} = k_{4\text{f}}[\text{C}_6\text{-rad}][\text{N-Cl}]_0 e^{-k_{5\text{f}}[\text{C}_6\text{-rad}]_0 t} \tag{55}$$

Inputting the steady-state expression for N in eq 54 will again yield eq 29. Since a steady-state for **C_6-rad** is achieved, the concentration of the **C_6-rad** radical can be treated as a constant. Consequently, the temporal evolution of the **C_2-Cl** concentration can be determined by separating variables and integrating, as shown in the equations below.

$$\int_0^{[\text{C}_2\text{-Cl}](t)} d[\text{C}_2\text{-Cl}] = k_{4\text{f}}[\text{C}_6\text{-rad}]_0 \int_0^t [\text{N-Cl}]_0 e^{-k_{5\text{f}}[\text{C}_6\text{-rad}]_0 t} dt \tag{56}$$

$$[\text{C}_2\text{-Cl}](t) = \frac{k_{4\text{f}}[\text{N-Cl}]_0}{k_{5\text{f}}}\left(1 - e^{-k_{5\text{f}}[\text{C}_6\text{-rad}]_0 t}\right) \tag{57}$$

Analysis of product ratios and reaction half-lives

To calculate the reaction half-lives, the time $t_{1/2}$ is determined at which the concentration of each product reach half of its final equilibrium value. Since the concentrations of the intermediates **N-rad**, **C_5-rad**, and **C_6-rad** are assumed to be at steady-state, their half-lives cannot be calculated in the usual manner. Once the steady-state assumption is no longer valid, the system of differential equations becomes nonlinear and interdependent, making it analytically unsolvable. However, under the steady-state assumption, the half-lives of final products **C_6-Cl**, **C_5-Cl** and **C_2-Cl** may be estimated.

Firstly, it can be noted that the final expressions for the time dependencies of the product concentrations are all mathematically similar, given that the expenditure of the **N-Cl** reactant is assumed to be primarily driven by the main **C_6-Cl** reaction pathway. The general solution for all three products is given by the following equation.

$$[\text{product}](t) = c_{\max}(\text{product})\left(1 - e^{-k_{5\text{f}}[\text{C}_6\text{-rad}]_0 t}\right) \tag{58}$$

c_{max} represents the maximum concentration of the product, which can be determined by calculating the limit as time approaches infinity. This limit is shown in eq 59. As the exponent tends toward negative infinity, the exponential part of the equation approaches zero.

$$\lim_{t \to \infty} [\text{product}](t) = c_{\text{max}}(\text{product}) \tag{59}$$

Consequently, the approximated concentration of each product at the end of the reaction is equal to c_{max}(product), as shown in the equations below.

$$[\text{C}_6\text{-Cl}](t = \infty) \approx [\text{N-Cl}]_0 \tag{60}$$

$$[\text{C}_5\text{-Cl}](t = \infty) \approx \frac{k_{3\text{f}}[\text{C}_5\text{-rad}]_0[\text{N-Cl}]_0}{k_{5\text{f}}[\text{C}_6\text{-rad}]_0} \tag{61}$$

$$[\text{C}_2\text{-Cl}](t = \infty) \approx \frac{k_{4\text{f}}[\text{N-Cl}]_0}{k_{5\text{f}}} \tag{62}$$

The calculation suggests that the final concentration of **C_6-Cl** is equal to the initial concentration of **N-Cl**. This is an approximation made for modeling simplicity, implicitly assumed by the dominance of the **C_6-Cl** formation pathway. However, in reality, secondary reactions that produce **C_5-Cl** and **C_2-Cl** also consume **N-Cl**. Consequently, the actual concentration of **C_6-Cl** at the end of the reaction will be slightly lower than the initial **N-Cl** concentration. Although this discrepancy is minor, it should be acknowledged as a limitation of the model. Moreover, as time approaches infinity, the steady-state condition may no longer be fully satisfied, particularly as reactants are depleted and the system dynamics evolve. This potential deviation from steady-state could further contribute to the slight discrepancy between the modeled and actual final concentrations of **C_6-Cl** and other minor products.

It might be convenient to estimate the ratios of [C_5-Cl] and [C_2-Cl] to [C_6-Cl] to better understand the relative production rates of these products, highlighting the kinetic control of these reactions. These ratios are given in the following equations.

$$\frac{[\text{C}_5\text{-Cl}](t=\infty)}{[\text{C}_6\text{-Cl}](t=\infty)} \approx \frac{k_{3\text{f}}[\text{C}_5\text{-rad}]_0}{k_{5\text{f}}[\text{C}_6\text{-rad}]_0} \tag{63}$$

$$\frac{[\mathrm{C_2\text{-}Cl}](t=\infty)}{[\mathrm{C_6\text{-}Cl}](t=\infty)} \approx \frac{k_{4\mathrm{f}}}{k_{5\mathrm{f}}} \tag{64}$$

As the half-life is defined as the time it takes for the concentration of a product to reach half of its final value, the half-life of each final product in the studied system can be approximated. Due to the mathematical similarities in the expressions for the product concentrations, the half-life of any particular final product follows a general form, as showcased in eq 65.

$$t_{1/2} \approx \frac{\ln(2)}{k_{5\mathrm{f}}[\mathrm{C_6\text{-}rad}]_0} \tag{65}$$

The half-live expression in eq 65 was obtained as shown in the following equations.

$$[\mathrm{product}](t_{1/2}) = \frac{1}{2} c_{\mathrm{max}}(\mathrm{product}) = c_{\mathrm{max}}(\mathrm{product})\left(1 - e^{-k_{5\mathrm{f}}[\mathrm{C_6\text{-}rad}]_0 t_{1/2}}\right) \tag{66}$$

$$\frac{1}{2} = 1 - e^{-k_{5\mathrm{f}}[\mathrm{C_6\text{-}rad}]_0 t_{1/2}} \tag{67}$$

$$\ln\left(\frac{1}{2}\right) = -k_{5\mathrm{f}}[\mathrm{C_6\text{-}rad}]_0 t_{1/2} \tag{68}$$

$$t_{1/2} = \frac{\ln(2)}{k_{5\mathrm{f}}[\mathrm{C_6\text{-}rad}]_0} \tag{69}$$

According to the model approximations, the derived half-life expression (eq 69) showcases how the kinetics of a system can be dominated by a single, fast reaction pathway, thereby controlling the overall reaction time. This result, while consistent with the assumptions made in the model, reinforces the idea that the depletion of the **N-Cl** reactant by the primary pathway dictates the timing for the entire system. In other words, calculated (approximated) half-lives of secondary products are the same as the one for the primary product, even though those reactions are likely significantly slower. The uniformity of half-lives across different products is a direct consequence of the pseudo-first-order kinetics assumed in the model, where the product concentrations cancel out in the half-life calculation. This cancellation is specific to (pseudo-)first-order kinetics and would not generally occur in higher-order reactions, where the half-lives depend more directly on the varying concentrations of reactants and/or products.[46,47]

Estimation of maximum radical yield

To estimate the upper limit of radicals produced during laser photolysis experiments, the calculations were performed as detailed below.

First, the energy of a single photon can be estimated as shown in eq 70.

$$E_{\text{photon}} = \frac{hc}{\lambda} \tag{70}$$

h is Planck's constant; *c* is the speed of light and λ is the wavelength of the photolytic radiation. Since the lowest wavelength corresponds to the highest energy, the value λ = 266 nm was used for the calculation as it represents the lowest excitation wavelength used in the experiments.

$$E_{\text{photon}} = \frac{6.626 \cdot 10^{-34}\ \text{Js} \cdot 3 \cdot 10^{8}\ \text{m/s}}{266 \cdot 10^{-9}\ \text{m}} \approx 7.468 \cdot 10^{-19}\ \text{J} \tag{71}$$

The number of photons per pulse can be estimated by dividing the pulse energy ($E_{\text{pulse}} \geq 22 \cdot 10^{-3}$J) by the energy of a single photon, as shown in eq 72.

$$N_{\text{photons}} = \frac{E_{\text{pulse}}}{E_{\text{photon}}} \tag{72}$$

Substituting numerical values produces the following result (eq 73).

$$N_{\text{photons}} = \frac{22 \cdot 10^{-3}\ \text{J}}{7.468 \cdot 10^{-19}\ \text{J}} \approx 2.946 \times 10^{16} \tag{73}$$

A fraction of photons absorbed in the solution (f_{abs}) can be calculated utilizing eq 74.

$$f_{\text{abs}} = 1 - 10^{-A} \tag{74}$$

For the absorbance values utilized in the experiments ($A \approx 0.3$), the numerical results are provided in eq 75.

$$f_{\text{abs}} = 1 - 10^{-0.3} \approx 0.499 \tag{75}$$

It can be noted that approximately half of the laser-emitted photons are not absorbed in the solution; hence, this fraction does not contribute to the photolytic reaction. Consequently, the number of absorbed photons can be calculated by scaling the total number of photons per pulse with the f_{abs} factor, as given in eq 76.

$$N_{\text{photons, abs}} = N_{\text{photons}} \cdot f_{\text{abs}} \tag{76}$$

Inputting numerical values in the previous equation gives the following result provided in eq 77.

$$N_{\text{photons, abs}} \approx 2.946 \cdot 10^{16} \cdot 0.499 \approx 1.469 \cdot 10^{16} \tag{77}$$

The volume in which the radicals are generated is determined by the beam cross-sectional area and the path length of the beam in the solution. For the calculation of the cross-sectional area, beam diameter value of 6.5 mm was used.[48] The beam radius, *r*, is half of the diameter. Moreover, it should be noted that the decrease in the beam's intensity due to divergence was not included in the calculation. Including this effect would further decrease the estimated number of radicals formed *via* photolysis. The third dimension required for volume calculation, the optical path length, is l = 1 cm. The expression for this volume of interaction is given in eq 9 and the numerical results are provided in eq 79.

$$V = \sigma \times l = \pi r^2 l \tag{78}$$

$$V = \pi(0.325\ \text{cm})^2 \cdot 1\ \text{cm} = 0.332\ \text{cm}^3 = 0.332 \cdot 10^{-3}\ \text{L} \tag{79}$$

σ is the cross-section surface area.

The estimation of the maximum volume concentration of radicals can be obtained using eq 80.

$$c_{\text{radicals}} = \frac{N_{\text{photons, abs}} \cdot \Phi}{N_{\text{A}} \cdot V} \tag{80}$$

N_{A} is Avogadro's constant and Φ is the quantum yield. Due to the lack of literature data regarding the compound's quantum yields, we employ the most conservative upper estimate

with $\Phi = 1$. In reality, this value is likely significantly lower, resulting in lower upper estimates for the maximum radical concentration. The numerical results are given in eq 81.

$$c_{\text{radicals}} = \frac{1.469\times10^{16}\cdot1}{6.022\times10^{23}\ \text{mol}^{-1}\cdot0.332\cdot10^{-3}\ \text{L}} = 7.353\cdot10^{-5}\text{molL}^{-1} \tag{81}$$

For the initial compound concentration of $c_{\text{compound, initial}} = 3.000\cdot10^{-4}\ \text{molL}^{-1}$, the maximum relative percentage of radicals can be calculated as shown below.

$$\%_{\text{radicals}} = \frac{c_{\text{radicals}}}{c_{\text{compound, initial}}} \tag{82}$$

$$\%_{\text{radicals}} = \frac{7.353\cdot10^{-5}\text{molL}^{-1}}{(3.000\cdot10^{-4}-7.353\cdot10^{-5})\text{molL}^{-1}} = 32.47\% \tag{83}$$

Another important consideration is the effect of self-absorption. As the excitation beam passes through the absorptive sample, its intensity decreases, resulting in a non-uniform distribution of formed radicals. Consequently, the highest concentration of radicals is expected to form at the interface between the cuvette and the solution, where the light intensity is greatest.

S13. References

(1) Willcott, M. R. MestRe Nova. *J. Am. Chem. Soc.* **2009**, *131* (36), 13180–13180. https://doi.org/10.1021/ja906709t.

(2) Stoll, S.; Schweiger, A. EasySpin, a Comprehensive Software Package for Spectral Simulation and Analysis in EPR. *Journal of Magnetic Resonance* **2006**, *178* (1), 42–55. https://doi.org/10.1016/j.jmr.2005.08.013.

(3) Šakić, D. DSakicLab/visualEPR, 2023. https://github.com/DSakicLab/visualEPR (accessed 2024-10-30).

(4) Https://Www.Kessil.Com/Downloads/Science/PR160L_Intensity_Map_2.Pdf.

(5) Pracht, P.; Bohle, F.; Grimme, S. Automated Exploration of the Low-Energy Chemical Space with Fast Quantum Chemical Methods. *Phys. Chem. Chem. Phys.* **2020**, *22* (14), 7169–7192. https://doi.org/10.1039/C9CP06869D.

(6) Bannwarth, C.; Caldeweyher, E.; Ehlert, S.; Hansen, A.; Pracht, P.; Seibert, J.; Spicher, S.; Grimme, S. Extended TIGHT□BINDING Quantum Chemistry Methods. *WIREs Comput Mol Sci* **2021**, *11* (2), e1493. https://doi.org/10.1002/wcms.1493.

(7) Bannwarth, C.; Ehlert, S.; Grimme, S. GFN2-xTB—An Accurate and Broadly Parametrized Self-Consistent Tight-Binding Quantum Chemical Method with Multipole Electrostatics and Density-Dependent Dispersion Contributions. *J. Chem. Theory Comput.* **2019**, *15* (3), 1652–1671. https://doi.org/10.1021/acs.jctc.8b01176.

(8) Becke, A. D. Density-Functional Thermochemistry. III. The Role of Exact Exchange. *The Journal of Chemical Physics* **1993**, *98* (7), 5648–5652. https://doi.org/10.1063/1.464913.

(9) Stephens, P. J.; Devlin, F. J.; Chabalowski, C. F.; Frisch, M. J. Ab Initio Calculation of Vibrational Absorption and Circular Dichroism Spectra Using Density Functional Force Fields. *J. Phys. Chem.* **1994**, *98* (45), 11623–11627. https://doi.org/10.1021/j100096a001.

(10) Ditchfield, R.; Hehre, W. J.; Pople, J. A. Self-Consistent Molecular-Orbital Methods. IX. An Extended Gaussian-Type Basis for Molecular-Orbital Studies of Organic Molecules. *The Journal of Chemical Physics* **1971**, *54* (2), 724–728. https://doi.org/10.1063/1.1674902.

(11) Marenich, A. V.; Cramer, C. J.; Truhlar, D. G. Universal Solvation Model Based on Solute Electron Density and on a Continuum Model of the Solvent Defined by the Bulk Dielectric Constant and Atomic Surface Tensions. *J. Phys. Chem. B* **2009**, *113* (18), 6378–6396. https://doi.org/10.1021/jp810292n.

(12) Mennucci, B.; Tomasi, J.; Cammi, R.; Cheeseman, J. R.; Frisch, M. J.; Devlin, F. J.; Gabriel, S.; Stephens, P. J. Polarizable Continuum Model (PCM) Calculations of Solvent Effects on Optical Rotations of Chiral Molecules. *J. Phys. Chem. A* **2002**, *106* (25), 6102–6113. https://doi.org/10.1021/jp020124t.

(13) Grimme, S. Semiempirical Hybrid Density Functional with Perturbative Second-Order Correlation. *The Journal of Chemical Physics* **2006**, *124* (3), 034108. https://doi.org/10.1063/1.2148954.

(14) Neese, F.; Schwabe, T.; Grimme, S. Analytic Derivatives for Perturbatively Corrected “Double Hybrid” Density Functionals: Theory, Implementation, and Applications. *The Journal of Chemical Physics* **2007**, *126* (12), 124115. https://doi.org/10.1063/1.2712433.

(15) Curtiss, L. A.; Redfern, P. C.; Raghavachari, K.; Rassolov, V.; Pople, J. A. Gaussian-3 Theory Using Reduced Mo/Ller-Plesset Order. *The Journal of Chemical Physics* **1999**, *110* (10), 4703–4709. https://doi.org/10.1063/1.478385.

(16) Grimme, S.; Antony, J.; Ehrlich, S.; Krieg, H. A Consistent and Accurate *Ab Initio* Parametrization of Density Functional Dispersion Correction (DFT-D) for the 94 Elements H-Pu. *The Journal of Chemical Physics* **2010**, *132* (15), 154104. https://doi.org/10.1063/1.3382344.

(17) Vrček, I. V.; Šakić, D.; Vrček, V.; Zipse, H.; Biruš, M. Computational Study of Radicals Derived from Hydroxyurea and Its Methylated Analogues. *Org. Biomol. Chem.* **2012**, *10* (6), 1196–1206. https://doi.org/10.1039/C1OB06594G.

(18) Hermosilla, L.; Calle, P.; García De La Vega, J. M.; Sieiro, C. Density Functional Theory Study of ^{14} N Isotropic Hyperfine Coupling Constants of Organic Radicals. *J. Phys. Chem. A* **2006**, *110* (50), 13600–13608. https://doi.org/10.1021/jp064900z.
(19) Frisch, M. J.; Trucks, G. W.; Schlegel, H. B.; Scuseria, G. E.; Robb, M. A.; Cheeseman, J. R.; Scalmani, G.; Barone, V.; Petersson, G. A.; Nakatsuji, H.; et al. Gaussian 16, Revision C. 01. Gaussian, Inc.: Wallingford CT, 2016.
(20) HR-ZOO, Cluster Supek; University of Zagreb University Computing Centre SRCE. KK.01.1.1.08.0001, EU Funded within OPCC for Republic of Croatia: Zagreb, 2023.
(21) PharmInova Project, Cluster Sw.Pharma.Hr. University of Zagreb Faculty of Pharmacy and Biochemistry. KK.01.1.1.02.0021, EU Funded by the European Regional Development Fund: Zagreb 2023.).
(22) Bauernschmitt, R.; Häser, M.; Treutler, O.; Ahlrichs, R. Calculation of Excitation Energies within Time-Dependent Density Functional Theory Using Auxiliary Basis Set Expansions. *Chemical Physics Letters* **1997**, *264* (6), 573–578. https://doi.org/10.1016/S0009-2614(96)01343-7.
(23) Yanai, T.; Tew, D. P.; Handy, N. C. A New Hybrid Exchange–Correlation Functional Using the Coulomb-Attenuating Method (CAM-B3LYP). *Chemical Physics Letters* **2004**, *393* (1–3), 51–57. https://doi.org/10.1016/j.cplett.2004.06.011.
(24) Ardura, D.; López, R.; Sordo, T. L. Relative Gibbs Energies in Solution through Continuum Models: Effect of the Loss of Translational Degrees of Freedom in Bimolecular Reactions on Gibbs Energy Barriers. *J. Phys. Chem. B* **2005**, *109* (49), 23618–23623. https://doi.org/10.1021/jp0540499.
(25) Šakić, D.; Šonjić, P.; Tandarić, T.; Vrček, V. Chlorination of *N* -Methylacetamide and Amide-Containing Pharmaceuticals. Quantum-Chemical Study of the Reaction Mechanism. *J. Phys. Chem. A* **2014**, *118* (12), 2367–2376. https://doi.org/10.1021/jp5012846.
(26) *Frontiers | A modern analytic method to solve singular and non-singular linear and non-linear differential equations*. https://www.frontiersin.org/journals/physics/articles/10.3389/fphy.2023.1167797/full (accessed 2024-10-30).
(27) Cheng, C. M.; Peng, Z. K.; Zhang, W. M.; Meng, G. Volterra-Series-Based Nonlinear System Modeling and Its Engineering Applications: A State-of-the-Art Review. *Mechanical Systems and Signal Processing* **2017**, *87*, 340–364. https://doi.org/10.1016/j.ymssp.2016.10.029.
(28) Gavalas, G. R. *Nonlinear Differential Equations of Chemically Reacting Systems*; Coleman, B. D., Aris, R., Collatz, L., Ericksen, J. L., Germain, P., Gurtin, M. E., Schiffer, M. M., Sternberg, E., Truesdell, C., Series Eds.; Springer Tracts in Natural Philosophy; Springer Berlin Heidelberg: Berlin, Heidelberg, 1968; Vol. 17. https://doi.org/10.1007/978-3-642-87643-1.
(29) *Pachpatte, B. G. Nonlinear Integral Inequalities I. In Mathematics in Science and Engineering; Elsevier, 1998; Vol. 197, Pp 99–220.*
(30) Wang, W.-S.; Li, S.-F.; Su, K. Nonlinear Stability of Runge–Kutta Methods for Neutral Delay Differential Equations. *Journal of Computational and Applied Mathematics* **2008**, *214* (1), 175–185. https://doi.org/10.1016/j.cam.2007.02.031.
(31) Zennaro, M. Asymptotic Stability Analysis of Runge-Kutta Methods for Nonlinear Systems of Delay Differential Equations. *Numerische Mathematik* **1997**, *77* (4), 549–563. https://doi.org/10.1007/s002110050300.
(32) Iserles, A. Stability and Dynamics of Numerical Methods for Nonlinear Ordinary Differential Equations. *IMA J Numer Anal* **1990**, *10* (1), 1–30. https://doi.org/10.1093/imanum/10.1.1.
(33) Ricardo, H. J. Systems of Nonlinear Differential Equations. In *A Modern Introduction to Differential Equations*; Elsevier, 2021; pp 361–420. https://doi.org/10.1016/B978-0-12-818217-8.00014-2.
(34) El-Ajou, A.; Al-ghananeem, H.; Saadeh, R.; Qazza, A.; Oqielat, M. N. A Modern Analytic Method to Solve Singular and Non-Singular Linear and Non-Linear Differential Equations. *Front. Phys.* **2023**, *11*, 1167797. https://doi.org/10.3389/fphy.2023.1167797.
(35) Cheng, C. M.; Peng, Z. K.; Zhang, W. M.; Meng, G. Volterra-Series-Based Nonlinear System Modeling and Its Engineering Applications: A State-of-the-Art Review. *Mechanical Systems and Signal Processing* **2017**, *87*, 340–364. https://doi.org/10.1016/j.ymssp.2016.10.029.

(36) Gavalas, G. R. *Nonlinear Differential Equations of Chemically Reacting Systems*; Coleman, B. D., Aris, R., Collatz, L., Ericksen, J. L., Germain, P., Gurtin, M. E., Schiffer, M. M., Sternberg, E., Truesdell, C., Series Eds.; Springer Tracts in Natural Philosophy; Springer Berlin Heidelberg: Berlin, Heidelberg, 1968; Vol. 17. https://doi.org/10.1007/978-3-642-87643-1.
(37) Pachpatte, B. G. Nonlinear Integral Inequalities I. In *Mathematics in Science and Engineering*; Elsevier, 1998; Vol. 197, pp 99–220. https://doi.org/10.1016/S0076-5392(98)80004-0.
(38) Wang, W.-S.; Li, S.-F.; Su, K. Nonlinear Stability of Runge–Kutta Methods for Neutral Delay Differential Equations. *Journal of Computational and Applied Mathematics* **2008**, *214* (1), 175–185. https://doi.org/10.1016/j.cam.2007.02.031.
(39) Zennaro, M. Asymptotic Stability Analysis of Runge-Kutta Methods for Nonlinear Systems of Delay Differential Equations. *Numerische Mathematik* **1997**, *77* (4), 549–563. https://doi.org/10.1007/s002110050300.
(40) Iserles, A. Stability and Dynamics of Numerical Methods for Nonlinear Ordinary Differential Equations. *IMA J Numer Anal* **1990**, *10* (1), 1–30. https://doi.org/10.1093/imanum/10.1.1.
(41) Ricardo, H. J. Systems of Nonlinear Differential Equations. In *A Modern Introduction to Differential Equations*; Elsevier, 2021; pp 361–420. https://doi.org/10.1016/B978-0-12-818217-8.00014-2.
(42) Wang, S.; Hofmann, H. Strategies and Methods for the Investigation of Chemical Reaction Kinetics. *Chemical Engineering Science* **1999**, *54* (11), 1639–1647. https://doi.org/10.1016/S0009-2509(98)00449-7.
(43) Kozuch, S. Steady State Kinetics of Any Catalytic Network: Graph Theory, the Energy Span Model, the Analogy between Catalysis and Electrical Circuits, and the Meaning of "Mechanism." *ACS Catal.* **2015**, *5* (9), 5242–5255. https://doi.org/10.1021/acscatal.5b00694.
(44) Perez-Benito, J. F. Some Considerations on the Fundamentals of Chemical Kinetics: Steady State, Quasi-Equilibrium, and Transition State Theory. *J. Chem. Educ.* **2017**, *94* (9), 1238–1246. https://doi.org/10.1021/acs.jchemed.6b00957.
(45) Jørgensen, L. V.; Madsen, H. L.; Thomsen, M. K.; Dragsted, L. O.; Skibsted, L. H. Regeneration of Phenolic Antioxidants from Phenoxyl Radicals: An ESR and Electrochemical Study of Antioxidant Hierarchy. *Free Radical Research* **1999**, *30* (3), 207–220. https://doi.org/10.1080/10715769900300231.
(46) Meagher, N. E.; Rorabacher, D. B. Mathematical Treatment for Very Rapid Second-Order Reversible Kinetics As Measured by Stopped-Flow Spectrophotometry with Corrections for the Cell Concentration Gradient. *J. Phys. Chem.* **1994**, *98* (48), 12590–12593. https://doi.org/10.1021/j100099a022.
(47) Hladky, P. W. Chemical Dosing and First-Order Kinetics. *J. Chem. Educ.* **2011**, *88* (6), 776–781. https://doi.org/10.1021/ed100147p.
(48) http://www.lumibird.cn/uploads/file/20201218/20201218113604_67241.pdf (accessed 2024-10-30)
